# The diversification of PHIS transposon superfamily in eukaryotes

**DOI:** 10.1186/s13100-015-0043-7

**Published:** 2015-06-24

**Authors:** Min-Jin Han, Chu-Lin Xiong, Hong-Bo Zhang, Meng-Qiang Zhang, Hua-Hao Zhang, Ze Zhang

**Affiliations:** School of Life Sciences, Chongqing University, Chongqing, 400044 China; College of Pharmacy and Life Science, Jiujiang University, Jiujiang, 332000 China

**Keywords:** Transposable elements, PHIS, Diversification, Identification

## Abstract

**Background:**

PHIS transposon superfamily belongs to DNA transposons and includes *PIF/Harbinger*, *ISL2EU*, and *Spy* transposon groups. These three groups have similar DDE domain-containing transposases; however, their coding capacity, species distribution, and target site duplications (TSDs) are significantly different.

**Results:**

In this study, we systematically identified and analyzed PHIS transposons in 836 sequenced eukaryotic genomes using transposase homology search and structure approach. In total, 380 PHIS families were identified in 112 genomes and 168 of 380 families were firstly reported in this study. Besides previous identified *PIF/Harbinger*, *ISL2EU*, and *Spy* groups, three new types (called *Pangu*, *NuwaI*, and *NuwaII*) of PHIS superfamily were identified; each has its own distinctive characteristics, especially in TSDs. *Pangu* and *NuwaII* transposons are characterized by 5′-ANT-3′ and 5′-C|TNA|G-3′ TSDs, respectively. Both transposons are widely distributed in plants, fungi, and animals; the *NuwaI* transposons are characterized by 5′-CWG-3′ TSDs and mainly distributed in animals.

**Conclusions:**

Here, in total, 380 PHIS families were identified in eukaryotes. Among these 380 families, 168 were firstly reported in this study. Furthermore, three new types of PHIS superfamily were identified. Our results not only enrich the transposon diversity but also have extensive significance for improving genome sequence assembly and annotation of higher organisms.

**Electronic supplementary material:**

The online version of this article (doi:10.1186/s13100-015-0043-7) contains supplementary material, which is available to authorized users.

## Background

Transposable elements (TEs) are fragments of DNA that can move from one site to another in a genome [1, 2]. TEs are classified into two classes (class 1 and class 2) according to their mechanism of transposition. The transposition mechanism of class 1 elements can be described as copy-and-paste mode, whereas class 2 transposons can be transposed by cut-and-paste mechanism. Recently, more and more genome sequencing revealed that TEs constitute the largest components of most eukaryotic genomes [2–13]. TEs not only have significant impact on the evolution of the host genomes and biological complexity but also are challenges for host genome sequencing, assembly, and annotation due to their repeatability. Thus, the knowledge about TEs characteristics and categories will promote the development of genomics.

In the past decade, many studies focused on identification, annotation, and function of TEs. So far, huge amounts of TEs have been identified and annotated. For example, 42 class 1 superfamilies and 19 class 2 superfamilies were annotated and cataloged in the RepBase database. However, the number of reported TEs could be just the tip of the iceberg. There are a larger number of TEs to be annotated due to their great diversification. For instance, 658 families were classified into unknown TEs in the silkworm; 163 unknown TE families in the maize and about 0.38 % of mouse genome sequences are unknown TEs [12–14]. Thus, the work of identification and annotation of TEs is far from finished.

Recently, we have identified a new group of cut-and-paste transposons designated as *Spy* [15]. *Spy* transposons are distinct from all other groups of DNA transposons by their strong insertion preference within the AAATTT motif and the lack of target site duplications (TSDs) upon insertion. In addition, we showed that *PIF/Harbinger*, *ISL2EU*, and *Spy* are evolutionarily related and share a preference for insertion into AT-rich target sequences [15]. For instance, the *ISL2EU* transposons are characterized by 5′-AT-3′ TSDs and the *PIF/Harbinger* transposons by 5′-TWA-3′ [16, 17]. Thus, these three groups *PIF/Harbinger*, *ISL2EU*, and *Spy* were classified into the same superfamily that is designated as “PHIS”. The PHIS transposon superfamily is high polymorphism in the target sequences, coding capacity, and conserved motifs of transposase [15]. It is common to find some distinct groups within a given superfamily. Previously, variable nucleotide composition and length of TSDs were found in some superfamilies [16–18]. However, the detailed diversification of PHIS transposon superfamily still remains unclear.

Here, we systematically identified and analyzed PHIS transposons in 836 sequenced eukaryotic genomes using transposase homology search combined with structure approach. Totally, 380 PHIS families including 212 previously reported families and 168 unpublished families were identified in this study. The 380 PHIS families are classified into six groups including three previously reported groups (*PIF/Harbinger*, *ISL2EU*, and *Spy*) and three new groups, called *Pangu*, *NuwaI*, and *NuwaII*. Each new group has its own particular characteristics, especially in TSDs.

## Results

### The landscape of PHIS transposons in eukaryotic genomes

To investigate the detailed diversification and evolution of PHIS superfamily in eukaryotes, we systematically identified and analyzed the characteristics and distribution of PHIS transposons in 836 eukaryotic genomes using transposase homology search and structure approach. Finally, we identified 380 PHIS transposon families. Furthermore, each of the PHIS consensus sequence defined in this study was subject to homology search against RepBase (as of October 20, 2014) and National Center for Biotechnology Information (NCBI) non-redundant (nr) nucleotide database using Censor and BlastN program. The results of these searches showed that 168 of 380 PHIS families were not reported, and other TEs (212) had been released and cataloged in RepBase, NCBI, or published papers [15].

**Fig. 1:**
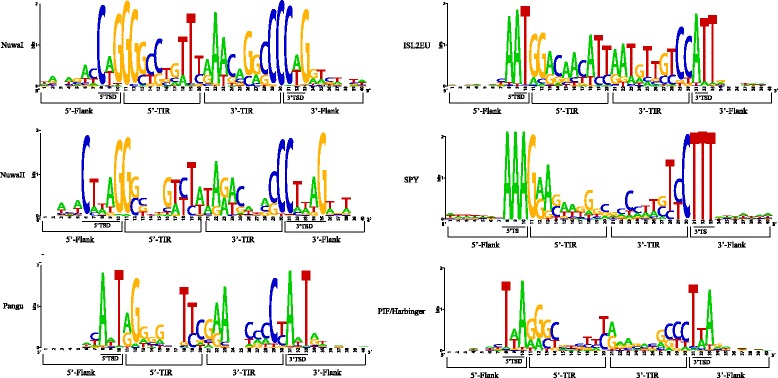
Sequence logos of TIRs (10 bp) and TSDs (10 bp) for each PHIS group. The TIRs and TSDs are *underlined*. These TIRs and TSDs sequences are derived from all full-length copies of all species. The individual with both complete TIRs was regarded as a full-length copy

**Fig. 2:**
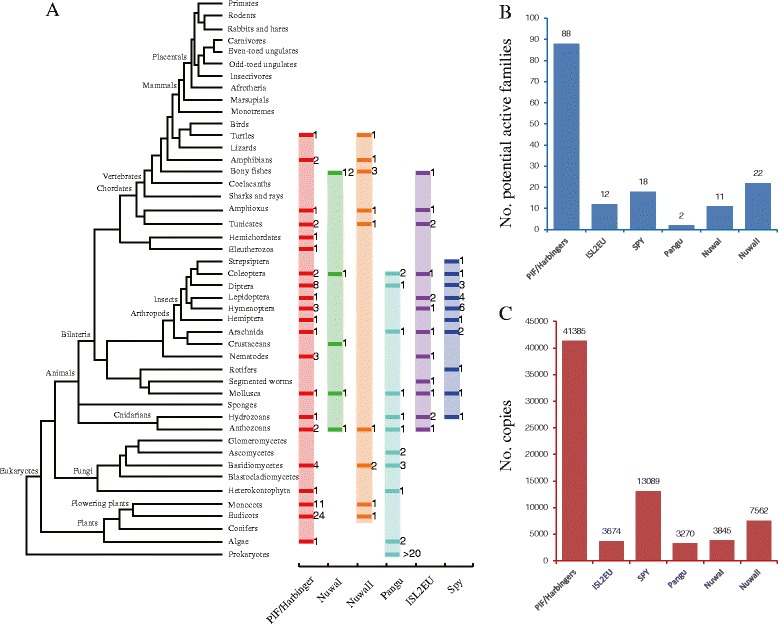
Distribution, abundance, and potential active families of PHIS transposons. **a** Taxonomic distribution of PHIS transposon groups across the eukaryotic tree of life. *Different colored boxes* indicate presence of the corresponding group, and flanking *numbers* represent the number of species. **b** The potential active families of each group in the eukaryotes. **c** The number of copies of each group in the eukaryotes

Based on the characteristics (TSDs, coding capacity, and secondary structure of transposase, etc.) of these 380 families, we found that 214 families belong to the *PIF/Harbinger* transposon group (Additional file 1: Table S1). Among the 214 families, 80 families had been previously identified and cataloged in RepBase, and 134 families were firstly identified in this study. These 214 families shared the following characteristics. (1) The TSD sequence is 5′-TWA-3′ tri-nucleotide (‘W’ represents A or T nucleotide) (Fig. 1). (2) Most candidate autonomous elements contain two open reading frames (ORFs), one ORF encoding the DDE and helix-turn-helix (HTH) motif-containing transposase and the other ORF encoding a DNA-binding protein with a Myb/SANT domain. The potential active families of PIF/Harbinger group were defined as those including both two intact ORFs. Finally, we identified 88 potential active families in the eukaryotic genomes (Additional file 1: Table S1 and Fig. 2b). (3) The TIR (terminal inverted repeat) lengths of different PIF/Harbinger families are highly variable (5–1042 bp), but the lengths of most TIRs (~93 %) are less than 60 bp, and the first nucleotide of TIRs is usually A or G (Fig. 1). (4) The average length of consensus sequences of candidate autonomous is ~4124 bp. (5) These families are distributed in 75 species including plants, fungi, and animals. The above-described characteristics of *PIF/Harbinger* transposons are consistent with previous reports [15, 16, 19].

Meanwhile, 25 families belong to *ISL2EU* group. Among these 25 families, 8 families were firstly identified in this study. The others had been cataloged in RepBase (Additional file 1: Table S2). These families shared the following characteristics. (1) The TSDs are 5′-AT-3′ di-nucleotide; however, there is a conserved single A nucleotide in the flank of 5′ terminal of TSDs and a conserved single T nucleotide in the flank of 3′ terminal of TSDs (Additional file 2: Figure S1). Thus, we speculated that the target site sequence of *ISL2EU* transposons is A|AT|T (where ‘|’ marks the cut site), the analysis of paralogous empty sites further confirmed the target site sequence of *ISL2EU*. Additional file 2: Figure S2 shows the possible generation mechanism of this TSDs. (2) Most autonomous candidate transposons of *ISL2EU* contain two ORFs, one ORF encoding the DDE, HTH, and THAP domain-containing transposase, the other ORF encoding a DNA-binding protein with a YqaJ exonuclease domain. Similar to a standard mentioned before, TEs with two intact ORFs are defined as the potential active transposons. Thus, 12 potential active families of *ISL2EU* group were identified in the eukaryotic genomes (Additional file 1: Table S2 and Fig. 2b) (3). The TIR length ranges from 6 to 259 bp, and the first two nucleotides of TIRs are usually “GG” di-nucleotide (Fig. 1). (4) The average length of consensus sequences of autonomous elements is ~4840 bp. (5) These families are distributed in 14 species. All these species belong to animals.

In this study, we found 54 families that belong to the *Spy* transposons; however, we did not identify any new *Spy* transposon family. All these families have been identified in previous study, and the characteristics of *Spy* transposons were also shown previously [15]. Besides the above three identified PHIS groups (*PIF/Harbinger*, *ISL2EU*, and *Spy*), we also found three new types of PHIS transposons distinct from the previous PHIS transposons in TSDs, and these new types transposons are called *Pangu*, *NuwaI*, and *NuwaII*, respectively.

### Characterization and distribution of *Pangu* transposons

**Fig. 3:**
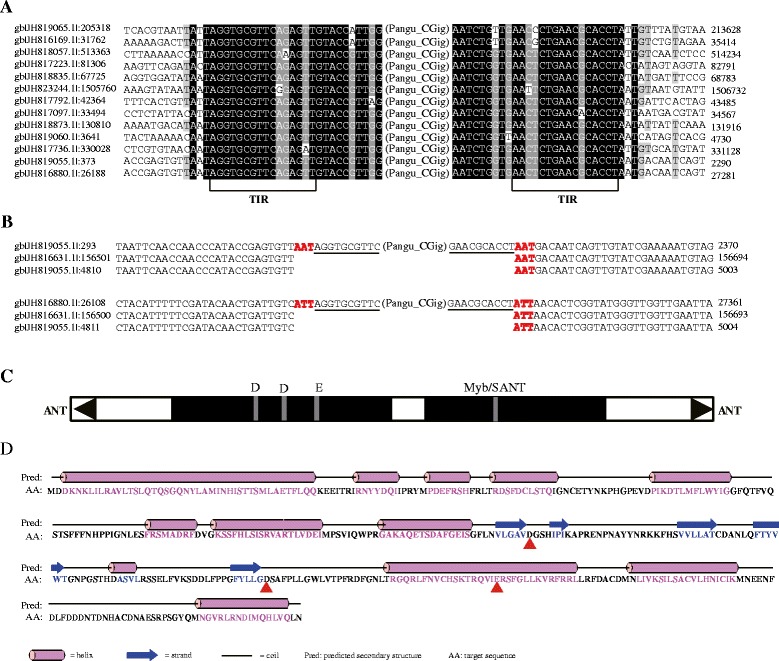
Characteristics of *Pangu* transposons. **a** Sequence alignments for *Pangu_CGig* family. The terminal inverted repeats (TIRs) and flanking sequences (10 bp) are shown. **b** Two examples of alignments of the flanking sequences of *Pangu_CGig* insertions with a paralogous sequences found within the same genome but devoid of the transposon. The TIRs of the element are *underlined*. **c** Structure of *Pangu_CGig. Black triangles* and *solid black boxes* represent the TIRs and ORFs, respectively, and the position of the DDE triad is shown. **d** Predicted secondary structure of the DDE motif-containing transposase of the *Pangu_CGig*. The DDE triads is marked with *red triangles* below the sequence

Thirty four *Pangu* families were identified in this study (Additional file 1: Table S3). The length of TIRs in these families varies from 11 to 40 bp, and the first two nucleotides of TIRs are usually “AG” and “GG” di-nucleotide (Fig. 1). The average consensus sequence length of autonomous candidates is ~3487 bp. Most autonomous candidates of *Pangu* transposon contain two ORFs, one ORF encoding the DDE motif-containing transposase and without any other domains. Meanwhile, we did not detect any known motifs in the other ORF. Given that the potential active families should contain the two intact ORFs, we identified two potential active families of *Pangu* group in the eukaryotic genomes (Additional file 1: Table S3 and Fig. 2b). Secondary structure prediction of *Pangu* DDE-containing transposases suggests that the first D is located between two beta-sheets, the second D is located between a beta-sheet and an alpha-helix, and the last E is present within an alpha-helix (Fig. 3). This result is consistent with the eukaryotic *PIF/Harbinger* and *ISL2EU* transposons [15]. The results of paralogous empty site confirmed that the TSDs of these families are 5′-ANT-3′ (‘N’ represents A, T, C, or G nucleotide) (Fig. 3). This characteristic of TSDs is significantly different from eukaryotic *PIF/Harbinger*, *ISL2EU*, and *Spy* transposons but consistent with the bacterial *IS5* transposons. Thus, both *Pangu* and *IS5* transposons could belong to the same group or were derived from the same ancient element.

These 34 *Pangu* transposons are distributed in 15 eukaryotic genomes. These species include two coleopterans, one dipteran, one arachnidan, one molluscan, one hydrozoan, one anthozoan, two ascomycetes, three basidiomycetes, one heterokontophyta, and two algae (Fig. 2). And these species are widely distributed in plants, fungi, and animals. Thus, the *Pangu* transposons could be ancient elements in the eukaryotic genomes. To estimate the abundance of *Pangu* transposons in the eukaryotic genomes, the consensus sequence of each family of *Pangu* was used as query in BlastN (*e* < 10^−5^) search against the corresponding genome. A copy for the same family was defined by *e* value less than *e*^−5^, length larger than 50 bp, and nucleotide identity larger than 80 %. Finally, we identified 3270 copies of *Pangu* group in the eukaryotic genomes (Additional file 1: Table S3, Additional file 3: Table S4, and Fig. 2c).

### Characterization and distribution of *NuwaI* transposons

**Fig. 4:**
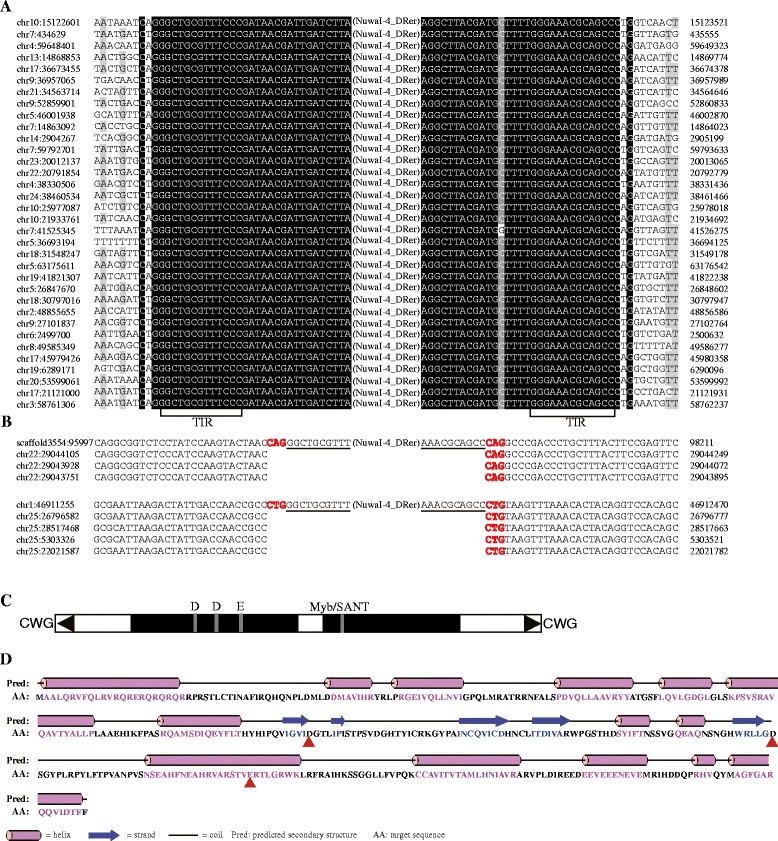
Characteristics of *NuwaI* transposons. **a** Sequence alignments for *NuwaI-4_DRer* family. The terminal inverted repeats (TIRs) and flanking sequences (10 bp) are shown. **b** Two examples of alignments of the flanking sequences of *NuwaI-4_DRer* insertions with a paralogous sequences found within the same genome but devoid of the transposon. The TIRs of the element are *underlined*. **c** Structure of *NuwaI-4_DRer. Black triangles* and *solid black boxes* represent the TIRs and ORFs, respectively, and the position of the DDE triad is shown. **d** Predicted secondary structure of the DDE motif-containing transposase of the *NuwaI-4_DRer*. The DDE triads is marked with *red triangles* below the sequence

Twenty-three *NuwaI* families were identified in this study (Additional file 1: Table S5). The results of paralogous empty site confirmed that the TSDs of these families are 5′-CWG-3′ (‘W’ represents A or T nucleotide) (Fig. 4). This characteristic is significantly different from previously the identified *PIF/Harbinger*, *ISL2EU*, and *Spy* transposons (AT-rich TSDs). Most autonomous candidates of *NuwaI* transposons contain two ORFs, one ORF encoding the DDE motif-containing transposase and without any other domain, the other ORF encoding a DNA-binding protein with a Myb/SANT domain. We identified 11 potential active families in the eukaryotic genomes because these TEs contain the two intact ORFs (Additional file 1: Table S5 and Fig. 2b). The secondary structure of *NuwaI* transposase is very similar to the *PIF/Harbinger*, *ISL2EU*, and *Pangu* transposases. For instance, the first D is located between two beta-sheets, the second D is typically between a beta-sheet and an alpha-helix, and the last E occurs within an alpha-helix (Fig. 4). The TIR lengths of *NuwaI* families range from 12 to 61 bp, and the first three nucleotides of TIRs are usually ‘GGG’ tri-nucleotide (Fig. 1). The average length of consensus sequences of autonomous candidates is ~4462 bp. These *NuwaI* transposons are distributed in 16 animal genomes. These species include 12 bony fish, 1 coleopteran, 1 crustacean, 1 molluscan, and 1 anthozoan (Fig. 2a). However, these species are distributed only in the kingdom of animals. Thus, the *NuwaI* transposons could be relatively younger elements in the eukaryotes. Finally, 3845 copies of *NuwaI* group were identified in the eukaryotic genomes. The genomic abundance and copy number of each *NuwaI* family in each species were shown in Fig. 2c, Additional file 1: Table S5, and Additional file 3: Table S6.

### Characterization and distribution of *NuwaII* transposons

**Fig. 5:**
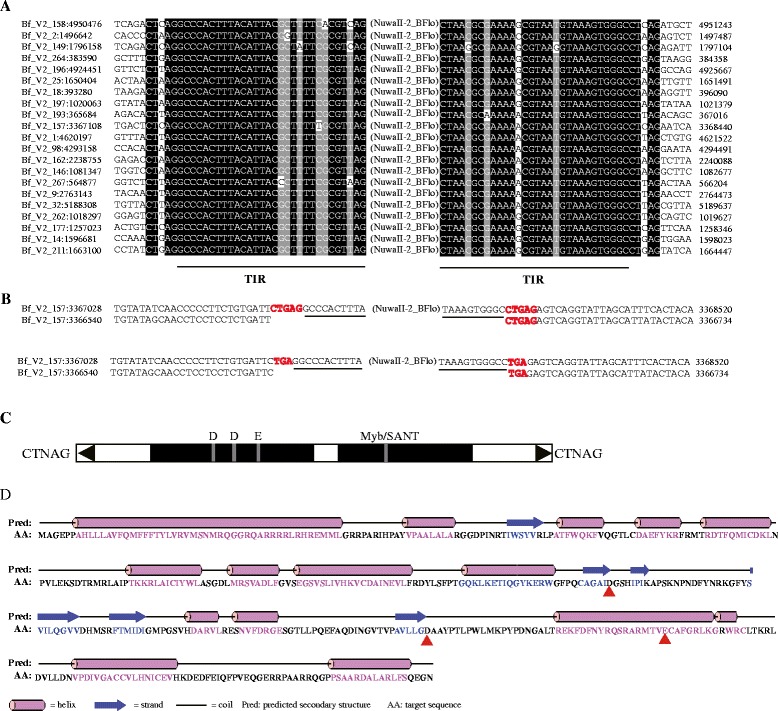
Characteristics of *NuwaII* transposons. **a** Sequence alignments for *NuwaII*-2*_BFlo* family. The terminal inverted repeats (TIRs) and flanking sequences (10 bp) are shown. **b** Examples of alignments of the flanking sequences of *NuwaII-2_BFlo* insertions with a paralogous sequences found within the same genome but devoid of the transposon. The TIRs of the element are *underlined*. **c** Structure of *NuwaII-2_BFlo. Black triangles* and *solid black boxes* represent the TIRs and ORFs, respectively, and the position of the DDE triad is shown. **d** Predicted secondary structure of the DDE motif-containing transposase of the *NuwaII-2_BFlo*. The DDE triads is marked with *red triangles* below the sequence

There are 30 out of 380 families which belong to the *NuwaII* families (Additional file 1: Table S7). According to the paralogous empty site, we cannot judge that the TSDs of NuwaII group are 3 bp (TNA) or 5 bp (CTNAG) (Fig. 5). However, most PHIS elements are typically associated with 3-bp TSD. Thus, the TSDs of NuwaII elements are most likely 3-bp TSDs. Meanwhile, there is a conserved single C nucleotide in the flank of 5′ terminal of TSDs and a conserved single G nucleotide in the flank of 3′ terminal of TSDs. Thus, the target of NuwaII is preferentially C|TNA|G (‘N’ represents A, T, C, or G nucleotide, ‘|’ represents the cut site).

The transposase of *NuwaII* is very similar to that of *NuwaI* in the coding capacity, conserved motifs, and second enzyme structure. For instance, the most autonomous elements of *NuwaII* transposons contain two ORFs, one ORF encoding the DDE motif-containing transposase (Additional file 2: Figure S3), and the other ORF encoding a Myb/SANT domain-containing protein. Twenty-two potential active *NuwaII* families with the two intact ORFs were identified in the eukaryotic genomes (Additional file 1: Table S7 and Fig. 2b). In the secondary structure of *NuwaII* transposase, the first D is located between two beta-sheets, the second D is typically between a beta-sheet and an alpha-helix, and the last E occurs within an alpha-helix (Fig. 5). The average length of consensus sequences of autonomous candidates is ~4685 bp; TIRs length of each family ranges from 13 to 46 bp, and the first two nucleotides of most TIRs are conserved GG. These *NuwaII* transposons are distributed in 12 species, including 1 turtle, 1 amphibian, 3 bony fishes, 1 amphioxus, 1 tunicate, 1 anthozoan, 2 basidiomycetes, 1 monocot, and 1 eudicot (Fig. 2a). Meanwhile, these species are also distributed in the kingdoms of plants, fungi, and animals. Thus, the *NuwaII* transposons could be also relatively old elements. Finally, we found 7564 copies of *NuwaII* group. The genomic abundance and copy number of each *NuwaII* family in each species are shown in Fig. 2c and Additional file 3: Table S8.

### Evolutionary relationships of PHIS transposons

**Fig. 6:**
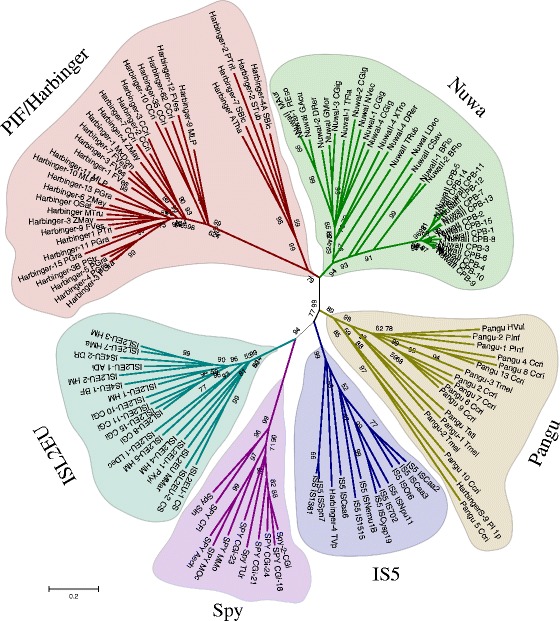
Phylogenetic tree based on DDE domain sequences of PHIS superfamily

To investigate the evolutionary relationships of six PHIS transposon groups (*PIF/Harbinger*, *ISL2EU*, *Spy*, *Pangu*, *NuwaI*, and *NuwaII*), the core catalytic DDE domain of 16 representative transposases (include intact DDE domain) of *Pangu*, 12 *NuwaI*, 21 *NuwaII*, 33 *PIF/Harbinger*, 18 *ISL2EU*, 11 *Spy*, and 11 bacterial *IS5* were used to perform a Bayesian phylogenetic analysis. The resulting tree (Fig. 6) showed that the eukaryotic PHIS transposases formed five distinct highly supported monophyletic clades beside the individual clade of bacterial *IS5* transposons. In the phylogenetic tree, *Pangu*, *PIF/Harbinger*, *ISL2EU*, and *SPY* transposons formed four separate clades. Meanwhile, *NuwaI* and *NuwaII* transposons formed a single clade in the phylogenetic tree.

## Discussion

### Identification and characterization of PHIS transposons

Previous study suggested that the PHIS is a DNA transposon superfamily with a great diversity in the eukaryotic genomes [15]. However, the detailed diversification and evolution of PHIS superfamily are still unknown. In this study, we systematically identified PHIS transposons in the eukaryotic genomes. A total of 380 families of PHIS superfamily were identified in 112 sequenced eukaryotic genomes. These families were classified into six groups based on the characteristic of each family’s TSDs. Among these groups, three (*PIF/Harbinger*, *ISL2EU*, and *Spy*) have been reported in the previous studies [15, 20, 21]. Beside the above three groups, we found three new transposon groups, called *Pangu*, *NuwaI*, and *NuwaII*.

These types shared similar transposases with DDE motif. However, each group has unique TSDs distinguished from others (Additional file 2: Figure S2). According to the criteria of previous TE classification [16], the transposases can be aligned over their entire catalytic regions (*e* value less than *e*^−4^), then they belong to the same superfamily. The same group of a superfamily was defined by the same TSD composition. In addition, previous studies showed that variable length or composition of TSDs have been identified in some superfamilies, such as 8–9 bp TSDs in *Merlin* superfamily, 5–8 bp in *hAT*, 2–4 bp in *CMC*, and 4–5 bp in *Ginger* [16, 22, 23]. Thus, it may be better to define *Spy*, *PIF/Harbinger*, and *ISL2EU* and *Pangu*, *NuwaI*, and *NuwaII* as different groups (at the same level) of the same superfamily (PHIS).

To estimate the abundance of each group in the eukaryotic genomes, the consensus sequence of each family of each group was used as a query in BlastN (*e* < 10^−5^) search against corresponding genome. Finally, we found that the abundances of these transposon groups varied in the eukaryotic genomes. For instance, there were 41,385 copies of *PIF/Harbinger* group, 3647 copies of *ISL2EU*, 13,089 copies of *SPY*, 3270 copies of *Pangu*, 3845 copies of *NuwaI*, and 7562 copies of *NuwaII* in the eukaryotic genomes (Additional files 1 and 3: Table S1–S8 and Fig. 2c). However, it should be noted that PHIS transposons were investigated using transposase homology search. Thus, some nonautonomous PHIS transposons (such as MITEs) might be missed in this study. In addition, we found that the number of potential active families varied. For example, there were 88 potential active families of *PIF/harbinger*, 12 families of *ISL2EU*, 18 families of *SPY*, 2 families of *Pangu*, 11 families of *NuwaI*, and 22 families of *NuwaII* in the eukaryotes (Fig. 2b). Furthermore, the abundance of each group was significantly positively correlated with the number of potential active families (Pearson’s product-moment correlation, *r* = 0.9816605, *P* = 0.0005). This phenomenon is easy to understand, and the more potential active families will have more copies for a group of PHIS transposon superfamily.

Most groups of PHIS superfamily include two ORFs, one coding for transposase containing DDE motif and the other ORF encoding a DNA-binding protein. However, *SPY* transposons include only one transposase containing DDE motif [15]. In addition, the additional ORFs of the four groups (including *Pangu*, *PIF/Harbinger*, *NuwaI*, and *NuwaII*) encode a protein with Myb/SANT domain except that of the *ISL2EU* transposon that encodes a protein with the Yqaj domain. At present, the functions of the additional ORFs are still unknown, and whether these ORFs are related to the transposition mechanisms also remains unclear [24]. This question could be answered using biochemical studies in the future.

The results of species distribution of PHIS transposons showed that the PHIS elements are completely absent in mammals, birds, sponges, sharks, and coelacanths. This is consistent with a previous study [16]. In addition, it is interesting to see that in some lineages, there is only one of the six groups of PHIS superfamily or only one of the six groups is absent. To our knowledge, the above results could be caused by two reasons. First, some PHIS transposons were lost or degenerated in some species by drift or selection in their original lineages. Second, some species gain different families from other species through horizontal transfer (HT). In addition, almost all of the DNA transposons have the ability of HT, and more and more HT of DNA transposons have been reported in the eukaryotic genomes [25–29]. Furthermore, previous studies suggested that PIF/Harbinger experienced HT events between *Drosophila* species [30]. However, HT of PHIS transposons remains to be studied in the future.

### Evolutionary relationships of PHIS transposons

The result of phylogenetic analysis showed that *Pangu* elements formed a single clade and were adjacent to *IS5* group in the phylogenetic tree. In addition, both *Pangu* and *IS5* transposons shared the same target site sequence (5′-ANT-3′). Furthermore, *Pangu* elements were widely distributed in plants, fungi, and animals. Thus, we proposed that *Pangu* is a relatively old PHIS group in the eukaryotic genomes.

Meanwhile, *NuwaI* and *NuwaII* transposons formed a single clade in the phylogenetic tree, and they shared the same coding capacity (encoding two ORFs) and the conserved domains (DDE motif and Myb/SANT domain). However, the TSDs of *NuwaI* are significantly different from the *NuwaII* transposons. *NuwaI* and *NuwaII* transposons should belong to two different groups of PHIS superfamily. Nevertheless, these two types might diverge recently. Thus, the two types cannot be distinguished from each other in the phylogenetic tree.

*HarbingerS-9_PI* and *Harbinger-4_TV* had been released as *PIF/Harbinger* families cataloged in RepBase. However, our phylogenetic analysis indicated that *HarbingerS-9_PI* was grouped into the clade of *Pangu* group. Meanwhile, *Harbinger-4_TV* was grouped into the *IS5* clade (Fig. 6). However, we could not find distinct target site duplications (TSDs) in the flank of *HarbingerS-9_PI* and *Harbinger-4_TV* families. Right now, we cannot judge if both families should belong to which group of PHIS superfamily.

## Conclusions

In the present study, 380 PHIS transposon families were identified in 112 of 836 sequenced eukaryotic genomes using transposase homology search and structure approach. Among these families, 168 families are firstly identified in this study. We systematically analyzed their characteristics including TSDs, TIRs, coding capacity, conserved transposase domain and species distribution, etc. The phylogenetic analysis based on the core catalytic DDE domain of these identified transposases showed that these PHIS transposon families were divided into five clusters including three previous reported clusters (*PIF/Harbinger*, *ISL2EU*, and *Spy*) and two new clusters (*Pangu* and *Nuwa*). *Nuwa* cluster includes two groups called *NuwaI* and *NuwaII*. Furthermore, each new group has its own distinctive characteristics, especially in target site sequences. For instance, the *Pangu* transposons are characterized by 5′-ANT-3′ TSDs, the *NuwaI* transposons by 5′-CWG-3′, and the *NuwaII* transposons by 5′-C|TNA|G-3′. Our results reveal the diversification and evolution of PHIS transposons in the eukaryotic genomes and imply that further study on the generation mechanism of varied target sequences of PHIS superfamily will promote the development of new transgenic vectors.

## Methods

### Identification of PHIS superfamily

Eukaryotic genomes including animals (295 species), plants (105 species), fungi (315 species), and protists (121 species) were downloaded from NCBI (http://www.ncbi.nlm.nih.gov/) (as of January 16, 2014), and the information of each species is listed in Additional file 3: Table S9. All published autonomous PHIS elements were downloaded from RepBase (v19.07) [31]. PHIS elements of eukaryotic genomes were identified using the transposase homology search that includes three steps (Additional file 2: Figure S4): (1) the transposase sequences of published PHIS elements were used as a query to do TblastN and TESeeker searches against each genome [32], where a hit with *e* value less than 10^−4^ was considered as candidate PHIS sequence; (2) each candidate PHIS nucleotide sequence was used as a query to BlastN search (*e* value < *e*^−5^, sequence length >50 bp, and nucleotide identity >80 %) against the corresponding genome; (3) the sequences of each cluster were extended in both directions using a Perl script and aligned using MUltiple Sequence Comparison by Log-Expectation (MUSCLE) [33], then the boundaries of each cluster were manually defined.

### Characterization and phylogenetic analysis of PHIS superfamily

To estimate the abundance of each PHIS family in the corresponding genome, the consensus sequence of each family was used as a query in BlastN search against the corresponding genome. Finally, the sequences with the *e* value less than *e*^−5^, length larger than 50 bp, and a minimum nucleotide identity of 80 % were classified as members of the same family. Transposase coding sequences, transposase domains, secondary structures of representative transposases, and the paralogous empty sites were analyzed as described previously [15]. Sequence logos of TIRs and TSDs were created by WebLogo (http://weblogo.berkeley.edu/logo.cgi) [34]. Multiple sequences alignments were performed using MUSCLE software with default parameters. The phylogenetic tree was constructed based on the DDE domains of transposases using MrBayes software (v3.1.2) [35] with the Blosum model and other parameters with default. The Blosum model was estimated by protest-3.2 software [36]. Meanwhile 3,000,000 generations of Bayesian inference were performed.

## Additional files

Additional file 1: Table S1. Distribution and characteristics of all identified *PIF/Harbinger* transposons. **Table S2.** Distribution and characteristics of all identified *ISL2EU* transposons. **Table S3.** Distribution and characteristics of all identified *Pangu* transposons. **Table S5.** Distribution and characteristics of all identified *NuwaI* transposons. **Table S7.** Distribution and characteristics of all identified *NuwaII* transposons. **Table S9.** The eukaryotes used in this study. Additional file 2: Figure S1. (A) Sequence alignments for *ISL2EU-2_Pxyl* family. The terminal inverted repeats (TIRs) and flanking sequences (10 bp) are shown. (B) An example of alignments of the flanking sequences of *ISL2EU-2_Pxyl* insertion with a paralogous sequences found within the same genome but devoid of the transposon. The TIRs of the element are underlined. **Figure S2.** Speculated transposition mechanism of each PHIS groups. **Figure S3.** The alignment of DDE domain of *Pangu* and *Nuwa* groups after redundancy elimination. Distances between the conserved blocks are indicated in the number of amino acid residues. Conserved residues within each superfamily are highlighted in the same color. The DDE triad identified here is marked with asterisks below alignments. **Figure S4.** Pipeline for PHIS transposons identification. Where a hit with *e* value less than 10^−4^ was considered as a homology sequence. The ones with an *e* value less than *e* ^−5^, sequence length larger than 50 bp, and nucleotide sequence identity larger than 80 % were classified as member of the same family. Target site duplications (TSDs) were identified using the paralogous empty sites. Additional file 3: Table S4. Positions of *Pangu* transposons in the corresponding genome. **Table S6.** Positions of *NuwaI* transposons in the corresponding genome. **Table S8.** Positions of *NuwaII* transposons in the corresponding genome.

## Abbreviations

MITEs: Miniature inverted-repeat transposable elements
TEs: Transposable elements
TIRs: Terminal inverted repeats
TSDs: Target site duplications
