# Supplementary material for: The diversification of PHIS transposon superfamily in eukaryotes
Source: Mob DNA. 2015 Jun 24;6:12. doi: 10.1186/s13100-015-0043-7 (PMC4482050; doi:10.1186/s13100-015-0043-7)
Supplement: Additional file 1: Table S1. — Distribution and characteristics of all identified PIF/Harbinger transposons. Table S2. Distribution and characteristics of all identified ISL2EU transposons. Table S3. Distribution and characteristics of all identified Pangu transposons. Table S5. Distribution and characteristics of all identified NuwaI transposons. Table S7. Distribution and characteristics of all identified NuwaII transposons. Table S9. The eukaryotes used in this study. [file 13100_2015_43_MOESM1_ESM.pdf]

<u>Table S1.</u> Distribution and characteristics of all identified *PIF*/*Harbinger* transposons

Species	Family name	Copies	Elements Length(bp)	TSD	TIR Length(bp)	Domain of ORF1	ORF1 length(aa)	Domain of ORF2	ORF2 length(aa)	Annotation
Acropora digitifera	*Harbinger-1_Adig*	154	769	TTA	37	-	-	-	-	New
	Harbinger-2_Adig	10	4664	TAA	16	DDE_Tnp_4	393	Myb_DNA-bind_4	188	New
	Harbinger-3_Adig	21	2883	TWA	44	DDE_Tnp_4	444	-	-	New
Acyrthosiphon pisum	*Harbinger-1_APis*	300	4359	TWA	41	-	-	SANT	253	New
	Harbinger-2_APis	6	3584	TWA	39	-	-	-	-	New
Aedes aegypti	*Harbinger-1_Aaae*	168	2897	TWA	17	-	-	SANT	240	New
	Harbinger-2_Aaae	495	3010	TWA	71	DDE_Tnp_4	349	-	-	New
	Harbinger-3_Aaae	454	3327	NNN	16	DDE_Tnp_4	350	SANT	244	New
Amphimedon queenslandica	*Harbinger-1_Aque*	102	2752	TWA	15	-	-	-	-	New
	Harbinger-2_Aque	260	3371	TWA	62	-	-	-	-	New
Anopheles gambiae	*Harbinger-1_Agam*	948	5377	TWA	69	DDE_Tnp_1_6,HTH_Tnp_4	471	Myb/SANT	245	HARBINGER1_AG
	Harbinger-2_Agam	4198	5036	TWA	26	DDE_Tnp_1_6	413	SANT	148	New
Anoplophora glabripennis	*Harbinger-1_Agla*	250	1246	TWA	16	-	-	-	-	New
	Harbinger-2_Agla	9	3040	TWA	45	DDE_Tnp_4	369	-	-	New
	Harbinger-3_Agla	15	3968	TWA	12	DDE_Tnp_4,HTH_Tnp_4	321	Myb_DNA-bind_5	345	New
	Harbinger-4_Agla	5	3091	TWA	14	-	-	-	-	New
Arabidopsis lyrata	*Harbinger-1_Alyr*	68	3720	TAA	11	Plant_tran	432	RPA_2b-aaRSs_OBF_like	373	HARB-1_ALy
	Harbinger-2_Alyr	235	5260	TWA	15	Plant_tran	435	SANT	311	HARB-2_ALy
	Harbinger-3_Alyr	233	5072	TWA	14	Plant_tran	350	SANT	261	HARB-3_ALy
	Harbinger-4_Alyr	235	5142	TWA	11	Plant_tran	428	NAM-associated	292	HARB-4_ALy
	Harbinger-5_Alyr	319	5139	TWA	15	Plant_tran	433	Myb_DNA-bind_4	316	HARB-6_ALy
	Harbinger-6_Alyr	327	4937	TWA	11	Plant_tran	429	SANT	294	HARB-7_ALy

	Harbinger-7_Alyr	318	5150	TWA	26	Plant_tran	429	SANT	320	HARB-8_ALy
	Harbinger-8_Alyr	40	3370	TWA	23	Plant_tran	573	-	-	HARB-9_ALy
	Harbinger-9_Alyr	66	1439	TWA	14	-	-	-	-	HARB-N1_ALy
	Harbinger-10_Alyr	144	1986	TWA	37	-	-	-	-	New
Arabidopsis thaliana	*Harbinger_Atha*	30	5736	TWA	15	DDE_Tnp_4	404	-	189	HARBINGER
Beta vulgaris	*Harbinger-1_Bvul*	153	4613	TWA	33	Plant_tran	457	RPA_2b-aaRSs_OBF_like	462	New
	Harbinger-2_Bvul	162	4353	TWA	17	Plant_tran	477	-	197	New
	Harbinger-3_Bvul	108	3934	TWA	16	Plant_tran	391	-	407	New
	Harbinger-4_Bvul	161	4073	TWA	11	Plant_tran	455	NAM-associated	344	New
	Harbinger-5_Bvul	169	3912	TWA	7	Plant_tran	341	NAM-associated	297	New
	Harbinger-6_Bvul	271	4565	TWA	11	Plant_tran	666	-	-	New
	Harbinger-7_Bvul	260	4631	TWA	31	-	255	-	-	New
Botryllus schlosseri	*Harbinger-1_BSch*	29	3965	TWA	22	DDE_Tnp_1_6	214	MADF_DNA_bdg	227	New
	Harbinger-2_BSch	264	4586	TWA	15	DDE_Tnp_4	351	SANT	128	New
	Harbinger-3_BSch	63	3657	TWA	16	DDE_Tnp_1_6	293	SANT	119	New
	Harbinger-4_BSch	306	4156	TWA	17	DDE_Tnp_1_6	262	-	196	New
	Harbinger-5_BSch	35	3293	TWA	18	DDE_Tnp_4,Myb_DNA-bind_5	559	-	-	New
	Harbinger-6_BSch	290	4129	TWA	40	DDE_Tnp_4	417	MADF	250	New
	Harbinger-7_BSch	274	4081	TWA	20	DDE_Tnp_4	354	SANT	252	New
Brachypodium distachyon	*Harbinger-1_BDis*	16	4185	TWA	18	DDE_Tnp_4	280	-	-	New
	Harbinger-2_BDis	82	4131	TWA	16	Plant_tran	439	NAM-associated	252	New
	Harbinger-3_BDis	59	4370	TWA	21	DDE_Tnp_4	412	PHA03269	305	New
	Harbinger-4_BDis	199	2070	TWA	21	DDE_Tnp_1_6	536	-	-	New

	Harbinger-5_BDis	40	3780	TWA	14	DDE_Tnp_4,Myb_DNA-bind_3	865	-	-	New
	Harbinger-6_BDis	73	2528	TWA	11	DDE_Tnp_1_6	326	-	-	New
	Harbinger-7_BDis	44	4363	TWA	14	DDE_Tnp_4,SANT	480	-	-	New
	Harbinger-8_BDis	94	4337	TWA	15	Plant_tran	434	-	-	New
	Harbinger-9_BDis	37	4430	TWA	6	Plant_tran	423	Myb_DNA-bind_4	382	New
Branchiostoma floridae	*Harbinger_Bflo*	275	4515	TWA	29	DDE_Tnp_4	360	MADF_DNA_bdg	457	Harbinger-4_BF
Brassica oleracea oleracea	*Harbinger_Bole*	586	5091	TWA	14	Plant_tran	422	Myb_DNA-bind_4	380	New
Brassica_rapa	*Harbinger-1_BRap*	264	2780	TWA	14	Plant_tran	520	-	-	New
	Harbinger-2_BRap	250	3649	TWA	12	Plant_tran	424	-	-	New
	Harbinger-3_BRap	262	4454	TWA	18	Plant_tran	447	NAM-associated	322	New
	Harbinger-4_BRap	302	924	TWA	12	-	-	-	-	New
	Harbinger-5_BRap	249	3073	TWA	12	Plant_tran	424	-	-	New
	Harbinger-6_BRap	470	2116	TWA	1042	DDE_Tnp_1_6	184	-	-	New
Caenorhabditis brenneri	*Harbinger_BBre*	14	3488	TWA	126	DDE_Tnp_1_6	442	-	-	New
Caenorhabditis elegans	*Harbinger_CEle*	6	5024	TWA	31	DDE_Tnp_4	354	-	-	TURMOIL1
Caenorhabditis japonica	*Harbinger-1_CJap*	558	3587	TWA	379	DDE_Tnp_1_6,SANT	696	-	-	New
	Harbinger-2_CJap	484	3420	TWA	406	DDE_Tnp_1_6	226	SANT	241	New
Capsella rubella	*Harbinger_CRub*	82	2041	TWA	21	DDE_Tnp_1_6	195	-	-	New
Ceratitis capitata	*Harbinger_CCap*	273	3083	TWA	15	DDE_Tnp_1_6	246	-	-	New
Chondrus crispus	*Harbinger-1_CCri*	7	4609	TWA	16	DDE_Tnp_1_6	534	PRK14971	396	New
	Harbinger-2_CCri	8	1071	TWA	16	-	-	-	-	Harbinger-N3_CCri
	Harbinger-3_CCri	28	3082	TWA	15	Plant_tran	423	-	-	Harbinger-4_CCri
	Harbinger-4_CCri	31	4159	TWA	15	DDE_Tnp_1_6	386	-	325	Harbinger-3_CCri
	Harbinger-5_CCri	14	4301	TWA	15	DDE_Tnp_1_6	434	-	418	Harbinger-35_CCri
	Harbinger-6_CCri	27	3756	TWA	15	DDE_Tnp_1_6	462	NAM-associated	493	Harbinger-6_CCri

	Harbinger-7_CCri	11	3331	TWA	35	DDE_Tnp_1_6	483	PRK12678	304	Harbinger-2_CCri
	Harbinger-8_CCri	11	3533	TWA	13	DDE_Tnp_1_6	243	-	278	Harbinger-37_CCri
	Harbinger-9_CCri	8	2611	TWA	16	DDE_Tnp_1_6	377	-	293	Harbinger-51_CCri
	Harbinger-10_CCri	4	3142	TWA	16	DDE_Tnp_1_6	444	-	350	Harbinger-60_CCri
	Harbinger-11_CCri	9	3095	TWA	16	DDE_Tnp_1_6	250	-	347	New
	Harbinger-12_CCri	200	7215	TWA	15	DDE_Tnp_4	911	-	430	Harbinger-5_CCri
	Harbinger-13_CCri	19	4865	TWA	15	DDE_Tnp_4	445	NAM-associated	451	Harbinger-7_CCri
	Harbinger-14_CCri	120	10107	TWA	38	DDE_Tnp_4	291	-	648	Harbinger-9_CCri
	Harbinger-15_CCri	236	563	TWA	38	-	-	-	-	Harbinger-9N1B_CCri
	Harbinger-16_CCri	94	5260	TWA	15	DDE_Tnp_4	488	NAM-associated	521	Harbinger-10_CCri
	Harbinger-17_CCri	51	6641	TWA	51	DDE_Tnp_4	480	-	547	Harbinger-46_CCri
	Harbinger-18_CCri	46	8561	TWA	51	DDE_Tnp_4	772	-	-	Harbinger-47_CCri
	Harbinger-19_CCri	54	5487	TWA	16	DDE_Tnp_4	702	-	367	Harbinger-62_CCri
	Harbinger-20_CCri	144	8673	TWA	49	DDE_Tnp_4	610	-	803	Harbinger-74_CCri
	Harbinger-21_CCri	10	3046	TWA	16	DDE_Tnp_4	477	-	346	Harbinger-75_CCri
Chrysemys picta bellii	*Harbinger-1_CPB*	281	4103	TWA	18	DDE_Tnp_4	338	Myb_DNA-bind_4	261	Harbinger-4_CPB
	Harbinger-2_CPB	276	3903	TWA	27	DDE_Tnp_4	415	Myb_DNA-bind_4	314	Harbinger-5B_CPB
Ciona savignyi	*Harbinger-1_CSav*	61	3700	TWA	23	DDE_Tnp_1_6,HTH_Tnp_4	269	BESS	115	New
	Harbinger-2_CSav	38	4148	TWA	18	DDE_Tnp_1_6,HTH_Tnp_4	414	-	-	New
	Harbinger-3_CSav	252	3877	TWA	25	DDE_Tnp_1_6,HTH_Tnp_4	321	-	157	New
	Harbinger-4_CSav	65	4382	TWA	16	-	-	-	-	New
	Harbinger-5_CSav	110	4508	TWA	16	DDE_Tnp_1_6,SANT	452	-	-	New
Citrus_clementina	*Harbinger-1_CCle*	214	5244	TWA	14	DDE_Tnp_1_6,Myb_DNA-bind_3	584	-	-	New

	Harbinger-2_CCle	310	3942	TWA	17	DDE_Tnp_4,Myb_DNA-bind_3,HTH_Tnp_4	660	-	-	New
Citrus_sinensis	*Harbinger-1_CSin*	290	3900	TWA	17	DDE_Tnp_4,HTH_Tnp_4	397	SANT	192	New
Crassostrea_gigas	*Harbinger_CGig*	284	4026	TWA	28	DDE_Tnp_4	433	SANT	379	Harbinger-6_CGi
Dianthus caryophyllus	*Harbinger-1_DCar*	5	3644	TWA	15	-	-	SANT	295	New
	Harbinger-2_DCar	164	3926	TWA	15	DDE_Tnp_1_6	199	NAM-associated	203	New
	Harbinger-3_DCar	97	3973	TWA	5	DDE_Tnp_1_6	228	-	-	New
Drosophila_bipectinata	*Harbinger_DBip*	132	1603	TWA	38	DDE_Tnp_4	433	-	-	New
Drosophila_rhopaloa	*Harbinger-1_DRho*	543	5073	TWA	20	DDE_Tnp_4	384	-	129	Harbinger-2B_DRh
	Harbinger-2_DRho	256	2644	TWA	18	DDE_Tnp_4	329	-	-	Harbinger-1_DRh
	Harbinger-3_DRho	261	754	TWA	18	-	-	-	-	Harbinger-1N1_DRh
Drosophila_takahashii	*Harbinger-1_DTak*	231	1197	TWA	19	-	-	-	-	New
	Harbinger-2_DTak	36	2836	TWA	40	-	-	SANT	183	New
	Harbinger-3_DTak	143	3075	TWA	25	DDE_Tnp_1_6	260	MADF	153	New
	Harbinger-4_DTak	66	2768	TWA	13	DDE_Tnp_4	353	SANT	164	New
Drosophila_willistoni	*Harbinger_DWil*	594	3689	TWA	36	DDE_Tnp_4	251	-	-	Harbinger-1_DW
Drosophila_yakuba	*Harbinger_DYak*	37	2324	TWA	35	DDE_Tnp_4	381	-	-	Harbinger1_DYa
Fragaria vesca	*Harbinger-1_FVSV*	170	2450	TWA	15	DDE_Tnp_1_6	311	-	-	HARB-1_FV
	Harbinger-3_FVSV	101	4126	TWA	13	Plant_tran	387	NAM-associated	294	HARB-3_FV
	Harbinger-4_FVSV	41	3762	TWA	21	Plant_tran	431	-	-	HARB-7_FV
	Harbinger-5_FVSV	32	3621	TWA	25	Plant_tran	433	NAM-associated	320	HARB-9_FV
	Harbinger-6_FVSV	59	3562	TWA	20	DDE_Tnp_1_6	423	NAM-associated	263	HARB-12_FV
Glycine max	*Harbinger-1_GMax*	218	4370	TWA	17	DDE_Tnp_4,SANT	760	-	-	New
	Harbinger-2_GMax	256	4351	TWA	16	Plant_tran	369	NAM-associated	231	HARB-1_GM
Gossypium raimondii	*Harbinger_GRai*	288	6055	TWA	13	DDE_Tnp_1_6,Myb_DNA-bind_3	416	-	-	New

Hydra vulgaris	*Harbinger-1_HVul*	106	3178	TWA	47	DDE_Tnp_4	321	-	-	New
	Harbinger-2_HVul	298	6190	TWA	14	DDE_Tnp_4	526	-	-	New
	Harbinger-3_HVul	138	3714	TWA	17	-	-	SANT	276	New
	Harbinger-4_HVul	391	3023	TWA	49	DDE_Tnp_1_6	240	SANT	116	New
	Harbinger-5_HVul	339	3126	TWA	41	-	-	-	-	New
	Harbinger-6_HVul	107	3119	TWA	51	DDE_Tnp_4	279	SANT	292	New
	Harbinger-7_HVul	48	2932	TWA	17	DDE_Tnp_1_6	204	-	319	New
Leptinotarsa_decemlineata	*Harbinger-1_LDec*	9	2983	TWA	10	DDE_Tnp_4	352	-	-	New
	Harbinger-2_LDec	25	2963	TWA	16	DDE_Tnp_1_6	210	SANT	184	New
	Harbinger-3_LDec	8	2968	TWA	49	-	-	MADF_DNA_bdg	299	New
	Harbinger-4_LDec	27	2964	TWA	36	DDE_Tnp_4	272	-	-	New
	Harbinger-5_LDec	59	2735	TWA	61	-	-	BESS,SANT	285	New
	Harbinger-6_LDec	271	3300	TWA	21	DDE_Tnp_4	370	MADF	182	New
Linepithema_humile	*Harbinger-1_LHum*	9	4250	TWA	13	DDE_Tnp_1_6	171	Myb_DNA-bind_4	314	New
	Harbinger-2_LHum	17	1154	TWA	37	-	-	-	-	New
	Harbinger-3_LHum	45	3498	TWA	42	-	-	-	-	New
Malus x domestica	*Harbinger_MXD*	290	2818	TWA	19	Plant_tran	467	-	-	HARB-1_Mad
Medicago truncatula	*Harbinger-1_MTru*	417	4117	TWA	16	Plant_tran	426	APG6	374	MTIS112A
	Harbinger-2_MTru	440	4701	TWA	17	-	-	Myb_DNA-bind_3	440	MtPH-A6-4-Ia
	Harbinger-3_MTru	171	4877	TWA	19	DDE_Tnp_4,Myb_DNA-bind_3	613	-	-	MtPH-A6-3-Ia
Melampsora larici populina	*Harbinger-1_MLP*	114	3357	TWA	13	DDE_Tnp_1_6	318	NAM-associated	322	Harbinger-4_MLP
	Harbinger-2_MLP	196	2871	TWA	13	DDE_Tnp_1_6	425	NAM-associated	290	Harbinger-10_MLP
	Harbinger-3_MLP	214	3072	TWA	13	DDE_Tnp_1_6	299	NAM-associated	237	Harbinger-11_MLP
Mesobuthus martensii	*Harbinger-1_MMar*	87	3256	TWA	25	DDE_Tnp_1_6	133	-	-	New
	Harbinger-2_MMar	142	3322	TWA	58	DDE_Tnp_1_6	255	-	-	New

	Harbinger-3_MMar	160	2902	TWA	57	DDE_Tnp_1_6	261	-	136	New
	Harbinger-4_MMar	275	3652	TWA	31	DDE_Tnp_4	231	SANT,BESS	199	New
	Harbinger-5_MMar	97	3709	TWA	24	-	-	Myb_DNA-bind_5	265	New
	Harbinger-6_MMar	144	3152	TWA	16	-	-	-	-	New
Mimulus guttatus	*Harbinger-1_MGut*	99	5234	TWA	26	Plant_tran	326	NAM-associated	241	New
	Harbinger-2_MGut	94	4599	TWA	26	-	-	-	-	New
Nasonia vitripennis	*Harbinger_NVit*	2	2503	TWA	56	-	-	-	-	New
Nematostella vectensis	*Harbinger_NVec*	17	3054	TWA	66	DDE_Tnp_1_6	153	SANT	180	New
Oryza_barthii	*Harbinger-1_OBar*	31	4981	TWA	13	DDE_Tnp_1_6	462	Myb_DNA-bind_4, NAM-associated	433	New
	Harbinger-2_OBar	379	2906	TWA	12	DDE_Tnp_1_6	384	-	-	New
Oryza brachyantha	*Harbinger-1_OBra*	278	3516	TWA	21	Plant_tran	540	-	-	New
	Harbinger-2_OBra	64	5032	TWA	21	DDE_Tnp_4,SANT	604	-	-	New
Oryza glaberrima	*Harbinger-1_OGla*	20	6405	TWA	12	DDE_Tnp_4,Myb_DNA-bind_3	650	-	-	New
	Harbinger-2_OGla	48	4478	TWA	15	Myb_DNA-bind_4	362	Plant_tran	450	New
	Harbinger-3_OGla	6	4376	TWA	21	DDE_Tnp_4	396	Myb_DNA-bind_3	321	New
	Harbinger-4_OGla	37	4063	TWA	25	DDE_Tnp_4	395	Myb_DNA-bind_3	370	New
Oryza glumipatula	*Harbinger_OGlu*	60	2658	TWA	15	Plant_tran	432	-	-	New
Oryza_sativa_Indica_Group	*Harbinger-1_OSIG*	6	2429	TWA	21	-	-	-	-	New
	Harbinger-2_OSIG	9	5166	TWA	14	Plant_tran	482	SANT,NAM-associated	340	HARB-1_OS
Oryza_sativa_Japonica_Group	*Harbinger-1_OSJG*	315	2758	TWA	15	DDE_Tnp_1_6	442	-	-	HARB-1_OS
	Harbinger-2_OSJG	32	4757	TWA	16	Plant_tran	545	Myb_DNA-bind_4, NAM-associated	409	New
	Harbinger-3_OSJG	12	2416	TWA	21	-	-	-	-	New

Phaseolus_vulgaris	*Harbinger_PVul*	264	5475	TWA	19	DDE_Tnp_1_6	133	-	-	New
Physcomitrella_patens	*Harbinger_PPat*	6	2554	TWA	15	Plant_tran	445	NAM-associated	197	New
Phytophthora infestans	*Harbinger_PInf*	9	2449	TWA	15	DDE_Tnp_4	375	SANT	257	PIF_Harbinger-2_PI
Plutella xylostella	*Harbinger-1_PXyl*	401	4269	TWA	20	DDE_Tnp_4	302	SANT	199	New
	Harbinger-2_PXyl	17	2941	TWA	13	DDE_Tnp_4	397	-	-	New
	Harbinger-3_PXyl	18	2698	TWA	37	-	-	MADF_DNA_bdg	323	New
	Harbinger-4_PXyl	4	3222	TWA	14	-	-	-	-	New
Pogonomyrmex barbatus	*Harbinger_PBar*	330	4102	TWA	27	DDE_Tnp_4	376	-	-	New
Populus_euphratica	*Harbinger_PEup*	225	4132	TWA	13	Plant_tran	428	NAM-associated	146	New
Populus trichocarpa	*Harbinger_PTri*	163	4335	TWA	16	Plant_tran	364	NAM-associated	161	Harbinger1_PTr
Priapulus caudatus	*Harbinger_PCau*	148	1953	TWA	45	DDE_Tnp_1_6	277	-	-	New
Prunus_mume	*Harbinger-1_PMum*	285	2540	TWA	16	Plant_tran	394	-	-	New
	Harbinger-2_PMum	281	4138	TWA	11	DDE_Tnp_1_6	298	NAM-associated	151	New
Prunus_persica	*Harbinger-1_PPer*	436	4231	TWA	5	Plant_tran	462	NAM-associated	180	New
	Harbinger-2_PPer	454	4237	TWA	11	Plant_tran	450	NAM-associated	294	Harbinger-1_PPe
	Harbinger-3_PPer	454	4143	TWA	11	DDE_Tnp_1_6	371	NAM-associated	181	New
	Harbinger-4_PPer	728	5374	TWA	14	DDE_Tnp_1_6	307	NAM-associated	145	New
Puccinia graminis	*Harbinger-1_PGra*	84	3526	TWA	15	Plant_tran	416	NAM-associated	365	Harbinger-3_PGr
	Harbinger-2_PGra	75	3376	TWA	15	Plant_tran	431	NAM-associated	439	Harbinger-4_PGr
	Harbinger-3_PGra	53	3362	TWA	17	Plant_tran	432	NAM-associated	414	Harbinger-5_PGr
	Harbinger-4_PGra	17	3292	TWA	15	Plant_tran	429	NAM-associated	230	Harbinger-11_PGr
	Harbinger-5_PGra	30	3171	TWA	12	Plant_tran	406	NAM-associated	309	Harbinger-13_PGr
	Harbinger-6_PGra	39	3628	TWA	26	DDE_Tnp_1_6	380	NAM-associated	404	Harbinger-15_PGr
	Harbinger-7_PGra	22	3152	TWA	25	Plant_tran	420	NAM-associated	312	Harbinger-16_PGr
	Harbinger-8_PGra	73	3490	TWA	15	Plant_tran	456	NAM-associated	416	Harbinger-19_PGr
	Harbinger-9_PGra	118	3687	TWA	34	Plant_tran	511	NAM-associated	411	Harbinger-22_PGr

Puccinia striiformis	*Harbinger_PStr*	97	3555	TWA	22	Plant_tran	432	NAM-associated	301	Harbinger-3B_PSt
Puccinia triticina	*Harbinger-1_PTri*	143	2923	TWA	16	DDE_Tnp_1_6	375	-	293	Harbinger-1_PTrit
	Harbinger-2_PTri	117	3436	TWA	14	DDE_Tnp_1_6	312	SANT	382	Harbinger-2_PTrit
Pyrus_x_bretschneideri	*Harbinger-1_PBre*	400	3092	TWA	17	Plant_tran	430	-	-	New
	Harbinger-2_PBre	279	4717	TWA	16	DDE_Tnp_1_6	374	SANT	301	New
	Harbinger-3_PBre	268	5140	TWA	17	Plant_tran	462	NAM-associated	322	New
Saccoglossus kowalevskii	*Harbinger_SKow*	256	2347	TWA	33	-	-	-	-	Harbinger-N1_SK
Setaria italica	*Harbinger-1_SIta*	241	5160	TWA	20	DDE_Tnp_4,Myb_DNA-bind_3	779	-	-	New
	Harbinger-2_SIta	104	5286	TWA	19	-	-	SANT	323	New
	Harbinger-3_SIta	220	8167	TWA	11	DDE_Tnp_4,Myb_DNA-bind_3	547	-	-	New
Solanum_lycopersicum	*Harbinger_SLyc*	302	1229	TWA	17	DDE_Tnp_1_6	139	-	-	New
Solanum_pennellii	*Harbinger_SPen*	258	1244	TWA	18	-	-	-	-	New
Solanum_tuberosum	*Harbinger-1_STub*	283	1753	TWA	15	Plant_tran	231	-	-	HARB-1_STu
	Harbinger-2_STub	265	7742	TWA	14	DDE_Tnp_4	297	SANT	455	HARB-2_STu
Sorghum_bicolor	*Harbinger_SBic*	295	1248	TWA	17	-	-	-	-	New
Strongylocentrotus_purpuratus	*Harbinger-1_SPur*	329	2160	TWA	48	DDE_Tnp_4	429	-	-	Harbinger-1_SP
	Harbinger-2_SPur	262	2966	TWA	22	DDE_Tnp_1_6	419	-	-	New
Vitis_vinifera	*Harbinger-1_VVin*	285	5286	TWA	11	DDE_Tnp_1_6,Myb_DNA-bind_3	602	-	-	VHARB4_VV
	Harbinger-2_VVin	245	734	TWA	20	-	-	-	-	Harbinger-1N1_VV
Xenopus_Silurana_tropicalis	*Harbinger_XST*	261	5790	TWA	15	DDE_Tnp_4	310	-	341	New
Xenopus_tropicalis	*Harbinger_XTro*	300	4687	TWA	34	-	-	MADF	331	Harbinger-3_XT
Zea mays	*Harbinger-1_ZMay*	936	5134	TWA	16	Plant_tran	455	NAM-associated	391	HARB3_ZM
	Harbinger-2_ZMay	323	5300	TWA	15	Plant_tran	515	NAM-associated	524	HARB4_ZM

	Harbinger-3_ZMay	312	2022	TWA	15	Plant_tran	421	-	-	HARB6_ZM

Table S2. Distribution and characteristics of all identified *ISL2EU* transposons

Species	Family name	Copies	Elements Length(bp)	TSD	TIR_Length(bp)	Domain of ORF1	ORF1 length(aa)	Domain of ORF2	ORF2 length(aa)	Annotation
Acropora digitifera	ISL2EU-ADig	113	4088	AT	33	DDE,THAP,HTH	525	YqaJ	646	ISL2EU-1_ADi
Bombyx mori	ISL2EU-BMor	110	2695	AT	9	-	-	-	-	ISL2EU-1_BM
Botryllus schlosseri	ISL2EU_BSch	87	2434	AT	12	DDE_Tnp_1_6	166	-	-	Unreleased
Branchiostoma floridae	ISL2EU_BFlo	243	5617	AT	12	DDE_Tnp_4,THAP,HTH_Tnp_4	426	YqaJ	512	IS4EU-1_BF
Caenorhabditis japonica	ISL2EU_CJap	269	3149	AT	259	DDE_Tnp_1_6	343	-	169	Unreleased
Capitella teleta	ISL2EU-CTel	89	1287	AT	9	-	-	-	-	Unreleased
Ciona savignyi	ISL2EU-1_CSav	361	4382	AT	12	DDE_Tnp_4,THAP,HTH_Tnp_4	454	YqaJ	500	ISL2EU-1_CS
	ISL2EU-2_CSav	44	4178	AT	6	DDE_Tnp_4,THAP,HTH_Tnp_4	448	YqaJ	200	ISL2EU-2_CS
	ISL2EU-3_CSav	22	4361	AT	148	DDE_Tnp_1_6	240	YqaJ	406	Unreleased
Crassostrea gigas	ISL2EU-1_CGig	12	5060	AT	14	DDE_Tnp_4	544	YqaJ	305	ISL2EU-8_CGi
	ISL2EU-2_CGig	17	4898	AT	14	DDE_Tnp_4,THAP	444	YqaJ	545	ISL2EU-10_CGi
	ISL2EU-3_CGig	19	4844	AT	18	DDE_Tnp_4,THAP,HTH_Tnp_4	534	YqaJ	477	ISL2EU-11_CGi
	ISL2EU-4_CGig	319	5351	AT	48	DDE_Tnp_4,THAP,HTH_Tnp_4	537	YqaJ	536	ISL2EU-15_CGi
Danio rerio	ISL2EU_DRer	141	5364	AT	14	DDE_Tnp_4,THAP,HTH_Tnp_4	475	YqaJ	515	IS4EU-2_DR
Hydra magnipapillata	ISL2EU-1_HMag	116	4359	AT	10	DDE_Tnp_4,THAP	528	-	-	ISL2EU-2_HM
	ISL2EU-2_HMag	180	7117	AT	12	DDE_Tnp_4,HTH_Tnp_4	353	YqaJ	488	ISL2EU-4_HM
	ISL2EU-3_HMag	92	3885	AT	81	DDE_Tnp_4,HTH_Tnp_4	524	YqaJ	631	ISL2EU-5_HM
	ISL2EU-4_HMag	158	3543	AT	68	DDE_Tnp_4,THAP,HTH_Tnp_4	552	-	354	ISL2EU-7_HM
	ISL2EU-5_HMag	100	3439	AT	70	DDE_Tnp_4,THAP,HTH_Tnp_4	523	-	-	ISL2EU_3_HM
	ISL2EU-6_HMag	256	2976	AT	126	-	-	YqaJ	647	ISL2EU_6_HM
Hydra vulgaris	ISL2EU_HVul	88	3443	AT	68	-	-	-	-	ISL2EU-3_HM
Leptinotarsa decemlineata	ISL2EU_LDec	136	4269	AT	13	DDE_Tnp_4,THAP,HTH_Tnp_4	576		391	Unreleased
Mesobuthus martensii	ISL2EU_MMar	24	4192	AT	59	DDE_Tnp_4,HTH_Tnp_4	330	-	-	Unreleased

Plutella xylostella	ISL2EU-1_PXyl	420	1483	AT	11	-	-	-	-	Unreleased
	ISL2EU-2_PXyl	256	746	AT	13	-	-	-	-	Unreleased

Table S3. **Distribution and characteristics of all identified *Pangu* transposons**

Species	**Family name**	**Copies**	**Elements Length(bp)**	**TSD**	**TIR Length(bp)**	**Domain of ORF1**	**ORF1 length(aa)**	**Domain of ORF2**	**ORF2 length(aa)**	**Annotation**
Acropora digitifera	*Pangu_ADig*	22	3222	ATT	40	DDE_Tnp_1_6	359	-	234	New
Aedes aegypti	*Pangu_AAeg*	1485	4548	AWT	16	DDE_Tnp_4	325	-	231	New
Anoplophora glabripennis	*Pangu_AGla*	5	2811	ANT	23	DDE_Tnp_4	216	Myb_DNA-bind_4	252	New
Chondrus crispus	*Pangu-1_CCri*	32	2754	ANT	15	DDE_Tnp_4	385	-	241	Harbinger-12_CCri
	Pangu-2_CCri	30	2516	ANT	18	-	111	-	266	DNA-6_CCri
	Pangu-3_Ccri	644	5516	ANT	28	DDE_Tnp_1_6	344	-	458	Harbinger-61_CCri
	Pangu-4_Ccri	14	3268	ANT	31	DDE_Tnp_1_6	418	-	413	Harbinger-63_CCri
	Pangu-5_Ccri	48	2631	ANT	13	DDE_Tnp_4	394	-	238	Harbinger-66_CCri
	Pangu-6_Ccri	18	2526	ANT	14	DDE_Tnp_1_6	398	-	286	Harbinger-33_CCri
	Pangu-7_Ccri	6	2722	ANT	14	DDE_Tnp_1_6	383	-	288	Harbinger-58_CCri
	Pangu-8_Ccri	10	2633	ANT	15	DDE_Tnp_4	367	-	254	Harbinger-16_CCri
	Pangu-9_Ccri	4	3862	AWT	15	DDE_Tnp_1_6	438	-	592	New
	Pangu-10_Ccri	14	3559	ANT	18	DDE_Tnp_1_6	392	-	351	Harbinger-25_CCri
	Pangu-11_Ccri	104	5150	ANT	17	DDE_Tnp_1_6	287	-	422	Harbinger-26_CCri
	Pangu-12_Ccri	9	2891	ANT	14	DDE_Tnp_4	382	-	424	Harbinger-36_CCri
	Pangu-13_Ccri	49	6364	ANT	22	DDE_Tnp_1_6	387	-	-	Harbinger-21_CCri
	Pangu-14_Ccri	10	3163	ANT	20	DDE_Tnp_1_6	394	-	451	Harbinger-23_CCri
	Pangu-15_Ccri	11	3153	ANT	16	DDE_Tnp_1_6	411	-	418	Harbinger-24_CCri
Crassostrea gigas	*Pangu_CGig*	71	3616	AWT	16	DDE_Tnp_4	402	Myb_DNA-bind_4	282	Harbinger-3_CGi
Ectocarpus siliculosus	*Pangu_ESil*	20	7867	AWT	15	DDE_Tnp_1_6	537	-	415	Harbinger2-1_ES
Hydra vulgaris	*Pangu_HVul*	94	2838	ANT	34	DDE_Tnp_1_6	307	-	230	Harbinger2-1_HM
Leptinotarsa decemlineata	*Pangu-1_LDec*	56	2619	ANT	22	DDE_Tnp_4	334	-	170	New
Melampsora larici populina	*Pangu-1_MLP*	35	5123	AWT	13	DDE_Tnp_4	487	-	555	Harbinger-1_MLP

	Pangu-2_MLP	35	4295	AWT	24	DDE_Tnp_4	438	-	451	Harbinger-3_MLP
Mesobuthus martensii	*Pangu-1_MMar*	48	1964	ATT	26	-	-	-	-	New
	Pangu-2_MMar	28	2915	AWT	15	-	-	-	-	New
Phytophthora infestans	*Pangu-1_PInf*	6	2729	ANT	22	DDE_Tnp_1_6	398	-	-	PIF_Harbinger-4_PI
	Pangu-2_PInf	32	2092	ANT	15	DDE_Tnp_1_6	303	-	207	PIF_Harbinger-5_PI
Puccinia striiformis	*Pangu_PStr*	237	199	ANT	18	-	-	-	-	Harbinger-N3_PSt
Talaromyces stipitatus	*Pangu_Tsti*	15	2567	AWT	14	DDE_Tnp_4	268	-	317	Harbinger2-1_TSt
Tremella mesenterica	*Pangu_Tmes*	11	3280	ANT	16	DDE_Tnp_1_6	322	-	398	PIF_Harbinger-1_TreMes
Tuber melanosporum	*Pangu-1_Tmel*	21	2731	AWT	27	DDE_Tnp_4	404	-	343	Harbinger2-1_TMe
	Pangu-2_Tmel	8	3156	ANT	14	DDE_Tnp_4	387	-	382	Harbinger2-2_TMe
	Pangu-3_Tmel	38	3060	ANT	11	DDE_Tnp_1_6	372	-	180	Harbinger2-3_TMe

Table S5. Distribution and characteristics of all identified *NuwaI* transposons

Species	Family name	Copies	Elements Length(bp)	TSD	TIRs length(bp)	Domain of ORF1	ORF1 length(aa)	Domain of ORF2	ORF2 length(aa)	Annotation
Crassostrea gigas	*NuwaI-1_CGig*	79	1581	CWG	19	DDE_Tnp_4	418	-	-	Harbinger-2_CGi
	NuwaI-2_CGig	249	5114	CWG	19	DDE_Tnp_4 and HTH	342	Myb_DNA-bind_5	256	Harbinger-4_CGi
	NuwaI-3_CGig	115	4889	CWG	18	DDE_Tnp_4	341	Myb_DNA-bind_5	228	Harbinger-5_CGi
	NuwaI-4_CGig	280	8497	CWG	24	DDE_Tnp_4	292	Myb_DNA-bind_5	171	Harbinger-8_CGi
Danio rerio	*NuwaI-1_DRer*	422	2531	CWG	18	-	-	-	-	Harbinger-N16B_DR
	NuwaI-2_DRer	293	4204	CWG	12	DDE_Tnp_4	343	Myb_DNA-bind_5	221	HARBINGER3_DR
	NuwaI-3_DRer	278	4080	CWG	23	DDE_Tnp_4	564	-	-	Harbinger-1_DR
	NuwaI-4_DRer	109	3727	CWG	13	DDE_Tnp_4	368	Myb_DNA-bind_5	276	HARBINGER2_DR
Daphnia pulex	*NuwaI-1_DPul*	26	3592	CWG	61	DDE_Tnp_1_6	298	Myb_DNA-bind_4	257	New
Gadus morhua	*NuwaI_GMor*	259	1894	CWG	17	DDE_Tnp_4	328	-	-	New
Gasterosteus aculeatus	*NuwaI_GAcu*	236	3798	CWG	23	DDE_Tnp_4	346	Myb_DNA-bind_5	198	Harbinger-1_GA
Leptinotarsa decemlineata	*NuwaI_LDec*	57	2807	CWG	15	DDE_Tnp_4	285	-	153	New
Melanochromis auratus	*NuwaI_MAur*	78	3004	CWG	25	DDE_Tnp_4	343	SANT	167	New
Nematostella vectensis	*NuwaI_NVec*	32	5945	CWG	24	DDE_Tnp_4	344	Myb_DNA-bind_5	280	Harbinger-1N1_NV
Oreochromis niloticus	*NuwaI_ONil*	100	1870	CWG	26	-	-	-	-	New
Pundamilia nyererei	*NuwaI-1_PNye*	32	1154	CWG	22	-	-	-	-	New
	NuwaI-2_PNye	234	1798	CWG	26	DDE_Tnp_1_6	224	-	-	New
Rhamphochromis esox	*NuwaI-1_REso*	83	2991	CWG	26	DDE_Tnp_4	343	-	-	New
Sebastes nigrocinctus	*NuwaI-1_SNig*	259	3810	CWG	15	-	-	-	-	New
Sebastes rubrivinctus	*NuwaI-1_SRub*	258	3399	CWG	15	DDE_Tnp_1_6	252	-	-	New
Takifugu flavidus	*NuwaI-1_TFla*	121	2779	CWG	14	DDE_Tnp_4	342	Myb_DNA-bind_5	160	New
Takifugu rubripes	*NuwaI-1_TRub*	35	3035	CWG	14	DDE_Tnp_1_6 and HTH	249	-	-	New
Thunnus orientalis	*NuwaI-1_TOri*	210	3534	CWG	22	DDE_Tnp_4	201	SANT	143	New

Table S7. Distribution and characteristics of all identified *NuwaII* transposons

Species	Family name	Copies	Elements Length(bp)	TSD	TIRs length(bp)	Domain of ORF1	ORF1 length(aa)	Domain of ORF2	ORF2 length(aa)	Annotation
Acropora digitifera	*NuwaII-1_ADig*	177	1149	C|TNA|G	28	-	-	-	-	New
Branchiostoma floridae	*NuwaII-1_BFlo*	192	5681	C|TNA|G	33	DDE_Tnp_4	426	Myb/SANT-like DNA-binding	370	Harbinger-2_BF
	NuwaII-2_BFlo	267	7602	C|TNA|G	30	DDE_Tnp_4 and HTH	423	Myb/SANT-like DNA-binding	394	Harbinger-3_BF
Chrysemys picta bellii	*NuwaII-1_CPB*	434	4453	C|TNA|G	15	DDE_Tnp_4	431	Myb_DNA-bind_4	307	Harbinger-2_CPB
	NuwaII-2_CPB	314	5034	C|TNA|G	18	DDE_Tnp_4	436	Myb_DNA-bind_4	351	Harbinger-2D_CPB
	NuwaII-3_CPB	289	4896	C|TNA|G	19	DDE_Tnp_4	427	Myb_DNA-bind_4	348	Harbinger-1_CPB
	NuwaII-4_CPB	286	4753	C|TNA|G	15	DDE_Tnp_4	427	Myb_DNA-bind_4	367	Harbinger-2E_CPB
	NuwaII-5_CPB	346	4028	C|TNA|G	17	DDE_Tnp_4	452	Myb_DNA-bind_4	364	Harbinger-4_CPB
	NuwaII-6_CPB	292	5081	C|TNA|G	17	DDE_Tnp_4	421	Myb_DNA-bind_4	231	Harbinger-2B_CPB
	NuwaII-7_CPB	289	4282	C|TNA|G	30	DDE_Tnp_4	418	Myb_DNA-bind_4	267	Harbinger-3B_CPB
	NuwaII-8_CPB	279	4443	C|TNA|G	15	DDE_Tnp_4	477	Myb_DNA-bind_4	358	Harbinger-2_CPB
	NuwaII-9_CPB	290	5013	C|TNA|G	18	DDE_Tnp_4	495	Myb_DNA-bind_4	368	Harbinger-2E_CPB
	NuwaII-10_CPB	286	4704	C|TNA|G	18	DDE_Tnp_4	427	Myb_DNA-bind_4	370	Harbinger-2C_CPB
	NuwaII-11_CPB	299	3943	C|TNA|G	19	DDE_Tnp_4	413	Myb_DNA-bind_4	362	Harbinger-5D_CPB
	NuwaII-12_CPB	365	4834	C|TNA|G	18	DDE_Tnp_4	411	Myb_DNA-bind_4	353	Harbinger-4B_CPB
	NuwaII-13_CPB	287	4319	C|TNA|G	23	DDE_Tnp_4	418	Myb_DNA-bind_4	333	Harbinger-3_CPB
	NuwaII-14_CPB	374	4039	C|TNA|G	17	DDE_Tnp_4	535	Myb_DNA-bind_4	316	Harbinger-4C_CPB
	NuwaII-15_CPB	265	5036	C|TNA|G	18	DDE_Tnp_4	433	Myb_DNA-bind_4	353	Harbinger-2D_CPB
Ciona savignyi	*NuwaII_CSav*	23	4109	C|TNA|G	18	DDE_Tnp_4	334	Myb_DNA-bind_4	154	New
Citrus clementina	*NuwaII_CCle*	309	3807	C|TNA|G	16	DDE_Tnp_4 and HTH	397	Myb_DNA-bind_3	279	New
Vitis vinifera	*NuwaII_VVin*	142	5919	C|TNA|G	22	DDE_Tnp_4	424	Myb_DNA-bind_3	271	Harbinger-3_VV
Maylandia zebra	*NuwaII-1_MZeb*	252	1848	C|CTG|G	26	DDE_Tnp_1_6	179	-	-	New
	NuwaII-2_MZeb	49	1114	C|CWG|G	17	-	-	-	-	New

Poecilia formosa	*NuwaII_PFor*	136	588	C|TNA|G	45	-	-	-	-	New
Puccinia graminis	*NuwaII_PGra*	99	3516	C|TNA|G	14	Plant_tran	453	NAM-associated	300	Harbinger-14_PGr
Puccinia striiformis	*NuwaII_PStr*	98	3595	C|TNA|G	14	Plant_tran	311	NAM-associated	395	Harbinger-3_PSt
Xenopus tropicalis	*NuwaII-1_XTro*	223	402	C|TNA|G	20	-	-	-	-	Harbinger-2N1A_XT
	NuwaII-2_XTro	134	427	C|TNA|G	23	-	-	-	-	Harbinger-2N1B_XT
	NuwaII-3_XTro	502	440	C|TNA|G	23	-	-	-	-	Harbinger-2N1C_XT
Takifugu rubripes	*NuwaII_TRub*	264	2224	C|NNN|G	17	DDE_Tnp_4	306	-	-	SENKUSHA1

Table S9. The eukaryotes used in this study

Organism/Name	Group	Size (Mb)	Level
Actinidia chinensis	Plants	604.217	Contig
Aegilops tauschii	Plants	3313.65	Scaffold
Aethionema arabicum	Plants	192.488	Scaffold
Amaranthus tuberculatus	Plants	4.34798	Contig
Amborella trichopoda	Plants	706.333	Scaffold
Arabidopsis lyrata subsp. lyrata	Plants	206.668	Scaffold
Arabidopsis thaliana	Plants	119.668	Chromosome with gaps
Azadirachta indica	Plants	261.122	Contig
Beta vulgaris subsp. vulgaris	Plants	566.567	Chromosome
Betula nana	Plants	564.011	Contig
Brachypodium distachyon	Plants	272.059	Chromosome
Brassica oleracea var. capitata	Plants	514.431	Scaffold
Brassica rapa	Plants	284.129	Chromosome
Cajanus cajan	Plants	510.809	Contig
Camelina sativa	Plants	641.356	Chromosome
Cannabis sativa	Plants	757.439	Scaffold
Capsella rubella	Plants	133.064	Scaffold
Capsicum annuum	Plants	3063.64	Scaffold
Carica papaya	Plants	369.782	Scaffold
Chlamydomonas reinhardtii	Plants	120.405	Scaffold
Chlorella variabilis	Plants	46.1595	Scaffold
Chondrus crispus	Other	104.98	Scaffold
Cicer arietinum	Plants	530.894	Chromosome
Citrullus lanatus	Plants	321.047	Contig
Citrus clementina	Plants	301.365	Scaffold
Citrus sinensis	Plants	327.83	Chromosome
Cleome hassleriana	Plants	249.93	Scaffold
Coccomyxa subellipsoidea C-169	Plants	48.8266	Contig
Cucumis melo	Plants	374.928	Scaffold
Cucumis sativus	Plants	323.986	Scaffold
Cyanidioschyzon merolae strain 10D	Other	16.5467	Complete Genome
Dianthus caryophyllus	Plants	567.662	Scaffold
Elaeis guineensis	Plants	1535.02	Chromosome
Ensete ventricosum	Plants	172.242	Scaffold
Eucalyptus camaldulensis	Plants	654.922	Contig
Eutrema parvulum	Plants	137.073	Chromosome
Eutrema salsugineum	Plants	243.11	Scaffold
Fragaria vesca subsp. vesca	Plants	214.373	Chromosome
Fragaria x ananassa	Plants	697.762	Scaffold
Galdieria sulphuraria	Other	13.712	Scaffold
Glycine max	Plants	973.779	Chromosome

Gossypium raimondii	Plants	761.405	Chromosome
Hevea brasiliensis	Plants	1301.4	Scaffold
Hordeum vulgare subsp. vulgare	Plants	1868.64	Contig
Jatropha curcas	Plants	318.363	Scaffold
Lactuca sativa	Plants	1133.66	Contig
Lagenaria siceraria	Plants	176.727	Scaffold
Leavenworthia alabamica	Plants	173.432	Scaffold
Leersia perrieri	Plants	266.688	Chromosome
Linum usitatissimum	Plants	282.202	Contig
Lotus japonicus	Plants	147.812	Contig
Lupinus angustifolius	Plants	523.298	Scaffold
Malus x domestica	Plants	1874.77	Chromosome
Medicago truncatula	Plants	314.478	Chromosome
Micromonas pusilla CCMP1545	Plants	22.0001	Scaffold
Micromonas sp. RCC299	Plants	21.1093	Complete Genome
Morus notabilis	Plants	320.379	Scaffold
Musa acuminata subsp. malaccensis	Plants	472.236	Chromosome
Nelumbo nucifera	Plants	804.648	Scaffold
Nicotiana sylvestris	Plants	2221.99	Scaffold
Nicotiana tomentosiformis	Plants	1688.47	Scaffold
Oryza barthii	Plants	308.272	Chromosome
Oryza brachyantha	Plants	259.908	Chromosome
Oryza glaberrima	Plants	17.4575	Scaffold
Oryza glumipatula	Plants	372.86	Chromosome
Oryza granulata	Plants	35.2457	Scaffold
Oryza meridionalis	Plants	335.668	Chromosome
Oryza sativa Indica Group	Plants	426.337	Chromosome
Ostreococcus lucimarinus CCE9901	Plants	13.2049	Complete Genome
Ostreococcus tauri	Plants	12.5723	Chromosome with gaps
Penstemon cyananthus	Plants	4.62226	Contig
Penstemon davidsonii	Plants	2.37523	Contig
Penstemon dissectus	Plants	2.62809	Contig
Penstemon fruticosus	Plants	2.31904	Contig
Phaseolus vulgaris	Plants	521.077	Chromosome
Phoenix dactylifera	Plants	556.481	Scaffold
Physcomitrella patens	Plants	477.948	Scaffold
Picea glauca	Plants	25471.9	Scaffold
Pinus taeda	Plants	265.48	Contig
Populus euphratica	Plants	495.876	Scaffold
Populus trichocarpa	Plants	417.287	Chromosome
Porphyridium purpureum	Other	19.4519	Contig
Prunus mume	Plants	234.03	Chromosome
Prunus persica	Plants	227.252	Scaffold

Pyrus x bretschneideri	Plants	508.551	Scaffold
Ricinus communis	Plants	350.622	Scaffold
Selaginella moellendorffii	Plants	212.502	Scaffold
Sesamum indicum	Plants	274.906	Chromosome
Setaria italica	Plants	405.737	Scaffold
Sisymbrium irio	Plants	245.55	Scaffold
Solanum lycopersicum	Plants	781.509	Chromosome
Solanum pimpinellifolium	Plants	688.247	Contig
Solanum tuberosum	Plants	705.934	Scaffold
Sorghum bicolor	Plants	739.15	Chromosome
Spinacia oleracea	Plants	474.077	Scaffold
Spirodela polyrhiza	Plants	132.009	Contig
Theobroma cacao	Plants	345.994	Chromosome
Triticum aestivum	Plants	3800.33	Contig
Triticum urartu	Plants	3747.05	Scaffold
Vigna angularis var. angularis	Plants	291.824	Scaffold
Vigna radiata var. radiata	Plants	463.085	Contig
Vitis vinifera	Plants	486.197	Chromosome
Volvox carteri f. nagariensis	Plants	137.684	Scaffold
Zea mays	Plants	2067.62	Chromosome
Zizania latifolia	Plants	603.989	Scaffold
Aciculosporium take MAFF-241224	Fungi	58.8364	Contig
Agaricus bisporus var. burnettii JB137-S8	Fungi	32.6144	Scaffold
Ajellomyces capsulatus H88	Fungi	37.9432	Scaffold
Ajellomyces dermatitidis ATCC 18187	Fungi	61.1344	Scaffold
Allomyces macrogynus ATCC 38327	Fungi	57.0606	Scaffold
Alternaria arborescens EGS 39-128	Fungi	33.8894	Contig
Alternaria brassicicola ATCC 96836	Fungi	29.5365	Contig
Amanita jacksonii TRTC168611	Fungi	30.8564	Scaffold
Anncaliia algerae	Fungi	13.8234	Contig
Arthrobotrys oligospora ATCC 24927	Fungi	40.0728	Scaffold
Arthroderma benhamiae CBS 112371	Fungi	22.2242	Scaffold
Microsporum gypseum CBS 118893	Fungi	23.2682	Scaffold
Arthroderma otae CBS 113480	Fungi	23.2631	Scaffold
Ascocoryne sarcoides NRRL 50072	Fungi	34.2474	Scaffold
Ascosphaera apis ARSEF 7405	Fungi	21.5221	Scaffold
Aspergillus clavatus NRRL 1	Fungi	27.8594	Scaffold
Aspergillus flavus NRRL3357	Fungi	36.8923	Scaffold
Aspergillus fumigatus A1163	Fungi	29.2054	Scaffold
Aspergillus kawachii IFO 4308	Fungi	37.1132	Scaffold
Aspergillus nidulans FGSC A4	Fungi	30.2427	Scaffold
Aspergillus niger ATCC 1015	Fungi	34.8533	Contig

Aspergillus oryzae 100-8	Fungi	36.7693	Contig
Aspergillus sojae NBRC 4239	Fungi	39.771	Scaffold
Aspergillus terreus NIH2624	Fungi	29.364	Scaffold
Aureobasidium pullulans AY4	Fungi	26.7227	Contig
Auricularia delicata TFB-10046 SS5	Fungi	74.9202	Scaffold
Batrachochytrium dendrobatidis JAM81	Fungi	24.3151	Scaffold
Baudoinia compniacensis UAMH 10762	Fungi	21.8756	Scaffold
Beauveria bassiana ARSEF 2860	Fungi	33.6978	Scaffold
Bipolaris maydis ATCC 48331	Fungi	32.9292	Scaffold
Cochliobolus sativus ND90Pr	Fungi	34.4092	Scaffold
Blumeria graminis f. sp. hordei DH14	Fungi	118.726	Scaffold
Botryotinia fuckeliana B05.10	Fungi	42.7438	Scaffold
Byssochlamys spectabilis No. 5	Fungi	29.7624	Contig
Candida albicans 12C	Fungi	14.897	Contig
Candida dubliniensis	Fungi	14.0449	Contig
Candida glabrata CCTCC M202019	Fungi	12.1169	Scaffold
Candida maltosa Xu316	Fungi	12.8263	Contig
Candida orthopsilosis AY2	Fungi	14.5117	Contig
Candida parapsilosis CDC317	Fungi	13.0785	Contig
Candida tenuis ATCC 10573	Fungi	10.747	Scaffold
Candida tropicalis MYA-3404	Fungi	14.6301	Scaffold
Cerataphis brasiliensis yeast-like symbiont	Fungi	25.4655	Scaffold
Ceratocystis fimbriata CBS 114723	Fungi	23.5898	Contig
Cercospora canescens BHU	Fungi	33.9672	Contig
Ceriporiopsis subvermispora B	Fungi	38.9697	Scaffold
Chaetomium globosum CBS 148.51	Fungi	34.8869	Scaffold
Chaetomium thermophilum var. thermophilum DSM 1495	Fungi	28.3228	Scaffold
Cladonia macilenta KoLRI003786	Fungi	37.1171	Scaffold
Cladonia metacorallifera KoLRI002260	Fungi	36.6821	Scaffold
Cladophialophora carrionii CBS 160.54	Fungi	28.9911	Scaffold
Cladosporium sphaerospermum UM 843	Fungi	26.8942	Scaffold
Claviceps fusiformis PRL 1980	Fungi	52.5856	Contig
Claviceps paspali RRC 1481	Fungi	28.9743	Contig
Claviceps purpurea 20.1	Fungi	32.0914	Contig
Clavispora lusitaniae ATCC 42720	Fungi	12.1149	Scaffold
Coccidioides immitis H538.4	Fungi	27.7811	Scaffold
Coccidioides posadasii C735 delta SOWgp	Fungi	27.0134	Scaffold
Colletotrichum gloeosporioides Cg-14	Fungi	53.2099	Contig
Glomerella graminicola M1.001	Fungi	51.6443	Scaffold
Colletotrichum higginsianum	Fungi	49.0849	Contig

Colletotrichum orbiculare MAFF 240422	Fungi	90.0861	Scaffold
Coniophora puteana RWD-64-598 SS2	Fungi	42.9685	Scaffold
Coniosporium apollinis CBS 352.97	Fungi	28.6453	Scaffold
Coprinopsis cinerea okayama7#130	Fungi	36.1926	Contig
Cordyceps militaris CM01	Fungi	32.2686	Scaffold
Cronartium comandrae C4	Fungi	68.6058	Scaffold
Cronartium quercuum f. sp. banksianae CqE3WM	Fungi	22.134	Scaffold
Cronartium ribicola 11-2	Fungi	94.3329	Scaffold
Cryomyces antarcticus CCFEE 534	Fungi	24.3237	Contig
Cryptococcus bestiolae CBS 10118	Fungi	24.3608	Scaffold
Cryptococcus dejecticola CBS 10117	Fungi	23.8624	Scaffold
Cryptococcus flavescens NRRL Y-50378	Fungi	22.7905	Contig
Cryptococcus gattii	Fungi	17.2566	Scaffold
Cryptococcus neoformans var. grubii H99	Fungi	18.9161	Chromosome with gaps
Cryptococcus pinus CBS 10737	Fungi	20.8286	Scaffold
Cyberlindnera jadinii NBRC 0988	Fungi	14.2753	Chromosome
Phialophora europaea CBS 101466	Fungi	28.7813	Scaffold
Dacryopinax sp. DJM-731 SS1	Fungi	29.5035	Scaffold
Dactylellina haptotyla CBS 200.50	Fungi	39.5319	Scaffold
Daldinia eschscholtzii	Fungi	35.5399	Contig
Debaryomyces hansenii CBS767	Fungi	12.1819	Chromosome with gaps
Dekkera bruxellensis CBS 2499	Fungi	13.3559	Scaffold
Dichomitus squalens LYAD-421 SS1	Fungi	42.7484	Scaffold
Edhazardia aedis USNM 41457	Fungi	50.7488	Contig
Encephalitozoon cuniculi EC1	Fungi	2.28811	Contig
Encephalitozoon hellem ATCC 50504	Fungi	2.25178	Chromosome
Encephalitozoon intestinalis ATCC 50506	Fungi	2.2169	Chromosome with gaps
Encephalitozoon romaleae SJ-2008	Fungi	2.18759	Chromosome
Endocarpon pusillum	Fungi	37.1732	Scaffold
Endocronartium harknessii PhW48OC	Fungi	56.9403	Scaffold
Enterocytozoon bieneusi H348	Fungi	3.86074	Scaffold
Epichloe amarillans E57	Fungi	38.0624	Contig
Epichloe brachyelytri E4804	Fungi	44.2319	Contig
Epichloe elymi E56	Fungi	32.3351	Contig
Epichloe festucae E2368	Fungi	35.0416	Scaffold
Epichloe glyceriae E277	Fungi	46.723	Contig
Epichloe typhina E5819	Fungi	34.1858	Contig
Eremothecium cymbalariae DBVPG#7215	Fungi	9.66942	Chromosome with gaps

Ashbya gossypii ATCC 10895	Fungi	9.11931	Complete Genome
Erysiphe pisi	Fungi	69.261	Contig
Eutypa lata UCREL1	Fungi	54.0058	Scaffold
Exophiala dermatitidis NIH/UT8656	Fungi	26.3768	Scaffold
Fibroporia radiculosa	Fungi	28.378	Scaffold
Flammulina velutipes KACC42780	Fungi	35.6425	Chromosome
Fomitiporia mediterranea MF3/22	Fungi	63.3544	Scaffold
Fomitopsis pinicola FP-58527 SS1	Fungi	41.6144	Scaffold
fungal sp. EF0021	Fungi	44.3721	Contig
Fusarium circinata FSP 34	Fungi	44.138	Contig
Fusarium fujikuroi B14	Fungi	43.8127	Scaffold
Fusarium graminearum	Fungi	36.6676	Chromosome
Fusarium oxysporum	Fungi	50.5502	Contig
Fusarium pseudograminearum CS3096	Fungi	36.9329	Contig
Fusarium verticillioides 7600	Fungi	41.8851	Chromosome
Gaeumannomyces graminis var. tritici R3-111a-1	Fungi	43.7687	Scaffold
Ganoderma lucidum BCRC 37177	Fungi	44.0804	Contig
Glarea lozoyensis 74030	Fungi	38.7183	Scaffold
Gloeophyllum trabeum ATCC 11539	Fungi	37.1782	Scaffold
Grosmannia clavigera kw1407	Fungi	29.794	Scaffold
Phyllosticta citricarpa CGMCC3.14348	Fungi	32.0072	Contig
Gyalolechia flavorubescens KoLRI002931	Fungi	34.4682	Scaffold
Hamiltosporidium tvaerminnensis OER-3-3	Fungi	13.2708	Contig
Helminthosporium solani B-AC-16A	Fungi	34.5316	Contig
Herpotrichiellaceae sp. UM238	Fungi	28.3643	Contig
Heterobasidion irregulare TC 32-1	Fungi	33.65	Scaffold
Hirsutella thompsonii MTCC3556	Fungi	34.5574	Contig
Rhizophydiales sp. JEL142	Fungi	26.1117	Scaffold
Hortaea werneckii EXF-2000	Fungi	51.6745	Scaffold
Hysterium pulicare CBS 123377	Fungi	38.2497	Contig
Kazachstania africana CBS 2517	Fungi	11.1301	Chromosome with gaps
Kazachstania naganishii CBS 8797	Fungi	10.8458	Chromosome with gaps
Kluyveromyces aestuarii ATCC 18862	Fungi	9.91011	Contig
Kluyveromyces lactis	Fungi	10.7294	Complete Genome
Kluyveromyces marxianus	Fungi	11.1654	Contig
Kluyveromyces wickerhamii UCD 54-210	Fungi	9.80774	Contig
Komagataella pastoris CBS 7435	Fungi	9.40272	Chromosome with gaps
Kwoniella heveanensis BCC8398	Fungi	25.4694	Scaffold
Kwoniella mangroviensis CBS 8886	Fungi	22.8727	Scaffold

Laccaria bicolor S238N-H82	Fungi	64.8774	Scaffold
Lachancea kluyveri NRRL Y-12651	Fungi	11.5091	Chromosome
Lachancea thermotolerans CBS 6340	Fungi	10.3929	Chromosome with gaps
Lachancea waltii NCYC 2644	Fungi	10.9121	Contig
Lecanosticta acicola CBS 871.95	Fungi	34.8734	Scaffold
Leptosphaeria maculans JN3	Fungi	45.1246	Scaffold
Leucoagaricus gongylophorus Ac12	Fungi	100.245	Contig
Lodderomyces elongisporus NRRL YB-4239	Fungi	15.5473	Scaffold
Macrophomina phaseolina MS6	Fungi	48.8828	Contig
Magnaporthe oryzae	Fungi	42.3067	Scaffold
Magnaporthe poae ATCC 64411	Fungi	39.6418	Scaffold
Malassezia globosa CBS 7966	Fungi	8.95812	Contig
Malassezia restricta CBS 7877	Fungi	4.62844	Contig
Malassezia sympodialis ATCC 42132	Fungi	7.66969	Scaffold
Marssonina brunnea f. sp. 'multigermtubi' MB_m1	Fungi	51.9499	Scaffold
Melampsora larici-populina 98AG31	Fungi	101.129	Scaffold
Metarhizium acridum CQMa 102	Fungi	39.4223	Scaffold
Metarhizium anisopliae	Fungi	38.4771	Contig
Metschnikowia fructicola 277	Fungi	24.4822	Contig
Meyerozyma guilliermondii ATCC 6260	Fungi	10.61	Scaffold
Microbotryum violaceum p1A1 Lamole	Fungi	26.1389	Scaffold
Millerozyma farinosa	Fungi	21.4987	Complete
Mixia osmundae IAM 14324	Fungi	13.6345	Scaffold
Moniliophthora perniciosa FA553	Fungi	26.6633	Contig
Moniliophthora roreri MCA 2997	Fungi	52.2049	Contig
Mortierella alpina ATCC 32222	Fungi	38.0421	Contig
Mucor circinelloides B8987	Fungi	36.7006	Contig
Myceliophthora thermophila ATCC 42464	Fungi	38.7442	Complete Genome
Mycosphaerella laricina CBS 326.52	Fungi	26.5981	Scaffold
Dothistroma septosporum NZE10	Fungi	30.2094	Scaffold
Mycosphaerella sp. Ston1	Fungi	27.5099	Scaffold
Naumovozyma castellii CBS 4309	Fungi	11.2195	Chromosome with gaps
Naumovozyma dairenensis CBS 421	Fungi	13.5276	Chromosome with gaps
Nectria haematococca mpVI 77-13-4	Fungi	51.2865	Scaffold
Nematocida parisii ERTm1	Fungi	4.07135	Scaffold
Nematocida sp. 1 ERTm2	Fungi	4.70071	Scaffold
Neofusicoccum parvum UCRNP2	Fungi	42.5928	Scaffold
Neosartorya fischeri NRRL 181	Fungi	31.77	Scaffold
Neotyphodium gansuense E7080	Fungi	39.6162	Contig

Neotyphodium gansuense var. inebrians E818	Fungi	29.6758	Contig
Neurospora crassa	Fungi	40.4162	Scaffold
Neurospora tetrasperma FGSC 2508	Fungi	39.1463	Scaffold
Nosema apis BRL 01	Fungi	8.5695	Scaffold
Nosema bombycis CQ1	Fungi	15.6898	Scaffold
Nosema ceranae BRL01	Fungi	7.86022	Contig
Ogataea parapolymorpha DL-1	Fungi	8.87459	Chromosome
Omphalotus olearius VT 653.13	Fungi	27.9937	Scaffold
Ophiocordyceps sinensis CO18	Fungi	78.5158	Scaffold
Ophiognomonia clavigignenti-juglandacearum	Fungi	15.9064	Contig
Ophiostoma novo-ulmi subsp. novo-ulmi H327	Fungi	31.8554	Scaffold
Orpinomyces sp. C1A	Fungi	100.954	Contig
Pachysolen tannophilus NRRL Y-2460	Fungi	12.2379	Contig
Paracoccidioides brasiliensis Pb03	Fungi	29.0627	Scaffold
Paracoccidioides sp. 'lutzii' Pb01	Fungi	32.9742	Scaffold
Phaeosphaeria nodorum SN15	Fungi	37.214	Scaffold
Passalora fulva CBS 131901	Fungi	61.1039	Scaffold
Penicillium camemberti FM 013	Fungi	35.012	Scaffold
Penicillium chrysogenum	Fungi	32.5255	Chromosome
Penicillium digitatum Pd01-ZJU	Fungi	25.0111	Contig
Penicillium decumbens 114-2	Fungi	30.1774	Scaffold
Penicillium paxilli ATCC 26601	Fungi	34.8025	Scaffold
Penicillium roqueforti	Fungi	27.9334	Contig
Periglandula ipomoeae IasaF13	Fungi	35.3016	Contig
Pestalotiopsis fici W106-1	Fungi	51.9107	Scaffold
Phanerochaete carnosa HHB-10118-sp	Fungi	46.2933	Scaffold
Phanerochaete chrysosporium RP-78	Fungi	29.8426	Contig
Phellinus noxius OVT-YTM/97	Fungi	31.2593	Contig
Pichia kudriavzevii	Fungi	12.9406	Contig
Piriformospora indica DSM 11827	Fungi	25.0437	Contig
Pleosporales sp. UM 1110	Fungi	36.9196	Contig
Pneumocystis jirovecii	Fungi	8.17998	Contig
Pneumocystis murina B123	Fungi	7.45136	Contig
Podospora anserina S mat+	Fungi	34.7189	Scaffold
Postia placenta Mad-698-R	Fungi	90.8919	Scaffold
Pseudocercospora fijiensis CIRAD86	Fungi	74.1412	Scaffold
Pseudocercospora pini-densiflorae CBS 125139	Fungi	45.1712	Scaffold
Pseudogymnoascus destructans M1379	Fungi	30.2827	Contig

Pseudogymnoascus pannorum var. pannorum M1372	Fungi	29.4559	Contig
Pseudozyma antarctica	Fungi	18.112	Scaffold
Pseudozyma aphidis DSM 70725	Fungi	17.9217	Contig
Pseudozyma flocculosa PF-1	Fungi	23.3054	Scaffold
Pseudozyma hubeiensis SY62	Fungi	18.4429	Scaffold
Pseudozyma sp. GHG001	Fungi	17.3254	Scaffold
Puccinia graminis f. sp. tritici CRL 75-36-700-3	Fungi	88.7244	Scaffold
Puccinia psidii MF-1	Fungi	0.362051	Contig
Puccinia striiformis f. sp. tritici 08/21	Fungi	56.2671	Contig
Puccinia triticina 1-1 BBBD Race 1	Fungi	162.949	Scaffold
Punctularia strigosozonata HHB-11173 SS5	Fungi	34.1719	Scaffold
Pyrenochaeta sp. UM 256	Fungi	35.4843	Scaffold
Pyrenophora seminiperda CCB06	Fungi	32.5392	Scaffold
Pyrenophora teres f. teres 0-1	Fungi	33.5833	Scaffold
Pyrenophora tritici-repentis Pt-1C-BFP	Fungi	37.9975	Scaffold
Rhizoctonia solani 123E	Fungi	39.4189	Contig
Rhizophagus irregularis DAOM 181602	Fungi	90.3752	Scaffold
Rhizopus delemar RA 99-880	Fungi	46.1489	Scaffold
Rhizopus microsporus B9738	Fungi	75.1334	Contig
Rhodosporidium toruloides MTCC 457	Fungi	20.0603	Scaffold
Rhodotorula glutinis ATCC 204091	Fungi	20.4789	Contig
Rhytidhysteron rufulum CBS 306.38	Fungi	39.8568	Contig
Rozella allomycis CSF55	Fungi	11.8593	Scaffold
Saccharomyces arboricola H-6	Fungi	11.6195	Chromosome
Saccharomyces bayanus 623-6C	Fungi	11.8653	Contig
Saccharomyces sp. 'boulardii'	Fungi	11.6369	Chromosome
Saccharomyces cerevisiae	Fungi	11.2113	Contig
Saccharomyces cerevisiae x Saccharomyces kudriavzevii VIN7	Fungi	23.3715	Contig
Saccharomyces kudriavzevii FM1056	Fungi	10.0757	Scaffold
Saccharomyces mikatae IFO 1815	Fungi	11.4703	Contig
Saccharomyces paradoxus NRRL Y-17217	Fungi	11.8726	Contig
Saccharomyces pastorianus CCY48 - 91	Fungi	24.2085	Contig
Saccharomycetaceae sp. 'Ashbya aceri'	Fungi	8.89452	Complete Genome
Saitoella complicata NRRL Y-17804	Fungi	14.2209	Contig
Scheffersomyces stipitis CBS 6054	Fungi	15.4412	Chromosome
Schizophyllum commune H4-8	Fungi	38.482	Scaffold
Schizosaccharomyces cryophilus OY26	Fungi	11.5548	Scaffold
Schizosaccharomyces japonicus yFS275	Fungi	11.3004	Scaffold

Schizosaccharomyces octosporus yFS286	Fungi	11.6345	Scaffold
Schizosaccharomyces pombe	Fungi	12.5913	Chromosome with gaps
Sclerotinia borealis F-4157	Fungi	39.4501	Scaffold
Sclerotinia homoeocarpa LT30	Fungi	29.7255	Contig
Sclerotinia sclerotiorum 1980 UF-70	Fungi	38.5328	Scaffold
Serpula lacrymans var. lacrymans S7.3	Fungi	46.9938	Scaffold
Setosphaeria turcica Et28A	Fungi	43.0135	Scaffold
Shiraia sp. slf14	Fungi	32.0674	Contig
Sordaria macrospora	Fungi	39.955	Contig
Spathaspora arborariae UFMG-19.1A	Fungi	12.8706	Scaffold
Spathaspora passalidarum NRRL Y-27907	Fungi	13.1821	Scaffold
Mycosphaerella populorum SO2202	Fungi	29.3521	Scaffold
Mycosphaerella populicola P02.02b	Fungi	33.1888	Scaffold
Spizellomyces punctatus DAOM BR117	Fungi	23.906	Contig
Sporisorium reilianum SRZ2	Fungi	18.4769	Chromosome
Spraguea lophii 42_110	Fungi	4.98088	Contig
Stereum hirsutum FP-91666 SS1	Fungi	46.5116	Scaffold
Talaromyces marneffei ATCC 18224	Fungi	28.6439	Scaffold
Talaromyces stipitatus ATCC 10500	Fungi	35.6854	Scaffold
Taphrina deformans PYCC 5710	Fungi	13.3942	Scaffold
Tetrapisispora blattae CBS 6284	Fungi	14.0486	Chromosome with gaps
Tetrapisispora phaffii CBS 4417	Fungi	12.1151	Chromosome
Thermomyces lanuginosus SSBP	Fungi	19.1555	Contig
Thielavia terrestris NRRL 8126	Fungi	36.9123	Complete Genome
Togninia minima UCRPA7	Fungi	47.4654	Scaffold
Tolypocladium inflatum NRRL8044	Fungi	30.2177	Contig
Torulaspora delbrueckii	Fungi	9.22068	Chromosome with gaps
Trachipleistophora hominis	Fungi	8.49818	Scaffold
Trametes versicolor FP-101664 SS1	Fungi	44.794	Scaffold
Tremella mesenterica DSM 1558	Fungi	28.6399	Scaffold
Trichoderma atroviride IMI 206040	Fungi	36.1437	Contig
Trichoderma hamatum GD12	Fungi	38.1761	Scaffold
Trichoderma pseudokoningii SMF2	Fungi	31.7431	Scaffold
Trichoderma reesei QM6a	Fungi	33.3957	Scaffold
Trichoderma virens FT-333	Fungi	38.6301	Contig
Trichophyton equinum CBS 127.97	Fungi	24.1582	Scaffold
Trichophyton rubrum CBS 100081	Fungi	23.0618	Scaffold
Trichophyton tonsurans CBS 112818	Fungi	22.9886	Scaffold
Trichophyton verrucosum HKI 0517	Fungi	22.541	Scaffold
Trichosporon asahii var. asahii CBS 2479	Fungi	24.5403	Scaffold

Tuber melanosporum	Fungi	124.946	Scaffold
Uncinocarpus reesii 1704	Fungi	22.3497	Scaffold
Ustilago hordei	Fungi	21.1507	Contig
Ustilago maydis 521	Fungi	19.7993	Contig
Vanderwaltozyma polyspora DSM 70294	Fungi	14.6746	Scaffold
Vavraia culicis 'floridensis'	Fungi	6.11869	Scaffold
Verticillium alfalfae VaMs.102	Fungi	32.863	Scaffold
Verticillium dahliae JR2	Fungi	36.1503	Complete Genome
Vittaforma corneae ATCC 50505	Fungi	3.21352	Scaffold
Volvariella volvacea PYd21	Fungi	36.6267	Contig
Wallemia ichthyophaga EXF-994	Fungi	9.65563	Scaffold
Wallemia sebi CBS 633.66	Fungi	9.81559	Scaffold
Wickerhamomyces anomalus NRRL Y-366	Fungi	26.555	Scaffold
Wickerhamomyces ciferrii	Fungi	15.9012	Contig
Wolfiporia cocos MD-104 SS10	Fungi	50.4836	Scaffold
Yarrowia lipolytica CLIB122	Fungi	20.5509	Chromosome with gaps
Zygosaccharomyces bailii ISA1307	Fungi	21.1411	Contig
Zygosaccharomyces rouxii	Fungi	9.76464	Chromosome with gaps
Zymoseptoria ardabiliae STIR04_1.1.1	Fungi	30.9575	Contig
Zymoseptoria passerinii SP63	Fungi	28.7863	Contig
Zymoseptoria pseudotritici STIR04_2.2.1	Fungi	31.624	Contig
Zymoseptoria tritici STIR04 A48b	Fungi	31.7941	Contig
Acanthamoeba castellanii	Protists	46.7146	Scaffold
Albugo candida	Protists	32.9905	Contig
Angomonas deanei	Protists	23.0794	Contig
Angomonas desouzai	Protists	24.2509	Contig
Ascogregarina taiwanensis	Protists	6.14941	Contig
Astrammina rara	Protists	1.45009	Contig
Aureococcus anophagefferens	Protists	56.6606	Scaffold
Babesia bovis	Protists	8.17971	Chromosome
Babesia equi strain WA	Protists	11.6745	Chromosome
Bigelowiella natans CCMP2755	Other	91.4059	Contig
Blastocystis hominis	Protists	18.8172	Scaffold
Crithidia acanthocephali	Protists	33.7832	Contig
Crithidia fasciculata	Protists	40.2474	Scaffold
Cryptosporidium hominis	Protists	8.74357	Contig
Cryptosporidium muris RN66	Protists	9.24525	Scaffold
Cryptosporidium parvum Iowa II	Protists	9.10232	Chromosome
Dictyostelium citrinum	Protists	26.2518	Scaffold
Dictyostelium discoideum AX4	Protists	34.205	Chromosome

Dictyostelium fasciculatum	Protists	31.0192	Scaffold
Dictyostelium firmibasis	Protists	30.5597	Scaffold
Dictyostelium intermedium	Protists	30.5295	Scaffold
Dictyostelium purpureum	Protists	32.9672	Scaffold
Ectocarpus siliculosus	Other	195.811	Chromosome
Eimeria acervulina	Protists	45.8306	Scaffold
Eimeria brunetti	Protists	66.8902	Scaffold
Eimeria maxima	Protists	45.9751	Scaffold
Eimeria mitis	Protists	60.4151	Scaffold
Eimeria necatrix	Protists	55.0079	Scaffold
Eimeria praecox	Protists	60.0833	Scaffold
Eimeria tenella	Protists	51.8596	Scaffold
Emiliania huxleyi CCMP1516	Protists	167.676	Scaffold
Endotrypanum monterogeii	Protists	32.5193	Scaffold
Entamoeba dispar SAW760	Protists	30.6332	Scaffold
Entamoeba histolytica HM-1:IMSS	Protists	20.8354	Scaffold
Entamoeba invadens IP1	Protists	40.8783	Scaffold
Entamoeba nuttalli P19	Protists	14.4	Scaffold
Giardia intestinalis	Protists	10.7039	Contig
Gregarina niphandrodes	Protists	14.0091	Scaffold
Guillardia theta CCMP2712	Other	87.1453	Scaffold
Hammondia hammondi	Protists	67.7014	Scaffold
Herpetomonas muscarum	Protists	30.8448	Contig
Heterococcus sp. DN1	Protists	60.7427	Contig
Hyaloperonospora arabidopsidis Emoy2	Protists	78.3805	Scaffold
Hyphochytrium catenoides	Protists	85.1426	Contig
Ichthyophthirius multifiliis	Protists	48.8	Scaffold
Leishmania aethiopica L147	Protists	31.9862	Scaffold
Leishmania amazonensis	Protists	29.0293	Scaffold
Leishmania arabica	Protists	31.4384	Scaffold
Leishmania braziliensis MHOM/BR/75/M2903	Protists	35.2112	Scaffold
Leishmania donovani	Protists	32.445	Chromosome with gaps
Leishmania enriettii	Protists	30.7805	Scaffold
Leishmania gerbilli	Protists	31.3986	Scaffold
Leishmania infantum JPCM5	Protists	32.1221	Chromosome
Leishmania major strain Friedlin	Protists	32.8551	Complete Genome
Leishmania mexicana MHOM/GT/2001/U1103	Protists	32.1087	Chromosome
Leishmania panamensis	Protists	30.6888	Chromosome with gaps
Leishmania sp. MAR LEM2494	Protists	30.873	Scaffold
Leishmania tropica L590	Protists	32.989	Scaffold
Leishmania turanica	Protists	32.32	Scaffold

Naegleria fowleri	Protists	27.7913	Contig
Naegleria gruberi	Protists	40.9641	Scaffold
Nannochloropsis gaditana	Protists	27.5893	Chromosome
Nannochloropsis oceanica	Protists	27.6399	Contig
Neospora caninum Liverpool	Protists	57.5474	Chromosome with gaps
Oxytricha trifallax	Protists	496.291	Contig
Paramecium tetraurelia	Protists	72.0945	Scaffold
Perkinsus marinus ATCC 50983	Protists	86.6051	Scaffold
Phaeodactylum tricornutum CCAP 1055/1	Other	27.4507	Chromosome
Physarum polycephalum	Protists	205.176	Scaffold
Phytomonas serpens 9T	Protists	25.6932	Contig
Phytophthora alni subsp. alni	Protists	236	Scaffold
Phytophthora cambivora	Protists	230.616	Scaffold
Phytophthora capsici LT1534	Protists	56.0343	Contig
Phytophthora cryptogea	Protists	103.037	Scaffold
Phytophthora infestans T30-4	Protists	228.544	Scaffold
Phytophthora kernoviae	Protists	37.2873	Scaffold
Phytophthora lateralis	Protists	46.4151	Scaffold
Phytophthora parasitica	Protists	48.0752	Scaffold
Phytophthora pinifolia	Protists	131.905	Scaffold
Phytophthora ramorum	Protists	66.6524	Scaffold
Phytophthora sojae	Protists	82.5976	Scaffold
Plasmodium berghei	Protists	17.9546	Scaffold
Plasmodium chabaudi chabaudi	Protists	16.8934	Scaffold
Plasmodium cynomolgi strain B	Protists	26.1813	Chromosome
Plasmodium falciparum 3D7	Protists	23.2703	Chromosome with gaps
Plasmodium knowlesi strain H	Protists	23.4622	Chromosome with gaps
Plasmodium vivax	Protists	27.0137	Chromosome
Plasmodium yoelii 17X	Protists	22.2224	Scaffold
Polysphondylium pallidum PN500	Protists	32.9702	Scaffold
Polysphondylium violaceum	Protists	25.7234	Contig
Pseudoperonospora cubensis	Protists	64.3328	Contig
Pythium aphanidermatum DAOM BR444	Protists	35.8768	Scaffold
Pythium arrhenomanes ATCC 12531	Protists	44.6726	Scaffold
Pythium irregulare DAOM BR486	Protists	42.9681	Scaffold
Pythium iwayamai DAOM BR242034	Protists	43.1992	Scaffold
Pythium ultimum DAOM BR144	Protists	44.9135	Scaffold
Pythium vexans DAOM BR484	Protists	33.8449	Scaffold
Reticulomyxa filosa	Protists	101.867	Contig
Saprolegnia diclina VS20	Protists	62.8858	Scaffold
Saprolegnia parasitica CBS 223.65	Protists	53.1316	Scaffold

Spironucleus salmonicida	Protists	12.9546	Scaffold
Strigomonas culicis	Protists	23.5872	Contig
Strigomonas galati	Protists	27.235	Contig
Strigomonas oncopelti	Protists	24.9629	Contig
Stylonychia lemnae	Protists	50.1645	Contig
Symbiodinium minutum Mf 1.05b.01	Protists	609.476	Scaffold
Tetrahymena borealis	Protists	93.5061	Scaffold
Tetrahymena elliotti 4EA	Protists	90.8376	Scaffold
Tetrahymena malaccensis 436	Protists	106.672	Scaffold
Tetrahymena thermophila SB210	Protists	103.014	Scaffold
Thalassiosira oceanica	Other	92.0438	Contig
Thalassiosira pseudonana CCMP1335	Other	32.4374	Chromosome
Amastigomonas sp. ATCC 50062	Protists	28.6806	Scaffold
Theileria annulata strain Ankara	Protists	8.35843	Chromosome
Theileria parva	Protists	8.34761	Chromosome
Toxoplasma gondii	Protists	63.0827	Contig
Trichomonas vaginalis G3	Protists	176.42	Scaffold
Trypanosoma brucei brucei TREU927	Protists	26.0755	Chromosome
Trypanosoma congolense IL3000	Protists	18.8055	Contig
Trypanosoma cruzi	Protists	89.9375	Scaffold
Trypanosoma vivax Y486	Protists	24.7793	Contig
Acromyrmex echinatior	Animals	295.945	Scaffold
Acropora digitifera	Animals	364.965	Contig
Acyrthosiphon pisum	Animals	541.692	Scaffold
Adineta vaga	Animals	217.934	Scaffold
Aedes aegypti	Animals	1376.42	Scaffold
Alatina moseri	Animals	1544.15	Contig
Amphimedon queenslandica	Animals	166.7	Scaffold
Angiostrongylus cantonensis	Animals	6.2549	Contig
Anopheles albimanus	Animals	170.508	Scaffold
Anopheles arabiensis	Animals	246.568	Scaffold
Anopheles atroparvus	Animals	224.29	Scaffold
Anopheles christyi	Animals	172.659	Scaffold
Anopheles culicifacies	Animals	202.999	Scaffold
Anopheles darlingi	Animals	136.936	Contig
Anopheles dirus	Animals	216.308	Scaffold
Anopheles epiroticus	Animals	223.487	Scaffold
Anopheles farauti	Animals	183.103	Scaffold
Anopheles funestus	Animals	225.224	Scaffold
Anopheles gambiae str. PEST	Animals	265.027	Chromosome
Anopheles maculatus	Animals	141.894	Scaffold
Anopheles melas	Animals	224.162	Scaffold
Anopheles merus	Animals	288.049	Scaffold

Anopheles minimus	Animals	201.793	Scaffold
Anopheles nili	Animals	98.32	Contig
Anopheles quadriannulatus	Animals	283.829	Scaffold
Anopheles sinensis	Animals	375.764	Scaffold
Anopheles stephensi	Animals	221.324	Scaffold
Anoplophora glabripennis	Animals	707.712	Scaffold
Apis dorsata	Animals	230.34	Scaffold
Apis florea	Animals	230.468	Scaffold
Apis mellifera	Animals	250.287	Chromosome
Aplysia californica	Animals	927.31	Scaffold
Ascaris suum	Animals	269.56	Scaffold
Athalia rosae	Animals	163.838	Scaffold
Atta cephalotes	Animals	317.691	Scaffold
Biomphalaria glabrata	Animals	916.374	Scaffold
Bombus impatiens	Animals	249.185	Scaffold
Bombus terrestris	Animals	248.654	Chromosome
Bombyx mori	Animals	481.819	Scaffold
Botryllus schlosseri	Animals	579.633	Scaffold
Branchiostoma floridae	Animals	521.895	Scaffold
Brugia malayi	Animals	93.6591	Scaffold
Bursaphelenchus xylophilus	Animals	73.0857	Contig
Caenorhabditis angaria	Animals	79.7615	Scaffold
Caenorhabditis brenneri	Animals	190.37	Scaffold
Caenorhabditis briggsae AF16	Animals	108.479	Chromosome
Caenorhabditis elegans	Animals	100.286	Chromosome
Caenorhabditis japonica	Animals	166.256	Scaffold
Caenorhabditis remanei	Animals	145.443	Scaffold
Caenorhabditis sp. 11 KK-2011	Animals	79.3214	Scaffold
Camponotus floridanus	Animals	232.685	Scaffold
Capitella teleta	Animals	333.283	Scaffold
Capsaspora owczarzaki ATCC 30864	Protists	28.0438	Scaffold
Ceratitis capitata	Animals	484.789	Scaffold
Ceratosolen solmsi marchali	Animals	277.059	Scaffold
Ciona intestinalis	Animals	115.227	Chromosome
Ciona savignyi	Animals	587.353	Scaffold
Clonorchis sinensis	Animals	547.288	Scaffold
Crassostrea gigas	Animals	557.718	Scaffold
Culex quinquefasciatus	Animals	579.042	Scaffold
Danaus plexippus	Animals	272.853	Scaffold
Daphnia pulex	Animals	197.206	Scaffold
Dendroctonus ponderosae	Animals	252.848	Scaffold
Diaphorina citri	Animals	485.705	Scaffold
Drosophila albomicans	Animals	253.56	Scaffold

Drosophila ananassae	Animals	230.993	Scaffold
Drosophila biarmipes	Animals	169.379	Scaffold
Drosophila bipectinata	Animals	167.264	Scaffold
Drosophila elegans	Animals	171.268	Scaffold
Drosophila erecta	Animals	152.712	Scaffold
Drosophila eugracilis	Animals	156.942	Scaffold
Drosophila ficusphila	Animals	152.439	Scaffold
Drosophila grimshawi	Animals	200.468	Scaffold
Drosophila kikkawai	Animals	164.293	Scaffold
Drosophila melanogaster	Animals	143.726	Chromosome
Drosophila miranda	Animals	136.729	Chromosome
Drosophila mojavensis	Animals	193.826	Scaffold
Drosophila persimilis	Animals	188.374	Scaffold
Drosophila pseudoobscura pseudoobscura	Animals	152.696	Chromosome
Drosophila rhopaloa	Animals	197.376	Scaffold
Drosophila sechellia	Animals	166.592	Scaffold
Drosophila simulans	Animals	124.966	Chromosome
Drosophila suzukii	Animals	232.923	Scaffold
Drosophila takahashii	Animals	182.107	Scaffold
Drosophila virilis	Animals	206.027	Scaffold
Drosophila willistoni	Animals	235.516	Scaffold
Drosophila yakuba	Animals	165.71	Chromosome
Echinococcus granulosus	Animals	110.838	Contig
Echinococcus multilocularis	Animals	113.769	Scaffold
Elaeophora elaphi	Animals	1.48083	Scaffold
Ephemera danica	Animals	475.911	Scaffold
Fonticula alba	Protists	31.2965	Scaffold
Haemonchus contortus	Animals	319.758	Scaffold
Harpegnathos saltator	Animals	294.466	Scaffold
Heliconius melpomene melpomene	Animals	273.786	Scaffold
Helobdella robusta	Animals	235.376	Scaffold
Heterodera glycines	Animals	81.908	Scaffold
Heterorhabditis bacteriophora	Animals	76.9743	Scaffold
Hydra vulgaris	Animals	852.171	Scaffold
Ixodes scapularis	Animals	1765.38	Scaffold
Ladona fulva	Animals	1158.11	Scaffold
Lepeophtheirus salmonis	Animals	790.052	Contig
Leptinotarsa decemlineata	Animals	1170.24	Scaffold
Limulus polyphemus	Animals	1828.26	Scaffold
Linepithema humile	Animals	219.501	Scaffold
Loa loa	Animals	86.8069	Contig
Lottia gigantea	Animals	359.506	Scaffold

Lutzomyia longipalpis	Animals	154.229	Scaffold
Lytechinus variegatus	Animals	951.76	Scaffold
Manduca sexta	Animals	419.424	Scaffold
Mayetiola destructor	Animals	185.828	Scaffold
Megachile rotundata	Animals	272.661	Scaffold
Megaselia scalaris	Animals	303.386	Contig
Meloidogyne hapla	Animals	53.013	Contig
Meloidogyne incognita	Animals	82.095	Contig
Mengenilla moldrzyki	Animals	155.727	Contig
Mesobuthus martensii	Animals	925.546	Contig
Metaseiulus occidentalis	Animals	151.724	Scaffold
Monosiga brevicollis MX1	Protists	41.7099	Scaffold
Musca domestica	Animals	750.404	Scaffold
Nasonia giraulti	Animals	283.607	Scaffold
Nasonia longicornis	Animals	285.726	Scaffold
Nasonia vitripennis	Animals	295.781	Chromosome
Necator americanus	Animals	244.075	Scaffold
Nematostella vectensis	Animals	356.614	Scaffold
Oikopleura dioica	Animals	70.4715	Scaffold
Onchocerca volvulus	Animals	96.4278	Scaffold
Panagrellus redivivus	Animals	65.0931	Scaffold
Parasteatoda tepidariorum	Animals	1443.91	Scaffold
Patiria miniata	Animals	811.029	Scaffold
Pediculus humanus corporis	Animals	110.781	Scaffold
Phlebotomus papatasi	Animals	363.768	Scaffold
Plutella xylostella	Animals	393.455	Scaffold
Pogonomyrmex barbatus	Animals	235.646	Scaffold
Priacma serrata	Animals	12.0783	Contig
Priapulus caudatus	Animals	420.178	Scaffold
Pristionchus pacificus	Animals	133.635	Contig
Rhipicephalus microplus	Animals	144.692	Contig
Rhodnius prolixus	Animals	702.645	Scaffold
Saccoglossus kowalevskii	Animals	775.84	Scaffold
Schistosoma japonicum	Animals	402.743	Scaffold
Schistosoma mansoni	Animals	364.538	Chromosome
Schmidtea mediterranea	Animals	700.726	Scaffold
Solenopsis invicta	Animals	396.009	Scaffold
Steinernema monticolum	Animals	89.159	Scaffold
Strigamia maritima	Animals	176.211	Scaffold
Strongylocentrotus purpuratus	Animals	936.581	Scaffold
Tetranychus urticae	Animals	90.8155	Scaffold
Tribolium castaneum	Animals	210.265	Chromosome
Trichinella spiralis	Animals	63.5254	Scaffold

Trichoplax adhaerens	Animals	105.632	Scaffold
Varroa destructor	Animals	294.134	Contig
Wuchereria bancrofti	Animals	81.5092	Contig
Alligator mississippiensis	Animals	2174.26	Scaffold
Alligator sinensis	Animals	2270.57	Scaffold
Amazona vittata	Animals	1175.4	Scaffold
Anas platyrhynchos	Animals	1105.05	Scaffold
Anolis carolinensis	Animals	1799.14	Chromosome
Anoplopoma fimbria	Animals	699.326	Contig
Apalone spinifera	Animals	1931.08	Scaffold
Ara macao	Animals	1204.7	Scaffold
Astyanax mexicanus	Animals	1191.24	Scaffold
Callorhinchus milii	Animals	974.499	Scaffold
Chelonia mydas	Animals	2208.41	Scaffold
Chrysemys picta bellii	Animals	2365.77	Chromosome
Columba livia	Animals	1107.99	Scaffold
Coturnix japonica	Animals	531.96	Scaffold
Danio rerio	Animals	1371.72	Chromosome
Dicentrarchus labrax	Animals	98.2281	Contig
Falco cherrug	Animals	1174.81	Scaffold
Falco peregrinus	Animals	1171.97	Scaffold
Ficedula albicollis	Animals	1118.34	Chromosome
Gadus morhua	Animals	824.311	Scaffold
Gallus gallus	Animals	1046.93	Chromosome
Gasterosteus aculeatus	Animals	446.611	Contig
Geospiza fortis	Animals	1065.29	Scaffold
Haplochromis burtoni	Animals	831.412	Scaffold
Labeotropheus fuelleborni	Animals	70.8584	Scaffold
Latimeria chalumnae	Animals	2860.59	Scaffold
Lepisosteus oculatus	Animals	945.878	Chromosome
Lethenteron camtschaticum	Animals	1030.66	Scaffold
Leucoraja erinacea	Animals	1555.44	Contig
Maylandia zebra	Animals	849.595	Scaffold
Mchenga conophoros	Animals	73.4256	Scaffold
Melanochromis auratus	Animals	68.2386	Scaffold
Meleagris gallopavo	Animals	1128.34	Chromosome
Melopsittacus undulatus	Animals	1117.37	Scaffold
Neolamprologus brichardi	Animals	847.91	Scaffold
Nothobranchius furzeri	Animals	5.31692	Contig
Nothobranchius kuhntae	Animals	5.23461	Contig
Ophiophagus hannah	Animals	1594.07	Contig
Oreochromis niloticus	Animals	927.696	Chromosome
Oryzias latipes	Animals	869.818	Chromosome

Pelodiscus sinensis	Animals	2202.48	Scaffold
Petromyzon marinus	Animals	885.535	Scaffold
Poecilia formosa	Animals	748.923	Scaffold
Pseudopodoces humilis	Animals	1043	Scaffold
Pundamilia nyererei	Animals	830.133	Scaffold
Python bivittatus	Animals	1435.05	Scaffold
Rhamphochromis esox	Animals	71.2951	Scaffold
Salmo salar	Animals	3047.41	Contig
Sebastes nigrocinctus	Animals	687.55	Scaffold
Sebastes rubrivinctus	Animals	756.297	Scaffold
Taeniopygia guttata	Animals	1232.14	Chromosome
Takifugu flavidus	Animals	378.032	Scaffold
Takifugu rubripes	Animals	391.485	Chromosome
Tetraodon nigroviridis	Animals	342.403	Contig
Thunnus orientalis	Animals	684.497	Contig
Xenopus (Silurana) tropicalis	Animals	1437.53	Scaffold
Xiphophorus maculatus	Animals	729.664	Scaffold
Zonotrichia albicollis	Animals	1052.6	Scaffold
Ailuropoda melanoleuca	Animals	2299.49	Scaffold
Balaenoptera acutorostrata scammoni	Animals	2431.69	Scaffold
Bos indicus	Animals	2673.95	Chromosome
Bos grunniens mutus	Animals	2645.16	Scaffold
Bos taurus	Animals	2670.04	Chromosome
Bubalus bubalis	Animals	2836.17	Scaffold
Callithrix jacchus	Animals	2914.96	Chromosome
Camelus ferus	Animals	2009.19	Scaffold
Canis lupus familiaris	Animals	2410.98	Chromosome
Capra hircus	Animals	2635.85	Chromosome
Cavia porcellus	Animals	2723.22	Scaffold
Ceratotherium simum simum	Animals	2464.37	Scaffold
Chinchilla lanigera	Animals	2390.87	Scaffold
Chlorocebus sabaeus	Animals	2789.66	Chromosome
Choloepus hoffmanni	Animals	3286.01	Scaffold
Chrysochloris asiatica	Animals	4210.11	Scaffold
Condylura cristata	Animals	1769.66	Scaffold
Cricetulus griseus	Animals	2399.79	Scaffold
Dasypus novemcinctus	Animals	3631.52	Scaffold
Daubentonia madagascariensis	Animals	2855.37	Contig
Dipodomys ordii	Animals	2236.37	Scaffold
Echinops telfairi	Animals	2947.02	Scaffold
Eidolon helvum	Animals	1837.75	Scaffold
Elephantulus edwardii	Animals	3843.98	Scaffold
Eptesicus fuscus	Animals	2026.63	Scaffold

Equus caballus	Animals	2474.93	Chromosome
Erinaceus europaeus	Animals	2715.72	Scaffold
Felis catus	Animals	2455.54	Chromosome
Gorilla gorilla gorilla	Animals	3035.66	Chromosome
Heterocephalus glaber	Animals	2618.2	Scaffold
Homo sapiens	Animals	3209.29	Chromosome
Ictidomys tridecemlineatus	Animals	1913.37	Contig
Jaculus jaculus	Animals	2835.25	Scaffold
Leptonychotes weddellii	Animals	3156.9	Scaffold
Lipotes vexillifer	Animals	2429.21	Scaffold
Loxodonta africana	Animals	3196.74	Scaffold
Macaca fascicularis	Animals	2946.84	Chromosome
Macaca mulatta	Animals	3097.39	Chromosome
Macropus eugenii	Animals	3075.18	Scaffold
Megaderma lyra	Animals	1735.93	Scaffold
Mesocricetus auratus	Animals	2504.93	Scaffold
Microcebus murinus	Animals	2902.27	Scaffold
Microtus ochrogaster	Animals	2287.34	Chromosome
Monodelphis domestica	Animals	3598.44	Chromosome
Mustela putorius furo	Animals	2410.88	Scaffold
Mus musculus	Animals	2798.79	Chromosome
Myotis brandtii	Animals	2107.24	Scaffold
Myotis davidii	Animals	2059.8	Scaffold
Myotis lucifugus	Animals	2034.58	Scaffold
Nomascus leucogenys	Animals	2962.06	Chromosome
Ochotona princeps	Animals	2229.84	Scaffold
Octodon degus	Animals	2995.89	Scaffold
Odobenus rosmarus divergens	Animals	2400.15	Scaffold
Odocoileus virginianus	Animals	37.6562	Contig
Orcinus orca	Animals	2372.92	Scaffold
Ornithorhynchus anatinus	Animals	1995.61	Chromosome
Orycteropus afer afer	Animals	4444.08	Scaffold
Oryctolagus cuniculus	Animals	2737.46	Chromosome
Otolemur garnettii	Animals	2519.72	Scaffold
Ovis aries	Animals	2619.05	Chromosome
Panthera tigris altaica	Animals	2391.08	Scaffold
Pantholops hodgsonii	Animals	2696.89	Scaffold
Pan paniscus	Animals	2869.21	Scaffold
Pan troglodytes	Animals	3309.58	Chromosome
Papio anubis	Animals	2948.4	Chromosome
Peromyscus maniculatus bairdii	Animals	2630.54	Scaffold
Physeter catodon	Animals	2280.73	Scaffold
Pongo abelii	Animals	3441.24	Chromosome

Procavia capensis	Animals	3602.18	Scaffold
Pteronotus parnellii	Animals	1960.32	Scaffold
Pteropus alecto	Animals	1985.98	Scaffold
Pteropus vampyrus	Animals	2198.28	Scaffold
Rattus norvegicus	Animals	2870.18	Chromosome
Rhinolophus ferrumequinum	Animals	1926.44	Scaffold
Saimiri boliviensis boliviensis	Animals	2608.59	Scaffold
Sarcophilus harrisii	Animals	3174.69	Scaffold
Sorex araneus	Animals	2423.16	Scaffold
Sus scrofa	Animals	2808.53	Chromosome
Tarsius syrichta	Animals	3453.86	Scaffold
Trichechus manatus latirostris	Animals	3103.81	Scaffold
Tupaia belangeri	Animals	2137.23	Contig
Tupaia chinensis	Animals	2846.58	Scaffold
Tursiops truncatus	Animals	2551.42	Scaffold
Vicugna pacos	Animals	2172.21	Scaffold
