# Supplementary material for: The diversification of PHIS transposon superfamily in eukaryotes
Source: Mob DNA. 2015 Jun 24;6:12. doi: 10.1186/s13100-015-0043-7 (PMC4482050; doi:10.1186/s13100-015-0043-7)

Figure S1. (A) Sequence alignments for ISL2EU-2_Pxyl family. The terminal inverted repeats (TIRs) and flanking sequences (10 bp) are shown. (B) An example of alignments of the flanking sequences of ISL2EU-2_Pxyl insertion with a paralogous sequences found within the same genome but devoid of the transposon. The TIRs of the element are underlined.

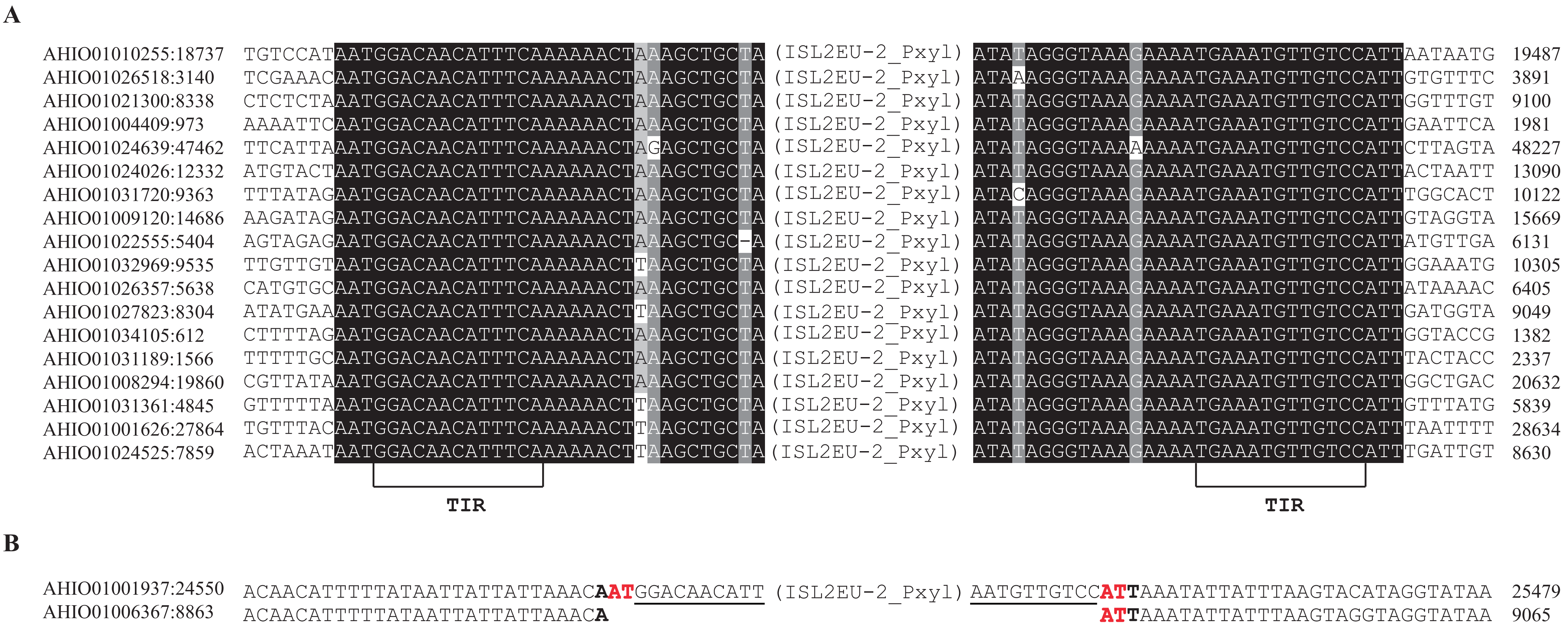

Figure S2. Speculated transposition mechanism of each PHIS group.

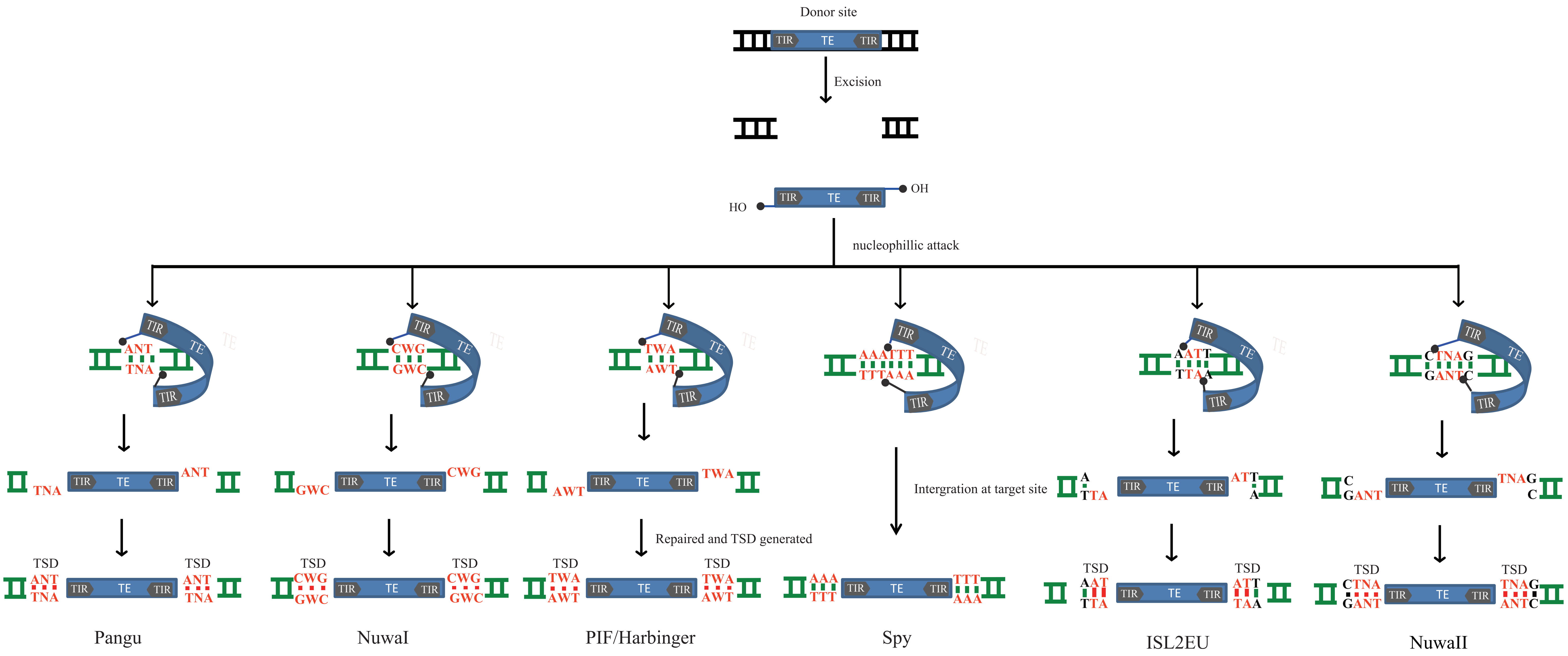

Figure S3. The alignment of DDE domain of Pangu and Nuwa groups after redundancy elimination. Distances between the conserved blocks are indicated in the number of amino acid residues. Conserved residues within each superfamily are highlighted in the same color. The DDE triad identified here is marked with asterisks below alignments.

```
                     D                                                        D                              E
Pangu_10_Ccri  : VGFLDGSRLRTARPGE(3)QNALYNA-KYGHNLAYQAVNCPDGLILDYQGPFGGCHADG(22)-NDAAYAR(8)-RGH(1)-NPTSLEERAFNALMNSLRITAEWGFG
Pangu_Tsti     : WGCIDGTFRATCRPSK(1)QRIAYSGYKKRHGFKYQGIITPDGMVLSLIGPFEGKIADS(25)-GDIGYQG(8)-TPF(3)-GELSARQIEINDRISAHRISVEQIFG
Pangu-1_Tmel   : WGFVDGTMRPFCRPGE(1)QGTFYSGYKKSHAFKFQSIVSPDGLLCSLIGPVPGPVGDW(30)-GDSAYAP(8)-GPY(5)-KPLTMEEEAANLVMSGERIVVEWGFA
Pangu_8_Ccri   : VGFMDCTKIQMCRPGG(4)QRSVYSGHKRFHCLIYQSITTPDGLMMHLFGPEVWRRHDL(26)-GDAAYVL(8)-IAF(2)-TIATPTEVEYNKKMSAAREAVEWSYK
Pangu-3_Tmel   : WGFIDGTIRGIARPSR(1)QRTCYNGWKHKHCLKYHAIITPDGLISHLFGPVDGRRNDA(27)-GDPAYSI(8)-SPY(2)-ARISHNQRLWNRSMSRVRIVAEWAFK
Pangu-2_PInf   : IGFIDGTVRGIARPVR(1)QRQCYNGHKRKHALKYQGVMAPDGLFIDFYGPVLGRRHDV(23)-GDPAYPN(8)-VGF(2)-SRLSRDQEAFNGAMSAVRVSVEWGFG
Pangu_7_Ccri   : VGFIDGTVVGVARPKG(3)QLVVYNGHKRKHSLKYQAVNTPDGMILHAYGPAEGRRHDW(26)-GDSGYNW(8)-VPF(2)-SALNTHQTAFNSSMSGVRITVEWIFK
Pangu_2_Ccri   : TGFIDGTVIAIARPSR(3)QNVAYNGHKRKHALKYQAITSPDGLVLHAAGPIEGRRHDW(26)-GDSGYNP(8)-VPY(2)-SNLSAHQRAFNGAMSKGRVTVEWYFK
Pangu-2_LDec   : IGALDCTQIEIQKPGL(0)HGDEYICRKGYPSINVQATCNALEQFTSISAEWPGSVHDG(22)-GDSGYGL(8)-TPF(1)-PPRTHQEREFNLIHARERVIIERCFG
Pangu_9_Ccri   : VGFIDGTVIGVARPGG(3)QLVMYNGHKRKHAIKFQAVTAPDGLILHVCGPVEGRRHDW(26)-GDSGYAK(8)-VPY(2)-SNLSASQRAFNKAMSSVRITVEWAFK
Pangu_HVul     : WGFIDGTLRPISRPRE(1)QRILYNGHKRCHGIKFQSLVAPNGLIANLYGPVEGKRHDS(27)-GDPAYPH(8)-CGF(2)-ANISPEQLIWNKNMSKVRVAVEWVFG
Pangu-1_PInf   : WGFIDGTIRPICRPSK(1)QQAMYNGHKRVHALKFQTVVTPDGLISHLFGPVDGRRHDL(24)-GDPAYGC(8)-CPF(2)-CRVDVPQHELNKAMSAIRVSVEWSYG
Pangu-2_MLP    : VGFVDGTNIPLQRPGL(0)DGETFFDRKKRYSINCQVVCDCDRRITAFYTGWPGSCHDY(23)-GDSAYKI(8)-STF(3)-AANLVENKAFNTLVAQARIRNEHTIG
Pangu_CGig     : LGAVDGSHIPIKAPRE(0)NPNAYYNRKKFHSVVLLATCDANLQFTYVWTGNPGSTHDA(23)-GDSAFPL(8)-TPF(3)-GNLTRGQRLFNVCHSKTRQVIERSFG

NuwaII_CCle    : IGAIDGTLIHACIPTD(0)KQVPYRGRGGECFQNVMALCDFDMIFRYVVVGWEGTAHDS(24)-VDAAYSH(8)-APY(13)GRARGREEIFNHAHAKLRNVIERAFG
NuwaII_TRub    : VGSIDGCHXRIKPPGA(0)DAQCYFNRKLFYSIQLQAVCDHQXLFMDXFTGYPGSVHDS(22)-GDGGYPC(11)TPX(2)-XVRHMMAARFNRHHAKARSVIERALG
NuwaII_CSav    : VGAIDGCHIAIPKPAI(0)NGEDYWNRKSFYSINMQAIVDHHGRSTQ------------(11)-GDSAYPL(7)-KP-(0)-YPDNASERNFNKKHSSARRVVEQAFG
NuwaII_CPB1    : GGAIDGTHIPILAPEH(0)QGTQYINRKGYFSMVLQALVDHKGRFTNINVGWPGRVHDA(35)-GDPAYPL(8)-KPY(1)-GSLDRSQELFNYRLSKCRMVVECAFG
NuwaII-1_BFlo  : AGAIDGSHIPIKAPSK(0)NPNDFYNRKGFYSVILQGVVDHMSRFTMIDIGMPGSVHDA(36)-GDAAYPT(8)-KPY(3)-GALTREKFDFNYRQSRARMTVECAFG
NuwaII-2_BFlo  : AGAVDGSHIPINAPTK(0)DPTDFFNRKGYHSVILQGVVDHLYRFTDICVGRPGSVHDA(36)-GDAAYPQ(8)-KPY(3)-GALSREKFDFNYRQSRARMTVECAFG
NuwaII-4_XTro  : LGAIDCTHVAFNPPLD(0)KEHIFRNRKSYHSLNIQIVCDSKMNIMSIVSGFPGSSHDS(23)-GDAGYPC(8)-TPI(1)-RPHTRAESAFNEAHVRARSVIERTFG

NuwaI-4_DRer   : IGVIDGTLIPISTPSV(0)DGHTYICRKGYPAINCQVICDHNCLITDIVARWPGSTHDS(22)-GDSGYPL(8)-TPV(1)-NPVSNSEAHFNEAHRVARSTVERTLG
NuwaI_NVec     : IGCIDGTHVRIQGPPR(0)NENDFVNRKGFHSINVQAICDNKGLFTNVVAKWPGNAHDS(25)-GDSGYGC(8)-TPF(1)-HPETPKQEAYNEALGKTRVKIEQTFG
NuwaI-1_TFla   : IGVIDCTHIHIQAPHE(0)REWEFVNRKGRHSINVQLIGNADLQIINCVVRWPGSVHDS(22)-GDSGYPL(8)-TPF(1)-TVTSNSQQNFNYAHTSTRGTIERING
NuwaI-4_CGig   : IGAIDGTQIPIGMRTD(0)DEHLYVCRKGFHSINVQAVVDADLRFTNAVCKFPGATHDA(22)-GDSGYPL(8)-TPI(1)-NPRSGQEERYNSAHCRTRNAIERAFG
NuwaI-1_CGig   : IGAIDGTHIPIQAPST(0)HEYLFVNRKNFHSINVMAVCNSNLKFLNLVAKWPGSSHDA(23)-GDSAYPL(8)-TPV(1)-NPSNPHEEAYNDAHAKTRAPIERAFG
NuwaI-1_REso   : IGCIDGTHIPIIAPSV(0)NEGDYVNRKSFHSINVQIICDAANIITNVEAKWPGSVHDS(22)-GDRGYPC(8)-TPY(1)-DPEPGPQQRYNLANCRTRARIEMTIG
NuwaI_GAcu     : VGCIDGTHIPIIAPSE(0)NEADYVNRRSIHSINVQIICDAAHLITNVEAKWPGSVHDS(22)-GDRGYPC(8)-TPY(1)-EPEPGPQRRFNVAHCRTRARVEMTIG
NuwaI-1_TOri   : IGCIDGTHIPITAPSH(0)NEADYVNRKSIHSINVQIICDAAYIISNVEAKWPGSVHDS(22)-GDRGYPC(8)-TLY(1)-DPEPGPQQNFNRAHCRTRARVEMTIG
NuwaI-2_DRer   : IGAIDCTHIAIKAPSE(0)DEFAYVNRKHFHSINVQIICDAQMRLTNIVARWPGSTHDS(23)-GDRGYPL(8)-TPL(1)-NPQTDQERRYNDAHSHTRSVVERAIG
NuwaI-3_CGig   : VGAIDGTHVQIIAPSD(0)YENEYVNRHHYHSINTQVVFDPFHKIIDVVAKWPGSTHDS(24)-GDSGYPS(8)-TPF(1)-RPNQGCQTNYNRAHKKTRSIVERGIG
NuwaI_GMor     : VGAIDGTHIKIIAPSK(0)DEDVFVNRKKVHSINTQ---------------WPGSTHDS(24)-GDSGYPC(8)-TPY(1)-NPQPGAQLKYNIAHKHTRNVVERGIG
NuwaI-2_CGig   : VCCVDGTLIRIRCPVQ(0)NEAEFVCRKGYYALNVQMACDATYRFVNCVAKWPGSVHDS(22)-GDSGYAL(8)-VPY(1)-TPSNRAEENFNNALCRTRVTIEQAFG
NuwaI_LDec     : CGAVDGILINIDAPTN(0)NEPVYVDRHGKHSINCMAVCGPDLRFYYISANWPGSVHDA(25)-GDSAYPL(8)-PPY(1)-DPMNPAQTAFNRAHKRTRRIFENAFG
```

Figure S4. A pipeline for PHIS transposons identification. A hit with e-value less than 10-4 was considered as a homology sequence. Sequences with the e-value less than e-5, sequence length larger than 50 bp, and nucleotide sequence identity larger than 80% were classified as members of the same transposon family. Target site duplications (TSDs) were identified using the paralogous empty sites.

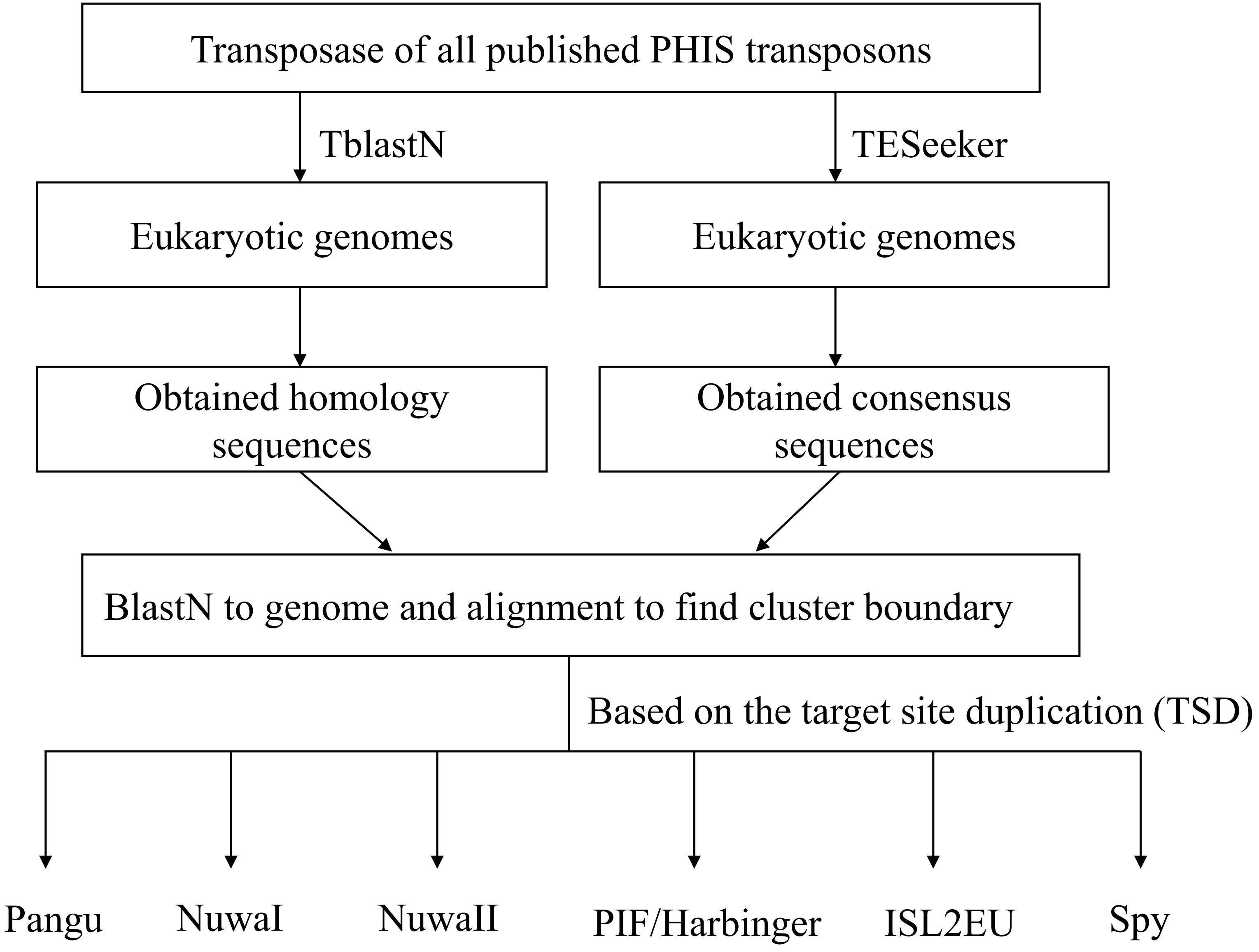

Supplement: Additional file 2: Figure S1. — (A) Sequence alignments for ISL2EU-2_Pxyl family. The terminal inverted repeats (TIRs) and flanking sequences (10 bp) are shown. (B) An example of alignments of the flanking sequences of ISL2EU-2_Pxyl insertion with a paralogous sequences found within the same genome but devoid of the transposon. The TIRs of the element are underlined. Figure S2. Speculated transposition mechanism of each PHIS groups. Figure S3. The alignment of DDE domain of Pangu and Nuwa groups after redundancy elimination. Distances between the conserved blocks are indicated in the number of amino acid residues. Conserved residues within each superfamily are highlighted in the same color. The DDE triad identified here is marked with asterisks below alignments. Figure S4. Pipeline for PHIS transposons identification. Where a hit with e value less than 10−4 was considered as a homology sequence. The ones with an e value less than e −5, sequence length larger than 50 bp, and nucleotide sequence identity larger than 80 % were classified as member of the same family. Target site duplications (TSDs) were identified using the paralogous empty sites. [file 13100_2015_43_MOESM2_ESM.pdf]
