# Supplementary material for: The diversification of PHIS transposon superfamily in eukaryotes
Source: Mob DNA. 2015 Jun 24;6:12. doi: 10.1186/s13100-015-0043-7 (PMC4482050; doi:10.1186/s13100-015-0043-7)
Supplement: Additional file 3: Table S4. — Positions of Pangu transposons in the corresponding genome. Table S6. Positions of NuwaI transposons in the corresponding genome. Table S8. Positions of NuwaII transposons in the corresponding genome. [file 13100_2015_43_MOESM3_ESM.pdf]

Table S4. Positions of *Pangu* transposons in the corresponding genome

Species	Families	Scaffold:start-end
Acropora digitifera	Pangu_ADig	dbj|BACK01001213.1|:1-2534
	Pangu_ADig	dbj|BACK01045489.1|:11598-8413
	Pangu_ADig	dbj|BACK01042934.1|:5121-8343
	Pangu_ADig	dbj|BACK01042934.1|:8343-5121
	Pangu_ADig	dbj|BACK01032808.1|:4473-9295
	Pangu_ADig	dbj|BACK01035181.1|:12931-11420
	Pangu_ADig	dbj|BACK01006697.1|:929-2119
	Pangu_ADig	dbj|BACK01024654.1|:1-1150
	Pangu_ADig	dbj|BACK01024653.1|:9480-10420
	Pangu_ADig	dbj|BACK01043413.1|:1860-7546
	Pangu_ADig	dbj|BACK01040586.1|:4535-7983
	Pangu_ADig	dbj|BACK01040586.1|:7983-4535
	Pangu_ADig	dbj|BACK01028732.1|:1-1087
	Pangu_ADig	dbj|BACK01028730.1|:6228-6897
	Pangu_ADig	dbj|BACK01024029.1|:8630-8846
	Pangu_ADig	dbj|BACK01028731.1|:486-620
	Pangu_ADig	dbj|BACK01028731.1|:120-43
	Pangu_ADig	dbj|BACK01045391.1|:2556-2489
	Pangu_ADig	dbj|BACK01038420.1|:879-937
	Pangu_ADig	dbj|BACK01051597.1|:984-915
	Pangu_ADig	dbj|BACK01002066.1|:971-1040
	Pangu_ADig	dbj|BACK01051418.1|:10874-10822
Aedes aegypti	Pangu_AAeg	gb|AAGE02018429.1|:36284-40830
	Pangu_AAeg	gb|AAGE02014591.1|:18237-27287
	Pangu_AAeg	gb|AAGE02019606.1|:6401-7169
	Pangu_AAeg	gb|AAGE02011928.1|:56561-64386
	Pangu_AAeg	gb|AAGE02008490.1|:4439-14775
	Pangu_AAeg	gb|AAGE02011736.1|:176602-184788
	Pangu_AAeg	gb|AAGE02005979.1|:36685-44528
	Pangu_AAeg	gb|AAGE02007208.1|:6160-6450
	Pangu_AAeg	gb|AAGE02027750.1|:27430-27720
	Pangu_AAeg	gb|AAGE02014591.1|:48425-48713
	Pangu_AAeg	gb|AAGE02001795.1|:180078-180367
	Pangu_AAeg	gb|AAGE02004366.1|:51609-51898
	Pangu_AAeg	gb|AAGE02028004.1|:14126-14413
	Pangu_AAeg	gb|AAGE02029992.1|:72962-73251
	Pangu_AAeg	gb|AAGE02008490.1|:42508-42796
	Pangu_AAeg	gb|AAGE02007814.1|:45817-46105
	Pangu_AAeg	gb|AAGE02006151.1|:159773-160062
	Pangu_AAeg	gb|AAGE02002748.1|:243055-243342
	Pangu_AAeg	gb|AAGE02010338.1|:341747-342037
	Pangu_AAeg	gb|AAGE02018391.1|:128327-128616

Pangu_AAeg	gb|AAGE02015896.1|:135693-135982
Pangu_AAeg	gb|AAGE02002121.1|:22668-22956
Pangu_AAeg	gb|AAGE02003653.1|:15107-15395
Pangu_AAeg	gb|AAGE02000147.1|:42622-42910
Pangu_AAeg	gb|AAGE02000147.1|:63727-64015
Pangu_AAeg	gb|AAGE02015611.1|:76354-76642
Pangu_AAeg	gb|AAGE02028287.1|:46600-46886
Pangu_AAeg	gb|AAGE02024541.1|:4946-5234
Pangu_AAeg	gb|AAGE02015098.1|:132419-132707
Pangu_AAeg	gb|AAGE02006339.1|:19733-20021
Pangu_AAeg	gb|AAGE02002711.1|:74880-75168
Pangu_AAeg	gb|AAGE02006941.1|:78510-78798
Pangu_AAeg	gb|AAGE02026958.1|:5997-6285
Pangu_AAeg	gb|AAGE02025797.1|:2535-2823
Pangu_AAeg	gb|AAGE02021745.1|:5681-5969
Pangu_AAeg	gb|AAGE02021197.1|:3236-3524
Pangu_AAeg	gb|AAGE02016393.1|:63865-64153
Pangu_AAeg	gb|AAGE02013647.1|:160335-160623
Pangu_AAeg	gb|AAGE02011723.1|:86271-86559
Pangu_AAeg	gb|AAGE02008243.1|:420547-420835
Pangu_AAeg	gb|AAGE02006261.1|:134993-135281
Pangu_AAeg	gb|AAGE02004148.1|:24883-25171
Pangu_AAeg	gb|AAGE02015829.1|:83109-83397
Pangu_AAeg	gb|AAGE02005254.1|:68203-68489
Pangu_AAeg	gb|AAGE02000676.1|:131182-131469
Pangu_AAeg	gb|AAGE02000867.1|:107086-107375
Pangu_AAeg	gb|AAGE02022141.1|:47132-47420
Pangu_AAeg	gb|AAGE02022141.1|:75892-76180
Pangu_AAeg	gb|AAGE02015907.1|:59123-59410
Pangu_AAeg	gb|AAGE02033319.1|:7033-7320
Pangu_AAeg	gb|AAGE02030520.1|:87309-87597
Pangu_AAeg	gb|AAGE02030002.1|:1081-1369
Pangu_AAeg	gb|AAGE02029808.1|:21095-21383
Pangu_AAeg	gb|AAGE02028215.1|:1312-1600
Pangu_AAeg	gb|AAGE02021915.1|:29291-29579
Pangu_AAeg	gb|AAGE02017552.1|:81690-81978
Pangu_AAeg	gb|AAGE02014508.1|:5128-5416
Pangu_AAeg	gb|AAGE02014033.1|:30203-30490
Pangu_AAeg	gb|AAGE02013686.1|:74557-74845
Pangu_AAeg	gb|AAGE02013575.1|:278340-278627
Pangu_AAeg	gb|AAGE02010065.1|:20844-21132
Pangu_AAeg	gb|AAGE02009318.1|:74162-74449
Pangu_AAeg	gb|AAGE02009023.1|:264692-264979
Pangu_AAeg	gb|AAGE02008491.1|:123796-124084

Pangu_AAeg	gb|AAGE02007356.1|:159107-159395
Pangu_AAeg	gb|AAGE02005979.1|:18590-18882
Pangu_AAeg	gb|AAGE02003900.1|:259475-259763
Pangu_AAeg	gb|AAGE02003503.1|:135844-136132
Pangu_AAeg	gb|AAGE02002785.1|:631-919
Pangu_AAeg	gb|AAGE02002748.1|:414999-415287
Pangu_AAeg	gb|AAGE02001765.1|:83127-83415
Pangu_AAeg	gb|AAGE02000768.1|:24657-24944
Pangu_AAeg	gb|AAGE02027420.1|:14567-14854
Pangu_AAeg	gb|AAGE02019372.1|:106889-107176
Pangu_AAeg	gb|AAGE02010446.1|:31134-31426
Pangu_AAeg	gb|AAGE02010338.1|:93399-93686
Pangu_AAeg	gb|AAGE02003151.1|:23386-23673
Pangu_AAeg	gb|AAGE02002963.1|:35388-35671
Pangu_AAeg	gb|AAGE02015896.1|:211430-211718
Pangu_AAeg	gb|AAGE02024042.1|:88162-88449
Pangu_AAeg	gb|AAGE02009481.1|:25633-25920
Pangu_AAeg	gb|AAGE02004665.1|:33093-33380
Pangu_AAeg	gb|AAGE02019725.1|:165401-165687
Pangu_AAeg	gb|AAGE02015611.1|:64383-64667
Pangu_AAeg	gb|AAGE02012075.1|:120194-120481
Pangu_AAeg	gb|AAGE02010960.1|:8009-8296
Pangu_AAeg	gb|AAGE02005049.1|:26708-26995
Pangu_AAeg	gb|AAGE02001230.1|:3878-4165
Pangu_AAeg	gb|AAGE02021610.1|:171194-171482
Pangu_AAeg	gb|AAGE02021610.1|:331399-331685
Pangu_AAeg	gb|AAGE02029992.1|:85290-85580
Pangu_AAeg	gb|AAGE02006790.1|:123959-124246
Pangu_AAeg	gb|AAGE02006261.1|:711-997
Pangu_AAeg	gb|AAGE02033251.1|:7262-7549
Pangu_AAeg	gb|AAGE02030993.1|:71-358
Pangu_AAeg	gb|AAGE02027880.1|:13261-13548
Pangu_AAeg	gb|AAGE02019338.1|:97869-98156
Pangu_AAeg	gb|AAGE02015829.1|:1721-2008
Pangu_AAeg	gb|AAGE02011379.1|:58121-58408
Pangu_AAeg	gb|AAGE02007101.1|:280482-280769
Pangu_AAeg	gb|AAGE02006411.1|:13403-13689
Pangu_AAeg	gb|AAGE02003458.1|:65358-65644
Pangu_AAeg	gb|AAGE02001543.1|:37954-38241
Pangu_AAeg	gb|AAGE02000676.1|:76947-77234
Pangu_AAeg	gb|AAGE02006151.1|:178858-179142
Pangu_AAeg	gb|AAGE02009111.1|:25100-25385
Pangu_AAeg	gb|AAGE02007822.1|:201019-201306
Pangu_AAeg	gb|AAGE02005979.1|:71537-71824

Pangu_AAeg	gb|AAGE02001151.1|:48277-48564
Pangu_AAeg	gb|AAGE02035607.1|:1793-2080
Pangu_AAeg	gb|AAGE02028592.1|:35441-35728
Pangu_AAeg	gb|AAGE02021163.1|:9033-9320
Pangu_AAeg	gb|AAGE02018714.1|:10721-11008
Pangu_AAeg	gb|AAGE02018276.1|:84906-85193
Pangu_AAeg	gb|AAGE02016623.1|:210332-210618
Pangu_AAeg	gb|AAGE02016305.1|:7941-8224
Pangu_AAeg	gb|AAGE02013478.1|:6678-6965
Pangu_AAeg	gb|AAGE02011524.1|:78864-79151
Pangu_AAeg	gb|AAGE02011048.1|:5627-5913
Pangu_AAeg	gb|AAGE02010446.1|:46509-46796
Pangu_AAeg	gb|AAGE02010338.1|:216418-216704
Pangu_AAeg	gb|AAGE02008223.1|:12239-12526
Pangu_AAeg	gb|AAGE02007643.1|:57348-57635
Pangu_AAeg	gb|AAGE02007638.1|:19217-19503
Pangu_AAeg	gb|AAGE02007105.1|:63154-63440
Pangu_AAeg	gb|AAGE02006492.1|:22065-22352
Pangu_AAeg	gb|AAGE02003151.1|:186360-186646
Pangu_AAeg	gb|AAGE02001849.1|:30199-30486
Pangu_AAeg	gb|AAGE02001272.1|:3057-3343
Pangu_AAeg	gb|AAGE02000601.1|:60544-60831
Pangu_AAeg	gb|AAGE02000381.1|:106001-106288
Pangu_AAeg	gb|AAGE02021954.1|:46188-46467
Pangu_AAeg	gb|AAGE02015896.1|:280878-281154
Pangu_AAeg	gb|AAGE02013748.1|:29539-29825
Pangu_AAeg	gb|AAGE02001795.1|:442552-442826
Pangu_AAeg	gb|AAGE02016692.1|:29804-30090
Pangu_AAeg	gb|AAGE02009793.1|:36727-37018
Pangu_AAeg	gb|AAGE02004341.1|:86317-86596
Pangu_AAeg	gb|AAGE02021690.1|:11158-11444
Pangu_AAeg	gb|AAGE02016816.1|:44035-44321
Pangu_AAeg	gb|AAGE02000421.1|:2300-2586
Pangu_AAeg	gb|AAGE02008899.1|:230530-230816
Pangu_AAeg	gb|AAGE02006411.1|:48806-49090
Pangu_AAeg	gb|AAGE02021505.1|:1134-1420
Pangu_AAeg	gb|AAGE02018407.1|:47521-47807
Pangu_AAeg	gb|AAGE02015204.1|:20163-20449
Pangu_AAeg	gb|AAGE02000867.1|:307058-307342
Pangu_AAeg	gb|AAGE02010026.1|:26694-26975
Pangu_AAeg	gb|AAGE02009008.1|:131435-131722
Pangu_AAeg	gb|AAGE02002785.1|:16292-16578
Pangu_AAeg	gb|AAGE02024623.1|:3320-3606
Pangu_AAeg	gb|AAGE02019155.1|:3072-3358

Pangu_AAeg	gb|AAGE02006602.1|:18588-18873
Pangu_AAeg	gb|AAGE02016642.1|:112651-112935
Pangu_AAeg	gb|AAGE02010417.1|:230860-231143
Pangu_AAeg	gb|AAGE02007208.1|:93949-94234
Pangu_AAeg	gb|AAGE02005254.1|:203247-203532
Pangu_AAeg	gb|AAGE02006854.1|:177739-178023
Pangu_AAeg	gb|AAGE02020810.1|:68970-69255
Pangu_AAeg	gb|AAGE02010825.1|:32519-32804
Pangu_AAeg	gb|AAGE02007714.1|:16482-16759
Pangu_AAeg	gb|AAGE02000372.1|:16186-16471
Pangu_AAeg	gb|AAGE02007822.1|:286450-286733
Pangu_AAeg	gb|AAGE02001765.1|:173787-174073
Pangu_AAeg	gb|AAGE02010446.1|:130653-130937
Pangu_AAeg	gb|AAGE02003151.1|:100448-101309
Pangu_AAeg	gb|AAGE02019155.1|:277604-277893
Pangu_AAeg	gb|AAGE02011469.1|:173033-173321
Pangu_AAeg	gb|AAGE02021610.1|:251285-251566
Pangu_AAeg	gb|AAGE02029641.1|:3824-4108
Pangu_AAeg	gb|AAGE02018051.1|:63994-64277
Pangu_AAeg	gb|AAGE02010183.1|:19429-19713
Pangu_AAeg	gb|AAGE02008243.1|:11573-11856
Pangu_AAeg	gb|AAGE02015829.1|:37242-37525
Pangu_AAeg	gb|AAGE02000676.1|:58122-59342
Pangu_AAeg	gb|AAGE02011344.1|:81763-82047
Pangu_AAeg	gb|AAGE02033647.1|:11919-12201
Pangu_AAeg	gb|AAGE02022216.1|:107604-107888
Pangu_AAeg	gb|AAGE02011019.1|:18546-20146
Pangu_AAeg	gb|AAGE02008148.1|:12609-12893
Pangu_AAeg	gb|AAGE02006727.1|:3528-3812
Pangu_AAeg	gb|AAGE02006135.1|:6752-7036
Pangu_AAeg	gb|AAGE02006135.1|:76013-76297
Pangu_AAeg	gb|AAGE02006135.1|:291379-295282
Pangu_AAeg	gb|AAGE02003028.1|:76802-77084
Pangu_AAeg	gb|AAGE02021954.1|:6639-6921
Pangu_AAeg	gb|AAGE02010232.1|:4576-4859
Pangu_AAeg	gb|AAGE02004627.1|:25853-26252
Pangu_AAeg	gb|AAGE02003317.1|:101371-101654
Pangu_AAeg	gb|AAGE02011736.1|:376668-376949
Pangu_AAeg	gb|AAGE02016124.1|:83733-84017
Pangu_AAeg	gb|AAGE02011379.1|:84920-85204
Pangu_AAeg	gb|AAGE02000588.1|:166111-166393
Pangu_AAeg	gb|AAGE02006854.1|:44653-44935
Pangu_AAeg	gb|AAGE02008490.1|:92757-93039
Pangu_AAeg	gb|AAGE02030520.1|:64414-64695

Pangu_AAeg	gb|AAGE02000601.1|:152375-152657
Pangu_AAeg	gb|AAGE02015896.1|:61816-62100
Pangu_AAeg	gb|AAGE02007855.1|:85781-86063
Pangu_AAeg	gb|AAGE02011736.1|:227900-228182
Pangu_AAeg	gb|AAGE02005254.1|:27837-28125
Pangu_AAeg	gb|AAGE02002748.1|:217332-217614
Pangu_AAeg	gb|AAGE02001272.1|:89742-90013
Pangu_AAeg	gb|AAGE02020335.1|:10680-10962
Pangu_AAeg	gb|AAGE02010922.1|:105087-105367
Pangu_AAeg	gb|AAGE02002711.1|:174790-175572
Pangu_AAeg	gb|AAGE02011736.1|:425484-425765
Pangu_AAeg	gb|AAGE02008899.1|:133583-133864
Pangu_AAeg	gb|AAGE02000676.1|:283217-283483
Pangu_AAeg	gb|AAGE02004250.1|:245232-245508
Pangu_AAeg	gb|AAGE02015907.1|:31955-33163
Pangu_AAeg	gb|AAGE02003709.1|:55595-55872
Pangu_AAeg	gb|AAGE02016623.1|:105426-105709
Pangu_AAeg	gb|AAGE02001795.1|:75960-77424
Pangu_AAeg	gb|AAGE02021610.1|:230285-230562
Pangu_AAeg	gb|AAGE02010417.1|:95189-95468
Pangu_AAeg	gb|AAGE02018509.1|:267083-267362
Pangu_AAeg	gb|AAGE02003028.1|:138641-138921
Pangu_AAeg	gb|AAGE02003028.1|:225082-225365
Pangu_AAeg	gb|AAGE02016623.1|:21195-21472
Pangu_AAeg	gb|AAGE02010417.1|:46567-46846
Pangu_AAeg	gb|AAGE02028549.1|:3070-3348
Pangu_AAeg	gb|AAGE02011469.1|:119518-119794
Pangu_AAeg	gb|AAGE02000588.1|:88834-89112
Pangu_AAeg	gb|AAGE02009023.1|:77671-77943
Pangu_AAeg	gb|AAGE02018276.1|:96015-97387
Pangu_AAeg	gb|AAGE02000381.1|:34845-35122
Pangu_AAeg	gb|AAGE02021610.1|:418650-418922
Pangu_AAeg	gb|AAGE02006338.1|:54542-54819
Pangu_AAeg	gb|AAGE02016464.1|:20071-20346
Pangu_AAeg	gb|AAGE02009008.1|:223130-223405
Pangu_AAeg	gb|AAGE02016124.1|:14921-15199
Pangu_AAeg	gb|AAGE02013686.1|:117285-117551
Pangu_AAeg	gb|AAGE02009023.1|:89756-90029
Pangu_AAeg	gb|AAGE02007105.1|:22489-22760
Pangu_AAeg	gb|AAGE02015611.1|:10675-10969
Pangu_AAeg	gb|AAGE02000676.1|:204815-205090
Pangu_AAeg	gb|AAGE02000896.1|:98661-98930
Pangu_AAeg	gb|AAGE02016283.1|:128870-129815
Pangu_AAeg	gb|AAGE02011469.1|:95297-103235

Pangu_AAeg	gb|AAGE02003317.1|:3146-4796
Pangu_AAeg	gb|AAGE02019725.1|:66394-66668
Pangu_AAeg	gb|AAGE02018429.1|:92949-93221
Pangu_AAeg	gb|AAGE02008243.1|:181230-181503
Pangu_AAeg	gb|AAGE02000588.1|:63436-63948
Pangu_AAeg	gb|AAGE02000601.1|:394984-395253
Pangu_AAeg	gb|AAGE02015829.1|:63831-64103
Pangu_AAeg	gb|AAGE02005254.1|:324990-325261
Pangu_AAeg	gb|AAGE02000676.1|:41455-41722
Pangu_AAeg	gb|AAGE02010442.1|:156120-156559
Pangu_AAeg	gb|AAGE02009008.1|:362088-362358
Pangu_AAeg	gb|AAGE02001795.1|:265349-265611
Pangu_AAeg	gb|AAGE02007208.1|:37841-38681
Pangu_AAeg	gb|AAGE02004498.1|:52000-52271
Pangu_AAeg	gb|AAGE02007356.1|:309978-311111
Pangu_AAeg	gb|AAGE02003503.1|:109660-110313
Pangu_AAeg	gb|AAGE02021610.1|:291419-291688
Pangu_AAeg	gb|AAGE02011736.1|:120969-121236
Pangu_AAeg	gb|AAGE02005254.1|:347752-348019
Pangu_AAeg	gb|AAGE02011344.1|:5429-5686
Pangu_AAeg	gb|AAGE02000867.1|:231980-232246
Pangu_AAeg	gb|AAGE02014033.1|:17331-17600
Pangu_AAeg	gb|AAGE02020249.1|:131599-131864
Pangu_AAeg	gb|AAGE02006151.1|:442575-442841
Pangu_AAeg	gb|AAGE02009008.1|:16269-16534
Pangu_AAeg	gb|AAGE02005979.1|:97374-97640
Pangu_AAeg	gb|AAGE02013686.1|:25180-25443
Pangu_AAeg	gb|AAGE02007643.1|:37339-37604
Pangu_AAeg	gb|AAGE02016642.1|:259636-259894
Pangu_AAeg	gb|AAGE02010983.1|:94260-94524
Pangu_AAeg	gb|AAGE02001795.1|:211592-211845
Pangu_AAeg	gb|AAGE02024042.1|:202137-202397
Pangu_AAeg	gb|AAGE02006135.1|:204644-204905
Pangu_AAeg	gb|AAGE02024623.1|:58674-58936
Pangu_AAeg	gb|AAGE02019155.1|:169088-169356
Pangu_AAeg	gb|AAGE02006854.1|:338951-346869
Pangu_AAeg	gb|AAGE02006135.1|:321115-321497
Pangu_AAeg	gb|AAGE02003458.1|:117799-118071
Pangu_AAeg	gb|AAGE02013686.1|:98033-98297
Pangu_AAeg	gb|AAGE02007093.1|:305116-305375
Pangu_AAeg	gb|AAGE02007093.1|:349441-349696
Pangu_AAeg	gb|AAGE02026416.1|:113001-113252
Pangu_AAeg	gb|AAGE02014973.1|:16974-17237
Pangu_AAeg	gb|AAGE02008223.1|:47315-47574

Pangu_AAeg	gb|AAGE02015896.1|:165932-166206
Pangu_AAeg	gb|AAGE02021610.1|:61694-61951
Pangu_AAeg	gb|AAGE02009023.1|:46616-46874
Pangu_AAeg	gb|AAGE02004627.1|:68280-68536
Pangu_AAeg	gb|AAGE02000867.1|:203695-203948
Pangu_AAeg	gb|AAGE02010338.1|:80882-81139
Pangu_AAeg	gb|AAGE02007714.1|:80354-80618
Pangu_AAeg	gb|AAGE02019988.1|:29558-29812
Pangu_AAeg	gb|AAGE02000147.1|:85896-86163
Pangu_AAeg	gb|AAGE02007356.1|:33785-34035
Pangu_AAeg	gb|AAGE02000556.1|:18676-18936
Pangu_AAeg	gb|AAGE02010338.1|:459706-459959
Pangu_AAeg	gb|AAGE02002711.1|:9363-9615
Pangu_AAeg	gb|AAGE02016464.1|:70187-70443
Pangu_AAeg	gb|AAGE02011524.1|:726-8329
Pangu_AAeg	gb|AAGE02011736.1|:200145-200398
Pangu_AAeg	gb|AAGE02005979.1|:170793-171049
Pangu_AAeg	gb|AAGE02000147.1|:1327-1569
Pangu_AAeg	gb|AAGE02019725.1|:84893-85149
Pangu_AAeg	gb|AAGE02001795.1|:501974-502244
Pangu_AAeg	gb|AAGE02007105.1|:179789-180037
Pangu_AAeg	gb|AAGE02019338.1|:130469-130716
Pangu_AAeg	gb|AAGE02016623.1|:85969-86217
Pangu_AAeg	gb|AAGE02013793.1|:88315-88557
Pangu_AAeg	gb|AAGE02002711.1|:194942-195186
Pangu_AAeg	gb|AAGE02006790.1|:73700-73992
Pangu_AAeg	gb|AAGE02007714.1|:57276-57527
Pangu_AAeg	gb|AAGE02021954.1|:35064-35301
Pangu_AAeg	gb|AAGE02016816.1|:28401-28645
Pangu_AAeg	gb|AAGE02011379.1|:24144-24386
Pangu_AAeg	gb|AAGE02001849.1|:53515-59940
Pangu_AAeg	gb|AAGE02016393.1|:139880-140119
Pangu_AAeg	gb|AAGE02007101.1|:252785-253041
Pangu_AAeg	gb|AAGE02021505.1|:50849-51083
Pangu_AAeg	gb|AAGE02007093.1|:8821-9075
Pangu_AAeg	gb|AAGE02003503.1|:292694-292935
Pangu_AAeg	gb|AAGE02000381.1|:9798-10058
Pangu_AAeg	gb|AAGE02003900.1|:140104-140340
Pangu_AAeg	gb|AAGE02011469.1|:236798-237034
Pangu_AAeg	gb|AAGE02010922.1|:132073-132304
Pangu_AAeg	gb|AAGE02010468.1|:17558-17793
Pangu_AAeg	gb|AAGE02004148.1|:58511-58747
Pangu_AAeg	gb|AAGE02005254.1|:172203-172431
Pangu_AAeg	gb|AAGE02015098.1|:29252-29485

Pangu_AAeg	gb|AAGE02010442.1|:103830-104066
Pangu_AAeg	gb|AAGE02016393.1|:11262-11491
Pangu_AAeg	gb|AAGE02003503.1|:152407-152636
Pangu_AAeg	gb|AAGE02006471.1|:6197-6424
Pangu_AAeg	gb|AAGE02000588.1|:152149-152379
Pangu_AAeg	gb|AAGE02001272.1|:48457-53559
Pangu_AAeg	gb|AAGE02002711.1|:33329-33555
Pangu_AAeg	gb|AAGE02008168.1|:11045-11266
Pangu_AAeg	gb|AAGE02009008.1|:277933-278595
Pangu_AAeg	gb|AAGE02021753.1|:6444-7854
Pangu_AAeg	gb|AAGE02003151.1|:210544-210802
Pangu_AAeg	gb|AAGE02001795.1|:413253-413473
Pangu_AAeg	gb|AAGE02010064.1|:131138-131389
Pangu_AAeg	gb|AAGE02023178.1|:1926-2146
Pangu_AAeg	gb|AAGE02001765.1|:48388-48608
Pangu_AAeg	gb|AAGE02006601.1|:2150-2368
Pangu_AAeg	gb|AAGE02015896.1|:15627-15854
Pangu_AAeg	gb|AAGE02005802.1|:32445-32664
Pangu_AAeg	gb|AAGE02001272.1|:23799-24016
Pangu_AAeg	gb|AAGE02002748.1|:16255-16474
Pangu_AAeg	gb|AAGE02019719.1|:11870-12087
Pangu_AAeg	gb|AAGE02002368.1|:31067-31283
Pangu_AAeg	gb|AAGE02016393.1|:46448-46665
Pangu_AAeg	gb|AAGE02010442.1|:172742-172953
Pangu_AAeg	gb|AAGE02004148.1|:42067-42280
Pangu_AAeg	gb|AAGE02005802.1|:20312-20528
Pangu_AAeg	gb|AAGE02021610.1|:147836-148051
Pangu_AAeg	gb|AAGE02006135.1|:180439-180654
Pangu_AAeg	gb|AAGE02007714.1|:108323-108536
Pangu_AAeg	gb|AAGE02008168.1|:113124-113337
Pangu_AAeg	gb|AAGE02006151.1|:255749-263602
Pangu_AAeg	gb|AAGE02014591.1|:76989-77192
Pangu_AAeg	gb|AAGE02020249.1|:67318-67530
Pangu_AAeg	gb|AAGE02006941.1|:21079-21287
Pangu_AAeg	gb|AAGE02016816.1|:14249-14457
Pangu_AAeg	gb|AAGE02011524.1|:121837-122045
Pangu_AAeg	gb|AAGE02016216.1|:24132-24336
Pangu_AAeg	gb|AAGE02011469.1|:64561-64768
Pangu_AAeg	gb|AAGE02000147.1|:101303-101507
Pangu_AAeg	gb|AAGE02021610.1|:74421-74625
Pangu_AAeg	gb|AAGE02020334.1|:49291-49496
Pangu_AAeg	gb|AAGE02005388.1|:117889-118089
Pangu_AAeg	gb|AAGE02000676.1|:220895-221099
Pangu_AAeg	gb|AAGE02002303.1|:18815-19019

Pangu_AAeg	gb|AAGE02013575.1|:8979-9183
Pangu_AAeg	gb|AAGE02011524.1|:50308-50514
Pangu_AAeg	gb|AAGE02010338.1|:122959-123163
Pangu_AAeg	gb|AAGE02024623.1|:40753-40956
Pangu_AAeg	gb|AAGE02021311.1|:132834-133037
Pangu_AAeg	gb|AAGE02027964.1|:84992-85196
Pangu_AAeg	gb|AAGE02000676.1|:168553-168756
Pangu_AAeg	gb|AAGE02011344.1|:19026-19229
Pangu_AAeg	gb|AAGE02006151.1|:141398-141601
Pangu_AAeg	gb|AAGE02009023.1|:244681-244884
Pangu_AAeg	gb|AAGE02011524.1|:109734-109937
Pangu_AAeg	gb|AAGE02004627.1|:3486-3729
Pangu_AAeg	gb|AAGE02010983.1|:220348-220549
Pangu_AAeg	gb|AAGE02007093.1|:256846-257050
Pangu_AAeg	gb|AAGE02006151.1|:210456-210661
Pangu_AAeg	gb|AAGE02013686.1|:139009-139213
Pangu_AAeg	gb|AAGE02011524.1|:66983-67187
Pangu_AAeg	gb|AAGE02015098.1|:16443-16645
Pangu_AAeg	gb|AAGE02002711.1|:53571-53772
Pangu_AAeg	gb|AAGE02020516.1|:106872-107073
Pangu_AAeg	gb|AAGE02021610.1|:94685-94887
Pangu_AAeg	gb|AAGE02021425.1|:1873-2074
Pangu_AAeg	gb|AAGE02006338.1|:840-1039
Pangu_AAeg	gb|AAGE02016464.1|:245101-245301
Pangu_AAeg	gb|AAGE02010183.1|:60737-60940
Pangu_AAeg	gb|AAGE02008243.1|:393245-393446
Pangu_AAeg	gb|AAGE02006471.1|:141500-141700
Pangu_AAeg	gb|AAGE02007101.1|:81990-82189
Pangu_AAeg	gb|AAGE02006854.1|:306656-306857
Pangu_AAeg	gb|AAGE02007093.1|:174671-174872
Pangu_AAeg	gb|AAGE02021710.1|:22628-22829
Pangu_AAeg	gb|AAGE02013793.1|:4997-5198
Pangu_AAeg	gb|AAGE02013575.1|:168730-168930
Pangu_AAeg	gb|AAGE02009318.1|:22176-22378
Pangu_AAeg	gb|AAGE02008491.1|:79653-79854
Pangu_AAeg	gb|AAGE02007822.1|:138151-138352
Pangu_AAeg	gb|AAGE02002748.1|:81663-81864
Pangu_AAeg	gb|AAGE02002748.1|:325160-325362
Pangu_AAeg	gb|AAGE02000896.1|:55470-55671
Pangu_AAeg	gb|AAGE02016576.1|:98141-98344
Pangu_AAeg	gb|AAGE02010338.1|:405414-405621
Pangu_AAeg	gb|AAGE02001272.1|:34239-34440
Pangu_AAeg	gb|AAGE02000601.1|:136758-136959
Pangu_AAeg	gb|AAGE02017113.1|:11232-11433

Pangu_AAeg	gb|AAGE02003458.1|:92726-93659
Pangu_AAeg	gb|AAGE02034720.1|:5381-5583
Pangu_AAeg	gb|AAGE02010338.1|:49095-49294
Pangu_AAeg	gb|AAGE02015098.1|:106062-106260
Pangu_AAeg	gb|AAGE02010922.1|:91384-92399
Pangu_AAeg	gb|AAGE02021311.1|:114464-114663
Pangu_AAeg	gb|AAGE02004250.1|:150255-150453
Pangu_AAeg	gb|AAGE02007643.1|:12003-12223
Pangu_AAeg	gb|AAGE02019155.1|:261999-262197
Pangu_AAeg	gb|AAGE02025641.1|:6067-6262
Pangu_AAeg	gb|AAGE02009008.1|:345883-346081
Pangu_AAeg	gb|AAGE02019372.1|:61956-62145
Pangu_AAeg	gb|AAGE02018391.1|:66362-66558
Pangu_AAeg	gb|AAGE02018051.1|:496-691
Pangu_AAeg	gb|AAGE02013575.1|:369694-369888
Pangu_AAeg	gb|AAGE02010442.1|:214818-215004
Pangu_AAeg	gb|AAGE02009111.1|:99385-99579
Pangu_AAeg	gb|AAGE02001795.1|:388916-389106
Pangu_AAeg	gb|AAGE02006159.1|:156544-156739
Pangu_AAeg	gb|AAGE02006135.1|:249524-249715
Pangu_AAeg	gb|AAGE02013575.1|:90189-90379
Pangu_AAeg	gb|AAGE02011736.1|:466459-466652
Pangu_AAeg	gb|AAGE02000768.1|:66811-67003
Pangu_AAeg	gb|AAGE02017809.1|:39296-39492
Pangu_AAeg	gb|AAGE02004341.1|:150876-151128
Pangu_AAeg	gb|AAGE02016464.1|:174097-174287
Pangu_AAeg	gb|AAGE02016464.1|:233016-233204
Pangu_AAeg	gb|AAGE02011469.1|:24507-24694
Pangu_AAeg	gb|AAGE02000676.1|:26022-26208
Pangu_AAeg	gb|AAGE02018429.1|:116731-116916
Pangu_AAeg	gb|AAGE02010468.1|:61001-61187
Pangu_AAeg	gb|AAGE02002121.1|:101903-102090
Pangu_AAeg	gb|AAGE02010232.1|:39932-40117
Pangu_AAeg	gb|AAGE02003317.1|:41530-41715
Pangu_AAeg	gb|AAGE02003317.1|:84163-84348
Pangu_AAeg	gb|AAGE02002368.1|:104945-105130
Pangu_AAeg	gb|AAGE02012075.1|:101435-101620
Pangu_AAeg	gb|AAGE02010960.1|:24853-25038
Pangu_AAeg	gb|AAGE02001230.1|:89190-89376
Pangu_AAeg	gb|AAGE02021610.1|:390226-390411
Pangu_AAeg	gb|AAGE02021425.1|:113229-113417
Pangu_AAeg	gb|AAGE02011736.1|:140751-140936
Pangu_AAeg	gb|AAGE02010417.1|:156821-157003
Pangu_AAeg	gb|AAGE02010983.1|:55698-55882

Pangu_AAeg	gb|AAGE02008899.1|:160564-160749
Pangu_AAeg	gb|AAGE02008899.1|:196369-196554
Pangu_AAeg	gb|AAGE02006790.1|:58456-58641
Pangu_AAeg	gb|AAGE02017113.1|:33006-33192
Pangu_AAeg	gb|AAGE02016464.1|:7059-7244
Pangu_AAeg	gb|AAGE02011723.1|:1550-1735
Pangu_AAeg	gb|AAGE02010183.1|:49642-49825
Pangu_AAeg	gb|AAGE02008243.1|:218961-219145
Pangu_AAeg	gb|AAGE02008243.1|:357581-357766
Pangu_AAeg	gb|AAGE02008243.1|:381508-381693
Pangu_AAeg	gb|AAGE02006261.1|:155858-156043
Pangu_AAeg	gb|AAGE02018509.1|:213646-213831
Pangu_AAeg	gb|AAGE02015829.1|:116124-116309
Pangu_AAeg	gb|AAGE02007101.1|:193056-193241
Pangu_AAeg	gb|AAGE02001543.1|:26171-26358
Pangu_AAeg	gb|AAGE02000676.1|:309889-310074
Pangu_AAeg	gb|AAGE02000676.1|:331084-331269
Pangu_AAeg	gb|AAGE02018407.1|:92120-92305
Pangu_AAeg	gb|AAGE02011344.1|:30486-30671
Pangu_AAeg	gb|AAGE02006854.1|:146927-147112
Pangu_AAeg	gb|AAGE02004250.1|:389087-389263
Pangu_AAeg	gb|AAGE02000867.1|:93881-94066
Pangu_AAeg	gb|AAGE02000278.1|:29623-29808
Pangu_AAeg	gb|AAGE02020249.1|:17129-17315
Pangu_AAeg	gb|AAGE02020249.1|:83025-83210
Pangu_AAeg	gb|AAGE02007814.1|:5284-5469
Pangu_AAeg	gb|AAGE02007093.1|:192184-192369
Pangu_AAeg	gb|AAGE02006151.1|:12533-12718
Pangu_AAeg	gb|AAGE02026416.1|:6573-6758
Pangu_AAeg	gb|AAGE02025641.1|:28122-28307
Pangu_AAeg	gb|AAGE02022216.1|:91786-91971
Pangu_AAeg	gb|AAGE02017552.1|:51331-51517
Pangu_AAeg	gb|AAGE02013575.1|:187001-187180
Pangu_AAeg	gb|AAGE02009008.1|:70569-70754
Pangu_AAeg	gb|AAGE02009008.1|:330177-330363
Pangu_AAeg	gb|AAGE02007822.1|:10668-10853
Pangu_AAeg	gb|AAGE02007822.1|:122299-122484
Pangu_AAeg	gb|AAGE02007822.1|:161417-161602
Pangu_AAeg	gb|AAGE02007488.1|:50687-50872
Pangu_AAeg	gb|AAGE02005979.1|:135196-135378
Pangu_AAeg	gb|AAGE02003900.1|:56401-57003
Pangu_AAeg	gb|AAGE02002748.1|:69310-69495
Pangu_AAeg	gb|AAGE02002748.1|:281758-281943
Pangu_AAeg	gb|AAGE02002748.1|:397125-397310

Pangu_AAeg	gb|AAGE02000896.1|:22996-23181
Pangu_AAeg	gb|AAGE02000896.1|:144321-144506
Pangu_AAeg	gb|AAGE02016623.1|:136378-136564
Pangu_AAeg	gb|AAGE02011048.1|:58789-58974
Pangu_AAeg	gb|AAGE02010338.1|:18944-19129
Pangu_AAeg	gb|AAGE02005486.1|:1433-1617
Pangu_AAeg	gb|AAGE02002831.1|:12891-13076
Pangu_AAeg	gb|AAGE02019155.1|:75134-75319
Pangu_AAeg	gb|AAGE02015896.1|:35693-35878
Pangu_AAeg	gb|AAGE02010922.1|:146887-147073
Pangu_AAeg	gb|AAGE02004341.1|:27584-27768
Pangu_AAeg	gb|AAGE02013738.1|:17101-17285
Pangu_AAeg	gb|AAGE02010417.1|:12095-12280
Pangu_AAeg	gb|AAGE02006790.1|:160214-160398
Pangu_AAeg	gb|AAGE02011723.1|:26713-26897
Pangu_AAeg	gb|AAGE02005388.1|:194633-194817
Pangu_AAeg	gb|AAGE02018509.1|:231627-231811
Pangu_AAeg	gb|AAGE02018407.1|:72330-72514
Pangu_AAeg	gb|AAGE02006854.1|:324351-324535
Pangu_AAeg	gb|AAGE02025641.1|:48641-48825
Pangu_AAeg	gb|AAGE02021915.1|:84697-84882
Pangu_AAeg	gb|AAGE02008491.1|:265095-265280
Pangu_AAeg	gb|AAGE02007488.1|:65806-65991
Pangu_AAeg	gb|AAGE02006135.1|:38493-38676
Pangu_AAeg	gb|AAGE02006135.1|:228331-228516
Pangu_AAeg	gb|AAGE02001765.1|:22751-22934
Pangu_AAeg	gb|AAGE02000896.1|:66457-66641
Pangu_AAeg	gb|AAGE02019372.1|:47001-47182
Pangu_AAeg	gb|AAGE02016623.1|:179663-179849
Pangu_AAeg	gb|AAGE02003151.1|:56135-56320
Pangu_AAeg	gb|AAGE02006854.1|:215990-216174
Pangu_AAeg	gb|AAGE02026416.1|:62045-62230
Pangu_AAeg	gb|AAGE02009318.1|:41164-41348
Pangu_AAeg	gb|AAGE02006135.1|:102919-103103
Pangu_AAeg	gb|AAGE02006159.1|:22337-22519
Pangu_AAeg	gb|AAGE02011723.1|:58932-59113
Pangu_AAeg	gb|AAGE02006471.1|:180178-180361
Pangu_AAeg	gb|AAGE02009008.1|:307121-314517
Pangu_AAeg	gb|AAGE02018891.1|:13834-14016
Pangu_AAeg	gb|AAGE02016216.1|:4969-5149
Pangu_AAeg	gb|AAGE02018051.1|:18104-18286
Pangu_AAeg	gb|AAGE02002748.1|:118616-118796
Pangu_AAeg	gb|AAGE02024623.1|:13761-13943
Pangu_AAeg	gb|AAGE02001795.1|:104544-104723

Pangu_AAeg	gb|AAGE02016393.1|:124778-124957
Pangu_AAeg	gb|AAGE02008243.1|:248019-248198
Pangu_AAeg	gb|AAGE02006261.1|:167548-167727
Pangu_AAeg	gb|AAGE02002303.1|:103886-104063
Pangu_AAeg	gb|AAGE02013793.1|:51287-51466
Pangu_AAeg	gb|AAGE02013755.1|:2-176
Pangu_AAeg	gb|AAGE02009008.1|:112842-113019
Pangu_AAeg	gb|AAGE02001572.1|:137077-137256
Pangu_AAeg	gb|AAGE02005254.1|:252910-253088
Pangu_AAeg	gb|AAGE02009008.1|:27302-27480
Pangu_AAeg	gb|AAGE02002963.1|:138428-138606
Pangu_AAeg	gb|AAGE02008899.1|:84698-84875
Pangu_AAeg	gb|AAGE02011379.1|:145924-146555
Pangu_AAeg	gb|AAGE02000867.1|:274735-274912
Pangu_AAeg	gb|AAGE02006151.1|:200013-200237
Pangu_AAeg	gb|AAGE02006151.1|:340541-340720
Pangu_AAeg	gb|AAGE02009023.1|:15378-23182
Pangu_AAeg	gb|AAGE02009008.1|:375673-375851
Pangu_AAeg	gb|AAGE02016623.1|:58693-58869
Pangu_AAeg	gb|AAGE02010446.1|:5883-6060
Pangu_AAeg	gb|AAGE02016124.1|:126741-126919
Pangu_AAeg	gb|AAGE02006338.1|:75374-75544
Pangu_AAeg	gb|AAGE02019767.1|:49653-49831
Pangu_AAeg	gb|AAGE02006151.1|:473843-474017
Pangu_AAeg	gb|AAGE02009008.1|:57837-58011
Pangu_AAeg	gb|AAGE02018509.1|:161180-161353
Pangu_AAeg	gb|AAGE02006159.1|:52162-52389
Pangu_AAeg	gb|AAGE02002963.1|:123127-123302
Pangu_AAeg	gb|AAGE02005388.1|:84129-84306
Pangu_AAeg	gb|AAGE02008899.1|:38033-38216
Pangu_AAeg	gb|AAGE02004341.1|:13292-13547
Pangu_AAeg	gb|AAGE02007101.1|:178679-178853
Pangu_AAeg	gb|AAGE02010825.1|:3074-3251
Pangu_AAeg	gb|AAGE02008491.1|:252059-252231
Pangu_AAeg	gb|AAGE02010338.1|:283872-284042
Pangu_AAeg	gb|AAGE02003151.1|:84903-85072
Pangu_AAeg	gb|AAGE02007809.1|:21511-22845
Pangu_AAeg	gb|AAGE02019338.1|:44598-44813
Pangu_AAeg	gb|AAGE02000896.1|:189251-189423
Pangu_AAeg	gb|AAGE02020252.1|:4396-4562
Pangu_AAeg	gb|AAGE02027964.1|:56091-62083
Pangu_AAeg	gb|AAGE02021710.1|:3752-3917
Pangu_AAeg	gb|AAGE02013575.1|:112740-112910
Pangu_AAeg	gb|AAGE02010232.1|:90612-90778

Pangu_AAeg	gb|AAGE02001572.1|:39926-40090
Pangu_AAeg	gb|AAGE02000601.1|:352952-353118
Pangu_AAeg	gb|AAGE02000278.1|:52607-52764
Pangu_AAeg	gb|AAGE02007105.1|:143396-143562
Pangu_AAeg	gb|AAGE02006471.1|:166912-167850
Pangu_AAeg	gb|AAGE02005254.1|:117588-117749
Pangu_AAeg	gb|AAGE02017552.1|:113147-113326
Pangu_AAeg	gb|AAGE02021610.1|:17750-17938
Pangu_AAeg	gb|AAGE02000601.1|:310396-310553
Pangu_AAeg	gb|AAGE02015907.1|:215598-215753
Pangu_AAeg	gb|AAGE02003867.1|:94805-94959
Pangu_AAeg	gb|AAGE02008243.1|:83296-91695
Pangu_AAeg	gb|AAGE02003990.1|:4429-11125
Pangu_AAeg	gb|AAGE02022216.1|:43527-43700
Pangu_AAeg	gb|AAGE02016692.1|:52546-52697
Pangu_AAeg	gb|AAGE02027964.1|:4178-4329
Pangu_AAeg	gb|AAGE02009023.1|:65208-65361
Pangu_AAeg	gb|AAGE02006151.1|:387527-387678
Pangu_AAeg	gb|AAGE02006727.1|:24461-24609
Pangu_AAeg	gb|AAGE02010922.1|:62647-62795
Pangu_AAeg	gb|AAGE02011019.1|:53991-54140
Pangu_AAeg	gb|AAGE02000867.1|:249085-249233
Pangu_AAeg	gb|AAGE02000601.1|:41316-41468
Pangu_AAeg	gb|AAGE02017552.1|:41022-41184
Pangu_AAeg	gb|AAGE02004665.1|:4-146
Pangu_AAeg	gb|AAGE02010417.1|:188546-188689
Pangu_AAeg	gb|AAGE02008899.1|:15454-15590
Pangu_AAeg	gb|AAGE02000601.1|:220740-220883
Pangu_AAeg	gb|AAGE02024042.1|:101717-102504
Pangu_AAeg	gb|AAGE02029992.1|:36801-36940
Pangu_AAeg	gb|AAGE02008243.1|:34350-34489
Pangu_AAeg	gb|AAGE02016283.1|:10057-10196
Pangu_AAeg	gb|AAGE02003151.1|:134786-134924
Pangu_AAeg	gb|AAGE02011736.1|:41164-41292
Pangu_AAeg	gb|AAGE02009023.1|:34449-34586
Pangu_AAeg	gb|AAGE02013575.1|:260860-260994
Pangu_AAeg	gb|AAGE02007822.1|:37214-37348
Pangu_AAeg	gb|AAGE02006135.1|:21807-21941
Pangu_AAeg	gb|AAGE02000676.1|:144843-144995
Pangu_AAeg	gb|AAGE02006159.1|:170557-178720
Pangu_AAeg	gb|AAGE02006151.1|:365395-365529
Pangu_AAeg	gb|AAGE02007101.1|:236719-236855
Pangu_AAeg	gb|AAGE02011736.1|:341092-341224
Pangu_AAeg	gb|AAGE02029992.1|:61393-61525

Pangu_AAeg	gb|AAGE02010508.1|:6699-7780
Pangu_AAeg	gb|AAGE02010442.1|:144958-145087
Pangu_AAeg	gb|AAGE02012075.1|:80207-80330
Pangu_AAeg	gb|AAGE02005254.1|:56378-56503
Pangu_AAeg	gb|AAGE02006854.1|:271830-271957
Pangu_AAeg	gb|AAGE02007488.1|:97756-97883
Pangu_AAeg	gb|AAGE02011736.1|:252251-252377
Pangu_AAeg	gb|AAGE02010983.1|:180460-180585
Pangu_AAeg	gb|AAGE02002534.1|:26361-26486
Pangu_AAeg	gb|AAGE02016283.1|:113954-114079
Pangu_AAeg	gb|AAGE02026416.1|:27395-27520
Pangu_AAeg	gb|AAGE02028020.1|:2864-2988
Pangu_AAeg	gb|AAGE02022141.1|:36565-36688
Pangu_AAeg	gb|AAGE02007101.1|:49924-50047
Pangu_AAeg	gb|AAGE02028215.1|:51233-51356
Pangu_AAeg	gb|AAGE02026591.1|:35531-35695
Pangu_AAeg	gb|AAGE02004341.1|:110882-111002
Pangu_AAeg	gb|AAGE02006790.1|:14004-14123
Pangu_AAeg	gb|AAGE02006854.1|:11729-11846
Pangu_AAeg	gb|AAGE02018509.1|:38768-39270
Pangu_AAeg	gb|AAGE02000867.1|:18057-18184
Pangu_AAeg	gb|AAGE02001765.1|:120803-129841
Pangu_AAeg	gb|AAGE02011736.1|:85366-85480
Pangu_AAeg	gb|AAGE02012075.1|:31691-31805
Pangu_AAeg	gb|AAGE02015907.1|:13584-13695
Pangu_AAeg	gb|AAGE02019725.1|:113691-115039
Pangu_AAeg	gb|AAGE02016642.1|:146619-146728
Pangu_AAeg	gb|AAGE02003503.1|:255127-255235
Pangu_AAeg	gb|AAGE02007105.1|:47904-48062
Pangu_AAeg	gb|AAGE02001230.1|:16543-16650
Pangu_AAeg	gb|AAGE02019988.1|:5986-6092
Pangu_AAeg	gb|AAGE02006151.1|:225168-225281
Pangu_AAeg	gb|AAGE02003867.1|:125795-132788
Pangu_AAeg	gb|AAGE02004250.1|:316065-316170
Pangu_AAeg	gb|AAGE02010983.1|:32530-42149
Pangu_AAeg	gb|AAGE02006854.1|:231290-231390
Pangu_AAeg	gb|AAGE02007809.1|:1745-1844
Pangu_AAeg	gb|AAGE02011469.1|:222917-223015
Pangu_AAeg	gb|AAGE02018407.1|:15418-15514
Pangu_AAeg	gb|AAGE02008243.1|:317444-317539
Pangu_AAeg	gb|AAGE02005254.1|:43641-43737
Pangu_AAeg	gb|AAGE02011736.1|:404825-404918
Pangu_AAeg	gb|AAGE02003151.1|:169385-169478
Pangu_AAeg	gb|AAGE02004665.1|:13141-13232

Pangu_AAeg	gb|AAGE02000601.1|:80384-80475
Pangu_AAeg	gb|AAGE02018509.1|:75979-76087
Pangu_AAeg	gb|AAGE02007101.1|:99229-99320
Pangu_AAeg	gb|AAGE02010338.1|:197935-198026
Pangu_AAeg	gb|AAGE02019155.1|:199037-199122
Pangu_AAeg	gb|AAGE02003317.1|:72090-72179
Pangu_AAeg	gb|AAGE02015098.1|:42661-42750
Pangu_AAeg	gb|AAGE02002748.1|:355420-355506
Pangu_AAeg	gb|AAGE02011379.1|:129114-129201
Pangu_AAeg	gb|AAGE02007101.1|:15919-16005
Pangu_AAeg	gb|AAGE02000601.1|:261601-261685
Pangu_AAeg	gb|AAGE02002368.1|:14727-14812
Pangu_AAeg	gb|AAGE02028020.1|:36483-36565
Pangu_AAeg	gb|AAGE02015896.1|:118724-118807
Pangu_AAeg	gb|AAGE02023712.1|:1029-1110
Pangu_AAeg	gb|AAGE02001795.1|:89705-89787
Pangu_AAeg	gb|AAGE02017552.1|:18846-18927
Pangu_AAeg	gb|AAGE02006339.1|:9-88
Pangu_AAeg	gb|AAGE02004250.1|:351990-352070
Pangu_AAeg	gb|AAGE02007356.1|:68723-68802
Pangu_AAeg	gb|AAGE02024783.1|:695-773
Pangu_AAeg	gb|AAGE02004250.1|:87826-87904
Pangu_AAeg	gb|AAGE02007093.1|:62498-62576
Pangu_AAeg	gb|AAGE02013793.1|:25717-25797
Pangu_AAeg	gb|AAGE02018429.1|:140663-140739
Pangu_AAeg	gb|AAGE02015907.1|:196747-196823
Pangu_AAeg	gb|AAGE02011736.1|:64841-64915
Pangu_AAeg	gb|AAGE02000588.1|:18527-18602
Pangu_AAeg	gb|AAGE02006151.1|:554351-554426
Pangu_AAeg	gb|AAGE02018714.1|:58894-58969
Pangu_AAeg	gb|AAGE02008899.1|:171260-171332
Pangu_AAeg	gb|AAGE02009008.1|:291553-291624
Pangu_AAeg	gb|AAGE02010468.1|:45041-45111
Pangu_AAeg	gb|AAGE02001230.1|:50463-50533
Pangu_AAeg	gb|AAGE02011736.1|:354670-354740
Pangu_AAeg	gb|AAGE02020249.1|:97208-97278
Pangu_AAeg	gb|AAGE02016393.1|:35993-36060
Pangu_AAeg	gb|AAGE02006261.1|:86579-86645
Pangu_AAeg	gb|AAGE02003900.1|:183839-183905
Pangu_AAeg	gb|AAGE02011723.1|:69376-69441
Pangu_AAeg	gb|AAGE02010338.1|:476136-476199
Pangu_AAeg	gb|AAGE02008491.1|:100687-100750
Pangu_AAeg	gb|AAGE02025641.1|:38565-38626
Pangu_AAeg	gb|AAGE02010366.1|:13405-13466

Pangu_AAeg	gb|AAGE02009111.1|:6425-6486
Pangu_AAeg	gb|AAGE02016283.1|:74906-74967
Pangu_AAeg	gb|AAGE02011469.1|:46899-46958
Pangu_AAeg	gb|AAGE02021505.1|:37448-37505
Pangu_AAeg	gb|AAGE02016567.1|:16751-16807
Pangu_AAeg	gb|AAGE02006151.1|:404479-404535
Pangu_AAeg	gb|AAGE02019372.1|:5816-5872
Pangu_AAeg	gb|AAGE02003028.1|:24544-24599
Pangu_AAeg	gb|AAGE02006471.1|:126454-126508
Pangu_AAeg	gb|AAGE02007814.1|:24479-24531
Pangu_AAeg	gb|AAGE02011048.1|:38285-38337
Pangu_AAeg	gb|AAGE02010442.1|:202945-202996
Pangu_AAeg	gb|AAGE02013748.1|:16978-21524
Pangu_AAeg	gb|AAGE02008243.1|:301352-304419
Pangu_AAeg	gb|AAGE02033647.1|:2385-3828
Pangu_AAeg	gb|AAGE02006159.1|:175567-177498
Pangu_AAeg	gb|AAGE02009356.1|:7386-15938
Pangu_AAeg	gb|AAGE02004250.1|:314908-318618
Pangu_AAeg	gb|AAGE02009023.1|:178265-182666
Pangu_AAeg	gb|AAGE02019725.1|:255696-263497
Pangu_AAeg	gb|AAGE02015896.1|:80437-80731
Pangu_AAeg	gb|AAGE02005388.1|:103418-103710
Pangu_AAeg	gb|AAGE02017809.1|:60524-60815
Pangu_AAeg	gb|AAGE02001572.1|:125465-125756
Pangu_AAeg	gb|AAGE02002303.1|:38011-38301
Pangu_AAeg	gb|AAGE02016283.1|:76011-76302
Pangu_AAeg	gb|AAGE02027480.1|:5510-5800
Pangu_AAeg	gb|AAGE02012075.1|:169022-169313
Pangu_AAeg	gb|AAGE02013738.1|:24210-24500
Pangu_AAeg	gb|AAGE02016124.1|:131835-132124
Pangu_AAeg	gb|AAGE02010983.1|:28214-28503
Pangu_AAeg	gb|AAGE02007714.1|:13436-13725
Pangu_AAeg	gb|AAGE02007356.1|:51749-52037
Pangu_AAeg	gb|AAGE02018429.1|:18947-19239
Pangu_AAeg	gb|AAGE02001795.1|:140838-141124
Pangu_AAeg	gb|AAGE02007855.1|:57189-57477
Pangu_AAeg	gb|AAGE02002121.1|:70648-70936
Pangu_AAeg	gb|AAGE02002121.1|:39981-40269
Pangu_AAeg	gb|AAGE02011469.1|:165276-165564
Pangu_AAeg	gb|AAGE02010232.1|:43048-43336
Pangu_AAeg	gb|AAGE02010064.1|:178405-178693
Pangu_AAeg	gb|AAGE02008802.1|:3131-3419
Pangu_AAeg	gb|AAGE02004627.1|:46618-46906
Pangu_AAeg	gb|AAGE02003317.1|:206535-206823

Pangu_AAeg	gb|AAGE02001991.1|:35884-36172
Pangu_AAeg	gb|AAGE02015611.1|:75241-75530
Pangu_AAeg	gb|AAGE02030335.1|:5906-6194
Pangu_AAeg	gb|AAGE02028287.1|:39756-40044
Pangu_AAeg	gb|AAGE02016642.1|:49011-49299
Pangu_AAeg	gb|AAGE02010922.1|:90724-91012
Pangu_AAeg	gb|AAGE02008262.1|:14336-14624
Pangu_AAeg	gb|AAGE02004341.1|:76246-76534
Pangu_AAeg	gb|AAGE02003867.1|:94514-94802
Pangu_AAeg	gb|AAGE02000575.1|:182-470
Pangu_AAeg	gb|AAGE02012075.1|:67830-68113
Pangu_AAeg	gb|AAGE02011736.1|:291111-291399
Pangu_AAeg	gb|AAGE02011736.1|:240333-240621
Pangu_AAeg	gb|AAGE02000421.1|:35156-35444
Pangu_AAeg	gb|AAGE02028594.1|:7715-8003
Pangu_AAeg	gb|AAGE02028499.1|:3393-3681
Pangu_AAeg	gb|AAGE02020334.1|:8296-8584
Pangu_AAeg	gb|AAGE02018878.1|:6365-6653
Pangu_AAeg	gb|AAGE02018051.1|:49823-50111
Pangu_AAeg	gb|AAGE02017442.1|:9324-9612
Pangu_AAeg	gb|AAGE02017113.1|:17870-18158
Pangu_AAeg	gb|AAGE02016567.1|:609-897
Pangu_AAeg	gb|AAGE02016464.1|:207410-207698
Pangu_AAeg	gb|AAGE02016393.1|:1375-1663
Pangu_AAeg	gb|AAGE02010366.1|:44975-45263
Pangu_AAeg	gb|AAGE02010183.1|:97789-98077
Pangu_AAeg	gb|AAGE02008243.1|:407421-407706
Pangu_AAeg	gb|AAGE02006471.1|:87654-87940
Pangu_AAeg	gb|AAGE02006471.1|:54321-54603
Pangu_AAeg	gb|AAGE02011928.1|:76192-76480
Pangu_AAeg	gb|AAGE02003458.1|:33978-34266
Pangu_AAeg	gb|AAGE02006854.1|:64180-64469
Pangu_AAeg	gb|AAGE02004250.1|:269744-270031
Pangu_AAeg	gb|AAGE02022141.1|:16212-16499
Pangu_AAeg	gb|AAGE02020249.1|:119672-119959
Pangu_AAeg	gb|AAGE02030354.1|:2237-2525
Pangu_AAeg	gb|AAGE02026591.1|:18814-19102
Pangu_AAeg	gb|AAGE02026416.1|:32776-33064
Pangu_AAeg	gb|AAGE02025641.1|:90238-90526
Pangu_AAeg	gb|AAGE02023178.1|:171-459
Pangu_AAeg	gb|AAGE02021710.1|:31983-32271
Pangu_AAeg	gb|AAGE02021432.1|:58114-58402
Pangu_AAeg	gb|AAGE02019767.1|:29474-29762
Pangu_AAeg	gb|AAGE02019719.1|:8313-8601

Pangu_AAeg	gb|AAGE02014973.1|:18972-19260
Pangu_AAeg	gb|AAGE02014074.1|:7334-7622
Pangu_AAeg	gb|AAGE02013793.1|:95158-95445
Pangu_AAeg	gb|AAGE02013755.1|:1069-1357
Pangu_AAeg	gb|AAGE02011019.1|:8841-9127
Pangu_AAeg	gb|AAGE02010508.1|:11860-12148
Pangu_AAeg	gb|AAGE02010442.1|:37269-37557
Pangu_AAeg	gb|AAGE02010026.1|:30962-31250
Pangu_AAeg	gb|AAGE02010026.1|:9468-9756
Pangu_AAeg	gb|AAGE02009111.1|:106740-107028
Pangu_AAeg	gb|AAGE02009008.1|:5644-5932
Pangu_AAeg	gb|AAGE02008038.1|:7555-7843
Pangu_AAeg	gb|AAGE02007822.1|:212223-212511
Pangu_AAeg	gb|AAGE02007822.1|:17798-18085
Pangu_AAeg	gb|AAGE02007488.1|:58037-58325
Pangu_AAeg	gb|AAGE02007356.1|:281861-282149
Pangu_AAeg	gb|AAGE02006727.1|:13356-13644
Pangu_AAeg	gb|AAGE02006135.1|:32647-32937
Pangu_AAeg	gb|AAGE02003709.1|:40274-40562
Pangu_AAeg	gb|AAGE02003028.1|:122356-122644
Pangu_AAeg	gb|AAGE02002780.1|:11006-11294
Pangu_AAeg	gb|AAGE02001151.1|:15873-16159
Pangu_AAeg	gb|AAGE02000896.1|:2955-3243
Pangu_AAeg	gb|AAGE02000768.1|:7754-8042
Pangu_AAeg	gb|AAGE02000556.1|:8459-8747
Pangu_AAeg	gb|AAGE02018891.1|:52362-52644
Pangu_AAeg	gb|AAGE02016623.1|:163935-164222
Pangu_AAeg	gb|AAGE02019155.1|:118681-118968
Pangu_AAeg	gb|AAGE02019155.1|:103548-103836
Pangu_AAeg	gb|AAGE02001795.1|:98329-98612
Pangu_AAeg	gb|AAGE02016216.1|:16508-16795
Pangu_AAeg	gb|AAGE02003317.1|:170184-170471
Pangu_AAeg	gb|AAGE02024042.1|:77939-78226
Pangu_AAeg	gb|AAGE02020516.1|:41328-41615
Pangu_AAeg	gb|AAGE02014173.1|:422-709
Pangu_AAeg	gb|AAGE02006941.1|:82123-82410
Pangu_AAeg	gb|AAGE02028549.1|:9168-9455
Pangu_AAeg	gb|AAGE02019988.1|:32265-32552
Pangu_AAeg	gb|AAGE02018509.1|:100374-100660
Pangu_AAeg	gb|AAGE02005254.1|:148462-148748
Pangu_AAeg	gb|AAGE02002534.1|:50120-50407
Pangu_AAeg	gb|AAGE02000676.1|:207860-208146
Pangu_AAeg	gb|AAGE02000588.1|:147369-147656
Pangu_AAeg	gb|AAGE02007814.1|:16802-17089

Pangu_AAeg	gb|AAGE02021710.1|:49561-49843
Pangu_AAeg	gb|AAGE02009023.1|:282418-282696
Pangu_AAeg	gb|AAGE02008491.1|:247616-247903
Pangu_AAeg	gb|AAGE02027420.1|:8171-8457
Pangu_AAeg	gb|AAGE02024783.1|:9878-10165
Pangu_AAeg	gb|AAGE02021753.1|:19646-19932
Pangu_AAeg	gb|AAGE02019372.1|:62326-62613
Pangu_AAeg	gb|AAGE02018891.1|:27820-28107
Pangu_AAeg	gb|AAGE02016305.1|:47263-47550
Pangu_AAeg	gb|AAGE02016283.1|:112996-113283
Pangu_AAeg	gb|AAGE02014236.1|:904-1190
Pangu_AAeg	gb|AAGE02011524.1|:102307-102594
Pangu_AAeg	gb|AAGE02008706.1|:3329-3616
Pangu_AAeg	gb|AAGE02007105.1|:53240-53526
Pangu_AAeg	gb|AAGE02003151.1|:250957-251244
Pangu_AAeg	gb|AAGE02002831.1|:7087-7374
Pangu_AAeg	gb|AAGE02001584.1|:47501-47788
Pangu_AAeg	gb|AAGE02014591.1|:41372-41658
Pangu_AAeg	gb|AAGE02015098.1|:150126-150395
Pangu_AAeg	gb|AAGE02010960.1|:14818-15098
Pangu_AAeg	gb|AAGE02028004.1|:16685-16971
Pangu_AAeg	gb|AAGE02021425.1|:60618-60904
Pangu_AAeg	gb|AAGE02020252.1|:76799-77085
Pangu_AAeg	gb|AAGE02010417.1|:253119-253405
Pangu_AAeg	gb|AAGE02007809.1|:14470-14756
Pangu_AAeg	gb|AAGE02013647.1|:78680-78969
Pangu_AAeg	gb|AAGE02025980.1|:1029-1316
Pangu_AAeg	gb|AAGE02000588.1|:60500-60786
Pangu_AAeg	gb|AAGE02006854.1|:150478-150764
Pangu_AAeg	gb|AAGE02004498.1|:30856-31142
Pangu_AAeg	gb|AAGE02007714.1|:104869-105155
Pangu_AAeg	gb|AAGE02006151.1|:274830-275115
Pangu_AAeg	gb|AAGE02026591.1|:111025-111308
Pangu_AAeg	gb|AAGE02006135.1|:243628-243902
Pangu_AAeg	gb|AAGE02003503.1|:193322-193608
Pangu_AAeg	gb|AAGE02003503.1|:160383-160848
Pangu_AAeg	gb|AAGE02003028.1|:274093-274378
Pangu_AAeg	gb|AAGE02000768.1|:47610-47894
Pangu_AAeg	gb|AAGE02001849.1|:51639-51925
Pangu_AAeg	gb|AAGE02035323.1|:5247-5532
Pangu_AAeg	gb|AAGE02031563.1|:1681-1967
Pangu_AAeg	gb|AAGE02023712.1|:6474-6760
Pangu_AAeg	gb|AAGE02019155.1|:265379-265663
Pangu_AAeg	gb|AAGE02018391.1|:113423-113709

Pangu_AAeg	gb|AAGE02014496.1|:45537-45823
Pangu_AAeg	gb|AAGE02010064.1|:87753-88038
Pangu_AAeg	gb|AAGE02002711.1|:153425-153707
Pangu_AAeg	gb|AAGE02011736.1|:510240-510515
Pangu_AAeg	gb|AAGE02011736.1|:268685-268970
Pangu_AAeg	gb|AAGE02021311.1|:91709-91994
Pangu_AAeg	gb|AAGE02010983.1|:197681-197964
Pangu_AAeg	gb|AAGE02008899.1|:134188-134473
Pangu_AAeg	gb|AAGE02005388.1|:171246-171531
Pangu_AAeg	gb|AAGE02005388.1|:19931-21002
Pangu_AAeg	gb|AAGE02005254.1|:257312-257597
Pangu_AAeg	gb|AAGE02000588.1|:48135-48417
Pangu_AAeg	gb|AAGE02011344.1|:99406-99691
Pangu_AAeg	gb|AAGE02028020.1|:59349-59634
Pangu_AAeg	gb|AAGE02020249.1|:31438-31723
Pangu_AAeg	gb|AAGE02007093.1|:231557-231842
Pangu_AAeg	gb|AAGE02003990.1|:4850-5135
Pangu_AAeg	gb|AAGE02003028.1|:74988-75265
Pangu_AAeg	gb|AAGE02001151.1|:53154-53439
Pangu_AAeg	gb|AAGE02002963.1|:79276-79551
Pangu_AAeg	gb|AAGE02029325.1|:3865-4151
Pangu_AAeg	gb|AAGE02020335.1|:22391-22675
Pangu_AAeg	gb|AAGE02019155.1|:130719-131003
Pangu_AAeg	gb|AAGE02001795.1|:357636-357917
Pangu_AAeg	gb|AAGE02010468.1|:22412-22696
Pangu_AAeg	gb|AAGE02019725.1|:214513-214791
Pangu_AAeg	gb|AAGE02002368.1|:31845-32128
Pangu_AAeg	gb|AAGE02021610.1|:245689-245971
Pangu_AAeg	gb|AAGE02029992.1|:61110-61393
Pangu_AAeg	gb|AAGE02027964.1|:16459-16743
Pangu_AAeg	gb|AAGE02018051.1|:17110-17394
Pangu_AAeg	gb|AAGE02008168.1|:13013-13297
Pangu_AAeg	gb|AAGE02006471.1|:140500-140784
Pangu_AAeg	gb|AAGE02002534.1|:15935-16218
Pangu_AAeg	gb|AAGE02000676.1|:178365-178647
Pangu_AAeg	gb|AAGE02000867.1|:2955-3238
Pangu_AAeg	gb|AAGE02022141.1|:90696-90980
Pangu_AAeg	gb|AAGE02006151.1|:122068-122350
Pangu_AAeg	gb|AAGE02034720.1|:4885-5169
Pangu_AAeg	gb|AAGE02033691.1|:9929-10213
Pangu_AAeg	gb|AAGE02032608.1|:6414-6698
Pangu_AAeg	gb|AAGE02027885.1|:1919-2203
Pangu_AAeg	gb|AAGE02014033.1|:35189-35473
Pangu_AAeg	gb|AAGE02005979.1|:92254-92538

Pangu_AAeg	gb|AAGE02003503.1|:134790-135065
Pangu_AAeg	gb|AAGE02001795.1|:247531-247815
Pangu_AAeg	gb|AAGE02001795.1|:218342-218616
Pangu_AAeg	gb|AAGE02015098.1|:72689-73590
Pangu_AAeg	gb|AAGE02009793.1|:12452-12732
Pangu_AAeg	gb|AAGE02011736.1|:373993-374274
Pangu_AAeg	gb|AAGE02026958.1|:20202-20512
Pangu_AAeg	gb|AAGE02013647.1|:16713-16993
Pangu_AAeg	gb|AAGE02006159.1|:69196-69468
Pangu_AAeg	gb|AAGE02009008.1|:231744-232028
Pangu_AAeg	gb|AAGE02027906.1|:6074-6357
Pangu_AAeg	gb|AAGE02025950.1|:6967-7250
Pangu_AAeg	gb|AAGE02018276.1|:74002-74283
Pangu_AAeg	gb|AAGE02024623.1|:44317-44602
Pangu_AAeg	gb|AAGE02011736.1|:143376-143678
Pangu_AAeg	gb|AAGE02018051.1|:28777-29059
Pangu_AAeg	gb|AAGE02010366.1|:19050-19332
Pangu_AAeg	gb|AAGE02005254.1|:215991-216273
Pangu_AAeg	gb|AAGE02000867.1|:143670-143952
Pangu_AAeg	gb|AAGE02000278.1|:45592-45874
Pangu_AAeg	gb|AAGE02010065.1|:19479-19760
Pangu_AAeg	gb|AAGE02006135.1|:105957-106238
Pangu_AAeg	gb|AAGE02016283.1|:155816-156099
Pangu_AAeg	gb|AAGE02016124.1|:45587-45865
Pangu_AAeg	gb|AAGE02008899.1|:47225-47504
Pangu_AAeg	gb|AAGE02006790.1|:150689-150966
Pangu_AAeg	gb|AAGE02016464.1|:44196-48570
Pangu_AAeg	gb|AAGE02008243.1|:223044-223323
Pangu_AAeg	gb|AAGE02003900.1|:254481-254762
Pangu_AAeg	gb|AAGE02016576.1|:37284-37556
Pangu_AAeg	gb|AAGE02000601.1|:401818-402094
Pangu_AAeg	gb|AAGE02004148.1|:55939-56216
Pangu_AAeg	gb|AAGE02007356.1|:33042-33319
Pangu_AAeg	gb|AAGE02006135.1|:71152-71426
Pangu_AAeg	gb|AAGE02011048.1|:17019-17299
Pangu_AAeg	gb|AAGE02003317.1|:99592-99969
Pangu_AAeg	gb|AAGE02003317.1|:29895-30174
Pangu_AAeg	gb|AAGE02015907.1|:206835-207124
Pangu_AAeg	gb|AAGE02000381.1|:72670-73062
Pangu_AAeg	gb|AAGE02016816.1|:2540-2810
Pangu_AAeg	gb|AAGE02011344.1|:54383-54660
Pangu_AAeg	gb|AAGE02007814.1|:58304-60175
Pangu_AAeg	gb|AAGE02026416.1|:95895-96173
Pangu_AAeg	gb|AAGE02021915.1|:111939-112207

Pangu_AAeg	gb|AAGE02004250.1|:256915-258653
Pangu_AAeg	gb|AAGE02018891.1|:11248-11524
Pangu_AAeg	gb|AAGE02003151.1|:161230-161511
Pangu_AAeg	gb|AAGE02012075.1|:7616-7885
Pangu_AAeg	gb|AAGE02020252.1|:32624-32903
Pangu_AAeg	gb|AAGE02006151.1|:464380-465601
Pangu_AAeg	gb|AAGE02001272.1|:41739-42015
Pangu_AAeg	gb|AAGE02011736.1|:493810-494090
Pangu_AAeg	gb|AAGE02010183.1|:25453-25724
Pangu_AAeg	gb|AAGE02006854.1|:22944-23218
Pangu_AAeg	gb|AAGE02006151.1|:439258-439530
Pangu_AAeg	gb|AAGE02013575.1|:177032-177305
Pangu_AAeg	gb|AAGE02016623.1|:80571-83222
Pangu_AAeg	gb|AAGE02001795.1|:45698-45967
Pangu_AAeg	gb|AAGE02010338.1|:224552-224824
Pangu_AAeg	gb|AAGE02017552.1|:151126-151731
Pangu_AAeg	gb|AAGE02010338.1|:29271-29536
Pangu_AAeg	gb|AAGE02006790.1|:81523-81793
Pangu_AAeg	gb|AAGE02009008.1|:146860-147146
Pangu_AAeg	gb|AAGE02011469.1|:106926-107192
Pangu_AAeg	gb|AAGE02012075.1|:186297-189500
Pangu_AAeg	gb|AAGE02006261.1|:44253-44512
Pangu_AAeg	gb|AAGE02011019.1|:29169-29432
Pangu_AAeg	gb|AAGE02018276.1|:36077-36344
Pangu_AAeg	gb|AAGE02016464.1|:124666-124932
Pangu_AAeg	gb|AAGE02008168.1|:52107-52369
Pangu_AAeg	gb|AAGE02002831.1|:22106-22373
Pangu_AAeg	gb|AAGE02010983.1|:246408-246674
Pangu_AAeg	gb|AAGE02018509.1|:239000-239298
Pangu_AAeg	gb|AAGE02007101.1|:214906-215170
Pangu_AAeg	gb|AAGE02002711.1|:1155-1409
Pangu_AAeg	gb|AAGE02013575.1|:348367-348643
Pangu_AAeg	gb|AAGE02009008.1|:62285-62547
Pangu_AAeg	gb|AAGE02001795.1|:324767-325026
Pangu_AAeg	gb|AAGE02012075.1|:145822-155935
Pangu_AAeg	gb|AAGE02011736.1|:160906-161170
Pangu_AAeg	gb|AAGE02018391.1|:245305-245557
Pangu_AAeg	gb|AAGE02017809.1|:76308-76583
Pangu_AAeg	gb|AAGE02022141.1|:39651-39929
Pangu_AAeg	gb|AAGE02002711.1|:81580-81836
Pangu_AAeg	gb|AAGE02010183.1|:131558-131819
Pangu_AAeg	gb|AAGE02000867.1|:126218-126468
Pangu_AAeg	gb|AAGE02021954.1|:61113-61361
Pangu_AAeg	gb|AAGE02008490.1|:53846-54104

Pangu_AAeg	gb|AAGE02017552.1|:68724-68982
Pangu_AAeg	gb|AAGE02007356.1|:338122-338381
Pangu_AAeg	gb|AAGE02016576.1|:6982-14430
Pangu_AAeg	gb|AAGE02007093.1|:55011-55293
Pangu_AAeg	gb|AAGE02013575.1|:194914-195178
Pangu_AAeg	gb|AAGE02004366.1|:27681-27939
Pangu_AAeg	gb|AAGE02011379.1|:21898-23457
Pangu_AAeg	gb|AAGE02003028.1|:61548-61808
Pangu_AAeg	gb|AAGE02001795.1|:310464-310735
Pangu_AAeg	gb|AAGE02010468.1|:64084-64335
Pangu_AAeg	gb|AAGE02014033.1|:11585-12107
Pangu_AAeg	gb|AAGE02001765.1|:43676-43920
Pangu_AAeg	gb|AAGE02006941.1|:66492-66744
Pangu_AAeg	gb|AAGE02016816.1|:42007-42251
Pangu_AAeg	gb|AAGE02009023.1|:48429-48680
Pangu_AAeg	gb|AAGE02018509.1|:18910-19161
Pangu_AAeg	gb|AAGE02006151.1|:525948-526199
Pangu_AAeg	gb|AAGE02019767.1|:14092-14551
Pangu_AAeg	gb|AAGE02013686.1|:126025-126277
Pangu_AAeg	gb|AAGE02008899.1|:101003-101261
Pangu_AAeg	gb|AAGE02000867.1|:233180-233430
Pangu_AAeg	gb|AAGE02005979.1|:144550-144800
Pangu_AAeg	gb|AAGE02030520.1|:70889-71140
Pangu_AAeg	gb|AAGE02002963.1|:140821-141077
Pangu_AAeg	gb|AAGE02021610.1|:81835-82661
Pangu_AAeg	gb|AAGE02000867.1|:45257-45507
Pangu_AAeg	gb|AAGE02029992.1|:6843-7094
Pangu_AAeg	gb|AAGE02005388.1|:184143-184388
Pangu_AAeg	gb|AAGE02006159.1|:102135-102379
Pangu_AAeg	gb|AAGE02014508.1|:2398-2633
Pangu_AAeg	gb|AAGE02024042.1|:152595-157275
Pangu_AAeg	gb|AAGE02006790.1|:104108-104348
Pangu_AAeg	gb|AAGE02007643.1|:92792-93032
Pangu_AAeg	gb|AAGE02003317.1|:45826-46067
Pangu_AAeg	gb|AAGE02003867.1|:129417-129658
Pangu_AAeg	gb|AAGE02002748.1|:349632-349875
Pangu_AAeg	gb|AAGE02000601.1|:38964-39206
Pangu_AAeg	gb|AAGE02010064.1|:23322-23562
Pangu_AAeg	gb|AAGE02008243.1|:115956-116195
Pangu_AAeg	gb|AAGE02009023.1|:252435-252683
Pangu_AAeg	gb|AAGE02006602.1|:28182-28415
Pangu_AAeg	gb|AAGE02013686.1|:78805-79043
Pangu_AAeg	gb|AAGE02007356.1|:148746-148988
Pangu_AAeg	gb|AAGE02006854.1|:219463-219697

Pangu_AAeg	gb|AAGE02008148.1|:37547-37783
Pangu_AAeg	gb|AAGE02008243.1|:16404-16633
Pangu_AAeg	gb|AAGE02026416.1|:120340-122181
Pangu_AAeg	gb|AAGE02015829.1|:95671-95906
Pangu_AAeg	gb|AAGE02011344.1|:80650-80886
Pangu_AAeg	gb|AAGE02002748.1|:27082-27316
Pangu_AAeg	gb|AAGE02008223.1|:37080-37313
Pangu_AAeg	gb|AAGE02006135.1|:211132-211393
Pangu_AAeg	gb|AAGE02003028.1|:93095-93328
Pangu_AAeg	gb|AAGE02011524.1|:48311-48546
Pangu_AAeg	gb|AAGE02015896.1|:35622-45192
Pangu_AAeg	gb|AAGE02007208.1|:61583-61812
Pangu_AAeg	gb|AAGE02003151.1|:136909-137136
Pangu_AAeg	gb|AAGE02010064.1|:124892-125122
Pangu_AAeg	gb|AAGE02000601.1|:52930-53157
Pangu_AAeg	gb|AAGE02011723.1|:84723-84943
Pangu_AAeg	gb|AAGE02008168.1|:90703-90929
Pangu_AAeg	gb|AAGE02007093.1|:345327-348485
Pangu_AAeg	gb|AAGE02001272.1|:115228-115445
Pangu_AAeg	gb|AAGE02010446.1|:60451-60676
Pangu_AAeg	gb|AAGE02001272.1|:19330-19555
Pangu_AAeg	gb|AAGE02015896.1|:251983-252197
Pangu_AAeg	gb|AAGE02003867.1|:17188-17408
Pangu_AAeg	gb|AAGE02008243.1|:368091-368303
Pangu_AAeg	gb|AAGE02015907.1|:187296-187527
Pangu_AAeg	gb|AAGE02022216.1|:17310-17533
Pangu_AAeg	gb|AAGE02007356.1|:349497-349722
Pangu_AAeg	gb|AAGE02008491.1|:33958-34180
Pangu_AAeg	gb|AAGE02001230.1|:78673-78893
Pangu_AAeg	gb|AAGE02018509.1|:30033-30251
Pangu_AAeg	gb|AAGE02026416.1|:82391-82612
Pangu_AAeg	gb|AAGE02011736.1|:449266-450895
Pangu_AAeg	gb|AAGE02001765.1|:91301-91517
Pangu_AAeg	gb|AAGE02016623.1|:117194-117411
Pangu_AAeg	gb|AAGE02003151.1|:2079-2302
Pangu_AAeg	gb|AAGE02016576.1|:85705-85944
Pangu_AAeg	gb|AAGE02016692.1|:36948-37165
Pangu_AAeg	gb|AAGE02004366.1|:62862-63079
Pangu_AAeg	gb|AAGE02003458.1|:13425-13643
Pangu_AAeg	gb|AAGE02000147.1|:13262-17744
Pangu_AAeg	gb|AAGE02010922.1|:154336-154550
Pangu_AAeg	gb|AAGE02002748.1|:69240-73762
Pangu_AAeg	gb|AAGE02025641.1|:105008-105223
Pangu_AAeg	gb|AAGE02019155.1|:183468-183677

Pangu_AAeg	gb|AAGE02002368.1|:56710-65874
Pangu_AAeg	gb|AAGE02018509.1|:226155-226367
Pangu_AAeg	gb|AAGE02010338.1|:296079-296501
Pangu_AAeg	gb|AAGE02006151.1|:161234-161446
Pangu_AAeg	gb|AAGE02004341.1|:110340-110553
Pangu_AAeg	gb|AAGE02006261.1|:132245-132453
Pangu_AAeg	gb|AAGE02008899.1|:181928-182135
Pangu_AAeg	gb|AAGE02015829.1|:60778-61030
Pangu_AAeg	gb|AAGE02013575.1|:288303-288509
Pangu_AAeg	gb|AAGE02010338.1|:9144-9353
Pangu_AAeg	gb|AAGE02018391.1|:178161-178367
Pangu_AAeg	gb|AAGE02001795.1|:1418-1617
Pangu_AAeg	gb|AAGE02010232.1|:131940-132148
Pangu_AAeg	gb|AAGE02006338.1|:22742-22999
Pangu_AAeg	gb|AAGE02016393.1|:83358-83566
Pangu_AAeg	gb|AAGE02003151.1|:227511-227718
Pangu_AAeg	gb|AAGE02001795.1|:438840-439044
Pangu_AAeg	gb|AAGE02007855.1|:83125-83329
Pangu_AAeg	gb|AAGE02004627.1|:6366-6571
Pangu_AAeg	gb|AAGE02015098.1|:128922-129126
Pangu_AAeg	gb|AAGE02004341.1|:148741-148945
Pangu_AAeg	gb|AAGE02012075.1|:110039-110244
Pangu_AAeg	gb|AAGE02008243.1|:322946-323153
Pangu_AAeg	gb|AAGE02026416.1|:65811-66015
Pangu_AAeg	gb|AAGE02017552.1|:109594-109798
Pangu_AAeg	gb|AAGE02005486.1|:18753-18958
Pangu_AAeg	gb|AAGE02011736.1|:343123-343327
Pangu_AAeg	gb|AAGE02006151.1|:185383-185586
Pangu_AAeg	gb|AAGE02022216.1|:59727-59930
Pangu_AAeg	gb|AAGE02002748.1|:255730-255933
Pangu_AAeg	gb|AAGE02001849.1|:28854-29057
Pangu_AAeg	gb|AAGE02010417.1|:121054-121257
Pangu_AAeg	gb|AAGE02016464.1|:253105-253310
Pangu_AAeg	gb|AAGE02021915.1|:98759-98964
Pangu_AAeg	gb|AAGE02010232.1|:19275-19472
Pangu_AAeg	gb|AAGE02010064.1|:41893-42094
Pangu_AAeg	gb|AAGE02003317.1|:146491-146692
Pangu_AAeg	gb|AAGE02024042.1|:189106-189307
Pangu_AAeg	gb|AAGE02010417.1|:91704-91905
Pangu_AAeg	gb|AAGE02016464.1|:86881-87085
Pangu_AAeg	gb|AAGE02019338.1|:35460-35661
Pangu_AAeg	gb|AAGE02000676.1|:190997-191193
Pangu_AAeg	gb|AAGE02004250.1|:95206-95407
Pangu_AAeg	gb|AAGE02000278.1|:5143-5344

Pangu_AAeg	gb|AAGE02013575.1|:139678-139873
Pangu_AAeg	gb|AAGE02009023.1|:26241-26447
Pangu_AAeg	gb|AAGE02008491.1|:138348-138549
Pangu_AAeg	gb|AAGE02008491.1|:63485-63683
Pangu_AAeg	gb|AAGE02003900.1|:95201-95403
Pangu_AAeg	gb|AAGE02003709.1|:18751-18952
Pangu_AAeg	gb|AAGE02018276.1|:62833-63034
Pangu_AAeg	gb|AAGE02001572.1|:58523-58726
Pangu_AAeg	gb|AAGE02015896.1|:204763-204965
Pangu_AAeg	gb|AAGE02013748.1|:6256-6457
Pangu_AAeg	gb|AAGE02011736.1|:354202-354402
Pangu_AAeg	gb|AAGE02007101.1|:262556-262756
Pangu_AAeg	gb|AAGE02006492.1|:20891-21094
Pangu_AAeg	gb|AAGE02015098.1|:91300-91501
Pangu_AAeg	gb|AAGE02013647.1|:49757-49956
Pangu_AAeg	gb|AAGE02000588.1|:30733-30931
Pangu_AAeg	gb|AAGE02013575.1|:163924-164122
Pangu_AAeg	gb|AAGE02024623.1|:8400-8599
Pangu_AAeg	gb|AAGE02019338.1|:64499-64695
Pangu_AAeg	gb|AAGE02019155.1|:67317-67513
Pangu_AAeg	gb|AAGE02000601.1|:274045-274232
Pangu_AAeg	gb|AAGE02015896.1|:6486-6684
Pangu_AAeg	gb|AAGE02003900.1|:126325-126745
Pangu_AAeg	gb|AAGE02006492.1|:7167-7418
Pangu_AAeg	gb|AAGE02020810.1|:34055-34247
Pangu_AAeg	gb|AAGE02008490.1|:8430-15752
Pangu_AAeg	gb|AAGE02009008.1|:368012-368206
Pangu_AAeg	gb|AAGE02009008.1|:102693-102910
Pangu_AAeg	gb|AAGE02007105.1|:3797-4609
Pangu_AAeg	gb|AAGE02011379.1|:71581-71775
Pangu_AAeg	gb|AAGE02009023.1|:95122-95312
Pangu_AAeg	gb|AAGE02011379.1|:59554-59995
Pangu_AAeg	gb|AAGE02021163.1|:16625-16818
Pangu_AAeg	gb|AAGE02011344.1|:68952-69142
Pangu_AAeg	gb|AAGE02020249.1|:72243-72438
Pangu_AAeg	gb|AAGE02008243.1|:71306-71494
Pangu_AAeg	gb|AAGE02018391.1|:89568-89759
Pangu_AAeg	gb|AAGE02006261.1|:16551-16738
Pangu_AAeg	gb|AAGE02005388.1|:211281-211467
Pangu_AAeg	gb|AAGE02000676.1|:8456-8642
Pangu_AAeg	gb|AAGE02018429.1|:59839-60024
Pangu_AAeg	gb|AAGE02016692.1|:11556-11741
Pangu_AAeg	gb|AAGE02011469.1|:227286-227471
Pangu_AAeg	gb|AAGE02010232.1|:31110-31295

Pangu_AAeg	gb|AAGE02024042.1|:103510-103695
Pangu_AAeg	gb|AAGE02009481.1|:14030-14214
Pangu_AAeg	gb|AAGE02019725.1|:114783-114968
Pangu_AAeg	gb|AAGE02006602.1|:3597-3782
Pangu_AAeg	gb|AAGE02016642.1|:25994-26179
Pangu_AAeg	gb|AAGE02010922.1|:5454-5639
Pangu_AAeg	gb|AAGE02014173.1|:12425-12610
Pangu_AAeg	gb|AAGE02006941.1|:4906-5091
Pangu_AAeg	gb|AAGE02021610.1|:335606-335792
Pangu_AAeg	gb|AAGE02007208.1|:39267-39452
Pangu_AAeg	gb|AAGE02016124.1|:72885-73070
Pangu_AAeg	gb|AAGE02010983.1|:69336-69521
Pangu_AAeg	gb|AAGE02027964.1|:38417-38602
Pangu_AAeg	gb|AAGE02016393.1|:50273-50458
Pangu_AAeg	gb|AAGE02013647.1|:6414-6599
Pangu_AAeg	gb|AAGE02011723.1|:73420-73605
Pangu_AAeg	gb|AAGE02005388.1|:79592-79777
Pangu_AAeg	gb|AAGE02019338.1|:3322-3508
Pangu_AAeg	gb|AAGE02015829.1|:76791-76976
Pangu_AAeg	gb|AAGE02000676.1|:147630-147814
Pangu_AAeg	gb|AAGE02000676.1|:43664-43849
Pangu_AAeg	gb|AAGE02000588.1|:220027-220212
Pangu_AAeg	gb|AAGE02000588.1|:84965-85150
Pangu_AAeg	gb|AAGE02006854.1|:161442-161627
Pangu_AAeg	gb|AAGE02006159.1|:157986-158172
Pangu_AAeg	gb|AAGE02020810.1|:13870-14057
Pangu_AAeg	gb|AAGE02006151.1|:313179-313364
Pangu_AAeg	gb|AAGE02030520.1|:85474-85659
Pangu_AAeg	gb|AAGE02026416.1|:43854-44039
Pangu_AAeg	gb|AAGE02019767.1|:48065-48250
Pangu_AAeg	gb|AAGE02014973.1|:3196-3381
Pangu_AAeg	gb|AAGE02010442.1|:178903-179088
Pangu_AAeg	gb|AAGE02010442.1|:82177-82363
Pangu_AAeg	gb|AAGE02009023.1|:215049-215235
Pangu_AAeg	gb|AAGE02008491.1|:107838-108023
Pangu_AAeg	gb|AAGE02003900.1|:43204-43389
Pangu_AAeg	gb|AAGE02003503.1|:214276-214461
Pangu_AAeg	gb|AAGE02003028.1|:247095-247280
Pangu_AAeg	gb|AAGE02000896.1|:143725-143910
Pangu_AAeg	gb|AAGE02000768.1|:60969-61154
Pangu_AAeg	gb|AAGE02000768.1|:27889-28074
Pangu_AAeg	gb|AAGE02018276.1|:50606-50791
Pangu_AAeg	gb|AAGE02016305.1|:14125-14310
Pangu_AAeg	gb|AAGE02010338.1|:189135-189362

Pangu_AAeg	gb|AAGE02003151.1|:94007-94192
Pangu_AAeg	gb|AAGE02024623.1|:62963-63148
Pangu_AAeg	gb|AAGE02018429.1|:159896-160080
Pangu_AAeg	gb|AAGE02001795.1|:493018-493202
Pangu_AAeg	gb|AAGE02007855.1|:29832-30018
Pangu_AAeg	gb|AAGE02016216.1|:2627-2811
Pangu_AAeg	gb|AAGE02015098.1|:173356-173541
Pangu_AAeg	gb|AAGE02021610.1|:424814-424998
Pangu_AAeg	gb|AAGE02011736.1|:405613-405797
Pangu_AAeg	gb|AAGE02021311.1|:6526-6710
Pangu_AAeg	gb|AAGE02018509.1|:251579-251763
Pangu_AAeg	gb|AAGE02000278.1|:30026-30209
Pangu_AAeg	gb|AAGE02007093.1|:282063-282247
Pangu_AAeg	gb|AAGE02007093.1|:187137-187321
Pangu_AAeg	gb|AAGE02006151.1|:536453-536637
Pangu_AAeg	gb|AAGE02006151.1|:345051-345236
Pangu_AAeg	gb|AAGE02009318.1|:54099-54283
Pangu_AAeg	gb|AAGE02009111.1|:31097-31282
Pangu_AAeg	gb|AAGE02009008.1|:357173-357357
Pangu_AAeg	gb|AAGE02006135.1|:299817-300001
Pangu_AAeg	gb|AAGE02019372.1|:20569-20753
Pangu_AAeg	gb|AAGE02010446.1|:34170-34356
Pangu_AAeg	gb|AAGE02007105.1|:165186-165370
Pangu_AAeg	gb|AAGE02003151.1|:57523-57707
Pangu_AAeg	gb|AAGE02001272.1|:76293-76477
Pangu_AAeg	gb|AAGE02000601.1|:195246-195430
Pangu_AAeg	gb|AAGE02016642.1|:141085-141264
Pangu_AAeg	gb|AAGE02011736.1|:536069-536254
Pangu_AAeg	gb|AAGE02025641.1|:73116-73299
Pangu_AAeg	gb|AAGE02000601.1|:363924-364107
Pangu_AAeg	gb|AAGE02004665.1|:10239-10425
Pangu_AAeg	gb|AAGE02001151.1|:63537-63721
Pangu_AAeg	gb|AAGE02010446.1|:4535-4711
Pangu_AAeg	gb|AAGE02007093.1|:17298-17480
Pangu_AAeg	gb|AAGE02017552.1|:37881-38062
Pangu_AAeg	gb|AAGE02011019.1|:43556-43736
Pangu_AAeg	gb|AAGE02011469.1|:239970-240150
Pangu_AAeg	gb|AAGE02021425.1|:44760-44939
Pangu_AAeg	gb|AAGE02011736.1|:62163-62367
Pangu_AAeg	gb|AAGE02016393.1|:110550-110729
Pangu_AAeg	gb|AAGE02018509.1|:122036-122215
Pangu_AAeg	gb|AAGE02023178.1|:14244-14423
Pangu_AAeg	gb|AAGE02021915.1|:69909-70089
Pangu_AAeg	gb|AAGE02021753.1|:789-968

Pangu_AAeg	gb|AAGE02016283.1|:60531-60708
Pangu_AAeg	gb|AAGE02018391.1|:205664-205843
Pangu_AAeg	gb|AAGE02004627.1|:103630-103808
Pangu_AAeg	gb|AAGE02002368.1|:94162-101223
Pangu_AAeg	gb|AAGE02021432.1|:31805-31983
Pangu_AAeg	gb|AAGE02010338.1|:121983-122160
Pangu_AAeg	gb|AAGE02006854.1|:46751-46928
Pangu_AAeg	gb|AAGE02000601.1|:148564-154969
Pangu_AAeg	gb|AAGE02007105.1|:103941-104124
Pangu_AAeg	gb|AAGE02000768.1|:125179-125354
Pangu_AAeg	gb|AAGE02011736.1|:220191-220364
Pangu_AAeg	gb|AAGE02019155.1|:36301-37608
Pangu_AAeg	gb|AAGE02018391.1|:161688-161861
Pangu_AAeg	gb|AAGE02009793.1|:31564-31729
Pangu_AAeg	gb|AAGE02018276.1|:23280-23442
Pangu_AAeg	gb|AAGE02000676.1|:70994-71162
Pangu_AAeg	gb|AAGE02003151.1|:189962-190131
Pangu_AAeg	gb|AAGE02025641.1|:55855-56025
Pangu_AAeg	gb|AAGE02018714.1|:53101-53265
Pangu_AAeg	gb|AAGE02006941.1|:43908-44798
Pangu_AAeg	gb|AAGE02007101.1|:245986-246162
Pangu_AAeg	gb|AAGE02002303.1|:22214-22378
Pangu_AAeg	gb|AAGE02025797.1|:2343-2504
Pangu_AAeg	gb|AAGE02010417.1|:11344-11505
Pangu_AAeg	gb|AAGE02000867.1|:72630-72792
Pangu_AAeg	gb|AAGE02021915.1|:45835-45994
Pangu_AAeg	gb|AAGE02013575.1|:252989-257863
Pangu_AAeg	gb|AAGE02007356.1|:124357-124515
Pangu_AAeg	gb|AAGE02020252.1|:17790-17950
Pangu_AAeg	gb|AAGE02011736.1|:184546-184717
Pangu_AAeg	gb|AAGE02007822.1|:289936-290095
Pangu_AAeg	gb|AAGE02011469.1|:50551-50709
Pangu_AAeg	gb|AAGE02010338.1|:371159-371363
Pangu_AAeg	gb|AAGE02006601.1|:10914-11256
Pangu_AAeg	gb|AAGE02001543.1|:16684-16838
Pangu_AAeg	gb|AAGE02016464.1|:101961-102117
Pangu_AAeg	gb|AAGE02024042.1|:30527-30680
Pangu_AAeg	gb|AAGE02010922.1|:58857-59008
Pangu_AAeg	gb|AAGE02002711.1|:199496-199647
Pangu_AAeg	gb|AAGE02010983.1|:122449-122599
Pangu_AAeg	gb|AAGE02000588.1|:19747-19896
Pangu_AAeg	gb|AAGE02016642.1|:187588-187736
Pangu_AAeg	gb|AAGE02004250.1|:139012-139161
Pangu_AAeg	gb|AAGE02009481.1|:49762-49946

Pangu_AAeg	gb|AAGE02010338.1|:435409-435557
Pangu_AAeg	gb|AAGE02005802.1|:30842-31007
Pangu_AAeg	gb|AAGE02029992.1|:20477-20654
Pangu_AAeg	gb|AAGE02008243.1|:280398-280546
Pangu_AAeg	gb|AAGE02007814.1|:4709-4860
Pangu_AAeg	gb|AAGE02007356.1|:99302-99457
Pangu_AAeg	gb|AAGE02001795.1|:387110-387283
Pangu_AAeg	gb|AAGE02000372.1|:17272-17418
Pangu_AAeg	gb|AAGE02013686.1|:45710-45857
Pangu_AAeg	gb|AAGE02009318.1|:104621-104774
Pangu_AAeg	gb|AAGE02007809.1|:1863-2007
Pangu_AAeg	gb|AAGE02009008.1|:301160-308170
Pangu_AAeg	gb|AAGE02024042.1|:119558-119705
Pangu_AAeg	gb|AAGE02008168.1|:113782-113926
Pangu_AAeg	gb|AAGE02019338.1|:109034-109177
Pangu_AAeg	gb|AAGE02013793.1|:25891-26083
Pangu_AAeg	gb|AAGE02016464.1|:135032-135193
Pangu_AAeg	gb|AAGE02005254.1|:178345-178488
Pangu_AAeg	gb|AAGE02030520.1|:35733-35899
Pangu_AAeg	gb|AAGE02009111.1|:68189-68332
Pangu_AAeg	gb|AAGE02005979.1|:57124-57266
Pangu_AAeg	gb|AAGE02005979.1|:43007-43149
Pangu_AAeg	gb|AAGE02016623.1|:37739-37881
Pangu_AAeg	gb|AAGE02011469.1|:61778-61920
Pangu_AAeg	gb|AAGE02003503.1|:180983-181124
Pangu_AAeg	gb|AAGE02001795.1|:293050-298523
Pangu_AAeg	gb|AAGE02007356.1|:372820-372959
Pangu_AAeg	gb|AAGE02005802.1|:3513-3649
Pangu_AAeg	gb|AAGE02005254.1|:348659-348785
Pangu_AAeg	gb|AAGE02025980.1|:11673-11809
Pangu_AAeg	gb|AAGE02000676.1|:296677-296813
Pangu_AAeg	gb|AAGE02000867.1|:21024-21159
Pangu_AAeg	gb|AAGE02002748.1|:220860-220996
Pangu_AAeg	gb|AAGE02000601.1|:306406-306538
Pangu_AAeg	gb|AAGE02009008.1|:42230-42363
Pangu_AAeg	gb|AAGE02028004.1|:42583-42713
Pangu_AAeg	gb|AAGE02007093.1|:29008-29138
Pangu_AAeg	gb|AAGE02013575.1|:92185-92315
Pangu_AAeg	gb|AAGE02005254.1|:98667-107771
Pangu_AAeg	gb|AAGE02021505.1|:24006-24136
Pangu_AAeg	gb|AAGE02001572.1|:86407-86537
Pangu_AAeg	gb|AAGE02019725.1|:201813-201941
Pangu_AAeg	gb|AAGE02016642.1|:160741-160869
Pangu_AAeg	gb|AAGE02010183.1|:36333-36462

Pangu_AAeg	gb|AAGE02021710.1|:72628-72756
Pangu_AAeg	gb|AAGE02018407.1|:24147-24293
Pangu_AAeg	gb|AAGE02000867.1|:270775-270903
Pangu_AAeg	gb|AAGE02010338.1|:41599-41782
Pangu_AAeg	gb|AAGE02001795.1|:163292-163417
Pangu_AAeg	gb|AAGE02019338.1|:131863-131987
Pangu_AAeg	gb|AAGE02010338.1|:150815-150930
Pangu_AAeg	gb|AAGE02010417.1|:176516-185719
Pangu_AAeg	gb|AAGE02016642.1|:116682-119466
Pangu_AAeg	gb|AAGE02007356.1|:296786-296901
Pangu_AAeg	gb|AAGE02019155.1|:169537-169656
Pangu_AAeg	gb|AAGE02000601.1|:26798-26917
Pangu_AAeg	gb|AAGE02004665.1|:41678-41796
Pangu_AAeg	gb|AAGE02015098.1|:19240-19356
Pangu_AAeg	gb|AAGE02021610.1|:58995-59112
Pangu_AAeg	gb|AAGE02007101.1|:118652-118763
Pangu_AAeg	gb|AAGE02003900.1|:112265-112381
Pangu_AAeg	gb|AAGE02006151.1|:553663-553771
Pangu_AAeg	gb|AAGE02015896.1|:236405-236519
Pangu_AAeg	gb|AAGE02016642.1|:64090-64202
Pangu_AAeg	gb|AAGE02011344.1|:175583-175695
Pangu_AAeg	gb|AAGE02004250.1|:282583-282692
Pangu_AAeg	gb|AAGE02019767.1|:3063-3175
Pangu_AAeg	gb|AAGE02008899.1|:36725-36835
Pangu_AAeg	gb|AAGE02004366.1|:74366-74474
Pangu_AAeg	gb|AAGE02006338.1|:38054-38163
Pangu_AAeg	gb|AAGE02001765.1|:243511-243618
Pangu_AAeg	gb|AAGE02000676.1|:60466-60570
Pangu_AAeg	gb|AAGE02018509.1|:176867-176970
Pangu_AAeg	gb|AAGE02010825.1|:3467-3569
Pangu_AAeg	gb|AAGE02009023.1|:77993-78118
Pangu_AAeg	gb|AAGE02021915.1|:80760-80856
Pangu_AAeg	gb|AAGE02010232.1|:96685-96783
Pangu_AAeg	gb|AAGE02021197.1|:281-377
Pangu_AAeg	gb|AAGE02011344.1|:128706-128805
Pangu_AAeg	gb|AAGE02003151.1|:19065-19164
Pangu_AAeg	gb|AAGE02021311.1|:141707-141803
Pangu_AAeg	gb|AAGE02010983.1|:92187-92283
Pangu_AAeg	gb|AAGE02006261.1|:68172-72522
Pangu_AAeg	gb|AAGE02006159.1|:121047-121139
Pangu_AAeg	gb|AAGE02007822.1|:97458-97550
Pangu_AAeg	gb|AAGE02010338.1|:80703-80794
Pangu_AAeg	gb|AAGE02007488.1|:17144-17234
Pangu_AAeg	gb|AAGE02005254.1|:244171-244258

Pangu_AAeg	gb|AAGE02000676.1|:266262-266351
Pangu_AAeg	gb|AAGE02007356.1|:315084-315173
Pangu_AAeg	gb|AAGE02008899.1|:167050-167137
Pangu_AAeg	gb|AAGE02004250.1|:219265-219352
Pangu_AAeg	gb|AAGE02011736.1|:50359-50445
Pangu_AAeg	gb|AAGE02005979.1|:14001-14086
Pangu_AAeg	gb|AAGE02004665.1|:53201-53287
Pangu_AAeg	gb|AAGE02007093.1|:148016-148101
Pangu_AAeg	gb|AAGE02003900.1|:11027-11113
Pangu_AAeg	gb|AAGE02002748.1|:132017-132103
Pangu_AAeg	gb|AAGE02000601.1|:83091-83174
Pangu_AAeg	gb|AAGE02001795.1|:450447-450531
Pangu_AAeg	gb|AAGE02016216.1|:53283-53367
Pangu_AAeg	gb|AAGE02001765.1|:144593-144676
Pangu_AAeg	gb|AAGE02002121.1|:86239-86320
Pangu_AAeg	gb|AAGE02013575.1|:224014-224095
Pangu_AAeg	gb|AAGE02007093.1|:304694-304772
Pangu_AAeg	gb|AAGE02016623.1|:68798-68878
Pangu_AAeg	gb|AAGE02006151.1|:214439-214517
Pangu_AAeg	gb|AAGE02013575.1|:50670-50748
Pangu_AAeg	gb|AAGE02007101.1|:180324-180399
Pangu_AAeg	gb|AAGE02005254.1|:17625-17699
Pangu_AAeg	gb|AAGE02007093.1|:41225-41300
Pangu_AAeg	gb|AAGE02017552.1|:138940-139015
Pangu_AAeg	gb|AAGE02009008.1|:261124-261200
Pangu_AAeg	gb|AAGE02010338.1|:388363-388438
Pangu_AAeg	gb|AAGE02006854.1|:36230-36304
Pangu_AAeg	gb|AAGE02004250.1|:169208-169307
Pangu_AAeg	gb|AAGE02008491.1|:224923-224995
Pangu_AAeg	gb|AAGE02018391.1|:137688-137759
Pangu_AAeg	gb|AAGE02010417.1|:212812-212881
Pangu_AAeg	gb|AAGE02021505.1|:13332-13400
Pangu_AAeg	gb|AAGE02008491.1|:122208-122276
Pangu_AAeg	gb|AAGE02001795.1|:80773-80840
Pangu_AAeg	gb|AAGE02005254.1|:58778-58845
Pangu_AAeg	gb|AAGE02005254.1|:29176-29243
Pangu_AAeg	gb|AAGE02021505.1|:44464-44530
Pangu_AAeg	gb|AAGE02000867.1|:185510-185577
Pangu_AAeg	gb|AAGE02006135.1|:179237-179325
Pangu_AAeg	gb|AAGE02021610.1|:294830-294893
Pangu_AAeg	gb|AAGE02003503.1|:18351-18415
Pangu_AAeg	gb|AAGE02001151.1|:1086-1150
Pangu_AAeg	gb|AAGE02001572.1|:148563-148627
Pangu_AAeg	gb|AAGE02006941.1|:27811-27874

	Pangu_AAeg	gb|AAGE02030335.1|:27583-27645
	Pangu_AAeg	gb|AAGE02006135.1|:55817-55879
	Pangu_AAeg	gb|AAGE02007105.1|:121385-121446
	Pangu_AAeg	gb|AAGE02006854.1|:343097-343157
	Pangu_AAeg	gb|AAGE02007714.1|:52906-52965
	Pangu_AAeg	gb|AAGE02001765.1|:118408-118466
	Pangu_AAeg	gb|AAGE02012075.1|:29038-29095
	Pangu_AAeg	gb|AAGE02020249.1|:45453-45510
	Pangu_AAeg	gb|AAGE02007356.1|:236052-236108
	Pangu_AAeg	gb|AAGE02017552.1|:80063-80117
	Pangu_AAeg	gb|AAGE02004250.1|:18022-18074
	Pangu_AAeg	gb|AAGE02010183.1|:85839-85889
	Pangu_AAeg	gb|AAGE02008899.1|:90655-90704
Anoplophora glabripennis	Pangu_AGl	gb|AQHT01012608.1|:1931-4707
	Pangu_AGl	gb|AQHT01090101.1|:213-2991
	Pangu_AGl	gb|AQHT01005958.1|:652-3380
	Pangu_AGl	gb|AQHT01073159.1|:4695-6043
	Pangu_AGl	gb|AQHT01028965.1|:356-1734
Chondrus crispus	Pangu-1_Ccri	emb|CAKH01002428.1|:14630-17326
	Pangu-1_Ccri	emb|CAKH01000227.1|:8045-8106
	Pangu-1_Ccri	emb|CAKH01000227.1|:6550-9471
	Pangu-1_Ccri	emb|CAKH01000284.1|:20809-20863
	Pangu-1_Ccri	emb|CAKH01000284.1|:19526-22228
	Pangu-1_Ccri	emb|CAKH01000980.1|:6580-6634
	Pangu-1_Ccri	emb|CAKH01000980.1|:5430-7999
	Pangu-1_Ccri	emb|CAKH01003140.1|:60207-62936
	Pangu-1_Ccri	emb|CAKH01001685.1|:4-1392
	Pangu-1_Ccri	emb|CAKH01002323.1|:98707-101389
	Pangu-1_Ccri	emb|CAKH01002146.1|:67138-69745
	Pangu-1_Ccri	emb|CAKH01002517.1|:62146-64830
	Pangu-1_Ccri	emb|CAKH01000542.1|:121976-125295
	Pangu-1_Ccri	emb|CAKH01001036.1|:1468-3440
	Pangu-1_Ccri	emb|CAKH01001686.1|:6352-7412
	Pangu-1_Ccri	emb|CAKH01002829.1|:91369-92525
	Pangu-1_Ccri	emb|CAKH01002340.1|:75738-78386
	Pangu-1_Ccri	emb|CAKH01002523.1|:5699-6012
	Pangu-1_Ccri	emb|CAKH01003051.1|:48463-48539
	Pangu-1_Ccri	emb|CAKH01003112.1|:130280-130501
	Pangu-1_Ccri	emb|CAKH01000258.1|:387-475
	Pangu-1_Ccri	emb|CAKH01001967.1|:70249-70603
	Pangu-1_Ccri	emb|CAKH01001144.1|:47081-47435
	Pangu-1_Ccri	emb|CAKH01002695.1|:89639-89689
	Pangu-1_Ccri	emb|CAKH01001647.1|:7655-7737
	Pangu-1_Ccri	emb|CAKH01001172.1|:8570-8683

Pangu-1_Ccri	emb|CAKH01000928.1|:9862-10087
Pangu-1_Ccri	emb|CAKH01000863.1|:50588-50942
Pangu-1_Ccri	emb|CAKH01000370.1|:9555-9909
Pangu-1_Ccri	emb|CAKH01000197.1|:4017-4126
Pangu-1_Ccri	emb|CAKH01000793.1|:151604-152544
Pangu-1_Ccri	emb|CAKH01003086.1|:19427-19538
Pangu-2_Ccri	emb|CAKH01002825.1|:3670-6170
Pangu-2_Ccri	emb|CAKH01002789.1|:22-905
Pangu-2_Ccri	emb|CAKH01002789.1|:8203-10687
Pangu-2_Ccri	emb|CAKH01002045.1|:50473-51376
Pangu-2_Ccri	emb|CAKH01001967.1|:28290-29181
Pangu-2_Ccri	emb|CAKH01002628.1|:20546-21438
Pangu-2_Ccri	emb|CAKH01000730.1|:35057-35927
Pangu-2_Ccri	emb|CAKH01003108.1|:3735-4628
Pangu-2_Ccri	emb|CAKH01002098.1|:1236-2127
Pangu-2_Ccri	emb|CAKH01002259.1|:1-882
Pangu-2_Ccri	emb|CAKH01002928.1|:2400-3291
Pangu-2_Ccri	emb|CAKH01003223.1|:3555-4443
Pangu-2_Ccri	emb|CAKH01002842.1|:24469-25361
Pangu-2_Ccri	emb|CAKH01001365.1|:23694-24564
Pangu-2_Ccri	emb|CAKH01001223.1|:7876-8762
Pangu-2_Ccri	emb|CAKH01002074.1|:124753-125627
Pangu-2_Ccri	emb|CAKH01002584.1|:67720-68609
Pangu-2_Ccri	emb|CAKH01000186.1|:5013-6405
Pangu-2_Ccri	emb|CAKH01001707.1|:17922-18329
Pangu-2_Ccri	emb|CAKH01000303.1|:7105-7519
Pangu-2_Ccri	emb|CAKH01002781.1|:14506-15275
Pangu-2_Ccri	emb|CAKH01000739.1|:13583-14492
Pangu-2_Ccri	emb|CAKH01002910.1|:111512-111775
Pangu-2_Ccri	emb|CAKH01002910.1|:125435-126066
Pangu-2_Ccri	emb|CAKH01003225.1|:14283-14919
Pangu-2_Ccri	emb|CAKH01003224.1|:178-437
Pangu-2_Ccri	emb|CAKH01002635.1|:735-1285
Pangu-2_Ccri	emb|CAKH01002782.1|:64623-64731
Pangu-2_Ccri	emb|CAKH01001064.1|:630-714
Pangu-2_Ccri	emb|CAKH01003218.1|:108880-108937
Pangu-3_Ccri	emb|CAKH01001183.1|:27244-30529
Pangu-3_Ccri	emb|CAKH01002473.1|:112819-115493
Pangu-3_Ccri	emb|CAKH01002473.1|:129558-129678
Pangu-3_Ccri	emb|CAKH01002473.1|:71480-71577
Pangu-3_Ccri	emb|CAKH01002473.1|:51024-51090
Pangu-3_Ccri	emb|CAKH01002473.1|:38670-38736
Pangu-3_Ccri	emb|CAKH01002238.1|:1363-1469
Pangu-3_Ccri	emb|CAKH01002238.1|:15832-15938

Pangu-3_Ccri	emb|CAKH01002238.1|:43801-43921
Pangu-3_Ccri	emb|CAKH01002238.1|:66590-71456
Pangu-3_Ccri	emb|CAKH01002238.1|:68428-71456
Pangu-3_Ccri	emb|CAKH01002238.1|:42641-42951
Pangu-3_Ccri	emb|CAKH01002115.1|:335-2899
Pangu-3_Ccri	emb|CAKH01003092.1|:659-1278
Pangu-3_Ccri	emb|CAKH01002754.1|:41565-42185
Pangu-3_Ccri	emb|CAKH01002754.1|:29088-29706
Pangu-3_Ccri	emb|CAKH01002642.1|:25038-25657
Pangu-3_Ccri	emb|CAKH01002642.1|:12569-13187
Pangu-3_Ccri	emb|CAKH01002627.1|:39119-39206
Pangu-3_Ccri	emb|CAKH01002627.1|:59731-60350
Pangu-3_Ccri	emb|CAKH01002627.1|:72350-72455
Pangu-3_Ccri	emb|CAKH01002258.1|:44193-44284
Pangu-3_Ccri	emb|CAKH01002258.1|:58596-58731
Pangu-3_Ccri	emb|CAKH01002258.1|:158649-158904
Pangu-3_Ccri	emb|CAKH01002258.1|:20393-21012
Pangu-3_Ccri	emb|CAKH01002258.1|:7886-8504
Pangu-3_Ccri	emb|CAKH01000626.1|:10084-10606
Pangu-3_Ccri	emb|CAKH01000352.1|:9448-10067
Pangu-3_Ccri	emb|CAKH01000207.1|:11038-11657
Pangu-3_Ccri	emb|CAKH01000006.1|:20214-20328
Pangu-3_Ccri	emb|CAKH01000006.1|:127806-127855
Pangu-3_Ccri	emb|CAKH01000006.1|:140688-140737
Pangu-3_Ccri	emb|CAKH01000006.1|:179390-180009
Pangu-3_Ccri	emb|CAKH01000006.1|:191915-192534
Pangu-3_Ccri	emb|CAKH01000006.1|:204389-205007
Pangu-3_Ccri	emb|CAKH01000006.1|:163453-164072
Pangu-3_Ccri	emb|CAKH01000006.1|:150942-151595
Pangu-3_Ccri	emb|CAKH01003156.1|:3591-4210
Pangu-3_Ccri	emb|CAKH01001901.1|:38315-38934
Pangu-3_Ccri	emb|CAKH01001901.1|:25836-26454
Pangu-3_Ccri	emb|CAKH01001200.1|:244333-244423
Pangu-3_Ccri	emb|CAKH01001200.1|:258713-259332
Pangu-3_Ccri	emb|CAKH01001200.1|:246241-246860
Pangu-3_Ccri	emb|CAKH01001200.1|:224669-224774
Pangu-3_Ccri	emb|CAKH01001200.1|:213811-213916
Pangu-3_Ccri	emb|CAKH01001200.1|:91510-91606
Pangu-3_Ccri	emb|CAKH01001129.1|:2330-2950
Pangu-3_Ccri	emb|CAKH01001129.1|:42420-42675
Pangu-3_Ccri	emb|CAKH01001044.1|:2976-3096
Pangu-3_Ccri	emb|CAKH01001044.1|:1521-2141
Pangu-3_Ccri	emb|CAKH01000882.1|:14307-14926
Pangu-3_Ccri	emb|CAKH01000882.1|:40094-40712

Pangu-3_Ccri	emb|CAKH01000810.1|:76591-76711
Pangu-3_Ccri	emb|CAKH01000810.1|:89551-89671
Pangu-3_Ccri	emb|CAKH01000810.1|:100055-100674
Pangu-3_Ccri	emb|CAKH01000810.1|:112558-113176
Pangu-3_Ccri	emb|CAKH01000810.1|:68190-68304
Pangu-3_Ccri	emb|CAKH01000537.1|:2715-3335
Pangu-3_Ccri	emb|CAKH01000243.1|:23582-23685
Pangu-3_Ccri	emb|CAKH01000243.1|:38068-38688
Pangu-3_Ccri	emb|CAKH01000243.1|:51062-51681
Pangu-3_Ccri	emb|CAKH01000243.1|:92894-92990
Pangu-3_Ccri	emb|CAKH01000243.1|:64566-64686
Pangu-3_Ccri	emb|CAKH01000243.1|:28834-28892
Pangu-3_Ccri	emb|CAKH01000213.1|:37304-37919
Pangu-3_Ccri	emb|CAKH01000213.1|:49779-50398
Pangu-3_Ccri	emb|CAKH01003105.1|:3474-4094
Pangu-3_Ccri	emb|CAKH01003082.1|:1274-1890
Pangu-3_Ccri	emb|CAKH01003082.1|:13820-14439
Pangu-3_Ccri	emb|CAKH01003082.1|:2836-3456
Pangu-3_Ccri	emb|CAKH01002177.1|:16854-17473
Pangu-3_Ccri	emb|CAKH01001908.1|:10690-10804
Pangu-3_Ccri	emb|CAKH01001908.1|:148484-148555
Pangu-3_Ccri	emb|CAKH01001908.1|:108711-109331
Pangu-3_Ccri	emb|CAKH01001908.1|:95719-96338
Pangu-3_Ccri	emb|CAKH01001908.1|:28111-28198
Pangu-3_Ccri	emb|CAKH01001908.1|:560-735
Pangu-3_Ccri	emb|CAKH01001812.1|:40656-41275
Pangu-3_Ccri	emb|CAKH01001812.1|:53167-53785
Pangu-3_Ccri	emb|CAKH01001812.1|:17890-18509
Pangu-3_Ccri	emb|CAKH01001812.1|:4916-5534
Pangu-3_Ccri	emb|CAKH01001785.1|:1015-1633
Pangu-3_Ccri	emb|CAKH01001726.1|:27013-27632
Pangu-3_Ccri	emb|CAKH01001726.1|:39489-40108
Pangu-3_Ccri	emb|CAKH01001726.1|:50934-52683
Pangu-3_Ccri	emb|CAKH01001726.1|:25380-25642
Pangu-3_Ccri	emb|CAKH01001633.1|:30358-30977
Pangu-3_Ccri	emb|CAKH01001336.1|:97879-98498
Pangu-3_Ccri	emb|CAKH01001336.1|:110357-110975
Pangu-3_Ccri	emb|CAKH01001336.1|:152360-152615
Pangu-3_Ccri	emb|CAKH01001336.1|:139110-139365
Pangu-3_Ccri	emb|CAKH01001336.1|:62842-63052
Pangu-3_Ccri	emb|CAKH01001336.1|:49782-49992
Pangu-3_Ccri	emb|CAKH01001310.1|:8384-9004
Pangu-3_Ccri	emb|CAKH01001310.1|:21370-21989
Pangu-3_Ccri	emb|CAKH01001310.1|:35087-35187

Pangu-3_Ccri	emb|CAKH01001310.1|:39170-39275
Pangu-3_Ccri	emb|CAKH01001214.1|:32631-32709
Pangu-3_Ccri	emb|CAKH01001214.1|:35075-35436
Pangu-3_Ccri	emb|CAKH01001051.1|:62594-62699
Pangu-3_Ccri	emb|CAKH01001051.1|:1-596
Pangu-3_Ccri	emb|CAKH01001017.1|:3316-3936
Pangu-3_Ccri	emb|CAKH01001017.1|:6042-6147
Pangu-3_Ccri	emb|CAKH01000580.1|:21936-22555
Pangu-3_Ccri	emb|CAKH01000580.1|:34416-35034
Pangu-3_Ccri	emb|CAKH01000379.1|:13120-13738
Pangu-3_Ccri	emb|CAKH01002944.1|:11153-11273
Pangu-3_Ccri	emb|CAKH01002944.1|:93017-93082
Pangu-3_Ccri	emb|CAKH01002944.1|:77368-77608
Pangu-3_Ccri	emb|CAKH01002944.1|:12557-13177
Pangu-3_Ccri	emb|CAKH01002833.1|:9964-10038
Pangu-3_Ccri	emb|CAKH01002833.1|:14662-15282
Pangu-3_Ccri	emb|CAKH01002376.1|:3299-3919
Pangu-3_Ccri	emb|CAKH01001469.1|:42745-42811
Pangu-3_Ccri	emb|CAKH01001469.1|:30438-30543
Pangu-3_Ccri	emb|CAKH01001469.1|:17533-18152
Pangu-3_Ccri	emb|CAKH01001912.1|:27727-28346
Pangu-3_Ccri	emb|CAKH01001789.1|:65523-66143
Pangu-3_Ccri	emb|CAKH01001789.1|:52539-53159
Pangu-3_Ccri	emb|CAKH01003227.1|:18014-18633
Pangu-3_Ccri	emb|CAKH01002279.1|:99617-99683
Pangu-3_Ccri	emb|CAKH01002279.1|:159940-160560
Pangu-3_Ccri	emb|CAKH01002279.1|:5995-6061
Pangu-3_Ccri	emb|CAKH01002003.1|:14646-15264
Pangu-3_Ccri	emb|CAKH01002003.1|:54328-54448
Pangu-3_Ccri	emb|CAKH01001977.1|:4669-5285
Pangu-3_Ccri	emb|CAKH01001977.1|:68888-68972
Pangu-3_Ccri	emb|CAKH01001977.1|:66605-66692
Pangu-3_Ccri	emb|CAKH01001977.1|:54011-54077
Pangu-3_Ccri	emb|CAKH01001839.1|:56530-57147
Pangu-3_Ccri	emb|CAKH01001839.1|:124473-124539
Pangu-3_Ccri	emb|CAKH01001839.1|:111401-111467
Pangu-3_Ccri	emb|CAKH01001839.1|:97634-98254
Pangu-3_Ccri	emb|CAKH01001839.1|:62220-62293
Pangu-3_Ccri	emb|CAKH01000878.1|:25838-26190
Pangu-3_Ccri	emb|CAKH01000878.1|:63034-63386
Pangu-3_Ccri	emb|CAKH01000878.1|:21525-21575
Pangu-3_Ccri	emb|CAKH01000485.1|:26449-26515
Pangu-3_Ccri	emb|CAKH01000485.1|:208719-208825
Pangu-3_Ccri	emb|CAKH01000485.1|:221183-221289

Pangu-3_Ccri	emb|CAKH01000485.1|:314510-314571
Pangu-3_Ccri	emb|CAKH01000485.1|:179228-179848
Pangu-3_Ccri	emb|CAKH01000485.1|:165143-165762
Pangu-3_Ccri	emb|CAKH01000485.1|:66425-66687
Pangu-3_Ccri	emb|CAKH01001940.1|:98249-98868
Pangu-3_Ccri	emb|CAKH01001940.1|:111124-111742
Pangu-3_Ccri	emb|CAKH01001940.1|:54376-54472
Pangu-3_Ccri	emb|CAKH01001631.1|:12644-13264
Pangu-3_Ccri	emb|CAKH01002332.1|:6735-7340
Pangu-3_Ccri	emb|CAKH01003210.1|:4397-5018
Pangu-3_Ccri	emb|CAKH01002739.1|:45668-46029
Pangu-3_Ccri	emb|CAKH01002739.1|:60025-60383
Pangu-3_Ccri	emb|CAKH01002739.1|:62785-62872
Pangu-3_Ccri	emb|CAKH01001406.1|:84249-84867
Pangu-3_Ccri	emb|CAKH01001406.1|:81286-81717
Pangu-3_Ccri	emb|CAKH01002937.1|:84463-84583
Pangu-3_Ccri	emb|CAKH01002937.1|:32748-33368
Pangu-3_Ccri	emb|CAKH01002937.1|:20660-21274
Pangu-3_Ccri	emb|CAKH01001285.1|:57689-58301
Pangu-3_Ccri	emb|CAKH01002716.1|:8579-9198
Pangu-3_Ccri	emb|CAKH01002716.1|:88592-88655
Pangu-3_Ccri	emb|CAKH01002716.1|:71422-71472
Pangu-3_Ccri	emb|CAKH01002716.1|:47732-47798
Pangu-3_Ccri	emb|CAKH01002716.1|:33199-33265
Pangu-3_Ccri	emb|CAKH01002551.1|:8486-8619
Pangu-3_Ccri	emb|CAKH01002551.1|:20584-20717
Pangu-3_Ccri	emb|CAKH01002551.1|:84128-84242
Pangu-3_Ccri	emb|CAKH01002551.1|:70794-71412
Pangu-3_Ccri	emb|CAKH01002551.1|:51706-52324
Pangu-3_Ccri	emb|CAKH01002551.1|:25283-25538
Pangu-3_Ccri	emb|CAKH01001059.1|:34021-34126
Pangu-3_Ccri	emb|CAKH01001059.1|:28206-28824
Pangu-3_Ccri	emb|CAKH01002297.1|:32044-32658
Pangu-3_Ccri	emb|CAKH01002297.1|:44485-45105
Pangu-3_Ccri	emb|CAKH01002297.1|:47233-47368
Pangu-3_Ccri	emb|CAKH01002171.1|:10089-10705
Pangu-3_Ccri	emb|CAKH01002259.1|:21894-22514
Pangu-3_Ccri	emb|CAKH01001124.1|:29206-29311
Pangu-3_Ccri	emb|CAKH01001124.1|:25164-25783
Pangu-3_Ccri	emb|CAKH01003189.1|:56220-56838
Pangu-3_Ccri	emb|CAKH01003189.1|:18116-18735
Pangu-3_Ccri	emb|CAKH01001881.1|:105664-106281
Pangu-3_Ccri	emb|CAKH01001445.1|:100721-101340
Pangu-3_Ccri	emb|CAKH01001445.1|:103472-103607

Pangu-3_Ccri	emb|CAKH01002960.1|:70172-70254
Pangu-3_Ccri	emb|CAKH01002960.1|:83812-83896
Pangu-3_Ccri	emb|CAKH01002960.1|:135714-135847
Pangu-3_Ccri	emb|CAKH01002960.1|:147581-147714
Pangu-3_Ccri	emb|CAKH01002960.1|:163867-163972
Pangu-3_Ccri	emb|CAKH01002960.1|:177395-178014
Pangu-3_Ccri	emb|CAKH01002960.1|:194382-194469
Pangu-3_Ccri	emb|CAKH01000682.1|:37647-37752
Pangu-3_Ccri	emb|CAKH01000682.1|:51279-51421
Pangu-3_Ccri	emb|CAKH01000682.1|:199048-199126
Pangu-3_Ccri	emb|CAKH01000682.1|:67461-68076
Pangu-3_Ccri	emb|CAKH01001588.1|:36776-37126
Pangu-3_Ccri	emb|CAKH01000212.1|:79814-79920
Pangu-3_Ccri	emb|CAKH01000212.1|:94100-94206
Pangu-3_Ccri	emb|CAKH01000212.1|:14071-14691
Pangu-3_Ccri	emb|CAKH01000100.1|:40771-41375
Pangu-3_Ccri	emb|CAKH01001222.1|:27527-28146
Pangu-3_Ccri	emb|CAKH01001222.1|:30274-30364
Pangu-3_Ccri	emb|CAKH01001077.1|:56855-56975
Pangu-3_Ccri	emb|CAKH01001077.1|:85998-86618
Pangu-3_Ccri	emb|CAKH01001077.1|:101085-101151
Pangu-3_Ccri	emb|CAKH01000850.1|:3260-3879
Pangu-3_Ccri	emb|CAKH01000850.1|:6007-6142
Pangu-3_Ccri	emb|CAKH01002318.1|:98353-98926
Pangu-3_Ccri	emb|CAKH01002318.1|:133937-134057
Pangu-3_Ccri	emb|CAKH01002318.1|:176053-176159
Pangu-3_Ccri	emb|CAKH01002318.1|:190426-190532
Pangu-3_Ccri	emb|CAKH01002318.1|:33681-33771
Pangu-3_Ccri	emb|CAKH01000418.1|:16857-17401
Pangu-3_Ccri	emb|CAKH01001118.1|:41077-41697
Pangu-3_Ccri	emb|CAKH01001118.1|:144497-144563
Pangu-3_Ccri	emb|CAKH01001118.1|:158214-158280
Pangu-3_Ccri	emb|CAKH01001118.1|:322633-322720
Pangu-3_Ccri	emb|CAKH01001118.1|:435326-435431
Pangu-3_Ccri	emb|CAKH01001118.1|:348123-348221
Pangu-3_Ccri	emb|CAKH01001118.1|:325792-325871
Pangu-3_Ccri	emb|CAKH01001118.1|:127674-127731
Pangu-3_Ccri	emb|CAKH01001118.1|:50189-50273
Pangu-3_Ccri	emb|CAKH01001821.1|:61499-62119
Pangu-3_Ccri	emb|CAKH01001821.1|:115843-115909
Pangu-3_Ccri	emb|CAKH01000111.1|:246729-247035
Pangu-3_Ccri	emb|CAKH01000111.1|:194222-194364
Pangu-3_Ccri	emb|CAKH01000111.1|:181816-181906
Pangu-3_Ccri	emb|CAKH01000111.1|:96326-96446

Pangu-3_Ccri	emb|CAKH01000111.1|:81994-82249
Pangu-3_Ccri	emb|CAKH01001011.1|:45778-46390
Pangu-3_Ccri	emb|CAKH01001011.1|:57233-57299
Pangu-3_Ccri	emb|CAKH01001673.1|:46957-47023
Pangu-3_Ccri	emb|CAKH01001673.1|:8065-8387
Pangu-3_Ccri	emb|CAKH01002921.1|:114472-114727
Pangu-3_Ccri	emb|CAKH01002921.1|:223812-224010
Pangu-3_Ccri	emb|CAKH01001591.1|:38796-40359
Pangu-3_Ccri	emb|CAKH01001591.1|:9772-9892
Pangu-3_Ccri	emb|CAKH01001954.1|:90752-94547
Pangu-3_Ccri	emb|CAKH01001688.1|:24022-24592
Pangu-3_Ccri	emb|CAKH01002889.1|:91643-92264
Pangu-3_Ccri	emb|CAKH01002889.1|:106081-106147
Pangu-3_Ccri	emb|CAKH01002889.1|:160239-160359
Pangu-3_Ccri	emb|CAKH01002889.1|:104068-104203
Pangu-3_Ccri	emb|CAKH01002889.1|:86814-86875
Pangu-3_Ccri	emb|CAKH01001961.1|:101076-101352
Pangu-3_Ccri	emb|CAKH01001961.1|:127996-128110
Pangu-3_Ccri	emb|CAKH01001961.1|:165590-165837
Pangu-3_Ccri	emb|CAKH01001961.1|:178102-178349
Pangu-3_Ccri	emb|CAKH01001961.1|:269346-269451
Pangu-3_Ccri	emb|CAKH01001961.1|:250930-251050
Pangu-3_Ccri	emb|CAKH01000663.1|:112239-112729
Pangu-3_Ccri	emb|CAKH01000663.1|:69612-69867
Pangu-3_Ccri	emb|CAKH01002153.1|:47017-47488
Pangu-3_Ccri	emb|CAKH01001320.1|:72845-73466
Pangu-3_Ccri	emb|CAKH01002366.1|:11590-12102
Pangu-3_Ccri	emb|CAKH01002366.1|:24365-24470
Pangu-3_Ccri	emb|CAKH01002366.1|:37044-37149
Pangu-3_Ccri	emb|CAKH01002366.1|:41533-41647
Pangu-3_Ccri	emb|CAKH01002907.1|:148912-148961
Pangu-3_Ccri	emb|CAKH01002907.1|:151262-151477
Pangu-3_Ccri	emb|CAKH01002907.1|:30940-31009
Pangu-3_Ccri	emb|CAKH01001389.1|:18319-18821
Pangu-3_Ccri	emb|CAKH01002880.1|:41232-41482
Pangu-3_Ccri	emb|CAKH01002880.1|:27416-27482
Pangu-3_Ccri	emb|CAKH01002880.1|:13439-13505
Pangu-3_Ccri	emb|CAKH01001418.1|:91227-91482
Pangu-3_Ccri	emb|CAKH01001418.1|:69364-69484
Pangu-3_Ccri	emb|CAKH01001418.1|:14453-14519
Pangu-3_Ccri	emb|CAKH01001023.1|:7775-8022
Pangu-3_Ccri	emb|CAKH01001023.1|:20259-20514
Pangu-3_Ccri	emb|CAKH01001023.1|:40864-41119
Pangu-3_Ccri	emb|CAKH01002616.1|:31529-32134

Pangu-3_Ccri	emb|CAKH01000672.1|:15509-15623
Pangu-3_Ccri	emb|CAKH01002905.1|:83709-83964
Pangu-3_Ccri	emb|CAKH01002148.1|:17181-17295
Pangu-3_Ccri	emb|CAKH01002148.1|:34611-34677
Pangu-3_Ccri	emb|CAKH01002148.1|:53028-53109
Pangu-3_Ccri	emb|CAKH01002148.1|:80826-81081
Pangu-3_Ccri	emb|CAKH01002148.1|:67060-67315
Pangu-3_Ccri	emb|CAKH01002148.1|:33953-34002
Pangu-3_Ccri	emb|CAKH01001990.1|:2182-2287
Pangu-3_Ccri	emb|CAKH01001990.1|:24668-24774
Pangu-3_Ccri	emb|CAKH01001990.1|:49045-49111
Pangu-3_Ccri	emb|CAKH01001990.1|:89105-89359
Pangu-3_Ccri	emb|CAKH01001990.1|:75601-75856
Pangu-3_Ccri	emb|CAKH01001990.1|:22553-22803
Pangu-3_Ccri	emb|CAKH01001700.1|:50927-51182
Pangu-3_Ccri	emb|CAKH01001394.1|:2862-3160
Pangu-3_Ccri	emb|CAKH01000499.1|:572-827
Pangu-3_Ccri	emb|CAKH01000499.1|:13816-14071
Pangu-3_Ccri	emb|CAKH01000499.1|:29822-30077
Pangu-3_Ccri	emb|CAKH01000488.1|:11101-11356
Pangu-3_Ccri	emb|CAKH01000335.1|:36072-36327
Pangu-3_Ccri	emb|CAKH01000335.1|:30030-30162
Pangu-3_Ccri	emb|CAKH01000335.1|:10390-10496
Pangu-3_Ccri	emb|CAKH01002490.1|:40403-40469
Pangu-3_Ccri	emb|CAKH01002490.1|:54439-54694
Pangu-3_Ccri	emb|CAKH01002490.1|:41249-41503
Pangu-3_Ccri	emb|CAKH01000448.1|:63655-63760
Pangu-3_Ccri	emb|CAKH01000448.1|:76240-76345
Pangu-3_Ccri	emb|CAKH01000448.1|:161956-162206
Pangu-3_Ccri	emb|CAKH01000448.1|:3415-3545
Pangu-3_Ccri	emb|CAKH01001284.1|:7359-7473
Pangu-3_Ccri	emb|CAKH01001284.1|:85064-85564
Pangu-3_Ccri	emb|CAKH01001284.1|:30988-31102
Pangu-3_Ccri	emb|CAKH01002615.1|:16569-16683
Pangu-3_Ccri	emb|CAKH01001720.1|:266710-266816
Pangu-3_Ccri	emb|CAKH01001720.1|:252627-252733
Pangu-3_Ccri	emb|CAKH01001720.1|:170013-170097
Pangu-3_Ccri	emb|CAKH01001720.1|:101758-101872
Pangu-3_Ccri	emb|CAKH01001525.1|:23641-23707
Pangu-3_Ccri	emb|CAKH01001525.1|:11704-11959
Pangu-3_Ccri	emb|CAKH01001225.1|:5664-5778
Pangu-3_Ccri	emb|CAKH01000979.1|:36117-36482
Pangu-3_Ccri	emb|CAKH01000979.1|:86267-86522
Pangu-3_Ccri	emb|CAKH01000979.1|:72988-73243

Pangu-3_Ccri	emb|CAKH01000692.1|:14827-15082
Pangu-3_Ccri	emb|CAKH01003222.1|:37776-38025
Pangu-3_Ccri	emb|CAKH01003215.1|:47348-47595
Pangu-3_Ccri	emb|CAKH01003215.1|:59832-60087
Pangu-3_Ccri	emb|CAKH01003100.1|:66073-66283
Pangu-3_Ccri	emb|CAKH01003100.1|:78327-78582
Pangu-3_Ccri	emb|CAKH01003100.1|:33366-33456
Pangu-3_Ccri	emb|CAKH01002613.1|:47153-47219
Pangu-3_Ccri	emb|CAKH01002613.1|:37430-37685
Pangu-3_Ccri	emb|CAKH01002613.1|:24942-25197
Pangu-3_Ccri	emb|CAKH01002576.1|:7335-7588
Pangu-3_Ccri	emb|CAKH01002576.1|:20599-20852
Pangu-3_Ccri	emb|CAKH01002576.1|:36395-36485
Pangu-3_Ccri	emb|CAKH01002576.1|:52236-52491
Pangu-3_Ccri	emb|CAKH01002576.1|:39748-40002
Pangu-3_Ccri	emb|CAKH01002560.1|:69132-69387
Pangu-3_Ccri	emb|CAKH01002560.1|:55393-55648
Pangu-3_Ccri	emb|CAKH01002560.1|:10309-10414
Pangu-3_Ccri	emb|CAKH01002534.1|:864-985
Pangu-3_Ccri	emb|CAKH01002534.1|:40895-41052
Pangu-3_Ccri	emb|CAKH01002499.1|:149996-150052
Pangu-3_Ccri	emb|CAKH01002499.1|:219215-219465
Pangu-3_Ccri	emb|CAKH01002499.1|:231647-231897
Pangu-3_Ccri	emb|CAKH01002499.1|:298711-298798
Pangu-3_Ccri	emb|CAKH01002499.1|:246902-247157
Pangu-3_Ccri	emb|CAKH01002499.1|:213788-214043
Pangu-3_Ccri	emb|CAKH01002499.1|:186116-186182
Pangu-3_Ccri	emb|CAKH01002499.1|:173770-173820
Pangu-3_Ccri	emb|CAKH01002499.1|:141667-141716
Pangu-3_Ccri	emb|CAKH01002499.1|:94087-94204
Pangu-3_Ccri	emb|CAKH01002441.1|:52064-52319
Pangu-3_Ccri	emb|CAKH01002262.1|:84243-84498
Pangu-3_Ccri	emb|CAKH01002262.1|:56010-56100
Pangu-3_Ccri	emb|CAKH01002262.1|:43454-43544
Pangu-3_Ccri	emb|CAKH01002180.1|:117710-117957
Pangu-3_Ccri	emb|CAKH01002180.1|:105216-105470
Pangu-3_Ccri	emb|CAKH01002180.1|:58460-58715
Pangu-3_Ccri	emb|CAKH01002093.1|:34390-34452
Pangu-3_Ccri	emb|CAKH01002093.1|:53043-53105
Pangu-3_Ccri	emb|CAKH01002093.1|:73990-74125
Pangu-3_Ccri	emb|CAKH01002093.1|:33674-33724
Pangu-3_Ccri	emb|CAKH01002093.1|:2081-2202
Pangu-3_Ccri	emb|CAKH01002029.1|:13833-14088
Pangu-3_Ccri	emb|CAKH01002029.1|:1545-1800

Pangu-3_Ccri	emb|CAKH01001972.1|:22264-22511
Pangu-3_Ccri	emb|CAKH01001972.1|:9777-10024
Pangu-3_Ccri	emb|CAKH01001941.1|:7292-7539
Pangu-3_Ccri	emb|CAKH01001928.1|:17764-17899
Pangu-3_Ccri	emb|CAKH01001913.1|:10149-10239
Pangu-3_Ccri	emb|CAKH01001913.1|:129910-130095
Pangu-3_Ccri	emb|CAKH01001913.1|:139881-139987
Pangu-3_Ccri	emb|CAKH01001913.1|:77966-78221
Pangu-3_Ccri	emb|CAKH01001913.1|:64273-64528
Pangu-3_Ccri	emb|CAKH01001875.1|:34949-35203
Pangu-3_Ccri	emb|CAKH01001875.1|:23097-23344
Pangu-3_Ccri	emb|CAKH01001875.1|:9148-9283
Pangu-3_Ccri	emb|CAKH01001780.1|:4501-4756
Pangu-3_Ccri	emb|CAKH01001731.1|:86998-87208
Pangu-3_Ccri	emb|CAKH01001731.1|:41371-42666
Pangu-3_Ccri	emb|CAKH01001658.1|:28536-28791
Pangu-3_Ccri	emb|CAKH01001658.1|:41018-41273
Pangu-3_Ccri	emb|CAKH01001658.1|:74709-74796
Pangu-3_Ccri	emb|CAKH01001658.1|:775-841
Pangu-3_Ccri	emb|CAKH01001392.1|:30532-30787
Pangu-3_Ccri	emb|CAKH01001392.1|:18336-18586
Pangu-3_Ccri	emb|CAKH01001168.1|:26498-26618
Pangu-3_Ccri	emb|CAKH01001168.1|:63782-64037
Pangu-3_Ccri	emb|CAKH01001168.1|:41429-41549
Pangu-3_Ccri	emb|CAKH01001168.1|:14696-14762
Pangu-3_Ccri	emb|CAKH01001168.1|:2646-2712
Pangu-3_Ccri	emb|CAKH01001162.1|:35552-35792
Pangu-3_Ccri	emb|CAKH01000728.1|:3863-4112
Pangu-3_Ccri	emb|CAKH01000728.1|:41648-41896
Pangu-3_Ccri	emb|CAKH01000728.1|:43660-43913
Pangu-3_Ccri	emb|CAKH01000520.1|:41336-41591
Pangu-3_Ccri	emb|CAKH01000520.1|:54546-54801
Pangu-3_Ccri	emb|CAKH01000415.1|:39296-39551
Pangu-3_Ccri	emb|CAKH01000127.1|:25821-25943
Pangu-3_Ccri	emb|CAKH01000127.1|:248914-249169
Pangu-3_Ccri	emb|CAKH01000127.1|:139884-139990
Pangu-3_Ccri	emb|CAKH01000127.1|:115288-115354
Pangu-3_Ccri	emb|CAKH01001309.1|:358-458
Pangu-3_Ccri	emb|CAKH01000705.1|:57164-57230
Pangu-3_Ccri	emb|CAKH01000705.1|:34840-35103
Pangu-3_Ccri	emb|CAKH01002829.1|:46090-46177
Pangu-3_Ccri	emb|CAKH01002829.1|:99528-99783
Pangu-3_Ccri	emb|CAKH01001516.1|:30723-34312
Pangu-3_Ccri	emb|CAKH01003195.1|:79612-79864

Pangu-3_Ccri	emb|CAKH01003195.1|:90292-90424
Pangu-3_Ccri	emb|CAKH01003195.1|:74991-75105
Pangu-3_Ccri	emb|CAKH01002864.1|:7334-7424
Pangu-3_Ccri	emb|CAKH01002864.1|:55671-55746
Pangu-3_Ccri	emb|CAKH01002864.1|:69696-69766
Pangu-3_Ccri	emb|CAKH01002864.1|:85579-85629
Pangu-3_Ccri	emb|CAKH01002864.1|:34543-34657
Pangu-3_Ccri	emb|CAKH01002726.1|:58199-58265
Pangu-3_Ccri	emb|CAKH01002726.1|:160022-160269
Pangu-3_Ccri	emb|CAKH01002726.1|:208812-208930
Pangu-3_Ccri	emb|CAKH01002726.1|:240997-241063
Pangu-3_Ccri	emb|CAKH01002726.1|:226912-226962
Pangu-3_Ccri	emb|CAKH01002572.1|:38977-39091
Pangu-3_Ccri	emb|CAKH01002572.1|:75051-75148
Pangu-3_Ccri	emb|CAKH01002572.1|:61077-61156
Pangu-3_Ccri	emb|CAKH01002491.1|:99315-99429
Pangu-3_Ccri	emb|CAKH01002491.1|:37795-37856
Pangu-3_Ccri	emb|CAKH01002491.1|:11846-12108
Pangu-3_Ccri	emb|CAKH01002361.1|:108246-108352
Pangu-3_Ccri	emb|CAKH01002361.1|:72821-72927
Pangu-3_Ccri	emb|CAKH01002312.1|:66009-66099
Pangu-3_Ccri	emb|CAKH01002312.1|:82380-82446
Pangu-3_Ccri	emb|CAKH01002312.1|:69204-69270
Pangu-3_Ccri	emb|CAKH01002312.1|:16738-16844
Pangu-3_Ccri	emb|CAKH01002312.1|:5085-5151
Pangu-3_Ccri	emb|CAKH01002176.1|:2323-2437
Pangu-3_Ccri	emb|CAKH01002176.1|:60-138
Pangu-3_Ccri	emb|CAKH01002085.1|:55264-55378
Pangu-3_Ccri	emb|CAKH01002037.1|:38252-38384
Pangu-3_Ccri	emb|CAKH01002037.1|:12348-17155
Pangu-3_Ccri	emb|CAKH01001872.1|:120836-120971
Pangu-3_Ccri	emb|CAKH01001872.1|:75721-75811
Pangu-3_Ccri	emb|CAKH01001838.1|:8427-8541
Pangu-3_Ccri	emb|CAKH01001838.1|:77868-77982
Pangu-3_Ccri	emb|CAKH01001838.1|:76303-76408
Pangu-3_Ccri	emb|CAKH01001838.1|:62959-63043
Pangu-3_Ccri	emb|CAKH01001764.1|:23615-23975
Pangu-3_Ccri	emb|CAKH01001764.1|:37349-37469
Pangu-3_Ccri	emb|CAKH01001764.1|:27077-27197
Pangu-3_Ccri	emb|CAKH01001745.1|:60736-60986
Pangu-3_Ccri	emb|CAKH01001745.1|:25171-25285
Pangu-3_Ccri	emb|CAKH01001616.1|:25898-25948
Pangu-3_Ccri	emb|CAKH01001616.1|:61028-61148
Pangu-3_Ccri	emb|CAKH01001616.1|:93484-93659

Pangu-3_Ccri	emb|CAKH01001616.1|:91389-91503
Pangu-3_Ccri	emb|CAKH01001536.1|:85429-85534
Pangu-3_Ccri	emb|CAKH01001536.1|:73901-74015
Pangu-3_Ccri	emb|CAKH01001536.1|:5547-5613
Pangu-3_Ccri	emb|CAKH01001504.1|:9211-9261
Pangu-3_Ccri	emb|CAKH01001504.1|:64099-64165
Pangu-3_Ccri	emb|CAKH01001504.1|:76596-76702
Pangu-3_Ccri	emb|CAKH01001504.1|:94145-94259
Pangu-3_Ccri	emb|CAKH01001504.1|:26649-26715
Pangu-3_Ccri	emb|CAKH01001504.1|:12611-12717
Pangu-3_Ccri	emb|CAKH01001501.1|:35857-35923
Pangu-3_Ccri	emb|CAKH01001501.1|:50983-51049
Pangu-3_Ccri	emb|CAKH01001501.1|:100843-100957
Pangu-3_Ccri	emb|CAKH01001501.1|:8161-8266
Pangu-3_Ccri	emb|CAKH01001226.1|:33567-33681
Pangu-3_Ccri	emb|CAKH01001088.1|:18373-18487
Pangu-3_Ccri	emb|CAKH01001088.1|:36818-36951
Pangu-3_Ccri	emb|CAKH01001084.1|:64085-64191
Pangu-3_Ccri	emb|CAKH01001084.1|:50977-51043
Pangu-3_Ccri	emb|CAKH01001084.1|:31492-31582
Pangu-3_Ccri	emb|CAKH01001033.1|:72663-72753
Pangu-3_Ccri	emb|CAKH01001033.1|:56976-57108
Pangu-3_Ccri	emb|CAKH01001033.1|:38247-38313
Pangu-3_Ccri	emb|CAKH01000885.1|:49202-49456
Pangu-3_Ccri	emb|CAKH01000885.1|:23626-23716
Pangu-3_Ccri	emb|CAKH01000837.1|:10077-10191
Pangu-3_Ccri	emb|CAKH01000676.1|:366-1960
Pangu-3_Ccri	emb|CAKH01000644.1|:22893-23007
Pangu-3_Ccri	emb|CAKH01000575.1|:27543-27657
Pangu-3_Ccri	emb|CAKH01000575.1|:62923-63016
Pangu-3_Ccri	emb|CAKH01002788.1|:106911-107165
Pangu-3_Ccri	emb|CAKH01002788.1|:131161-131416
Pangu-3_Ccri	emb|CAKH01002788.1|:4601-4667
Pangu-3_Ccri	emb|CAKH01002657.1|:77472-77719
Pangu-3_Ccri	emb|CAKH01002657.1|:44140-44350
Pangu-3_Ccri	emb|CAKH01002657.1|:32761-32827
Pangu-3_Ccri	emb|CAKH01002657.1|:20520-20586
Pangu-3_Ccri	emb|CAKH01002582.1|:65958-66078
Pangu-3_Ccri	emb|CAKH01002582.1|:80765-80885
Pangu-3_Ccri	emb|CAKH01002582.1|:40876-41011
Pangu-3_Ccri	emb|CAKH01002582.1|:25990-26245
Pangu-3_Ccri	emb|CAKH01002556.1|:5949-6038
Pangu-3_Ccri	emb|CAKH01002508.1|:82092-82252
Pangu-3_Ccri	emb|CAKH01002508.1|:139424-139529

Pangu-3_Ccri	emb|CAKH01002508.1|:9857-9953
Pangu-3_Ccri	emb|CAKH01002475.1|:70925-71180
Pangu-3_Ccri	emb|CAKH01002475.1|:83313-83568
Pangu-3_Ccri	emb|CAKH01002475.1|:59124-59256
Pangu-3_Ccri	emb|CAKH01002475.1|:46119-46206
Pangu-3_Ccri	emb|CAKH01002226.1|:49050-49305
Pangu-3_Ccri	emb|CAKH01001920.1|:15082-15337
Pangu-3_Ccri	emb|CAKH01001873.1|:73713-73968
Pangu-3_Ccri	emb|CAKH01001873.1|:112933-113053
Pangu-3_Ccri	emb|CAKH01001873.1|:100614-100734
Pangu-3_Ccri	emb|CAKH01001477.1|:290896-290962
Pangu-3_Ccri	emb|CAKH01001477.1|:210208-210463
Pangu-3_Ccri	emb|CAKH01001477.1|:184831-185085
Pangu-3_Ccri	emb|CAKH01001477.1|:102478-102544
Pangu-3_Ccri	emb|CAKH01001477.1|:90268-90334
Pangu-3_Ccri	emb|CAKH01001477.1|:33270-33367
Pangu-3_Ccri	emb|CAKH01001430.1|:29743-29831
Pangu-3_Ccri	emb|CAKH01001430.1|:16293-16548
Pangu-3_Ccri	emb|CAKH01001430.1|:3328-3583
Pangu-3_Ccri	emb|CAKH01001227.1|:11-266
Pangu-3_Ccri	emb|CAKH01001227.1|:10270-10525
Pangu-3_Ccri	emb|CAKH01001227.1|:11930-12185
Pangu-3_Ccri	emb|CAKH01000729.1|:24761-24881
Pangu-3_Ccri	emb|CAKH01000729.1|:59279-59534
Pangu-3_Ccri	emb|CAKH01000729.1|:27183-27393
Pangu-3_Ccri	emb|CAKH01000684.1|:12925-13180
Pangu-3_Ccri	emb|CAKH01000684.1|:557-812
Pangu-3_Ccri	emb|CAKH01000226.1|:19185-19274
Pangu-3_Ccri	emb|CAKH01000226.1|:151976-152081
Pangu-3_Ccri	emb|CAKH01000226.1|:139253-139319
Pangu-3_Ccri	emb|CAKH01000226.1|:56288-56338
Pangu-3_Ccri	emb|CAKH01000226.1|:26191-26401
Pangu-3_Ccri	emb|CAKH01002908.1|:19821-20076
Pangu-3_Ccri	emb|CAKH01002908.1|:97825-97930
Pangu-3_Ccri	emb|CAKH01002908.1|:93752-94006
Pangu-3_Ccri	emb|CAKH01002908.1|:80861-80995
Pangu-3_Ccri	emb|CAKH01002908.1|:56417-56513
Pangu-3_Ccri	emb|CAKH01002908.1|:26840-27095
Pangu-3_Ccri	emb|CAKH01002695.1|:107771-107887
Pangu-3_Ccri	emb|CAKH01000015.1|:9568-9656
Pangu-3_Ccri	emb|CAKH01001152.1|:14430-14553
Pangu-3_Ccri	emb|CAKH01001152.1|:26461-26559
Pangu-3_Ccri	emb|CAKH01000502.1|:38151-38246
Pangu-3_Ccri	emb|CAKH01003221.1|:113994-114084

Pangu-3_Ccri	emb|CAKH01003221.1|:145159-145225
Pangu-3_Ccri	emb|CAKH01003221.1|:96202-96322
Pangu-3_Ccri	emb|CAKH01003221.1|:83881-84001
Pangu-3_Ccri	emb|CAKH01003081.1|:1524-1734
Pangu-3_Ccri	emb|CAKH01003081.1|:15210-15420
Pangu-3_Ccri	emb|CAKH01002915.1|:2196-2301
Pangu-3_Ccri	emb|CAKH01002915.1|:6068-6158
Pangu-3_Ccri	emb|CAKH01002733.1|:41159-41294
Pangu-3_Ccri	emb|CAKH01002733.1|:26571-26706
Pangu-3_Ccri	emb|CAKH01002535.1|:96844-97278
Pangu-3_Ccri	emb|CAKH01002462.1|:51307-51562
Pangu-3_Ccri	emb|CAKH01002462.1|:35396-35510
Pangu-3_Ccri	emb|CAKH01002462.1|:17002-17107
Pangu-3_Ccri	emb|CAKH01002462.1|:4452-4557
Pangu-3_Ccri	emb|CAKH01002347.1|:22485-22599
Pangu-3_Ccri	emb|CAKH01002275.1|:50230-50336
Pangu-3_Ccri	emb|CAKH01002234.1|:11072-11158
Pangu-3_Ccri	emb|CAKH01002145.1|:12128-12226
Pangu-3_Ccri	emb|CAKH01002145.1|:76961-77051
Pangu-3_Ccri	emb|CAKH01002145.1|:64078-64168
Pangu-3_Ccri	emb|CAKH01002077.1|:65371-65626
Pangu-3_Ccri	emb|CAKH01002074.1|:84180-84300
Pangu-3_Ccri	emb|CAKH01002074.1|:22766-22856
Pangu-3_Ccri	emb|CAKH01002074.1|:2483-2567
Pangu-3_Ccri	emb|CAKH01002005.1|:18787-18853
Pangu-3_Ccri	emb|CAKH01002005.1|:45417-45537
Pangu-3_Ccri	emb|CAKH01002005.1|:16753-17004
Pangu-3_Ccri	emb|CAKH01001866.1|:68380-68458
Pangu-3_Ccri	emb|CAKH01001866.1|:128282-128530
Pangu-3_Ccri	emb|CAKH01001866.1|:43770-43876
Pangu-3_Ccri	emb|CAKH01001854.1|:40766-40872
Pangu-3_Ccri	emb|CAKH01001854.1|:49644-49710
Pangu-3_Ccri	emb|CAKH01001854.1|:19031-19121
Pangu-3_Ccri	emb|CAKH01001771.1|:29291-29546
Pangu-3_Ccri	emb|CAKH01001771.1|:31723-31933
Pangu-3_Ccri	emb|CAKH01001729.1|:20263-20377
Pangu-3_Ccri	emb|CAKH01001428.1|:91174-91288
Pangu-3_Ccri	emb|CAKH01001379.1|:36747-36837
Pangu-3_Ccri	emb|CAKH01001365.1|:48239-48353
Pangu-3_Ccri	emb|CAKH01001365.1|:125960-126050
Pangu-3_Ccri	emb|CAKH01001365.1|:109668-109878
Pangu-3_Ccri	emb|CAKH01001365.1|:97046-97145
Pangu-3_Ccri	emb|CAKH01001365.1|:56292-56380
Pangu-3_Ccri	emb|CAKH01001338.1|:43103-43358

Pangu-3_Ccri	emb|CAKH01001338.1|:6976-7066
Pangu-3_Ccri	emb|CAKH01001114.1|:44947-45053
Pangu-3_Ccri	emb|CAKH01001114.1|:67089-67351
Pangu-3_Ccri	emb|CAKH01001010.1|:164360-164464
Pangu-3_Ccri	emb|CAKH01001010.1|:173218-173284
Pangu-3_Ccri	emb|CAKH01001010.1|:1755-1869
Pangu-3_Ccri	emb|CAKH01000928.1|:28042-28156
Pangu-3_Ccri	emb|CAKH01000864.1|:25025-25085
Pangu-3_Ccri	emb|CAKH01000864.1|:29176-29266
Pangu-3_Ccri	emb|CAKH01000721.1|:23269-23359
Pangu-3_Ccri	emb|CAKH01000721.1|:34685-34775
Pangu-3_Ccri	emb|CAKH01000594.1|:14284-14398
Pangu-3_Ccri	emb|CAKH01000500.1|:98694-98935
Pangu-3_Ccri	emb|CAKH01000500.1|:109095-109158
Pangu-3_Ccri	emb|CAKH01000500.1|:48561-48651
Pangu-3_Ccri	emb|CAKH01000500.1|:34634-34724
Pangu-3_Ccri	emb|CAKH01000387.1|:51370-51476
Pangu-3_Ccri	emb|CAKH01000387.1|:37633-37845
Pangu-3_Ccri	emb|CAKH01000387.1|:26099-26354
Pangu-3_Ccri	emb|CAKH01000304.1|:704-794
Pangu-3_Ccri	emb|CAKH01000148.1|:151818-151908
Pangu-3_Ccri	emb|CAKH01000148.1|:138069-138153
Pangu-3_Ccri	emb|CAKH01000148.1|:102432-102537
Pangu-3_Ccri	emb|CAKH01000148.1|:89209-89314
Pangu-3_Ccri	emb|CAKH01000148.1|:59279-59376
Pangu-3_Ccri	emb|CAKH01002814.1|:59050-59305
Pangu-3_Ccri	emb|CAKH01002707.1|:73464-73714
Pangu-3_Ccri	emb|CAKH01002707.1|:114538-114604
Pangu-3_Ccri	emb|CAKH01002673.1|:23495-24227
Pangu-3_Ccri	emb|CAKH01002670.1|:105210-105460
Pangu-3_Ccri	emb|CAKH01002599.1|:75517-75772
Pangu-3_Ccri	emb|CAKH01002599.1|:62492-62747
Pangu-3_Ccri	emb|CAKH01002181.1|:23815-23912
Pangu-3_Ccri	emb|CAKH01002081.1|:25556-25818
Pangu-3_Ccri	emb|CAKH01001902.1|:96880-97135
Pangu-3_Ccri	emb|CAKH01001902.1|:44721-44787
Pangu-3_Ccri	emb|CAKH01001902.1|:32215-32281
Pangu-3_Ccri	emb|CAKH01001255.1|:48231-48336
Pangu-3_Ccri	emb|CAKH01001255.1|:17081-17186
Pangu-3_Ccri	emb|CAKH01001238.1|:12988-13243
Pangu-3_Ccri	emb|CAKH01000887.1|:23706-23961
Pangu-3_Ccri	emb|CAKH01000491.1|:3046-3301
Pangu-3_Ccri	emb|CAKH01000370.1|:55584-55839
Pangu-3_Ccri	emb|CAKH01000370.1|:42182-42437

Pangu-3_Ccri	emb|CAKH01002809.1|:13252-13340
Pangu-3_Ccri	emb|CAKH01002431.1|:63521-63768
Pangu-3_Ccri	emb|CAKH01002431.1|:39672-39882
Pangu-3_Ccri	emb|CAKH01001582.1|:27039-27126
Pangu-3_Ccri	emb|CAKH01001582.1|:33202-33457
Pangu-3_Ccri	emb|CAKH01000872.1|:9008-9124
Pangu-3_Ccri	emb|CAKH01002646.1|:66324-66760
Pangu-3_Ccri	emb|CAKH01002646.1|:21465-21585
Pangu-3_Ccri	emb|CAKH01001952.1|:62361-62609
Pangu-3_Ccri	emb|CAKH01001952.1|:49878-50126
Pangu-3_Ccri	emb|CAKH01001952.1|:23321-23453
Pangu-3_Ccri	emb|CAKH01001952.1|:9102-9234
Pangu-3_Ccri	emb|CAKH01000948.1|:79724-79774
Pangu-3_Ccri	emb|CAKH01000948.1|:42102-42336
Pangu-3_Ccri	emb|CAKH01000386.1|:15157-15223
Pangu-3_Ccri	emb|CAKH01000386.1|:52641-52738
Pangu-3_Ccri	emb|CAKH01000386.1|:101484-101571
Pangu-3_Ccri	emb|CAKH01003207.1|:7036-7156
Pangu-3_Ccri	emb|CAKH01003207.1|:20222-20342
Pangu-3_Ccri	emb|CAKH01003207.1|:26804-26861
Pangu-4_Ccri	emb|CAKH01000881.1|:65970-69192
Pangu-4_Ccri	emb|CAKH01000766.1|:32644-35869
Pangu-4_Ccri	emb|CAKH01000682.1|:28956-32174
Pangu-4_Ccri	emb|CAKH01000846.1|:3619-4972
Pangu-4_Ccri	emb|CAKH01002920.1|:9942-12497
Pangu-4_Ccri	emb|CAKH01001531.1|:3105-4549
Pangu-4_Ccri	emb|CAKH01002208.1|:60930-62509
Pangu-4_Ccri	emb|CAKH01000579.1|:77176-78514
Pangu-4_Ccri	emb|CAKH01002430.1|:13402-14820
Pangu-4_Ccri	emb|CAKH01001187.1|:39766-41078
Pangu-4_Ccri	emb|CAKH01001187.1|:39349-41074
Pangu-4_Ccri	emb|CAKH01000727.1|:130015-131080
Pangu-4_Ccri	emb|CAKH01001126.1|:2465-3530
Pangu-4_Ccri	emb|CAKH01000726.1|:27873-28938
Pangu-5_Ccri	emb|CAKH01001315.1|:125197-127820
Pangu-5_Ccri	emb|CAKH01002833.1|:1998-4624
Pangu-5_Ccri	emb|CAKH01002177.1|:38874-41408
Pangu-5_Ccri	emb|CAKH01000948.1|:87929-90432
Pangu-5_Ccri	emb|CAKH01001129.1|:5392-7505
Pangu-5_Ccri	emb|CAKH01002005.1|:7310-11548
Pangu-5_Ccri	emb|CAKH01002998.1|:9292-12662
Pangu-5_Ccri	emb|CAKH01002678.1|:42586-42949
Pangu-5_Ccri	emb|CAKH01001200.1|:101718-102301
Pangu-5_Ccri	emb|CAKH01001200.1|:38855-39368

Pangu-5_Ccri	emb|CAKH01002199.1|:2-271
Pangu-5_Ccri	emb|CAKH01001610.1|:143022-143096
Pangu-5_Ccri	emb|CAKH01001610.1|:66465-68888
Pangu-5_Ccri	emb|CAKH01001610.1|:52978-53075
Pangu-5_Ccri	emb|CAKH01000543.1|:4814-5120
Pangu-5_Ccri	emb|CAKH01000485.1|:36088-36769
Pangu-5_Ccri	emb|CAKH01002705.1|:71278-71537
Pangu-5_Ccri	emb|CAKH01001227.1|:38567-38736
Pangu-5_Ccri	emb|CAKH01001434.1|:564-748
Pangu-5_Ccri	emb|CAKH01001571.1|:9403-10158
Pangu-5_Ccri	emb|CAKH01001950.1|:46050-49260
Pangu-5_Ccri	emb|CAKH01001904.1|:26973-29666
Pangu-5_Ccri	emb|CAKH01001003.1|:3499-3703
Pangu-5_Ccri	emb|CAKH01002523.1|:31508-34945
Pangu-5_Ccri	emb|CAKH01002491.1|:9077-9175
Pangu-5_Ccri	emb|CAKH01002093.1|:30374-30550
Pangu-5_Ccri	emb|CAKH01000015.1|:3379-4018
Pangu-5_Ccri	emb|CAKH01001967.1|:21826-22541
Pangu-5_Ccri	emb|CAKH01001735.1|:15235-15429
Pangu-5_Ccri	emb|CAKH01001735.1|:15199-15248
Pangu-5_Ccri	emb|CAKH01002416.1|:4282-4363
Pangu-5_Ccri	emb|CAKH01001894.1|:659-711
Pangu-5_Ccri	emb|CAKH01003074.1|:49298-50744
Pangu-5_Ccri	emb|CAKH01002275.1|:36818-38262
Pangu-5_Ccri	emb|CAKH01002048.1|:65217-65284
Pangu-5_Ccri	emb|CAKH01001647.1|:4914-6357
Pangu-5_Ccri	emb|CAKH01000965.1|:10949-11036
Pangu-5_Ccri	emb|CAKH01000320.1|:196-1639
Pangu-5_Ccri	emb|CAKH01003139.1|:79507-80670
Pangu-5_Ccri	emb|CAKH01002613.1|:38342-41119
Pangu-5_Ccri	emb|CAKH01000862.1|:85635-85721
Pangu-5_Ccri	emb|CAKH01002441.1|:44589-44803
Pangu-5_Ccri	emb|CAKH01001365.1|:20453-21895
Pangu-5_Ccri	emb|CAKH01002218.1|:17891-17943
Pangu-5_Ccri	emb|CAKH01000133.1|:8381-8445
Pangu-5_Ccri	emb|CAKH01002198.1|:209388-209860
Pangu-5_Ccri	emb|CAKH01001552.1|:50219-50604
Pangu-5_Ccri	emb|CAKH01002739.1|:105015-105089
Pangu-6_Ccri	emb|CAKH01001964.1|:14179-16701
Pangu-6_Ccri	emb|CAKH01001669.1|:37120-39642
Pangu-6_Ccri	emb|CAKH01000160.1|:11938-14460
Pangu-6_Ccri	emb|CAKH01001718.1|:3399-5921
Pangu-6_Ccri	emb|CAKH01000234.1|:3874-6397
Pangu-6_Ccri	emb|CAKH01002002.1|:15510-18032

Pangu-6_Ccri	emb|CAKH01001448.1|:13571-16097
Pangu-6_Ccri	emb|CAKH01002805.1|:115979-116897
Pangu-6_Ccri	emb|CAKH01002805.1|:365294-367895
Pangu-6_Ccri	emb|CAKH01002613.1|:42571-45169
Pangu-6_Ccri	emb|CAKH01001812.1|:56401-57318
Pangu-6_Ccri	emb|CAKH01000793.1|:104391-105311
Pangu-6_Ccri	emb|CAKH01001342.1|:26249-27959
Pangu-6_Ccri	emb|CAKH01003102.1|:31132-35806
Pangu-6_Ccri	emb|CAKH01002441.1|:45910-45990
Pangu-6_Ccri	emb|CAKH01000485.1|:36898-36978
Pangu-6_Ccri	emb|CAKH01001200.1|:39494-39577
Pangu-6_Ccri	emb|CAKH01003147.1|:2947-3021
Pangu-7_Ccri	emb|CAKH01002905.1|:52883-55599
Pangu-7_Ccri	emb|CAKH01002806.1|:175-2887
Pangu-7_Ccri	emb|CAKH01002499.1|:198425-201113
Pangu-7_Ccri	emb|CAKH01001745.1|:57951-59359
Pangu-7_Ccri	emb|CAKH01001084.1|:49166-50528
Pangu-7_Ccri	emb|CAKH01001104.1|:18553-18701
Pangu-8_Ccri	emb|CAKH01002234.1|:35239-37844
Pangu-8_Ccri	emb|CAKH01001256.1|:172552-175173
Pangu-8_Ccri	emb|CAKH01000663.1|:227733-230354
Pangu-8_Ccri	emb|CAKH01000579.1|:1856-4484
Pangu-8_Ccri	emb|CAKH01000173.1|:11675-14298
Pangu-8_Ccri	emb|CAKH01002078.1|:160961-163582
Pangu-8_Ccri	emb|CAKH01000896.1|:5996-6199
Pangu-8_Ccri	emb|CAKH01000896.1|:34470-34574
Pangu-8_Ccri	emb|CAKH01000894.1|:7174-7265
Pangu-8_Ccri	emb|CAKH01001772.1|:28971-29020
Pangu-9_Ccri	emb|CAKH01002322.1|:19322-23168
Pangu-9_Ccri	emb|CAKH01002655.1|:15927-19779
Pangu-9_Ccri	emb|CAKH01001505.1|:158253-162104
Pangu-9_Ccri	emb|CAKH01001372.1|:4822-8666
Pangu-10_Ccri	emb|CAKH01002595.1|:64344-67129
Pangu-10_Ccri	emb|CAKH01002655.1|:2527-5313
Pangu-10_Ccri	emb|CAKH01000698.1|:14734-17519
Pangu-10_Ccri	emb|CAKH01001955.1|:1952-4738
Pangu-10_Ccri	emb|CAKH01001663.1|:60134-62918
Pangu-10_Ccri	emb|CAKH01001221.1|:1108-3897
Pangu-10_Ccri	emb|CAKH01002899.1|:73810-77364
Pangu-10_Ccri	emb|CAKH01002795.1|:11850-11921
Pangu-10_Ccri	emb|CAKH01001727.1|:46279-46350
Pangu-10_Ccri	emb|CAKH01001453.1|:27723-27794
Pangu-10_Ccri	emb|CAKH01002048.1|:12560-12631
Pangu-10_Ccri	emb|CAKH01001018.1|:4488-4559

Pangu-10_Ccri	emb|CAKH01002731.1|:12776-12841
Pangu-10_Ccri	emb|CAKH01002910.1|:72101-72177
Pangu-11_Ccri	emb|CAKH01001453.1|:26808-31885
Pangu-11_Ccri	emb|CAKH01002048.1|:11648-16691
Pangu-11_Ccri	emb|CAKH01002795.1|:7758-12833
Pangu-11_Ccri	emb|CAKH01001018.1|:464-5459
Pangu-11_Ccri	emb|CAKH01001727.1|:40452-47262
Pangu-11_Ccri	emb|CAKH01002881.1|:19175-22547
Pangu-11_Ccri	emb|CAKH01002221.1|:4523-7908
Pangu-11_Ccri	emb|CAKH01002739.1|:106585-107160
Pangu-11_Ccri	emb|CAKH01002504.1|:31563-32138
Pangu-11_Ccri	emb|CAKH01002504.1|:46644-47187
Pangu-11_Ccri	emb|CAKH01002009.1|:194498-195041
Pangu-11_Ccri	emb|CAKH01001875.1|:5088-5663
Pangu-11_Ccri	emb|CAKH01001787.1|:52051-52569
Pangu-11_Ccri	emb|CAKH01001200.1|:16862-17437
Pangu-11_Ccri	emb|CAKH01000524.1|:7591-8134
Pangu-11_Ccri	emb|CAKH01000501.1|:40995-41538
Pangu-11_Ccri	emb|CAKH01000440.1|:10333-10909
Pangu-11_Ccri	emb|CAKH01000374.1|:3542-13493
Pangu-11_Ccri	emb|CAKH01000047.1|:34416-34959
Pangu-11_Ccri	emb|CAKH01002816.1|:6305-6880
Pangu-11_Ccri	emb|CAKH01002424.1|:47849-48391
Pangu-11_Ccri	emb|CAKH01002198.1|:29681-30256
Pangu-11_Ccri	emb|CAKH01001963.1|:2817-3392
Pangu-11_Ccri	emb|CAKH01001537.1|:13776-14350
Pangu-11_Ccri	emb|CAKH01000993.1|:8757-9147
Pangu-11_Ccri	emb|CAKH01003193.1|:65400-65920
Pangu-11_Ccri	emb|CAKH01002678.1|:85050-85629
Pangu-11_Ccri	emb|CAKH01001853.1|:40407-40950
Pangu-11_Ccri	emb|CAKH01001406.1|:68556-69131
Pangu-11_Ccri	emb|CAKH01000632.1|:61235-61753
Pangu-11_Ccri	emb|CAKH01001302.1|:14597-15144
Pangu-11_Ccri	emb|CAKH01000663.1|:211479-213621
Pangu-11_Ccri	emb|CAKH01002576.1|:65951-66524
Pangu-11_Ccri	emb|CAKH01001842.1|:5439-5884
Pangu-11_Ccri	emb|CAKH01000810.1|:41946-42395
Pangu-11_Ccri	emb|CAKH01000301.1|:71073-71616
Pangu-11_Ccri	emb|CAKH01001902.1|:8745-9067
Pangu-11_Ccri	emb|CAKH01002905.1|:40736-40982
Pangu-11_Ccri	emb|CAKH01003199.1|:80662-81191
Pangu-11_Ccri	emb|CAKH01002629.1|:12609-13081
Pangu-11_Ccri	emb|CAKH01002439.1|:14834-16483
Pangu-11_Ccri	emb|CAKH01001940.1|:54687-55339

Pangu-11_Ccri	emb|CAKH01001407.1|:6147-9517
Pangu-11_Ccri	emb|CAKH01002316.1|:7396-12331
Pangu-11_Ccri	emb|CAKH01003035.1|:85-9478
Pangu-11_Ccri	emb|CAKH01000333.1|:75437-76536
Pangu-11_Ccri	emb|CAKH01002899.1|:75607-75678
Pangu-11_Ccri	emb|CAKH01002899.1|:57122-57524
Pangu-11_Ccri	emb|CAKH01002528.1|:78411-78830
Pangu-11_Ccri	emb|CAKH01001800.1|:66684-67237
Pangu-11_Ccri	emb|CAKH01003036.1|:38235-38573
Pangu-11_Ccri	emb|CAKH01000225.1|:27442-27800
Pangu-11_Ccri	emb|CAKH01000225.1|:14054-14205
Pangu-11_Ccri	emb|CAKH01000689.1|:29248-32114
Pangu-11_Ccri	emb|CAKH01002176.1|:19999-20423
Pangu-11_Ccri	emb|CAKH01002078.1|:33745-33918
Pangu-11_Ccri	emb|CAKH01000938.1|:77193-77530
Pangu-11_Ccri	emb|CAKH01002914.1|:332-552
Pangu-11_Ccri	emb|CAKH01002913.1|:1453-1674
Pangu-11_Ccri	emb|CAKH01002054.1|:41909-42516
Pangu-11_Ccri	emb|CAKH01003217.1|:101897-102067
Pangu-11_Ccri	emb|CAKH01002912.1|:205-366
Pangu-11_Ccri	emb|CAKH01002731.1|:14775-16334
Pangu-11_Ccri	emb|CAKH01001645.1|:14138-14459
Pangu-11_Ccri	emb|CAKH01002335.1|:3432-3806
Pangu-11_Ccri	emb|CAKH01001490.1|:6445-8008
Pangu-11_Ccri	emb|CAKH01002627.1|:12827-13047
Pangu-11_Ccri	emb|CAKH01002826.1|:31643-32048
Pangu-11_Ccri	emb|CAKH01002716.1|:63968-64116
Pangu-11_Ccri	emb|CAKH01001970.1|:80088-80236
Pangu-11_Ccri	emb|CAKH01001658.1|:96387-96823
Pangu-11_Ccri	emb|CAKH01000057.1|:5890-6434
Pangu-11_Ccri	emb|CAKH01003139.1|:87773-90072
Pangu-11_Ccri	emb|CAKH01002172.1|:122419-129373
Pangu-11_Ccri	emb|CAKH01002915.1|:24817-24973
Pangu-11_Ccri	emb|CAKH01001178.1|:15308-15413
Pangu-11_Ccri	emb|CAKH01000556.1|:42326-42431
Pangu-11_Ccri	emb|CAKH01002895.1|:3119-3397
Pangu-11_Ccri	emb|CAKH01002093.1|:29318-29406
Pangu-11_Ccri	emb|CAKH01001610.1|:135505-135660
Pangu-11_Ccri	emb|CAKH01001483.1|:9642-9765
Pangu-11_Ccri	emb|CAKH01002829.1|:108123-108194
Pangu-11_Ccri	emb|CAKH01002829.1|:92532-93281
Pangu-11_Ccri	emb|CAKH01002778.1|:5557-5628
Pangu-11_Ccri	emb|CAKH01002655.1|:4323-4394
Pangu-11_Ccri	emb|CAKH01002595.1|:65263-65334

Pangu-11_Ccri	emb|CAKH01001955.1|:2871-2942
Pangu-11_Ccri	emb|CAKH01001487.1|:36503-36574
Pangu-11_Ccri	emb|CAKH01001706.1|:27792-27963
Pangu-11_Ccri	emb|CAKH01001663.1|:61928-61999
Pangu-11_Ccri	emb|CAKH01001221.1|:2907-2978
Pangu-11_Ccri	emb|CAKH01002431.1|:73573-73763
Pangu-11_Ccri	emb|CAKH01002781.1|:43438-43571
Pangu-11_Ccri	emb|CAKH01002045.1|:58226-58359
Pangu-11_Ccri	emb|CAKH01002647.1|:101715-101827
Pangu-11_Ccri	emb|CAKH01002788.1|:79311-79453
Pangu-11_Ccri	emb|CAKH01000806.1|:22460-22626
Pangu-11_Ccri	emb|CAKH01000127.1|:34198-34334
Pangu-11_Ccri	emb|CAKH01002808.1|:3859-3990
Pangu-11_Ccri	emb|CAKH01001118.1|:188685-188816
Pangu-11_Ccri	emb|CAKH01000698.1|:16530-16600
Pangu-11_Ccri	emb|CAKH01000387.1|:47743-49662
Pangu-11_Ccri	emb|CAKH01002537.1|:91115-91165
Pangu-11_Ccri	emb|CAKH01001881.1|:2564-2617
Pangu-12_Ccri	emb|CAKH01002705.1|:54304-57193
Pangu-12_Ccri	emb|CAKH01002262.1|:72566-75455
Pangu-12_Ccri	emb|CAKH01002470.1|:60374-63246
Pangu-12_Ccri	emb|CAKH01000607.1|:58780-60080
Pangu-12_Ccri	emb|CAKH01003044.1|:3255-4445
Pangu-12_Ccri	emb|CAKH01003044.1|:19380-20878
Pangu-12_Ccri	emb|CAKH01002704.1|:17571-18724
Pangu-12_Ccri	emb|CAKH01002520.1|:4213-4376
Pangu-12_Ccri	emb|CAKH01000157.1|:82784-83336
Pangu-13_Ccri	emb|CAKH01001267.1|:62285-68650
Pangu-13_Ccri	emb|CAKH01001612.1|:102637-108993
Pangu-13_Ccri	emb|CAKH01001453.1|:26682-38127
Pangu-13_Ccri	emb|CAKH01002576.1|:78099-85488
Pangu-13_Ccri	emb|CAKH01001908.1|:64869-70888
Pangu-13_Ccri	emb|CAKH01001954.1|:107036-113060
Pangu-13_Ccri	emb|CAKH01002062.1|:92655-94841
Pangu-13_Ccri	emb|CAKH01002423.1|:42082-45303
Pangu-13_Ccri	emb|CAKH01002226.1|:6396-12261
Pangu-13_Ccri	emb|CAKH01003189.1|:12936-17738
Pangu-13_Ccri	emb|CAKH01003221.1|:320-1093
Pangu-13_Ccri	emb|CAKH01002180.1|:53600-54374
Pangu-13_Ccri	emb|CAKH01002877.1|:2223-2998
Pangu-13_Ccri	emb|CAKH01002766.1|:17837-18431
Pangu-13_Ccri	emb|CAKH01001407.1|:9561-10155
Pangu-13_Ccri	emb|CAKH01002788.1|:137279-137873
Pangu-13_Ccri	emb|CAKH01002499.1|:7243-7844

Pangu-13_Ccri	emb|CAKH01002499.1|:193164-193758
Pangu-13_Ccri	emb|CAKH01002499.1|:164202-164795
Pangu-13_Ccri	emb|CAKH01002291.1|:31967-32561
Pangu-13_Ccri	emb|CAKH01001669.1|:45084-45678
Pangu-13_Ccri	emb|CAKH01001114.1|:30128-30722
Pangu-13_Ccri	emb|CAKH01001100.1|:212785-212867
Pangu-13_Ccri	emb|CAKH01001100.1|:212867-213466
Pangu-13_Ccri	emb|CAKH01000301.1|:71648-72411
Pangu-13_Ccri	emb|CAKH01003126.1|:11599-12193
Pangu-13_Ccri	emb|CAKH01001913.1|:202323-203786
Pangu-13_Ccri	emb|CAKH01001913.1|:203793-204387
Pangu-13_Ccri	emb|CAKH01001385.1|:27392-27986
Pangu-13_Ccri	emb|CAKH01001151.1|:42010-42605
Pangu-13_Ccri	emb|CAKH01002171.1|:419-1015
Pangu-13_Ccri	emb|CAKH01003161.1|:88658-89252
Pangu-13_Ccri	emb|CAKH01000431.1|:22906-23507
Pangu-13_Ccri	emb|CAKH01001827.1|:7356-7957
Pangu-13_Ccri	emb|CAKH01002819.1|:29-379
Pangu-13_Ccri	emb|CAKH01001787.1|:18587-18925
Pangu-13_Ccri	emb|CAKH01000990.1|:976-1453
Pangu-13_Ccri	emb|CAKH01000117.1|:47378-50389
Pangu-13_Ccri	emb|CAKH01001317.1|:101682-101793
Pangu-13_Ccri	emb|CAKH01001317.1|:86621-86778
Pangu-13_Ccri	emb|CAKH01000506.1|:10780-11769
Pangu-13_Ccri	emb|CAKH01000317.1|:29293-30287
Pangu-13_Ccri	emb|CAKH01001302.1|:37696-37807
Pangu-13_Ccri	emb|CAKH01001953.1|:11102-11652
Pangu-13_Ccri	emb|CAKH01000984.1|:17346-17508
Pangu-13_Ccri	emb|CAKH01001379.1|:52807-52875
Pangu-13_Ccri	emb|CAKH01001537.1|:14387-14824
Pangu-13_Ccri	emb|CAKH01002892.1|:16107-16175
Pangu-13_Ccri	emb|CAKH01002137.1|:2777-6462
Pangu-14_Ccri	emb|CAKH01002205.1|:90536-93701
Pangu-14_Ccri	emb|CAKH01002605.1|:4555-7698
Pangu-14_Ccri	emb|CAKH01001841.1|:74266-78470
Pangu-14_Ccri	emb|CAKH01001397.1|:22792-24361
Pangu-14_Ccri	emb|CAKH01002296.1|:39474-43442
Pangu-14_Ccri	emb|CAKH01003125.1|:14834-16329
Pangu-14_Ccri	emb|CAKH01003136.1|:3823-3909
Pangu-14_Ccri	emb|CAKH01002731.1|:12698-12813
Pangu-14_Ccri	emb|CAKH01001453.1|:27642-27789
Pangu-14_Ccri	emb|CAKH01002572.1|:9913-9965
Pangu-15_Ccri	emb|CAKH01002829.1|:108991-112143
Pangu-15_Ccri	emb|CAKH01002075.1|:42605-45757

	Pangu-15_Ccri	emb|CAKH01001408.1|:21929-25080
	Pangu-15_Ccri	emb|CAKH01001952.1|:1-3147
	Pangu-15_Ccri	emb|CAKH01002669.1|:17603-20754
	Pangu-15_Ccri	emb|CAKH01002953.1|:9062-11641
	Pangu-15_Ccri	emb|CAKH01001033.1|:76511-79649
	Pangu-15_Ccri	emb|CAKH01002482.1|:10915-12750
	Pangu-15_Ccri	emb|CAKH01002481.1|:18-1318
	Pangu-15_Ccri	emb|CAKH01001547.1|:90830-91742
	Pangu-15_Ccri	emb|CAKH01001824.1|:1-285
Crassostrea gigas	Pangu_CGig	gb|AFTI01006564.1|:15754-20085
	Pangu_CGig	gb|AFTI01003402.1|:31782-35394
	Pangu_CGig	gb|AFTI01016308.1|:9557-14587
	Pangu_CGig	gb|AFTI01018658.1|:16762-21107
	Pangu_CGig	gb|AFTI01007612.1|:6244-9437
	Pangu_CGig	gb|AFTI01024346.1|:1291-6527
	Pangu_CGig	gb|AFTI01016181.1|:73174-74907
	Pangu_CGig	gb|AFTI01016182.1|:2614-4991
	Pangu_CGig	gb|AFTI01010614.1|:7540-8621
	Pangu_CGig	gb|AFTI01009625.1|:37914-38922
	Pangu_CGig	gb|AFTI01007996.1|:1479-2500
	Pangu_CGig	gb|AFTI01022375.1|:24489-25524
	Pangu_CGig	gb|AFTI01011470.1|:180-1625
	Pangu_CGig	gb|AFTI01010515.1|:33514-34547
	Pangu_CGig	gb|AFTI01020138.1|:24234-25269
	Pangu_CGig	gb|AFTI01015101.1|:14861-15921
	Pangu_CGig	gb|AFTI01000321.1|:24017-25081
	Pangu_CGig	gb|AFTI01030290.1|:29033-29965
	Pangu_CGig	gb|AFTI01024274.1|:393-2270
	Pangu_CGig	gb|AFTI01029869.1|:22516-23568
	Pangu_CGig	gb|AFTI01022612.1|:2914-3932
	Pangu_CGig	gb|AFTI01019640.1|:3438-4462
	Pangu_CGig	gb|AFTI01001708.1|:841-1854
	Pangu_CGig	gb|AFTI01015489.1|:7484-8565
	Pangu_CGig	gb|AFTI01011591.1|:7486-8451
	Pangu_CGig	gb|AFTI01014693.1|:39966-40947
	Pangu_CGig	gb|AFTI01007611.1|:9347-9546
	Pangu_CGig	gb|AFTI01004284.1|:12283-12478
	Pangu_CGig	gb|AFTI01014949.1|:19497-19676
	Pangu_CGig	gb|AFTI01008709.1|:77-252
	Pangu_CGig	gb|AFTI01004285.1|:32-193
	Pangu_CGig	gb|AFTI01000069.1|:1411-1569
	Pangu_CGig	gb|AFTI01017392.1|:2154-2266
	Pangu_CGig	gb|AFTI01024345.1|:8898-8983
	Pangu_CGig	gb|AFTI01010715.1|:11281-11359

	Pangu_CGig	gb|AFTI01003123.1|:28688-28762
	Pangu_CGig	gb|AFTI01021439.1|:10654-10708
	Pangu_CGig	gb|AFTI01015857.1|:9159-9209
	Pangu_CGig	gb|AFTI01010716.1|:74-124
	Pangu_CGig	gb|AFTI01007277.1|:7483-11020
	Pangu_CGig	gb|AFTI01005623.1|:6052-8033
	Pangu_CGig	gb|AFTI01023953.1|:14523-15268
	Pangu_CGig	gb|AFTI01017510.1|:5210-5982
	Pangu_CGig	gb|AFTI01022963.1|:15473-16539
	Pangu_CGig	gb|AFTI01006949.1|:3309-4370
	Pangu_CGig	gb|AFTI01029886.1|:73267-74328
	Pangu_CGig	gb|AFTI01024301.1|:3661-4710
	Pangu_CGig	gb|AFTI01005742.1|:97482-98730
	Pangu_CGig	gb|AFTI01008679.1|:1115-2168
	Pangu_CGig	gb|AFTI01020858.1|:19414-20317
	Pangu_CGig	gb|AFTI01016709.1|:60128-62306
	Pangu_CGig	gb|AFTI01012802.1|:3633-4623
	Pangu_CGig	gb|AFTI01002922.1|:43137-44126
	Pangu_CGig	gb|AFTI01001525.1|:10584-12070
	Pangu_CGig	gb|AFTI01017131.1|:6040-6871
	Pangu_CGig	gb|AFTI01005493.1|:47518-48428
	Pangu_CGig	gb|AFTI01016981.1|:5021-6039
	Pangu_CGig	gb|AFTI01021185.1|:68680-69523
	Pangu_CGig	gb|AFTI01004331.1|:1380-2677
	Pangu_CGig	gb|AFTI01007015.1|:74332-75213
	Pangu_CGig	gb|AFTI01023266.1|:2185-3188
	Pangu_CGig	gb|AFTI01019812.1|:3281-3478
	Pangu_CGig	gb|AFTI01024934.1|:36815-37008
	Pangu_CGig	gb|AFTI01025303.1|:2540-2722
	Pangu_CGig	gb|AFTI01009369.1|:3993-4169
	Pangu_CGig	gb|AFTI01026516.1|:148-316
	Pangu_CGig	gb|AFTI01019811.1|:11167-11335
	Pangu_CGig	gb|AFTI01001485.1|:31395-31469
	Pangu_CGig	gb|AFTI01004957.1|:16140-16210
	Pangu_CGig	gb|AFTI01012867.1|:28785-28844
	Pangu_CGig	gb|AFTI01011885.1|:7407-7457
Ectocarpus siliculosus	Pangu_ESil	emb|CABU01009199.1|:21065-28930
	Pangu_ESil	emb|CABU01009323.1|:50217-57998
	Pangu_ESil	emb|CABU01010940.1|:6557-12973
	Pangu_ESil	emb|CABU01007662.1|:12183-14518
	Pangu_ESil	emb|CABU01010525.1|:1059-2147
	Pangu_ESil	emb|CABU01011393.1|:1-159
	Pangu_ESil	emb|CABU01012007.1|:39549-39696
	Pangu_ESil	emb|CABU01007344.1|:6081-6228

	Pangu_ESil	emb|CABU01005014.1|:51018-51165
	Pangu_ESil	emb|CABU01002763.1|:5537-12263
	Pangu_ESil	emb|CABU01005798.1|:25085-27339
	Pangu_ESil	emb|CABU01012557.1|:20116-20263
	Pangu_ESil	emb|CABU01012362.1|:1346-1493
	Pangu_ESil	emb|CABU01012230.1|:10669-10816
	Pangu_ESil	emb|CABU01008887.1|:5992-6139
	Pangu_ESil	emb|CABU01010651.1|:41622-41769
	Pangu_ESil	emb|CABU01008023.1|:115812-115959
	Pangu_ESil	emb|CABU01007907.1|:19388-19535
	Pangu_ESil	emb|CABU01000588.1|:8855-8988
	Pangu_ESil	emb|CABU01012483.1|:8026-8104
Hydra vulgaris	Pangu_HVul	gb|ACZU01065412.1|:1628-4435
	Pangu_HVul	gb|ACZU01015265.1|:3146-5704
	Pangu_HVul	gb|ACZU01018302.1|:10542-13074
	Pangu_HVul	gb|ACZU01052009.1|:356-2813
	Pangu_HVul	gb|ACZU01039482.1|:21765-22346
	Pangu_HVul	gb|ACZU01072628.1|:9328-10133
	Pangu_HVul	gb|ACZU01008498.1|:6487-7219
	Pangu_HVul	gb|ACZU01021534.1|:2852-10566
	Pangu_HVul	gb|ACZU01040403.1|:22694-27081
	Pangu_HVul	gb|ACZU01062037.1|:300-2830
	Pangu_HVul	gb|ACZU01019831.1|:3995-5134
	Pangu_HVul	gb|ACZU01023113.1|:821-2627
	Pangu_HVul	gb|ACZU01047613.1|:6525-7248
	Pangu_HVul	gb|ACZU01041504.1|:23121-23796
	Pangu_HVul	gb|ACZU01080501.1|:1-879
	Pangu_HVul	gb|ACZU01021435.1|:25900-26153
	Pangu_HVul	gb|ACZU01058913.1|:70-700
	Pangu_HVul	gb|ACZU01071988.1|:3408-5808
	Pangu_HVul	gb|ACZU01058250.1|:1250-2467
	Pangu_HVul	gb|ACZU01105615.1|:3848-4848
	Pangu_HVul	gb|ACZU01000918.1|:2837-6545
	Pangu_HVul	gb|ACZU01037545.1|:32-929
	Pangu_HVul	gb|ACZU01050136.1|:12049-14602
	Pangu_HVul	gb|ACZU01035441.1|:8599-10528
	Pangu_HVul	gb|ACZU01110112.1|:163-1700
	Pangu_HVul	gb|ACZU01059897.1|:1294-2719
	Pangu_HVul	gb|ACZU01051826.1|:9103-10884
	Pangu_HVul	gb|ACZU01052009.1|:14138-15724
	Pangu_HVul	gb|ACZU01030637.1|:5085-6275
	Pangu_HVul	gb|ACZU01012470.1|:1275-2550
	Pangu_HVul	gb|ACZU01051827.1|:5-654
	Pangu_HVul	gb|ACZU01006773.1|:6078-6682

Pangu_HVul	gb|ACZU01026281.1|:5815-6676
Pangu_HVul	gb|ACZU01067190.1|:43-563
Pangu_HVul	gb|ACZU01072985.1|:25424-25748
Pangu_HVul	gb|ACZU01015052.1|:1312-1658
Pangu_HVul	gb|ACZU01030558.1|:5506-6493
Pangu_HVul	gb|ACZU01009532.1|:5432-5784
Pangu_HVul	gb|ACZU01070109.1|:13321-13677
Pangu_HVul	gb|ACZU01028928.1|:3783-4150
Pangu_HVul	gb|ACZU01099557.1|:143-448
Pangu_HVul	gb|ACZU01061264.1|:1692-1981
Pangu_HVul	gb|ACZU01030308.1|:1151-1442
Pangu_HVul	gb|ACZU01090437.1|:124-428
Pangu_HVul	gb|ACZU01083240.1|:236-529
Pangu_HVul	gb|ACZU01014090.1|:7824-8125
Pangu_HVul	gb|ACZU01080372.1|:756-1059
Pangu_HVul	gb|ACZU01091232.1|:9449-9739
Pangu_HVul	gb|ACZU01072867.1|:94-353
Pangu_HVul	gb|ACZU01029225.1|:9118-9368
Pangu_HVul	gb|ACZU01120782.1|:11-274
Pangu_HVul	gb|ACZU01111853.1|:1900-2162
Pangu_HVul	gb|ACZU01097302.1|:15918-16181
Pangu_HVul	gb|ACZU01099556.1|:2023-2261
Pangu_HVul	gb|ACZU01030309.1|:253-514
Pangu_HVul	gb|ACZU01080566.1|:749-989
Pangu_HVul	gb|ACZU01006694.1|:1531-1784
Pangu_HVul	gb|ACZU01037544.1|:10032-15043
Pangu_HVul	gb|ACZU01043309.1|:3622-3818
Pangu_HVul	gb|ACZU01005740.1|:26262-26446
Pangu_HVul	gb|ACZU01075126.1|:1510-1682
Pangu_HVul	gb|ACZU01102726.1|:3525-3698
Pangu_HVul	gb|ACZU01081889.1|:4578-4751
Pangu_HVul	gb|ACZU01000619.1|:500-672
Pangu_HVul	gb|ACZU01107947.1|:3763-3931
Pangu_HVul	gb|ACZU01066377.1|:124-284
Pangu_HVul	gb|ACZU01107797.1|:586-741
Pangu_HVul	gb|ACZU01018640.1|:804-956
Pangu_HVul	gb|ACZU01090891.1|:126-276
Pangu_HVul	gb|ACZU01063293.1|:10305-10458
Pangu_HVul	gb|ACZU01012469.1|:24848-24976
Pangu_HVul	gb|ACZU01059558.1|:910-1044
Pangu_HVul	gb|ACZU01096389.1|:1405-1539
Pangu_HVul	gb|ACZU01027244.1|:3730-3852
Pangu_HVul	gb|ACZU01090890.1|:1684-1802
Pangu_HVul	gb|ACZU01086416.1|:784-899

	Pangu_HVul	gb|ACZU01086414.1|:688-801
	Pangu_HVul	gb|ACZU01029225.1|:12745-12855
	Pangu_HVul	gb|ACZU01101316.1|:1865-1970
	Pangu_HVul	gb|ACZU01022220.1|:15944-16051
	Pangu_HVul	gb|ACZU01071989.1|:588-686
	Pangu_HVul	gb|ACZU01086727.1|:119-216
	Pangu_HVul	gb|ACZU01087279.1|:6291-6381
	Pangu_HVul	gb|ACZU01080500.1|:18679-18764
	Pangu_HVul	gb|ACZU01099019.1|:6520-6605
	Pangu_HVul	gb|ACZU01053336.1|:652-725
	Pangu_HVul	gb|ACZU01105616.1|:111-179
	Pangu_HVul	gb|ACZU01031018.1|:8907-8971
	Pangu_HVul	gb|ACZU01024830.1|:8297-8361
	Pangu_HVul	gb|ACZU01000922.1|:401-463
	Pangu_HVul	gb|ACZU01116397.1|:648-710
	Pangu_HVul	gb|ACZU01071272.1|:7782-7837
	Pangu_HVul	gb|ACZU01092322.1|:1979-2031
	Pangu_HVul	gb|ACZU01029431.1|:12609-12659
Leptinotarsa decemlineata	Pangu-1_LDec	gb|AYNB01197336.1|:8220-10685
	Pangu-1_LDec	gb|AYNB01094252.1|:8838-11427
	Pangu-1_LDec	gb|AYNB01163968.1|:694-3223
	Pangu-1_LDec	gb|AYNB01185382.1|:3028-5616
	Pangu-1_LDec	gb|AYNB01246520.1|:6397-8857
	Pangu-1_LDec	gb|AYNB01238516.1|:2475-5014
	Pangu-1_LDec	gb|AYNB01022507.1|:1-1089
	Pangu-1_LDec	gb|AYNB01130454.1|:545-1834
	Pangu-1_LDec	gb|AYNB01297604.1|:3867-5421
	Pangu-1_LDec	gb|AYNB01170355.1|:364-2782
	Pangu-1_LDec	gb|AYNB01218726.1|:1-1807
	Pangu-1_LDec	gb|AYNB01277993.1|:2490-3496
	Pangu-1_LDec	gb|AYNB01227866.1|:1-1688
	Pangu-1_LDec	gb|AYNB01145160.1|:860-3388
	Pangu-1_LDec	gb|AYNB01213658.1|:1400-3681
	Pangu-1_LDec	gb|AYNB01227231.1|:933-3216
	Pangu-1_LDec	gb|AYNB01247935.1|:1306-3745
	Pangu-1_LDec	gb|AYNB01000341.1|:9003-10760
	Pangu-1_LDec	gb|AYNB01105752.1|:2613-5175
	Pangu-1_LDec	gb|AYNB01045794.1|:5-2088
	Pangu-1_LDec	gb|AYNB01303112.1|:4465-6250
	Pangu-1_LDec	gb|AYNB01159184.1|:3259-5664
	Pangu-1_LDec	gb|AYNB01277994.1|:1-1164
	Pangu-1_LDec	gb|AYNB01296448.1|:8835-10606
	Pangu-1_LDec	gb|AYNB01223822.1|:1-779
	Pangu-1_LDec	gb|AYNB01044273.1|:4245-6753

	Pangu-1_LDec	gb|AYNB01169973.1|:2982-4864
	Pangu-1_LDec	gb|AYNB01044203.1|:5-772
	Pangu-1_LDec	gb|AYNB01220194.1|:26-1170
	Pangu-1_LDec	gb|AYNB01102991.1|:1-717
	Pangu-1_LDec	gb|AYNB01083343.1|:574-1181
	Pangu-1_LDec	gb|AYNB01293316.1|:625-1233
	Pangu-1_LDec	gb|AYNB01183198.1|:242-842
	Pangu-1_LDec	gb|AYNB01112814.1|:2306-4462
	Pangu-1_LDec	gb|AYNB01248180.1|:152-1783
	Pangu-1_LDec	gb|AYNB01092057.1|:272-829
	Pangu-1_LDec	gb|AYNB01255870.1|:1-420
	Pangu-1_LDec	gb|AYNB01044202.1|:2485-3026
	Pangu-1_LDec	gb|AYNB01149932.1|:1-385
	Pangu-1_LDec	gb|AYNB01012317.1|:1-426
	Pangu-1_LDec	gb|AYNB01062476.1|:2577-3119
	Pangu-1_LDec	gb|AYNB01237428.1|:1-365
	Pangu-1_LDec	gb|AYNB01218725.1|:2574-3081
	Pangu-1_LDec	gb|AYNB01258910.1|:17-385
	Pangu-1_LDec	gb|AYNB01255869.1|:1-268
	Pangu-1_LDec	gb|AYNB01229954.1|:34-364
	Pangu-1_LDec	gb|AYNB01065163.1|:1-381
	Pangu-1_LDec	gb|AYNB01223821.1|:4522-4990
	Pangu-1_LDec	gb|AYNB01222017.1|:1-393
	Pangu-1_LDec	gb|AYNB01220195.1|:1-243
	Pangu-1_LDec	gb|AYNB01229238.1|:1-393
	Pangu-1_LDec	gb|AYNB01130455.1|:1-209
	Pangu-1_LDec	gb|AYNB01191033.1|:1-202
	Pangu-1_LDec	gb|AYNB01220193.1|:1-318
	Pangu-1_LDec	gb|AYNB01092055.1|:1-396
	Pangu-1_LDec	gb|AYNB01293317.1|:1-110
Melampsora larici populina	Pangu-1_MLP	gb|AECX01001054.1|:15122-20252
	Pangu-1_MLP	gb|AECX01001549.1|:5780-10880
	Pangu-1_MLP	gb|AECX01001824.1|:5591-10713
	Pangu-1_MLP	gb|AECX01000113.1|:100459-108265
	Pangu-1_MLP	gb|AECX01000582.1|:122779-128142
	Pangu-1_MLP	gb|AECX01001580.1|:67308-71800
	Pangu-1_MLP	gb|AECX01001580.1|:42772-43271
	Pangu-1_MLP	gb|AECX01001381.1|:94680-98905
	Pangu-1_MLP	gb|AECX01002867.1|:1233-5416
	Pangu-1_MLP	gb|AECX01002323.1|:10940-15739
	Pangu-1_MLP	gb|AECX01001172.1|:17525-17742
	Pangu-1_MLP	gb|AECX01000342.1|:8650-8855
	Pangu-1_MLP	gb|AECX01000366.1|:131646-135578
	Pangu-1_MLP	gb|AECX01002608.1|:24101-24306

Pangu-1_MLP	gb|AECX01001840.1|:49758-50176
Pangu-1_MLP	gb|AECX01001892.1|:20255-20507
Pangu-1_MLP	gb|AECX01002612.1|:6416-6519
Pangu-1_MLP	gb|AECX01000454.1|:46894-46985
Pangu-1_MLP	gb|AECX01000248.1|:67210-70216
Pangu-1_MLP	gb|AECX01002422.1|:10493-10629
Pangu-1_MLP	gb|AECX01000311.1|:112793-112893
Pangu-1_MLP	gb|AECX01001758.1|:156508-156631
Pangu-1_MLP	gb|AECX01001990.1|:39820-39918
Pangu-1_MLP	gb|AECX01002024.1|:14777-15299
Pangu-1_MLP	gb|AECX01001590.1|:90543-90595
Pangu-1_MLP	gb|AECX01000682.1|:208335-208431
Pangu-1_MLP	gb|AECX01000376.1|:123461-123521
Pangu-1_MLP	gb|AECX01001493.1|:72976-73039
Pangu-1_MLP	gb|AECX01001462.1|:116084-116187
Pangu-1_MLP	gb|AECX01001077.1|:135035-135090
Pangu-1_MLP	gb|AECX01000377.1|:3171-3266
Pangu-1_MLP	gb|AECX01000138.1|:76058-76161
Pangu-1_MLP	gb|AECX01000047.1|:17682-17745
Pangu-1_MLP	gb|AECX01001299.1|:60370-60500
Pangu-1_MLP	gb|AECX01000464.1|:160660-160790
Pangu-2_MLP	gb|AECX01001335.1|:81025-85319
Pangu-2_MLP	gb|AECX01002227.1|:41872-46181
Pangu-2_MLP	gb|AECX01000472.1|:245279-250358
Pangu-2_MLP	gb|AECX01001932.1|:5484-10410
Pangu-2_MLP	gb|AECX01000157.1|:177307-182195
Pangu-2_MLP	gb|AECX01002157.1|:202632-203256
Pangu-2_MLP	gb|AECX01002157.1|:1811-1872
Pangu-2_MLP	gb|AECX01000745.1|:92854-97224
Pangu-2_MLP	gb|AECX01001231.1|:1391-1497
Pangu-2_MLP	gb|AECX01001037.1|:174519-174625
Pangu-2_MLP	gb|AECX01000366.1|:131386-131492
Pangu-2_MLP	gb|AECX01000138.1|:252417-252495
Pangu-2_MLP	gb|AECX01000453.1|:108422-108507
Pangu-2_MLP	gb|AECX01001905.1|:252339-252418
Pangu-2_MLP	gb|AECX01001610.1|:12399-12484
Pangu-2_MLP	gb|AECX01001112.1|:425908-426052
Pangu-2_MLP	gb|AECX01002544.1|:7429-7525
Pangu-2_MLP	gb|AECX01001652.1|:19729-19835
Pangu-2_MLP	gb|AECX01000577.1|:165657-165763
Pangu-2_MLP	gb|AECX01000046.1|:154-272
Pangu-2_MLP	gb|AECX01000424.1|:86926-87011
Pangu-2_MLP	gb|AECX01000324.1|:36793-36854
Pangu-2_MLP	gb|AECX01000373.1|:32510-32582

	Pangu-2_MLP	gb|AECX01000103.1|:72415-74063
	Pangu-2_MLP	gb|AECX01001734.1|:28786-29113
	Pangu-2_MLP	gb|AECX01001319.1|:39700-39806
	Pangu-2_MLP	gb|AECX01001103.1|:146597-146694
	Pangu-2_MLP	gb|AECX01000484.1|:8828-8897
	Pangu-2_MLP	gb|AECX01001968.1|:27466-27550
	Pangu-2_MLP	gb|AECX01000302.1|:1286-1390
	Pangu-2_MLP	gb|AECX01001840.1|:49746-49817
	Pangu-2_MLP	gb|AECX01000857.1|:57719-57817
	Pangu-2_MLP	gb|AECX01002400.1|:42649-42746
	Pangu-2_MLP	gb|AECX01000924.1|:103807-103884
	Pangu-2_MLP	gb|AECX01000047.1|:17285-17358
Mesobuthus martensii	Pangu-1_MMar	gb|AYEL01059388.1|:17089-19015
	Pangu-1_MMar	gb|AYEL01062859.1|:3612-5930
	Pangu-1_MMar	gb|AYEL01087261.1|:1057-2963
	Pangu-1_MMar	gb|AYEL01075768.1|:36306-38277
	Pangu-1_MMar	gb|AYEL01063600.1|:1-919
	Pangu-1_MMar	gb|AYEL01075176.1|:18924-20639
	Pangu-1_MMar	gb|AYEL01071447.1|:46915-48369
	Pangu-1_MMar	gb|AYEL01091930.1|:1-1145
	Pangu-1_MMar	gb|AYEL01089746.1|:2408-3539
	Pangu-1_MMar	gb|AYEL01012590.1|:1-589
	Pangu-1_MMar	gb|AYEL01051825.1|:1-512
	Pangu-1_MMar	gb|AYEL01052010.1|:1-560
	Pangu-1_MMar	gb|AYEL01054359.1|:2-537
	Pangu-1_MMar	gb|AYEL01001656.1|:1-525
	Pangu-1_MMar	gb|AYEL01067275.1|:52898-53608
	Pangu-1_MMar	gb|AYEL01067088.1|:21287-22071
	Pangu-1_MMar	gb|AYEL01074971.1|:8347-9629
	Pangu-1_MMar	gb|AYEL01058465.1|:1-844
	Pangu-1_MMar	gb|AYEL01071277.1|:52993-53428
	Pangu-1_MMar	gb|AYEL01079525.1|:7-626
	Pangu-1_MMar	gb|AYEL01012478.1|:11960-12973
	Pangu-1_MMar	gb|AYEL01065082.1|:8504-8923
	Pangu-1_MMar	gb|AYEL01088157.1|:1516-9011
	Pangu-1_MMar	gb|AYEL01022256.1|:87-529
	Pangu-1_MMar	gb|AYEL01072531.1|:20219-20808
	Pangu-1_MMar	gb|AYEL01010637.1|:1-244
	Pangu-1_MMar	gb|AYEL01086568.1|:1075-1786
	Pangu-1_MMar	gb|AYEL01064531.1|:83549-84043
	Pangu-1_MMar	gb|AYEL01086567.1|:8693-9024
	Pangu-1_MMar	gb|AYEL01042086.1|:3042-3160
	Pangu-1_MMar	gb|AYEL01090188.1|:2991-3264
	Pangu-1_MMar	gb|AYEL01008167.1|:211-330

	Pangu-1_MMar	gb|AYEL01077366.1|:10142-10586
	Pangu-1_MMar	gb|AYEL01039375.1|:702-858
	Pangu-1_MMar	gb|AYEL01020770.1|:1221-1352
	Pangu-1_MMar	gb|AYEL01023641.1|:361-448
	Pangu-1_MMar	gb|AYEL01089435.1|:19085-19196
	Pangu-1_MMar	gb|AYEL01068117.1|:21184-21268
	Pangu-1_MMar	gb|AYEL01079589.1|:1-130
	Pangu-1_MMar	gb|AYEL01087685.1|:51594-51702
	Pangu-1_MMar	gb|AYEL01089499.1|:8546-8613
	Pangu-1_MMar	gb|AYEL01084532.1|:2692-2775
	Pangu-1_MMar	gb|AYEL01090970.1|:19368-19420
	Pangu-1_MMar	gb|AYEL01055643.1|:115876-117627
	Pangu-1_MMar	gb|AYEL01056057.1|:7105-7204
	Pangu-1_MMar	gb|AYEL01052009.1|:6195-6254
	Pangu-1_MMar	gb|AYEL01062530.1|:68496-68544
	Pangu-2_MMar	gb|AYEL01063958.1|:18289-21207
	Pangu-2_MMar	gb|AYEL01090302.1|:23749-26793
	Pangu-2_MMar	gb|AYEL01088368.1|:1-1679
	Pangu-2_MMar	gb|AYEL01066202.1|:12168-15378
	Pangu-2_MMar	gb|AYEL01071013.1|:1-1864
	Pangu-2_MMar	gb|AYEL01090644.1|:1-862
	Pangu-2_MMar	gb|AYEL01091341.1|:914-2218
	Pangu-2_MMar	gb|AYEL01085045.1|:15904-16651
	Pangu-2_MMar	gb|AYEL01088367.1|:41871-42620
	Pangu-2_MMar	gb|AYEL01079297.1|:30076-30596
	Pangu-2_MMar	gb|AYEL01079297.1|:27549-27686
	Pangu-2_MMar	gb|AYEL01079297.1|:14581-17436
	Pangu-2_MMar	gb|AYEL01085046.1|:20-560
	Pangu-2_MMar	gb|AYEL01084444.1|:80851-81259
	Pangu-2_MMar	gb|AYEL01051203.1|:1-368
	Pangu-2_MMar	gb|AYEL01005635.1|:1-351
	Pangu-2_MMar	gb|AYEL01005461.1|:1-338
	Pangu-2_MMar	gb|AYEL01076566.1|:53772-54152
	Pangu-2_MMar	gb|AYEL01064694.1|:29864-30243
	Pangu-2_MMar	gb|AYEL01080916.1|:1619-2100
	Pangu-2_MMar	gb|AYEL01062156.1|:1-363
	Pangu-2_MMar	gb|AYEL01073361.1|:26746-27125
	Pangu-2_MMar	gb|AYEL01078397.1|:35338-35490
	Pangu-2_MMar	gb|AYEL01060927.1|:19305-19447
	Pangu-2_MMar	gb|AYEL01032430.1|:1-158
	Pangu-2_MMar	gb|AYEL01035030.1|:1-151
	Pangu-2_MMar	gb|AYEL01087673.1|:4836-4911
	Pangu-2_MMar	gb|AYEL01084409.1|:1505-1554
Phytophthora infestans	Pangu-1_PInf	gb|AATU01004830.1|:136270-138998

	Pangu-1_PInf	gb|AATU01004830.1|:213530-210803
	Pangu-1_PInf	gb|AATU01016103.1|:218-2802
	Pangu-1_PInf	gb|AATU01000625.1|:132514-129788
	Pangu-1_PInf	gb|AATU01007449.1|:2018-4744
	Pangu-1_PInf	gb|AATU01005283.1|:15930-14894
	Pangu-2_PInf	gb|AATU01002213.1|:170938-173029
	Pangu-2_PInf	gb|AATU01003152.1|:27520-25429
	Pangu-2_PInf	gb|AATU01003739.1|:2783-4871
	Pangu-2_PInf	gb|AATU01006509.1|:25793-27888
	Pangu-2_PInf	gb|AATU01006509.1|:26596-26414
	Pangu-2_PInf	gb|AATU01013993.1|:360-1284
	Pangu-2_PInf	gb|AATU01012672.1|:2090-2951
	Pangu-2_PInf	gb|AATU01005256.1|:21196-22056
	Pangu-2_PInf	gb|AATU01001040.1|:50159-51020
	Pangu-2_PInf	gb|AATU01006668.1|:14529-13682
	Pangu-2_PInf	gb|AATU01003500.1|:80663-79803
	Pangu-2_PInf	gb|AATU01003912.1|:7150-6290
	Pangu-2_PInf	gb|AATU01005263.1|:47943-47093
	Pangu-2_PInf	gb|AATU01005466.1|:28286-29145
	Pangu-2_PInf	gb|AATU01004830.1|:85501-84644
	Pangu-2_PInf	gb|AATU01000908.1|:46709-47571
	Pangu-2_PInf	gb|AATU01004774.1|:461-1309
	Pangu-2_PInf	gb|AATU01004295.1|:38671-39517
	Pangu-2_PInf	gb|AATU01002850.1|:54103-54181
	Pangu-2_PInf	gb|AATU01002850.1|:23852-23070
	Pangu-2_PInf	gb|AATU01000617.1|:15076-15789
	Pangu-2_PInf	gb|AATU01001320.1|:153541-154566
	Pangu-2_PInf	gb|AATU01010986.1|:191-975
	Pangu-2_PInf	gb|AATU01001818.1|:2032-1318
	Pangu-2_PInf	gb|AATU01007940.1|:17626-18423
	Pangu-2_PInf	gb|AATU01007297.1|:20641-21239
	Pangu-2_PInf	gb|AATU01011744.1|:639-1236
	Pangu-2_PInf	gb|AATU01005163.1|:28489-36687
	Pangu-2_PInf	gb|AATU01005787.1|:3361-3632
	Pangu-2_PInf	gb|AATU01007299.1|:5624-5371
	Pangu-2_PInf	gb|AATU01013465.1|:6083-6349
	Pangu-2_PInf	gb|AATU01005535.1|:104293-105532
Puccinia striiformis	Pangu_PStr	gb|ANHQ01010974.1|:1313-1511
	Pangu_PStr	gb|ANHQ01001709.1|:3151-3349
	Pangu_PStr	gb|ANHQ01000139.1|:748-946
	Pangu_PStr	gb|ANHQ01012471.1|:1254-1452
	Pangu_PStr	gb|ANHQ01006037.1|:14137-14335
	Pangu_PStr	gb|ANHQ01012205.1|:1262-1460
	Pangu_PStr	gb|ANHQ01011250.1|:4503-4701

Pangu_PStr	gb|ANHQ01008513.1|:11425-11623
Pangu_PStr	gb|ANHQ01006466.1|:2735-2933
Pangu_PStr	gb|ANHQ01003181.1|:2918-3116
Pangu_PStr	gb|ANHQ01002325.1|:5291-5489
Pangu_PStr	gb|ANHQ01007827.1|:463-656
Pangu_PStr	gb|ANHQ01009115.1|:33666-33864
Pangu_PStr	gb|ANHQ01012062.1|:3514-3712
Pangu_PStr	gb|ANHQ01001167.1|:42017-42215
Pangu_PStr	gb|ANHQ01002332.1|:28190-28386
Pangu_PStr	gb|ANHQ01012253.1|:42195-42393
Pangu_PStr	gb|ANHQ01003571.1|:9031-9229
Pangu_PStr	gb|ANHQ01009330.1|:399-598
Pangu_PStr	gb|ANHQ01001538.1|:15040-15238
Pangu_PStr	gb|ANHQ01001345.1|:8717-8915
Pangu_PStr	gb|ANHQ01011131.1|:3590-3788
Pangu_PStr	gb|ANHQ01009942.1|:12322-12520
Pangu_PStr	gb|ANHQ01000527.1|:18829-19027
Pangu_PStr	gb|ANHQ01011447.1|:18841-20293
Pangu_PStr	gb|ANHQ01011202.1|:10098-10294
Pangu_PStr	gb|ANHQ01006065.1|:1164-1352
Pangu_PStr	gb|ANHQ01011537.1|:4273-4461
Pangu_PStr	gb|ANHQ01002288.1|:8114-8302
Pangu_PStr	gb|ANHQ01009128.1|:9765-9958
Pangu_PStr	gb|ANHQ01002909.1|:2402-2595
Pangu_PStr	gb|ANHQ01003154.1|:1058-1252
Pangu_PStr	gb|ANHQ01000157.1|:11418-12906
Pangu_PStr	gb|ANHQ01006093.1|:10057-10239
Pangu_PStr	gb|ANHQ01010122.1|:9822-12435
Pangu_PStr	gb|ANHQ01005297.1|:20329-20520
Pangu_PStr	gb|ANHQ01007827.1|:16792-16979
Pangu_PStr	gb|ANHQ01000395.1|:7463-7650
Pangu_PStr	gb|ANHQ01001054.1|:17029-17204
Pangu_PStr	gb|ANHQ01001970.1|:574-752
Pangu_PStr	gb|ANHQ01009284.1|:5616-5790
Pangu_PStr	gb|ANHQ01007666.1|:27692-27855
Pangu_PStr	gb|ANHQ01012474.1|:11244-11890
Pangu_PStr	gb|ANHQ01008118.1|:34675-34840
Pangu_PStr	gb|ANHQ01011077.1|:692-857
Pangu_PStr	gb|ANHQ01005292.1|:9052-9217
Pangu_PStr	gb|ANHQ01001453.1|:4572-4738
Pangu_PStr	gb|ANHQ01002520.1|:2395-2560
Pangu_PStr	gb|ANHQ01006036.1|:9838-9987
Pangu_PStr	gb|ANHQ01005453.1|:697-852
Pangu_PStr	gb|ANHQ01005827.1|:33930-34087

Pangu_PStr	gb|ANHQ01002936.1|:3073-3230
Pangu_PStr	gb|ANHQ01000637.1|:8974-9130
Pangu_PStr	gb|ANHQ01002134.1|:428-1337
Pangu_PStr	gb|ANHQ01003410.1|:27917-28091
Pangu_PStr	gb|ANHQ01007933.1|:12902-13041
Pangu_PStr	gb|ANHQ01009393.1|:9072-9625
Pangu_PStr	gb|ANHQ01006587.1|:1168-1722
Pangu_PStr	gb|ANHQ01009954.1|:1315-1466
Pangu_PStr	gb|ANHQ01004909.1|:32687-32838
Pangu_PStr	gb|ANHQ01006936.1|:7550-7669
Pangu_PStr	gb|ANHQ01002487.1|:107978-108092
Pangu_PStr	gb|ANHQ01005736.1|:17397-17508
Pangu_PStr	gb|ANHQ01001168.1|:7863-7974
Pangu_PStr	gb|ANHQ01006198.1|:17538-17735
Pangu_PStr	gb|ANHQ01007413.1|:17603-17801
Pangu_PStr	gb|ANHQ01003978.1|:24495-24693
Pangu_PStr	gb|ANHQ01007728.1|:10059-10257
Pangu_PStr	gb|ANHQ01001387.1|:4156-4353
Pangu_PStr	gb|ANHQ01012434.1|:3630-3850
Pangu_PStr	gb|ANHQ01012291.1|:5350-5451
Pangu_PStr	gb|ANHQ01012290.1|:29431-29532
Pangu_PStr	gb|ANHQ01009394.1|:18250-18351
Pangu_PStr	gb|ANHQ01001281.1|:7550-7762
Pangu_PStr	gb|ANHQ01002831.1|:3933-4045
Pangu_PStr	gb|ANHQ01006951.1|:40048-40243
Pangu_PStr	gb|ANHQ01008761.1|:5658-5836
Pangu_PStr	gb|ANHQ01002666.1|:4961-5154
Pangu_PStr	gb|ANHQ01000909.1|:5992-6185
Pangu_PStr	gb|ANHQ01001639.1|:511-606
Pangu_PStr	gb|ANHQ01008644.1|:14885-15083
Pangu_PStr	gb|ANHQ01002292.1|:1353-1551
Pangu_PStr	gb|ANHQ01010453.1|:16002-16200
Pangu_PStr	gb|ANHQ01001120.1|:2485-2683
Pangu_PStr	gb|ANHQ01004752.1|:2880-3078
Pangu_PStr	gb|ANHQ01004143.1|:1564-1754
Pangu_PStr	gb|ANHQ01011971.1|:408-606
Pangu_PStr	gb|ANHQ01008263.1|:12256-12453
Pangu_PStr	gb|ANHQ01007952.1|:558-757
Pangu_PStr	gb|ANHQ01006286.1|:11255-11438
Pangu_PStr	gb|ANHQ01004546.1|:2606-2795
Pangu_PStr	gb|ANHQ01000621.1|:18808-18893
Pangu_PStr	gb|ANHQ01005084.1|:25981-26065
Pangu_PStr	gb|ANHQ01005298.1|:8106-8299
Pangu_PStr	gb|ANHQ01000283.1|:7675-7868

Pangu_PStr	gb|ANHQ01001280.1|:15335-15416
Pangu_PStr	gb|ANHQ01011239.1|:26416-26593
Pangu_PStr	gb|ANHQ01003256.1|:34393-34569
Pangu_PStr	gb|ANHQ01002574.1|:30402-30479
Pangu_PStr	gb|ANHQ01011631.1|:54051-54129
Pangu_PStr	gb|ANHQ01007549.1|:1235-1309
Pangu_PStr	gb|ANHQ01002487.1|:88066-88133
Pangu_PStr	gb|ANHQ01007741.1|:10043-10110
Pangu_PStr	gb|ANHQ01004141.1|:18406-18471
Pangu_PStr	gb|ANHQ01000392.1|:4902-4967
Pangu_PStr	gb|ANHQ01011309.1|:26526-26585
Pangu_PStr	gb|ANHQ01005131.1|:6582-6641
Pangu_PStr	gb|ANHQ01004299.1|:81954-82009
Pangu_PStr	gb|ANHQ01010003.1|:2355-2405
Pangu_PStr	gb|ANHQ01007961.1|:740-790
Pangu_PStr	gb|ANHQ01011064.1|:10783-10833
Pangu_PStr	gb|ANHQ01009337.1|:4646-4696
Pangu_PStr	gb|ANHQ01011011.1|:11044-11094
Pangu_PStr	gb|ANHQ01007083.1|:14814-14864
Pangu_PStr	gb|ANHQ01005967.1|:6540-6590
Pangu_PStr	gb|ANHQ01001594.1|:45391-45441
Pangu_PStr	gb|ANHQ01005242.1|:72231-72281
Pangu_PStr	gb|ANHQ01011354.1|:12313-12362
Pangu_PStr	gb|ANHQ01011266.1|:9338-9387
Pangu_PStr	gb|ANHQ01011971.1|:408-606
Pangu_PStr	gb|ANHQ01010453.1|:16002-16200
Pangu_PStr	gb|ANHQ01001120.1|:2485-2683
Pangu_PStr	gb|ANHQ01006198.1|:17538-17735
Pangu_PStr	gb|ANHQ01007952.1|:558-757
Pangu_PStr	gb|ANHQ01004752.1|:2880-3078
Pangu_PStr	gb|ANHQ01011785.1|:51839-52037
Pangu_PStr	gb|ANHQ01007413.1|:17603-17801
Pangu_PStr	gb|ANHQ01004143.1|:1564-1754
Pangu_PStr	gb|ANHQ01010003.1|:2355-2553
Pangu_PStr	gb|ANHQ01005298.1|:8106-8299
Pangu_PStr	gb|ANHQ01000283.1|:7675-7868
Pangu_PStr	gb|ANHQ01007573.1|:4961-5160
Pangu_PStr	gb|ANHQ01003256.1|:34371-34569
Pangu_PStr	gb|ANHQ01002666.1|:941-1139
Pangu_PStr	gb|ANHQ01000909.1|:2265-2463
Pangu_PStr	gb|ANHQ01003978.1|:24495-24693
Pangu_PStr	gb|ANHQ01007728.1|:10059-10257
Pangu_PStr	gb|ANHQ01001387.1|:4156-4353
Pangu_PStr	gb|ANHQ01008644.1|:14885-15083

Pangu_PStr	gb|ANHQ01002292.1|:1353-1551
Pangu_PStr	gb|ANHQ01007083.1|:14814-15011
Pangu_PStr	gb|ANHQ01011354.1|:12313-12509
Pangu_PStr	gb|ANHQ01011266.1|:9338-9534
Pangu_PStr	gb|ANHQ01008263.1|:12256-12453
Pangu_PStr	gb|ANHQ01006951.1|:40048-40243
Pangu_PStr	gb|ANHQ01007634.1|:4-191
Pangu_PStr	gb|ANHQ01008029.1|:6753-6941
Pangu_PStr	gb|ANHQ01011011.1|:10900-11094
Pangu_PStr	gb|ANHQ01000643.1|:29824-34253
Pangu_PStr	gb|ANHQ01006286.1|:11255-11438
Pangu_PStr	gb|ANHQ01002132.1|:37-968
Pangu_PStr	gb|ANHQ01011239.1|:26416-26594
Pangu_PStr	gb|ANHQ01012434.1|:3675-3850
Pangu_PStr	gb|ANHQ01005967.1|:6540-6736
Pangu_PStr	gb|ANHQ01003605.1|:10112-10282
Pangu_PStr	gb|ANHQ01005412.1|:7400-7564
Pangu_PStr	gb|ANHQ01004546.1|:2606-2771
Pangu_PStr	gb|ANHQ01009433.1|:1075-1234
Pangu_PStr	gb|ANHQ01008118.1|:51414-51563
Pangu_PStr	gb|ANHQ01012141.1|:14804-14960
Pangu_PStr	gb|ANHQ01005242.1|:72231-72928
Pangu_PStr	gb|ANHQ01004474.1|:1-153
Pangu_PStr	gb|ANHQ01011064.1|:10783-10935
Pangu_PStr	gb|ANHQ01010867.1|:1289-1439
Pangu_PStr	gb|ANHQ01005205.1|:1090-1240
Pangu_PStr	gb|ANHQ01007961.1|:740-889
Pangu_PStr	gb|ANHQ01004039.1|:38596-38745
Pangu_PStr	gb|ANHQ01004039.1|:8697-8846
Pangu_PStr	gb|ANHQ01001281.1|:7550-7762
Pangu_PStr	gb|ANHQ01003410.1|:27595-28091
Pangu_PStr	gb|ANHQ01001594.1|:45299-45441
Pangu_PStr	gb|ANHQ01000357.1|:18663-18805
Pangu_PStr	gb|ANHQ01010337.1|:4481-4607
Pangu_PStr	gb|ANHQ01001746.1|:21601-21727
Pangu_PStr	gb|ANHQ01003511.1|:7347-7484
Pangu_PStr	gb|ANHQ01012162.1|:40669-40799
Pangu_PStr	gb|ANHQ01001231.1|:2756-2882
Pangu_PStr	gb|ANHQ01000266.1|:2800-2926
Pangu_PStr	gb|ANHQ01001689.1|:40367-40641
Pangu_PStr	gb|ANHQ01010869.1|:10197-10319
Pangu_PStr	gb|ANHQ01003843.1|:784-903
Pangu_PStr	gb|ANHQ01004791.1|:143-289
Pangu_PStr	gb|ANHQ01004790.1|:102-248

Pangu_PStr	gb|ANHQ01009337.1|:4583-4696
Pangu_PStr	gb|ANHQ01000867.1|:38625-38736
Pangu_PStr	gb|ANHQ01006709.1|:9991-10102
Pangu_PStr	gb|ANHQ01008175.1|:3568-3672
Pangu_PStr	gb|ANHQ01007961.1|:24148-24252
Pangu_PStr	gb|ANHQ01010485.1|:6104-6208
Pangu_PStr	gb|ANHQ01001910.1|:14851-14952
Pangu_PStr	gb|ANHQ01006037.1|:14137-14335
Pangu_PStr	gb|ANHQ01011250.1|:4503-4701
Pangu_PStr	gb|ANHQ01003181.1|:2918-3116
Pangu_PStr	gb|ANHQ01002332.1|:28190-28386
Pangu_PStr	gb|ANHQ01003571.1|:9031-9229
Pangu_PStr	gb|ANHQ01001538.1|:15040-15238
Pangu_PStr	gb|ANHQ01011131.1|:3590-3788
Pangu_PStr	gb|ANHQ01000157.1|:11418-12906
Pangu_PStr	gb|ANHQ01000644.1|:4774-4886
Pangu_PStr	gb|ANHQ01004818.1|:26817-26995
Pangu_PStr	gb|ANHQ01001110.1|:12648-12826
Pangu_PStr	gb|ANHQ01003683.1|:2189-2286
Pangu_PStr	gb|ANHQ01009583.1|:14677-24429
Pangu_PStr	gb|ANHQ01010974.1|:1313-1511
Pangu_PStr	gb|ANHQ01001709.1|:3151-3349
Pangu_PStr	gb|ANHQ01000139.1|:748-946
Pangu_PStr	gb|ANHQ01012205.1|:1262-1460
Pangu_PStr	gb|ANHQ01009942.1|:12322-12520
Pangu_PStr	gb|ANHQ01000527.1|:18829-19027
Pangu_PStr	gb|ANHQ01010122.1|:9822-12435
Pangu_PStr	gb|ANHQ01008118.1|:34651-34840
Pangu_PStr	gb|ANHQ01002040.1|:1083-1168
Pangu_PStr	gb|ANHQ01002410.1|:3190-3274
Pangu_PStr	gb|ANHQ01001234.1|:7163-7263
Pangu_PStr	gb|ANHQ01007827.1|:463-656
Pangu_PStr	gb|ANHQ01001652.1|:17485-17799
Pangu_PStr	gb|ANHQ01004857.1|:26878-26955
Pangu_PStr	gb|ANHQ01002520.1|:2395-2578
Pangu_PStr	gb|ANHQ01010883.1|:27446-27518
Pangu_PStr	gb|ANHQ01011826.1|:6118-6188
Pangu_PStr	gb|ANHQ01008782.1|:15481-15550
Pangu_PStr	gb|ANHQ01008103.1|:16917-16984
Pangu_PStr	gb|ANHQ01005798.1|:6751-6818
Pangu_PStr	gb|ANHQ01001353.1|:9640-9707
Pangu_PStr	gb|ANHQ01006235.1|:3224-3269
Pangu_PStr	gb|ANHQ01008923.1|:2335-2394
Pangu_PStr	gb|ANHQ01000504.1|:1516-1572

	Pangu_PStr	gb|ANHQ01002165.1|:808-863
	Pangu_PStr	gb|ANHQ01008513.1|:11573-11623
	Pangu_PStr	gb|ANHQ01006466.1|:2883-2933
	Pangu_PStr	gb|ANHQ01009115.1|:33666-33716
	Pangu_PStr	gb|ANHQ01001167.1|:42165-42215
	Pangu_PStr	gb|ANHQ01012253.1|:42195-42245
	Pangu_PStr	gb|ANHQ01001453.1|:4688-4738
	Pangu_PStr	gb|ANHQ01001280.1|:15335-15385
	Pangu_PStr	gb|ANHQ01003154.1|:1058-1109
	Pangu_PStr	gb|ANHQ01006093.1|:10189-10239
Talaromyces stipitatus	Pangu_Tsti	gb|ABAS01000028.1|:1862-4428
	Pangu_Tsti	gb|ABAS01000002.1|:2688957-2690938
	Pangu_Tsti	gb|ABAS01000220.1|:1-1489
	Pangu_Tsti	gb|ABAS01000388.1|:1-1056
	Pangu_Tsti	gb|ABAS01000047.1|:6480-6640
	Pangu_Tsti	gb|ABAS01000009.1|:872672-872732
	Pangu_Tsti	gb|ABAS01000009.1|:872576-875142
	Pangu_Tsti	gb|ABAS01000003.1|:1916895-1919430
	Pangu_Tsti	gb|ABAS01000317.1|:1-1155
	Pangu_Tsti	gb|ABAS01000408.1|:1-1041
	Pangu_Tsti	gb|ABAS01000016.1|:1-270
	Pangu_Tsti	gb|ABAS01000009.1|:910744-910906
	Pangu_Tsti	gb|ABAS01000006.1|:151021-151313
	Pangu_Tsti	gb|ABAS01000037.1|:4464-4613
	Pangu_Tsti	gb|ABAS01000028.1|:4272-4332
Tremella mesenterica	Pangu_Tmes	gb|AFVY01000242.1|:240-3519
	Pangu_Tmes	gb|AFVY01000171.1|:374121-377405
	Pangu_Tmes	gb|AFVY01000330.1|:33827-33939
	Pangu_Tmes	gb|AFVY01000176.1|:197932-201211
	Pangu_Tmes	gb|AFVY01000305.1|:11799-15078
	Pangu_Tmes	gb|AFVY01000171.1|:422343-425622
	Pangu_Tmes	gb|AFVY01000340.1|:1-2612
	Pangu_Tmes	gb|AFVY01000323.1|:44010-47874
	Pangu_Tmes	gb|AFVY01000289.1|:64081-67374
	Pangu_Tmes	gb|AFVY01000354.1|:13578-14096
	Pangu_Tmes	gb|AFVY01000440.1|:62299-63203
Tuber melanosporum	Pangu-1_Tmel	emb|CABJ01003125.1|:56615-59336
	Pangu-1_Tmel	emb|CABJ01003125.1|:56615-59336
	Pangu-1_Tmel	emb|CABJ01003101.1|:50022-50322
	Pangu-1_Tmel	emb|CABJ01003101.1|:37288-39721
	Pangu-1_Tmel	emb|CABJ01004116.1|:8289-17901
	Pangu-1_Tmel	emb|CABJ01004116.1|:8289-17901
	Pangu-1_Tmel	emb|CABJ01001933.1|:14862-15949
	Pangu-1_Tmel	emb|CABJ01001940.1|:6104-7753

Pangu-1_Tmel	emb|CABJ01001194.1|:11663-19335
Pangu-1_Tmel	emb|CABJ01002821.1|:57025-57163
Pangu-1_Tmel	emb|CABJ01002785.1|:36493-36599
Pangu-1_Tmel	emb|CABJ01002605.1|:23930-24032
Pangu-1_Tmel	emb|CABJ01001999.1|:34677-34782
Pangu-1_Tmel	emb|CABJ01001749.1|:20811-20930
Pangu-1_Tmel	emb|CABJ01001014.1|:91105-91206
Pangu-1_Tmel	emb|CABJ01003973.1|:30553-30647
Pangu-1_Tmel	emb|CABJ01001487.1|:5919-5985
Pangu-1_Tmel	emb|CABJ01001536.1|:16297-16384
Pangu-1_Tmel	emb|CABJ01002340.1|:16481-18215
Pangu-1_Tmel	emb|CABJ01002196.1|:33191-33249
Pangu-1_Tmel	emb|CABJ01001542.1|:54661-54715
Pangu-2_Tmel	emb|CABJ01004228.1|:7376-10532
Pangu-2_Tmel	emb|CABJ01001370.1|:8004-11160
Pangu-2_Tmel	emb|CABJ01000461.1|:16547-19702
Pangu-2_Tmel	emb|CABJ01001463.1|:20852-24016
Pangu-2_Tmel	emb|CABJ01003125.1|:43401-48122
Pangu-2_Tmel	emb|CABJ01003864.1|:11287-12677
Pangu-2_Tmel	emb|CABJ01000399.1|:161883-161948
Pangu-2_Tmel	emb|CABJ01002525.1|:47686-47751
Pangu-3_Tmel	emb|CABJ01000811.1|:17482-20541
Pangu-3_Tmel	emb|CABJ01001118.1|:38194-41253
Pangu-3_Tmel	emb|CABJ01000583.1|:12121-15177
Pangu-3_Tmel	emb|CABJ01003909.1|:29100-32154
Pangu-3_Tmel	emb|CABJ01002939.1|:23385-26454
Pangu-3_Tmel	emb|CABJ01003122.1|:13612-13680
Pangu-3_Tmel	emb|CABJ01001494.1|:8212-8362
Pangu-3_Tmel	emb|CABJ01000307.1|:73857-73942
Pangu-3_Tmel	emb|CABJ01000307.1|:90011-90097
Pangu-3_Tmel	emb|CABJ01000307.1|:115554-115607
Pangu-3_Tmel	emb|CABJ01000307.1|:28648-28733
Pangu-3_Tmel	emb|CABJ01003874.1|:42080-42157
Pangu-3_Tmel	emb|CABJ01002137.1|:233460-233513
Pangu-3_Tmel	emb|CABJ01002137.1|:218103-218180
Pangu-3_Tmel	emb|CABJ01002137.1|:182361-182444
Pangu-3_Tmel	emb|CABJ01002137.1|:135275-135350
Pangu-3_Tmel	emb|CABJ01002198.1|:128281-128348
Pangu-3_Tmel	emb|CABJ01002170.1|:61102-61204
Pangu-3_Tmel	emb|CABJ01002355.1|:74716-74777
Pangu-3_Tmel	emb|CABJ01002531.1|:2123-2183
Pangu-3_Tmel	emb|CABJ01002531.1|:22044-22102
Pangu-3_Tmel	emb|CABJ01000412.1|:191324-191484
Pangu-3_Tmel	emb|CABJ01000250.1|:4221-4281

Pangu-3_Tmel	emb|CABJ01001229.1|:766778-766839
Pangu-3_Tmel	emb|CABJ01001229.1|:605052-605128
Pangu-3_Tmel	emb|CABJ01000170.1|:58748-58823
Pangu-3_Tmel	emb|CABJ01003330.1|:24297-24351
Pangu-3_Tmel	emb|CABJ01003119.1|:5929-6007
Pangu-3_Tmel	emb|CABJ01000530.1|:436-506
Pangu-3_Tmel	emb|CABJ01000029.1|:4030-4116
Pangu-3_Tmel	emb|CABJ01002820.1|:10121-10174
Pangu-3_Tmel	emb|CABJ01002529.1|:34118-34179
Pangu-3_Tmel	emb|CABJ01002290.1|:17968-18029
Pangu-3_Tmel	emb|CABJ01001299.1|:2746-2823
Pangu-3_Tmel	emb|CABJ01002291.1|:6012-6073
Pangu-3_Tmel	emb|CABJ01002291.1|:28294-28360
Pangu-3_Tmel	emb|CABJ01000668.1|:6916-6970
Pangu-3_Tmel	emb|CABJ01002590.1|:59294-59371

Table S6. Positions of *NuwaI* transposons in the corresponding genome

Species	Families	Scaffold:start-end
Crassostrea gigas	*NuwaI-1_CGig*	gb|AFTI01010522.1|:2623-4469
	NuwaI-1_CGig	gb|AFTI01001420.1|:8434-10281
	NuwaI-1_CGig	gb|AFTI01020034.1|:31692-33527
	NuwaI-1_CGig	gb|AFTI01014247.1|:31737-35002
	NuwaI-1_CGig	gb|AFTI01030136.1|:3826-5902
	NuwaI-1_CGig	gb|AFTI01001165.1|:11017-13177
	NuwaI-1_CGig	gb|AFTI01020961.1|:22336-23847
	NuwaI-1_CGig	gb|AFTI01020961.1|:9516-10696
	NuwaI-1_CGig	gb|AFTI01029430.1|:1-1797
	NuwaI-1_CGig	gb|AFTI01011834.1|:5388-7226
	NuwaI-1_CGig	gb|AFTI01029966.1|:12355-14193
	NuwaI-1_CGig	gb|AFTI01010026.1|:77525-79709
	NuwaI-1_CGig	gb|AFTI01006849.1|:18066-19899
	NuwaI-1_CGig	gb|AFTI01003459.1|:7578-9188
	NuwaI-1_CGig	gb|AFTI01020189.1|:65857-67697
	NuwaI-1_CGig	gb|AFTI01014643.1|:57191-58590
	NuwaI-1_CGig	gb|AFTI01016499.1|:1-897
	NuwaI-1_CGig	gb|AFTI01001913.1|:11034-12846
	NuwaI-1_CGig	gb|AFTI01020574.1|:2686-4247
	NuwaI-1_CGig	gb|AFTI01003992.1|:2862-5176
	NuwaI-1_CGig	gb|AFTI01004755.1|:15186-17652
	NuwaI-1_CGig	gb|AFTI01006567.1|:4810-5486
	NuwaI-1_CGig	gb|AFTI01028434.1|:3067-4206
	NuwaI-1_CGig	gb|AFTI01005958.1|:4954-5850
	NuwaI-1_CGig	gb|AFTI01005530.1|:20917-22721
	NuwaI-1_CGig	gb|AFTI01009772.1|:3716-5524
	NuwaI-1_CGig	gb|AFTI01019539.1|:3666-5475
	NuwaI-1_CGig	gb|AFTI01020158.1|:4551-5150
	NuwaI-1_CGig	gb|AFTI01017772.1|:2922-4444
	NuwaI-1_CGig	gb|AFTI01017772.1|:1719-1999
	NuwaI-1_CGig	gb|AFTI01005389.1|:245-771
	NuwaI-1_CGig	gb|AFTI01015274.1|:21779-24814
	NuwaI-1_CGig	gb|AFTI01014991.1|:7394-9375
	NuwaI-1_CGig	gb|AFTI01012002.1|:12419-13475
	NuwaI-1_CGig	gb|AFTI01012002.1|:725-810
	NuwaI-1_CGig	gb|AFTI01003940.1|:2761-6744
	NuwaI-1_CGig	gb|AFTI01014330.1|:34743-35708
	NuwaI-1_CGig	gb|AFTI01020050.1|:2849-4936
	NuwaI-1_CGig	gb|AFTI01024323.1|:12813-14620
	NuwaI-1_CGig	gb|AFTI01007190.1|:16585-16893
	NuwaI-1_CGig	gb|AFTI01006568.1|:1-246
	NuwaI-1_CGig	gb|AFTI01014008.1|:4724-5234

NuwaI-1_CGig	gb|AFTI01006535.1|:14218-14489
NuwaI-1_CGig	gb|AFTI01012086.1|:37362-37603
NuwaI-1_CGig	gb|AFTI01004657.1|:1126-1327
NuwaI-1_CGig	gb|AFTI01000548.1|:22686-22854
NuwaI-1_CGig	gb|AFTI01025084.1|:4321-4612
NuwaI-1_CGig	gb|AFTI01023391.1|:55636-55788
NuwaI-1_CGig	gb|AFTI01014806.1|:96400-96671
NuwaI-1_CGig	gb|AFTI01011280.1|:12727-16578
NuwaI-1_CGig	gb|AFTI01000325.1|:273-4071
NuwaI-1_CGig	gb|AFTI01023119.1|:9362-12350
NuwaI-1_CGig	gb|AFTI01004348.1|:35360-39097
NuwaI-1_CGig	gb|AFTI01016730.1|:2338-4117
NuwaI-1_CGig	gb|AFTI01021277.1|:26292-26408
NuwaI-1_CGig	gb|AFTI01019340.1|:1976-5112
NuwaI-1_CGig	gb|AFTI01008991.1|:3184-3277
NuwaI-1_CGig	gb|AFTI01010155.1|:3211-3325
NuwaI-1_CGig	gb|AFTI01030385.1|:2271-2339
NuwaI-1_CGig	gb|AFTI01007145.1|:190-270
NuwaI-1_CGig	gb|AFTI01006583.1|:7461-7529
NuwaI-1_CGig	gb|AFTI01023063.1|:71792-72145
NuwaI-1_CGig	gb|AFTI01007359.1|:19583-22331
NuwaI-1_CGig	gb|AFTI01003628.1|:32384-32442
NuwaI-1_CGig	gb|AFTI01002897.1|:7535-7615
NuwaI-1_CGig	gb|AFTI01021375.1|:7531-7614
NuwaI-1_CGig	gb|AFTI01014254.1|:1508-1598
NuwaI-1_CGig	gb|AFTI01012571.1|:4044-4094
NuwaI-1_CGig	gb|AFTI01008040.1|:41940-42045
NuwaI-1_CGig	gb|AFTI01014255.1|:3193-3273
NuwaI-1_CGig	gb|AFTI01012616.1|:8989-9101
NuwaI-1_CGig	gb|AFTI01015703.1|:6228-6291
NuwaI-1_CGig	gb|AFTI01008208.1|:7169-7220
NuwaI-1_CGig	gb|AFTI01015083.1|:3742-3857
NuwaI-1_CGig	gb|AFTI01029755.1|:1957-2044
NuwaI-1_CGig	gb|AFTI01003920.1|:22757-22815
NuwaI-1_CGig	gb|AFTI01025057.1|:932-1022
NuwaI-1_CGig	gb|AFTI01023529.1|:13761-13827
NuwaI-1_CGig	gb|AFTI01021553.1|:64668-64738
NuwaI-2_CGig	gb|AFTI01015002.1|:24848-29950
NuwaI-2_CGig	gb|AFTI01023045.1|:12819-17923
NuwaI-2_CGig	gb|AFTI01017987.1|:10041-12035
NuwaI-2_CGig	gb|AFTI01016201.1|:22547-24541
NuwaI-2_CGig	gb|AFTI01008350.1|:7419-11546
NuwaI-2_CGig	gb|AFTI01016202.1|:1-2170
NuwaI-2_CGig	gb|AFTI01000809.1|:10582-15618

NuwaI-2_CGig	gb|AFTI01014037.1|:45769-48437
NuwaI-2_CGig	gb|AFTI01002362.1|:1861-8300
NuwaI-2_CGig	gb|AFTI01016950.1|:12278-15462
NuwaI-2_CGig	gb|AFTI01024809.1|:1602-2252
NuwaI-2_CGig	gb|AFTI01021615.1|:8117-8753
NuwaI-2_CGig	gb|AFTI01001065.1|:657-1246
NuwaI-2_CGig	gb|AFTI01016529.1|:4334-4578
NuwaI-2_CGig	gb|AFTI01029911.1|:3724-4079
NuwaI-2_CGig	gb|AFTI01015611.1|:6095-12509
NuwaI-2_CGig	gb|AFTI01008844.1|:19447-19607
NuwaI-2_CGig	gb|AFTI01015956.1|:62157-62377
NuwaI-2_CGig	gb|AFTI01019507.1|:20009-20163
NuwaI-2_CGig	gb|AFTI01018027.1|:25126-25462
NuwaI-2_CGig	gb|AFTI01010737.1|:12790-12855
NuwaI-2_CGig	gb|AFTI01017435.1|:5157-5313
NuwaI-2_CGig	gb|AFTI01023011.1|:744-809
NuwaI-2_CGig	gb|AFTI01022529.1|:15333-15398
NuwaI-2_CGig	gb|AFTI01022087.1|:5285-5350
NuwaI-2_CGig	gb|AFTI01022059.1|:39659-39724
NuwaI-2_CGig	gb|AFTI01022035.1|:3450-3515
NuwaI-2_CGig	gb|AFTI01018401.1|:3384-3449
NuwaI-2_CGig	gb|AFTI01018247.1|:8233-8298
NuwaI-2_CGig	gb|AFTI01017665.1|:31156-31221
NuwaI-2_CGig	gb|AFTI01017556.1|:33322-33387
NuwaI-2_CGig	gb|AFTI01016915.1|:9364-9429
NuwaI-2_CGig	gb|AFTI01014820.1|:11134-11199
NuwaI-2_CGig	gb|AFTI01013446.1|:13349-13414
NuwaI-2_CGig	gb|AFTI01013213.1|:11323-11388
NuwaI-2_CGig	gb|AFTI01012007.1|:51571-51636
NuwaI-2_CGig	gb|AFTI01011883.1|:115623-115688
NuwaI-2_CGig	gb|AFTI01010833.1|:5957-6022
NuwaI-2_CGig	gb|AFTI01010408.1|:30399-30464
NuwaI-2_CGig	gb|AFTI01007984.1|:15674-15739
NuwaI-2_CGig	gb|AFTI01006616.1|:15438-15503
NuwaI-2_CGig	gb|AFTI01006401.1|:2140-2205
NuwaI-2_CGig	gb|AFTI01004800.1|:3959-4019
NuwaI-2_CGig	gb|AFTI01004800.1|:38468-38533
NuwaI-2_CGig	gb|AFTI01004103.1|:35023-35088
NuwaI-2_CGig	gb|AFTI01004103.1|:2654-2718
NuwaI-2_CGig	gb|AFTI01003412.1|:15046-15111
NuwaI-2_CGig	gb|AFTI01003150.1|:6549-6614
NuwaI-2_CGig	gb|AFTI01001879.1|:413-478
NuwaI-2_CGig	gb|AFTI01001878.1|:5778-5843
NuwaI-2_CGig	gb|AFTI01000415.1|:18479-18544

NuwaI-2_CGig	gb|AFTI01030319.1|:12679-12743
NuwaI-2_CGig	gb|AFTI01012756.1|:13495-13559
NuwaI-2_CGig	gb|AFTI01009689.1|:23789-23853
NuwaI-2_CGig	gb|AFTI01006953.1|:31028-31092
NuwaI-2_CGig	gb|AFTI01028840.1|:156-283
NuwaI-2_CGig	gb|AFTI01008366.1|:4196-4259
NuwaI-2_CGig	gb|AFTI01030334.1|:1775-1837
NuwaI-2_CGig	gb|AFTI01006301.1|:18234-18296
NuwaI-2_CGig	gb|AFTI01005512.1|:22210-22272
NuwaI-2_CGig	gb|AFTI01003237.1|:17940-18095
NuwaI-2_CGig	gb|AFTI01017531.1|:9702-9801
NuwaI-2_CGig	gb|AFTI01017382.1|:23174-23239
NuwaI-2_CGig	gb|AFTI01013034.1|:1776-1841
NuwaI-2_CGig	gb|AFTI01009154.1|:16354-16419
NuwaI-2_CGig	gb|AFTI01007944.1|:46904-46969
NuwaI-2_CGig	gb|AFTI01006691.1|:14262-14327
NuwaI-2_CGig	gb|AFTI01003857.1|:19661-19726
NuwaI-2_CGig	gb|AFTI01003849.1|:245-310
NuwaI-2_CGig	gb|AFTI01002703.1|:17014-17079
NuwaI-2_CGig	gb|AFTI01030147.1|:33013-33077
NuwaI-2_CGig	gb|AFTI01030140.1|:669-733
NuwaI-2_CGig	gb|AFTI01029237.1|:9967-10031
NuwaI-2_CGig	gb|AFTI01028919.1|:4760-4824
NuwaI-2_CGig	gb|AFTI01028567.1|:790-854
NuwaI-2_CGig	gb|AFTI01027721.1|:1174-1238
NuwaI-2_CGig	gb|AFTI01025377.1|:33-97
NuwaI-2_CGig	gb|AFTI01024959.1|:12624-12688
NuwaI-2_CGig	gb|AFTI01024903.1|:4064-4128
NuwaI-2_CGig	gb|AFTI01024757.1|:34537-34601
NuwaI-2_CGig	gb|AFTI01024223.1|:1418-1482
NuwaI-2_CGig	gb|AFTI01022573.1|:5574-5638
NuwaI-2_CGig	gb|AFTI01022463.1|:16002-16066
NuwaI-2_CGig	gb|AFTI01020723.1|:10998-11062
NuwaI-2_CGig	gb|AFTI01019842.1|:1200-1264
NuwaI-2_CGig	gb|AFTI01019280.1|:15848-15912
NuwaI-2_CGig	gb|AFTI01018860.1|:10682-10746
NuwaI-2_CGig	gb|AFTI01018674.1|:8407-8471
NuwaI-2_CGig	gb|AFTI01017799.1|:5350-5414
NuwaI-2_CGig	gb|AFTI01017080.1|:6901-6965
NuwaI-2_CGig	gb|AFTI01016017.1|:2246-2310
NuwaI-2_CGig	gb|AFTI01015523.1|:10148-10212
NuwaI-2_CGig	gb|AFTI01015327.1|:11230-11294
NuwaI-2_CGig	gb|AFTI01015199.1|:9997-10061
NuwaI-2_CGig	gb|AFTI01014152.1|:30638-30702

NuwaI-2_CGig	gb|AFTI01013808.1|:7188-7252
NuwaI-2_CGig	gb|AFTI01013535.1|:24004-24068
NuwaI-2_CGig	gb|AFTI01013160.1|:19632-19696
NuwaI-2_CGig	gb|AFTI01013055.1|:24687-24751
NuwaI-2_CGig	gb|AFTI01012762.1|:5144-5208
NuwaI-2_CGig	gb|AFTI01011212.1|:960-1024
NuwaI-2_CGig	gb|AFTI01010952.1|:20038-20102
NuwaI-2_CGig	gb|AFTI01010418.1|:5385-5449
NuwaI-2_CGig	gb|AFTI01010182.1|:10592-10656
NuwaI-2_CGig	gb|AFTI01009915.1|:7821-7885
NuwaI-2_CGig	gb|AFTI01009811.1|:55916-55980
NuwaI-2_CGig	gb|AFTI01009108.1|:12682-12746
NuwaI-2_CGig	gb|AFTI01008793.1|:21747-21811
NuwaI-2_CGig	gb|AFTI01008766.1|:55506-55570
NuwaI-2_CGig	gb|AFTI01008416.1|:13079-13143
NuwaI-2_CGig	gb|AFTI01007234.1|:143-207
NuwaI-2_CGig	gb|AFTI01006273.1|:29078-29142
NuwaI-2_CGig	gb|AFTI01005289.1|:339-403
NuwaI-2_CGig	gb|AFTI01005282.1|:5942-6006
NuwaI-2_CGig	gb|AFTI01005152.1|:5072-5136
NuwaI-2_CGig	gb|AFTI01004296.1|:2048-2112
NuwaI-2_CGig	gb|AFTI01004141.1|:18174-18238
NuwaI-2_CGig	gb|AFTI01003947.1|:4091-4155
NuwaI-2_CGig	gb|AFTI01003731.1|:32938-33002
NuwaI-2_CGig	gb|AFTI01003212.1|:14803-14867
NuwaI-2_CGig	gb|AFTI01001410.1|:7940-8004
NuwaI-2_CGig	gb|AFTI01001181.1|:19450-19514
NuwaI-2_CGig	gb|AFTI01000193.1|:975-1039
NuwaI-2_CGig	gb|AFTI01029398.1|:15797-15852
NuwaI-2_CGig	gb|AFTI01018347.1|:13099-13159
NuwaI-2_CGig	gb|AFTI01018347.1|:5962-6017
NuwaI-2_CGig	gb|AFTI01015684.1|:11212-11267
NuwaI-2_CGig	gb|AFTI01011565.1|:16391-16450
NuwaI-2_CGig	gb|AFTI01006932.1|:1091-1154
NuwaI-2_CGig	gb|AFTI01004445.1|:65305-65360
NuwaI-2_CGig	gb|AFTI01002827.1|:41726-41789
NuwaI-2_CGig	gb|AFTI01001131.1|:50564-50619
NuwaI-2_CGig	gb|AFTI01030171.1|:13016-13078
NuwaI-2_CGig	gb|AFTI01029226.1|:1434-1495
NuwaI-2_CGig	gb|AFTI01024188.1|:51372-51429
NuwaI-2_CGig	gb|AFTI01019105.1|:29834-29898
NuwaI-2_CGig	gb|AFTI01017881.1|:3828-3892
NuwaI-2_CGig	gb|AFTI01013575.1|:75447-75504
NuwaI-2_CGig	gb|AFTI01011378.1|:2111-2176

NuwaI-2_CGig	gb|AFTI01007636.1|:21354-21418
NuwaI-2_CGig	gb|AFTI01002755.1|:8054-8115
NuwaI-2_CGig	gb|AFTI01030256.1|:14158-14222
NuwaI-2_CGig	gb|AFTI01030099.1|:2675-2739
NuwaI-2_CGig	gb|AFTI01029890.1|:9985-10041
NuwaI-2_CGig	gb|AFTI01029647.1|:47972-48036
NuwaI-2_CGig	gb|AFTI01029303.1|:3730-3794
NuwaI-2_CGig	gb|AFTI01028845.1|:1323-1387
NuwaI-2_CGig	gb|AFTI01028729.1|:5347-5411
NuwaI-2_CGig	gb|AFTI01028055.1|:2024-2088
NuwaI-2_CGig	gb|AFTI01024686.1|:12-76
NuwaI-2_CGig	gb|AFTI01023819.1|:42080-42144
NuwaI-2_CGig	gb|AFTI01022912.1|:52189-52253
NuwaI-2_CGig	gb|AFTI01022273.1|:7839-7903
NuwaI-2_CGig	gb|AFTI01022011.1|:2382-2446
NuwaI-2_CGig	gb|AFTI01020783.1|:4583-4647
NuwaI-2_CGig	gb|AFTI01019578.1|:16949-17013
NuwaI-2_CGig	gb|AFTI01019027.1|:64361-64425
NuwaI-2_CGig	gb|AFTI01018404.1|:5703-5763
NuwaI-2_CGig	gb|AFTI01017404.1|:22310-22374
NuwaI-2_CGig	gb|AFTI01014337.1|:22406-22466
NuwaI-2_CGig	gb|AFTI01014105.1|:8782-8846
NuwaI-2_CGig	gb|AFTI01013967.1|:877-941
NuwaI-2_CGig	gb|AFTI01012729.1|:36983-37047
NuwaI-2_CGig	gb|AFTI01012729.1|:26214-26278
NuwaI-2_CGig	gb|AFTI01012708.1|:11506-11570
NuwaI-2_CGig	gb|AFTI01011862.1|:6728-6792
NuwaI-2_CGig	gb|AFTI01011709.1|:18253-18317
NuwaI-2_CGig	gb|AFTI01010227.1|:25806-25870
NuwaI-2_CGig	gb|AFTI01009495.1|:2593-2657
NuwaI-2_CGig	gb|AFTI01009197.1|:6510-6574
NuwaI-2_CGig	gb|AFTI01007350.1|:5069-5133
NuwaI-2_CGig	gb|AFTI01006804.1|:11302-11366
NuwaI-2_CGig	gb|AFTI01004713.1|:1807-1871
NuwaI-2_CGig	gb|AFTI01003910.1|:29085-29149
NuwaI-2_CGig	gb|AFTI01003838.1|:93-157
NuwaI-2_CGig	gb|AFTI01030145.1|:14023-14078
NuwaI-2_CGig	gb|AFTI01019728.1|:21537-21588
NuwaI-2_CGig	gb|AFTI01015261.1|:18363-18418
NuwaI-2_CGig	gb|AFTI01014157.1|:54978-55120
NuwaI-2_CGig	gb|AFTI01013579.1|:1601-1656
NuwaI-2_CGig	gb|AFTI01005913.1|:10347-10397
NuwaI-2_CGig	gb|AFTI01029777.1|:6182-6244
NuwaI-2_CGig	gb|AFTI01029070.1|:6141-6195

NuwaI-2_CGig	gb|AFTI01029029.1|:2415-2552
NuwaI-2_CGig	gb|AFTI01013078.1|:2783-2837
NuwaI-2_CGig	gb|AFTI01029362.1|:9224-9289
NuwaI-2_CGig	gb|AFTI01018380.1|:56408-56469
NuwaI-2_CGig	gb|AFTI01013971.1|:13167-13232
NuwaI-2_CGig	gb|AFTI01011738.1|:64962-65023
NuwaI-2_CGig	gb|AFTI01009213.1|:17645-17706
NuwaI-2_CGig	gb|AFTI01008405.1|:42155-42216
NuwaI-2_CGig	gb|AFTI01008196.1|:13248-13309
NuwaI-2_CGig	gb|AFTI01028685.1|:1227-1291
NuwaI-2_CGig	gb|AFTI01028437.1|:3666-3730
NuwaI-2_CGig	gb|AFTI01028008.1|:395-459
NuwaI-2_CGig	gb|AFTI01019535.1|:5000-5064
NuwaI-2_CGig	gb|AFTI01018049.1|:2801-2865
NuwaI-2_CGig	gb|AFTI01017016.1|:3749-3813
NuwaI-2_CGig	gb|AFTI01014485.1|:9064-9128
NuwaI-2_CGig	gb|AFTI01014416.1|:13890-13954
NuwaI-2_CGig	gb|AFTI01013666.1|:2071-2135
NuwaI-2_CGig	gb|AFTI01011678.1|:6694-6758
NuwaI-2_CGig	gb|AFTI01010640.1|:41381-41445
NuwaI-2_CGig	gb|AFTI01010257.1|:2307-2371
NuwaI-2_CGig	gb|AFTI01009672.1|:3668-3732
NuwaI-2_CGig	gb|AFTI01008589.1|:6975-7039
NuwaI-2_CGig	gb|AFTI01007398.1|:4522-4586
NuwaI-2_CGig	gb|AFTI01006324.1|:4952-5016
NuwaI-2_CGig	gb|AFTI01004599.1|:5248-5312
NuwaI-2_CGig	gb|AFTI01003970.1|:15188-15252
NuwaI-2_CGig	gb|AFTI01003257.1|:59360-59424
NuwaI-2_CGig	gb|AFTI01002893.1|:29854-29918
NuwaI-2_CGig	gb|AFTI01000421.1|:12480-12544
NuwaI-2_CGig	gb|AFTI01021478.1|:2920-2983
NuwaI-2_CGig	gb|AFTI01008932.1|:3966-4017
NuwaI-2_CGig	gb|AFTI01001403.1|:52438-52493
NuwaI-2_CGig	gb|AFTI01025331.1|:57842-57904
NuwaI-2_CGig	gb|AFTI01020069.1|:11144-11206
NuwaI-2_CGig	gb|AFTI01019956.1|:10165-10227
NuwaI-2_CGig	gb|AFTI01019631.1|:22789-22851
NuwaI-2_CGig	gb|AFTI01017644.1|:1329-1391
NuwaI-2_CGig	gb|AFTI01017393.1|:1383-1449
NuwaI-2_CGig	gb|AFTI01016689.1|:5392-5454
NuwaI-2_CGig	gb|AFTI01016180.1|:6618-6680
NuwaI-2_CGig	gb|AFTI01013636.1|:7665-7727
NuwaI-2_CGig	gb|AFTI01013282.1|:3822-3884
NuwaI-2_CGig	gb|AFTI01010323.1|:60808-60870

NuwaI-2_CGig	gb|AFTI01008673.1|:21363-21425
NuwaI-2_CGig	gb|AFTI01008399.1|:31481-31543
NuwaI-2_CGig	gb|AFTI01005259.1|:5880-5946
NuwaI-2_CGig	gb|AFTI01004450.1|:7065-7127
NuwaI-2_CGig	gb|AFTI01003758.1|:2583-2633
NuwaI-2_CGig	gb|AFTI01002075.1|:902-964
NuwaI-2_CGig	gb|AFTI01001080.1|:28049-28111
NuwaI-2_CGig	gb|AFTI01030413.1|:787-848
NuwaI-2_CGig	gb|AFTI01029210.1|:9394-9455
NuwaI-2_CGig	gb|AFTI01028441.1|:3879-3940
NuwaI-2_CGig	gb|AFTI01028234.1|:2054-2115
NuwaI-2_CGig	gb|AFTI01024884.1|:863-924
NuwaI-2_CGig	gb|AFTI01024867.1|:29736-29797
NuwaI-2_CGig	gb|AFTI01024656.1|:73985-74046
NuwaI-2_CGig	gb|AFTI01024224.1|:12931-12992
NuwaI-2_CGig	gb|AFTI01023684.1|:5734-5795
NuwaI-2_CGig	gb|AFTI01023462.1|:102828-102889
NuwaI-2_CGig	gb|AFTI01023369.1|:8602-8667
NuwaI-2_CGig	gb|AFTI01021822.1|:1853-1914
NuwaI-2_CGig	gb|AFTI01019895.1|:20641-20702
NuwaI-2_CGig	gb|AFTI01018498.1|:8513-8570
NuwaI-2_CGig	gb|AFTI01018291.1|:35922-35983
NuwaI-3_CGig	gb|AFTI01008757.1|:66372-72842
NuwaI-3_CGig	gb|AFTI01001802.1|:16-2528
NuwaI-3_CGig	gb|AFTI01029268.1|:6335-10373
NuwaI-3_CGig	gb|AFTI01014290.1|:1-3518
NuwaI-3_CGig	gb|AFTI01006654.1|:7733-8842
NuwaI-3_CGig	gb|AFTI01004877.1|:19012-27109
NuwaI-3_CGig	gb|AFTI01028414.1|:56-1184
NuwaI-3_CGig	gb|AFTI01028391.1|:1173-2159
NuwaI-3_CGig	gb|AFTI01001803.1|:34683-35897
NuwaI-3_CGig	gb|AFTI01006922.1|:90-1858
NuwaI-3_CGig	gb|AFTI01029863.1|:799-3555
NuwaI-3_CGig	gb|AFTI01014289.1|:1709-2340
NuwaI-3_CGig	gb|AFTI01020728.1|:9903-11150
NuwaI-3_CGig	gb|AFTI01006255.1|:5936-8069
NuwaI-3_CGig	gb|AFTI01020605.1|:50945-53608
NuwaI-3_CGig	gb|AFTI01008350.1|:12686-13329
NuwaI-3_CGig	gb|AFTI01029455.1|:12196-13326
NuwaI-3_CGig	gb|AFTI01025299.1|:14496-14637
NuwaI-3_CGig	gb|AFTI01023805.1|:7280-7421
NuwaI-3_CGig	gb|AFTI01013028.1|:20382-20523
NuwaI-3_CGig	gb|AFTI01021729.1|:4125-4267
NuwaI-3_CGig	gb|AFTI01000196.1|:10409-10550

NuwaI-3_CGig	gb|AFTI01005607.1|:23052-23191
NuwaI-3_CGig	gb|AFTI01004450.1|:19404-19544
NuwaI-3_CGig	gb|AFTI01012288.1|:24689-24826
NuwaI-3_CGig	gb|AFTI01002018.1|:18590-18730
NuwaI-3_CGig	gb|AFTI01013767.1|:2367-2509
NuwaI-3_CGig	gb|AFTI01024607.1|:2722-2862
NuwaI-3_CGig	gb|AFTI01024138.1|:4261-4401
NuwaI-3_CGig	gb|AFTI01016882.1|:644-784
NuwaI-3_CGig	gb|AFTI01010590.1|:73411-73551
NuwaI-3_CGig	gb|AFTI01006365.1|:9684-9825
NuwaI-3_CGig	gb|AFTI01004300.1|:6975-7115
NuwaI-3_CGig	gb|AFTI01025234.1|:26515-26817
NuwaI-3_CGig	gb|AFTI01001804.1|:321-3039
NuwaI-3_CGig	gb|AFTI01008400.1|:20784-20925
NuwaI-3_CGig	gb|AFTI01024120.1|:77458-77598
NuwaI-3_CGig	gb|AFTI01021591.1|:9874-10015
NuwaI-3_CGig	gb|AFTI01021532.1|:58853-58993
NuwaI-3_CGig	gb|AFTI01012019.1|:27591-27731
NuwaI-3_CGig	gb|AFTI01008861.1|:144-284
NuwaI-3_CGig	gb|AFTI01001462.1|:20623-20759
NuwaI-3_CGig	gb|AFTI01021337.1|:939-1075
NuwaI-3_CGig	gb|AFTI01016065.1|:5557-5696
NuwaI-3_CGig	gb|AFTI01004875.1|:5524-5671
NuwaI-3_CGig	gb|AFTI01004216.1|:43708-43848
NuwaI-3_CGig	gb|AFTI01009934.1|:6991-7132
NuwaI-3_CGig	gb|AFTI01019732.1|:3025-3164
NuwaI-3_CGig	gb|AFTI01016676.1|:17117-17257
NuwaI-3_CGig	gb|AFTI01029311.1|:6158-6304
NuwaI-3_CGig	gb|AFTI01011430.1|:5028-5174
NuwaI-3_CGig	gb|AFTI01024415.1|:3216-3345
NuwaI-3_CGig	gb|AFTI01019080.1|:25690-25816
NuwaI-3_CGig	gb|AFTI01017515.1|:4296-4420
NuwaI-3_CGig	gb|AFTI01011774.1|:12484-12619
NuwaI-3_CGig	gb|AFTI01015561.1|:14140-14268
NuwaI-3_CGig	gb|AFTI01017435.1|:27492-27632
NuwaI-3_CGig	gb|AFTI01029979.1|:14161-14295
NuwaI-3_CGig	gb|AFTI01017671.1|:28498-28644
NuwaI-3_CGig	gb|AFTI01008995.1|:11135-11281
NuwaI-3_CGig	gb|AFTI01009892.1|:16651-16787
NuwaI-3_CGig	gb|AFTI01023838.1|:25544-25681
NuwaI-3_CGig	gb|AFTI01016383.1|:10920-11067
NuwaI-3_CGig	gb|AFTI01024403.1|:2488-2634
NuwaI-3_CGig	gb|AFTI01020092.1|:10795-10937
NuwaI-3_CGig	gb|AFTI01000868.1|:30836-30963

NuwaI-3_CGig	gb|AFTI01024401.1|:41302-41450
NuwaI-3_CGig	gb|AFTI01017090.1|:22419-22557
NuwaI-3_CGig	gb|AFTI01021338.1|:1555-1704
NuwaI-3_CGig	gb|AFTI01000409.1|:5012-5155
NuwaI-3_CGig	gb|AFTI01029802.1|:222-348
NuwaI-3_CGig	gb|AFTI01022541.1|:855-981
NuwaI-3_CGig	gb|AFTI01022431.1|:19205-19331
NuwaI-3_CGig	gb|AFTI01021370.1|:115706-115832
NuwaI-3_CGig	gb|AFTI01009796.1|:26671-26797
NuwaI-3_CGig	gb|AFTI01004154.1|:24833-24959
NuwaI-3_CGig	gb|AFTI01004280.1|:1435-1562
NuwaI-3_CGig	gb|AFTI01030163.1|:18314-18440
NuwaI-3_CGig	gb|AFTI01024116.1|:1026-1118
NuwaI-3_CGig	gb|AFTI01006330.1|:29013-29988
NuwaI-3_CGig	gb|AFTI01000849.1|:37250-37377
NuwaI-3_CGig	gb|AFTI01002309.1|:6732-6858
NuwaI-3_CGig	gb|AFTI01011853.1|:473-569
NuwaI-3_CGig	gb|AFTI01022301.1|:47056-47194
NuwaI-3_CGig	gb|AFTI01029934.1|:41717-41849
NuwaI-3_CGig	gb|AFTI01010043.1|:7214-7360
NuwaI-3_CGig	gb|AFTI01017311.1|:2463-2614
NuwaI-3_CGig	gb|AFTI01011852.1|:36016-36104
NuwaI-3_CGig	gb|AFTI01008414.1|:22531-22622
NuwaI-3_CGig	gb|AFTI01018071.1|:4695-4797
NuwaI-3_CGig	gb|AFTI01017728.1|:961-1075
NuwaI-3_CGig	gb|AFTI01004722.1|:5400-5514
NuwaI-3_CGig	gb|AFTI01021780.1|:21795-21908
NuwaI-3_CGig	gb|AFTI01019039.1|:2787-2880
NuwaI-3_CGig	gb|AFTI01013924.1|:15509-15641
NuwaI-3_CGig	gb|AFTI01029689.1|:54402-54470
NuwaI-3_CGig	gb|AFTI01008963.1|:4631-4762
NuwaI-3_CGig	gb|AFTI01017122.1|:6172-6303
NuwaI-3_CGig	gb|AFTI01022612.1|:23244-23367
NuwaI-3_CGig	gb|AFTI01030272.1|:103356-103451
NuwaI-3_CGig	gb|AFTI01017187.1|:7386-7478
NuwaI-3_CGig	gb|AFTI01023953.1|:17589-17722
NuwaI-3_CGig	gb|AFTI01000360.1|:2058-3538
NuwaI-3_CGig	gb|AFTI01024406.1|:40048-40126
NuwaI-3_CGig	gb|AFTI01029008.1|:3713-3831
NuwaI-3_CGig	gb|AFTI01021185.1|:49510-49636
NuwaI-3_CGig	gb|AFTI01012095.1|:107179-107265
NuwaI-3_CGig	gb|AFTI01019478.1|:8644-8740
NuwaI-3_CGig	gb|AFTI01007206.1|:3404-3516
NuwaI-3_CGig	gb|AFTI01008494.1|:6057-6172

NuwaI-3_CGig	gb|AFTI01025121.1|:5115-5166
NuwaI-3_CGig	gb|AFTI01024142.1|:16645-16720
NuwaI-3_CGig	gb|AFTI01002051.1|:2232-2281
NuwaI-3_CGig	gb|AFTI01002154.1|:26931-26994
NuwaI-3_CGig	gb|AFTI01002311.1|:15124-15214
NuwaI-4_CGig	gb|AFTI01015697.1|:36736-46268
NuwaI-4_CGig	gb|AFTI01004892.1|:16776-16956
NuwaI-4_CGig	gb|AFTI01004892.1|:15762-17922
NuwaI-4_CGig	gb|AFTI01015597.1|:1314-5815
NuwaI-4_CGig	gb|AFTI01016082.1|:24069-27455
NuwaI-4_CGig	gb|AFTI01014298.1|:8731-10508
NuwaI-4_CGig	gb|AFTI01025053.1|:1-1864
NuwaI-4_CGig	gb|AFTI01014209.1|:1-2440
NuwaI-4_CGig	gb|AFTI01014209.1|:772-953
NuwaI-4_CGig	gb|AFTI01005443.1|:7486-9421
NuwaI-4_CGig	gb|AFTI01025054.1|:1-4957
NuwaI-4_CGig	gb|AFTI01025054.1|:3861-3956
NuwaI-4_CGig	gb|AFTI01025309.1|:11689-12729
NuwaI-4_CGig	gb|AFTI01023856.1|:14565-15593
NuwaI-4_CGig	gb|AFTI01011042.1|:18905-26539
NuwaI-4_CGig	gb|AFTI01010358.1|:21267-24043
NuwaI-4_CGig	gb|AFTI01003745.1|:31682-32769
NuwaI-4_CGig	gb|AFTI01009810.1|:5797-6418
NuwaI-4_CGig	gb|AFTI01009998.1|:2316-4519
NuwaI-4_CGig	gb|AFTI01024593.1|:11988-14209
NuwaI-4_CGig	gb|AFTI01013386.1|:14-954
NuwaI-4_CGig	gb|AFTI01009450.1|:30876-31395
NuwaI-4_CGig	gb|AFTI01010692.1|:1-931
NuwaI-4_CGig	gb|AFTI01011953.1|:4732-5370
NuwaI-4_CGig	gb|AFTI01011045.1|:9495-10487
NuwaI-4_CGig	gb|AFTI01018375.1|:5827-8166
NuwaI-4_CGig	gb|AFTI01010691.1|:13962-14710
NuwaI-4_CGig	gb|AFTI01005248.1|:1-689
NuwaI-4_CGig	gb|AFTI01005499.1|:1-969
NuwaI-4_CGig	gb|AFTI01013354.1|:1-692
NuwaI-4_CGig	gb|AFTI01003746.1|:1-736
NuwaI-4_CGig	gb|AFTI01019802.1|:35-516
NuwaI-4_CGig	gb|AFTI01019133.1|:2259-2991
NuwaI-4_CGig	gb|AFTI01019801.1|:57614-58366
NuwaI-4_CGig	gb|AFTI01021720.1|:40982-41748
NuwaI-4_CGig	gb|AFTI01007234.1|:38312-41863
NuwaI-4_CGig	gb|AFTI01023553.1|:72586-73237
NuwaI-4_CGig	gb|AFTI01023553.1|:50016-50887
NuwaI-4_CGig	gb|AFTI01005247.1|:1014-1713

NuwaI-4_CGig	gb|AFTI01024379.1|:3286-4289
NuwaI-4_CGig	gb|AFTI01012802.1|:2538-3111
NuwaI-4_CGig	gb|AFTI01022553.1|:27178-27462
NuwaI-4_CGig	gb|AFTI01018293.1|:4893-7394
NuwaI-4_CGig	gb|AFTI01007857.1|:13219-13767
NuwaI-4_CGig	gb|AFTI01008039.1|:47986-48106
NuwaI-4_CGig	gb|AFTI01008039.1|:35838-36742
NuwaI-4_CGig	gb|AFTI01001722.1|:19221-19772
NuwaI-4_CGig	gb|AFTI01024318.1|:32987-33564
NuwaI-4_CGig	gb|AFTI01021193.1|:605-1337
NuwaI-4_CGig	gb|AFTI01020520.1|:85473-85573
NuwaI-4_CGig	gb|AFTI01017409.1|:19202-19749
NuwaI-4_CGig	gb|AFTI01010332.1|:48745-49297
NuwaI-4_CGig	gb|AFTI01009944.1|:5-212
NuwaI-4_CGig	gb|AFTI01004865.1|:32037-32609
NuwaI-4_CGig	gb|AFTI01000922.1|:98-727
NuwaI-4_CGig	gb|AFTI01028231.1|:2383-3154
NuwaI-4_CGig	gb|AFTI01023526.1|:97557-97611
NuwaI-4_CGig	gb|AFTI01023526.1|:66162-67056
NuwaI-4_CGig	gb|AFTI01017511.1|:6095-6175
NuwaI-4_CGig	gb|AFTI01017511.1|:16587-17481
NuwaI-4_CGig	gb|AFTI01009608.1|:5119-6167
NuwaI-4_CGig	gb|AFTI01008530.1|:14031-15853
NuwaI-4_CGig	gb|AFTI01006798.1|:7284-8173
NuwaI-4_CGig	gb|AFTI01005000.1|:81-973
NuwaI-4_CGig	gb|AFTI01006997.1|:14105-14991
NuwaI-4_CGig	gb|AFTI01030353.1|:18306-18464
NuwaI-4_CGig	gb|AFTI01006528.1|:5018-5740
NuwaI-4_CGig	gb|AFTI01001094.1|:4501-4665
NuwaI-4_CGig	gb|AFTI01001094.1|:44875-45605
NuwaI-4_CGig	gb|AFTI01029340.1|:10859-11746
NuwaI-4_CGig	gb|AFTI01023087.1|:12428-13161
NuwaI-4_CGig	gb|AFTI01018029.1|:109013-109745
NuwaI-4_CGig	gb|AFTI01016993.1|:8236-8443
NuwaI-4_CGig	gb|AFTI01015258.1|:46810-47708
NuwaI-4_CGig	gb|AFTI01013076.1|:32973-34195
NuwaI-4_CGig	gb|AFTI01012786.1|:5552-5759
NuwaI-4_CGig	gb|AFTI01008646.1|:86-820
NuwaI-4_CGig	gb|AFTI01028014.1|:26511-27075
NuwaI-4_CGig	gb|AFTI01015886.1|:40705-41605
NuwaI-4_CGig	gb|AFTI01015886.1|:93676-93762
NuwaI-4_CGig	gb|AFTI01010940.1|:11082-11966
NuwaI-4_CGig	gb|AFTI01008984.1|:7523-8096
NuwaI-4_CGig	gb|AFTI01029558.1|:11798-12554

NuwaI-4_CGig	gb|AFTI01014006.1|:19123-20329
NuwaI-4_CGig	gb|AFTI01014006.1|:12671-12737
NuwaI-4_CGig	gb|AFTI01003277.1|:32580-33454
NuwaI-4_CGig	gb|AFTI01015716.1|:19108-19311
NuwaI-4_CGig	gb|AFTI01015021.1|:22298-23280
NuwaI-4_CGig	gb|AFTI01013709.1|:24009-25415
NuwaI-4_CGig	gb|AFTI01010315.1|:9118-9682
NuwaI-4_CGig	gb|AFTI01029512.1|:14-221
NuwaI-4_CGig	gb|AFTI01021893.1|:7394-7729
NuwaI-4_CGig	gb|AFTI01017356.1|:12-218
NuwaI-4_CGig	gb|AFTI01016953.1|:14169-15029
NuwaI-4_CGig	gb|AFTI01015110.1|:56241-56449
NuwaI-4_CGig	gb|AFTI01015110.1|:51322-52058
NuwaI-4_CGig	gb|AFTI01015110.1|:1062-1147
NuwaI-4_CGig	gb|AFTI01014299.1|:10868-11414
NuwaI-4_CGig	gb|AFTI01004330.1|:3819-4716
NuwaI-4_CGig	gb|AFTI01029801.1|:33432-34303
NuwaI-4_CGig	gb|AFTI01024995.1|:19026-19402
NuwaI-4_CGig	gb|AFTI01023438.1|:17963-18169
NuwaI-4_CGig	gb|AFTI01021121.1|:14-220
NuwaI-4_CGig	gb|AFTI01020097.1|:25778-26659
NuwaI-4_CGig	gb|AFTI01019630.1|:5881-5967
NuwaI-4_CGig	gb|AFTI01017847.1|:8486-9371
NuwaI-4_CGig	gb|AFTI01016208.1|:9494-10035
NuwaI-4_CGig	gb|AFTI01015284.1|:25641-25820
NuwaI-4_CGig	gb|AFTI01015284.1|:1710-2599
NuwaI-4_CGig	gb|AFTI01015105.1|:15834-16392
NuwaI-4_CGig	gb|AFTI01015001.1|:38348-38554
NuwaI-4_CGig	gb|AFTI01013964.1|:187-397
NuwaI-4_CGig	gb|AFTI01009074.1|:6321-7217
NuwaI-4_CGig	gb|AFTI01007621.1|:459-1346
NuwaI-4_CGig	gb|AFTI01004239.1|:17011-17217
NuwaI-4_CGig	gb|AFTI01013254.1|:7171-7538
NuwaI-4_CGig	gb|AFTI01012680.1|:35122-35841
NuwaI-4_CGig	gb|AFTI01012095.1|:2699-3496
NuwaI-4_CGig	gb|AFTI01011729.1|:29603-30356
NuwaI-4_CGig	gb|AFTI01025529.1|:1-203
NuwaI-4_CGig	gb|AFTI01023202.1|:10080-10288
NuwaI-4_CGig	gb|AFTI01021740.1|:43315-44181
NuwaI-4_CGig	gb|AFTI01020939.1|:60108-60194
NuwaI-4_CGig	gb|AFTI01020316.1|:46451-46647
NuwaI-4_CGig	gb|AFTI01019890.1|:26699-27265
NuwaI-4_CGig	gb|AFTI01018764.1|:23372-24747
NuwaI-4_CGig	gb|AFTI01018764.1|:52153-52240

NuwaI-4_CGig	gb|AFTI01017447.1|:3521-4436
NuwaI-4_CGig	gb|AFTI01014529.1|:2276-3173
NuwaI-4_CGig	gb|AFTI01014067.1|:23376-23940
NuwaI-4_CGig	gb|AFTI01012322.1|:16628-16836
NuwaI-4_CGig	gb|AFTI01012322.1|:69-155
NuwaI-4_CGig	gb|AFTI01004864.1|:38554-38762
NuwaI-4_CGig	gb|AFTI01004690.1|:2830-3725
NuwaI-4_CGig	gb|AFTI01000829.1|:39913-40989
NuwaI-4_CGig	gb|AFTI01018210.1|:300-503
NuwaI-4_CGig	gb|AFTI01003035.1|:12633-13525
NuwaI-4_CGig	gb|AFTI01030154.1|:3857-4063
NuwaI-4_CGig	gb|AFTI01024985.1|:18168-18374
NuwaI-4_CGig	gb|AFTI01022457.1|:11057-11950
NuwaI-4_CGig	gb|AFTI01020116.1|:6189-6565
NuwaI-4_CGig	gb|AFTI01019302.1|:3507-3792
NuwaI-4_CGig	gb|AFTI01015609.1|:123111-123188
NuwaI-4_CGig	gb|AFTI01015609.1|:128597-128973
NuwaI-4_CGig	gb|AFTI01015609.1|:6266-6470
NuwaI-4_CGig	gb|AFTI01014554.1|:3474-4199
NuwaI-4_CGig	gb|AFTI01006099.1|:3333-3709
NuwaI-4_CGig	gb|AFTI01001792.1|:22524-23091
NuwaI-4_CGig	gb|AFTI01001242.1|:6389-7606
NuwaI-4_CGig	gb|AFTI01024247.1|:24018-24915
NuwaI-4_CGig	gb|AFTI01023587.1|:27323-27524
NuwaI-4_CGig	gb|AFTI01023129.1|:51367-51450
NuwaI-4_CGig	gb|AFTI01023129.1|:27637-28562
NuwaI-4_CGig	gb|AFTI01023129.1|:13512-14450
NuwaI-4_CGig	gb|AFTI01022351.1|:4541-5440
NuwaI-4_CGig	gb|AFTI01018138.1|:37-902
NuwaI-4_CGig	gb|AFTI01017691.1|:4016-4914
NuwaI-4_CGig	gb|AFTI01017032.1|:51361-52255
NuwaI-4_CGig	gb|AFTI01014089.1|:3168-4063
NuwaI-4_CGig	gb|AFTI01013829.1|:3167-3368
NuwaI-4_CGig	gb|AFTI01013470.1|:8140-8896
NuwaI-4_CGig	gb|AFTI01013037.1|:15169-16064
NuwaI-4_CGig	gb|AFTI01012149.1|:38523-39403
NuwaI-4_CGig	gb|AFTI01012149.1|:22949-23853
NuwaI-4_CGig	gb|AFTI01011872.1|:47589-48483
NuwaI-4_CGig	gb|AFTI01009280.1|:37951-38506
NuwaI-4_CGig	gb|AFTI01008699.1|:2113-3089
NuwaI-4_CGig	gb|AFTI01005585.1|:33940-34835
NuwaI-4_CGig	gb|AFTI01029811.1|:17792-18362
NuwaI-4_CGig	gb|AFTI01028647.1|:3789-4357
NuwaI-4_CGig	gb|AFTI01023849.1|:2840-3409

NuwaI-4_CGig	gb|AFTI01023080.1|:10-218
NuwaI-4_CGig	gb|AFTI01021803.1|:33492-34393
NuwaI-4_CGig	gb|AFTI01021786.1|:19893-20461
NuwaI-4_CGig	gb|AFTI01020770.1|:79472-80345
NuwaI-4_CGig	gb|AFTI01019831.1|:7-215
NuwaI-4_CGig	gb|AFTI01017633.1|:358-565
NuwaI-4_CGig	gb|AFTI01017476.1|:74135-74704
NuwaI-4_CGig	gb|AFTI01015653.1|:45840-46415
NuwaI-4_CGig	gb|AFTI01015016.1|:28707-29274
NuwaI-4_CGig	gb|AFTI01014088.1|:4871-5438
NuwaI-4_CGig	gb|AFTI01010355.1|:440-1013
NuwaI-4_CGig	gb|AFTI01030308.1|:68954-69841
NuwaI-4_CGig	gb|AFTI01024471.1|:7774-7923
NuwaI-4_CGig	gb|AFTI01024471.1|:33739-33938
NuwaI-4_CGig	gb|AFTI01016294.1|:41293-42039
NuwaI-4_CGig	gb|AFTI01013234.1|:321-523
NuwaI-4_CGig	gb|AFTI01012414.1|:10548-10753
NuwaI-4_CGig	gb|AFTI01010721.1|:11106-11992
NuwaI-4_CGig	gb|AFTI01006379.1|:5762-6138
NuwaI-4_CGig	gb|AFTI01030139.1|:5251-6135
NuwaI-4_CGig	gb|AFTI01028013.1|:40382-40587
NuwaI-4_CGig	gb|AFTI01018987.1|:38635-39210
NuwaI-4_CGig	gb|AFTI01016120.1|:12786-13850
NuwaI-4_CGig	gb|AFTI01014851.1|:10820-10904
NuwaI-4_CGig	gb|AFTI01014851.1|:36410-37309
NuwaI-4_CGig	gb|AFTI01014157.1|:58766-58843
NuwaI-4_CGig	gb|AFTI01014157.1|:28616-29911
NuwaI-4_CGig	gb|AFTI01012254.1|:3723-4622
NuwaI-4_CGig	gb|AFTI01006998.1|:9722-9923
NuwaI-4_CGig	gb|AFTI01006737.1|:32583-33471
NuwaI-4_CGig	gb|AFTI01003021.1|:28-229
NuwaI-4_CGig	gb|AFTI01030044.1|:240-448
NuwaI-4_CGig	gb|AFTI01029296.1|:4473-5538
NuwaI-4_CGig	gb|AFTI01023898.1|:17782-18351
NuwaI-4_CGig	gb|AFTI01020130.1|:34558-35628
NuwaI-4_CGig	gb|AFTI01018635.1|:8641-8727
NuwaI-4_CGig	gb|AFTI01017700.1|:71591-72657
NuwaI-4_CGig	gb|AFTI01017564.1|:7310-7877
NuwaI-4_CGig	gb|AFTI01017366.1|:45278-45485
NuwaI-4_CGig	gb|AFTI01013431.1|:12893-13100
NuwaI-4_CGig	gb|AFTI01011327.1|:7-215
NuwaI-4_CGig	gb|AFTI01010570.1|:4468-4676
NuwaI-4_CGig	gb|AFTI01010365.1|:17699-18618
NuwaI-4_CGig	gb|AFTI01009188.1|:4602-4801

NuwaI-4_CGig	gb|AFTI01006592.1|:55395-56287
NuwaI-4_CGig	gb|AFTI01003926.1|:19859-20754
NuwaI-4_CGig	gb|AFTI01028925.1|:104-1003
NuwaI-4_CGig	gb|AFTI01028371.1|:3009-3764
NuwaI-4_CGig	gb|AFTI01024878.1|:3328-4386
NuwaI-4_CGig	gb|AFTI01024560.1|:55334-55905
NuwaI-4_CGig	gb|AFTI01024262.1|:15430-16330
NuwaI-4_CGig	gb|AFTI01021939.1|:7453-8337
NuwaI-4_CGig	gb|AFTI01020847.1|:12264-13002
NuwaI-4_CGig	gb|AFTI01014773.1|:9704-10768
NuwaI-4_CGig	gb|AFTI01012127.1|:14263-14473
NuwaI-4_CGig	gb|AFTI01011783.1|:2736-3299
NuwaI-4_CGig	gb|AFTI01010318.1|:44657-45557
NuwaI-4_CGig	gb|AFTI01010318.1|:1332-1417
NuwaI-4_CGig	gb|AFTI01006290.1|:6474-7038
NuwaI-4_CGig	gb|AFTI01004327.1|:22979-24047
NuwaI-4_CGig	gb|AFTI01029743.1|:18356-19414
NuwaI-4_CGig	gb|AFTI01027462.1|:496-872
NuwaI-4_CGig	gb|AFTI01025094.1|:9194-9406
NuwaI-4_CGig	gb|AFTI01024783.1|:18474-18850
NuwaI-4_CGig	gb|AFTI01020660.1|:21355-22235
NuwaI-4_CGig	gb|AFTI01019268.1|:3942-4148
NuwaI-4_CGig	gb|AFTI01019028.1|:25750-26646
NuwaI-4_CGig	gb|AFTI01018972.1|:61055-62102
NuwaI-4_CGig	gb|AFTI01010061.1|:109-659
NuwaI-4_CGig	gb|AFTI01009173.1|:22244-22446
NuwaI-4_CGig	gb|AFTI01009106.1|:78876-79082
NuwaI-4_CGig	gb|AFTI01009010.1|:369-571
NuwaI-4_CGig	gb|AFTI01006222.1|:3398-4298
NuwaI-4_CGig	gb|AFTI01005049.1|:55376-56083
NuwaI-4_CGig	gb|AFTI01003920.1|:49544-49920
NuwaI-4_CGig	gb|AFTI01000176.1|:41054-41123
NuwaI-4_CGig	gb|AFTI01000176.1|:44480-44856
NuwaI-4_CGig	gb|AFTI01030215.1|:24594-24985
NuwaI-4_CGig	gb|AFTI01024645.1|:2427-2631
NuwaI-4_CGig	gb|AFTI01022996.1|:4288-4867
NuwaI-4_CGig	gb|AFTI01022784.1|:7347-8247
NuwaI-4_CGig	gb|AFTI01015398.1|:40399-41294
NuwaI-4_CGig	gb|AFTI01014431.1|:16147-17037
NuwaI-4_CGig	gb|AFTI01008508.1|:31959-32522
NuwaI-4_CGig	gb|AFTI01004147.1|:5345-6240
NuwaI-4_CGig	gb|AFTI01003714.1|:15667-16717
NuwaI-4_CGig	gb|AFTI01024114.1|:13833-14891
NuwaI-4_CGig	gb|AFTI01023043.1|:15666-16443

	NuwaI-4_CGig	gb|AFTI01018615.1|:48229-48799
	NuwaI-4_CGig	gb|AFTI01018615.1|:63615-63702
	NuwaI-4_CGig	gb|AFTI01017093.1|:15658-15978
	NuwaI-4_CGig	gb|AFTI01008405.1|:37755-38337
	NuwaI-4_CGig	gb|AFTI01004800.1|:24596-24882
	NuwaI-4_CGig	gb|AFTI01004800.1|:11179-11919
	NuwaI-4_CGig	gb|AFTI01003560.1|:7373-8477
	NuwaI-4_CGig	gb|AFTI01020137.1|:17672-18241
	NuwaI-4_CGig	gb|AFTI01015059.1|:10955-11277
	NuwaI-4_CGig	gb|AFTI01015005.1|:46666-46877
	NuwaI-4_CGig	gb|AFTI01015005.1|:11626-12217
	NuwaI-4_CGig	gb|AFTI01014996.1|:5230-5437
	NuwaI-4_CGig	gb|AFTI01013701.1|:1715-2776
	NuwaI-4_CGig	gb|AFTI01013701.1|:41255-41942
	NuwaI-4_CGig	gb|AFTI01011156.1|:27479-28045
	NuwaI-4_CGig	gb|AFTI01008949.1|:12395-12974
	NuwaI-4_CGig	gb|AFTI01006970.1|:48380-48962
	NuwaI-4_CGig	gb|AFTI01006970.1|:14799-14871
	NuwaI-4_CGig	gb|AFTI01005388.1|:3657-4559
	NuwaI-4_CGig	gb|AFTI01004868.1|:6930-7136
	NuwaI-4_CGig	gb|AFTI01004838.1|:11273-12015
Danio rerio	*NuwaI-1_DRer*	emb|CABZ01045000.1|:17875-19618
	NuwaI-1_DRer	emb|CABZ01001887.1|:2982-5362
	NuwaI-1_DRer	emb|CABZ01001887.1|:3097-3164
	NuwaI-1_DRer	emb|CABZ01054974.1|:32430-32497
	NuwaI-1_DRer	emb|CABZ01054974.1|:30195-32614
	NuwaI-1_DRer	emb|CABZ01062533.1|:5104-7523
	NuwaI-1_DRer	emb|CABZ01062533.1|:5221-5288
	NuwaI-1_DRer	emb|CABZ01060459.1|:309-2729
	NuwaI-1_DRer	emb|CABZ01060459.1|:426-493
	NuwaI-1_DRer	emb|CABZ01016350.1|:20773-23182
	NuwaI-1_DRer	emb|CABZ01016350.1|:20890-20957
	NuwaI-1_DRer	emb|CABZ01023848.1|:23022-24799
	NuwaI-1_DRer	emb|CABZ01029056.1|:13969-14036
	NuwaI-1_DRer	emb|CABZ01029056.1|:11733-14153
	NuwaI-1_DRer	emb|CABZ01027988.1|:22947-24272
	NuwaI-1_DRer	emb|CABZ01009018.1|:760-3187
	NuwaI-1_DRer	emb|CABZ01009018.1|:877-944
	NuwaI-1_DRer	emb|CABZ01071341.1|:3121-3188
	NuwaI-1_DRer	emb|CABZ01071341.1|:859-3305
	NuwaI-1_DRer	emb|CABZ01060819.1|:17958-18025
	NuwaI-1_DRer	emb|CABZ01060819.1|:15717-18147
	NuwaI-1_DRer	emb|CABZ01015055.1|:3528-5164
	NuwaI-1_DRer	emb|CABZ01069583.1|:3570-3637

NuwaI-1_DRer	emb|CABZ01069583.1|:1332-3754
NuwaI-1_DRer	emb|CABZ01015920.1|:24155-24222
NuwaI-1_DRer	emb|CABZ01015920.1|:21919-24339
NuwaI-1_DRer	emb|CABZ01005866.1|:13743-13810
NuwaI-1_DRer	emb|CABZ01005866.1|:11503-13927
NuwaI-1_DRer	emb|CABZ01002017.1|:45092-47513
NuwaI-1_DRer	emb|CABZ01002017.1|:45209-45276
NuwaI-1_DRer	emb|CABZ01050684.1|:8005-8072
NuwaI-1_DRer	emb|CABZ01050684.1|:6301-8189
NuwaI-1_DRer	emb|CABZ01006468.1|:24928-27136
NuwaI-1_DRer	emb|CABZ01006468.1|:25045-25112
NuwaI-1_DRer	emb|CABZ01073611.1|:44835-44902
NuwaI-1_DRer	emb|CABZ01073611.1|:42597-45019
NuwaI-1_DRer	emb|CABZ01052789.1|:2491-2558
NuwaI-1_DRer	emb|CABZ01052789.1|:623-2675
NuwaI-1_DRer	emb|CABZ01034283.1|:20413-22575
NuwaI-1_DRer	emb|CABZ01034283.1|:20530-20597
NuwaI-1_DRer	emb|CABZ01058692.1|:9606-16588
NuwaI-1_DRer	emb|CABZ01058692.1|:7368-9790
NuwaI-1_DRer	emb|CABZ01040373.1|:17525-17592
NuwaI-1_DRer	emb|CABZ01040373.1|:15289-17702
NuwaI-1_DRer	emb|CABZ01036761.1|:22578-22645
NuwaI-1_DRer	emb|CABZ01036761.1|:20302-22762
NuwaI-1_DRer	emb|CABZ01027320.1|:15386-15453
NuwaI-1_DRer	emb|CABZ01027320.1|:13171-15570
NuwaI-1_DRer	emb|CABZ01035747.1|:20985-21052
NuwaI-1_DRer	emb|CABZ01035747.1|:18735-21169
NuwaI-1_DRer	emb|CABZ01014746.1|:14113-14180
NuwaI-1_DRer	emb|CABZ01014746.1|:11888-14297
NuwaI-1_DRer	emb|CABZ01069331.1|:9706-9773
NuwaI-1_DRer	emb|CABZ01069331.1|:7473-9890
NuwaI-1_DRer	emb|CABZ01077546.1|:189-1510
NuwaI-1_DRer	emb|CABZ01077546.1|:20558-22964
NuwaI-1_DRer	emb|CABZ01077546.1|:20675-20740
NuwaI-1_DRer	emb|CABZ01042839.1|:103-2526
NuwaI-1_DRer	emb|CABZ01042839.1|:220-284
NuwaI-1_DRer	emb|CABZ01000480.1|:6176-6243
NuwaI-1_DRer	emb|CABZ01000480.1|:3932-6360
NuwaI-1_DRer	emb|CABZ01044530.1|:9586-11961
NuwaI-1_DRer	emb|CABZ01044530.1|:9703-9770
NuwaI-1_DRer	emb|CABZ01042381.1|:20715-20782
NuwaI-1_DRer	emb|CABZ01042381.1|:18475-20899
NuwaI-1_DRer	emb|CABZ01021656.1|:28488-28555
NuwaI-1_DRer	emb|CABZ01021656.1|:43202-43343

NuwaI-1_DRer	emb|CABZ01021656.1|:41694-42382
NuwaI-1_DRer	emb|CABZ01021656.1|:26262-28672
NuwaI-1_DRer	emb|CABZ01013466.1|:17010-19424
NuwaI-1_DRer	emb|CABZ01013466.1|:17127-17194
NuwaI-1_DRer	emb|CABZ01002692.1|:64562-64629
NuwaI-1_DRer	emb|CABZ01002692.1|:62338-64746
NuwaI-1_DRer	emb|CABZ01037173.1|:23865-26311
NuwaI-1_DRer	emb|CABZ01037173.1|:23982-24049
NuwaI-1_DRer	emb|CABZ01079295.1|:4620-4687
NuwaI-1_DRer	emb|CABZ01079295.1|:2380-4804
NuwaI-1_DRer	emb|CABZ01059863.1|:25224-27614
NuwaI-1_DRer	emb|CABZ01059863.1|:25341-25408
NuwaI-1_DRer	emb|CABZ01039056.1|:25230-25297
NuwaI-1_DRer	emb|CABZ01039056.1|:22994-25414
NuwaI-1_DRer	emb|CABZ01000479.1|:6829-6896
NuwaI-1_DRer	emb|CABZ01000479.1|:2884-7013
NuwaI-1_DRer	emb|CABZ01079242.1|:38424-40877
NuwaI-1_DRer	emb|CABZ01079242.1|:38541-38608
NuwaI-1_DRer	emb|CABZ01077884.1|:13974-16392
NuwaI-1_DRer	emb|CABZ01077884.1|:14091-14158
NuwaI-1_DRer	emb|CABZ01070045.1|:19415-21836
NuwaI-1_DRer	emb|CABZ01070045.1|:19532-19599
NuwaI-1_DRer	emb|CABZ01035322.1|:23505-23572
NuwaI-1_DRer	emb|CABZ01035322.1|:21285-23689
NuwaI-1_DRer	emb|CABZ01015261.1|:27558-29979
NuwaI-1_DRer	emb|CABZ01015261.1|:27675-27742
NuwaI-1_DRer	emb|CABZ01002101.1|:14803-14870
NuwaI-1_DRer	emb|CABZ01002101.1|:12844-14987
NuwaI-1_DRer	emb|CABZ01000448.1|:5577-5644
NuwaI-1_DRer	emb|CABZ01000448.1|:3331-5761
NuwaI-1_DRer	emb|CABZ01077574.1|:20722-23146
NuwaI-1_DRer	emb|CABZ01077574.1|:20839-20906
NuwaI-1_DRer	emb|CABZ01059045.1|:3384-5427
NuwaI-1_DRer	emb|CABZ01059045.1|:3501-3568
NuwaI-1_DRer	emb|CABZ01043961.1|:13572-15982
NuwaI-1_DRer	emb|CABZ01043961.1|:13689-13756
NuwaI-1_DRer	emb|CABZ01039880.1|:22919-25342
NuwaI-1_DRer	emb|CABZ01039880.1|:23036-23103
NuwaI-1_DRer	emb|CABZ01015032.1|:3344-5752
NuwaI-1_DRer	emb|CABZ01015032.1|:3461-3528
NuwaI-1_DRer	emb|CABZ01004380.1|:29-2442
NuwaI-1_DRer	emb|CABZ01004380.1|:146-209
NuwaI-1_DRer	emb|CABZ01027989.1|:1338-1405
NuwaI-1_DRer	emb|CABZ01027989.1|:1-1522

NuwaI-1_DRer	emb|CABZ01066463.1|:13678-16112
NuwaI-1_DRer	emb|CABZ01066463.1|:13795-13862
NuwaI-1_DRer	emb|CABZ01060687.1|:43260-43327
NuwaI-1_DRer	emb|CABZ01060687.1|:41011-43444
NuwaI-1_DRer	emb|CABZ01073430.1|:17828-17895
NuwaI-1_DRer	emb|CABZ01073430.1|:16321-18012
NuwaI-1_DRer	emb|CABZ01019356.1|:24189-26185
NuwaI-1_DRer	emb|CABZ01019356.1|:24306-24373
NuwaI-1_DRer	emb|CABZ01075191.1|:28201-30622
NuwaI-1_DRer	emb|CABZ01075191.1|:28318-28385
NuwaI-1_DRer	emb|CABZ01060089.1|:121-2554
NuwaI-1_DRer	emb|CABZ01060089.1|:238-305
NuwaI-1_DRer	emb|CABZ01049310.1|:103736-106177
NuwaI-1_DRer	emb|CABZ01049310.1|:103853-103920
NuwaI-1_DRer	emb|CABZ01040375.1|:13169-13236
NuwaI-1_DRer	emb|CABZ01040375.1|:10943-13353
NuwaI-1_DRer	emb|CABZ01104612.1|:1-1814
NuwaI-1_DRer	emb|CABZ01070468.1|:30018-32433
NuwaI-1_DRer	emb|CABZ01070468.1|:30135-30202
NuwaI-1_DRer	emb|CABZ01050748.1|:12415-12482
NuwaI-1_DRer	emb|CABZ01050748.1|:10169-12599
NuwaI-1_DRer	emb|CABZ01060110.1|:4241-6660
NuwaI-1_DRer	emb|CABZ01060110.1|:4358-4425
NuwaI-1_DRer	emb|CABZ01117584.1|:2978-5147
NuwaI-1_DRer	emb|CABZ01069544.1|:2570-4965
NuwaI-1_DRer	emb|CABZ01069544.1|:2687-2754
NuwaI-1_DRer	emb|CABZ01025183.1|:9495-9562
NuwaI-1_DRer	emb|CABZ01025183.1|:8836-9679
NuwaI-1_DRer	emb|CABZ01015655.1|:91193-94020
NuwaI-1_DRer	emb|CABZ01015655.1|:91310-91377
NuwaI-1_DRer	emb|CABZ01028852.1|:3583-5983
NuwaI-1_DRer	emb|CABZ01028852.1|:3700-3767
NuwaI-1_DRer	emb|CABZ01054688.1|:45365-47791
NuwaI-1_DRer	emb|CABZ01054688.1|:45482-45549
NuwaI-1_DRer	emb|CABZ01023841.1|:41280-41347
NuwaI-1_DRer	emb|CABZ01023841.1|:39050-41464
NuwaI-1_DRer	emb|CABZ01018185.1|:1126-3544
NuwaI-1_DRer	emb|CABZ01018185.1|:1243-1310
NuwaI-1_DRer	emb|CABZ01111462.1|:5419-6872
NuwaI-1_DRer	emb|CABZ01021616.1|:17132-19543
NuwaI-1_DRer	emb|CABZ01021616.1|:17249-17316
NuwaI-1_DRer	emb|CABZ01036358.1|:17937-20351
NuwaI-1_DRer	emb|CABZ01036358.1|:18054-18121
NuwaI-1_DRer	emb|CABZ01016180.1|:80222-82655

NuwaI-1_DRer	emb|CABZ01016180.1|:80339-80402
NuwaI-1_DRer	emb|CABZ01006517.1|:12995-13062
NuwaI-1_DRer	emb|CABZ01006517.1|:10786-13179
NuwaI-1_DRer	emb|CABZ01061269.1|:5339-7473
NuwaI-1_DRer	emb|CABZ01061269.1|:5456-5523
NuwaI-1_DRer	emb|CABZ01058175.1|:22698-22765
NuwaI-1_DRer	emb|CABZ01058175.1|:20459-22882
NuwaI-1_DRer	emb|CABZ01057567.1|:5366-5433
NuwaI-1_DRer	emb|CABZ01057567.1|:3130-5550
NuwaI-1_DRer	emb|CABZ01047939.1|:14519-16923
NuwaI-1_DRer	emb|CABZ01047939.1|:14636-14699
NuwaI-1_DRer	emb|CABZ01025690.1|:51096-53335
NuwaI-1_DRer	emb|CABZ01025690.1|:63778-63907
NuwaI-1_DRer	emb|CABZ01025690.1|:51213-51280
NuwaI-1_DRer	emb|CABZ01079633.1|:8420-8487
NuwaI-1_DRer	emb|CABZ01079633.1|:6193-8604
NuwaI-1_DRer	emb|CABZ01048794.1|:19932-19999
NuwaI-1_DRer	emb|CABZ01048794.1|:17707-20116
NuwaI-1_DRer	emb|CABZ01113073.1|:2034-4440
NuwaI-1_DRer	emb|CABZ01113073.1|:2151-2218
NuwaI-1_DRer	emb|CABZ01069926.1|:2000-2067
NuwaI-1_DRer	emb|CABZ01069926.1|:259-2184
NuwaI-1_DRer	emb|CABZ01031629.1|:17484-17551
NuwaI-1_DRer	emb|CABZ01031629.1|:15368-21114
NuwaI-1_DRer	emb|CABZ01007852.1|:33628-35626
NuwaI-1_DRer	emb|CABZ01007852.1|:33749-33812
NuwaI-1_DRer	emb|CABZ01075716.1|:6412-6479
NuwaI-1_DRer	emb|CABZ01075716.1|:4185-6596
NuwaI-1_DRer	emb|CABZ01008644.1|:39327-41717
NuwaI-1_DRer	emb|CABZ01008644.1|:39444-39511
NuwaI-1_DRer	emb|CABZ01067894.1|:35473-35540
NuwaI-1_DRer	emb|CABZ01067894.1|:33252-35657
NuwaI-1_DRer	emb|CABZ01020591.1|:41379-43798
NuwaI-1_DRer	emb|CABZ01020591.1|:41496-41563
NuwaI-1_DRer	emb|CABZ01007604.1|:11408-11475
NuwaI-1_DRer	emb|CABZ01007604.1|:9164-11592
NuwaI-1_DRer	emb|CABZ01074879.1|:5358-7945
NuwaI-1_DRer	emb|CABZ01074879.1|:6172-6239
NuwaI-1_DRer	emb|CABZ01044383.1|:2869-2936
NuwaI-1_DRer	emb|CABZ01044383.1|:667-3053
NuwaI-1_DRer	emb|CABZ01013827.1|:1-2209
NuwaI-1_DRer	emb|CABZ01013825.1|:5-1441
NuwaI-1_DRer	emb|CABZ01001257.1|:6384-6451
NuwaI-1_DRer	emb|CABZ01001257.1|:4144-6568

NuwaI-1_DRer	emb|CABZ01005655.1|:1927-4332
NuwaI-1_DRer	emb|CABZ01005655.1|:2044-2111
NuwaI-1_DRer	emb|CABZ01075378.1|:17589-19955
NuwaI-1_DRer	emb|CABZ01075378.1|:17706-17772
NuwaI-1_DRer	emb|CABZ01070850.1|:15890-18302
NuwaI-1_DRer	emb|CABZ01070850.1|:16007-16074
NuwaI-1_DRer	emb|CABZ01049508.1|:10291-10358
NuwaI-1_DRer	emb|CABZ01049508.1|:8048-10475
NuwaI-1_DRer	emb|CABZ01015415.1|:3905-6322
NuwaI-1_DRer	emb|CABZ01015415.1|:4022-4089
NuwaI-1_DRer	emb|CABZ01073091.1|:2857-2924
NuwaI-1_DRer	emb|CABZ01073091.1|:957-3041
NuwaI-1_DRer	emb|CABZ01026489.1|:14030-14097
NuwaI-1_DRer	emb|CABZ01026489.1|:11866-14214
NuwaI-1_DRer	emb|CABZ01056157.1|:2390-4625
NuwaI-1_DRer	emb|CABZ01056157.1|:2507-2574
NuwaI-1_DRer	emb|CABZ01071500.1|:673-2307
NuwaI-1_DRer	emb|CABZ01077545.1|:29377-30170
NuwaI-1_DRer	emb|CABZ01077545.1|:29494-29559
NuwaI-1_DRer	emb|CABZ01054590.1|:37101-37168
NuwaI-1_DRer	emb|CABZ01054590.1|:34856-37285
NuwaI-1_DRer	emb|CABZ01099136.1|:2684-2751
NuwaI-1_DRer	emb|CABZ01099136.1|:441-2868
NuwaI-1_DRer	emb|CABZ01048483.1|:30501-30568
NuwaI-1_DRer	emb|CABZ01048483.1|:28257-30685
NuwaI-1_DRer	emb|CABZ01048324.1|:13291-13358
NuwaI-1_DRer	emb|CABZ01048324.1|:11047-13475
NuwaI-1_DRer	emb|CABZ01040132.1|:5299-5366
NuwaI-1_DRer	emb|CABZ01040132.1|:3046-5483
NuwaI-1_DRer	emb|CABZ01095804.1|:586-653
NuwaI-1_DRer	emb|CABZ01095804.1|:11-770
NuwaI-1_DRer	emb|CABZ01015056.1|:638-705
NuwaI-1_DRer	emb|CABZ01015056.1|:90-822
NuwaI-1_DRer	emb|CABZ01026030.1|:41335-41402
NuwaI-1_DRer	emb|CABZ01026030.1|:39081-41519
NuwaI-1_DRer	emb|CABZ01045075.1|:842-3267
NuwaI-1_DRer	emb|CABZ01045075.1|:959-1026
NuwaI-1_DRer	emb|CABZ01023047.1|:26042-28468
NuwaI-1_DRer	emb|CABZ01023047.1|:26159-26226
NuwaI-1_DRer	emb|CABZ01082540.1|:1007-1631
NuwaI-1_DRer	emb|CABZ01082540.1|:1124-1191
NuwaI-1_DRer	emb|CABZ01013626.1|:31023-33430
NuwaI-1_DRer	emb|CABZ01013626.1|:31140-31207
NuwaI-1_DRer	emb|CABZ01117585.1|:428-495

NuwaI-1_DRer	emb|CABZ01117585.1|:5-612
NuwaI-1_DRer	emb|CABZ01078626.1|:5674-8164
NuwaI-1_DRer	emb|CABZ01078626.1|:5791-5858
NuwaI-1_DRer	emb|CABZ01010689.1|:3399-3466
NuwaI-1_DRer	emb|CABZ01010689.1|:1206-3583
NuwaI-1_DRer	emb|CABZ01046423.1|:8018-8085
NuwaI-1_DRer	emb|CABZ01046423.1|:5472-8202
NuwaI-1_DRer	emb|CABZ01013004.1|:2836-5249
NuwaI-1_DRer	emb|CABZ01013004.1|:2953-3018
NuwaI-1_DRer	emb|CABZ01065696.1|:13934-16403
NuwaI-1_DRer	emb|CABZ01065696.1|:14051-14118
NuwaI-1_DRer	emb|CABZ01052544.1|:19083-19150
NuwaI-1_DRer	emb|CABZ01052544.1|:12573-19267
NuwaI-1_DRer	emb|CABZ01035305.1|:85-1530
NuwaI-1_DRer	emb|CABZ01035305.1|:202-266
NuwaI-1_DRer	emb|CABZ01023194.1|:64284-65596
NuwaI-1_DRer	emb|CABZ01073938.1|:11161-11227
NuwaI-1_DRer	emb|CABZ01073938.1|:8892-11344
NuwaI-1_DRer	emb|CABZ01054394.1|:42970-43037
NuwaI-1_DRer	emb|CABZ01054394.1|:41038-43154
NuwaI-1_DRer	emb|CABZ01056156.1|:11919-11986
NuwaI-1_DRer	emb|CABZ01056156.1|:9697-12103
NuwaI-1_DRer	emb|CABZ01053494.1|:6080-6147
NuwaI-1_DRer	emb|CABZ01053494.1|:3805-6264
NuwaI-1_DRer	emb|CABZ01045001.1|:160-1490
NuwaI-1_DRer	emb|CABZ01002719.1|:18841-18908
NuwaI-1_DRer	emb|CABZ01002719.1|:16691-19023
NuwaI-1_DRer	emb|CABZ01016395.1|:53868-59570
NuwaI-1_DRer	emb|CABZ01052546.1|:322-1274
NuwaI-1_DRer	emb|CABZ01021339.1|:81-776
NuwaI-1_DRer	emb|CABZ01001184.1|:8057-16703
NuwaI-1_DRer	emb|CABZ01001184.1|:8174-8241
NuwaI-1_DRer	emb|CABZ01049453.1|:16125-16192
NuwaI-1_DRer	emb|CABZ01049453.1|:14086-16200
NuwaI-1_DRer	emb|CABZ01045605.1|:1421-2793
NuwaI-1_DRer	emb|CABZ01054658.1|:528-1452
NuwaI-1_DRer	emb|CABZ01029953.1|:3183-3250
NuwaI-1_DRer	emb|CABZ01029953.1|:1004-3357
NuwaI-1_DRer	emb|CABZ01049727.1|:4751-4818
NuwaI-1_DRer	emb|CABZ01049727.1|:2990-4935
NuwaI-1_DRer	emb|CABZ01062498.1|:157-888
NuwaI-1_DRer	emb|CABZ01047829.1|:15885-17952
NuwaI-1_DRer	emb|CABZ01047829.1|:16002-16069
NuwaI-1_DRer	emb|CABZ01058563.1|:1-748

NuwaI-1_DRer	emb|CABZ01052408.1|:1096-3658
NuwaI-1_DRer	emb|CABZ01052408.1|:1213-1280
NuwaI-1_DRer	emb|CABZ01004379.1|:4623-4690
NuwaI-1_DRer	emb|CABZ01004379.1|:2478-4690
NuwaI-1_DRer	emb|CABZ01003847.1|:103-1026
NuwaI-1_DRer	emb|CABZ01069925.1|:28256-29192
NuwaI-1_DRer	emb|CABZ01076986.1|:17234-17301
NuwaI-1_DRer	emb|CABZ01076986.1|:15026-17411
NuwaI-1_DRer	emb|CABZ01118797.1|:4081-4140
NuwaI-1_DRer	emb|CABZ01118797.1|:3299-4257
NuwaI-1_DRer	emb|CABZ01000489.1|:1-1913
NuwaI-1_DRer	emb|CABZ01007830.1|:15256-17325
NuwaI-1_DRer	emb|CABZ01007830.1|:15373-15440
NuwaI-1_DRer	emb|CABZ01047336.1|:12390-12457
NuwaI-1_DRer	emb|CABZ01047336.1|:10189-12575
NuwaI-1_DRer	emb|CABZ01035509.1|:6341-6408
NuwaI-1_DRer	emb|CABZ01035509.1|:4145-6525
NuwaI-1_DRer	emb|CABZ01034284.1|:7459-9132
NuwaI-1_DRer	emb|CABZ01071499.1|:49473-50205
NuwaI-1_DRer	emb|CABZ01071499.1|:49590-49657
NuwaI-1_DRer	emb|CABZ01009515.1|:5450-5517
NuwaI-1_DRer	emb|CABZ01009515.1|:4402-5634
NuwaI-1_DRer	emb|CABZ01049305.1|:25576-27963
NuwaI-1_DRer	emb|CABZ01049305.1|:25693-25757
NuwaI-1_DRer	emb|CABZ01025618.1|:12905-12972
NuwaI-1_DRer	emb|CABZ01025618.1|:10753-13089
NuwaI-1_DRer	emb|CABZ01088985.1|:2585-3321
NuwaI-1_DRer	emb|CABZ01036404.1|:2453-5388
NuwaI-1_DRer	emb|CABZ01036404.1|:2566-2624
NuwaI-1_DRer	emb|CABZ01034643.1|:27285-30478
NuwaI-1_DRer	emb|CABZ01034643.1|:27402-27469
NuwaI-1_DRer	emb|CABZ01059046.1|:1-304
NuwaI-1_DRer	emb|CABZ01095276.1|:563-1708
NuwaI-1_DRer	emb|CABZ01086719.1|:7577-7973
NuwaI-1_DRer	emb|CABZ01062497.1|:29709-30928
NuwaI-1_DRer	emb|CABZ01062497.1|:29826-29893
NuwaI-1_DRer	emb|CABZ01058562.1|:5988-8101
NuwaI-1_DRer	emb|CABZ01058562.1|:6105-6172
NuwaI-1_DRer	emb|CABZ01027451.1|:2122-3020
NuwaI-1_DRer	emb|CABZ01049117.1|:9895-11303
NuwaI-1_DRer	emb|CABZ01049117.1|:10010-10077
NuwaI-1_DRer	emb|CABZ01052545.1|:25153-26581
NuwaI-1_DRer	emb|CABZ01052545.1|:25270-25337
NuwaI-1_DRer	emb|CABZ01003846.1|:17401-20100

NuwaI-1_DRer	emb|CABZ01060161.1|:18812-20777
NuwaI-1_DRer	emb|CABZ01060161.1|:18929-18996
NuwaI-1_DRer	emb|CABZ01085757.1|:6225-8886
NuwaI-1_DRer	emb|CABZ01038371.1|:2704-4307
NuwaI-1_DRer	emb|CABZ01028468.1|:4919-7506
NuwaI-1_DRer	emb|CABZ01028468.1|:5037-5103
NuwaI-1_DRer	emb|CABZ01048291.1|:23283-25402
NuwaI-1_DRer	emb|CABZ01034644.1|:1483-2329
NuwaI-1_DRer	emb|CABZ01028469.1|:64-1325
NuwaI-1_DRer	emb|CABZ01002176.1|:14049-15117
NuwaI-1_DRer	emb|CABZ01011492.1|:22688-23466
NuwaI-1_DRer	emb|CABZ01053925.1|:8378-8445
NuwaI-1_DRer	emb|CABZ01053925.1|:7500-8561
NuwaI-1_DRer	emb|CABZ01038369.1|:22080-23712
NuwaI-1_DRer	emb|CABZ01003093.1|:163-1246
NuwaI-1_DRer	emb|CABZ01049118.1|:136-1020
NuwaI-1_DRer	emb|CABZ01035306.1|:1737-2997
NuwaI-1_DRer	emb|CABZ01027039.1|:9929-11300
NuwaI-1_DRer	emb|CABZ01010799.1|:53118-54623
NuwaI-1_DRer	emb|CABZ01042090.1|:2265-5146
NuwaI-1_DRer	emb|CABZ01115611.1|:1094-1647
NuwaI-1_DRer	emb|CABZ01086192.1|:5918-5985
NuwaI-1_DRer	emb|CABZ01086192.1|:5089-6102
NuwaI-1_DRer	emb|CABZ01012834.1|:41483-43390
NuwaI-1_DRer	emb|CABZ01081417.1|:10067-10134
NuwaI-1_DRer	emb|CABZ01081417.1|:9767-10251
NuwaI-1_DRer	emb|CABZ01045760.1|:12629-12914
NuwaI-1_DRer	emb|CABZ01016112.1|:8372-8657
NuwaI-1_DRer	emb|CABZ01043034.1|:14077-14835
NuwaI-1_DRer	emb|CABZ01043034.1|:14194-14261
NuwaI-1_DRer	emb|CABZ01056831.1|:9759-9943
NuwaI-1_DRer	emb|CABZ01056831.1|:9876-9943
NuwaI-1_DRer	emb|CABZ01054514.1|:1630-2069
NuwaI-1_DRer	emb|CABZ01001311.1|:17868-18611
NuwaI-1_DRer	emb|CABZ01001311.1|:21225-21703
NuwaI-1_DRer	emb|CABZ01032644.1|:15728-18576
NuwaI-1_DRer	emb|CABZ01003096.1|:23064-23729
NuwaI-1_DRer	emb|CABZ01087471.1|:1393-3057
NuwaI-1_DRer	emb|CABZ01087471.1|:1503-1568
NuwaI-1_DRer	emb|CABZ01051460.1|:39348-40156
NuwaI-1_DRer	emb|CABZ01087143.1|:8985-9180
NuwaI-1_DRer	emb|CABZ01049856.1|:7296-9250
NuwaI-1_DRer	emb|CABZ01043444.1|:54779-56857
NuwaI-1_DRer	emb|CABZ01021338.1|:480-1395

NuwaI-1_DRer	emb|CABZ01013536.1|:9870-11310
NuwaI-1_DRer	emb|CABZ01013536.1|:9974-10038
NuwaI-1_DRer	emb|CABZ01054269.1|:11261-11328
NuwaI-1_DRer	emb|CABZ01054269.1|:10950-11450
NuwaI-1_DRer	emb|CABZ01018109.1|:14690-16541
NuwaI-1_DRer	emb|CABZ01055812.1|:2599-3665
NuwaI-1_DRer	emb|CABZ01055812.1|:2710-2769
NuwaI-1_DRer	emb|CABZ01044993.1|:1431-1498
NuwaI-1_DRer	emb|CABZ01044993.1|:81-1604
NuwaI-1_DRer	emb|CABZ01051223.1|:6583-10353
NuwaI-1_DRer	emb|CABZ01013232.1|:2668-4845
NuwaI-1_DRer	emb|CABZ01009970.1|:26160-29985
NuwaI-1_DRer	emb|CABZ01009970.1|:26274-26341
NuwaI-1_DRer	emb|CABZ01029236.1|:8536-10150
NuwaI-1_DRer	emb|CABZ01005831.1|:1259-1323
NuwaI-1_DRer	emb|CABZ01005831.1|:1259-1438
NuwaI-1_DRer	emb|CABZ01057653.1|:11512-12702
NuwaI-1_DRer	emb|CABZ01027450.1|:8132-8289
NuwaI-1_DRer	emb|CABZ01023722.1|:182-244
NuwaI-1_DRer	emb|CABZ01023722.1|:5-387
NuwaI-1_DRer	emb|CABZ01007383.1|:30369-30887
NuwaI-1_DRer	emb|CABZ01007383.1|:47432-47631
NuwaI-1_DRer	emb|CABZ01063612.1|:18394-19454
NuwaI-1_DRer	emb|CABZ01030477.1|:5282-6590
NuwaI-1_DRer	emb|CABZ01030477.1|:5392-5445
NuwaI-1_DRer	emb|CABZ01009243.1|:28148-28350
NuwaI-1_DRer	emb|CABZ01074242.1|:53-193
NuwaI-1_DRer	emb|CABZ01042103.1|:1188-1393
NuwaI-1_DRer	emb|CABZ01005767.1|:22794-23534
NuwaI-1_DRer	emb|CABZ01118626.1|:972-1201
NuwaI-1_DRer	emb|CABZ01038436.1|:21699-23578
NuwaI-1_DRer	emb|CABZ01016218.1|:30908-31441
NuwaI-1_DRer	emb|CABZ01038382.1|:74971-77778
NuwaI-1_DRer	emb|CABZ01036946.1|:6621-7266
NuwaI-1_DRer	emb|CABZ01063857.1|:457-2169
NuwaI-1_DRer	emb|CABZ01039488.1|:13403-14228
NuwaI-1_DRer	emb|CABZ01012846.1|:26725-27146
NuwaI-1_DRer	emb|CABZ01001967.1|:3927-4644
NuwaI-1_DRer	emb|CABZ01065501.1|:28961-29133
NuwaI-1_DRer	emb|CABZ01084966.1|:724-951
NuwaI-1_DRer	emb|CABZ01084966.1|:782-846
NuwaI-1_DRer	emb|CABZ01078959.1|:3020-3180
NuwaI-1_DRer	emb|CABZ01050344.1|:1828-3027
NuwaI-1_DRer	emb|CABZ01040931.1|:54192-55393

NuwaI-1_DRer	emb|CABZ01006801.1|:387-547
NuwaI-1_DRer	emb|CABZ01022176.1|:411-1044
NuwaI-1_DRer	emb|CABZ01022174.1|:15912-16164
NuwaI-2_DRer	emb|CABZ01002837.1|:41655-45166
NuwaI-2_DRer	emb|CABZ01002838.1|:12249-17741
NuwaI-2_DRer	emb|CABZ01034645.1|:15425-18921
NuwaI-2_DRer	emb|CABZ01042099.1|:20255-24745
NuwaI-2_DRer	emb|CABZ01042099.1|:8803-9950
NuwaI-2_DRer	emb|CABZ01085684.1|:5732-9186
NuwaI-2_DRer	emb|CABZ01056313.1|:16719-19992
NuwaI-2_DRer	emb|CABZ01001742.1|:23987-35183
NuwaI-2_DRer	emb|CABZ01046457.1|:2545-4358
NuwaI-2_DRer	emb|CABZ01007910.1|:7509-8902
NuwaI-2_DRer	emb|CABZ01054094.1|:11786-12518
NuwaI-2_DRer	emb|CABZ01033283.1|:18022-18743
NuwaI-2_DRer	emb|CABZ01033450.1|:22664-23811
NuwaI-2_DRer	emb|CABZ01015604.1|:12724-13439
NuwaI-2_DRer	emb|CABZ01075029.1|:17410-18143
NuwaI-2_DRer	emb|CABZ01014855.1|:85873-86607
NuwaI-2_DRer	emb|CABZ01005639.1|:2319-3763
NuwaI-2_DRer	emb|CABZ01061452.1|:19476-25557
NuwaI-2_DRer	emb|CABZ01035241.1|:19927-20298
NuwaI-2_DRer	emb|CABZ01052688.1|:6389-6983
NuwaI-2_DRer	emb|CABZ01037270.1|:8827-9958
NuwaI-2_DRer	emb|CABZ01003322.1|:6138-6863
NuwaI-2_DRer	emb|CABZ01003322.1|:27023-27310
NuwaI-2_DRer	emb|CABZ01052027.1|:57068-57798
NuwaI-2_DRer	emb|CABZ01095854.1|:558-1286
NuwaI-2_DRer	emb|CABZ01035052.1|:9032-9756
NuwaI-2_DRer	emb|CABZ01032137.1|:21272-22407
NuwaI-2_DRer	emb|CABZ01011872.1|:51367-52495
NuwaI-2_DRer	emb|CABZ01001735.1|:4689-5895
NuwaI-2_DRer	emb|CABZ01042992.1|:5944-7021
NuwaI-2_DRer	emb|CABZ01075457.1|:11330-12482
NuwaI-2_DRer	emb|CABZ01049094.1|:16269-16996
NuwaI-2_DRer	emb|CABZ01035660.1|:29068-30207
NuwaI-2_DRer	emb|CABZ01004537.1|:41071-41963
NuwaI-2_DRer	emb|CABZ01063887.1|:6632-7775
NuwaI-2_DRer	emb|CABZ01032776.1|:8946-10064
NuwaI-2_DRer	emb|CABZ01027101.1|:2463-3391
NuwaI-2_DRer	emb|CABZ01016226.1|:71511-73132
NuwaI-2_DRer	emb|CABZ01016226.1|:30007-32559
NuwaI-2_DRer	emb|CABZ01015996.1|:49357-50501
NuwaI-2_DRer	emb|CABZ01015996.1|:35844-36990

NuwaI-2_DRer	emb|CABZ01060331.1|:11636-21880
NuwaI-2_DRer	emb|CABZ01056215.1|:32005-32737
NuwaI-2_DRer	emb|CABZ01026537.1|:10308-11452
NuwaI-2_DRer	emb|CABZ01009802.1|:3989-5131
NuwaI-2_DRer	emb|CABZ01076192.1|:1800-2527
NuwaI-2_DRer	emb|CABZ01058629.1|:24402-25546
NuwaI-2_DRer	emb|CABZ01085639.1|:6803-9261
NuwaI-2_DRer	emb|CABZ01060161.1|:11681-12400
NuwaI-2_DRer	emb|CABZ01050457.1|:15624-16694
NuwaI-2_DRer	emb|CABZ01026886.1|:45407-47653
NuwaI-2_DRer	emb|CABZ01056518.1|:17466-18500
NuwaI-2_DRer	emb|CABZ01041767.1|:1170-2311
NuwaI-2_DRer	emb|CABZ01041207.1|:4651-5789
NuwaI-2_DRer	emb|CABZ01007099.1|:4675-5412
NuwaI-2_DRer	emb|CABZ01004534.1|:2646-3723
NuwaI-2_DRer	emb|CABZ01078157.1|:2083-3227
NuwaI-2_DRer	emb|CABZ01008621.1|:11398-12093
NuwaI-2_DRer	emb|CABZ01026301.1|:2824-3188
NuwaI-2_DRer	emb|CABZ01014124.1|:1844-2574
NuwaI-2_DRer	emb|CABZ01003936.1|:5685-6818
NuwaI-2_DRer	emb|CABZ01094628.1|:332-1476
NuwaI-2_DRer	emb|CABZ01022310.1|:9968-10144
NuwaI-2_DRer	emb|CABZ01022310.1|:41636-42780
NuwaI-2_DRer	emb|CABZ01019513.1|:5623-6478
NuwaI-2_DRer	emb|CABZ01013909.1|:28699-29839
NuwaI-2_DRer	emb|CABZ01009502.1|:4908-6049
NuwaI-2_DRer	emb|CABZ01087942.1|:2614-3751
NuwaI-2_DRer	emb|CABZ01052094.1|:23473-23983
NuwaI-2_DRer	emb|CABZ01052094.1|:22397-23387
NuwaI-2_DRer	emb|CABZ01051092.1|:3071-4073
NuwaI-2_DRer	emb|CABZ01050348.1|:12353-13484
NuwaI-2_DRer	emb|CABZ01043846.1|:6040-6754
NuwaI-2_DRer	emb|CABZ01041086.1|:96829-97970
NuwaI-2_DRer	emb|CABZ01039183.1|:18149-19298
NuwaI-2_DRer	emb|CABZ01026397.1|:14060-14272
NuwaI-2_DRer	emb|CABZ01026397.1|:30273-31411
NuwaI-2_DRer	emb|CABZ01021023.1|:3360-4489
NuwaI-2_DRer	emb|CABZ01090133.1|:8476-9619
NuwaI-2_DRer	emb|CABZ01055655.1|:33765-34897
NuwaI-2_DRer	emb|CABZ01052986.1|:13503-14042
NuwaI-2_DRer	emb|CABZ01035162.1|:20955-22114
NuwaI-2_DRer	emb|CABZ01032265.1|:15291-16167
NuwaI-2_DRer	emb|CABZ01032265.1|:129503-129867
NuwaI-2_DRer	emb|CABZ01067460.1|:12286-13420

NuwaI-2_DRer	emb|CABZ01040288.1|:4968-5337
NuwaI-2_DRer	emb|CABZ01036498.1|:10770-11334
NuwaI-2_DRer	emb|CABZ01036498.1|:44311-44753
NuwaI-2_DRer	emb|CABZ01023026.1|:11367-12504
NuwaI-2_DRer	emb|CABZ01018380.1|:22568-22931
NuwaI-2_DRer	emb|CABZ01001330.1|:11254-12723
NuwaI-2_DRer	emb|CABZ01076371.1|:3996-5145
NuwaI-2_DRer	emb|CABZ01059716.1|:16152-17270
NuwaI-2_DRer	emb|CABZ01050672.1|:19588-20725
NuwaI-2_DRer	emb|CABZ01037942.1|:10047-11472
NuwaI-2_DRer	emb|CABZ01011502.1|:7734-8474
NuwaI-2_DRer	emb|CABZ01010049.1|:22210-23353
NuwaI-2_DRer	emb|CABZ01010049.1|:577-1380
NuwaI-2_DRer	emb|CABZ01055774.1|:9929-11071
NuwaI-2_DRer	emb|CABZ01023468.1|:13146-14172
NuwaI-2_DRer	emb|CABZ01022407.1|:20048-21191
NuwaI-2_DRer	emb|CABZ01007726.1|:66965-68107
NuwaI-2_DRer	emb|CABZ01051066.1|:2535-3666
NuwaI-2_DRer	emb|CABZ01035037.1|:16430-17455
NuwaI-2_DRer	emb|CABZ01029286.1|:338-1486
NuwaI-2_DRer	emb|CABZ01026761.1|:48674-49822
NuwaI-2_DRer	emb|CABZ01012305.1|:40627-41899
NuwaI-2_DRer	emb|CABZ01012305.1|:32431-32935
NuwaI-2_DRer	emb|CABZ01011770.1|:1998-3128
NuwaI-2_DRer	emb|CABZ01073756.1|:3388-4528
NuwaI-2_DRer	emb|CABZ01061022.1|:27134-27791
NuwaI-2_DRer	emb|CABZ01018841.1|:12889-13845
NuwaI-2_DRer	emb|CABZ01009152.1|:28213-29350
NuwaI-2_DRer	emb|CABZ01022989.1|:10443-11586
NuwaI-2_DRer	emb|CABZ01008074.1|:3748-4925
NuwaI-2_DRer	emb|CABZ01084774.1|:35657-36798
NuwaI-2_DRer	emb|CABZ01078154.1|:6057-7208
NuwaI-2_DRer	emb|CABZ01051662.1|:27160-28284
NuwaI-2_DRer	emb|CABZ01040864.1|:1586-2595
NuwaI-2_DRer	emb|CABZ01033970.1|:51956-53105
NuwaI-2_DRer	emb|CABZ01029703.1|:1796-2926
NuwaI-2_DRer	emb|CABZ01029446.1|:19717-20867
NuwaI-2_DRer	emb|CABZ01028773.1|:4095-4381
NuwaI-2_DRer	emb|CABZ01028773.1|:30518-31667
NuwaI-2_DRer	emb|CABZ01015320.1|:18473-19610
NuwaI-2_DRer	emb|CABZ01011955.1|:9724-10841
NuwaI-2_DRer	emb|CABZ01011955.1|:58964-59925
NuwaI-2_DRer	emb|CABZ01004692.1|:94454-95610
NuwaI-2_DRer	emb|CABZ01003184.1|:23300-24395

NuwaI-2_DRer	emb|CABZ01003184.1|:1659-3349
NuwaI-2_DRer	emb|CABZ01000786.1|:35786-36908
NuwaI-2_DRer	emb|CABZ01081727.1|:5842-6987
NuwaI-2_DRer	emb|CABZ01046998.1|:11474-12579
NuwaI-2_DRer	emb|CABZ01046998.1|:21138-22286
NuwaI-2_DRer	emb|CABZ01017840.1|:15246-16387
NuwaI-2_DRer	emb|CABZ01005862.1|:1141-2318
NuwaI-2_DRer	emb|CABZ01075990.1|:7889-9002
NuwaI-2_DRer	emb|CABZ01074351.1|:651-1794
NuwaI-2_DRer	emb|CABZ01027543.1|:931-1669
NuwaI-2_DRer	emb|CABZ01016138.1|:21830-22975
NuwaI-2_DRer	emb|CABZ01016138.1|:43737-44146
NuwaI-2_DRer	emb|CABZ01074103.1|:13649-14380
NuwaI-2_DRer	emb|CABZ01073813.1|:13151-14289
NuwaI-2_DRer	emb|CABZ01059320.1|:38361-39502
NuwaI-2_DRer	emb|CABZ01014640.1|:18879-20025
NuwaI-2_DRer	emb|CABZ01067029.1|:496-595
NuwaI-2_DRer	emb|CABZ01067029.1|:11820-12961
NuwaI-2_DRer	emb|CABZ01065819.1|:14353-15482
NuwaI-2_DRer	emb|CABZ01061263.1|:3273-4413
NuwaI-2_DRer	emb|CABZ01060450.1|:6413-7654
NuwaI-2_DRer	emb|CABZ01060450.1|:107610-108752
NuwaI-2_DRer	emb|CABZ01059503.1|:30691-31834
NuwaI-2_DRer	emb|CABZ01041373.1|:9801-9902
NuwaI-2_DRer	emb|CABZ01041373.1|:31779-33916
NuwaI-2_DRer	emb|CABZ01038554.1|:22405-23547
NuwaI-2_DRer	emb|CABZ01028507.1|:26336-27479
NuwaI-2_DRer	emb|CABZ01000693.1|:5988-7138
NuwaI-2_DRer	emb|CABZ01000693.1|:20751-22563
NuwaI-2_DRer	emb|CABZ01000377.1|:7704-8844
NuwaI-2_DRer	emb|CABZ01086471.1|:11136-12280
NuwaI-2_DRer	emb|CABZ01076385.1|:14439-15573
NuwaI-2_DRer	emb|CABZ01070838.1|:21965-23111
NuwaI-2_DRer	emb|CABZ01065667.1|:28579-29720
NuwaI-2_DRer	emb|CABZ01065667.1|:3165-4382
NuwaI-2_DRer	emb|CABZ01065141.1|:29254-30398
NuwaI-2_DRer	emb|CABZ01065141.1|:6924-7701
NuwaI-2_DRer	emb|CABZ01064959.1|:1812-2953
NuwaI-2_DRer	emb|CABZ01041517.1|:45245-46385
NuwaI-2_DRer	emb|CABZ01013663.1|:32888-34028
NuwaI-2_DRer	emb|CABZ01004505.1|:13099-13953
NuwaI-2_DRer	emb|CABZ01111554.1|:19293-20396
NuwaI-2_DRer	emb|CABZ01039241.1|:6222-7368
NuwaI-2_DRer	emb|CABZ01020622.1|:175-544

NuwaI-2_DRer	emb|CABZ01001713.1|:18194-19337
NuwaI-2_DRer	emb|CABZ01077510.1|:11779-12925
NuwaI-2_DRer	emb|CABZ01071420.1|:22687-23827
NuwaI-2_DRer	emb|CABZ01043221.1|:3071-4221
NuwaI-2_DRer	emb|CABZ01042970.1|:65362-65839
NuwaI-2_DRer	emb|CABZ01016764.1|:509-1657
NuwaI-2_DRer	emb|CABZ01002925.1|:7026-8163
NuwaI-2_DRer	emb|CABZ01111433.1|:13230-14375
NuwaI-2_DRer	emb|CABZ01075643.1|:9212-10355
NuwaI-2_DRer	emb|CABZ01073754.1|:13179-14328
NuwaI-2_DRer	emb|CABZ01048643.1|:5647-6489
NuwaI-2_DRer	emb|CABZ01042505.1|:23126-24207
NuwaI-2_DRer	emb|CABZ01042505.1|:66409-67545
NuwaI-2_DRer	emb|CABZ01032732.1|:4014-5136
NuwaI-2_DRer	emb|CABZ01017482.1|:26428-27573
NuwaI-2_DRer	emb|CABZ01016392.1|:34682-35848
NuwaI-2_DRer	emb|CABZ01016392.1|:22762-23881
NuwaI-2_DRer	emb|CABZ01014762.1|:30560-31708
NuwaI-2_DRer	emb|CABZ01011887.1|:12267-13415
NuwaI-2_DRer	emb|CABZ01009867.1|:7376-8525
NuwaI-2_DRer	emb|CABZ01009867.1|:46208-47349
NuwaI-2_DRer	emb|CABZ01005194.1|:14190-15295
NuwaI-2_DRer	emb|CABZ01118911.1|:6285-7427
NuwaI-2_DRer	emb|CABZ01080067.1|:18156-19304
NuwaI-2_DRer	emb|CABZ01079647.1|:4449-5590
NuwaI-2_DRer	emb|CABZ01077117.1|:6904-7610
NuwaI-2_DRer	emb|CABZ01062111.1|:42207-43342
NuwaI-2_DRer	emb|CABZ01062111.1|:30126-31274
NuwaI-2_DRer	emb|CABZ01061696.1|:7983-9134
NuwaI-2_DRer	emb|CABZ01055048.1|:12446-13583
NuwaI-2_DRer	emb|CABZ01054459.1|:2421-3565
NuwaI-2_DRer	emb|CABZ01053743.1|:10518-11160
NuwaI-2_DRer	emb|CABZ01053743.1|:1618-2757
NuwaI-2_DRer	emb|CABZ01046276.1|:18535-19678
NuwaI-2_DRer	emb|CABZ01045653.1|:4206-5345
NuwaI-2_DRer	emb|CABZ01040773.1|:56-2139
NuwaI-2_DRer	emb|CABZ01040773.1|:903-2048
NuwaI-2_DRer	emb|CABZ01020559.1|:33949-35090
NuwaI-2_DRer	emb|CABZ01018831.1|:10530-11673
NuwaI-2_DRer	emb|CABZ01017381.1|:14439-16040
NuwaI-2_DRer	emb|CABZ01017381.1|:15103-15273
NuwaI-2_DRer	emb|CABZ01011036.1|:11318-12466
NuwaI-2_DRer	emb|CABZ01003063.1|:10215-11319
NuwaI-2_DRer	emb|CABZ01079241.1|:42778-45091

NuwaI-2_DRer	emb|CABZ01075819.1|:5411-6561
NuwaI-2_DRer	emb|CABZ01071421.1|:50258-51402
NuwaI-2_DRer	emb|CABZ01071421.1|:63065-64205
NuwaI-2_DRer	emb|CABZ01046771.1|:7949-9112
NuwaI-2_DRer	emb|CABZ01045040.1|:1712-2167
NuwaI-2_DRer	emb|CABZ01038224.1|:71760-72916
NuwaI-2_DRer	emb|CABZ01038224.1|:12661-13686
NuwaI-2_DRer	emb|CABZ01027320.1|:37630-38772
NuwaI-2_DRer	emb|CABZ01019908.1|:17198-18334
NuwaI-2_DRer	emb|CABZ01019908.1|:5068-6215
NuwaI-2_DRer	emb|CABZ01003371.1|:14036-15174
NuwaI-2_DRer	emb|CABZ01102031.1|:3741-4444
NuwaI-2_DRer	emb|CABZ01090223.1|:6216-7077
NuwaI-2_DRer	emb|CABZ01041299.1|:21874-24140
NuwaI-2_DRer	emb|CABZ01041299.1|:24728-25866
NuwaI-2_DRer	emb|CABZ01021396.1|:2699-3834
NuwaI-2_DRer	emb|CABZ01088299.1|:6452-7593
NuwaI-2_DRer	emb|CABZ01086483.1|:29741-30893
NuwaI-2_DRer	emb|CABZ01074179.1|:8914-9165
NuwaI-2_DRer	emb|CABZ01074179.1|:20523-21668
NuwaI-2_DRer	emb|CABZ01065675.1|:15601-16746
NuwaI-2_DRer	emb|CABZ01058465.1|:85149-86241
NuwaI-2_DRer	emb|CABZ01058465.1|:74352-75461
NuwaI-2_DRer	emb|CABZ01056101.1|:4601-5741
NuwaI-2_DRer	emb|CABZ01055553.1|:7391-8532
NuwaI-2_DRer	emb|CABZ01053544.1|:52083-52159
NuwaI-2_DRer	emb|CABZ01053544.1|:50178-51346
NuwaI-2_DRer	emb|CABZ01042943.1|:6203-7338
NuwaI-2_DRer	emb|CABZ01042943.1|:85166-85741
NuwaI-2_DRer	emb|CABZ01042943.1|:76578-79414
NuwaI-2_DRer	emb|CABZ01039165.1|:42931-44054
NuwaI-2_DRer	emb|CABZ01037620.1|:34458-35535
NuwaI-2_DRer	emb|CABZ01032995.1|:7378-8530
NuwaI-2_DRer	emb|CABZ01030272.1|:628-1771
NuwaI-2_DRer	emb|CABZ01016245.1|:8452-9601
NuwaI-2_DRer	emb|CABZ01088692.1|:39566-40510
NuwaI-2_DRer	emb|CABZ01088692.1|:7644-8313
NuwaI-2_DRer	emb|CABZ01087281.1|:35160-36304
NuwaI-2_DRer	emb|CABZ01075625.1|:7102-8240
NuwaI-2_DRer	emb|CABZ01068451.1|:1369-2516
NuwaI-2_DRer	emb|CABZ01066022.1|:27028-28172
NuwaI-2_DRer	emb|CABZ01042884.1|:63506-64653
NuwaI-2_DRer	emb|CABZ01042884.1|:42161-42348
NuwaI-2_DRer	emb|CABZ01032165.1|:5795-6938

NuwaI-2_DRer	emb|CABZ01031923.1|:25528-26664
NuwaI-2_DRer	emb|CABZ01025743.1|:4028-5135
NuwaI-2_DRer	emb|CABZ01020689.1|:1-1008
NuwaI-2_DRer	emb|CABZ01009958.1|:20170-21308
NuwaI-2_DRer	emb|CABZ01007951.1|:12803-13956
NuwaI-2_DRer	emb|CABZ01007271.1|:7451-7821
NuwaI-2_DRer	emb|CABZ01083797.1|:2555-3009
NuwaI-2_DRer	emb|CABZ01073709.1|:14300-15447
NuwaI-2_DRer	emb|CABZ01054962.1|:4804-5867
NuwaI-2_DRer	emb|CABZ01039638.1|:12878-14058
NuwaI-2_DRer	emb|CABZ01034904.1|:3591-4736
NuwaI-2_DRer	emb|CABZ01032790.1|:14986-16131
NuwaI-2_DRer	emb|CABZ01031377.1|:3913-5066
NuwaI-2_DRer	emb|CABZ01031377.1|:5107-5304
NuwaI-2_DRer	emb|CABZ01031117.1|:26912-27959
NuwaI-2_DRer	emb|CABZ01031117.1|:16960-17692
NuwaI-2_DRer	emb|CABZ01029682.1|:20823-21962
NuwaI-2_DRer	emb|CABZ01029540.1|:21634-22087
NuwaI-2_DRer	emb|CABZ01008695.1|:9848-10971
NuwaI-2_DRer	emb|CABZ01001738.1|:30157-30933
NuwaI-2_DRer	emb|CABZ01080532.1|:5163-6262
NuwaI-2_DRer	emb|CABZ01077120.1|:21128-22279
NuwaI-2_DRer	emb|CABZ01070372.1|:6418-7562
NuwaI-2_DRer	emb|CABZ01060887.1|:6682-7541
NuwaI-2_DRer	emb|CABZ01053824.1|:18713-19863
NuwaI-2_DRer	emb|CABZ01052277.1|:16039-17183
NuwaI-2_DRer	emb|CABZ01043985.1|:2505-3647
NuwaI-2_DRer	emb|CABZ01041076.1|:32303-33182
NuwaI-2_DRer	emb|CABZ01026615.1|:20184-21324
NuwaI-2_DRer	emb|CABZ01025695.1|:42918-44012
NuwaI-2_DRer	emb|CABZ01023073.1|:37758-39103
NuwaI-2_DRer	emb|CABZ01023073.1|:51279-52358
NuwaI-3_DRer	emb|CABZ01038382.1|:65794-69128
NuwaI-3_DRer	emb|CABZ01034087.1|:11690-14250
NuwaI-3_DRer	emb|CABZ01018303.1|:38692-39496
NuwaI-3_DRer	emb|CABZ01019257.1|:11734-14682
NuwaI-3_DRer	emb|CABZ01040807.1|:124-1760
NuwaI-3_DRer	emb|CABZ01040807.1|:2679-2749
NuwaI-3_DRer	emb|CABZ01042392.1|:14103-14892
NuwaI-3_DRer	emb|CABZ01010594.1|:8211-14007
NuwaI-3_DRer	emb|CABZ01018305.1|:329-1646
NuwaI-3_DRer	emb|CABZ01077003.1|:42-3049
NuwaI-3_DRer	emb|CABZ01034088.1|:405-1715
NuwaI-3_DRer	emb|CABZ01034088.1|:5159-6705

NuwaI-3_DRer	emb|CABZ01040808.1|:141-1455
NuwaI-3_DRer	emb|CABZ01060257.1|:1-1494
NuwaI-3_DRer	emb|CABZ01018304.1|:127-1749
NuwaI-3_DRer	emb|CABZ01085278.1|:1-760
NuwaI-3_DRer	emb|CABZ01062295.1|:7599-9242
NuwaI-3_DRer	emb|CABZ01100426.1|:256-542
NuwaI-3_DRer	emb|CABZ01059097.1|:2054-3712
NuwaI-3_DRer	emb|CABZ01026162.1|:3381-5050
NuwaI-3_DRer	emb|CABZ01011890.1|:2716-3002
NuwaI-3_DRer	emb|CABZ01009397.1|:7420-9086
NuwaI-3_DRer	emb|CABZ01024150.1|:460-2115
NuwaI-3_DRer	emb|CABZ01061929.1|:3478-3753
NuwaI-3_DRer	emb|CABZ01059129.1|:13455-15084
NuwaI-3_DRer	emb|CABZ01059129.1|:9636-11725
NuwaI-3_DRer	emb|CABZ01005480.1|:47421-49081
NuwaI-3_DRer	emb|CABZ01005480.1|:41487-43122
NuwaI-3_DRer	emb|CABZ01002288.1|:16858-18523
NuwaI-3_DRer	emb|CABZ01103276.1|:51-1704
NuwaI-3_DRer	emb|CABZ01093042.1|:5914-7587
NuwaI-3_DRer	emb|CABZ01071070.1|:13758-15392
NuwaI-3_DRer	emb|CABZ01065538.1|:24607-26264
NuwaI-3_DRer	emb|CABZ01063618.1|:47421-49078
NuwaI-3_DRer	emb|CABZ01063618.1|:42667-44847
NuwaI-3_DRer	emb|CABZ01052578.1|:5794-7456
NuwaI-3_DRer	emb|CABZ01050603.1|:5668-7324
NuwaI-3_DRer	emb|CABZ01046775.1|:11738-13399
NuwaI-3_DRer	emb|CABZ01045229.1|:6188-7824
NuwaI-3_DRer	emb|CABZ01043937.1|:18435-20092
NuwaI-3_DRer	emb|CABZ01038046.1|:10531-12157
NuwaI-3_DRer	emb|CABZ01036898.1|:6189-7855
NuwaI-3_DRer	emb|CABZ01036808.1|:2458-2744
NuwaI-3_DRer	emb|CABZ01034623.1|:18021-19691
NuwaI-3_DRer	emb|CABZ01007052.1|:43458-45125
NuwaI-3_DRer	emb|CABZ01080236.1|:7215-8831
NuwaI-3_DRer	emb|CABZ01060634.1|:59965-60028
NuwaI-3_DRer	emb|CABZ01060634.1|:79409-81058
NuwaI-3_DRer	emb|CABZ01060634.1|:60199-61585
NuwaI-3_DRer	emb|CABZ01054518.1|:60267-63503
NuwaI-3_DRer	emb|CABZ01046323.1|:31656-33294
NuwaI-3_DRer	emb|CABZ01024148.1|:12563-14219
NuwaI-3_DRer	emb|CABZ01001063.1|:15549-17216
NuwaI-3_DRer	emb|CABZ01001063.1|:5026-6695
NuwaI-3_DRer	emb|CABZ01042458.1|:6-1637
NuwaI-3_DRer	emb|CABZ01061838.1|:995-2618

NuwaI-3_DRer	emb|CABZ01040215.1|:682-2349
NuwaI-3_DRer	emb|CABZ01040215.1|:4085-5660
NuwaI-3_DRer	emb|CABZ01034104.1|:29790-31448
NuwaI-3_DRer	emb|CABZ01009831.1|:10336-11925
NuwaI-3_DRer	emb|CABZ01007829.1|:1249-1524
NuwaI-3_DRer	emb|CABZ01083783.1|:994-2656
NuwaI-3_DRer	emb|CABZ01077516.1|:2229-3877
NuwaI-3_DRer	emb|CABZ01073812.1|:18829-20473
NuwaI-3_DRer	emb|CABZ01073663.1|:39471-41125
NuwaI-3_DRer	emb|CABZ01069982.1|:3340-5006
NuwaI-3_DRer	emb|CABZ01067306.1|:22503-24164
NuwaI-3_DRer	emb|CABZ01065920.1|:8454-10118
NuwaI-3_DRer	emb|CABZ01055425.1|:1466-3100
NuwaI-3_DRer	emb|CABZ01047395.1|:44183-45838
NuwaI-3_DRer	emb|CABZ01047395.1|:29955-30744
NuwaI-3_DRer	emb|CABZ01046422.1|:23411-24990
NuwaI-3_DRer	emb|CABZ01036836.1|:64572-66237
NuwaI-3_DRer	emb|CABZ01031948.1|:2290-3933
NuwaI-3_DRer	emb|CABZ01031611.1|:514-2185
NuwaI-3_DRer	emb|CABZ01030903.1|:9516-11171
NuwaI-3_DRer	emb|CABZ01024887.1|:19752-21410
NuwaI-3_DRer	emb|CABZ01022132.1|:12266-13930
NuwaI-3_DRer	emb|CABZ01021612.1|:12460-14120
NuwaI-3_DRer	emb|CABZ01015321.1|:10956-12596
NuwaI-3_DRer	emb|CABZ01015208.1|:15754-17419
NuwaI-3_DRer	emb|CABZ01014003.1|:20450-22118
NuwaI-3_DRer	emb|CABZ01002647.1|:470-2117
NuwaI-3_DRer	emb|CABZ01112280.1|:1-1627
NuwaI-3_DRer	emb|CABZ01072313.1|:4-1647
NuwaI-3_DRer	emb|CABZ01056303.1|:5325-6833
NuwaI-3_DRer	emb|CABZ01056303.1|:3405-5026
NuwaI-3_DRer	emb|CABZ01054402.1|:32719-34024
NuwaI-3_DRer	emb|CABZ01008639.1|:4121-5776
NuwaI-3_DRer	emb|CABZ01006481.1|:16628-19313
NuwaI-3_DRer	emb|CABZ01061930.1|:285-1953
NuwaI-3_DRer	emb|CABZ01059690.1|:12574-14479
NuwaI-3_DRer	emb|CABZ01006480.1|:47907-49510
NuwaI-3_DRer	emb|CABZ01092893.1|:21724-23387
NuwaI-3_DRer	emb|CABZ01068366.1|:20750-22377
NuwaI-3_DRer	emb|CABZ01068362.1|:31580-33248
NuwaI-3_DRer	emb|CABZ01067426.1|:3642-5299
NuwaI-3_DRer	emb|CABZ01063069.1|:7291-8957
NuwaI-3_DRer	emb|CABZ01054218.1|:1112-2746
NuwaI-3_DRer	emb|CABZ01051460.1|:44149-45742

NuwaI-3_DRer	emb|CABZ01041789.1|:53063-54720
NuwaI-3_DRer	emb|CABZ01041405.1|:8932-10560
NuwaI-3_DRer	emb|CABZ01039056.1|:22723-26780
NuwaI-3_DRer	emb|CABZ01027676.1|:13915-15546
NuwaI-3_DRer	emb|CABZ01023896.1|:106-1316
NuwaI-3_DRer	emb|CABZ01017041.1|:8007-9657
NuwaI-3_DRer	emb|CABZ01015436.1|:4439-6040
NuwaI-3_DRer	emb|CABZ01013709.1|:11667-13305
NuwaI-3_DRer	emb|CABZ01003054.1|:15448-17117
NuwaI-3_DRer	emb|CABZ01001296.1|:23511-25172
NuwaI-3_DRer	emb|CABZ01105229.1|:137-1753
NuwaI-3_DRer	emb|CABZ01102181.1|:6673-8316
NuwaI-3_DRer	emb|CABZ01089294.1|:736-1022
NuwaI-3_DRer	emb|CABZ01087072.1|:13980-15633
NuwaI-3_DRer	emb|CABZ01083571.1|:14436-16079
NuwaI-3_DRer	emb|CABZ01074271.1|:22028-23695
NuwaI-3_DRer	emb|CABZ01072314.1|:177-463
NuwaI-3_DRer	emb|CABZ01065976.1|:39735-41381
NuwaI-3_DRer	emb|CABZ01061959.1|:2624-4260
NuwaI-3_DRer	emb|CABZ01059792.1|:2086-2372
NuwaI-3_DRer	emb|CABZ01058366.1|:10321-11956
NuwaI-3_DRer	emb|CABZ01058108.1|:3672-3958
NuwaI-3_DRer	emb|CABZ01058027.1|:27961-29611
NuwaI-3_DRer	emb|CABZ01048564.1|:5636-7085
NuwaI-3_DRer	emb|CABZ01046996.1|:30375-31995
NuwaI-3_DRer	emb|CABZ01046996.1|:21867-23319
NuwaI-3_DRer	emb|CABZ01042104.1|:7288-8423
NuwaI-3_DRer	emb|CABZ01042104.1|:44500-46158
NuwaI-3_DRer	emb|CABZ01042104.1|:38958-40340
NuwaI-3_DRer	emb|CABZ01036319.1|:13944-15591
NuwaI-3_DRer	emb|CABZ01034196.1|:18532-20148
NuwaI-3_DRer	emb|CABZ01033944.1|:42504-44169
NuwaI-3_DRer	emb|CABZ01033944.1|:56010-57949
NuwaI-3_DRer	emb|CABZ01033944.1|:30230-34414
NuwaI-3_DRer	emb|CABZ01031897.1|:6708-8379
NuwaI-3_DRer	emb|CABZ01030424.1|:7225-8876
NuwaI-3_DRer	emb|CABZ01030332.1|:7229-8856
NuwaI-3_DRer	emb|CABZ01027255.1|:26384-28034
NuwaI-3_DRer	emb|CABZ01027255.1|:28870-30520
NuwaI-3_DRer	emb|CABZ01024040.1|:2245-3889
NuwaI-3_DRer	emb|CABZ01021656.1|:24071-26258
NuwaI-3_DRer	emb|CABZ01018034.1|:27500-29147
NuwaI-3_DRer	emb|CABZ01017912.1|:6081-7752
NuwaI-3_DRer	emb|CABZ01009105.1|:12547-14193

NuwaI-3_DRer	emb|CABZ01009105.1|:10594-12234
NuwaI-3_DRer	emb|CABZ01005651.1|:4541-6206
NuwaI-3_DRer	emb|CABZ01004881.1|:18786-20031
NuwaI-3_DRer	emb|CABZ01001491.1|:2660-4313
NuwaI-3_DRer	emb|CABZ01000712.1|:6031-7668
NuwaI-3_DRer	emb|CABZ01117878.1|:3359-4986
NuwaI-3_DRer	emb|CABZ01087220.1|:29785-31440
NuwaI-3_DRer	emb|CABZ01087220.1|:21114-22678
NuwaI-3_DRer	emb|CABZ01087053.1|:8662-10325
NuwaI-3_DRer	emb|CABZ01071528.1|:756-2413
NuwaI-3_DRer	emb|CABZ01060452.1|:38079-40549
NuwaI-3_DRer	emb|CABZ01050686.1|:5147-6766
NuwaI-3_DRer	emb|CABZ01040136.1|:25890-27447
NuwaI-3_DRer	emb|CABZ01038678.1|:698-2365
NuwaI-3_DRer	emb|CABZ01037432.1|:2318-2587
NuwaI-3_DRer	emb|CABZ01036013.1|:22423-23956
NuwaI-3_DRer	emb|CABZ01035688.1|:24370-26038
NuwaI-3_DRer	emb|CABZ01026358.1|:23288-24942
NuwaI-3_DRer	emb|CABZ01026063.1|:559-2188
NuwaI-3_DRer	emb|CABZ01025336.1|:26165-27822
NuwaI-3_DRer	emb|CABZ01024159.1|:3422-5037
NuwaI-3_DRer	emb|CABZ01021250.1|:16194-17815
NuwaI-3_DRer	emb|CABZ01003168.1|:7483-10238
NuwaI-3_DRer	emb|CABZ01002706.1|:14524-16185
NuwaI-3_DRer	emb|CABZ01063049.1|:19293-20928
NuwaI-3_DRer	emb|CABZ01054299.1|:755-2389
NuwaI-3_DRer	emb|CABZ01000517.1|:33944-36342
NuwaI-3_DRer	emb|CABZ01097498.1|:32-1365
NuwaI-3_DRer	emb|CABZ01088724.1|:1169-2781
NuwaI-3_DRer	emb|CABZ01070141.1|:12321-12596
NuwaI-3_DRer	emb|CABZ01070141.1|:10861-12321
NuwaI-3_DRer	emb|CABZ01060020.1|:357-1998
NuwaI-3_DRer	emb|CABZ01059755.1|:5317-6977
NuwaI-3_DRer	emb|CABZ01059231.1|:13690-15330
NuwaI-3_DRer	emb|CABZ01054360.1|:5951-7224
NuwaI-3_DRer	emb|CABZ01054360.1|:3827-5302
NuwaI-3_DRer	emb|CABZ01034851.1|:7414-9074
NuwaI-3_DRer	emb|CABZ01033548.1|:25700-27365
NuwaI-3_DRer	emb|CABZ01030430.1|:4083-5740
NuwaI-3_DRer	emb|CABZ01029503.1|:8571-10216
NuwaI-3_DRer	emb|CABZ01023427.1|:67626-69247
NuwaI-3_DRer	emb|CABZ01023340.1|:19652-21298
NuwaI-3_DRer	emb|CABZ01019809.1|:13553-15219
NuwaI-3_DRer	emb|CABZ01017465.1|:21167-22831

NuwaI-3_DRer	emb|CABZ01007290.1|:1351-1638
NuwaI-3_DRer	emb|CABZ01006803.1|:36706-38341
NuwaI-3_DRer	emb|CABZ01109030.1|:379-665
NuwaI-3_DRer	emb|CABZ01092891.1|:1367-3007
NuwaI-3_DRer	emb|CABZ01089151.1|:8089-9593
NuwaI-3_DRer	emb|CABZ01079424.1|:8020-9320
NuwaI-3_DRer	emb|CABZ01079423.1|:634-2264
NuwaI-3_DRer	emb|CABZ01078138.1|:4050-5675
NuwaI-3_DRer	emb|CABZ01076532.1|:29630-31289
NuwaI-3_DRer	emb|CABZ01073739.1|:11647-13176
NuwaI-3_DRer	emb|CABZ01072100.1|:5415-7039
NuwaI-3_DRer	emb|CABZ01070636.1|:4259-7274
NuwaI-3_DRer	emb|CABZ01065746.1|:31451-32041
NuwaI-3_DRer	emb|CABZ01065746.1|:43803-45441
NuwaI-3_DRer	emb|CABZ01065254.1|:12474-14080
NuwaI-3_DRer	emb|CABZ01065254.1|:45171-45456
NuwaI-3_DRer	emb|CABZ01065134.1|:12119-13783
NuwaI-3_DRer	emb|CABZ01063772.1|:6709-8374
NuwaI-3_DRer	emb|CABZ01049314.1|:4674-6283
NuwaI-3_DRer	emb|CABZ01044354.1|:177-1816
NuwaI-3_DRer	emb|CABZ01039751.1|:12008-13662
NuwaI-3_DRer	emb|CABZ01021843.1|:23450-25107
NuwaI-3_DRer	emb|CABZ01020351.1|:77040-78636
NuwaI-3_DRer	emb|CABZ01018382.1|:14127-15784
NuwaI-3_DRer	emb|CABZ01016032.1|:7219-8885
NuwaI-3_DRer	emb|CABZ01015245.1|:19872-21480
NuwaI-3_DRer	emb|CABZ01013688.1|:6917-8559
NuwaI-3_DRer	emb|CABZ01011526.1|:12063-13685
NuwaI-3_DRer	emb|CABZ01008364.1|:62024-63686
NuwaI-3_DRer	emb|CABZ01007398.1|:85862-87388
NuwaI-3_DRer	emb|CABZ01006523.1|:5250-6795
NuwaI-3_DRer	emb|CABZ01003085.1|:4244-5904
NuwaI-3_DRer	emb|CABZ01000753.1|:3809-5440
NuwaI-3_DRer	emb|CABZ01069077.1|:9990-11618
NuwaI-3_DRer	emb|CABZ01063521.1|:5545-7167
NuwaI-3_DRer	emb|CABZ01051935.1|:459-1591
NuwaI-3_DRer	emb|CABZ01051559.1|:168-1793
NuwaI-3_DRer	emb|CABZ01040451.1|:8931-11583
NuwaI-3_DRer	emb|CABZ01038677.1|:7015-8674
NuwaI-3_DRer	emb|CABZ01016319.1|:2207-3833
NuwaI-3_DRer	emb|CABZ01016319.1|:16001-17581
NuwaI-3_DRer	emb|CABZ01012596.1|:990-2630
NuwaI-3_DRer	emb|CABZ01006928.1|:16233-17890
NuwaI-3_DRer	emb|CABZ01118891.1|:1168-2802

NuwaI-3_DRer	emb|CABZ01078092.1|:709-2230
NuwaI-3_DRer	emb|CABZ01077510.1|:2-762
NuwaI-3_DRer	emb|CABZ01071087.1|:9078-10693
NuwaI-3_DRer	emb|CABZ01023016.1|:3919-5582
NuwaI-3_DRer	emb|CABZ01112206.1|:20122-21775
NuwaI-3_DRer	emb|CABZ01101869.1|:2342-3995
NuwaI-3_DRer	emb|CABZ01086692.1|:351-1711
NuwaI-3_DRer	emb|CABZ01075208.1|:16275-17926
NuwaI-3_DRer	emb|CABZ01070534.1|:2309-3975
NuwaI-3_DRer	emb|CABZ01068023.1|:17258-18799
NuwaI-3_DRer	emb|CABZ01068023.1|:18807-20426
NuwaI-3_DRer	emb|CABZ01065949.1|:24914-26516
NuwaI-3_DRer	emb|CABZ01059912.1|:37779-39435
NuwaI-3_DRer	emb|CABZ01058107.1|:72431-74098
NuwaI-3_DRer	emb|CABZ01051638.1|:4127-8764
NuwaI-3_DRer	emb|CABZ01049377.1|:554-2199
NuwaI-3_DRer	emb|CABZ01044570.1|:9010-10575
NuwaI-3_DRer	emb|CABZ01033956.1|:3429-5091
NuwaI-3_DRer	emb|CABZ01032346.1|:1796-3399
NuwaI-3_DRer	emb|CABZ01032335.1|:27740-29381
NuwaI-3_DRer	emb|CABZ01010145.1|:404-2039
NuwaI-3_DRer	emb|CABZ01009389.1|:15143-16789
NuwaI-3_DRer	emb|CABZ01003682.1|:10412-12046
NuwaI-3_DRer	emb|CABZ01117756.1|:2746-4331
NuwaI-3_DRer	emb|CABZ01109014.1|:1136-1422
NuwaI-3_DRer	emb|CABZ01093379.1|:1890-3560
NuwaI-3_DRer	emb|CABZ01085046.1|:8978-10587
NuwaI-3_DRer	emb|CABZ01079295.1|:2122-6212
NuwaI-3_DRer	emb|CABZ01075308.1|:15945-17607
NuwaI-3_DRer	emb|CABZ01074476.1|:1638-2955
NuwaI-3_DRer	emb|CABZ01074240.1|:629-2098
NuwaI-3_DRer	emb|CABZ01074240.1|:2103-3755
NuwaI-3_DRer	emb|CABZ01071156.1|:58957-60613
NuwaI-3_DRer	emb|CABZ01066917.1|:17106-17380
NuwaI-3_DRer	emb|CABZ01066917.1|:13095-14728
NuwaI-3_DRer	emb|CABZ01066519.1|:17613-19228
NuwaI-3_DRer	emb|CABZ01066519.1|:35534-37110
NuwaI-3_DRer	emb|CABZ01060972.1|:2428-4093
NuwaI-3_DRer	emb|CABZ01060594.1|:11703-13358
NuwaI-3_DRer	emb|CABZ01056532.1|:360-2020
NuwaI-3_DRer	emb|CABZ01053281.1|:8519-10116
NuwaI-3_DRer	emb|CABZ01052982.1|:937-2570
NuwaI-3_DRer	emb|CABZ01049025.1|:2511-4170
NuwaI-3_DRer	emb|CABZ01046769.1|:25195-26828

NuwaI-3_DRer	emb|CABZ01045977.1|:4349-6016
NuwaI-3_DRer	emb|CABZ01045125.1|:5296-6955
NuwaI-4_DRer	emb|CABZ01037752.1|:7238-11194
NuwaI-4_DRer	emb|CABZ01040630.1|:4542-8497
NuwaI-4_DRer	emb|CABZ01007274.1|:52531-56487
NuwaI-4_DRer	emb|CABZ01019249.1|:91-2059
NuwaI-4_DRer	emb|CABZ01008464.1|:119-2086
NuwaI-4_DRer	emb|CABZ01019248.1|:8716-10451
NuwaI-4_DRer	emb|CABZ01028605.1|:20636-22080
NuwaI-4_DRer	emb|CABZ01069800.1|:11414-12866
NuwaI-4_DRer	emb|CABZ01040631.1|:7-1084
NuwaI-4_DRer	emb|CABZ01017746.1|:14855-16002
NuwaI-4_DRer	emb|CABZ01039033.1|:14462-15614
NuwaI-4_DRer	emb|CABZ01032008.1|:6301-7455
NuwaI-4_DRer	emb|CABZ01058465.1|:43572-44674
NuwaI-4_DRer	emb|CABZ01009681.1|:22804-23820
NuwaI-4_DRer	emb|CABZ01005962.1|:20538-21553
NuwaI-4_DRer	emb|CABZ01060687.1|:15146-16161
NuwaI-4_DRer	emb|CABZ01080922.1|:1-1011
NuwaI-4_DRer	emb|CABZ01008335.1|:20399-21416
NuwaI-4_DRer	emb|CABZ01023308.1|:12867-13882
NuwaI-4_DRer	emb|CABZ01014023.1|:23859-24874
NuwaI-4_DRer	emb|CABZ01005007.1|:9710-10725
NuwaI-4_DRer	emb|CABZ01067809.1|:24667-25550
NuwaI-4_DRer	emb|CABZ01010058.1|:15598-16479
NuwaI-4_DRer	emb|CABZ01083833.1|:7702-8584
NuwaI-4_DRer	emb|CABZ01015173.1|:8107-8986
NuwaI-4_DRer	emb|CABZ01032825.1|:14297-15177
NuwaI-4_DRer	emb|CABZ01033148.1|:1092-1984
NuwaI-4_DRer	emb|CABZ01023377.1|:13805-14697
NuwaI-4_DRer	emb|CABZ01022196.1|:9820-10724
NuwaI-4_DRer	emb|CABZ01012209.1|:64919-65807
NuwaI-4_DRer	emb|CABZ01011580.1|:4350-5238
NuwaI-4_DRer	emb|CABZ01080594.1|:6991-7883
NuwaI-4_DRer	emb|CABZ01030239.1|:17573-18460
NuwaI-4_DRer	emb|CABZ01013753.1|:11980-12872
NuwaI-4_DRer	emb|CABZ01042460.1|:1350-2241
NuwaI-4_DRer	emb|CABZ01037780.1|:12237-13128
NuwaI-4_DRer	emb|CABZ01018308.1|:80162-81053
NuwaI-4_DRer	emb|CABZ01005606.1|:27688-28579
NuwaI-4_DRer	emb|CABZ01049762.1|:3867-4772
NuwaI-4_DRer	emb|CABZ01043341.1|:35478-36362
NuwaI-4_DRer	emb|CABZ01038249.1|:1653-2530
NuwaI-4_DRer	emb|CABZ01016748.1|:145785-146498

NuwaI-4_DRer	emb|CABZ01051167.1|:15334-15935
NuwaI-4_DRer	emb|CABZ01051270.1|:6057-6523
NuwaI-4_DRer	emb|CABZ01008463.1|:4848-5199
NuwaI-4_DRer	emb|CABZ01071374.1|:54554-54739
NuwaI-4_DRer	emb|CABZ01071428.1|:3568-3715
NuwaI-4_DRer	emb|CABZ01042853.1|:2340-2452
NuwaI-4_DRer	emb|CABZ01040894.1|:13303-13409
NuwaI-4_DRer	emb|CABZ01042205.1|:599-694
NuwaI-4_DRer	emb|CABZ01074045.1|:1227-1316
NuwaI-4_DRer	emb|CABZ01088915.1|:1991-2066
NuwaI-4_DRer	emb|CABZ01027116.1|:11739-11799
NuwaI-4_DRer	emb|CABZ01019072.1|:2252-2309
NuwaI-4_DRer	emb|CABZ01001120.1|:3272-5285
NuwaI-4_DRer	emb|CABZ01058686.1|:2595-4164
NuwaI-4_DRer	emb|CABZ01059238.1|:1-1124
NuwaI-4_DRer	emb|CABZ01059237.1|:18345-19406
NuwaI-4_DRer	emb|CABZ01016598.1|:12251-13355
NuwaI-4_DRer	emb|CABZ01068957.1|:9396-10503
NuwaI-4_DRer	emb|CABZ01076100.1|:25551-26701
NuwaI-4_DRer	emb|CABZ01051271.1|:43-1171
NuwaI-4_DRer	emb|CABZ01039803.1|:15281-16296
NuwaI-4_DRer	emb|CABZ01034158.1|:7681-8697
NuwaI-4_DRer	emb|CABZ01026639.1|:34854-35869
NuwaI-4_DRer	emb|CABZ01012854.1|:2914-3935
NuwaI-4_DRer	emb|CABZ01004730.1|:8973-9992
NuwaI-4_DRer	emb|CABZ01016748.1|:144759-145777
NuwaI-4_DRer	emb|CABZ01074657.1|:1981-2867
NuwaI-4_DRer	emb|CABZ01009976.1|:7453-8333
NuwaI-4_DRer	emb|CABZ01046528.1|:4580-5472
NuwaI-4_DRer	emb|CABZ01035703.1|:3992-4880
NuwaI-4_DRer	emb|CABZ01027164.1|:17209-18101
NuwaI-4_DRer	emb|CABZ01074847.1|:8954-9846
NuwaI-4_DRer	emb|CABZ01060524.1|:5879-6770
NuwaI-4_DRer	emb|CABZ01048119.1|:32436-33327
NuwaI-4_DRer	emb|CABZ01047983.1|:84485-85376
NuwaI-4_DRer	emb|CABZ01031881.1|:47057-47948
NuwaI-4_DRer	emb|CABZ01112342.1|:1477-2368
NuwaI-4_DRer	emb|CABZ01085225.1|:1432-2327
NuwaI-4_DRer	emb|CABZ01059981.1|:18845-19736
NuwaI-4_DRer	emb|CABZ01001127.1|:2068-2960
NuwaI-4_DRer	emb|CABZ01070235.1|:10077-10968
NuwaI-4_DRer	emb|CABZ01046477.1|:5256-6146
NuwaI-4_DRer	emb|CABZ01001312.1|:5331-6223
NuwaI-4_DRer	emb|CABZ01051327.1|:13364-14324

	NuwaI-4_DRer	emb|CABZ01011101.1|:9395-10280
	NuwaI-4_DRer	emb|CABZ01001967.1|:2237-3131
	NuwaI-4_DRer	emb|CABZ01042265.1|:9104-9997
	NuwaI-4_DRer	emb|CABZ01007609.1|:6878-7772
	NuwaI-4_DRer	emb|CABZ01094194.1|:1-942
	NuwaI-4_DRer	emb|CABZ01015942.1|:22201-23077
	NuwaI-4_DRer	emb|CABZ01016118.1|:2945-3839
	NuwaI-4_DRer	emb|CABZ01066577.1|:1227-1870
	NuwaI-4_DRer	emb|CABZ01071485.1|:52-643
	NuwaI-4_DRer	emb|CABZ01036329.1|:28-589
	NuwaI-4_DRer	emb|CABZ01093232.1|:50-613
	NuwaI-4_DRer	emb|CABZ01086670.1|:1-484
	NuwaI-4_DRer	emb|CABZ01027772.1|:1-434
	NuwaI-4_DRer	emb|CABZ01034902.1|:12245-12612
	NuwaI-4_DRer	emb|CABZ01020218.1|:12900-13014
	NuwaI-4_DRer	emb|CABZ01082390.1|:4547-4649
	NuwaI-4_DRer	emb|CABZ01069896.1|:10829-10910
	NuwaI-4_DRer	emb|CABZ01050486.1|:8720-8798
	NuwaI-4_DRer	emb|CABZ01047380.1|:14954-15029
	NuwaI-4_DRer	emb|CABZ01005209.1|:8773-8846
	NuwaI-4_DRer	emb|CABZ01088460.1|:3941-4011
	NuwaI-4_DRer	emb|CABZ01031042.1|:29510-29561
	NuwaI-4_DRer	emb|CABZ01054613.1|:41313-41362
Daphnia pulex	*NuwaI-1_DPul*	gb|ACJG01004500.1|:7122-10688
	NuwaI-1_DPul	gb|ACJG01002434.1|:61928-65279
	NuwaI-1_DPul	gb|ACJG01004813.1|:20851-23691
	NuwaI-1_DPul	gb|ACJG01002509.1|:12526-15362
	NuwaI-1_DPul	gb|ACJG01004831.1|:7244-8793
	NuwaI-1_DPul	gb|ACJG01004835.1|:2476-3524
	NuwaI-1_DPul	gb|ACJG01007315.1|:3431-4033
	NuwaI-1_DPul	gb|ACJG01004266.1|:79560-80202
	NuwaI-1_DPul	gb|ACJG01015844.1|:3835-4400
	NuwaI-1_DPul	gb|ACJG01002210.1|:14756-15424
	NuwaI-1_DPul	gb|ACJG01001316.1|:45388-46056
	NuwaI-1_DPul	gb|ACJG01006453.1|:32950-33618
	NuwaI-1_DPul	gb|ACJG01008223.1|:4149-4817
	NuwaI-1_DPul	gb|ACJG01009246.1|:3797-4463
	NuwaI-1_DPul	gb|ACJG01006593.1|:4244-4905
	NuwaI-1_DPul	gb|ACJG01002204.1|:78447-79108
	NuwaI-1_DPul	gb|ACJG01013312.1|:9-631
	NuwaI-1_DPul	gb|ACJG01008289.1|:4-600
	NuwaI-1_DPul	gb|ACJG01006696.1|:9683-10276
	NuwaI-1_DPul	gb|ACJG01010944.1|:7198-7782
	NuwaI-1_DPul	gb|ACJG01014410.1|:404-980

	NuwaI-1_DPul	gb|ACJG01017998.1|:777-944
	NuwaI-1_DPul	gb|ACJG01018137.1|:243-410
	NuwaI-1_DPul	gb|ACJG01004832.1|:4495-4645
	NuwaI-1_DPul	gb|ACJG01002300.1|:1360-1484
	NuwaI-1_DPul	gb|ACJG01018625.1|:348-472
Gadus morhua	*NuwaI_GMor*	emb|CAEA01165212.1|:4702-6582
	NuwaI_GMor	emb|CAEA01478506.1|:2960-4832
	NuwaI_GMor	emb|CAEA01540840.1|:5205-6903
	NuwaI_GMor	emb|CAEA01397192.1|:1621-3239
	NuwaI_GMor	emb|CAEA01545026.1|:1696-3312
	NuwaI_GMor	emb|CAEA01008387.1|:1-1398
	NuwaI_GMor	emb|CAEA01544387.1|:5486-6808
	NuwaI_GMor	emb|CAEA01404062.1|:1863-3003
	NuwaI_GMor	emb|CAEA01200413.1|:1-1188
	NuwaI_GMor	emb|CAEA01005520.1|:610-1740
	NuwaI_GMor	emb|CAEA01538403.1|:1658-2768
	NuwaI_GMor	emb|CAEA01118015.1|:9-977
	NuwaI_GMor	emb|CAEA01113478.1|:1-908
	NuwaI_GMor	emb|CAEA01533608.1|:2234-3121
	NuwaI_GMor	emb|CAEA01168909.1|:3-909
	NuwaI_GMor	emb|CAEA01141401.1|:1-916
	NuwaI_GMor	emb|CAEA01049311.1|:1-905
	NuwaI_GMor	emb|CAEA01417852.1|:3904-4741
	NuwaI_GMor	emb|CAEA01438511.1|:1-836
	NuwaI_GMor	emb|CAEA01076109.1|:1-890
	NuwaI_GMor	emb|CAEA01126769.1|:38-863
	NuwaI_GMor	emb|CAEA01378753.1|:340-1134
	NuwaI_GMor	emb|CAEA01000515.1|:1-846
	NuwaI_GMor	emb|CAEA01415167.1|:1-905
	NuwaI_GMor	emb|CAEA01132711.1|:222-1040
	NuwaI_GMor	emb|CAEA01337057.1|:4-827
	NuwaI_GMor	emb|CAEA01395548.1|:2015-2785
	NuwaI_GMor	emb|CAEA01294703.1|:24-754
	NuwaI_GMor	emb|CAEA01098515.1|:1138-1847
	NuwaI_GMor	emb|CAEA01137883.1|:76-831
	NuwaI_GMor	emb|CAEA01156388.1|:612-1320
	NuwaI_GMor	emb|CAEA01531766.1|:7228-7942
	NuwaI_GMor	emb|CAEA01550346.1|:12-718
	NuwaI_GMor	emb|CAEA01096798.1|:2-989
	NuwaI_GMor	emb|CAEA01210727.1|:1573-2263
	NuwaI_GMor	emb|CAEA01147556.1|:529-1170
	NuwaI_GMor	emb|CAEA01072672.1|:3-727
	NuwaI_GMor	emb|CAEA01277320.1|:818-1473
	NuwaI_GMor	emb|CAEA01352966.1|:4-630

NuwaI_GMor	emb|CAEA01143718.1|:1-603
NuwaI_GMor	emb|CAEA01123679.1|:1-599
NuwaI_GMor	emb|CAEA01486528.1|:1-671
NuwaI_GMor	emb|CAEA01260928.1|:351-944
NuwaI_GMor	emb|CAEA01441769.1|:1-573
NuwaI_GMor	emb|CAEA01002227.1|:2-615
NuwaI_GMor	emb|CAEA01408491.1|:1-600
NuwaI_GMor	emb|CAEA01475261.1|:5-567
NuwaI_GMor	emb|CAEA01415035.1|:2-583
NuwaI_GMor	emb|CAEA01113897.1|:3-559
NuwaI_GMor	emb|CAEA01114988.1|:23-558
NuwaI_GMor	emb|CAEA01439188.1|:3-728
NuwaI_GMor	emb|CAEA01550401.1|:12-589
NuwaI_GMor	emb|CAEA01313732.1|:307-843
NuwaI_GMor	emb|CAEA01535031.1|:3377-3907
NuwaI_GMor	emb|CAEA01374928.1|:1-553
NuwaI_GMor	emb|CAEA01002261.1|:1-502
NuwaI_GMor	emb|CAEA01550154.1|:34-591
NuwaI_GMor	emb|CAEA01369470.1|:1-698
NuwaI_GMor	emb|CAEA01373444.1|:1-543
NuwaI_GMor	emb|CAEA01130878.1|:2-453
NuwaI_GMor	emb|CAEA01393664.1|:2194-2674
NuwaI_GMor	emb|CAEA01113291.1|:2-453
NuwaI_GMor	emb|CAEA01531062.1|:3105-3576
NuwaI_GMor	emb|CAEA01475357.1|:1-443
NuwaI_GMor	emb|CAEA01550399.1|:1-427
NuwaI_GMor	emb|CAEA01270344.1|:14-461
NuwaI_GMor	emb|CAEA01132211.1|:1-421
NuwaI_GMor	emb|CAEA01137855.1|:1-428
NuwaI_GMor	emb|CAEA01463312.1|:1417-1885
NuwaI_GMor	emb|CAEA01444592.1|:1-426
NuwaI_GMor	emb|CAEA01287282.1|:9-454
NuwaI_GMor	emb|CAEA01480879.1|:489-891
NuwaI_GMor	emb|CAEA01130963.1|:196-647
NuwaI_GMor	emb|CAEA01031516.1|:43-430
NuwaI_GMor	emb|CAEA01494842.1|:1688-2071
NuwaI_GMor	emb|CAEA01478145.1|:12-398
NuwaI_GMor	emb|CAEA01395899.1|:53-439
NuwaI_GMor	emb|CAEA01250084.1|:82-468
NuwaI_GMor	emb|CAEA01226504.1|:50-436
NuwaI_GMor	emb|CAEA01121297.1|:44-430
NuwaI_GMor	emb|CAEA01470351.1|:5-375
NuwaI_GMor	emb|CAEA01544025.1|:3859-4354
NuwaI_GMor	emb|CAEA01403341.1|:1-367

NuwaI_GMor	emb|CAEA01406123.1|:8-383
NuwaI_GMor	emb|CAEA01385906.1|:139-513
NuwaI_GMor	emb|CAEA01449775.1|:493-873
NuwaI_GMor	emb|CAEA01003058.1|:38-378
NuwaI_GMor	emb|CAEA01137544.1|:1-340
NuwaI_GMor	emb|CAEA01142759.1|:5-334
NuwaI_GMor	emb|CAEA01474336.1|:46-433
NuwaI_GMor	emb|CAEA01489181.1|:1-323
NuwaI_GMor	emb|CAEA01154425.1|:4-319
NuwaI_GMor	emb|CAEA01469774.1|:40-357
NuwaI_GMor	emb|CAEA01210728.1|:1-312
NuwaI_GMor	emb|CAEA01168623.1|:1-312
NuwaI_GMor	emb|CAEA01467406.1|:1010-1320
NuwaI_GMor	emb|CAEA01067725.1|:1-301
NuwaI_GMor	emb|CAEA01487757.1|:86-378
NuwaI_GMor	emb|CAEA01126687.1|:1-291
NuwaI_GMor	emb|CAEA01475798.1|:1-287
NuwaI_GMor	emb|CAEA01337056.1|:2564-2836
NuwaI_GMor	emb|CAEA01125183.1|:1-267
NuwaI_GMor	emb|CAEA01427203.1|:2-267
NuwaI_GMor	emb|CAEA01113600.1|:1-262
NuwaI_GMor	emb|CAEA01364317.1|:10-267
NuwaI_GMor	emb|CAEA01446716.1|:1-256
NuwaI_GMor	emb|CAEA01120046.1|:1976-2232
NuwaI_GMor	emb|CAEA01474964.1|:1-253
NuwaI_GMor	emb|CAEA01438825.1|:5089-5343
NuwaI_GMor	emb|CAEA01435215.1|:489-743
NuwaI_GMor	emb|CAEA01112246.1|:1-245
NuwaI_GMor	emb|CAEA01432278.1|:5-240
NuwaI_GMor	emb|CAEA01369203.1|:243-474
NuwaI_GMor	emb|CAEA01133839.1|:1912-2143
NuwaI_GMor	emb|CAEA01001286.1|:529-760
NuwaI_GMor	emb|CAEA01005920.1|:392-621
NuwaI_GMor	emb|CAEA01362857.1|:549-768
NuwaI_GMor	emb|CAEA01440276.1|:1-225
NuwaI_GMor	emb|CAEA01002668.1|:4074-4288
NuwaI_GMor	emb|CAEA01426971.1|:317-472
NuwaI_GMor	emb|CAEA01078409.1|:1940-2092
NuwaI_GMor	emb|CAEA01113780.1|:595-736
NuwaI_GMor	emb|CAEA01362759.1|:4565-6039
NuwaI_GMor	emb|CAEA01479547.1|:5-1889
NuwaI_GMor	emb|CAEA01531965.1|:1222-2584
NuwaI_GMor	emb|CAEA01541088.1|:3178-4444
NuwaI_GMor	emb|CAEA01153639.1|:667-1814

NuwaI_GMor	emb|CAEA01150301.1|:348-1460
NuwaI_GMor	emb|CAEA01131715.1|:4-1045
NuwaI_GMor	emb|CAEA01507564.1|:1-1046
NuwaI_GMor	emb|CAEA01487757.1|:2254-3276
NuwaI_GMor	emb|CAEA01142873.1|:951-3261
NuwaI_GMor	emb|CAEA01542694.1|:1-944
NuwaI_GMor	emb|CAEA01123934.1|:2-987
NuwaI_GMor	emb|CAEA01006149.1|:220-1233
NuwaI_GMor	emb|CAEA01219816.1|:5114-6134
NuwaI_GMor	emb|CAEA01458232.1|:30-992
NuwaI_GMor	emb|CAEA01544308.1|:1103-3322
NuwaI_GMor	emb|CAEA01138731.1|:5017-6027
NuwaI_GMor	emb|CAEA01156563.1|:500-1534
NuwaI_GMor	emb|CAEA01396468.1|:21-973
NuwaI_GMor	emb|CAEA01496137.1|:47-876
NuwaI_GMor	emb|CAEA01299389.1|:38-844
NuwaI_GMor	emb|CAEA01346985.1|:425-1263
NuwaI_GMor	emb|CAEA01155371.1|:1-892
NuwaI_GMor	emb|CAEA01437835.1|:1-870
NuwaI_GMor	emb|CAEA01129612.1|:2407-3497
NuwaI_GMor	emb|CAEA01390361.1|:8-794
NuwaI_GMor	emb|CAEA01545925.1|:1-711
NuwaI_GMor	emb|CAEA01078409.1|:1212-2128
NuwaI_GMor	emb|CAEA01131719.1|:1-719
NuwaI_GMor	emb|CAEA01487722.1|:8025-8820
NuwaI_GMor	emb|CAEA01130471.1|:1-667
NuwaI_GMor	emb|CAEA01184934.1|:26-755
NuwaI_GMor	emb|CAEA01210730.1|:413-1129
NuwaI_GMor	emb|CAEA01352527.1|:139-864
NuwaI_GMor	emb|CAEA01210732.1|:2-1784
NuwaI_GMor	emb|CAEA01426971.1|:25-851
NuwaI_GMor	emb|CAEA01476794.1|:1-644
NuwaI_GMor	emb|CAEA01206578.1|:478-1124
NuwaI_GMor	emb|CAEA01129473.1|:2-610
NuwaI_GMor	emb|CAEA01146635.1|:1-696
NuwaI_GMor	emb|CAEA01074368.1|:23-618
NuwaI_GMor	emb|CAEA01131812.1|:1-655
NuwaI_GMor	emb|CAEA01382667.1|:15-580
NuwaI_GMor	emb|CAEA01514017.1|:1837-2446
NuwaI_GMor	emb|CAEA01004200.1|:1-560
NuwaI_GMor	emb|CAEA01353363.1|:8-540
NuwaI_GMor	emb|CAEA01451211.1|:41-581
NuwaI_GMor	emb|CAEA01121451.1|:1-588
NuwaI_GMor	emb|CAEA01162460.1|:1-535

NuwaI_GMor	emb|CAEA01133063.1|:1-561
NuwaI_GMor	emb|CAEA01490193.1|:30-594
NuwaI_GMor	emb|CAEA01483796.1|:3975-4527
NuwaI_GMor	emb|CAEA01114372.1|:1-585
NuwaI_GMor	emb|CAEA01538549.1|:23302-23868
NuwaI_GMor	emb|CAEA01379851.1|:24-550
NuwaI_GMor	emb|CAEA01463340.1|:1-532
NuwaI_GMor	emb|CAEA01403567.1|:3-507
NuwaI_GMor	emb|CAEA01013878.1|:48-556
NuwaI_GMor	emb|CAEA01432993.1|:2497-3040
NuwaI_GMor	emb|CAEA01248082.1|:7-485
NuwaI_GMor	emb|CAEA01247712.1|:1-475
NuwaI_GMor	emb|CAEA01381339.1|:1-476
NuwaI_GMor	emb|CAEA01396171.1|:17-476
NuwaI_GMor	emb|CAEA01136810.1|:11-510
NuwaI_GMor	emb|CAEA01049598.1|:11-467
NuwaI_GMor	emb|CAEA01523584.1|:40-491
NuwaI_GMor	emb|CAEA01445287.1|:4-465
NuwaI_GMor	emb|CAEA01406847.1|:2-445
NuwaI_GMor	emb|CAEA01421254.1|:1-431
NuwaI_GMor	emb|CAEA01497755.1|:27-462
NuwaI_GMor	emb|CAEA01343374.1|:2-486
NuwaI_GMor	emb|CAEA01432994.1|:1-434
NuwaI_GMor	emb|CAEA01151014.1|:1790-2264
NuwaI_GMor	emb|CAEA01406574.1|:1-439
NuwaI_GMor	emb|CAEA01001382.1|:1-434
NuwaI_GMor	emb|CAEA01404922.1|:9-401
NuwaI_GMor	emb|CAEA01407020.1|:36-434
NuwaI_GMor	emb|CAEA01442060.1|:1-387
NuwaI_GMor	emb|CAEA01114509.1|:107-493
NuwaI_GMor	emb|CAEA01001019.1|:2004-2390
NuwaI_GMor	emb|CAEA01382732.1|:1-383
NuwaI_GMor	emb|CAEA01133874.1|:2-412
NuwaI_GMor	emb|CAEA01263137.1|:2448-2840
NuwaI_GMor	emb|CAEA01452304.1|:1-383
NuwaI_GMor	emb|CAEA01511728.1|:7-389
NuwaI_GMor	emb|CAEA01486025.1|:24-424
NuwaI_GMor	emb|CAEA01042976.1|:4272-4639
NuwaI_GMor	emb|CAEA01068717.1|:33-399
NuwaI_GMor	emb|CAEA01001412.1|:78-497
NuwaI_GMor	emb|CAEA01113500.1|:1-352
NuwaI_GMor	emb|CAEA01120963.1|:18-361
NuwaI_GMor	emb|CAEA01131804.1|:1-421
NuwaI_GMor	emb|CAEA01427348.1|:64-408

NuwaI_GMor	emb|CAEA01357899.1|:3624-3977
NuwaI_GMor	emb|CAEA01138877.1|:2-375
NuwaI_GMor	emb|CAEA01431490.1|:32-359
NuwaI_GMor	emb|CAEA01117187.1|:1-322
NuwaI_GMor	emb|CAEA01184935.1|:3-332
NuwaI_GMor	emb|CAEA01219567.1|:1106-1423
NuwaI_GMor	emb|CAEA01111966.1|:1-313
NuwaI_GMor	emb|CAEA01432838.1|:88-403
NuwaI_GMor	emb|CAEA01001753.1|:10-328
NuwaI_GMor	emb|CAEA01459657.1|:773-1072
NuwaI_GMor	emb|CAEA01474393.1|:1-305
NuwaI_GMor	emb|CAEA01376966.1|:4-304
NuwaI_GMor	emb|CAEA01124194.1|:1-296
NuwaI_GMor	emb|CAEA01465244.1|:7-300
NuwaI_GMor	emb|CAEA01366654.1|:362-656
NuwaI_GMor	emb|CAEA01149633.1|:1-293
NuwaI_GMor	emb|CAEA01184933.1|:2364-2654
NuwaI_GMor	emb|CAEA01101754.1|:4-286
NuwaI_GMor	emb|CAEA01004993.1|:3-283
NuwaI_GMor	emb|CAEA01115690.1|:4686-4965
NuwaI_GMor	emb|CAEA01028536.1|:151-425
NuwaI_GMor	emb|CAEA01275611.1|:15-291
NuwaI_GMor	emb|CAEA01534137.1|:137-438
NuwaI_GMor	emb|CAEA01142210.1|:1-272
NuwaI_GMor	emb|CAEA01394679.1|:385-640
NuwaI_GMor	emb|CAEA01056307.1|:1-256
NuwaI_GMor	emb|CAEA01130963.1|:647-928
NuwaI_GMor	emb|CAEA01369560.1|:1-253
NuwaI_GMor	emb|CAEA01272948.1|:15-269
NuwaI_GMor	emb|CAEA01113780.1|:9-262
NuwaI_GMor	emb|CAEA01465801.1|:24-279
NuwaI_GMor	emb|CAEA01028113.1|:16-266
NuwaI_GMor	emb|CAEA01137631.1|:564-811
NuwaI_GMor	emb|CAEA01449760.1|:1-240
NuwaI_GMor	emb|CAEA01527541.1|:1-238
NuwaI_GMor	emb|CAEA01136124.1|:12-249
NuwaI_GMor	emb|CAEA01475750.1|:7-238
NuwaI_GMor	emb|CAEA01386098.1|:7-235
NuwaI_GMor	emb|CAEA01536357.1|:1-210
NuwaI_GMor	emb|CAEA01155522.1|:1-188
NuwaI_GMor	emb|CAEA01369470.1|:297-453
NuwaI_GMor	emb|CAEA01439188.1|:518-632
NuwaI_GMor	emb|CAEA01137883.1|:1-94
NuwaI_GMor	emb|CAEA01544025.1|:4152-4240

Gasterosteus aculeatus	*NuwaI_GAcu*	gb|AANH01002764.1|:730-4541
	NuwaI_GAcu	gb|AANH01007278.1|:156302-160090
	NuwaI_GAcu	gb|AANH01007814.1|:166604-170403
	NuwaI_GAcu	gb|AANH01010679.1|:18574-22372
	NuwaI_GAcu	gb|AANH01002892.1|:19514-23155
	NuwaI_GAcu	gb|AANH01009787.1|:110697-114039
	NuwaI_GAcu	gb|AANH01010542.1|:52727-55780
	NuwaI_GAcu	gb|AANH01014597.1|:5960-8185
	NuwaI_GAcu	gb|AANH01001740.1|:68618-70844
	NuwaI_GAcu	gb|AANH01007931.1|:5584-7595
	NuwaI_GAcu	gb|AANH01007932.1|:5936-7947
	NuwaI_GAcu	gb|AANH01014660.1|:1-1598
	NuwaI_GAcu	gb|AANH01002643.1|:5601-7074
	NuwaI_GAcu	gb|AANH01004580.1|:147148-148618
	NuwaI_GAcu	gb|AANH01005212.1|:17785-19151
	NuwaI_GAcu	gb|AANH01001599.1|:29529-30895
	NuwaI_GAcu	gb|AANH01009813.1|:13758-15117
	NuwaI_GAcu	gb|AANH01007930.1|:1-542
	NuwaI_GAcu	gb|AANH01002862.1|:93254-96463
	NuwaI_GAcu	gb|AANH01003772.1|:101619-103486
	NuwaI_GAcu	gb|AANH01000918.1|:12478-13579
	NuwaI_GAcu	gb|AANH01001499.1|:64791-68065
	NuwaI_GAcu	gb|AANH01001610.1|:29113-30424
	NuwaI_GAcu	gb|AANH01011648.1|:11041-14351
	NuwaI_GAcu	gb|AANH01006472.1|:435719-437324
	NuwaI_GAcu	gb|AANH01006919.1|:6021-7520
	NuwaI_GAcu	gb|AANH01007831.1|:34606-36582
	NuwaI_GAcu	gb|AANH01010996.1|:5948-6208
	NuwaI_GAcu	gb|AANH01009832.1|:16143-17848
	NuwaI_GAcu	gb|AANH01011204.1|:131336-132698
	NuwaI_GAcu	gb|AANH01007313.1|:117742-118269
	NuwaI_GAcu	gb|AANH01000630.1|:108648-110608
	NuwaI_GAcu	gb|AANH01002757.1|:302512-303004
	NuwaI_GAcu	gb|AANH01002553.1|:7847-9114
	NuwaI_GAcu	gb|AANH01009712.1|:66892-68023
	NuwaI_GAcu	gb|AANH01001387.1|:39992-40861
	NuwaI_GAcu	gb|AANH01005358.1|:62539-63178
	NuwaI_GAcu	gb|AANH01003775.1|:140059-140267
	NuwaI_GAcu	gb|AANH01000455.1|:27085-27457
	NuwaI_GAcu	gb|AANH01011201.1|:133128-133327
	NuwaI_GAcu	gb|AANH01003010.1|:39557-40284
	NuwaI_GAcu	gb|AANH01007281.1|:30663-31689
	NuwaI_GAcu	gb|AANH01002465.1|:43872-44048
	NuwaI_GAcu	gb|AANH01000512.1|:91156-92297

NuwaI_GAcu	gb|AANH01008796.1|:30416-30593
NuwaI_GAcu	gb|AANH01004681.1|:125675-125868
NuwaI_GAcu	gb|AANH01010482.1|:2429-5247
NuwaI_GAcu	gb|AANH01006041.1|:216130-217113
NuwaI_GAcu	gb|AANH01007132.1|:58235-58521
NuwaI_GAcu	gb|AANH01010928.1|:194447-199766
NuwaI_GAcu	gb|AANH01010473.1|:6476-8579
NuwaI_GAcu	gb|AANH01013024.1|:8402-8806
NuwaI_GAcu	gb|AANH01010480.1|:12156-18165
NuwaI_GAcu	gb|AANH01006896.1|:1593-2560
NuwaI_GAcu	gb|AANH01004280.1|:59846-66902
NuwaI_GAcu	gb|AANH01004238.1|:27802-27920
NuwaI_GAcu	gb|AANH01009791.1|:86163-86283
NuwaI_GAcu	gb|AANH01010367.1|:88401-88519
NuwaI_GAcu	gb|AANH01003022.1|:92701-94412
NuwaI_GAcu	gb|AANH01000084.1|:7049-7162
NuwaI_GAcu	gb|AANH01007532.1|:25249-25770
NuwaI_GAcu	gb|AANH01008747.1|:100684-101599
NuwaI_GAcu	gb|AANH01001507.1|:11326-11830
NuwaI_GAcu	gb|AANH01002845.1|:48898-49001
NuwaI_GAcu	gb|AANH01008611.1|:21602-22415
NuwaI_GAcu	gb|AANH01001501.1|:98320-98420
NuwaI_GAcu	gb|AANH01008716.1|:112367-112468
NuwaI_GAcu	gb|AANH01004541.1|:24527-24625
NuwaI_GAcu	gb|AANH01014572.1|:4048-4149
NuwaI_GAcu	gb|AANH01002437.1|:11682-11779
NuwaI_GAcu	gb|AANH01002437.1|:58231-58326
NuwaI_GAcu	gb|AANH01004214.1|:26333-26422
NuwaI_GAcu	gb|AANH01012378.1|:10518-10605
NuwaI_GAcu	gb|AANH01002437.1|:25237-25323
NuwaI_GAcu	gb|AANH01005457.1|:474408-475484
NuwaI_GAcu	gb|AANH01009835.1|:13165-13250
NuwaI_GAcu	gb|AANH01009702.1|:166538-166686
NuwaI_GAcu	gb|AANH01010010.1|:59975-60057
NuwaI_GAcu	gb|AANH01007819.1|:35040-36192
NuwaI_GAcu	gb|AANH01007296.1|:38667-38748
NuwaI_GAcu	gb|AANH01016123.1|:2342-2419
NuwaI_GAcu	gb|AANH01015903.1|:2355-2432
NuwaI_GAcu	gb|AANH01010938.1|:27094-27169
NuwaI_GAcu	gb|AANH01010477.1|:7086-8337
NuwaI_GAcu	gb|AANH01005292.1|:15590-15663
NuwaI_GAcu	gb|AANH01004319.1|:47686-47758
NuwaI_GAcu	gb|AANH01010047.1|:99906-100140
NuwaI_GAcu	gb|AANH01009235.1|:32946-33016

NuwaI_GAcu	gb|AANH01002856.1|:20559-20797
NuwaI_GAcu	gb|AANH01007928.1|:23031-23097
NuwaI_GAcu	gb|AANH01009264.1|:77343-77406
NuwaI_GAcu	gb|AANH01000132.1|:73396-73459
NuwaI_GAcu	gb|AANH01010935.1|:70938-71000
NuwaI_GAcu	gb|AANH01009764.1|:30152-30211
NuwaI_GAcu	gb|AANH01010466.1|:67018-67077
NuwaI_GAcu	gb|AANH01009717.1|:12328-12386
NuwaI_GAcu	gb|AANH01007542.1|:6625-6682
NuwaI_GAcu	gb|AANH01004065.1|:4539-4596
NuwaI_GAcu	gb|AANH01009739.1|:92601-92658
NuwaI_GAcu	gb|AANH01000940.1|:91172-91229
NuwaI_GAcu	gb|AANH01003115.1|:97514-97571
NuwaI_GAcu	gb|AANH01003756.1|:42680-42733
NuwaI_GAcu	gb|AANH01010479.1|:8016-8071
NuwaI_GAcu	gb|AANH01010479.1|:31609-31664
NuwaI_GAcu	gb|AANH01012723.1|:54143-54196
NuwaI_GAcu	gb|AANH01006034.1|:9465-9516
NuwaI_GAcu	gb|AANH01001495.1|:16493-16543
NuwaI_GAcu	gb|AANH01012680.1|:12954-13003
NuwaI_GAcu	gb|AANH01014853.1|:1471-5271
NuwaI_GAcu	gb|AANH01001213.1|:41416-45203
NuwaI_GAcu	gb|AANH01009965.1|:10555-14343
NuwaI_GAcu	gb|AANH01008796.1|:3830-7608
NuwaI_GAcu	gb|AANH01011214.1|:10919-14716
NuwaI_GAcu	gb|AANH01003773.1|:24442-28233
NuwaI_GAcu	gb|AANH01014743.1|:1584-5302
NuwaI_GAcu	gb|AANH01003170.1|:35783-39489
NuwaI_GAcu	gb|AANH01006016.1|:3191-6765
NuwaI_GAcu	gb|AANH01015604.1|:4653-7828
NuwaI_GAcu	gb|AANH01002606.1|:1-2218
NuwaI_GAcu	gb|AANH01007784.1|:103995-105468
NuwaI_GAcu	gb|AANH01007688.1|:280134-281607
NuwaI_GAcu	gb|AANH01012843.1|:259-1733
NuwaI_GAcu	gb|AANH01000919.1|:6037-10289
NuwaI_GAcu	gb|AANH01004421.1|:25096-26462
NuwaI_GAcu	gb|AANH01002465.1|:40759-42125
NuwaI_GAcu	gb|AANH01008549.1|:83222-84588
NuwaI_GAcu	gb|AANH01004347.1|:129416-130782
NuwaI_GAcu	gb|AANH01010373.1|:33724-35089
NuwaI_GAcu	gb|AANH01003045.1|:167736-169102
NuwaI_GAcu	gb|AANH01015711.1|:2397-4193
NuwaI_GAcu	gb|AANH01008878.1|:140983-142349
NuwaI_GAcu	gb|AANH01015592.1|:1-1273

NuwaI_GAcu	gb|AANH01008691.1|:17129-18257
NuwaI_GAcu	gb|AANH01016104.1|:4654-5303
NuwaI_GAcu	gb|AANH01006466.1|:14607-18308
NuwaI_GAcu	gb|AANH01000518.1|:51740-53727
NuwaI_GAcu	gb|AANH01007293.1|:101167-101768
NuwaI_GAcu	gb|AANH01000396.1|:286022-287273
NuwaI_GAcu	gb|AANH01005797.1|:27548-28370
NuwaI_GAcu	gb|AANH01012111.1|:3912-4883
NuwaI_GAcu	gb|AANH01000512.1|:330624-330877
NuwaI_GAcu	gb|AANH01000918.1|:18629-20417
NuwaI_GAcu	gb|AANH01008892.1|:66409-67726
NuwaI_GAcu	gb|AANH01010273.1|:81398-88782
NuwaI_GAcu	gb|AANH01010115.1|:11238-13213
NuwaI_GAcu	gb|AANH01001499.1|:42228-42443
NuwaI_GAcu	gb|AANH01008628.1|:110573-110772
NuwaI_GAcu	gb|AANH01000477.1|:48261-48992
NuwaI_GAcu	gb|AANH01007278.1|:28239-29307
NuwaI_GAcu	gb|AANH01000484.1|:5774-6447
NuwaI_GAcu	gb|AANH01002325.1|:20904-25988
NuwaI_GAcu	gb|AANH01015028.1|:5453-5632
NuwaI_GAcu	gb|AANH01005616.1|:101519-101694
NuwaI_GAcu	gb|AANH01007191.1|:11338-12543
NuwaI_GAcu	gb|AANH01001624.1|:13780-14960
NuwaI_GAcu	gb|AANH01000499.1|:88915-90136
NuwaI_GAcu	gb|AANH01005538.1|:23587-23745
NuwaI_GAcu	gb|AANH01005882.1|:58529-58886
NuwaI_GAcu	gb|AANH01000415.1|:10931-11841
NuwaI_GAcu	gb|AANH01008624.1|:2911-3063
NuwaI_GAcu	gb|AANH01002886.1|:180163-180308
NuwaI_GAcu	gb|AANH01007662.1|:3904-4048
NuwaI_GAcu	gb|AANH01006983.1|:47977-48717
NuwaI_GAcu	gb|AANH01012152.1|:128214-128348
NuwaI_GAcu	gb|AANH01011210.1|:9054-9248
NuwaI_GAcu	gb|AANH01006891.1|:52980-59264
NuwaI_GAcu	gb|AANH01013164.1|:38264-38391
NuwaI_GAcu	gb|AANH01010363.1|:50621-51171
NuwaI_GAcu	gb|AANH01012024.1|:110967-112607
NuwaI_GAcu	gb|AANH01008215.1|:27208-28249
NuwaI_GAcu	gb|AANH01010793.1|:71798-71916
NuwaI_GAcu	gb|AANH01011599.1|:14635-14755
NuwaI_GAcu	gb|AANH01000502.1|:77171-77290
NuwaI_GAcu	gb|AANH01000504.1|:7826-9412
NuwaI_GAcu	gb|AANH01002637.1|:91064-91182
NuwaI_GAcu	gb|AANH01008873.1|:1929-2046

NuwaI_GAcu	gb|AANH01006653.1|:18829-18945
NuwaI_GAcu	gb|AANH01009722.1|:15324-15439
NuwaI_GAcu	gb|AANH01006476.1|:43943-44840
NuwaI_GAcu	gb|AANH01007261.1|:7115-7226
NuwaI_GAcu	gb|AANH01002849.1|:9735-9881
NuwaI_GAcu	gb|AANH01007281.1|:32279-34310
NuwaI_GAcu	gb|AANH01008892.1|:81621-81792
NuwaI_GAcu	gb|AANH01012882.1|:6494-6600
NuwaI_GAcu	gb|AANH01008704.1|:22988-23088
NuwaI_GAcu	gb|AANH01016195.1|:1396-1493
NuwaI_GAcu	gb|AANH01006463.1|:38065-38163
NuwaI_GAcu	gb|AANH01008716.1|:122595-123040
NuwaI_GAcu	gb|AANH01007270.1|:19223-19316
NuwaI_GAcu	gb|AANH01011213.1|:49386-49477
NuwaI_GAcu	gb|AANH01009744.1|:105513-105605
NuwaI_GAcu	gb|AANH01010459.1|:6168-6256
NuwaI_GAcu	gb|AANH01003734.1|:9264-9352
NuwaI_GAcu	gb|AANH01005582.1|:251178-251265
NuwaI_GAcu	gb|AANH01012882.1|:59936-60023
NuwaI_GAcu	gb|AANH01004541.1|:13105-13190
NuwaI_GAcu	gb|AANH01007275.1|:34180-34764
NuwaI_GAcu	gb|AANH01008215.1|:12255-12996
NuwaI_GAcu	gb|AANH01004214.1|:23522-23604
NuwaI_GAcu	gb|AANH01009702.1|:204634-204716
NuwaI_GAcu	gb|AANH01005586.1|:23128-23274
NuwaI_GAcu	gb|AANH01005212.1|:24019-24096
NuwaI_GAcu	gb|AANH01007260.1|:33653-33730
NuwaI_GAcu	gb|AANH01003147.1|:4798-4875
NuwaI_GAcu	gb|AANH01001666.1|:307814-307890
NuwaI_GAcu	gb|AANH01010935.1|:71067-71142
NuwaI_GAcu	gb|AANH01007837.1|:124008-124083
NuwaI_GAcu	gb|AANH01007048.1|:4523-4591
NuwaI_GAcu	gb|AANH01010679.1|:92936-93006
NuwaI_GAcu	gb|AANH01013128.1|:10402-10470
NuwaI_GAcu	gb|AANH01006424.1|:55318-55379
NuwaI_GAcu	gb|AANH01013413.1|:8924-8983
NuwaI_GAcu	gb|AANH01011976.1|:1094-1152
NuwaI_GAcu	gb|AANH01007842.1|:26669-26728
NuwaI_GAcu	gb|AANH01009764.1|:121845-121903
NuwaI_GAcu	gb|AANH01006509.1|:425-483
NuwaI_GAcu	gb|AANH01013871.1|:18015-18073
NuwaI_GAcu	gb|AANH01012076.1|:3936-3994
NuwaI_GAcu	gb|AANH01011496.1|:3219-3277
NuwaI_GAcu	gb|AANH01001404.1|:19700-19758

	NuwaI_GAcu	gb|AANH01012331.1|:26924-26982
	NuwaI_GAcu	gb|AANH01002717.1|:27951-28009
	NuwaI_GAcu	gb|AANH01007054.1|:49932-49989
	NuwaI_GAcu	gb|AANH01009807.1|:39192-39249
	NuwaI_GAcu	gb|AANH01002893.1|:171438-171495
	NuwaI_GAcu	gb|AANH01007661.1|:3287-3407
	NuwaI_GAcu	gb|AANH01006433.1|:15044-15100
	NuwaI_GAcu	gb|AANH01015218.1|:3401-3457
	NuwaI_GAcu	gb|AANH01005793.1|:16294-16350
	NuwaI_GAcu	gb|AANH01007648.1|:55940-55995
	NuwaI_GAcu	gb|AANH01007648.1|:30570-30625
	NuwaI_GAcu	gb|AANH01000473.1|:180249-180303
	NuwaI_GAcu	gb|AANH01012190.1|:19716-19769
	NuwaI_GAcu	gb|AANH01000494.1|:19977-20030
	NuwaI_GAcu	gb|AANH01003564.1|:16042-16094
	NuwaI_GAcu	gb|AANH01010764.1|:2326-2377
Leptinotarsa decemlineata	*NuwaI_LDec*	gb|AYNB01261911.1|:2520-5178
	NuwaI_LDec	gb|AYNB01170011.1|:4391-7018
	NuwaI_LDec	gb|AYNB01308641.1|:10055-12608
	NuwaI_LDec	gb|AYNB01214088.1|:1750-4165
	NuwaI_LDec	gb|AYNB01103230.1|:3124-5508
	NuwaI_LDec	gb|AYNB01151821.1|:169-2312
	NuwaI_LDec	gb|AYNB01059409.1|:1-2008
	NuwaI_LDec	gb|AYNB01236706.1|:1-1989
	NuwaI_LDec	gb|AYNB01011530.1|:1-1431
	NuwaI_LDec	gb|AYNB01007869.1|:1151-2313
	NuwaI_LDec	gb|AYNB01152849.1|:850-2001
	NuwaI_LDec	gb|AYNB01236705.1|:1-937
	NuwaI_LDec	gb|AYNB01195565.1|:2457-3271
	NuwaI_LDec	gb|AYNB01301582.1|:176-987
	NuwaI_LDec	gb|AYNB01214087.1|:3015-3737
	NuwaI_LDec	gb|AYNB01104213.1|:5-748
	NuwaI_LDec	gb|AYNB01195566.1|:1-670
	NuwaI_LDec	gb|AYNB01014089.1|:98-717
	NuwaI_LDec	gb|AYNB01226749.1|:1-531
	NuwaI_LDec	gb|AYNB01291323.1|:1-481
	NuwaI_LDec	gb|AYNB01292036.1|:1-380
	NuwaI_LDec	gb|AYNB01007870.1|:1-323
	NuwaI_LDec	gb|AYNB01088258.1|:1-327
	NuwaI_LDec	gb|AYNB01041745.1|:1968-2285
	NuwaI_LDec	gb|AYNB01301581.1|:1-320
	NuwaI_LDec	gb|AYNB01195567.1|:1-312
	NuwaI_LDec	gb|AYNB01059410.1|:31-320
	NuwaI_LDec	gb|AYNB01294320.1|:3-290

	NuwaI_LDec	gb|AYNB01104212.1|:4-285
	NuwaI_LDec	gb|AYNB01011529.1|:1059-1302
	NuwaI_LDec	gb|AYNB01014088.1|:1-130
	NuwaI_LDec	gb|AYNB01236704.1|:1067-1153
	NuwaI_LDec	gb|AYNB01062937.1|:2644-5347
	NuwaI_LDec	gb|AYNB01054722.1|:38-3453
	NuwaI_LDec	gb|AYNB01007141.1|:25-1997
	NuwaI_LDec	gb|AYNB01135157.1|:1-1904
	NuwaI_LDec	gb|AYNB01154216.1|:44-1758
	NuwaI_LDec	gb|AYNB01290227.1|:3-1237
	NuwaI_LDec	gb|AYNB01134008.1|:1667-2471
	NuwaI_LDec	gb|AYNB01011531.1|:1-704
	NuwaI_LDec	gb|AYNB01216646.1|:91-727
	NuwaI_LDec	gb|AYNB01011530.1|:1703-2215
	NuwaI_LDec	gb|AYNB01195431.1|:1-478
	NuwaI_LDec	gb|AYNB01134007.1|:6-487
	NuwaI_LDec	gb|AYNB01226750.1|:1-405
	NuwaI_LDec	gb|AYNB01257317.1|:1-385
	NuwaI_LDec	gb|AYNB01195432.1|:1-324
	NuwaI_LDec	gb|AYNB01210689.1|:1-344
	NuwaI_LDec	gb|AYNB01199405.1|:1-327
	NuwaI_LDec	gb|AYNB01291324.1|:1-303
	NuwaI_LDec	gb|AYNB01081464.1|:170-434
	NuwaI_LDec	gb|AYNB01135158.1|:1-200
	NuwaI_LDec	gb|AYNB01134006.1|:769-908
	NuwaI_LDec	gb|AYNB01210690.1|:1-134
	NuwaI_LDec	gb|AYNB01290228.1|:1-112
	NuwaI_LDec	gb|AYNB01125246.1|:1-104
	NuwaI_LDec	gb|AYNB01001877.1|:2364-2435
Melanochromis auratus	*NuwaI_MAur*	gb|ABPL01000763.1|:1372-4359
	NuwaI_MAur	gb|ABPL01000259.1|:1718-4703
	NuwaI_MAur	gb|ABPL01000487.1|:349-2846
	NuwaI_MAur	gb|ABPL01001819.1|:218-2694
	NuwaI_MAur	gb|ABPL01001804.1|:14-2504
	NuwaI_MAur	gb|ABPL01001138.1|:680-3166
	NuwaI_MAur	gb|ABPL01004462.1|:214-2593
	NuwaI_MAur	gb|ABPL01000472.1|:14-2314
	NuwaI_MAur	gb|ABPL01001577.1|:49-1947
	NuwaI_MAur	gb|ABPL01005554.1|:303-2076
	NuwaI_MAur	gb|ABPL01002722.1|:187-1785
	NuwaI_MAur	gb|ABPL01008891.1|:427-1757
	NuwaI_MAur	gb|ABPL01004780.1|:85-1382
	NuwaI_MAur	gb|ABPL01002554.1|:42-1337
	NuwaI_MAur	gb|ABPL01002616.1|:37-1334

NuwaI_MAur	gb|ABPL01001675.1|:14-1305
NuwaI_MAur	gb|ABPL01007265.1|:733-2011
NuwaI_MAur	gb|ABPL01002299.1|:67-1346
NuwaI_MAur	gb|ABPL01001012.1|:16-1297
NuwaI_MAur	gb|ABPL01000951.1|:37-1306
NuwaI_MAur	gb|ABPL01004032.1|:792-2117
NuwaI_MAur	gb|ABPL01002650.1|:1153-2446
NuwaI_MAur	gb|ABPL01003111.1|:994-2107
NuwaI_MAur	gb|ABPL01005732.1|:1184-2316
NuwaI_MAur	gb|ABPL01013593.1|:365-1454
NuwaI_MAur	gb|ABPL01040472.1|:28-1050
NuwaI_MAur	gb|ABPL01011697.1|:7-1438
NuwaI_MAur	gb|ABPL01035265.1|:1-983
NuwaI_MAur	gb|ABPL01010038.1|:27-1006
NuwaI_MAur	gb|ABPL01010168.1|:626-1593
NuwaI_MAur	gb|ABPL01036469.1|:53-1003
NuwaI_MAur	gb|ABPL01009409.1|:1-951
NuwaI_MAur	gb|ABPL01002928.1|:151-974
NuwaI_MAur	gb|ABPL01002540.1|:279-992
NuwaI_MAur	gb|ABPL01011530.1|:797-1505
NuwaI_MAur	gb|ABPL01072929.1|:46-709
NuwaI_MAur	gb|ABPL01082522.1|:1-645
NuwaI_MAur	gb|ABPL01003247.1|:139-782
NuwaI_MAur	gb|ABPL01035726.1|:60-683
NuwaI_MAur	gb|ABPL01052183.1|:47-708
NuwaI_MAur	gb|ABPL01002615.1|:8-539
NuwaI_MAur	gb|ABPL01010555.1|:1-487
NuwaI_MAur	gb|ABPL01068274.1|:363-808
NuwaI_MAur	gb|ABPL01033047.1|:1-435
NuwaI_MAur	gb|ABPL01010556.1|:1-435
NuwaI_MAur	gb|ABPL01001139.1|:1-434
NuwaI_MAur	gb|ABPL01000488.1|:1-434
NuwaI_MAur	gb|ABPL01001820.1|:1-434
NuwaI_MAur	gb|ABPL01004781.1|:1-434
NuwaI_MAur	gb|ABPL01009407.1|:387-822
NuwaI_MAur	gb|ABPL01002553.1|:631-1065
NuwaI_MAur	gb|ABPL01010037.1|:157-591
NuwaI_MAur	gb|ABPL01001803.1|:542-976
NuwaI_MAur	gb|ABPL01000471.1|:924-1357
NuwaI_MAur	gb|ABPL01000950.1|:752-1178
NuwaI_MAur	gb|ABPL01012042.1|:75-487
NuwaI_MAur	gb|ABPL01018221.1|:14-410
NuwaI_MAur	gb|ABPL01016582.1|:944-1330
NuwaI_MAur	gb|ABPL01001011.1|:757-1142

	NuwaI_MAur	gb|ABPL01007706.1|:1600-1930
	NuwaI_MAur	gb|ABPL01005615.1|:453-747
	NuwaI_MAur	gb|ABPL01005443.1|:145-426
	NuwaI_MAur	gb|ABPL01013525.1|:2-278
	NuwaI_MAur	gb|ABPL01009360.1|:1-233
	NuwaI_MAur	gb|ABPL01079918.1|:1-232
	NuwaI_MAur	gb|ABPL01083323.1|:435-659
	NuwaI_MAur	gb|ABPL01030694.1|:9-232
	NuwaI_MAur	gb|ABPL01000473.1|:211-398
	NuwaI_MAur	gb|ABPL01009874.1|:1447-1634
	NuwaI_MAur	gb|ABPL01033046.1|:5-182
	NuwaI_MAur	gb|ABPL01002300.1|:9-166
	NuwaI_MAur	gb|ABPL01039992.1|:877-1022
	NuwaI_MAur	gb|ABPL01018344.1|:1105-1247
	NuwaI_MAur	gb|ABPL01004388.1|:54-185
	NuwaI_MAur	gb|ABPL01001674.1|:1-104
	NuwaI_MAur	gb|ABPL01049324.1|:63-159
	NuwaI_MAur	gb|ABPL01077880.1|:27-117
	NuwaI_MAur	gb|ABPL01000152.1|:417-477
Nematostella vectensis	*NuwaI_NVec*	gb|ABAV01002819.1|:10606-15315
	NuwaI_NVec	gb|ABAV01004261.1|:8095-12405
	NuwaI_NVec	gb|ABAV01013602.1|:1-2464
	NuwaI_NVec	gb|ABAV01008431.1|:1718-3198
	NuwaI_NVec	gb|ABAV01013600.1|:1-1262
	NuwaI_NVec	gb|ABAV01013601.1|:1-1131
	NuwaI_NVec	gb|ABAV01013599.1|:950-2004
	NuwaI_NVec	gb|ABAV01019777.1|:26119-26725
	NuwaI_NVec	gb|ABAV01056840.1|:38-638
	NuwaI_NVec	gb|ABAV01007521.1|:18167-22562
	NuwaI_NVec	gb|ABAV01013603.1|:1-168
	NuwaI_NVec	gb|ABAV01011183.1|:8272-8371
	NuwaI_NVec	gb|ABAV01010169.1|:2650-2749
	NuwaI_NVec	gb|ABAV01016191.1|:19439-19549
	NuwaI_NVec	gb|ABAV01043773.1|:349-440
	NuwaI_NVec	gb|ABAV01002863.1|:11302-11388
	NuwaI_NVec	gb|ABAV01014247.1|:10925-10990
	NuwaI_NVec	gb|ABAV01034014.1|:126-180
	NuwaI_NVec	gb|ABAV01001030.1|:45271-45325
	NuwaI_NVec	gb|ABAV01009706.1|:9391-15286
	NuwaI_NVec	gb|ABAV01003252.1|:13948-21645
	NuwaI_NVec	gb|ABAV01047842.1|:1-197
	NuwaI_NVec	gb|ABAV01002849.1|:10583-10672
	NuwaI_NVec	gb|ABAV01005794.1|:16992-17089
	NuwaI_NVec	gb|ABAV01005532.1|:9126-9216

	NuwaI_NVec	gb|ABAV01013321.1|:27012-27098
	NuwaI_NVec	gb|ABAV01008006.1|:4776-4840
	NuwaI_NVec	gb|ABAV01053402.1|:886-940
	NuwaI_NVec	gb|ABAV01011128.1|:240-293
	NuwaI_NVec	gb|ABAV01000807.1|:14572-14625
	NuwaI_NVec	gb|ABAV01032527.1|:18-70
	NuwaI_NVec	gb|ABAV01023383.1|:4098-4148
Oreochromis niloticus	*NuwaI_ONil*	gb|AERX01065474.2|:4869-6731
	NuwaI_ONil	gb|AERX01067289.2|:1035-1941
	NuwaI_ONil	gb|AERX01047077.2|:23704-25301
	NuwaI_ONil	gb|AERX01047374.1|:862-1690
	NuwaI_ONil	gb|AERX01055748.2|:298-1843
	NuwaI_ONil	gb|AERX01032961.2|:25129-25840
	NuwaI_ONil	gb|AERX01047375.1|:1-696
	NuwaI_ONil	gb|AERX01075617.2|:1-681
	NuwaI_ONil	gb|AERX01035987.1|:6082-6677
	NuwaI_ONil	gb|AERX01052376.2|:6811-7367
	NuwaI_ONil	gb|AERX01034619.1|:3225-3770
	NuwaI_ONil	gb|AERX01050509.1|:5487-6074
	NuwaI_ONil	gb|AERX01067865.1|:39-588
	NuwaI_ONil	gb|AERX01054847.1|:1-1751
	NuwaI_ONil	gb|AERX01047516.1|:18898-19374
	NuwaI_ONil	gb|AERX01046788.2|:496-968
	NuwaI_ONil	gb|AERX01004888.1|:7818-8292
	NuwaI_ONil	gb|AERX01051853.1|:40-475
	NuwaI_ONil	gb|AERX01036101.2|:250-686
	NuwaI_ONil	gb|AERX01032962.2|:28-451
	NuwaI_ONil	gb|AERX01048800.1|:6539-6895
	NuwaI_ONil	gb|AERX01010644.1|:1132-1460
	NuwaI_ONil	gb|AERX01046890.1|:14-315
	NuwaI_ONil	gb|AERX01035988.1|:168-471
	NuwaI_ONil	gb|AERX01067302.2|:4014-4311
	NuwaI_ONil	gb|AERX01054846.2|:3661-3938
	NuwaI_ONil	gb|AERX01076381.1|:901-1328
	NuwaI_ONil	gb|AERX01034620.1|:1-236
	NuwaI_ONil	gb|AERX01010643.1|:3473-3702
	NuwaI_ONil	gb|AERX01004015.2|:2112-2290
	NuwaI_ONil	gb|AERX01034742.2|:2649-2826
	NuwaI_ONil	gb|AERX01046789.2|:1-168
	NuwaI_ONil	gb|AERX01046889.2|:8179-8343
	NuwaI_ONil	gb|AERX01066922.1|:1690-1850
	NuwaI_ONil	gb|AERX01067303.1|:20-174
	NuwaI_ONil	gb|AERX01052377.2|:10-164
	NuwaI_ONil	gb|AERX01050510.1|:20-174

NuwaI_ONil	gb|AERX01047517.2|:2-156
NuwaI_ONil	gb|AERX01069441.1|:535-689
NuwaI_ONil	gb|AERX01004889.1|:19-172
NuwaI_ONil	gb|AERX01036100.2|:1720-1864
NuwaI_ONil	gb|AERX01067864.1|:1422-1556
NuwaI_ONil	gb|AERX01017838.1|:5134-5228
NuwaI_ONil	gb|AERX01004016.1|:1-109
NuwaI_ONil	gb|AERX01026306.2|:5290-5396
NuwaI_ONil	gb|AERX01051852.1|:2819-2921
NuwaI_ONil	gb|AERX01076252.1|:1-56
NuwaI_ONil	gb|AERX01044663.2|:407-2136
NuwaI_ONil	gb|AERX01027269.2|:299-1742
NuwaI_ONil	gb|AERX01046459.2|:1-1273
NuwaI_ONil	gb|AERX01020827.2|:928-2154
NuwaI_ONil	gb|AERX01032726.2|:673-1935
NuwaI_ONil	gb|AERX01059522.2|:1372-2275
NuwaI_ONil	gb|AERX01046962.2|:927-1857
NuwaI_ONil	gb|AERX01045754.2|:1708-2639
NuwaI_ONil	gb|AERX01059521.1|:1653-2589
NuwaI_ONil	gb|AERX01005450.2|:1-776
NuwaI_ONil	gb|AERX01045755.1|:1-758
NuwaI_ONil	gb|AERX01062734.2|:4-718
NuwaI_ONil	gb|AERX01027476.1|:1-673
NuwaI_ONil	gb|AERX01047077.2|:22555-23260
NuwaI_ONil	gb|AERX01057150.1|:6579-7288
NuwaI_ONil	gb|AERX01053423.2|:1-646
NuwaI_ONil	gb|AERX01005449.2|:8086-8709
NuwaI_ONil	gb|AERX01046458.1|:12217-12821
NuwaI_ONil	gb|AERX01044110.1|:1-521
NuwaI_ONil	gb|AERX01009603.1|:1-508
NuwaI_ONil	gb|AERX01050883.1|:2341-2880
NuwaI_ONil	gb|AERX01015524.1|:8281-8827
NuwaI_ONil	gb|AERX01055210.1|:301-786
NuwaI_ONil	gb|AERX01057370.1|:5521-5956
NuwaI_ONil	gb|AERX01048797.1|:14627-15060
NuwaI_ONil	gb|AERX01035842.2|:6933-7317
NuwaI_ONil	gb|AERX01015525.1|:1-332
NuwaI_ONil	gb|AERX01040261.2|:4095-4394
NuwaI_ONil	gb|AERX01027475.1|:3515-3818
NuwaI_ONil	gb|AERX01058251.2|:2339-2634
NuwaI_ONil	gb|AERX01047903.2|:8096-8388
NuwaI_ONil	gb|AERX01050884.2|:1-290
NuwaI_ONil	gb|AERX01034743.1|:1-230
NuwaI_ONil	gb|AERX01040262.2|:1-230

	NuwaI_ONil	gb|AERX01027270.2|:1-203
	NuwaI_ONil	gb|AERX01035844.2|:1-198
	NuwaI_ONil	gb|AERX01055211.2|:1-167
	NuwaI_ONil	gb|AERX01046753.1|:1-167
	NuwaI_ONil	gb|AERX01044109.1|:2230-2384
	NuwaI_ONil	gb|AERX01009602.2|:2459-2613
	NuwaI_ONil	gb|AERX01048801.1|:1-148
	NuwaI_ONil	gb|AERX01001947.1|:1-145
	NuwaI_ONil	gb|AERX01075181.1|:1-126
	NuwaI_ONil	gb|AERX01048798.1|:1-103
	NuwaI_ONil	gb|AERX01058252.1|:1-102
	NuwaI_ONil	gb|AERX01057151.2|:1-102
	NuwaI_ONil	gb|AERX01001946.2|:3215-3306
	NuwaI_ONil	gb|AERX01068646.2|:1-89
	NuwaI_ONil	gb|AERX01047904.1|:1-88
	NuwaI_ONil	gb|AERX01055080.2|:692-773
	NuwaI_ONil	gb|AERX01055081.1|:1-72
	NuwaI_ONil	gb|AERX01044111.2|:1-70
	NuwaI_ONil	gb|AERX01062733.1|:4962-5016
Pundamilia nyererei	*NuwaI-1_PNye*	gb|AFNX01016362.1|:17812-18964
	NuwaI-1_PNye	gb|AFNX01033722.1|:25702-26817
	NuwaI-1_PNye	gb|AFNX01000429.1|:28680-29796
	NuwaI-1_PNye	gb|AFNX01036052.1|:2250-2728
	NuwaI-1_PNye	gb|AFNX01023336.1|:1-889
	NuwaI-1_PNye	gb|AFNX01053753.1|:1880-2775
	NuwaI-1_PNye	gb|AFNX01007656.1|:1379-2016
	NuwaI-1_PNye	gb|AFNX01036053.1|:991-1627
	NuwaI-1_PNye	gb|AFNX01021546.1|:816-1447
	NuwaI-1_PNye	gb|AFNX01006186.1|:1-322
	NuwaI-1_PNye	gb|AFNX01007655.1|:9588-9837
	NuwaI-1_PNye	gb|AFNX01036302.1|:1-236
	NuwaI-1_PNye	gb|AFNX01024991.1|:1-208
	NuwaI-1_PNye	gb|AFNX01013270.1|:10449-10630
	NuwaI-1_PNye	gb|AFNX01060519.1|:1-180
	NuwaI-1_PNye	gb|AFNX01055106.1|:1517-1684
	NuwaI-1_PNye	gb|AFNX01009622.1|:1-167
	NuwaI-1_PNye	gb|AFNX01059781.1|:2169-2332
	NuwaI-1_PNye	gb|AFNX01036301.1|:2209-2383
	NuwaI-1_PNye	gb|AFNX01055107.1|:1-166
	NuwaI-1_PNye	gb|AFNX01025935.1|:12541-12693
	NuwaI-1_PNye	gb|AFNX01046526.1|:6965-7097
	NuwaI-1_PNye	gb|AFNX01036221.1|:13974-14106
	NuwaI-1_PNye	gb|AFNX01024917.1|:9191-9320
	NuwaI-1_PNye	gb|AFNX01059782.1|:1-117

NuwaI-1_PNye	gb|AFNX01021796.1|:3376-3486
NuwaI-1_PNye	gb|AFNX01046527.1|:1-107
NuwaI-1_PNye	gb|AFNX01036222.1|:1-107
NuwaI-1_PNye	gb|AFNX01023335.1|:6815-6967
NuwaI-1_PNye	gb|AFNX01046591.1|:14269-14339
NuwaI-1_PNye	gb|AFNX01028093.1|:1780-1849
NuwaI-1_PNye	gb|AFNX01046592.1|:1-72
NuwaI-2_PNye	gb|AFNX01059683.1|:9609-11404
NuwaI-2_PNye	gb|AFNX01047949.1|:45189-46982
NuwaI-2_PNye	gb|AFNX01027200.1|:1794-3587
NuwaI-2_PNye	gb|AFNX01031130.1|:1892-3687
NuwaI-2_PNye	gb|AFNX01041158.1|:4148-5459
NuwaI-2_PNye	gb|AFNX01051206.1|:1009-2798
NuwaI-2_PNye	gb|AFNX01030958.1|:15605-17390
NuwaI-2_PNye	gb|AFNX01039344.1|:6503-8289
NuwaI-2_PNye	gb|AFNX01043500.1|:9292-11080
NuwaI-2_PNye	gb|AFNX01002014.1|:10398-12057
NuwaI-2_PNye	gb|AFNX01032001.1|:35333-37125
NuwaI-2_PNye	gb|AFNX01050571.1|:5491-6744
NuwaI-2_PNye	gb|AFNX01049991.1|:5204-7009
NuwaI-2_PNye	gb|AFNX01056007.1|:4529-6086
NuwaI-2_PNye	gb|AFNX01017323.1|:6540-7779
NuwaI-2_PNye	gb|AFNX01019968.1|:8017-9800
NuwaI-2_PNye	gb|AFNX01034976.1|:1-1364
NuwaI-2_PNye	gb|AFNX01038672.1|:7268-8969
NuwaI-2_PNye	gb|AFNX01023942.1|:9099-10872
NuwaI-2_PNye	gb|AFNX01056641.1|:20551-21858
NuwaI-2_PNye	gb|AFNX01059353.1|:3441-5019
NuwaI-2_PNye	gb|AFNX01056026.1|:1507-1977
NuwaI-2_PNye	gb|AFNX01036267.1|:1-408
NuwaI-2_PNye	gb|AFNX01003335.1|:1196-1521
NuwaI-2_PNye	gb|AFNX01050840.1|:1-382
NuwaI-2_PNye	gb|AFNX01060359.1|:1-322
NuwaI-2_PNye	gb|AFNX01035232.1|:51336-51641
NuwaI-2_PNye	gb|AFNX01005702.1|:5772-6062
NuwaI-2_PNye	gb|AFNX01003315.1|:423-1249
NuwaI-2_PNye	gb|AFNX01046273.1|:1-377
NuwaI-2_PNye	gb|AFNX01058063.1|:1235-1525
NuwaI-2_PNye	gb|AFNX01023781.1|:39542-39892
NuwaI-2_PNye	gb|AFNX01011387.1|:1-415
NuwaI-2_PNye	gb|AFNX01003511.1|:11151-11433
NuwaI-2_PNye	gb|AFNX01059027.1|:7396-7694
NuwaI-2_PNye	gb|AFNX01016547.1|:3693-4129
NuwaI-2_PNye	gb|AFNX01037975.1|:3039-3845

NuwaI-2_PNye	gb|AFNX01018541.1|:6270-6562
NuwaI-2_PNye	gb|AFNX01020117.1|:1-386
NuwaI-2_PNye	gb|AFNX01047231.1|:25594-25868
NuwaI-2_PNye	gb|AFNX01003337.1|:1-439
NuwaI-2_PNye	gb|AFNX01050137.1|:2795-3366
NuwaI-2_PNye	gb|AFNX01037673.1|:20-310
NuwaI-2_PNye	gb|AFNX01026302.1|:633-1018
NuwaI-2_PNye	gb|AFNX01036266.1|:2403-2668
NuwaI-2_PNye	gb|AFNX01030153.1|:1-290
NuwaI-2_PNye	gb|AFNX01022417.1|:343-396
NuwaI-2_PNye	gb|AFNX01022417.1|:1-313
NuwaI-2_PNye	gb|AFNX01008914.1|:1-396
NuwaI-2_PNye	gb|AFNX01053860.1|:6-254
NuwaI-2_PNye	gb|AFNX01006977.1|:9581-9856
NuwaI-2_PNye	gb|AFNX01055100.1|:4552-5179
NuwaI-2_PNye	gb|AFNX01042745.1|:1-406
NuwaI-2_PNye	gb|AFNX01035233.1|:1-249
NuwaI-2_PNye	gb|AFNX01017285.1|:1878-2137
NuwaI-2_PNye	gb|AFNX01043580.1|:1878-2132
NuwaI-2_PNye	gb|AFNX01039347.1|:3218-3452
NuwaI-2_PNye	gb|AFNX01030908.1|:6466-6728
NuwaI-2_PNye	gb|AFNX01009457.1|:1-226
NuwaI-2_PNye	gb|AFNX01016832.1|:1-224
NuwaI-2_PNye	gb|AFNX01040686.1|:1-230
NuwaI-2_PNye	gb|AFNX01018588.1|:1543-1764
NuwaI-2_PNye	gb|AFNX01054043.1|:1-251
NuwaI-2_PNye	gb|AFNX01048316.1|:1-237
NuwaI-2_PNye	gb|AFNX01057058.1|:1-234
NuwaI-2_PNye	gb|AFNX01064276.1|:24-312
NuwaI-2_PNye	gb|AFNX01051968.1|:10237-10488
NuwaI-2_PNye	gb|AFNX01056542.1|:3574-3804
NuwaI-2_PNye	gb|AFNX01042744.1|:1-279
NuwaI-2_PNye	gb|AFNX01030870.1|:33540-33724
NuwaI-2_PNye	gb|AFNX01030809.1|:26888-27071
NuwaI-2_PNye	gb|AFNX01004289.1|:2017-2196
NuwaI-2_PNye	gb|AFNX01030810.1|:1160-1346
NuwaI-2_PNye	gb|AFNX01051738.1|:177-374
NuwaI-2_PNye	gb|AFNX01000972.1|:14114-14293
NuwaI-2_PNye	gb|AFNX01052966.1|:3116-3637
NuwaI-2_PNye	gb|AFNX01058162.1|:1-198
NuwaI-2_PNye	gb|AFNX01009689.1|:6197-6381
NuwaI-2_PNye	gb|AFNX01002718.1|:1489-1659
NuwaI-2_PNye	gb|AFNX01065734.1|:1-202
NuwaI-2_PNye	gb|AFNX01065734.1|:228-303

NuwaI-2_PNye	gb|AFNX01005703.1|:1-177
NuwaI-2_PNye	gb|AFNX01040685.1|:1276-1455
NuwaI-2_PNye	gb|AFNX01042370.1|:5463-5645
NuwaI-2_PNye	gb|AFNX01031656.1|:5947-6125
NuwaI-2_PNye	gb|AFNX01051867.1|:1374-1710
NuwaI-2_PNye	gb|AFNX01060002.1|:1-177
NuwaI-2_PNye	gb|AFNX01049229.1|:1-180
NuwaI-2_PNye	gb|AFNX01043642.1|:1798-1977
NuwaI-2_PNye	gb|AFNX01062988.1|:4394-4571
NuwaI-2_PNye	gb|AFNX01054657.1|:1-166
NuwaI-2_PNye	gb|AFNX01057277.1|:1-165
NuwaI-2_PNye	gb|AFNX01056146.1|:1-181
NuwaI-2_PNye	gb|AFNX01035456.1|:9816-9984
NuwaI-2_PNye	gb|AFNX01030871.1|:1-181
NuwaI-2_PNye	gb|AFNX01035991.1|:1-176
NuwaI-2_PNye	gb|AFNX01030147.1|:5586-5753
NuwaI-2_PNye	gb|AFNX01032903.1|:1865-2025
NuwaI-2_PNye	gb|AFNX01055868.1|:1-162
NuwaI-2_PNye	gb|AFNX01000102.1|:3772-3953
NuwaI-2_PNye	gb|AFNX01062690.1|:2244-2399
NuwaI-2_PNye	gb|AFNX01055783.1|:4956-5114
NuwaI-2_PNye	gb|AFNX01004290.1|:1-158
NuwaI-2_PNye	gb|AFNX01016066.1|:1-161
NuwaI-2_PNye	gb|AFNX01054590.1|:2-161
NuwaI-2_PNye	gb|AFNX01051693.1|:1360-1537
NuwaI-2_PNye	gb|AFNX01007677.1|:5674-5835
NuwaI-2_PNye	gb|AFNX01010767.1|:44341-44530
NuwaI-2_PNye	gb|AFNX01044759.1|:45838-45993
NuwaI-2_PNye	gb|AFNX01048691.1|:1-159
NuwaI-2_PNye	gb|AFNX01029868.1|:1687-1864
NuwaI-2_PNye	gb|AFNX01006978.1|:1-180
NuwaI-2_PNye	gb|AFNX01008665.1|:1-149
NuwaI-2_PNye	gb|AFNX01030125.1|:52420-52563
NuwaI-2_PNye	gb|AFNX01024998.1|:5400-5543
NuwaI-2_PNye	gb|AFNX01024575.1|:1397-1526
NuwaI-2_PNye	gb|AFNX01017284.1|:23459-23587
NuwaI-2_PNye	gb|AFNX01054042.1|:5123-5312
NuwaI-2_PNye	gb|AFNX01057739.1|:1-129
NuwaI-2_PNye	gb|AFNX01056314.1|:6987-7151
NuwaI-2_PNye	gb|AFNX01042466.1|:1-128
NuwaI-2_PNye	gb|AFNX01057380.1|:1-127
NuwaI-2_PNye	gb|AFNX01056421.1|:7259-7385
NuwaI-2_PNye	gb|AFNX01066276.1|:1-122
NuwaI-2_PNye	gb|AFNX01065033.1|:1-186

NuwaI-2_PNye	gb|AFNX01043972.1|:10273-10394
NuwaI-2_PNye	gb|AFNX01059639.1|:1-188
NuwaI-2_PNye	gb|AFNX01058407.1|:1228-1345
NuwaI-2_PNye	gb|AFNX01050839.1|:8350-8471
NuwaI-2_PNye	gb|AFNX01058186.1|:1-149
NuwaI-2_PNye	gb|AFNX01065039.1|:1-114
NuwaI-2_PNye	gb|AFNX01063390.1|:1-114
NuwaI-2_PNye	gb|AFNX01056511.1|:5682-5803
NuwaI-2_PNye	gb|AFNX01056493.1|:1-114
NuwaI-2_PNye	gb|AFNX01039351.1|:4845-4958
NuwaI-2_PNye	gb|AFNX01030250.1|:1761-1874
NuwaI-2_PNye	gb|AFNX01000825.1|:1-114
NuwaI-2_PNye	gb|AFNX01049378.1|:8234-8370
NuwaI-2_PNye	gb|AFNX01030126.1|:1-117
NuwaI-2_PNye	gb|AFNX01062747.1|:1-111
NuwaI-2_PNye	gb|AFNX01055842.1|:9215-9372
NuwaI-2_PNye	gb|AFNX01066976.1|:1-114
NuwaI-2_PNye	gb|AFNX01062052.1|:4079-4192
NuwaI-2_PNye	gb|AFNX01054105.1|:1-114
NuwaI-2_PNye	gb|AFNX01049398.1|:1-114
NuwaI-2_PNye	gb|AFNX01031657.1|:1-114
NuwaI-2_PNye	gb|AFNX01058106.1|:1-109
NuwaI-2_PNye	gb|AFNX01048248.1|:14603-14710
NuwaI-2_PNye	gb|AFNX01031446.1|:963-1070
NuwaI-2_PNye	gb|AFNX01059794.1|:11729-11843
NuwaI-2_PNye	gb|AFNX01056196.1|:10050-10163
NuwaI-2_PNye	gb|AFNX01052504.1|:8121-8285
NuwaI-2_PNye	gb|AFNX01059693.1|:4517-4619
NuwaI-2_PNye	gb|AFNX01043973.1|:1-103
NuwaI-2_PNye	gb|AFNX01062117.1|:1-102
NuwaI-2_PNye	gb|AFNX01051695.1|:7-168
NuwaI-2_PNye	gb|AFNX01037672.1|:15139-15247
NuwaI-2_PNye	gb|AFNX01037346.1|:13192-13296
NuwaI-2_PNye	gb|AFNX01034975.1|:16387-16495
NuwaI-2_PNye	gb|AFNX01063055.1|:1-103
NuwaI-2_PNye	gb|AFNX01064258.1|:1-142
NuwaI-2_PNye	gb|AFNX01060854.1|:1-114
NuwaI-2_PNye	gb|AFNX01030221.1|:1-114
NuwaI-2_PNye	gb|AFNX01063233.1|:1245-1358
NuwaI-2_PNye	gb|AFNX01060870.1|:1236-1327
NuwaI-2_PNye	gb|AFNX01049589.1|:11206-11360
NuwaI-2_PNye	gb|AFNX01025647.1|:1-91
NuwaI-2_PNye	gb|AFNX01023782.1|:1-91
NuwaI-2_PNye	gb|AFNX01048249.1|:1-98

NuwaI-2_PNye	gb|AFNX01047232.1|:1-94
NuwaI-2_PNye	gb|AFNX01048932.1|:1-129
NuwaI-2_PNye	gb|AFNX01048689.1|:2247-2360
NuwaI-2_PNye	gb|AFNX01018589.1|:1-91
NuwaI-2_PNye	gb|AFNX01047558.1|:6254-6335
NuwaI-2_PNye	gb|AFNX01046031.1|:1-94
NuwaI-2_PNye	gb|AFNX01020118.1|:2156-2249
NuwaI-2_PNye	gb|AFNX01043643.1|:1-81
NuwaI-2_PNye	gb|AFNX01058657.1|:1-94
NuwaI-2_PNye	gb|AFNX01004816.1|:37305-37382
NuwaI-2_PNye	gb|AFNX01052449.1|:1-76
NuwaI-2_PNye	gb|AFNX01057013.1|:1-81
NuwaI-2_PNye	gb|AFNX01024979.1|:6054-6130
NuwaI-2_PNye	gb|AFNX01059424.1|:1-72
NuwaI-2_PNye	gb|AFNX01054562.1|:1298-1373
NuwaI-2_PNye	gb|AFNX01051616.1|:3444-3514
NuwaI-2_PNye	gb|AFNX01003336.1|:1-75
NuwaI-2_PNye	gb|AFNX01063624.1|:1921-2014
NuwaI-2_PNye	gb|AFNX01061390.1|:7-100
NuwaI-2_PNye	gb|AFNX01030152.1|:43762-43855
NuwaI-2_PNye	gb|AFNX01008913.1|:28653-28746
NuwaI-2_PNye	gb|AFNX01060133.1|:5446-5518
NuwaI-2_PNye	gb|AFNX01043014.1|:1-76
NuwaI-2_PNye	gb|AFNX01029196.1|:1-76
NuwaI-2_PNye	gb|AFNX01039348.1|:1-67
NuwaI-2_PNye	gb|AFNX01063234.1|:1-66
NuwaI-2_PNye	gb|AFNX01033579.1|:49692-49755
NuwaI-2_PNye	gb|AFNX01003314.1|:2336-2411
NuwaI-2_PNye	gb|AFNX01053309.1|:1202-1264
NuwaI-2_PNye	gb|AFNX01044104.1|:2093-2155
NuwaI-2_PNye	gb|AFNX01033580.1|:1-61
NuwaI-2_PNye	gb|AFNX01048299.1|:1-60
NuwaI-2_PNye	gb|AFNX01062616.1|:4860-4922
NuwaI-2_PNye	gb|AFNX01042743.1|:13047-13105
NuwaI-2_PNye	gb|AFNX01000221.1|:2160-2398
NuwaI-2_PNye	gb|AFNX01000135.1|:7715-7773
NuwaI-2_PNye	gb|AFNX01064610.1|:1-58
NuwaI-2_PNye	gb|AFNX01037501.1|:16343-16412
NuwaI-2_PNye	gb|AFNX01029195.1|:3017-3074
NuwaI-2_PNye	gb|AFNX01005449.1|:15-108
NuwaI-2_PNye	gb|AFNX01032927.1|:5883-5938
NuwaI-2_PNye	gb|AFNX01006251.1|:122-176
NuwaI-2_PNye	gb|AFNX01066243.1|:1-74
NuwaI-2_PNye	gb|AFNX01064918.1|:2051-2104

	NuwaI-2_PNye	gb|AFNX01052469.1|:1-70
	NuwaI-2_PNye	gb|AFNX01042235.1|:2515-2584
	NuwaI-2_PNye	gb|AFNX01018459.1|:1-54
	NuwaI-2_PNye	gb|AFNX01015974.1|:1-70
	NuwaI-2_PNye	gb|AFNX01036681.1|:9297-9368
	NuwaI-2_PNye	gb|AFNX01002719.1|:1-52
	NuwaI-2_PNye	gb|AFNX01028483.1|:1032-1082
	NuwaI-2_PNye	gb|AFNX01065020.1|:1-54
	NuwaI-2_PNye	gb|AFNX01049397.1|:2099-2168
	NuwaI-2_PNye	gb|AFNX01037502.1|:1-50
	NuwaI-2_PNye	gb|AFNX01032335.1|:1-66
	NuwaI-2_PNye	gb|AFNX01030220.1|:14889-14953
	NuwaI-2_PNye	gb|AFNX01042465.1|:6652-6703
	NuwaI-2_PNye	gb|AFNX01052560.1|:5-54
	NuwaI-2_PNye	gb|AFNX01042721.1|:9985-10038
	NuwaI-2_PNye	gb|AFNX01042236.1|:1-50
	NuwaI-2_PNye	gb|AFNX01031447.1|:1-50
	NuwaI-2_PNye	gb|AFNX01017745.1|:1-50
	NuwaI-2_PNye	gb|AFNX01051246.1|:1651-1715
	NuwaI-2_PNye	gb|AFNX01023582.1|:8730-8779
	NuwaI-2_PNye	gb|AFNX01036377.1|:1-53
Rhamphochromis esox	*NuwaI-1_REso*	gb|ABPN01000538.1|:48-3038
	NuwaI-1_REso	gb|ABPN01000340.1|:1-2507
	NuwaI-1_REso	gb|ABPN01000855.1|:859-3836
	NuwaI-1_REso	gb|ABPN01000036.1|:1042-3549
	NuwaI-1_REso	gb|ABPN01000846.1|:173-3149
	NuwaI-1_REso	gb|ABPN01001983.1|:422-2362
	NuwaI-1_REso	gb|ABPN01002442.1|:1310-3664
	NuwaI-1_REso	gb|ABPN01002433.1|:30-2899
	NuwaI-1_REso	gb|ABPN01001910.1|:1-1365
	NuwaI-1_REso	gb|ABPN01002695.1|:339-3511
	NuwaI-1_REso	gb|ABPN01002195.1|:2045-3293
	NuwaI-1_REso	gb|ABPN01018193.1|:44-1171
	NuwaI-1_REso	gb|ABPN01000190.1|:218-1448
	NuwaI-1_REso	gb|ABPN01006308.1|:1-1149
	NuwaI-1_REso	gb|ABPN01001262.1|:12-2327
	NuwaI-1_REso	gb|ABPN01010082.1|:1-1021
	NuwaI-1_REso	gb|ABPN01003344.1|:500-2296
	NuwaI-1_REso	gb|ABPN01000815.1|:594-1606
	NuwaI-1_REso	gb|ABPN01012965.1|:507-1539
	NuwaI-1_REso	gb|ABPN01002161.1|:1673-2847
	NuwaI-1_REso	gb|ABPN01003579.1|:1-1312
	NuwaI-1_REso	gb|ABPN01009551.1|:31-1295
	NuwaI-1_REso	gb|ABPN01001059.1|:1363-3106

NuwaI-1_REso	gb|ABPN01003913.1|:1-981
NuwaI-1_REso	gb|ABPN01000687.1|:189-1497
NuwaI-1_REso	gb|ABPN01001056.1|:532-2326
NuwaI-1_REso	gb|ABPN01000599.1|:2223-3183
NuwaI-1_REso	gb|ABPN01000202.1|:1586-3378
NuwaI-1_REso	gb|ABPN01001906.1|:551-1864
NuwaI-1_REso	gb|ABPN01003830.1|:158-1953
NuwaI-1_REso	gb|ABPN01004115.1|:28-973
NuwaI-1_REso	gb|ABPN01000761.1|:425-1394
NuwaI-1_REso	gb|ABPN01002809.1|:285-2072
NuwaI-1_REso	gb|ABPN01002037.1|:24-1292
NuwaI-1_REso	gb|ABPN01009370.1|:898-1826
NuwaI-1_REso	gb|ABPN01004789.1|:1783-2621
NuwaI-1_REso	gb|ABPN01000232.1|:2126-3729
NuwaI-1_REso	gb|ABPN01010993.1|:9-872
NuwaI-1_REso	gb|ABPN01003373.1|:1812-2555
NuwaI-1_REso	gb|ABPN01042084.1|:66-1014
NuwaI-1_REso	gb|ABPN01012935.1|:727-1506
NuwaI-1_REso	gb|ABPN01000010.1|:1136-2671
NuwaI-1_REso	gb|ABPN01000617.1|:195-915
NuwaI-1_REso	gb|ABPN01041378.1|:485-1142
NuwaI-1_REso	gb|ABPN01005837.1|:1-987
NuwaI-1_REso	gb|ABPN01010846.1|:55-1030
NuwaI-1_REso	gb|ABPN01048636.1|:60-1065
NuwaI-1_REso	gb|ABPN01028564.1|:2-1005
NuwaI-1_REso	gb|ABPN01070770.1|:7-807
NuwaI-1_REso	gb|ABPN01009111.1|:1-897
NuwaI-1_REso	gb|ABPN01036870.1|:153-678
NuwaI-1_REso	gb|ABPN01011095.1|:290-1646
NuwaI-1_REso	gb|ABPN01002410.1|:2082-2621
NuwaI-1_REso	gb|ABPN01045243.1|:40-804
NuwaI-1_REso	gb|ABPN01004726.1|:8-442
NuwaI-1_REso	gb|ABPN01001909.1|:2168-2601
NuwaI-1_REso	gb|ABPN01000339.1|:440-874
NuwaI-1_REso	gb|ABPN01003374.1|:1-427
NuwaI-1_REso	gb|ABPN01010975.1|:101-536
NuwaI-1_REso	gb|ABPN01002038.1|:1-436
NuwaI-1_REso	gb|ABPN01000688.1|:1-435
NuwaI-1_REso	gb|ABPN01001907.1|:1-436
NuwaI-1_REso	gb|ABPN01007992.1|:1-437
NuwaI-1_REso	gb|ABPN01009110.1|:283-717
NuwaI-1_REso	gb|ABPN01005838.1|:1-437
NuwaI-1_REso	gb|ABPN01000037.1|:1-438
NuwaI-1_REso	gb|ABPN01010847.1|:1-437

	NuwaI-1_REso	gb|ABPN01000389.1|:1033-1472
	NuwaI-1_REso	gb|ABPN01002144.1|:167-617
	NuwaI-1_REso	gb|ABPN01007991.1|:90-578
	NuwaI-1_REso	gb|ABPN01007293.1|:1759-2181
	NuwaI-1_REso	gb|ABPN01039301.1|:29-342
	NuwaI-1_REso	gb|ABPN01039515.1|:30-449
	NuwaI-1_REso	gb|ABPN01010081.1|:422-687
	NuwaI-1_REso	gb|ABPN01005142.1|:694-996
	NuwaI-1_REso	gb|ABPN01000618.1|:1-333
	NuwaI-1_REso	gb|ABPN01010229.1|:1058-1317
	NuwaI-1_REso	gb|ABPN01007161.1|:1825-2109
	NuwaI-1_REso	gb|ABPN01010976.1|:1-229
	NuwaI-1_REso	gb|ABPN01010043.1|:1-227
	NuwaI-1_REso	gb|ABPN01000657.1|:2018-2120
	NuwaI-1_REso	gb|ABPN01040216.1|:302-490
	NuwaI-1_REso	gb|ABPN01003430.1|:1119-1214
Sebastes nigrocinctus	*NuwaI-1_SNig*	gb|AUPR01062543.1|:4335-7405
	NuwaI-1_SNig	gb|AUPR01100979.1|:10114-13725
	NuwaI-1_SNig	gb|AUPR01155455.1|:3629-5673
	NuwaI-1_SNig	gb|AUPR01079087.1|:850-2475
	NuwaI-1_SNig	gb|AUPR01002174.1|:8621-10191
	NuwaI-1_SNig	gb|AUPR01101706.1|:2826-5784
	NuwaI-1_SNig	gb|AUPR01079100.1|:3516-5096
	NuwaI-1_SNig	gb|AUPR01010336.1|:16221-17487
	NuwaI-1_SNig	gb|AUPR01031519.1|:987-2572
	NuwaI-1_SNig	gb|AUPR01117727.1|:2491-3708
	NuwaI-1_SNig	gb|AUPR01000013.1|:6131-7166
	NuwaI-1_SNig	gb|AUPR01075943.1|:466-2164
	NuwaI-1_SNig	gb|AUPR01063083.1|:11826-13571
	NuwaI-1_SNig	gb|AUPR01071543.1|:1960-3346
	NuwaI-1_SNig	gb|AUPR01118219.1|:1481-2508
	NuwaI-1_SNig	gb|AUPR01013700.1|:917-1900
	NuwaI-1_SNig	gb|AUPR01125040.1|:9851-11288
	NuwaI-1_SNig	gb|AUPR01056757.1|:2331-2990
	NuwaI-1_SNig	gb|AUPR01053396.1|:11348-12279
	NuwaI-1_SNig	gb|AUPR01047099.1|:8928-10395
	NuwaI-1_SNig	gb|AUPR01118313.1|:4525-5485
	NuwaI-1_SNig	gb|AUPR01136841.1|:531-2636
	NuwaI-1_SNig	gb|AUPR01113178.1|:3854-4995
	NuwaI-1_SNig	gb|AUPR01081865.1|:17168-17769
	NuwaI-1_SNig	gb|AUPR01098778.1|:866-1448
	NuwaI-1_SNig	gb|AUPR01045191.1|:1-720
	NuwaI-1_SNig	gb|AUPR01084267.1|:937-1534
	NuwaI-1_SNig	gb|AUPR01087080.1|:904-2257

NuwaI-1_SNig	gb|AUPR01146956.1|:588-1594
NuwaI-1_SNig	gb|AUPR01045193.1|:4697-5529
NuwaI-1_SNig	gb|AUPR01037647.1|:1606-2102
NuwaI-1_SNig	gb|AUPR01112428.1|:403-950
NuwaI-1_SNig	gb|AUPR01067753.1|:7040-7623
NuwaI-1_SNig	gb|AUPR01079359.1|:35-1514
NuwaI-1_SNig	gb|AUPR01061329.1|:1496-1911
NuwaI-1_SNig	gb|AUPR01095755.1|:8112-8823
NuwaI-1_SNig	gb|AUPR01099084.1|:33-460
NuwaI-1_SNig	gb|AUPR01057978.1|:750-1118
NuwaI-1_SNig	gb|AUPR01020037.1|:70-497
NuwaI-1_SNig	gb|AUPR01074597.1|:96-939
NuwaI-1_SNig	gb|AUPR01107102.1|:1102-1511
NuwaI-1_SNig	gb|AUPR01019857.1|:5027-5477
NuwaI-1_SNig	gb|AUPR01046793.1|:8467-10531
NuwaI-1_SNig	gb|AUPR01053324.1|:14836-16224
NuwaI-1_SNig	gb|AUPR01000645.1|:2205-2574
NuwaI-1_SNig	gb|AUPR01086502.1|:4358-5082
NuwaI-1_SNig	gb|AUPR01027439.1|:6575-7431
NuwaI-1_SNig	gb|AUPR01001651.1|:2553-3073
NuwaI-1_SNig	gb|AUPR01046855.1|:868-1243
NuwaI-1_SNig	gb|AUPR01121230.1|:1-320
NuwaI-1_SNig	gb|AUPR01067741.1|:2364-3093
NuwaI-1_SNig	gb|AUPR01097937.1|:590-1760
NuwaI-1_SNig	gb|AUPR01057426.1|:23109-23414
NuwaI-1_SNig	gb|AUPR01029943.1|:11728-12475
NuwaI-1_SNig	gb|AUPR01077095.1|:1852-2212
NuwaI-1_SNig	gb|AUPR01008762.1|:5188-5521
NuwaI-1_SNig	gb|AUPR01041617.1|:56-324
NuwaI-1_SNig	gb|AUPR01118220.1|:135-583
NuwaI-1_SNig	gb|AUPR01041618.1|:1-292
NuwaI-1_SNig	gb|AUPR01080413.1|:10061-10714
NuwaI-1_SNig	gb|AUPR01149736.1|:112-394
NuwaI-1_SNig	gb|AUPR01031710.1|:9975-10252
NuwaI-1_SNig	gb|AUPR01108510.1|:117-389
NuwaI-1_SNig	gb|AUPR01006434.1|:177-430
NuwaI-1_SNig	gb|AUPR01077633.1|:10-263
NuwaI-1_SNig	gb|AUPR01066606.1|:5-195
NuwaI-1_SNig	gb|AUPR01140033.1|:1-255
NuwaI-1_SNig	gb|AUPR01041619.1|:167-758
NuwaI-1_SNig	gb|AUPR01022002.1|:1-255
NuwaI-1_SNig	gb|AUPR01093379.1|:156-546
NuwaI-1_SNig	gb|AUPR01099716.1|:486-742
NuwaI-1_SNig	gb|AUPR01083518.1|:813-1054

NuwaI-1_SNig	gb|AUPR01004357.1|:17092-17319
NuwaI-1_SNig	gb|AUPR01022001.1|:1065-1295
NuwaI-1_SNig	gb|AUPR01017643.1|:12370-12648
NuwaI-1_SNig	gb|AUPR01070340.1|:1-223
NuwaI-1_SNig	gb|AUPR01157819.1|:2213-3013
NuwaI-1_SNig	gb|AUPR01059160.1|:4422-4639
NuwaI-1_SNig	gb|AUPR01143299.1|:2-229
NuwaI-1_SNig	gb|AUPR01045190.1|:2093-2305
NuwaI-1_SNig	gb|AUPR01058032.1|:1-197
NuwaI-1_SNig	gb|AUPR01066597.1|:5030-5422
NuwaI-1_SNig	gb|AUPR01120786.1|:2786-3217
NuwaI-1_SNig	gb|AUPR01041620.1|:11-249
NuwaI-1_SNig	gb|AUPR01066608.1|:1-537
NuwaI-1_SNig	gb|AUPR01066607.1|:2-197
NuwaI-1_SNig	gb|AUPR01159028.1|:93-424
NuwaI-1_SNig	gb|AUPR01092818.1|:152-436
NuwaI-1_SNig	gb|AUPR01181954.1|:125-323
NuwaI-1_SNig	gb|AUPR01078845.1|:5373-5560
NuwaI-1_SNig	gb|AUPR01107103.1|:13-169
NuwaI-1_SNig	gb|AUPR01056126.1|:17-109
NuwaI-1_SNig	gb|AUPR01056127.1|:4-165
NuwaI-1_SNig	gb|AUPR01091987.1|:1597-1747
NuwaI-1_SNig	gb|AUPR01005053.1|:3602-3764
NuwaI-1_SNig	gb|AUPR01128148.1|:361-509
NuwaI-1_SNig	gb|AUPR01075942.1|:2939-3086
NuwaI-1_SNig	gb|AUPR01139140.1|:31-171
NuwaI-1_SNig	gb|AUPR01118961.1|:150-293
NuwaI-1_SNig	gb|AUPR01020204.1|:4598-4762
NuwaI-1_SNig	gb|AUPR01122194.1|:756-887
NuwaI-1_SNig	gb|AUPR01192729.1|:1138-1269
NuwaI-1_SNig	gb|AUPR01048831.1|:266-507
NuwaI-1_SNig	gb|AUPR01127001.1|:7077-7198
NuwaI-1_SNig	gb|AUPR01195481.1|:2578-2699
NuwaI-1_SNig	gb|AUPR01090659.1|:1-135
NuwaI-1_SNig	gb|AUPR01066605.1|:1-108
NuwaI-1_SNig	gb|AUPR01015260.1|:19205-19313
NuwaI-1_SNig	gb|AUPR01050012.1|:9236-9342
NuwaI-1_SNig	gb|AUPR01008601.1|:12587-12684
NuwaI-1_SNig	gb|AUPR01144142.1|:689-783
NuwaI-1_SNig	gb|AUPR01000992.1|:6546-6640
NuwaI-1_SNig	gb|AUPR01046080.1|:6880-6973
NuwaI-1_SNig	gb|AUPR01149117.1|:9-92
NuwaI-1_SNig	gb|AUPR01194829.1|:1411-1490
NuwaI-1_SNig	gb|AUPR01153784.1|:47-122

NuwaI-1_SNig	gb|AUPR01112293.1|:1-71
NuwaI-1_SNig	gb|AUPR01116430.1|:6913-6963
NuwaI-1_SNig	gb|AUPR01045978.1|:11106-14401
NuwaI-1_SNig	gb|AUPR01145213.1|:7440-10580
NuwaI-1_SNig	gb|AUPR01080035.1|:4780-7230
NuwaI-1_SNig	gb|AUPR01161625.1|:1306-4181
NuwaI-1_SNig	gb|AUPR01146731.1|:10175-11573
NuwaI-1_SNig	gb|AUPR01045168.1|:8046-9574
NuwaI-1_SNig	gb|AUPR01034536.1|:9113-10613
NuwaI-1_SNig	gb|AUPR01036341.1|:11435-13025
NuwaI-1_SNig	gb|AUPR01081810.1|:1827-3386
NuwaI-1_SNig	gb|AUPR01119914.1|:4216-5802
NuwaI-1_SNig	gb|AUPR01070452.1|:14132-16257
NuwaI-1_SNig	gb|AUPR01088986.1|:5597-6571
NuwaI-1_SNig	gb|AUPR01011974.1|:1469-2756
NuwaI-1_SNig	gb|AUPR01046038.1|:4061-4957
NuwaI-1_SNig	gb|AUPR01049931.1|:3496-4718
NuwaI-1_SNig	gb|AUPR01017394.1|:2265-3366
NuwaI-1_SNig	gb|AUPR01182454.1|:4738-5780
NuwaI-1_SNig	gb|AUPR01076492.1|:241-860
NuwaI-1_SNig	gb|AUPR01010855.1|:274-951
NuwaI-1_SNig	gb|AUPR01117507.1|:1199-2538
NuwaI-1_SNig	gb|AUPR01089383.1|:71-730
NuwaI-1_SNig	gb|AUPR01044239.1|:329-956
NuwaI-1_SNig	gb|AUPR01091440.1|:196-770
NuwaI-1_SNig	gb|AUPR01041832.1|:948-3182
NuwaI-1_SNig	gb|AUPR01064422.1|:4463-5713
NuwaI-1_SNig	gb|AUPR01168598.1|:118-670
NuwaI-1_SNig	gb|AUPR01097330.1|:1-514
NuwaI-1_SNig	gb|AUPR01167475.1|:9-516
NuwaI-1_SNig	gb|AUPR01128174.1|:304-855
NuwaI-1_SNig	gb|AUPR01027439.1|:2127-3163
NuwaI-1_SNig	gb|AUPR01107001.1|:4-643
NuwaI-1_SNig	gb|AUPR01001145.1|:1-453
NuwaI-1_SNig	gb|AUPR01077853.1|:4207-4643
NuwaI-1_SNig	gb|AUPR01047209.1|:8-418
NuwaI-1_SNig	gb|AUPR01087548.1|:2199-2864
NuwaI-1_SNig	gb|AUPR01001095.1|:833-1273
NuwaI-1_SNig	gb|AUPR01011681.1|:4460-4969
NuwaI-1_SNig	gb|AUPR01070815.1|:3381-4039
NuwaI-1_SNig	gb|AUPR01025969.1|:2347-3415
NuwaI-1_SNig	gb|AUPR01084889.1|:70-470
NuwaI-1_SNig	gb|AUPR01120103.1|:20-363
NuwaI-1_SNig	gb|AUPR01119846.1|:657-1230

NuwaI-1_SNig	gb|AUPR01059777.1|:128-857
NuwaI-1_SNig	gb|AUPR01039348.1|:2824-3167
NuwaI-1_SNig	gb|AUPR01155455.1|:5732-6338
NuwaI-1_SNig	gb|AUPR01019624.1|:5-358
NuwaI-1_SNig	gb|AUPR01122757.1|:5677-6675
NuwaI-1_SNig	gb|AUPR01049024.1|:15589-15943
NuwaI-1_SNig	gb|AUPR01082773.1|:12018-12402
NuwaI-1_SNig	gb|AUPR01103090.1|:1350-2101
NuwaI-1_SNig	gb|AUPR01116430.1|:6692-7060
NuwaI-1_SNig	gb|AUPR01030020.1|:257-928
NuwaI-1_SNig	gb|AUPR01037471.1|:4-388
NuwaI-1_SNig	gb|AUPR01151628.1|:1-334
NuwaI-1_SNig	gb|AUPR01021314.1|:2872-3277
NuwaI-1_SNig	gb|AUPR01059136.1|:150-455
NuwaI-1_SNig	gb|AUPR01144142.1|:186-860
NuwaI-1_SNig	gb|AUPR01097786.1|:101-447
NuwaI-1_SNig	gb|AUPR01119158.1|:344-910
NuwaI-1_SNig	gb|AUPR01131622.1|:446-790
NuwaI-1_SNig	gb|AUPR01118160.1|:3071-4431
NuwaI-1_SNig	gb|AUPR01001198.1|:2294-4498
NuwaI-1_SNig	gb|AUPR01148361.1|:694-1283
NuwaI-1_SNig	gb|AUPR01157819.1|:1581-1898
NuwaI-1_SNig	gb|AUPR01021281.1|:2-246
NuwaI-1_SNig	gb|AUPR01050728.1|:2561-2842
NuwaI-1_SNig	gb|AUPR01085661.1|:84-359
NuwaI-1_SNig	gb|AUPR01169752.1|:1-326
NuwaI-1_SNig	gb|AUPR01024069.1|:16946-17234
NuwaI-1_SNig	gb|AUPR01044163.1|:1060-1345
NuwaI-1_SNig	gb|AUPR01089381.1|:3119-3392
NuwaI-1_SNig	gb|AUPR01046889.1|:1135-1471
NuwaI-1_SNig	gb|AUPR01143258.1|:1-274
NuwaI-1_SNig	gb|AUPR01072836.1|:590-972
NuwaI-1_SNig	gb|AUPR01073457.1|:3721-4143
NuwaI-1_SNig	gb|AUPR01081621.1|:2237-2477
NuwaI-1_SNig	gb|AUPR01037007.1|:61-323
NuwaI-1_SNig	gb|AUPR01165246.1|:176-445
NuwaI-1_SNig	gb|AUPR01103091.1|:362-632
NuwaI-1_SNig	gb|AUPR01062260.1|:3119-3345
NuwaI-1_SNig	gb|AUPR01124079.1|:2318-2485
NuwaI-1_SNig	gb|AUPR01083904.1|:1-231
NuwaI-1_SNig	gb|AUPR01116044.1|:214-553
NuwaI-1_SNig	gb|AUPR01001094.1|:927-1159
NuwaI-1_SNig	gb|AUPR01089205.1|:1640-1859
NuwaI-1_SNig	gb|AUPR01049234.1|:2165-2489

NuwaI-1_SNig	gb|AUPR01002736.1|:6336-6552
NuwaI-1_SNig	gb|AUPR01091439.1|:681-891
NuwaI-1_SNig	gb|AUPR01125279.1|:155-442
NuwaI-1_SNig	gb|AUPR01179364.1|:343-732
NuwaI-1_SNig	gb|AUPR01033833.1|:1-216
NuwaI-1_SNig	gb|AUPR01151145.1|:5-155
NuwaI-1_SNig	gb|AUPR01045193.1|:5749-5976
NuwaI-1_SNig	gb|AUPR01103266.1|:1887-2113
NuwaI-1_SNig	gb|AUPR01117122.1|:324-547
NuwaI-1_SNig	gb|AUPR01050729.1|:4-198
NuwaI-1_SNig	gb|AUPR01002171.1|:3379-3572
NuwaI-1_SNig	gb|AUPR01080036.1|:1-184
NuwaI-1_SNig	gb|AUPR01179117.1|:311-560
NuwaI-1_SNig	gb|AUPR01058033.1|:13858-14294
NuwaI-1_SNig	gb|AUPR01021282.1|:1-211
NuwaI-1_SNig	gb|AUPR01119159.1|:1-223
NuwaI-1_SNig	gb|AUPR01068452.1|:3982-4181
NuwaI-1_SNig	gb|AUPR01147839.1|:1134-1334
NuwaI-1_SNig	gb|AUPR01040746.1|:283-511
NuwaI-1_SNig	gb|AUPR01119245.1|:58-231
NuwaI-1_SNig	gb|AUPR01056546.1|:2593-2764
NuwaI-1_SNig	gb|AUPR01053524.1|:267-439
NuwaI-1_SNig	gb|AUPR01120102.1|:260-422
NuwaI-1_SNig	gb|AUPR01151146.1|:10-166
NuwaI-1_SNig	gb|AUPR01123394.1|:36-192
NuwaI-1_SNig	gb|AUPR01155641.1|:40-196
NuwaI-1_SNig	gb|AUPR01114329.1|:10-166
NuwaI-1_SNig	gb|AUPR01052821.1|:1805-1954
NuwaI-1_SNig	gb|AUPR01155640.1|:2-172
NuwaI-1_SNig	gb|AUPR01181309.1|:43-214
NuwaI-1_SNig	gb|AUPR01110632.1|:1205-1454
NuwaI-1_SNig	gb|AUPR01181827.1|:1152-1298
NuwaI-1_SNig	gb|AUPR01074055.1|:5137-5306
NuwaI-1_SNig	gb|AUPR01091779.1|:3207-3344
NuwaI-1_SNig	gb|AUPR01132183.1|:3-140
NuwaI-1_SNig	gb|AUPR01131306.1|:1-535
NuwaI-1_SNig	gb|AUPR01051783.1|:1098-1241
NuwaI-1_SNig	gb|AUPR01047208.1|:35-159
NuwaI-1_SNig	gb|AUPR01152503.1|:8425-8604
NuwaI-1_SNig	gb|AUPR01047849.1|:431-550
NuwaI-1_SNig	gb|AUPR01011722.1|:8-121
NuwaI-1_SNig	gb|AUPR01128908.1|:312-422
NuwaI-1_SNig	gb|AUPR01164107.1|:830-939
NuwaI-1_SNig	gb|AUPR01098561.1|:4274-4381

	NuwaI-1_SNig	gb|AUPR01085978.1|:1714-1820
	NuwaI-1_SNig	gb|AUPR01097788.1|:83-184
	NuwaI-1_SNig	gb|AUPR01191662.1|:2205-2303
	NuwaI-1_SNig	gb|AUPR01187714.1|:1-82
	NuwaI-1_SNig	gb|AUPR01060603.1|:1919-1994
	NuwaI-1_SNig	gb|AUPR01119244.1|:81-155
	NuwaI-1_SNig	gb|AUPR01126043.1|:333-407
	NuwaI-1_SNig	gb|AUPR01076541.1|:798-870
	NuwaI-1_SNig	gb|AUPR01098778.1|:1077-1131
	NuwaI-1_SNig	gb|AUPR01062543.1|:4455-4505
	NuwaI-1_SNig	gb|AUPR01000013.1|:6340-6388
Sebastes rubrivinctus	*NuwaI-1_SRub*	gb|AUPQ01109926.1|:5874-9112
	NuwaI-1_SRub	gb|AUPQ01078678.1|:9-2680
	NuwaI-1_SRub	gb|AUPQ01059709.1|:106-1566
	NuwaI-1_SRub	gb|AUPQ01057975.1|:4622-6672
	NuwaI-1_SRub	gb|AUPQ01060170.1|:6012-7527
	NuwaI-1_SRub	gb|AUPQ01042300.1|:206-2654
	NuwaI-1_SRub	gb|AUPQ01096510.1|:1595-2953
	NuwaI-1_SRub	gb|AUPQ01034443.1|:14105-15540
	NuwaI-1_SRub	gb|AUPQ01057820.1|:7800-9307
	NuwaI-1_SRub	gb|AUPQ01020323.1|:10860-12445
	NuwaI-1_SRub	gb|AUPQ01025820.1|:83-1816
	NuwaI-1_SRub	gb|AUPQ01036839.1|:4062-5540
	NuwaI-1_SRub	gb|AUPQ01019901.1|:4761-5801
	NuwaI-1_SRub	gb|AUPQ01041344.1|:15-1488
	NuwaI-1_SRub	gb|AUPQ01072975.1|:850-1723
	NuwaI-1_SRub	gb|AUPQ01043118.1|:3261-4547
	NuwaI-1_SRub	gb|AUPQ01079859.1|:4500-6172
	NuwaI-1_SRub	gb|AUPQ01038221.1|:4180-5344
	NuwaI-1_SRub	gb|AUPQ01029039.1|:10435-11872
	NuwaI-1_SRub	gb|AUPQ01056162.1|:3764-5066
	NuwaI-1_SRub	gb|AUPQ01078202.1|:305-2253
	NuwaI-1_SRub	gb|AUPQ01071833.1|:1-587
	NuwaI-1_SRub	gb|AUPQ01109866.1|:2421-3104
	NuwaI-1_SRub	gb|AUPQ01110874.1|:838-2185
	NuwaI-1_SRub	gb|AUPQ01067228.1|:12577-13583
	NuwaI-1_SRub	gb|AUPQ01104432.1|:239-860
	NuwaI-1_SRub	gb|AUPQ01083142.1|:845-1427
	NuwaI-1_SRub	gb|AUPQ01036236.1|:6443-7301
	NuwaI-1_SRub	gb|AUPQ01018062.1|:1-1115
	NuwaI-1_SRub	gb|AUPQ01015637.1|:6190-7246
	NuwaI-1_SRub	gb|AUPQ01061474.1|:1877-2978
	NuwaI-1_SRub	gb|AUPQ01035733.1|:1204-1823
	NuwaI-1_SRub	gb|AUPQ01105141.1|:3924-4468

NuwaI-1_SRub	gb|AUPQ01030134.1|:387-935
NuwaI-1_SRub	gb|AUPQ01020829.1|:5882-6407
NuwaI-1_SRub	gb|AUPQ01109661.1|:565-1109
NuwaI-1_SRub	gb|AUPQ01129971.1|:2935-3425
NuwaI-1_SRub	gb|AUPQ01054784.1|:185-778
NuwaI-1_SRub	gb|AUPQ01114059.1|:619-1170
NuwaI-1_SRub	gb|AUPQ01017230.1|:56-6561
NuwaI-1_SRub	gb|AUPQ01110105.1|:85-554
NuwaI-1_SRub	gb|AUPQ01014877.1|:3928-5069
NuwaI-1_SRub	gb|AUPQ01069046.1|:56228-56640
NuwaI-1_SRub	gb|AUPQ01100295.1|:135-1179
NuwaI-1_SRub	gb|AUPQ01014788.1|:2924-3990
NuwaI-1_SRub	gb|AUPQ01003719.1|:3405-3777
NuwaI-1_SRub	gb|AUPQ01097374.1|:1247-1952
NuwaI-1_SRub	gb|AUPQ01096656.1|:88-469
NuwaI-1_SRub	gb|AUPQ01077154.1|:4755-5797
NuwaI-1_SRub	gb|AUPQ01116813.1|:580-940
NuwaI-1_SRub	gb|AUPQ01059050.1|:962-1337
NuwaI-1_SRub	gb|AUPQ01085756.1|:1-361
NuwaI-1_SRub	gb|AUPQ01012369.1|:1918-2438
NuwaI-1_SRub	gb|AUPQ01054785.1|:132-860
NuwaI-1_SRub	gb|AUPQ01064591.1|:1-844
NuwaI-1_SRub	gb|AUPQ01011837.1|:774-1273
NuwaI-1_SRub	gb|AUPQ01094413.1|:1-346
NuwaI-1_SRub	gb|AUPQ01060727.1|:6376-6720
NuwaI-1_SRub	gb|AUPQ01066978.1|:8301-8602
NuwaI-1_SRub	gb|AUPQ01076809.1|:415-709
NuwaI-1_SRub	gb|AUPQ01066979.1|:4-331
NuwaI-1_SRub	gb|AUPQ01044838.1|:686-967
NuwaI-1_SRub	gb|AUPQ01046847.1|:2028-2324
NuwaI-1_SRub	gb|AUPQ01045804.1|:791-1060
NuwaI-1_SRub	gb|AUPQ01091344.1|:119-373
NuwaI-1_SRub	gb|AUPQ01124147.1|:11-264
NuwaI-1_SRub	gb|AUPQ01086081.1|:1266-1505
NuwaI-1_SRub	gb|AUPQ01024983.1|:5428-6340
NuwaI-1_SRub	gb|AUPQ01059382.1|:1-248
NuwaI-1_SRub	gb|AUPQ01003077.1|:1995-2222
NuwaI-1_SRub	gb|AUPQ01041418.1|:24538-24779
NuwaI-1_SRub	gb|AUPQ01084814.1|:12789-13061
NuwaI-1_SRub	gb|AUPQ01105670.1|:1700-2485
NuwaI-1_SRub	gb|AUPQ01079341.1|:72-324
NuwaI-1_SRub	gb|AUPQ01045405.1|:17344-17771
NuwaI-1_SRub	gb|AUPQ01098817.1|:1216-1439
NuwaI-1_SRub	gb|AUPQ01077432.1|:437-648

NuwaI-1_SRub	gb|AUPQ01110106.1|:9-587
NuwaI-1_SRub	gb|AUPQ01059381.1|:21379-21576
NuwaI-1_SRub	gb|AUPQ01074101.1|:103-293
NuwaI-1_SRub	gb|AUPQ01041343.1|:312-503
NuwaI-1_SRub	gb|AUPQ01097333.1|:25-216
NuwaI-1_SRub	gb|AUPQ01122969.1|:175-611
NuwaI-1_SRub	gb|AUPQ01023640.1|:1-191
NuwaI-1_SRub	gb|AUPQ01085755.1|:1740-1928
NuwaI-1_SRub	gb|AUPQ01071026.1|:5011-5196
NuwaI-1_SRub	gb|AUPQ01017178.1|:831-1011
NuwaI-1_SRub	gb|AUPQ01026300.1|:171-350
NuwaI-1_SRub	gb|AUPQ01080377.1|:2649-2821
NuwaI-1_SRub	gb|AUPQ01071816.1|:1-169
NuwaI-1_SRub	gb|AUPQ01008294.1|:49342-49629
NuwaI-1_SRub	gb|AUPQ01101703.1|:1-163
NuwaI-1_SRub	gb|AUPQ01005080.1|:4384-4550
NuwaI-1_SRub	gb|AUPQ01003720.1|:57-213
NuwaI-1_SRub	gb|AUPQ01053141.1|:2526-2675
NuwaI-1_SRub	gb|AUPQ01043463.1|:120-270
NuwaI-1_SRub	gb|AUPQ01079173.1|:3383-3545
NuwaI-1_SRub	gb|AUPQ01053367.1|:7960-8101
NuwaI-1_SRub	gb|AUPQ01031571.1|:64-206
NuwaI-1_SRub	gb|AUPQ01127120.1|:94-231
NuwaI-1_SRub	gb|AUPQ01097332.1|:228-359
NuwaI-1_SRub	gb|AUPQ01135686.1|:67-198
NuwaI-1_SRub	gb|AUPQ01086192.1|:266-507
NuwaI-1_SRub	gb|AUPQ01118413.1|:14-158
NuwaI-1_SRub	gb|AUPQ01102813.1|:1-189
NuwaI-1_SRub	gb|AUPQ01104274.1|:926-1050
NuwaI-1_SRub	gb|AUPQ01095719.1|:3019-3141
NuwaI-1_SRub	gb|AUPQ01022524.1|:6666-6787
NuwaI-1_SRub	gb|AUPQ01061419.1|:772-888
NuwaI-1_SRub	gb|AUPQ01029011.1|:5268-5387
NuwaI-1_SRub	gb|AUPQ01038222.1|:1-114
NuwaI-1_SRub	gb|AUPQ01091343.1|:6-287
NuwaI-1_SRub	gb|AUPQ01014847.1|:15428-15528
NuwaI-1_SRub	gb|AUPQ01062220.1|:1-154
NuwaI-1_SRub	gb|AUPQ01064221.1|:397-496
NuwaI-1_SRub	gb|AUPQ01091881.1|:9813-9911
NuwaI-1_SRub	gb|AUPQ01134598.1|:6538-6631
NuwaI-1_SRub	gb|AUPQ01059778.1|:477-570
NuwaI-1_SRub	gb|AUPQ01004647.1|:311-399
NuwaI-1_SRub	gb|AUPQ01039098.1|:6223-6299
NuwaI-1_SRub	gb|AUPQ01132776.1|:102-174

NuwaI-1_SRub	gb|AUPQ01106948.1|:4-76
NuwaI-1_SRub	gb|AUPQ01028427.1|:10113-10183
NuwaI-1_SRub	gb|AUPQ01067143.1|:11174-11243
NuwaI-1_SRub	gb|AUPQ01125114.1|:255-305
NuwaI-1_SRub	gb|AUPQ01030667.1|:1046-1096
NuwaI-1_SRub	gb|AUPQ01066774.1|:11089-14221
NuwaI-1_SRub	gb|AUPQ01125815.1|:698-3571
NuwaI-1_SRub	gb|AUPQ01026464.1|:30026-33637
NuwaI-1_SRub	gb|AUPQ01098321.1|:10317-11741
NuwaI-1_SRub	gb|AUPQ01051838.1|:10627-12055
NuwaI-1_SRub	gb|AUPQ01007125.1|:8138-9628
NuwaI-1_SRub	gb|AUPQ01026032.1|:23260-24821
NuwaI-1_SRub	gb|AUPQ01079342.1|:449-2003
NuwaI-1_SRub	gb|AUPQ01080041.1|:3963-5665
NuwaI-1_SRub	gb|AUPQ01085488.1|:642-2438
NuwaI-1_SRub	gb|AUPQ01060523.1|:5913-7188
NuwaI-1_SRub	gb|AUPQ01081014.1|:15880-17995
NuwaI-1_SRub	gb|AUPQ01012811.1|:2462-3847
NuwaI-1_SRub	gb|AUPQ01016648.1|:5593-6462
NuwaI-1_SRub	gb|AUPQ01026982.1|:17597-18623
NuwaI-1_SRub	gb|AUPQ01032623.1|:1-881
NuwaI-1_SRub	gb|AUPQ01103283.1|:1-1260
NuwaI-1_SRub	gb|AUPQ01070373.1|:977-1636
NuwaI-1_SRub	gb|AUPQ01041702.1|:19061-20024
NuwaI-1_SRub	gb|AUPQ01059709.1|:1573-2969
NuwaI-1_SRub	gb|AUPQ01023640.1|:411-1243
NuwaI-1_SRub	gb|AUPQ01075625.1|:5992-7156
NuwaI-1_SRub	gb|AUPQ01110043.1|:385-1289
NuwaI-1_SRub	gb|AUPQ01013304.1|:301-1384
NuwaI-1_SRub	gb|AUPQ01003733.1|:298-1001
NuwaI-1_SRub	gb|AUPQ01060111.1|:181-792
NuwaI-1_SRub	gb|AUPQ01052047.1|:1641-2891
NuwaI-1_SRub	gb|AUPQ01032997.1|:8398-8999
NuwaI-1_SRub	gb|AUPQ01122498.1|:34-699
NuwaI-1_SRub	gb|AUPQ01045760.1|:1-543
NuwaI-1_SRub	gb|AUPQ01070274.1|:34-520
NuwaI-1_SRub	gb|AUPQ01077987.1|:394-1141
NuwaI-1_SRub	gb|AUPQ01009179.1|:4849-5358
NuwaI-1_SRub	gb|AUPQ01125114.1|:1-425
NuwaI-1_SRub	gb|AUPQ01001013.1|:11372-11955
NuwaI-1_SRub	gb|AUPQ01052212.1|:1143-1852
NuwaI-1_SRub	gb|AUPQ01021978.1|:1228-1685
NuwaI-1_SRub	gb|AUPQ01043259.1|:1588-2025
NuwaI-1_SRub	gb|AUPQ01070372.1|:6093-6493

NuwaI-1_SRub	gb|AUPQ01102956.1|:4-390
NuwaI-1_SRub	gb|AUPQ01112019.1|:136-624
NuwaI-1_SRub	gb|AUPQ01126200.1|:55-440
NuwaI-1_SRub	gb|AUPQ01013305.1|:146-504
NuwaI-1_SRub	gb|AUPQ01019935.1|:12623-13352
NuwaI-1_SRub	gb|AUPQ01107804.1|:74-976
NuwaI-1_SRub	gb|AUPQ01033912.1|:1-334
NuwaI-1_SRub	gb|AUPQ01009724.1|:2175-2527
NuwaI-1_SRub	gb|AUPQ01018554.1|:438-793
NuwaI-1_SRub	gb|AUPQ01058725.1|:2201-2533
NuwaI-1_SRub	gb|AUPQ01027692.1|:12-333
NuwaI-1_SRub	gb|AUPQ01123680.1|:207-554
NuwaI-1_SRub	gb|AUPQ01022713.1|:1450-2201
NuwaI-1_SRub	gb|AUPQ01057820.1|:2226-4430
NuwaI-1_SRub	gb|AUPQ01105670.1|:1058-1376
NuwaI-1_SRub	gb|AUPQ01018875.1|:2822-3166
NuwaI-1_SRub	gb|AUPQ01047615.1|:1-282
NuwaI-1_SRub	gb|AUPQ01014978.1|:3399-4146
NuwaI-1_SRub	gb|AUPQ01041772.1|:10293-11015
NuwaI-1_SRub	gb|AUPQ01047249.1|:4482-4851
NuwaI-1_SRub	gb|AUPQ01030667.1|:825-1193
NuwaI-1_SRub	gb|AUPQ01086460.1|:3496-3795
NuwaI-1_SRub	gb|AUPQ01087941.1|:7239-7650
NuwaI-1_SRub	gb|AUPQ01032144.1|:3077-3346
NuwaI-1_SRub	gb|AUPQ01060110.1|:488-760
NuwaI-1_SRub	gb|AUPQ01055601.1|:5895-6172
NuwaI-1_SRub	gb|AUPQ01094084.1|:388-955
NuwaI-1_SRub	gb|AUPQ01096042.1|:2136-2409
NuwaI-1_SRub	gb|AUPQ01081306.1|:2680-2933
NuwaI-1_SRub	gb|AUPQ01066646.1|:849-1102
NuwaI-1_SRub	gb|AUPQ01070272.1|:399-652
NuwaI-1_SRub	gb|AUPQ01104818.1|:723-976
NuwaI-1_SRub	gb|AUPQ01133438.1|:1-497
NuwaI-1_SRub	gb|AUPQ01022714.1|:429-699
NuwaI-1_SRub	gb|AUPQ01094083.1|:608-859
NuwaI-1_SRub	gb|AUPQ01084384.1|:2682-2935
NuwaI-1_SRub	gb|AUPQ01115425.1|:726-980
NuwaI-1_SRub	gb|AUPQ01125275.1|:664-946
NuwaI-1_SRub	gb|AUPQ01021634.1|:3611-3863
NuwaI-1_SRub	gb|AUPQ01069341.1|:41049-41488
NuwaI-1_SRub	gb|AUPQ01045414.1|:904-1185
NuwaI-1_SRub	gb|AUPQ01036231.1|:1-227
NuwaI-1_SRub	gb|AUPQ01087420.1|:688-1029
NuwaI-1_SRub	gb|AUPQ01018552.1|:5848-6080

NuwaI-1_SRub	gb|AUPQ01032621.1|:2520-2740
NuwaI-1_SRub	gb|AUPQ01002464.1|:5463-5679
NuwaI-1_SRub	gb|AUPQ01090411.1|:2749-2964
NuwaI-1_SRub	gb|AUPQ01069807.1|:471-708
NuwaI-1_SRub	gb|AUPQ01130804.1|:102-304
NuwaI-1_SRub	gb|AUPQ01102955.1|:1-226
NuwaI-1_SRub	gb|AUPQ01085384.1|:1480-1754
NuwaI-1_SRub	gb|AUPQ01039098.1|:5736-6128
NuwaI-1_SRub	gb|AUPQ01096352.1|:292-520
NuwaI-1_SRub	gb|AUPQ01041164.1|:3270-3463
NuwaI-1_SRub	gb|AUPQ01050775.1|:1-205
NuwaI-1_SRub	gb|AUPQ01103282.1|:172-359
NuwaI-1_SRub	gb|AUPQ01050892.1|:9-211
NuwaI-1_SRub	gb|AUPQ01050893.1|:42-231
NuwaI-1_SRub	gb|AUPQ01015637.1|:1853-2077
NuwaI-1_SRub	gb|AUPQ01038856.1|:16184-16382
NuwaI-1_SRub	gb|AUPQ01094811.1|:17564-17734
NuwaI-1_SRub	gb|AUPQ01052214.1|:2498-2660
NuwaI-1_SRub	gb|AUPQ01093759.1|:409-581
NuwaI-1_SRub	gb|AUPQ01053983.1|:6201-6479
NuwaI-1_SRub	gb|AUPQ01033913.1|:5-161
NuwaI-1_SRub	gb|AUPQ01094222.1|:2116-2272
NuwaI-1_SRub	gb|AUPQ01012798.1|:18802-18954
NuwaI-1_SRub	gb|AUPQ01085491.1|:798-945
NuwaI-1_SRub	gb|AUPQ01001437.1|:7123-7269
NuwaI-1_SRub	gb|AUPQ01061904.1|:74-220
NuwaI-1_SRub	gb|AUPQ01112068.1|:119-257
NuwaI-1_SRub	gb|AUPQ01109127.1|:514-653
NuwaI-1_SRub	gb|AUPQ01112069.1|:36-169
NuwaI-1_SRub	gb|AUPQ01034191.1|:3308-3439
NuwaI-1_SRub	gb|AUPQ01060395.1|:4530-4670
NuwaI-1_SRub	gb|AUPQ01070640.1|:1-120
NuwaI-1_SRub	gb|AUPQ01017875.1|:3006-3125
NuwaI-1_SRub	gb|AUPQ01027693.1|:6-121
NuwaI-1_SRub	gb|AUPQ01067537.1|:1058-1164
NuwaI-1_SRub	gb|AUPQ01017264.1|:33-138
NuwaI-1_SRub	gb|AUPQ01089951.1|:1-131
NuwaI-1_SRub	gb|AUPQ01025030.1|:231-331
NuwaI-1_SRub	gb|AUPQ01017265.1|:40-138
NuwaI-1_SRub	gb|AUPQ01013485.1|:11743-11840
NuwaI-1_SRub	gb|AUPQ01102583.1|:3811-3908
NuwaI-1_SRub	gb|AUPQ01006280.1|:2681-2775
NuwaI-1_SRub	gb|AUPQ01091140.1|:1-88
NuwaI-1_SRub	gb|AUPQ01127330.1|:156-242

	NuwaI-1_SRub	gb|AUPQ01070273.1|:56-136
	NuwaI-1_SRub	gb|AUPQ01093145.1|:7433-7508
	NuwaI-1_SRub	gb|AUPQ01064929.1|:5676-5751
	NuwaI-1_SRub	gb|AUPQ01112481.1|:4269-4338
	NuwaI-1_SRub	gb|AUPQ01025744.1|:7281-7334
Takifugu flavidus	*NuwaI-1_TFla*	gb|AOOT01010657.1|:5219-8060
	NuwaI-1_TFla	gb|AOOT01020657.1|:6783-9068
	NuwaI-1_TFla	gb|AOOT01120744.1|:982-2580
	NuwaI-1_TFla	gb|AOOT01109459.1|:1-1392
	NuwaI-1_TFla	gb|AOOT01069121.1|:1-1164
	NuwaI-1_TFla	gb|AOOT01105930.1|:1-1010
	NuwaI-1_TFla	gb|AOOT01077494.1|:259-1136
	NuwaI-1_TFla	gb|AOOT01105045.1|:1-828
	NuwaI-1_TFla	gb|AOOT01031852.1|:1-689
	NuwaI-1_TFla	gb|AOOT01107447.1|:1-649
	NuwaI-1_TFla	gb|AOOT01104475.1|:1-634
	NuwaI-1_TFla	gb|AOOT01031851.1|:511-1082
	NuwaI-1_TFla	gb|AOOT01117129.1|:1-562
	NuwaI-1_TFla	gb|AOOT01128081.1|:31-580
	NuwaI-1_TFla	gb|AOOT01055784.1|:4-583
	NuwaI-1_TFla	gb|AOOT01077495.1|:1-307
	NuwaI-1_TFla	gb|AOOT01116414.1|:1-289
	NuwaI-1_TFla	gb|AOOT01105929.1|:1092-1207
	NuwaI-1_TFla	gb|AOOT01013332.1|:2581-2667
	NuwaI-1_TFla	gb|AOOT01112235.1|:451-531
	NuwaI-1_TFla	gb|AOOT01098573.1|:1271-1351
	NuwaI-1_TFla	gb|AOOT01008045.1|:141-220
	NuwaI-1_TFla	gb|AOOT01126794.1|:3780-3859
	NuwaI-1_TFla	gb|AOOT01133443.1|:322-401
	NuwaI-1_TFla	gb|AOOT01014968.1|:3269-3346
	NuwaI-1_TFla	gb|AOOT01093611.1|:1310-1388
	NuwaI-1_TFla	gb|AOOT01003654.1|:354-432
	NuwaI-1_TFla	gb|AOOT01014497.1|:271-349
	NuwaI-1_TFla	gb|AOOT01104241.1|:98-176
	NuwaI-1_TFla	gb|AOOT01074650.1|:7626-7704
	NuwaI-1_TFla	gb|AOOT01122606.1|:206-283
	NuwaI-1_TFla	gb|AOOT01122604.1|:754-831
	NuwaI-1_TFla	gb|AOOT01060357.1|:480-557
	NuwaI-1_TFla	gb|AOOT01010328.1|:7431-7508
	NuwaI-1_TFla	gb|AOOT01125638.1|:135-212
	NuwaI-1_TFla	gb|AOOT01115923.1|:1210-1287
	NuwaI-1_TFla	gb|AOOT01106240.1|:1255-1332
	NuwaI-1_TFla	gb|AOOT01098516.1|:9652-9729
	NuwaI-1_TFla	gb|AOOT01029047.1|:941-1018

NuwaI-1_TFla	gb|AOOT01014026.1|:28930-29007
NuwaI-1_TFla	gb|AOOT01045390.1|:1836-1913
NuwaI-1_TFla	gb|AOOT01008876.1|:6958-7035
NuwaI-1_TFla	gb|AOOT01124054.1|:1004-1081
NuwaI-1_TFla	gb|AOOT01109519.1|:1089-1164
NuwaI-1_TFla	gb|AOOT01060307.1|:6381-6454
NuwaI-1_TFla	gb|AOOT01004671.1|:15278-15351
NuwaI-1_TFla	gb|AOOT01005229.1|:2195-2268
NuwaI-1_TFla	gb|AOOT01124588.1|:64-137
NuwaI-1_TFla	gb|AOOT01049700.1|:335-406
NuwaI-1_TFla	gb|AOOT01055246.1|:4279-4349
NuwaI-1_TFla	gb|AOOT01115226.1|:688-754
NuwaI-1_TFla	gb|AOOT01089237.1|:5378-5436
NuwaI-1_TFla	gb|AOOT01117374.1|:1307-1356
NuwaI-1_TFla	gb|AOOT01048307.1|:17240-23767
NuwaI-1_TFla	gb|AOOT01023480.1|:55-1927
NuwaI-1_TFla	gb|AOOT01105368.1|:753-2553
NuwaI-1_TFla	gb|AOOT01083230.1|:16721-18484
NuwaI-1_TFla	gb|AOOT01129837.1|:1-719
NuwaI-1_TFla	gb|AOOT01105846.1|:1-609
NuwaI-1_TFla	gb|AOOT01129620.1|:2-542
NuwaI-1_TFla	gb|AOOT01008245.1|:234-729
NuwaI-1_TFla	gb|AOOT01023479.1|:3797-4302
NuwaI-1_TFla	gb|AOOT01120212.1|:588-1047
NuwaI-1_TFla	gb|AOOT01120798.1|:5-455
NuwaI-1_TFla	gb|AOOT01120800.1|:2-374
NuwaI-1_TFla	gb|AOOT01129836.1|:5-370
NuwaI-1_TFla	gb|AOOT01120799.1|:2-359
NuwaI-1_TFla	gb|AOOT01129838.1|:1-340
NuwaI-1_TFla	gb|AOOT01120200.1|:476-777
NuwaI-1_TFla	gb|AOOT01129839.1|:2-298
NuwaI-1_TFla	gb|AOOT01098823.1|:7-312
NuwaI-1_TFla	gb|AOOT01025785.1|:7539-7795
NuwaI-1_TFla	gb|AOOT01126439.1|:4-250
NuwaI-1_TFla	gb|AOOT01105847.1|:38-299
NuwaI-1_TFla	gb|AOOT01120197.1|:680-825
NuwaI-1_TFla	gb|AOOT01120209.1|:648-787
NuwaI-1_TFla	gb|AOOT01120210.1|:1-88
NuwaI-1_TFla	gb|AOOT01078630.1|:1582-1668
NuwaI-1_TFla	gb|AOOT01111749.1|:116-196
NuwaI-1_TFla	gb|AOOT01109578.1|:401-481
NuwaI-1_TFla	gb|AOOT01116189.1|:2091-2171
NuwaI-1_TFla	gb|AOOT01008345.1|:18815-18894
NuwaI-1_TFla	gb|AOOT01039403.1|:2092-2171

	NuwaI-1_TFla	gb|AOOT01129292.1|:93-171
	NuwaI-1_TFla	gb|AOOT01125672.1|:4-82
	NuwaI-1_TFla	gb|AOOT01113597.1|:476-554
	NuwaI-1_TFla	gb|AOOT01106682.1|:314-392
	NuwaI-1_TFla	gb|AOOT01082772.1|:551-629
	NuwaI-1_TFla	gb|AOOT01070722.1|:404-482
	NuwaI-1_TFla	gb|AOOT01009210.1|:37616-37694
	NuwaI-1_TFla	gb|AOOT01097985.1|:948-1026
	NuwaI-1_TFla	gb|AOOT01095376.1|:31-109
	NuwaI-1_TFla	gb|AOOT01114226.1|:548-625
	NuwaI-1_TFla	gb|AOOT01111278.1|:713-790
	NuwaI-1_TFla	gb|AOOT01113911.1|:7-84
	NuwaI-1_TFla	gb|AOOT01112965.1|:6-83
	NuwaI-1_TFla	gb|AOOT01107183.1|:299-376
	NuwaI-1_TFla	gb|AOOT01093467.1|:339-416
	NuwaI-1_TFla	gb|AOOT01087763.1|:3388-3465
	NuwaI-1_TFla	gb|AOOT01086717.1|:6348-6425
	NuwaI-1_TFla	gb|AOOT01063298.1|:529-606
	NuwaI-1_TFla	gb|AOOT01055737.1|:52-129
	NuwaI-1_TFla	gb|AOOT01036393.1|:135-212
	NuwaI-1_TFla	gb|AOOT01023484.1|:3806-3883
	NuwaI-1_TFla	gb|AOOT01117534.1|:8-85
	NuwaI-1_TFla	gb|AOOT01002551.1|:4222-4297
	NuwaI-1_TFla	gb|AOOT01132032.1|:2561-2636
	NuwaI-1_TFla	gb|AOOT01132029.1|:408-483
	NuwaI-1_TFla	gb|AOOT01022281.1|:3202-3276
	NuwaI-1_TFla	gb|AOOT01128744.1|:2-76
	NuwaI-1_TFla	gb|AOOT01110656.1|:1-74
	NuwaI-1_TFla	gb|AOOT01004239.1|:17791-17864
	NuwaI-1_TFla	gb|AOOT01103741.1|:704-777
	NuwaI-1_TFla	gb|AOOT01016244.1|:17865-17938
	NuwaI-1_TFla	gb|AOOT01109072.1|:79-152
	NuwaI-1_TFla	gb|AOOT01010857.1|:10944-11017
	NuwaI-1_TFla	gb|AOOT01012396.1|:2592-2664
	NuwaI-1_TFla	gb|AOOT01120144.1|:1-71
	NuwaI-1_TFla	gb|AOOT01028619.1|:689-758
	NuwaI-1_TFla	gb|AOOT01110557.1|:827-884
	NuwaI-1_TFla	gb|AOOT01119611.1|:1435-1484
Takifugu rubripes	*NuwaI-1_TRub*	emb|CAAB02006272.1|:5230-8069
	NuwaI-1_TRub	emb|CAAB02011275.1|:18733-25257
	NuwaI-1_TRub	emb|CAAB02013295.1|:970-2581
	NuwaI-1_TRub	emb|CAAB02024132.1|:1-1397
	NuwaI-1_TRub	emb|CAAB02027860.1|:1-1255
	NuwaI-1_TRub	emb|CAAB02028680.1|:1-828

	NuwaI-1_TRub	emb|CAAB02016926.1|:1-728
	NuwaI-1_TRub	emb|CAAB02026226.1|:1-649
	NuwaI-1_TRub	emb|CAAB02029309.1|:1-640
	NuwaI-1_TRub	emb|CAAB02006040.1|:1172-1723
	NuwaI-1_TRub	emb|CAAB02014088.1|:1-515
	NuwaI-1_TRub	emb|CAAB02017780.1|:1-289
	NuwaI-1_TRub	emb|CAAB02013191.1|:1-171
	NuwaI-1_TRub	emb|CAAB02027859.1|:1080-1223
	NuwaI-1_TRub	emb|CAAB02013234.1|:5953-8920
	NuwaI-1_TRub	emb|CAAB02004452.1|:3010-5572
	NuwaI-1_TRub	emb|CAAB02006216.1|:2-2292
	NuwaI-1_TRub	emb|CAAB02004124.1|:25702-27747
	NuwaI-1_TRub	emb|CAAB02007337.1|:89986-92004
	NuwaI-1_TRub	emb|CAAB02011486.1|:55-1935
	NuwaI-1_TRub	emb|CAAB02028371.1|:1348-3153
	NuwaI-1_TRub	emb|CAAB02027940.1|:1-1081
	NuwaI-1_TRub	emb|CAAB02018126.1|:1729-2664
	NuwaI-1_TRub	emb|CAAB02019861.1|:1-878
	NuwaI-1_TRub	emb|CAAB02006040.1|:1-616
	NuwaI-1_TRub	emb|CAAB02011809.1|:39770-40349
	NuwaI-1_TRub	emb|CAAB02011485.1|:3797-4302
	NuwaI-1_TRub	emb|CAAB02013886.1|:724-1183
	NuwaI-1_TRub	emb|CAAB02012164.1|:6919-7226
	NuwaI-1_TRub	emb|CAAB02013881.1|:1486-1787
	NuwaI-1_TRub	emb|CAAB02004968.1|:42332-42588
	NuwaI-1_TRub	emb|CAAB02013880.1|:680-826
	NuwaI-1_TRub	emb|CAAB02013883.1|:649-789
	NuwaI-1_TRub	emb|CAAB02019860.1|:841-942
	NuwaI-1_TRub	emb|CAAB02013884.1|:1-100
Thunnus orientalis	*NuwaI-1_TOri*	dbj|BADN01058449.1|:1564-4997
	NuwaI-1_TOri	dbj|BADN01074808.1|:7154-9697
	NuwaI-1_TOri	dbj|BADN01002231.1|:5-1354
	NuwaI-1_TOri	dbj|BADN01028106.1|:8-1199
	NuwaI-1_TOri	dbj|BADN01063732.1|:1073-2296
	NuwaI-1_TOri	dbj|BADN01063733.1|:1-1123
	NuwaI-1_TOri	dbj|BADN01087412.1|:4086-5103
	NuwaI-1_TOri	dbj|BADN01068580.1|:9139-10127
	NuwaI-1_TOri	dbj|BADN01120619.1|:116-958
	NuwaI-1_TOri	dbj|BADN01043323.1|:33-883
	NuwaI-1_TOri	dbj|BADN01005625.1|:960-1753
	NuwaI-1_TOri	dbj|BADN01118427.1|:838-1580
	NuwaI-1_TOri	dbj|BADN01096103.1|:17341-18085
	NuwaI-1_TOri	dbj|BADN01012170.1|:1-626
	NuwaI-1_TOri	dbj|BADN01126314.1|:2-618

NuwaI-1_TOri	dbj|BADN01008517.1|:4903-5397
NuwaI-1_TOri	dbj|BADN01119850.1|:471-956
NuwaI-1_TOri	dbj|BADN01089596.1|:5-481
NuwaI-1_TOri	dbj|BADN01097842.1|:9525-9956
NuwaI-1_TOri	dbj|BADN01094569.1|:582-1003
NuwaI-1_TOri	dbj|BADN01012169.1|:5376-5760
NuwaI-1_TOri	dbj|BADN01039740.1|:7972-8313
NuwaI-1_TOri	dbj|BADN01067035.1|:7110-7449
NuwaI-1_TOri	dbj|BADN01032365.1|:15666-16005
NuwaI-1_TOri	dbj|BADN01059980.1|:5173-6665
NuwaI-1_TOri	dbj|BADN01096978.1|:410-705
NuwaI-1_TOri	dbj|BADN01028105.1|:30687-30974
NuwaI-1_TOri	dbj|BADN01002860.1|:1-285
NuwaI-1_TOri	dbj|BADN01044386.1|:3-281
NuwaI-1_TOri	dbj|BADN01096979.1|:2-266
NuwaI-1_TOri	dbj|BADN01002901.1|:671-928
NuwaI-1_TOri	dbj|BADN01048491.1|:1339-1605
NuwaI-1_TOri	dbj|BADN01128559.1|:1086-1302
NuwaI-1_TOri	dbj|BADN01116510.1|:67-280
NuwaI-1_TOri	dbj|BADN01062811.1|:9353-9554
NuwaI-1_TOri	dbj|BADN01117118.1|:1-202
NuwaI-1_TOri	dbj|BADN01026522.1|:253-2531
NuwaI-1_TOri	dbj|BADN01044596.1|:99-1457
NuwaI-1_TOri	dbj|BADN01126313.1|:2923-3098
NuwaI-1_TOri	dbj|BADN01088728.1|:3051-3225
NuwaI-1_TOri	dbj|BADN01025023.1|:6-179
NuwaI-1_TOri	dbj|BADN01065029.1|:976-1196
NuwaI-1_TOri	dbj|BADN01048492.1|:3-164
NuwaI-1_TOri	dbj|BADN01077888.1|:4702-6292
NuwaI-1_TOri	dbj|BADN01026432.1|:3680-3833
NuwaI-1_TOri	dbj|BADN01127037.1|:866-1018
NuwaI-1_TOri	dbj|BADN01122693.1|:2066-2275
NuwaI-1_TOri	dbj|BADN01026427.1|:8324-8472
NuwaI-1_TOri	dbj|BADN01047596.1|:133-276
NuwaI-1_TOri	dbj|BADN01083916.1|:2307-2482
NuwaI-1_TOri	dbj|BADN01061676.1|:3551-3686
NuwaI-1_TOri	dbj|BADN01001926.1|:4947-5082
NuwaI-1_TOri	dbj|BADN01059764.1|:295-431
NuwaI-1_TOri	dbj|BADN01072298.1|:1305-2531
NuwaI-1_TOri	dbj|BADN01061791.1|:6204-6333
NuwaI-1_TOri	dbj|BADN01094689.1|:3996-4117
NuwaI-1_TOri	dbj|BADN01111814.1|:3749-3877
NuwaI-1_TOri	dbj|BADN01095016.1|:13804-13929
NuwaI-1_TOri	dbj|BADN01028161.1|:5634-5755

NuwaI-1_TOri	dbj|BADN01076116.1|:6495-6617
NuwaI-1_TOri	dbj|BADN01058562.1|:667-1169
NuwaI-1_TOri	dbj|BADN01062474.1|:1808-1929
NuwaI-1_TOri	dbj|BADN01037544.1|:13364-13485
NuwaI-1_TOri	dbj|BADN01032988.1|:1-121
NuwaI-1_TOri	dbj|BADN01065518.1|:11454-11570
NuwaI-1_TOri	dbj|BADN01064519.1|:698-815
NuwaI-1_TOri	dbj|BADN01070583.1|:9306-9421
NuwaI-1_TOri	dbj|BADN01015169.1|:425-540
NuwaI-1_TOri	dbj|BADN01083576.1|:2181-2293
NuwaI-1_TOri	dbj|BADN01131322.1|:2233-2347
NuwaI-1_TOri	dbj|BADN01043121.1|:19821-19933
NuwaI-1_TOri	dbj|BADN01087413.1|:2-115
NuwaI-1_TOri	dbj|BADN01093913.1|:11220-11332
NuwaI-1_TOri	dbj|BADN01013627.1|:30092-30205
NuwaI-1_TOri	dbj|BADN01057332.1|:1959-2070
NuwaI-1_TOri	dbj|BADN01025416.1|:2883-2991
NuwaI-1_TOri	dbj|BADN01052254.1|:3080-3185
NuwaI-1_TOri	dbj|BADN01121733.1|:1-101
NuwaI-1_TOri	dbj|BADN01121577.1|:563-663
NuwaI-1_TOri	dbj|BADN01108890.1|:5652-6858
NuwaI-1_TOri	dbj|BADN01115917.1|:503-594
NuwaI-1_TOri	dbj|BADN01118995.1|:336-427
NuwaI-1_TOri	dbj|BADN01067537.1|:1044-1134
NuwaI-1_TOri	dbj|BADN01027911.1|:623-1964
NuwaI-1_TOri	dbj|BADN01065028.1|:2951-3037
NuwaI-1_TOri	dbj|BADN01093121.1|:629-714
NuwaI-1_TOri	dbj|BADN01119851.1|:1-85
NuwaI-1_TOri	dbj|BADN01040715.1|:3662-3745
NuwaI-1_TOri	dbj|BADN01095049.1|:2347-2428
NuwaI-1_TOri	dbj|BADN01003814.1|:38240-38315
NuwaI-1_TOri	dbj|BADN01041247.1|:149-225
NuwaI-1_TOri	dbj|BADN01088814.1|:1-74
NuwaI-1_TOri	dbj|BADN01016798.1|:7043-7115
NuwaI-1_TOri	dbj|BADN01005311.1|:8057-8126
NuwaI-1_TOri	dbj|BADN01050111.1|:11515-11583
NuwaI-1_TOri	dbj|BADN01056088.1|:3980-4042
NuwaI-1_TOri	dbj|BADN01069258.1|:3041-3103
NuwaI-1_TOri	dbj|BADN01047595.1|:5466-5528
NuwaI-1_TOri	dbj|BADN01031201.1|:7892-7954
NuwaI-1_TOri	dbj|BADN01008208.1|:11176-11237
NuwaI-1_TOri	dbj|BADN01020348.1|:12857-12918
NuwaI-1_TOri	dbj|BADN01052057.1|:3267-3327
NuwaI-1_TOri	dbj|BADN01065530.1|:1820-1877

NuwaI-1_TOri	dbj|BADN01085801.1|:1553-1609
NuwaI-1_TOri	dbj|BADN01013153.1|:4681-4737
NuwaI-1_TOri	dbj|BADN01110352.1|:912-967
NuwaI-1_TOri	dbj|BADN01122694.1|:2222-2276
NuwaI-1_TOri	dbj|BADN01091390.1|:301-355
NuwaI-1_TOri	dbj|BADN01126713.1|:980-1034
NuwaI-1_TOri	dbj|BADN01059989.1|:4359-4413
NuwaI-1_TOri	dbj|BADN01121677.1|:767-821
NuwaI-1_TOri	dbj|BADN01111368.1|:2400-2453
NuwaI-1_TOri	dbj|BADN01005473.1|:2673-2726
NuwaI-1_TOri	dbj|BADN01122200.1|:3302-3354
NuwaI-1_TOri	dbj|BADN01115663.1|:712-763
NuwaI-1_TOri	dbj|BADN01091029.1|:9687-13170
NuwaI-1_TOri	dbj|BADN01123310.1|:591-3025
NuwaI-1_TOri	dbj|BADN01007369.1|:1248-2598
NuwaI-1_TOri	dbj|BADN01128487.1|:1-1363
NuwaI-1_TOri	dbj|BADN01117285.1|:492-1678
NuwaI-1_TOri	dbj|BADN01112564.1|:658-1770
NuwaI-1_TOri	dbj|BADN01047479.1|:13307-14212
NuwaI-1_TOri	dbj|BADN01097011.1|:509-1758
NuwaI-1_TOri	dbj|BADN01063214.1|:955-3043
NuwaI-1_TOri	dbj|BADN01043129.1|:1518-2263
NuwaI-1_TOri	dbj|BADN01082564.1|:4-633
NuwaI-1_TOri	dbj|BADN01057994.1|:10014-10657
NuwaI-1_TOri	dbj|BADN01013411.1|:1-625
NuwaI-1_TOri	dbj|BADN01013410.1|:3789-4351
NuwaI-1_TOri	dbj|BADN01124061.1|:884-1428
NuwaI-1_TOri	dbj|BADN01087767.1|:1-573
NuwaI-1_TOri	dbj|BADN01047480.1|:1-524
NuwaI-1_TOri	dbj|BADN01054079.1|:25-587
NuwaI-1_TOri	dbj|BADN01126635.1|:2828-3267
NuwaI-1_TOri	dbj|BADN01054078.1|:2415-2808
NuwaI-1_TOri	dbj|BADN01043034.1|:5702-6092
NuwaI-1_TOri	dbj|BADN01028994.1|:6524-6902
NuwaI-1_TOri	dbj|BADN01043128.1|:8272-8644
NuwaI-1_TOri	dbj|BADN01076790.1|:16215-16574
NuwaI-1_TOri	dbj|BADN01115132.1|:2058-2407
NuwaI-1_TOri	dbj|BADN01043035.1|:20-348
NuwaI-1_TOri	dbj|BADN01011316.1|:29-316
NuwaI-1_TOri	dbj|BADN01043020.1|:1-235
NuwaI-1_TOri	dbj|BADN01010592.1|:3177-3388
NuwaI-1_TOri	dbj|BADN01103500.1|:358-506
NuwaI-1_TOri	dbj|BADN01122142.1|:3403-3595
NuwaI-1_TOri	dbj|BADN01000501.1|:24383-25707

NuwaI-1_TOri	dbj|BADN01124382.1|:4863-5019
NuwaI-1_TOri	dbj|BADN01053339.1|:1304-1460
NuwaI-1_TOri	dbj|BADN01107127.1|:3258-3405
NuwaI-1_TOri	dbj|BADN01108116.1|:9704-9846
NuwaI-1_TOri	dbj|BADN01043319.1|:5040-6338
NuwaI-1_TOri	dbj|BADN01051162.1|:17638-18947
NuwaI-1_TOri	dbj|BADN01033638.1|:1336-1471
NuwaI-1_TOri	dbj|BADN01048236.1|:21163-21299
NuwaI-1_TOri	dbj|BADN01047262.1|:4450-4579
NuwaI-1_TOri	dbj|BADN01005829.1|:664-793
NuwaI-1_TOri	dbj|BADN01044939.1|:18760-18889
NuwaI-1_TOri	dbj|BADN01001869.1|:191-319
NuwaI-1_TOri	dbj|BADN01028034.1|:1649-1771
NuwaI-1_TOri	dbj|BADN01037550.1|:6406-6526
NuwaI-1_TOri	dbj|BADN01046216.1|:11228-11348
NuwaI-1_TOri	dbj|BADN01078564.1|:7187-7309
NuwaI-1_TOri	dbj|BADN01042179.1|:3968-4090
NuwaI-1_TOri	dbj|BADN01003927.1|:5414-5536
NuwaI-1_TOri	dbj|BADN01012044.1|:4703-4837
NuwaI-1_TOri	dbj|BADN01023020.1|:1-121
NuwaI-1_TOri	dbj|BADN01019810.1|:2461-2581
NuwaI-1_TOri	dbj|BADN01005186.1|:25931-26052
NuwaI-1_TOri	dbj|BADN01046753.1|:3940-4048
NuwaI-1_TOri	dbj|BADN01022995.1|:14086-14192
NuwaI-1_TOri	dbj|BADN01095155.1|:3776-4715
NuwaI-1_TOri	dbj|BADN01029097.1|:711-2441
NuwaI-1_TOri	dbj|BADN01065381.1|:4528-4624
NuwaI-1_TOri	dbj|BADN01051033.1|:3222-3380
NuwaI-1_TOri	dbj|BADN01095381.1|:3333-3430
NuwaI-1_TOri	dbj|BADN01078887.1|:3474-4851
NuwaI-1_TOri	dbj|BADN01075277.1|:2820-2915
NuwaI-1_TOri	dbj|BADN01095182.1|:1974-2066
NuwaI-1_TOri	dbj|BADN01124566.1|:500-595
NuwaI-1_TOri	dbj|BADN01036754.1|:802-897
NuwaI-1_TOri	dbj|BADN01058739.1|:2651-2745
NuwaI-1_TOri	dbj|BADN01056171.1|:933-1025
NuwaI-1_TOri	dbj|BADN01063529.1|:2519-2609
NuwaI-1_TOri	dbj|BADN01041322.1|:3774-3865
NuwaI-1_TOri	dbj|BADN01131481.1|:1596-1678
NuwaI-1_TOri	dbj|BADN01089816.1|:1-80
NuwaI-1_TOri	dbj|BADN01039792.1|:1516-1589
NuwaI-1_TOri	dbj|BADN01036753.1|:1636-1708
NuwaI-1_TOri	dbj|BADN01029534.1|:401-473
NuwaI-1_TOri	dbj|BADN01038580.1|:1368-1439

NuwaI-1_TOri	dbj|BADN01128989.1|:2-71
NuwaI-1_TOri	dbj|BADN01026323.1|:3786-3854
NuwaI-1_TOri	dbj|BADN01060521.1|:5721-5789
NuwaI-1_TOri	dbj|BADN01092340.1|:19253-19318
NuwaI-1_TOri	dbj|BADN01084149.1|:179-242
NuwaI-1_TOri	dbj|BADN01067809.1|:2036-2099
NuwaI-1_TOri	dbj|BADN01100778.1|:1602-1664
NuwaI-1_TOri	dbj|BADN01132072.1|:404-464
NuwaI-1_TOri	dbj|BADN01117641.1|:583-642
NuwaI-1_TOri	dbj|BADN01081978.1|:4-60
NuwaI-1_TOri	dbj|BADN01113781.1|:4-60
NuwaI-1_TOri	dbj|BADN01053703.1|:2292-2348
NuwaI-1_TOri	dbj|BADN01036820.1|:1557-1613
NuwaI-1_TOri	dbj|BADN01044538.1|:1674-1728
NuwaI-1_TOri	dbj|BADN01057188.1|:4354-4408
NuwaI-1_TOri	dbj|BADN01053766.1|:4652-4706
NuwaI-1_TOri	dbj|BADN01046491.1|:233-287
NuwaI-1_TOri	dbj|BADN01043406.1|:28462-28514
NuwaI-1_TOri	dbj|BADN01081977.1|:3143-3195

Table S8. Positions of *NuwaII* transposons in the corresponding genome

Species	Families	Scaffold:start-end
Acropora digitifera	*NuwaII-1_ADig*	dbj|BACK01008037.1|:5187-6321
	NuwaII-1_ADig	dbj|BACK01031808.1|:53-1179
	NuwaII-1_ADig	dbj|BACK01006636.1|:9307-10451
	NuwaII-1_ADig	dbj|BACK01037618.1|:11890-12879
	NuwaII-1_ADig	dbj|BACK01049284.1|:5493-6847
	NuwaII-1_ADig	dbj|BACK01019312.1|:18148-18770
	NuwaII-1_ADig	dbj|BACK01044634.1|:6755-7732
	NuwaII-1_ADig	dbj|BACK01004396.1|:33360-34372
	NuwaII-1_ADig	dbj|BACK01034143.1|:1460-2873
	NuwaII-1_ADig	dbj|BACK01039893.1|:1-233
	NuwaII-1_ADig	dbj|BACK01051264.1|:2884-6701
	NuwaII-1_ADig	dbj|BACK01014744.1|:552-1534
	NuwaII-1_ADig	dbj|BACK01006786.1|:11278-12337
	NuwaII-1_ADig	dbj|BACK01013665.1|:849-1862
	NuwaII-1_ADig	dbj|BACK01042925.1|:2001-4412
	NuwaII-1_ADig	dbj|BACK01040793.1|:1-376
	NuwaII-1_ADig	dbj|BACK01039892.1|:5432-5837
	NuwaII-1_ADig	dbj|BACK01029457.1|:1-407
	NuwaII-1_ADig	dbj|BACK01020086.1|:15786-16394
	NuwaII-1_ADig	dbj|BACK01014637.1|:17298-17681
	NuwaII-1_ADig	dbj|BACK01011703.1|:1452-1844
	NuwaII-1_ADig	dbj|BACK01031551.1|:493-1295
	NuwaII-1_ADig	dbj|BACK01007522.1|:1-261
	NuwaII-1_ADig	dbj|BACK01006493.1|:1-263
	NuwaII-1_ADig	dbj|BACK01018917.1|:1-258
	NuwaII-1_ADig	dbj|BACK01044599.1|:5390-5662
	NuwaII-1_ADig	dbj|BACK01011704.1|:1-233
	NuwaII-1_ADig	dbj|BACK01047039.1|:909-1650
	NuwaII-1_ADig	dbj|BACK01025151.1|:4525-5249
	NuwaII-1_ADig	dbj|BACK01042384.1|:18583-19312
	NuwaII-1_ADig	dbj|BACK01011912.1|:2259-2991
	NuwaII-1_ADig	dbj|BACK01045356.1|:4181-8275
	NuwaII-1_ADig	dbj|BACK01018671.1|:951-1323
	NuwaII-1_ADig	dbj|BACK01025863.1|:5551-6318
	NuwaII-1_ADig	dbj|BACK01036247.1|:1-184
	NuwaII-1_ADig	dbj|BACK01036630.1|:1896-2663
	NuwaII-1_ADig	dbj|BACK01025853.1|:7607-8225
	NuwaII-1_ADig	dbj|BACK01046709.1|:690-1064
	NuwaII-1_ADig	dbj|BACK01045179.1|:2958-3649
	NuwaII-1_ADig	dbj|BACK01010187.1|:8719-9416
	NuwaII-1_ADig	dbj|BACK01032238.1|:13977-14903
	NuwaII-1_ADig	dbj|BACK01035268.1|:724-856

NuwaII-1_ADig	dbj|BACK01003435.1|:2294-3442
NuwaII-1_ADig	dbj|BACK01035266.1|:832-1015
NuwaII-1_ADig	dbj|BACK01022272.1|:35456-35736
NuwaII-1_ADig	dbj|BACK01018674.1|:5444-6180
NuwaII-1_ADig	dbj|BACK01019096.1|:22532-23300
NuwaII-1_ADig	dbj|BACK01032129.1|:9909-10177
NuwaII-1_ADig	dbj|BACK01040027.1|:4029-4222
NuwaII-1_ADig	dbj|BACK01032901.1|:10565-10741
NuwaII-1_ADig	dbj|BACK01030644.1|:3843-4244
NuwaII-1_ADig	dbj|BACK01030360.1|:2273-2708
NuwaII-1_ADig	dbj|BACK01003411.1|:33421-33619
NuwaII-1_ADig	dbj|BACK01010684.1|:18617-18793
NuwaII-1_ADig	dbj|BACK01049245.1|:1943-2119
NuwaII-1_ADig	dbj|BACK01030304.1|:1466-2209
NuwaII-1_ADig	dbj|BACK01052868.1|:1064-1272
NuwaII-1_ADig	dbj|BACK01026066.1|:32-227
NuwaII-1_ADig	dbj|BACK01004782.1|:1781-2002
NuwaII-1_ADig	dbj|BACK01039191.1|:1-566
NuwaII-1_ADig	dbj|BACK01034752.1|:21-281
NuwaII-1_ADig	dbj|BACK01003436.1|:2602-2828
NuwaII-1_ADig	dbj|BACK01030646.1|:5187-5328
NuwaII-1_ADig	dbj|BACK01000470.1|:11209-11285
NuwaII-1_ADig	dbj|BACK01049000.1|:1080-1217
NuwaII-1_ADig	dbj|BACK01035639.1|:3000-3201
NuwaII-1_ADig	dbj|BACK01036259.1|:552-646
NuwaII-1_ADig	dbj|BACK01034163.1|:2808-2874
NuwaII-1_ADig	dbj|BACK01032290.1|:5836-5902
NuwaII-1_ADig	dbj|BACK01045425.1|:2124-2237
NuwaII-1_ADig	dbj|BACK01011928.1|:408-816
NuwaII-1_ADig	dbj|BACK01011288.1|:17845-18035
NuwaII-1_ADig	dbj|BACK01007308.1|:412-817
NuwaII-1_ADig	dbj|BACK01034765.1|:8183-8245
NuwaII-1_ADig	dbj|BACK01007302.1|:10055-10468
NuwaII-1_ADig	dbj|BACK01001666.1|:5491-5636
NuwaII-1_ADig	dbj|BACK01038729.1|:4681-5376
NuwaII-1_ADig	dbj|BACK01018677.1|:3276-3408
NuwaII-1_ADig	dbj|BACK01011925.1|:25686-26099
NuwaII-1_ADig	dbj|BACK01034466.1|:5869-6122
NuwaII-1_ADig	dbj|BACK01041867.1|:749-937
NuwaII-1_ADig	dbj|BACK01006843.1|:1901-2278
NuwaII-1_ADig	dbj|BACK01010787.1|:15857-15937
NuwaII-1_ADig	dbj|BACK01034751.1|:1737-1807
NuwaII-1_ADig	dbj|BACK01018672.1|:1-94
NuwaII-1_ADig	dbj|BACK01049611.1|:3024-3167

NuwaII-1_ADig	dbj|BACK01008857.1|:906-977
NuwaII-1_ADig	dbj|BACK01049045.1|:11437-11576
NuwaII-1_ADig	dbj|BACK01021458.1|:23482-23577
NuwaII-1_ADig	dbj|BACK01031009.1|:46103-46170
NuwaII-1_ADig	dbj|BACK01023077.1|:1127-1176
NuwaII-1_ADig	dbj|BACK01023064.1|:1536-1585
NuwaII-1_ADig	dbj|BACK01004219.1|:3799-3848
NuwaII-1_ADig	dbj|BACK01044831.1|:9248-9297
NuwaII-1_ADig	dbj|BACK01022207.1|:1917-1965
NuwaII-1_ADig	dbj|BACK01015181.1|:6185-6234
NuwaII-1_ADig	dbj|BACK01008037.1|:5187-6321
NuwaII-1_ADig	dbj|BACK01031808.1|:53-1179
NuwaII-1_ADig	dbj|BACK01026246.1|:646-1777
NuwaII-1_ADig	dbj|BACK01041091.1|:12810-14360
NuwaII-1_ADig	dbj|BACK01037618.1|:11890-12879
NuwaII-1_ADig	dbj|BACK01051542.1|:1-862
NuwaII-1_ADig	dbj|BACK01001114.1|:21939-22597
NuwaII-1_ADig	dbj|BACK01028589.1|:11250-11926
NuwaII-1_ADig	dbj|BACK01044634.1|:6755-7732
NuwaII-1_ADig	dbj|BACK01004396.1|:33360-34296
NuwaII-1_ADig	dbj|BACK01024435.1|:2182-2858
NuwaII-1_ADig	dbj|BACK01034143.1|:1460-2873
NuwaII-1_ADig	dbj|BACK01027036.1|:5654-6137
NuwaII-1_ADig	dbj|BACK01039893.1|:972-2086
NuwaII-1_ADig	dbj|BACK01001945.1|:62-1079
NuwaII-1_ADig	dbj|BACK01013236.1|:14-451
NuwaII-1_ADig	dbj|BACK01021156.1|:1303-2050
NuwaII-1_ADig	dbj|BACK01023168.1|:2-439
NuwaII-1_ADig	dbj|BACK01022652.1|:1624-2473
NuwaII-1_ADig	dbj|BACK01001115.1|:1-404
NuwaII-1_ADig	dbj|BACK01025723.1|:6486-6810
NuwaII-1_ADig	dbj|BACK01023202.1|:9185-9486
NuwaII-1_ADig	dbj|BACK01029947.1|:2252-2484
NuwaII-1_ADig	dbj|BACK01032719.1|:2777-3306
NuwaII-1_ADig	dbj|BACK01043491.1|:1257-1996
NuwaII-1_ADig	dbj|BACK01043499.1|:2642-3388
NuwaII-1_ADig	dbj|BACK01011351.1|:1-7705
NuwaII-1_ADig	dbj|BACK01012141.1|:12840-13368
NuwaII-1_ADig	dbj|BACK01045536.1|:5778-6537
NuwaII-1_ADig	dbj|BACK01012663.1|:2854-3053
NuwaII-1_ADig	dbj|BACK01049037.1|:2866-3608
NuwaII-1_ADig	dbj|BACK01028787.1|:1467-1837
NuwaII-1_ADig	dbj|BACK01028777.1|:4261-4710
NuwaII-1_ADig	dbj|BACK01042845.1|:515-1052

NuwaII-1_ADig	dbj|BACK01001986.1|:16574-16941
NuwaII-1_ADig	dbj|BACK01033176.1|:2558-3289
NuwaII-1_ADig	dbj|BACK01046710.1|:4958-5744
NuwaII-1_ADig	dbj|BACK01012740.1|:9507-9906
NuwaII-1_ADig	dbj|BACK01046709.1|:1307-1490
NuwaII-1_ADig	dbj|BACK01028783.1|:4286-5023
NuwaII-1_ADig	dbj|BACK01030114.1|:1962-2599
NuwaII-1_ADig	dbj|BACK01035268.1|:1149-1523
NuwaII-1_ADig	dbj|BACK01035266.1|:1258-1638
NuwaII-1_ADig	dbj|BACK01022272.1|:36381-36708
NuwaII-1_ADig	dbj|BACK01045737.1|:3161-3415
NuwaII-1_ADig	dbj|BACK01051044.1|:14106-14774
NuwaII-1_ADig	dbj|BACK01021623.1|:11559-12299
NuwaII-1_ADig	dbj|BACK01011817.1|:6274-6385
NuwaII-1_ADig	dbj|BACK01047859.1|:848-958
NuwaII-1_ADig	dbj|BACK01043495.1|:1407-2060
NuwaII-1_ADig	dbj|BACK01028527.1|:310-466
NuwaII-1_ADig	dbj|BACK01020008.1|:41-230
NuwaII-1_ADig	dbj|BACK01042389.1|:8949-9692
NuwaII-1_ADig	dbj|BACK01048704.1|:2528-3104
NuwaII-1_ADig	dbj|BACK01051795.1|:1102-1469
NuwaII-1_ADig	dbj|BACK01024330.1|:10050-10847
NuwaII-1_ADig	dbj|BACK01041223.1|:2153-2681
NuwaII-1_ADig	dbj|BACK01044579.1|:25848-25968
NuwaII-1_ADig	dbj|BACK01010366.1|:1226-1407
NuwaII-1_ADig	dbj|BACK01011350.1|:1520-1613
NuwaII-1_ADig	dbj|BACK01026293.1|:4667-4805
NuwaII-1_ADig	dbj|BACK01029880.1|:787-956
NuwaII-1_ADig	dbj|BACK01006063.1|:3524-4968
NuwaII-1_ADig	dbj|BACK01022654.1|:1936-2129
NuwaII-1_ADig	dbj|BACK01041485.1|:5591-5717
NuwaII-1_ADig	dbj|BACK01019939.1|:3382-3566
NuwaII-1_ADig	dbj|BACK01037630.1|:746-1477
NuwaII-1_ADig	dbj|BACK01031550.1|:1-68
NuwaII-1_ADig	dbj|BACK01002368.1|:659-768
NuwaII-1_ADig	dbj|BACK01029823.1|:5871-6091
NuwaII-1_ADig	dbj|BACK01039318.1|:7070-7202
NuwaII-1_ADig	dbj|BACK01014265.1|:4978-5057
NuwaII-1_ADig	dbj|BACK01001988.1|:4361-4430
NuwaII-1_ADig	dbj|BACK01000565.1|:2236-2302
NuwaII-1_ADig	dbj|BACK01010649.1|:16-206
NuwaII-1_ADig	dbj|BACK01001400.1|:830-1009
NuwaII-1_ADig	dbj|BACK01049045.1|:12153-12224
NuwaII-1_ADig	dbj|BACK01032574.1|:6220-6294

	NuwaII-1_ADig	dbj|BACK01049913.1|:13930-13979
	NuwaII-1_ADig	dbj|BACK01022153.1|:970-1019
	NuwaII-1_ADig	dbj|BACK01048202.1|:142-191
Branchiostoma floridae	*NuwaII-1_BFlo*	gb|ABEP02014146.1|:8224-13904
	NuwaII-1_BFlo	gb|ABEP02014146.1|:8224-13904
	NuwaII-1_BFlo	gb|ABEP02002554.1|:19097-19823
	NuwaII-1_BFlo	gb|ABEP02005432.1|:58452-59150
	NuwaII-1_BFlo	gb|ABEP02037147.1|:360-1077
	NuwaII-1_BFlo	gb|ABEP02023383.1|:35119-35846
	NuwaII-1_BFlo	gb|ABEP02004337.1|:585-1285
	NuwaII-1_BFlo	gb|ABEP02038613.1|:517-1235
	NuwaII-1_BFlo	gb|ABEP02027378.1|:20835-22145
	NuwaII-1_BFlo	gb|ABEP02027378.1|:20835-22145
	NuwaII-1_BFlo	gb|ABEP02025847.1|:5525-6823
	NuwaII-1_BFlo	gb|ABEP02025847.1|:5525-6823
	NuwaII-1_BFlo	gb|ABEP02025846.1|:955-1217
	NuwaII-1_BFlo	gb|ABEP02024236.1|:1911-2173
	NuwaII-1_BFlo	gb|ABEP02005815.1|:7808-9125
	NuwaII-1_BFlo	gb|ABEP02005815.1|:7808-9125
	NuwaII-1_BFlo	gb|ABEP02005464.1|:48849-50147
	NuwaII-1_BFlo	gb|ABEP02005464.1|:48849-50147
	NuwaII-1_BFlo	gb|ABEP02004648.1|:34910-36196
	NuwaII-1_BFlo	gb|ABEP02004648.1|:34910-36196
	NuwaII-1_BFlo	gb|ABEP02028903.1|:206-1513
	NuwaII-1_BFlo	gb|ABEP02023043.1|:60804-62096
	NuwaII-1_BFlo	gb|ABEP02005353.1|:12920-14233
	NuwaII-1_BFlo	gb|ABEP02005353.1|:12920-14233
	NuwaII-1_BFlo	gb|ABEP02000284.1|:1478-2763
	NuwaII-1_BFlo	gb|ABEP02000284.1|:1478-2763
	NuwaII-1_BFlo	gb|ABEP02003086.1|:28673-29968
	NuwaII-1_BFlo	gb|ABEP02023935.1|:190-1477
	NuwaII-1_BFlo	gb|ABEP02021560.1|:26300-27584
	NuwaII-1_BFlo	gb|ABEP02021560.1|:26300-27584
	NuwaII-1_BFlo	gb|ABEP02000520.1|:807-1069
	NuwaII-1_BFlo	gb|ABEP02041318.1|:4931-6217
	NuwaII-1_BFlo	gb|ABEP02041318.1|:4931-6217
	NuwaII-1_BFlo	gb|ABEP02028555.1|:502-764
	NuwaII-1_BFlo	gb|ABEP02026599.1|:18199-19489
	NuwaII-1_BFlo	gb|ABEP02026599.1|:18199-19489
	NuwaII-1_BFlo	gb|ABEP02020735.1|:60279-61569
	NuwaII-1_BFlo	gb|ABEP02020735.1|:60279-61569
	NuwaII-1_BFlo	gb|ABEP02013871.1|:3132-4425
	NuwaII-1_BFlo	gb|ABEP02013871.1|:3132-4425
	NuwaII-1_BFlo	gb|ABEP02011461.1|:12249-13532

NuwaII-1_BFlo	gb|ABEP02011461.1|:12249-13532
NuwaII-1_BFlo	gb|ABEP02005599.1|:22330-23622
NuwaII-1_BFlo	gb|ABEP02005599.1|:22330-23622
NuwaII-1_BFlo	gb|ABEP02016699.1|:18308-19600
NuwaII-1_BFlo	gb|ABEP02016699.1|:18308-19600
NuwaII-1_BFlo	gb|ABEP02020307.1|:29849-30913
NuwaII-1_BFlo	gb|ABEP02026300.1|:16766-18058
NuwaII-1_BFlo	gb|ABEP02026300.1|:16766-18058
NuwaII-1_BFlo	gb|ABEP02024016.1|:59802-61095
NuwaII-1_BFlo	gb|ABEP02024016.1|:59802-61095
NuwaII-1_BFlo	gb|ABEP02006147.1|:82419-83708
NuwaII-1_BFlo	gb|ABEP02006147.1|:82419-83708
NuwaII-1_BFlo	gb|ABEP02023616.1|:7797-9071
NuwaII-1_BFlo	gb|ABEP02041607.1|:546-808
NuwaII-1_BFlo	gb|ABEP02032780.1|:4413-5715
NuwaII-1_BFlo	gb|ABEP02032780.1|:4413-5715
NuwaII-1_BFlo	gb|ABEP02028778.1|:192-454
NuwaII-1_BFlo	gb|ABEP02028288.1|:298-560
NuwaII-1_BFlo	gb|ABEP02019891.1|:34745-35007
NuwaII-1_BFlo	gb|ABEP02019258.1|:9873-11164
NuwaII-1_BFlo	gb|ABEP02006483.1|:1873-3175
NuwaII-1_BFlo	gb|ABEP02004939.1|:6482-6744
NuwaII-1_BFlo	gb|ABEP02003027.1|:17566-17828
NuwaII-1_BFlo	gb|ABEP02002530.1|:32276-33578
NuwaII-1_BFlo	gb|ABEP02002530.1|:32276-33578
NuwaII-1_BFlo	gb|ABEP02018532.1|:15212-16432
NuwaII-1_BFlo	gb|ABEP02007592.1|:3576-4286
NuwaII-1_BFlo	gb|ABEP02030515.1|:5953-7260
NuwaII-1_BFlo	gb|ABEP02030515.1|:5953-7260
NuwaII-1_BFlo	gb|ABEP02025862.1|:23069-24352
NuwaII-1_BFlo	gb|ABEP02025862.1|:23069-24352
NuwaII-1_BFlo	gb|ABEP02014280.1|:20676-21965
NuwaII-1_BFlo	gb|ABEP02002647.1|:18636-19885
NuwaII-1_BFlo	gb|ABEP02036758.1|:13414-14701
NuwaII-1_BFlo	gb|ABEP02024262.1|:11141-12425
NuwaII-1_BFlo	gb|ABEP02024262.1|:11141-12425
NuwaII-1_BFlo	gb|ABEP02001922.1|:673-1963
NuwaII-1_BFlo	gb|ABEP02001922.1|:673-1963
NuwaII-1_BFlo	gb|ABEP02025452.1|:373-635
NuwaII-1_BFlo	gb|ABEP02038612.1|:10633-11357
NuwaII-1_BFlo	gb|ABEP02012641.1|:33315-33578
NuwaII-1_BFlo	gb|ABEP02031785.1|:22328-22588
NuwaII-1_BFlo	gb|ABEP02030131.1|:6150-8768
NuwaII-1_BFlo	gb|ABEP02026414.1|:26902-27079

NuwaII-1_BFlo	gb|ABEP02007590.1|:9015-9340
NuwaII-1_BFlo	gb|ABEP02027683.1|:8986-10278
NuwaII-1_BFlo	gb|ABEP02036301.1|:1-178
NuwaII-1_BFlo	gb|ABEP02006532.1|:7838-9115
NuwaII-1_BFlo	gb|ABEP02006532.1|:7838-9115
NuwaII-1_BFlo	gb|ABEP02031732.1|:14525-14781
NuwaII-1_BFlo	gb|ABEP02036745.1|:1185-2464
NuwaII-1_BFlo	gb|ABEP02024479.1|:17553-18285
NuwaII-1_BFlo	gb|ABEP02000633.1|:10717-11522
NuwaII-1_BFlo	gb|ABEP02003939.1|:391-1113
NuwaII-1_BFlo	gb|ABEP02003937.1|:9639-10304
NuwaII-1_BFlo	gb|ABEP02002021.1|:16726-17041
NuwaII-1_BFlo	gb|ABEP02036050.1|:821-2111
NuwaII-1_BFlo	gb|ABEP02036050.1|:821-2111
NuwaII-1_BFlo	gb|ABEP02003396.1|:3408-3573
NuwaII-1_BFlo	gb|ABEP02039156.1|:56671-57292
NuwaII-1_BFlo	gb|ABEP02038078.1|:1924-2127
NuwaII-1_BFlo	gb|ABEP02012536.1|:55850-56604
NuwaII-1_BFlo	gb|ABEP02008224.1|:139-342
NuwaII-1_BFlo	gb|ABEP02030303.1|:435-546
NuwaII-1_BFlo	gb|ABEP02003415.1|:3112-3315
NuwaII-1_BFlo	gb|ABEP02022957.1|:5137-5749
NuwaII-1_BFlo	gb|ABEP02040999.1|:7249-7346
NuwaII-1_BFlo	gb|ABEP02040576.1|:2867-3070
NuwaII-1_BFlo	gb|ABEP02017958.1|:17132-17277
NuwaII-1_BFlo	gb|ABEP02024998.1|:88453-88656
NuwaII-1_BFlo	gb|ABEP02022500.1|:2692-2895
NuwaII-1_BFlo	gb|ABEP02014956.1|:21471-21573
NuwaII-1_BFlo	gb|ABEP02026979.1|:7145-15716
NuwaII-1_BFlo	gb|ABEP02024407.1|:14931-15075
NuwaII-1_BFlo	gb|ABEP02021552.1|:20778-21684
NuwaII-1_BFlo	gb|ABEP02036300.1|:1124-1235
NuwaII-1_BFlo	gb|ABEP02033509.1|:119-308
NuwaII-1_BFlo	gb|ABEP02031698.1|:1221-1410
NuwaII-1_BFlo	gb|ABEP02028287.1|:2279-2390
NuwaII-1_BFlo	gb|ABEP02020509.1|:15714-16245
NuwaII-1_BFlo	gb|ABEP02004940.1|:334-445
NuwaII-1_BFlo	gb|ABEP02003029.1|:561-672
NuwaII-1_BFlo	gb|ABEP02019972.1|:5103-5198
NuwaII-1_BFlo	gb|ABEP02037654.1|:561-672
NuwaII-1_BFlo	gb|ABEP02031272.1|:1387-1456
NuwaII-1_BFlo	gb|ABEP02028554.1|:14854-14965
NuwaII-1_BFlo	gb|ABEP02023886.1|:1852-1963
NuwaII-1_BFlo	gb|ABEP02035651.1|:768-856

NuwaII-1_BFlo	gb|ABEP02030697.1|:25767-25956
NuwaII-1_BFlo	gb|ABEP02028777.1|:44263-44373
NuwaII-1_BFlo	gb|ABEP02025450.1|:18384-18495
NuwaII-1_BFlo	gb|ABEP02013484.1|:2490-3409
NuwaII-1_BFlo	gb|ABEP02002681.1|:3451-3620
NuwaII-1_BFlo	gb|ABEP02029218.1|:12847-12911
NuwaII-1_BFlo	gb|ABEP02008593.1|:25767-25907
NuwaII-1_BFlo	gb|ABEP02041283.1|:30263-31196
NuwaII-1_BFlo	gb|ABEP02040847.1|:4497-4566
NuwaII-1_BFlo	gb|ABEP02018220.1|:1-90
NuwaII-1_BFlo	gb|ABEP02038660.1|:293-481
NuwaII-1_BFlo	gb|ABEP02027587.1|:29499-29604
NuwaII-1_BFlo	gb|ABEP02000610.1|:14498-15021
NuwaII-1_BFlo	gb|ABEP02000492.1|:3832-3921
NuwaII-1_BFlo	gb|ABEP02014063.1|:2012-2069
NuwaII-1_BFlo	gb|ABEP02023239.1|:70224-70312
NuwaII-1_BFlo	gb|ABEP02017040.1|:2209-2297
NuwaII-2_BFlo	gb|ABEP02023239.1|:64454-72121
NuwaII-2_BFlo	gb|ABEP02017040.1|:397-2892
NuwaII-2_BFlo	gb|ABEP02017043.1|:1-1169
NuwaII-2_BFlo	gb|ABEP02017041.1|:4-910
NuwaII-2_BFlo	gb|ABEP02017042.1|:45-1988
NuwaII-2_BFlo	gb|ABEP02014720.1|:39757-40475
NuwaII-2_BFlo	gb|ABEP02023963.1|:4708-5498
NuwaII-2_BFlo	gb|ABEP02020527.1|:11895-12740
NuwaII-2_BFlo	gb|ABEP02009135.1|:98793-99459
NuwaII-2_BFlo	gb|ABEP02020471.1|:24484-25332
NuwaII-2_BFlo	gb|ABEP02007729.1|:32857-33696
NuwaII-2_BFlo	gb|ABEP02024645.1|:29504-30336
NuwaII-2_BFlo	gb|ABEP02021888.1|:39830-40628
NuwaII-2_BFlo	gb|ABEP02005688.1|:699-1534
NuwaII-2_BFlo	gb|ABEP02009270.1|:13745-14580
NuwaII-2_BFlo	gb|ABEP02001566.1|:729-1568
NuwaII-2_BFlo	gb|ABEP02031842.1|:4636-5469
NuwaII-2_BFlo	gb|ABEP02021884.1|:18592-19426
NuwaII-2_BFlo	gb|ABEP02004653.1|:9381-10135
NuwaII-2_BFlo	gb|ABEP02004245.1|:14565-15402
NuwaII-2_BFlo	gb|ABEP02017087.1|:2-585
NuwaII-2_BFlo	gb|ABEP02026740.1|:14794-15596
NuwaII-2_BFlo	gb|ABEP02014299.1|:31629-32454
NuwaII-2_BFlo	gb|ABEP02027227.1|:12829-13654
NuwaII-2_BFlo	gb|ABEP02040555.1|:11351-12157
NuwaII-2_BFlo	gb|ABEP02024486.1|:8784-9630
NuwaII-2_BFlo	gb|ABEP02023755.1|:64080-64921

NuwaII-2_BFlo	gb|ABEP02041633.1|:8577-9383
NuwaII-2_BFlo	gb|ABEP02005464.1|:37576-38538
NuwaII-2_BFlo	gb|ABEP02004637.1|:560-1383
NuwaII-2_BFlo	gb|ABEP02039716.1|:2301-3129
NuwaII-2_BFlo	gb|ABEP02027834.1|:13639-14467
NuwaII-2_BFlo	gb|ABEP02027834.1|:69620-69758
NuwaII-2_BFlo	gb|ABEP02040018.1|:3458-4283
NuwaII-2_BFlo	gb|ABEP02021898.1|:3984-4820
NuwaII-2_BFlo	gb|ABEP02024553.1|:2618-3441
NuwaII-2_BFlo	gb|ABEP02010397.1|:59227-60051
NuwaII-2_BFlo	gb|ABEP02003470.1|:42911-43736
NuwaII-2_BFlo	gb|ABEP02032478.1|:9067-9888
NuwaII-2_BFlo	gb|ABEP02002524.1|:6426-7257
NuwaII-2_BFlo	gb|ABEP02038003.1|:33845-34655
NuwaII-2_BFlo	gb|ABEP02032815.1|:15152-15977
NuwaII-2_BFlo	gb|ABEP02017096.1|:6888-7713
NuwaII-2_BFlo	gb|ABEP02037968.1|:7-762
NuwaII-2_BFlo	gb|ABEP02015264.1|:49773-50588
NuwaII-2_BFlo	gb|ABEP02014969.1|:33-866
NuwaII-2_BFlo	gb|ABEP02005697.1|:4060-4778
NuwaII-2_BFlo	gb|ABEP02019611.1|:3416-3830
NuwaII-2_BFlo	gb|ABEP02016344.1|:11609-12427
NuwaII-2_BFlo	gb|ABEP02000056.1|:10084-10667
NuwaII-2_BFlo	gb|ABEP02041018.1|:10730-10936
NuwaII-2_BFlo	gb|ABEP02005655.1|:8973-9761
NuwaII-2_BFlo	gb|ABEP02005090.1|:16802-17344
NuwaII-2_BFlo	gb|ABEP02005090.1|:28119-28355
NuwaII-2_BFlo	gb|ABEP02015141.1|:4198-4436
NuwaII-2_BFlo	gb|ABEP02039496.1|:9276-9537
NuwaII-2_BFlo	gb|ABEP02006438.1|:7310-7539
NuwaII-2_BFlo	gb|ABEP02026551.1|:6370-6600
NuwaII-2_BFlo	gb|ABEP02022959.1|:6290-6520
NuwaII-2_BFlo	gb|ABEP02022959.1|:23070-23197
NuwaII-2_BFlo	gb|ABEP02004961.1|:12829-13059
NuwaII-2_BFlo	gb|ABEP02004562.1|:5539-5769
NuwaII-2_BFlo	gb|ABEP02030516.1|:5920-6159
NuwaII-2_BFlo	gb|ABEP02010253.1|:12093-12332
NuwaII-2_BFlo	gb|ABEP02008881.1|:92697-92939
NuwaII-2_BFlo	gb|ABEP02009182.1|:2777-3091
NuwaII-2_BFlo	gb|ABEP02024481.1|:12523-12753
NuwaII-2_BFlo	gb|ABEP02018171.1|:13240-13470
NuwaII-2_BFlo	gb|ABEP02007497.1|:7367-7597
NuwaII-2_BFlo	gb|ABEP02027254.1|:5151-5390
NuwaII-2_BFlo	gb|ABEP02008615.1|:13675-13914

NuwaII-2_BFlo	gb|ABEP02006449.1|:14175-14408
NuwaII-2_BFlo	gb|ABEP02006449.1|:6903-7130
NuwaII-2_BFlo	gb|ABEP02040440.1|:3262-3492
NuwaII-2_BFlo	gb|ABEP02034233.1|:34831-35061
NuwaII-2_BFlo	gb|ABEP02032252.1|:18332-18562
NuwaII-2_BFlo	gb|ABEP02029676.1|:53604-53833
NuwaII-2_BFlo	gb|ABEP02029676.1|:68448-68586
NuwaII-2_BFlo	gb|ABEP02016691.1|:36019-36249
NuwaII-2_BFlo	gb|ABEP02011164.1|:14029-14259
NuwaII-2_BFlo	gb|ABEP02005338.1|:28829-28967
NuwaII-2_BFlo	gb|ABEP02005282.1|:5340-5570
NuwaII-2_BFlo	gb|ABEP02002601.1|:7140-7278
NuwaII-2_BFlo	gb|ABEP02002371.1|:42614-42752
NuwaII-2_BFlo	gb|ABEP02033495.1|:56931-57170
NuwaII-2_BFlo	gb|ABEP02013004.1|:9667-9906
NuwaII-2_BFlo	gb|ABEP02006160.1|:4055-4294
NuwaII-2_BFlo	gb|ABEP02001597.1|:34662-34901
NuwaII-2_BFlo	gb|ABEP02009505.1|:4513-4741
NuwaII-2_BFlo	gb|ABEP02029793.1|:2913-3143
NuwaII-2_BFlo	gb|ABEP02019613.1|:13690-13756
NuwaII-2_BFlo	gb|ABEP02019613.1|:3083-3221
NuwaII-2_BFlo	gb|ABEP02017630.1|:72137-72367
NuwaII-2_BFlo	gb|ABEP02017295.1|:10966-11104
NuwaII-2_BFlo	gb|ABEP02007103.1|:26197-26335
NuwaII-2_BFlo	gb|ABEP02005432.1|:48812-48905
NuwaII-2_BFlo	gb|ABEP02005432.1|:51166-51396
NuwaII-2_BFlo	gb|ABEP02005091.1|:770-1006
NuwaII-2_BFlo	gb|ABEP02000283.1|:539-761
NuwaII-2_BFlo	gb|ABEP02036397.1|:13934-14140
NuwaII-2_BFlo	gb|ABEP02036397.1|:8785-9016
NuwaII-2_BFlo	gb|ABEP02035903.1|:4897-5126
NuwaII-2_BFlo	gb|ABEP02028440.1|:48918-49157
NuwaII-2_BFlo	gb|ABEP02018885.1|:5262-5491
NuwaII-2_BFlo	gb|ABEP02035598.1|:15284-15420
NuwaII-2_BFlo	gb|ABEP02039091.1|:4300-4530
NuwaII-2_BFlo	gb|ABEP02028473.1|:16242-16380
NuwaII-2_BFlo	gb|ABEP02027684.1|:1586-1724
NuwaII-2_BFlo	gb|ABEP02023961.1|:12889-13119
NuwaII-2_BFlo	gb|ABEP02019520.1|:43512-43650
NuwaII-2_BFlo	gb|ABEP02016716.1|:2878-3016
NuwaII-2_BFlo	gb|ABEP02016506.1|:96127-96265
NuwaII-2_BFlo	gb|ABEP02015697.1|:2575-2804
NuwaII-2_BFlo	gb|ABEP02015147.1|:4549-4778
NuwaII-2_BFlo	gb|ABEP02013741.1|:34985-35123

NuwaII-2_BFlo	gb|ABEP02006453.1|:971-1200
NuwaII-2_BFlo	gb|ABEP02000203.1|:18889-19119
NuwaII-2_BFlo	gb|ABEP02040222.1|:8027-8261
NuwaII-2_BFlo	gb|ABEP02004650.1|:14996-15180
NuwaII-2_BFlo	gb|ABEP02000367.1|:21726-21866
NuwaII-2_BFlo	gb|ABEP02036051.1|:6774-6906
NuwaII-2_BFlo	gb|ABEP02000902.1|:1368-1599
NuwaII-2_BFlo	gb|ABEP02003291.1|:4618-4849
NuwaII-2_BFlo	gb|ABEP02041853.1|:16682-16820
NuwaII-2_BFlo	gb|ABEP02041853.1|:14972-15110
NuwaII-2_BFlo	gb|ABEP02041438.1|:17599-17737
NuwaII-2_BFlo	gb|ABEP02038763.1|:6813-6951
NuwaII-2_BFlo	gb|ABEP02037320.1|:7413-7551
NuwaII-2_BFlo	gb|ABEP02034230.1|:14523-14661
NuwaII-2_BFlo	gb|ABEP02033441.1|:18273-18411
NuwaII-2_BFlo	gb|ABEP02031631.1|:27617-27755
NuwaII-2_BFlo	gb|ABEP02029613.1|:83-221
NuwaII-2_BFlo	gb|ABEP02027193.1|:27247-27385
NuwaII-2_BFlo	gb|ABEP02027120.1|:34138-34276
NuwaII-2_BFlo	gb|ABEP02025860.1|:3145-3283
NuwaII-2_BFlo	gb|ABEP02025418.1|:49599-49737
NuwaII-2_BFlo	gb|ABEP02024894.1|:10432-10570
NuwaII-2_BFlo	gb|ABEP02024027.1|:5311-5449
NuwaII-2_BFlo	gb|ABEP02023789.1|:14792-14930
NuwaII-2_BFlo	gb|ABEP02023148.1|:27145-27283
NuwaII-2_BFlo	gb|ABEP02017660.1|:58123-58261
NuwaII-2_BFlo	gb|ABEP02015701.1|:2877-3015
NuwaII-2_BFlo	gb|ABEP02015051.1|:1043-1181
NuwaII-2_BFlo	gb|ABEP02014190.1|:6277-6415
NuwaII-2_BFlo	gb|ABEP02013215.1|:1303-1441
NuwaII-2_BFlo	gb|ABEP02012407.1|:13429-13659
NuwaII-2_BFlo	gb|ABEP02009457.1|:14584-14722
NuwaII-2_BFlo	gb|ABEP02006959.1|:6239-6377
NuwaII-2_BFlo	gb|ABEP02006817.1|:9065-9203
NuwaII-2_BFlo	gb|ABEP02006816.1|:4336-4572
NuwaII-2_BFlo	gb|ABEP02006816.1|:6326-6464
NuwaII-2_BFlo	gb|ABEP02006422.1|:96682-96820
NuwaII-2_BFlo	gb|ABEP02005926.1|:2050-2187
NuwaII-2_BFlo	gb|ABEP02005926.1|:30515-30653
NuwaII-2_BFlo	gb|ABEP02005573.1|:25735-25873
NuwaII-2_BFlo	gb|ABEP02005232.1|:12541-12679
NuwaII-2_BFlo	gb|ABEP02005230.1|:27247-27385
NuwaII-2_BFlo	gb|ABEP02004044.1|:28481-28619
NuwaII-2_BFlo	gb|ABEP02002626.1|:4732-4870

NuwaII-2_BFlo	gb|ABEP02002430.1|:9271-9409
NuwaII-2_BFlo	gb|ABEP02001567.1|:5933-6071
NuwaII-2_BFlo	gb|ABEP02034936.1|:3906-4135
NuwaII-2_BFlo	gb|ABEP02012096.1|:104172-104401
NuwaII-2_BFlo	gb|ABEP02012096.1|:76214-76349
NuwaII-2_BFlo	gb|ABEP02036705.1|:1360-1597
NuwaII-2_BFlo	gb|ABEP02015134.1|:21916-22147
NuwaII-2_BFlo	gb|ABEP02040910.1|:2374-2512
NuwaII-2_BFlo	gb|ABEP02040300.1|:4238-4382
NuwaII-2_BFlo	gb|ABEP02039075.1|:36210-36348
NuwaII-2_BFlo	gb|ABEP02039075.1|:64815-64947
NuwaII-2_BFlo	gb|ABEP02036597.1|:3618-3756
NuwaII-2_BFlo	gb|ABEP02035789.1|:6768-6906
NuwaII-2_BFlo	gb|ABEP02032469.1|:1926-2063
NuwaII-2_BFlo	gb|ABEP02024081.1|:23367-23505
NuwaII-2_BFlo	gb|ABEP02020313.1|:6739-6877
NuwaII-2_BFlo	gb|ABEP02014330.1|:12561-12699
NuwaII-2_BFlo	gb|ABEP02014188.1|:716-854
NuwaII-2_BFlo	gb|ABEP02012175.1|:1238-1376
NuwaII-2_BFlo	gb|ABEP02002232.1|:17154-17292
NuwaII-2_BFlo	gb|ABEP02001425.1|:16811-16949
NuwaII-2_BFlo	gb|ABEP02011461.1|:15236-15470
NuwaII-2_BFlo	gb|ABEP02026919.1|:14527-14663
NuwaII-2_BFlo	gb|ABEP02020266.1|:2291-2524
NuwaII-2_BFlo	gb|ABEP02005241.1|:19167-19395
NuwaII-2_BFlo	gb|ABEP02039257.1|:6136-6274
NuwaII-2_BFlo	gb|ABEP02036854.1|:15874-16012
NuwaII-2_BFlo	gb|ABEP02034254.1|:2554-2692
NuwaII-2_BFlo	gb|ABEP02033447.1|:28418-28544
NuwaII-2_BFlo	gb|ABEP02026489.1|:5963-6194
NuwaII-2_BFlo	gb|ABEP02024936.1|:708-846
NuwaII-2_BFlo	gb|ABEP02024597.1|:58550-58779
NuwaII-2_BFlo	gb|ABEP02016850.1|:10170-10308
NuwaII-2_BFlo	gb|ABEP02010430.1|:18714-18852
NuwaII-2_BFlo	gb|ABEP02003777.1|:5458-5596
NuwaII-2_BFlo	gb|ABEP02034568.1|:3363-3602
NuwaII-2_BFlo	gb|ABEP02023927.1|:11997-12134
NuwaII-2_BFlo	gb|ABEP02022359.1|:4119-4253
NuwaII-2_BFlo	gb|ABEP02016212.1|:276-508
NuwaII-2_BFlo	gb|ABEP02013096.1|:23825-24070
NuwaII-2_BFlo	gb|ABEP02001616.1|:936-1078
NuwaII-2_BFlo	gb|ABEP02022441.1|:45061-45197
NuwaII-2_BFlo	gb|ABEP02038967.1|:4307-4446
NuwaII-2_BFlo	gb|ABEP02016582.1|:21794-22024

NuwaII-2_BFlo	gb|ABEP02015981.1|:14735-14961
NuwaII-2_BFlo	gb|ABEP02038543.1|:988-1126
NuwaII-2_BFlo	gb|ABEP02037049.1|:487-625
NuwaII-2_BFlo	gb|ABEP02023577.1|:32805-32942
NuwaII-2_BFlo	gb|ABEP02023568.1|:4327-4559
NuwaII-2_BFlo	gb|ABEP02017136.1|:22952-23090
NuwaII-2_BFlo	gb|ABEP02005605.1|:630-860
NuwaII-2_BFlo	gb|ABEP02019240.1|:237-350
NuwaII-2_BFlo	gb|ABEP02026411.1|:22797-23025
NuwaII-2_BFlo	gb|ABEP02029121.1|:21003-21227
NuwaII-2_BFlo	gb|ABEP02016227.1|:923-1167
NuwaII-2_BFlo	gb|ABEP02038839.1|:1832-1969
NuwaII-2_BFlo	gb|ABEP02032172.1|:4230-4368
NuwaII-2_BFlo	gb|ABEP02025668.1|:27165-27302
NuwaII-2_BFlo	gb|ABEP02009977.1|:786-924
NuwaII-2_BFlo	gb|ABEP02007676.1|:664-790
NuwaII-2_BFlo	gb|ABEP02003593.1|:7574-7712
NuwaII-2_BFlo	gb|ABEP02000030.1|:70586-70723
NuwaII-2_BFlo	gb|ABEP02021594.1|:12078-12263
NuwaII-2_BFlo	gb|ABEP02016543.1|:955-1087
NuwaII-2_BFlo	gb|ABEP02008751.1|:31697-31822
NuwaII-2_BFlo	gb|ABEP02032159.1|:10753-10889
NuwaII-2_BFlo	gb|ABEP02029713.1|:9754-9878
NuwaII-2_BFlo	gb|ABEP02027371.1|:4443-4579
NuwaII-2_BFlo	gb|ABEP02024901.1|:5617-5844
NuwaII-2_BFlo	gb|ABEP02009352.1|:15041-15177
NuwaII-2_BFlo	gb|ABEP02005210.1|:14402-14538
NuwaII-2_BFlo	gb|ABEP02001502.1|:13515-13651
NuwaII-2_BFlo	gb|ABEP02021100.1|:1570-1707
NuwaII-2_BFlo	gb|ABEP02000015.1|:42829-42964
NuwaII-2_BFlo	gb|ABEP02037048.1|:3907-4044
NuwaII-2_BFlo	gb|ABEP02025821.1|:18-152
NuwaII-2_BFlo	gb|ABEP02023693.1|:2745-2980
NuwaII-2_BFlo	gb|ABEP02022963.1|:33459-33597
NuwaII-2_BFlo	gb|ABEP02014819.1|:10516-10654
NuwaII-2_BFlo	gb|ABEP02009476.1|:4207-4345
NuwaII-2_BFlo	gb|ABEP02004092.1|:3000-3206
NuwaII-2_BFlo	gb|ABEP02002845.1|:32788-32926
NuwaII-2_BFlo	gb|ABEP02009446.1|:4643-4787
NuwaII-2_BFlo	gb|ABEP02029234.1|:7604-7740
NuwaII-2_BFlo	gb|ABEP02020789.1|:4252-4388
NuwaII-2_BFlo	gb|ABEP02012086.1|:2281-2413
NuwaII-2_BFlo	gb|ABEP02011581.1|:27941-28076
NuwaII-2_BFlo	gb|ABEP02040672.1|:3698-3785

	NuwaII-2_BFlo	gb|ABEP02040672.1|:3331-3470
	NuwaII-2_BFlo	gb|ABEP02030142.1|:19473-19608
	NuwaII-2_BFlo	gb|ABEP02023178.1|:8418-8550
	NuwaII-2_BFlo	gb|ABEP02005580.1|:160404-160543
	NuwaII-2_BFlo	gb|ABEP02040425.1|:7752-7890
	NuwaII-2_BFlo	gb|ABEP02031502.1|:4477-4615
	NuwaII-2_BFlo	gb|ABEP02031025.1|:25406-25502
	NuwaII-2_BFlo	gb|ABEP02031025.1|:21242-21348
	NuwaII-2_BFlo	gb|ABEP02006440.1|:10508-10646
	NuwaII-2_BFlo	gb|ABEP02021147.1|:419680-419814
	NuwaII-2_BFlo	gb|ABEP02021147.1|:232761-232990
	NuwaII-2_BFlo	gb|ABEP02008895.1|:12331-12560
	NuwaII-2_BFlo	gb|ABEP02008895.1|:19428-19556
	NuwaII-2_BFlo	gb|ABEP02041090.1|:2393-2529
	NuwaII-2_BFlo	gb|ABEP02035779.1|:9509-9645
	NuwaII-2_BFlo	gb|ABEP02029368.1|:5360-5488
	NuwaII-2_BFlo	gb|ABEP02025401.1|:16668-16804
	NuwaII-2_BFlo	gb|ABEP02019153.1|:38814-38949
	NuwaII-2_BFlo	gb|ABEP02013795.1|:35488-35624
	NuwaII-2_BFlo	gb|ABEP02012694.1|:3704-3832
Chrysemys picta bellii	*NuwaII-1_CPB*	gb|AHGY02069326.1|:13805-13979
	NuwaII-1_CPB	gb|AHGY02069326.1|:26679-28852
	NuwaII-1_CPB	gb|AHGY02069326.1|:347-511
	NuwaII-1_CPB	gb|AHGY02022687.1|:3818-5844
	NuwaII-1_CPB	gb|AHGY02022687.1|:14806-18921
	NuwaII-1_CPB	gb|AHGY02106282.1|:1176-3082
	NuwaII-1_CPB	gb|AHGY02168012.1|:33-1891
	NuwaII-1_CPB	gb|AHGY02093383.1|:13703-16670
	NuwaII-1_CPB	gb|AHGY02078411.1|:2458-6049
	NuwaII-1_CPB	gb|AHGY02224275.1|:6940-11244
	NuwaII-1_CPB	gb|AHGY02005108.1|:49-2003
	NuwaII-1_CPB	gb|AHGY02068534.1|:979-5276
	NuwaII-1_CPB	gb|AHGY02121678.1|:32-2047
	NuwaII-1_CPB	gb|AHGY02261766.1|:4403-4507
	NuwaII-1_CPB	gb|AHGY02261766.1|:41925-42035
	NuwaII-1_CPB	gb|AHGY02261766.1|:41879-41983
	NuwaII-1_CPB	gb|AHGY02261766.1|:22772-27038
	NuwaII-1_CPB	gb|AHGY02261766.1|:4360-4501
	NuwaII-1_CPB	gb|AHGY02045820.1|:32-1995
	NuwaII-1_CPB	gb|AHGY02248773.1|:62-1869
	NuwaII-1_CPB	gb|AHGY02257336.1|:3841-8084
	NuwaII-1_CPB	gb|AHGY02257336.1|:30006-30059
	NuwaII-1_CPB	gb|AHGY02257336.1|:17746-17852
	NuwaII-1_CPB	gb|AHGY02048211.1|:1-1686

NuwaII-1_CPB	gb|AHGY02046857.1|:16043-18077
NuwaII-1_CPB	gb|AHGY02046857.1|:8086-9230
NuwaII-1_CPB	gb|AHGY02240340.1|:1-2758
NuwaII-1_CPB	gb|AHGY02197365.1|:1-2846
NuwaII-1_CPB	gb|AHGY02023669.1|:16665-20235
NuwaII-1_CPB	gb|AHGY02247874.1|:1-1858
NuwaII-1_CPB	gb|AHGY02209249.1|:21716-25979
NuwaII-1_CPB	gb|AHGY02209249.1|:42559-42649
NuwaII-1_CPB	gb|AHGY02033209.1|:10753-15033
NuwaII-1_CPB	gb|AHGY02203774.1|:1-3018
NuwaII-1_CPB	gb|AHGY02091554.1|:8411-10814
NuwaII-1_CPB	gb|AHGY02259948.1|:3136-7386
NuwaII-1_CPB	gb|AHGY02259948.1|:11390-11494
NuwaII-1_CPB	gb|AHGY02107096.1|:1-2196
NuwaII-1_CPB	gb|AHGY02260055.1|:68658-68782
NuwaII-1_CPB	gb|AHGY02260055.1|:58911-63279
NuwaII-1_CPB	gb|AHGY02260055.1|:43915-44038
NuwaII-1_CPB	gb|AHGY02260055.1|:8321-8484
NuwaII-1_CPB	gb|AHGY02213674.1|:12578-12890
NuwaII-1_CPB	gb|AHGY02213674.1|:1-1832
NuwaII-1_CPB	gb|AHGY02212112.1|:26822-28645
NuwaII-1_CPB	gb|AHGY02259587.1|:3850-3977
NuwaII-1_CPB	gb|AHGY02259587.1|:5-3024
NuwaII-1_CPB	gb|AHGY02201910.1|:9206-13493
NuwaII-1_CPB	gb|AHGY02016084.1|:3903-4033
NuwaII-1_CPB	gb|AHGY02016084.1|:9270-13601
NuwaII-1_CPB	gb|AHGY02133661.1|:16-1588
NuwaII-1_CPB	gb|AHGY02228717.1|:9909-11800
NuwaII-1_CPB	gb|AHGY02228717.1|:22-79
NuwaII-1_CPB	gb|AHGY02244988.1|:9-1955
NuwaII-1_CPB	gb|AHGY02200515.1|:3177-3353
NuwaII-1_CPB	gb|AHGY02200515.1|:22245-22326
NuwaII-1_CPB	gb|AHGY02200515.1|:22196-22303
NuwaII-1_CPB	gb|AHGY02200515.1|:4540-8907
NuwaII-1_CPB	gb|AHGY02216237.1|:13-2146
NuwaII-1_CPB	gb|AHGY02051070.1|:1-2125
NuwaII-1_CPB	gb|AHGY02233368.1|:3383-6252
NuwaII-1_CPB	gb|AHGY02078242.1|:36099-36231
NuwaII-1_CPB	gb|AHGY02078242.1|:57885-57938
NuwaII-1_CPB	gb|AHGY02078242.1|:83553-83631
NuwaII-1_CPB	gb|AHGY02078242.1|:103332-106186
NuwaII-1_CPB	gb|AHGY02078242.1|:83507-83611
NuwaII-1_CPB	gb|AHGY02078242.1|:67916-68089
NuwaII-1_CPB	gb|AHGY02078242.1|:55575-55625

NuwaII-1_CPB	gb|AHGY02078242.1|:36071-36231
NuwaII-1_CPB	gb|AHGY02078242.1|:19078-19190
NuwaII-1_CPB	gb|AHGY02244552.1|:19047-21064
NuwaII-1_CPB	gb|AHGY02244552.1|:7189-7317
NuwaII-1_CPB	gb|AHGY02188066.1|:1-1783
NuwaII-1_CPB	gb|AHGY02040509.1|:11-3108
NuwaII-1_CPB	gb|AHGY02038509.1|:30778-33095
NuwaII-1_CPB	gb|AHGY02018612.1|:1-1835
NuwaII-1_CPB	gb|AHGY02224864.1|:3039-7318
NuwaII-1_CPB	gb|AHGY02224864.1|:1-74
NuwaII-1_CPB	gb|AHGY02007392.1|:1-1788
NuwaII-1_CPB	gb|AHGY02216291.1|:24088-28370
NuwaII-1_CPB	gb|AHGY02060100.1|:7468-9710
NuwaII-1_CPB	gb|AHGY02038035.1|:69213-73480
NuwaII-1_CPB	gb|AHGY02038035.1|:80661-80710
NuwaII-1_CPB	gb|AHGY02038035.1|:65111-65168
NuwaII-1_CPB	gb|AHGY02250035.1|:26331-29439
NuwaII-1_CPB	gb|AHGY02250035.1|:19314-24986
NuwaII-1_CPB	gb|AHGY02068690.1|:10300-14652
NuwaII-1_CPB	gb|AHGY02086772.1|:3205-3261
NuwaII-1_CPB	gb|AHGY02086772.1|:1-3073
NuwaII-1_CPB	gb|AHGY02059235.1|:21930-24774
NuwaII-1_CPB	gb|AHGY02091307.1|:5-1209
NuwaII-1_CPB	gb|AHGY02091307.1|:27514-31728
NuwaII-1_CPB	gb|AHGY02091307.1|:42395-42594
NuwaII-1_CPB	gb|AHGY02091307.1|:42389-42555
NuwaII-1_CPB	gb|AHGY02252936.1|:897-5146
NuwaII-1_CPB	gb|AHGY02213973.1|:153-4403
NuwaII-1_CPB	gb|AHGY02114207.1|:1280-4033
NuwaII-1_CPB	gb|AHGY02009355.1|:5-2503
NuwaII-1_CPB	gb|AHGY02031832.1|:23792-23865
NuwaII-1_CPB	gb|AHGY02031832.1|:36973-41327
NuwaII-1_CPB	gb|AHGY02216594.1|:2128-2192
NuwaII-1_CPB	gb|AHGY02216594.1|:12641-12815
NuwaII-1_CPB	gb|AHGY02216594.1|:24-2086
NuwaII-1_CPB	gb|AHGY02229652.1|:6278-10526
NuwaII-1_CPB	gb|AHGY02229652.1|:33994-34115
NuwaII-1_CPB	gb|AHGY02229652.1|:19653-19728
NuwaII-1_CPB	gb|AHGY02229652.1|:1963-2052
NuwaII-1_CPB	gb|AHGY02184721.1|:25-1845
NuwaII-1_CPB	gb|AHGY02028455.1|:23961-28132
NuwaII-1_CPB	gb|AHGY02196083.1|:6818-7016
NuwaII-1_CPB	gb|AHGY02196083.1|:19703-19784
NuwaII-1_CPB	gb|AHGY02196083.1|:1-3339

NuwaII-1_CPB	gb|AHGY02019508.1|:101-2056
NuwaII-1_CPB	gb|AHGY02222592.1|:9409-19929
NuwaII-1_CPB	gb|AHGY02222592.1|:42264-42376
NuwaII-1_CPB	gb|AHGY02222592.1|:45731-48019
NuwaII-1_CPB	gb|AHGY02222592.1|:21261-21452
NuwaII-1_CPB	gb|AHGY02090559.1|:26973-30847
NuwaII-1_CPB	gb|AHGY02067123.1|:3485-5437
NuwaII-1_CPB	gb|AHGY02067123.1|:2757-3070
NuwaII-1_CPB	gb|AHGY02225752.1|:5273-8086
NuwaII-1_CPB	gb|AHGY02070395.1|:1-2378
NuwaII-1_CPB	gb|AHGY02035013.1|:22206-22270
NuwaII-1_CPB	gb|AHGY02035013.1|:81397-81453
NuwaII-1_CPB	gb|AHGY02035013.1|:93042-93109
NuwaII-1_CPB	gb|AHGY02035013.1|:87656-87725
NuwaII-1_CPB	gb|AHGY02035013.1|:44126-46984
NuwaII-1_CPB	gb|AHGY02211006.1|:32-3618
NuwaII-1_CPB	gb|AHGY02084662.1|:8688-12426
NuwaII-1_CPB	gb|AHGY02059063.1|:12406-16650
NuwaII-1_CPB	gb|AHGY02224675.1|:43163-47452
NuwaII-1_CPB	gb|AHGY02224675.1|:126178-126271
NuwaII-1_CPB	gb|AHGY02224675.1|:126129-126236
NuwaII-1_CPB	gb|AHGY02224675.1|:684-4020
NuwaII-1_CPB	gb|AHGY02089064.1|:25335-27470
NuwaII-1_CPB	gb|AHGY02089064.1|:21278-21410
NuwaII-1_CPB	gb|AHGY02000240.1|:10-3124
NuwaII-1_CPB	gb|AHGY02241538.1|:12112-15044
NuwaII-1_CPB	gb|AHGY02198438.1|:1-2650
NuwaII-1_CPB	gb|AHGY02013138.1|:17183-21361
NuwaII-1_CPB	gb|AHGY02245719.1|:1-3912
NuwaII-1_CPB	gb|AHGY02235599.1|:2561-2665
NuwaII-1_CPB	gb|AHGY02235599.1|:19375-23629
NuwaII-1_CPB	gb|AHGY02235599.1|:2536-2619
NuwaII-1_CPB	gb|AHGY02214474.1|:8725-10741
NuwaII-1_CPB	gb|AHGY02206923.1|:8063-10068
NuwaII-1_CPB	gb|AHGY02068362.1|:63-435
NuwaII-1_CPB	gb|AHGY02068362.1|:12626-13094
NuwaII-1_CPB	gb|AHGY02068362.1|:23701-27666
NuwaII-1_CPB	gb|AHGY02068362.1|:63-407
NuwaII-1_CPB	gb|AHGY02063001.1|:2741-6958
NuwaII-1_CPB	gb|AHGY02057344.1|:16399-19374
NuwaII-1_CPB	gb|AHGY02043597.1|:506-11421
NuwaII-1_CPB	gb|AHGY02078052.1|:11625-15854
NuwaII-1_CPB	gb|AHGY02078052.1|:16598-16681
NuwaII-1_CPB	gb|AHGY02027409.1|:1-1670

NuwaII-1_CPB	gb|AHGY02051368.1|:17-3491
NuwaII-1_CPB	gb|AHGY02234716.1|:17601-20238
NuwaII-1_CPB	gb|AHGY02227475.1|:34933-38437
NuwaII-1_CPB	gb|AHGY02221948.1|:10708-10795
NuwaII-1_CPB	gb|AHGY02221948.1|:22592-24467
NuwaII-1_CPB	gb|AHGY02221948.1|:10695-10766
NuwaII-1_CPB	gb|AHGY02214758.1|:13845-17542
NuwaII-1_CPB	gb|AHGY02214758.1|:5389-6189
NuwaII-1_CPB	gb|AHGY02214148.1|:238-340
NuwaII-1_CPB	gb|AHGY02214148.1|:20774-20883
NuwaII-1_CPB	gb|AHGY02214148.1|:22219-22392
NuwaII-1_CPB	gb|AHGY02214148.1|:60-11566
NuwaII-1_CPB	gb|AHGY02245566.1|:5859-5939
NuwaII-1_CPB	gb|AHGY02245566.1|:45510-47847
NuwaII-1_CPB	gb|AHGY02245566.1|:5847-5917
NuwaII-1_CPB	gb|AHGY02061654.1|:5462-9741
NuwaII-1_CPB	gb|AHGY02061654.1|:3252-3353
NuwaII-1_CPB	gb|AHGY02220509.1|:3-4098
NuwaII-1_CPB	gb|AHGY02004209.1|:6920-7027
NuwaII-1_CPB	gb|AHGY02004209.1|:1-2932
NuwaII-1_CPB	gb|AHGY02207468.1|:8102-16595
NuwaII-1_CPB	gb|AHGY02207468.1|:10377-14657
NuwaII-1_CPB	gb|AHGY02092910.1|:1923-6992
NuwaII-1_CPB	gb|AHGY02092910.1|:11062-15290
NuwaII-1_CPB	gb|AHGY02083276.1|:2111-2198
NuwaII-1_CPB	gb|AHGY02083276.1|:16600-16679
NuwaII-1_CPB	gb|AHGY02083276.1|:2928-7169
NuwaII-1_CPB	gb|AHGY02047457.1|:5-1842
NuwaII-1_CPB	gb|AHGY02115226.1|:52-2478
NuwaII-1_CPB	gb|AHGY02111306.1|:6159-8050
NuwaII-1_CPB	gb|AHGY02111306.1|:3897-4024
NuwaII-1_CPB	gb|AHGY02086800.1|:30946-31105
NuwaII-1_CPB	gb|AHGY02086800.1|:30940-31099
NuwaII-1_CPB	gb|AHGY02086800.1|:4-2861
NuwaII-1_CPB	gb|AHGY02081377.1|:1-4109
NuwaII-1_CPB	gb|AHGY02071616.1|:7328-11722
NuwaII-1_CPB	gb|AHGY02246178.1|:87657-89378
NuwaII-1_CPB	gb|AHGY02246178.1|:116647-119501
NuwaII-1_CPB	gb|AHGY02246178.1|:116579-116646
NuwaII-1_CPB	gb|AHGY02246178.1|:89267-89339
NuwaII-1_CPB	gb|AHGY02060981.1|:18340-18443
NuwaII-1_CPB	gb|AHGY02060981.1|:31851-31979
NuwaII-1_CPB	gb|AHGY02060981.1|:23525-27873
NuwaII-1_CPB	gb|AHGY02060981.1|:2952-3051

NuwaII-1_CPB	gb|AHGY02056999.1|:2013-5419
NuwaII-1_CPB	gb|AHGY02233175.1|:1616-3311
NuwaII-1_CPB	gb|AHGY02089592.1|:483-3652
NuwaII-1_CPB	gb|AHGY02095516.1|:4927-7996
NuwaII-1_CPB	gb|AHGY02060561.1|:13199-17443
NuwaII-1_CPB	gb|AHGY02247599.1|:299-1991
NuwaII-1_CPB	gb|AHGY02247599.1|:3187-7294
NuwaII-1_CPB	gb|AHGY02032387.1|:36341-41136
NuwaII-1_CPB	gb|AHGY02032387.1|:3865-3927
NuwaII-1_CPB	gb|AHGY02261568.1|:5-112
NuwaII-1_CPB	gb|AHGY02261568.1|:53959-58658
NuwaII-1_CPB	gb|AHGY02261568.1|:68885-68978
NuwaII-1_CPB	gb|AHGY02261568.1|:71111-75068
NuwaII-1_CPB	gb|AHGY02261568.1|:49101-49334
NuwaII-1_CPB	gb|AHGY02261568.1|:2-63
NuwaII-1_CPB	gb|AHGY02239339.1|:23526-25649
NuwaII-1_CPB	gb|AHGY02218990.1|:1-2926
NuwaII-1_CPB	gb|AHGY02000371.1|:8218-10233
NuwaII-1_CPB	gb|AHGY02000371.1|:3940-4003
NuwaII-1_CPB	gb|AHGY02248671.1|:3503-3606
NuwaII-1_CPB	gb|AHGY02248671.1|:62-2602
NuwaII-1_CPB	gb|AHGY02225945.1|:6914-9295
NuwaII-1_CPB	gb|AHGY02063764.1|:4368-6984
NuwaII-1_CPB	gb|AHGY02238205.1|:10461-14615
NuwaII-1_CPB	gb|AHGY02066120.1|:14081-16295
NuwaII-1_CPB	gb|AHGY02034939.1|:1-2852
NuwaII-1_CPB	gb|AHGY02252200.1|:1-1793
NuwaII-1_CPB	gb|AHGY02216516.1|:1-2349
NuwaII-1_CPB	gb|AHGY02051488.1|:23770-27251
NuwaII-1_CPB	gb|AHGY02051488.1|:29128-29230
NuwaII-1_CPB	gb|AHGY02051488.1|:50-3291
NuwaII-1_CPB	gb|AHGY02217205.1|:3631-10829
NuwaII-1_CPB	gb|AHGY02217205.1|:10704-10813
NuwaII-1_CPB	gb|AHGY02217205.1|:1-51
NuwaII-1_CPB	gb|AHGY02028632.1|:16190-16259
NuwaII-1_CPB	gb|AHGY02028632.1|:22-2435
NuwaII-1_CPB	gb|AHGY02009988.1|:19726-24003
NuwaII-1_CPB	gb|AHGY02009988.1|:26090-27606
NuwaII-1_CPB	gb|AHGY02009988.1|:7167-7261
NuwaII-1_CPB	gb|AHGY02225872.1|:10891-13525
NuwaII-1_CPB	gb|AHGY02225872.1|:354-415
NuwaII-1_CPB	gb|AHGY02118303.1|:2336-5647
NuwaII-1_CPB	gb|AHGY02059078.1|:2640-6137
NuwaII-1_CPB	gb|AHGY02029330.1|:20924-21046

NuwaII-1_CPB	gb|AHGY02029330.1|:41044-41135
NuwaII-1_CPB	gb|AHGY02029330.1|:64-2410
NuwaII-1_CPB	gb|AHGY02223513.1|:41-2714
NuwaII-1_CPB	gb|AHGY02191504.1|:2-2344
NuwaII-1_CPB	gb|AHGY02081156.1|:2165-5180
NuwaII-1_CPB	gb|AHGY02136695.1|:4-1360
NuwaII-1_CPB	gb|AHGY02106031.1|:1-1661
NuwaII-1_CPB	gb|AHGY02254732.1|:11672-14419
NuwaII-1_CPB	gb|AHGY02027290.1|:5857-10124
NuwaII-1_CPB	gb|AHGY02227596.1|:3035-7242
NuwaII-1_CPB	gb|AHGY02227596.1|:1-85
NuwaII-1_CPB	gb|AHGY02021084.1|:906-2735
NuwaII-1_CPB	gb|AHGY02080969.1|:12-3032
NuwaII-1_CPB	gb|AHGY02252834.1|:54232-54320
NuwaII-1_CPB	gb|AHGY02252834.1|:878-3994
NuwaII-1_CPB	gb|AHGY02237777.1|:6120-8289
NuwaII-1_CPB	gb|AHGY02098892.1|:1673-5497
NuwaII-1_CPB	gb|AHGY02098892.1|:342-467
NuwaII-1_CPB	gb|AHGY02096159.1|:2168-4468
NuwaII-1_CPB	gb|AHGY02035302.1|:4195-7954
NuwaII-1_CPB	gb|AHGY02135020.1|:32-1402
NuwaII-1_CPB	gb|AHGY02034655.1|:27638-27719
NuwaII-1_CPB	gb|AHGY02034655.1|:15722-19123
NuwaII-1_CPB	gb|AHGY02015990.1|:5079-5133
NuwaII-1_CPB	gb|AHGY02015990.1|:348-2769
NuwaII-1_CPB	gb|AHGY02244204.1|:2812-7065
NuwaII-1_CPB	gb|AHGY02220991.1|:11732-11793
NuwaII-1_CPB	gb|AHGY02220991.1|:5373-9649
NuwaII-1_CPB	gb|AHGY02050904.1|:52995-53124
NuwaII-1_CPB	gb|AHGY02050904.1|:36712-36809
NuwaII-1_CPB	gb|AHGY02050904.1|:9026-13343
NuwaII-1_CPB	gb|AHGY02231058.1|:6772-9416
NuwaII-1_CPB	gb|AHGY02198759.1|:28-2582
NuwaII-1_CPB	gb|AHGY02053493.1|:27-2370
NuwaII-1_CPB	gb|AHGY02071282.1|:531-3410
NuwaII-1_CPB	gb|AHGY02222055.1|:28474-28568
NuwaII-1_CPB	gb|AHGY02222055.1|:38760-38821
NuwaII-1_CPB	gb|AHGY02222055.1|:38749-38818
NuwaII-1_CPB	gb|AHGY02222055.1|:23156-27303
NuwaII-1_CPB	gb|AHGY02201719.1|:12495-12552
NuwaII-1_CPB	gb|AHGY02201719.1|:13809-17898
NuwaII-1_CPB	gb|AHGY02058661.1|:14-2846
NuwaII-1_CPB	gb|AHGY02055260.1|:1-2223
NuwaII-1_CPB	gb|AHGY02052592.1|:16265-20565

NuwaII-1_CPB	gb|AHGY02248835.1|:7870-10513
NuwaII-1_CPB	gb|AHGY02211461.1|:2032-6367
NuwaII-1_CPB	gb|AHGY02195294.1|:17-1967
NuwaII-1_CPB	gb|AHGY02031200.1|:17073-20808
NuwaII-1_CPB	gb|AHGY02216700.1|:589-1861
NuwaII-1_CPB	gb|AHGY02216700.1|:15228-15384
NuwaII-1_CPB	gb|AHGY02216700.1|:27985-32174
NuwaII-1_CPB	gb|AHGY02216700.1|:15182-15370
NuwaII-1_CPB	gb|AHGY02216700.1|:546-1859
NuwaII-1_CPB	gb|AHGY02078256.1|:6364-6448
NuwaII-1_CPB	gb|AHGY02078256.1|:1-1800
NuwaII-1_CPB	gb|AHGY02111195.1|:1-2069
NuwaII-1_CPB	gb|AHGY02025132.1|:1-3005
NuwaII-1_CPB	gb|AHGY02029420.1|:19698-20762
NuwaII-1_CPB	gb|AHGY02029420.1|:10525-14827
NuwaII-1_CPB	gb|AHGY02238736.1|:13289-24418
NuwaII-1_CPB	gb|AHGY02238736.1|:24218-24369
NuwaII-1_CPB	gb|AHGY02226590.1|:1948-2018
NuwaII-1_CPB	gb|AHGY02226590.1|:44384-46720
NuwaII-1_CPB	gb|AHGY02226590.1|:37573-37636
NuwaII-1_CPB	gb|AHGY02226590.1|:1880-2006
NuwaII-1_CPB	gb|AHGY02221836.1|:8-2173
NuwaII-1_CPB	gb|AHGY02101302.1|:11924-14056
NuwaII-1_CPB	gb|AHGY02061456.1|:8622-12731
NuwaII-1_CPB	gb|AHGY02016316.1|:6902-10933
NuwaII-1_CPB	gb|AHGY02016316.1|:15185-15270
NuwaII-1_CPB	gb|AHGY02258243.1|:13632-17881
NuwaII-1_CPB	gb|AHGY02258243.1|:27754-27814
NuwaII-1_CPB	gb|AHGY02113302.1|:10729-14934
NuwaII-1_CPB	gb|AHGY02113302.1|:10533-10626
NuwaII-1_CPB	gb|AHGY02247059.1|:4709-4877
NuwaII-1_CPB	gb|AHGY02247059.1|:27810-27864
NuwaII-1_CPB	gb|AHGY02247059.1|:57369-57435
NuwaII-1_CPB	gb|AHGY02247059.1|:57323-57427
NuwaII-1_CPB	gb|AHGY02247059.1|:25078-29516
NuwaII-1_CPB	gb|AHGY02247059.1|:4667-4871
NuwaII-1_CPB	gb|AHGY02172462.1|:26-2652
NuwaII-1_CPB	gb|AHGY02251256.1|:22052-22180
NuwaII-1_CPB	gb|AHGY02251256.1|:16780-21058
NuwaII-1_CPB	gb|AHGY02253757.1|:1136-3362
NuwaII-1_CPB	gb|AHGY02253757.1|:34-83
NuwaII-1_CPB	gb|AHGY02241992.1|:2496-4314
NuwaII-1_CPB	gb|AHGY02241992.1|:1-118
NuwaII-1_CPB	gb|AHGY02206144.1|:1322-1425

NuwaII-1_CPB	gb|AHGY02206144.1|:45280-49497
NuwaII-1_CPB	gb|AHGY02206144.1|:20230-20333
NuwaII-1_CPB	gb|AHGY02206144.1|:2767-3957
NuwaII-1_CPB	gb|AHGY02200636.1|:212-274
NuwaII-1_CPB	gb|AHGY02200636.1|:18617-18685
NuwaII-1_CPB	gb|AHGY02200636.1|:8710-13405
NuwaII-1_CPB	gb|AHGY02078285.1|:1-4074
NuwaII-1_CPB	gb|AHGY02076616.1|:5097-9023
NuwaII-1_CPB	gb|AHGY02074831.1|:4328-8612
NuwaII-1_CPB	gb|AHGY02243422.1|:293-548
NuwaII-1_CPB	gb|AHGY02243422.1|:1179-5237
NuwaII-1_CPB	gb|AHGY02235706.1|:4783-7899
NuwaII-1_CPB	gb|AHGY02235706.1|:11-93
NuwaII-1_CPB	gb|AHGY02200852.1|:35-134
NuwaII-1_CPB	gb|AHGY02200852.1|:15027-15173
NuwaII-1_CPB	gb|AHGY02200852.1|:38679-38736
NuwaII-1_CPB	gb|AHGY02200852.1|:7890-11740
NuwaII-1_CPB	gb|AHGY02039525.1|:7562-11840
NuwaII-1_CPB	gb|AHGY02243518.1|:621-2431
NuwaII-1_CPB	gb|AHGY02239332.1|:937-4835
NuwaII-1_CPB	gb|AHGY02224424.1|:1854-3747
NuwaII-1_CPB	gb|AHGY02082289.1|:7751-11990
NuwaII-1_CPB	gb|AHGY02082289.1|:83-162
NuwaII-1_CPB	gb|AHGY02020665.1|:9698-11321
NuwaII-1_CPB	gb|AHGY02259633.1|:1-1821
NuwaII-1_CPB	gb|AHGY02245327.1|:5-2499
NuwaII-1_CPB	gb|AHGY02132194.1|:2-1729
NuwaII-1_CPB	gb|AHGY02131122.1|:176-1853
NuwaII-1_CPB	gb|AHGY02043770.1|:20549-24858
NuwaII-1_CPB	gb|AHGY02010520.1|:2-2987
NuwaII-1_CPB	gb|AHGY02252853.1|:10226-10298
NuwaII-1_CPB	gb|AHGY02252853.1|:1-2008
NuwaII-1_CPB	gb|AHGY02227567.1|:3843-7209
NuwaII-1_CPB	gb|AHGY02111265.1|:5174-5314
NuwaII-1_CPB	gb|AHGY02111265.1|:1-3874
NuwaII-1_CPB	gb|AHGY02038161.1|:32231-35323
NuwaII-1_CPB	gb|AHGY02038161.1|:31-2493
NuwaII-1_CPB	gb|AHGY02016152.1|:10048-14594
NuwaII-1_CPB	gb|AHGY02016152.1|:40810-42019
NuwaII-1_CPB	gb|AHGY02016152.1|:14220-14372
NuwaII-1_CPB	gb|AHGY02212346.1|:1-2896
NuwaII-1_CPB	gb|AHGY02088131.1|:1456-3736
NuwaII-1_CPB	gb|AHGY02088131.1|:12620-16922
NuwaII-1_CPB	gb|AHGY02073392.1|:15424-15508

NuwaII-1_CPB	gb|AHGY02073392.1|:36810-36922
NuwaII-1_CPB	gb|AHGY02073392.1|:32951-36319
NuwaII-1_CPB	gb|AHGY02073392.1|:15378-15481
NuwaII-1_CPB	gb|AHGY02064253.1|:497-2326
NuwaII-1_CPB	gb|AHGY02054488.1|:8277-10135
NuwaII-1_CPB	gb|AHGY02054488.1|:3349-3521
NuwaII-1_CPB	gb|AHGY02001913.1|:28685-28758
NuwaII-1_CPB	gb|AHGY02001913.1|:11724-15968
NuwaII-1_CPB	gb|AHGY02251341.1|:1-2122
NuwaII-1_CPB	gb|AHGY02026873.1|:15801-18855
NuwaII-1_CPB	gb|AHGY02026873.1|:133-186
NuwaII-1_CPB	gb|AHGY02000634.1|:4367-8674
NuwaII-1_CPB	gb|AHGY02000634.1|:16865-16970
NuwaII-1_CPB	gb|AHGY02103253.1|:205-3350
NuwaII-1_CPB	gb|AHGY02028823.1|:4935-8554
NuwaII-1_CPB	gb|AHGY02028823.1|:46-3666
NuwaII-1_CPB	gb|AHGY02001345.1|:52718-54443
NuwaII-1_CPB	gb|AHGY02001345.1|:45406-45887
NuwaII-1_CPB	gb|AHGY02001345.1|:17099-17261
NuwaII-1_CPB	gb|AHGY02237991.1|:18361-21285
NuwaII-1_CPB	gb|AHGY02230485.1|:1-2832
NuwaII-1_CPB	gb|AHGY02078217.1|:16837-16996
NuwaII-1_CPB	gb|AHGY02078217.1|:18-2372
NuwaII-1_CPB	gb|AHGY02048031.1|:2064-6278
NuwaII-1_CPB	gb|AHGY02054557.1|:34-4102
NuwaII-1_CPB	gb|AHGY02054557.1|:7753-7858
NuwaII-1_CPB	gb|AHGY02001584.1|:11884-11939
NuwaII-1_CPB	gb|AHGY02001584.1|:6839-11097
NuwaII-1_CPB	gb|AHGY02253341.1|:11537-13160
NuwaII-1_CPB	gb|AHGY02222514.1|:106-214
NuwaII-1_CPB	gb|AHGY02222514.1|:15985-18362
NuwaII-1_CPB	gb|AHGY02222514.1|:75-164
NuwaII-1_CPB	gb|AHGY02100196.1|:1864-6075
NuwaII-1_CPB	gb|AHGY02197879.1|:39-2842
NuwaII-1_CPB	gb|AHGY02057758.1|:8718-20474
NuwaII-1_CPB	gb|AHGY02057758.1|:29557-29742
NuwaII-1_CPB	gb|AHGY02023702.1|:1-1845
NuwaII-1_CPB	gb|AHGY02211217.1|:5083-5289
NuwaII-1_CPB	gb|AHGY02211217.1|:64100-64255
NuwaII-1_CPB	gb|AHGY02211217.1|:1-1802
NuwaII-1_CPB	gb|AHGY02114649.1|:48-2652
NuwaII-1_CPB	gb|AHGY02096161.1|:64-165
NuwaII-1_CPB	gb|AHGY02096161.1|:15847-17895
NuwaII-1_CPB	gb|AHGY02096161.1|:3347-7666

NuwaII-1_CPB	gb|AHGY02085001.1|:12986-13089
NuwaII-1_CPB	gb|AHGY02085001.1|:28709-33006
NuwaII-1_CPB	gb|AHGY02083372.1|:10-101
NuwaII-1_CPB	gb|AHGY02083372.1|:20584-24389
NuwaII-1_CPB	gb|AHGY02083372.1|:29574-33790
NuwaII-1_CPB	gb|AHGY02083372.1|:7647-7712
NuwaII-1_CPB	gb|AHGY02031770.1|:18904-19857
NuwaII-1_CPB	gb|AHGY02031770.1|:11772-16028
NuwaII-1_CPB	gb|AHGY02018507.1|:8005-12249
NuwaII-1_CPB	gb|AHGY02238721.1|:15022-18972
NuwaII-1_CPB	gb|AHGY02208046.1|:686-4970
NuwaII-1_CPB	gb|AHGY02201950.1|:9591-13764
NuwaII-2_CPB	gb|AHGY02076372.1|:21814-26741
NuwaII-2_CPB	gb|AHGY02074467.1|:23683-28587
NuwaII-2_CPB	gb|AHGY02219490.1|:1-2883
NuwaII-2_CPB	gb|AHGY02007375.1|:56-3298
NuwaII-2_CPB	gb|AHGY02222453.1|:12668-16280
NuwaII-2_CPB	gb|AHGY02088514.1|:1-2129
NuwaII-2_CPB	gb|AHGY02077288.1|:38883-41903
NuwaII-2_CPB	gb|AHGY02252247.1|:38579-42447
NuwaII-2_CPB	gb|AHGY02200568.1|:13-2532
NuwaII-2_CPB	gb|AHGY02026189.1|:9949-13019
NuwaII-2_CPB	gb|AHGY02023095.1|:571-3564
NuwaII-2_CPB	gb|AHGY02090592.1|:14-4196
NuwaII-2_CPB	gb|AHGY02060718.1|:5035-5256
NuwaII-2_CPB	gb|AHGY02060718.1|:81-2484
NuwaII-2_CPB	gb|AHGY02083042.1|:3683-6612
NuwaII-2_CPB	gb|AHGY02253428.1|:59240-62816
NuwaII-2_CPB	gb|AHGY02253428.1|:56303-56489
NuwaII-2_CPB	gb|AHGY02253428.1|:27457-27629
NuwaII-2_CPB	gb|AHGY02252367.1|:59-2474
NuwaII-2_CPB	gb|AHGY02213695.1|:366-2693
NuwaII-2_CPB	gb|AHGY02171094.1|:643-3362
NuwaII-2_CPB	gb|AHGY02055882.1|:1-2422
NuwaII-2_CPB	gb|AHGY02004490.1|:10414-15660
NuwaII-2_CPB	gb|AHGY02224582.1|:23-4277
NuwaII-2_CPB	gb|AHGY02169580.1|:1-3503
NuwaII-2_CPB	gb|AHGY02213579.1|:3-4944
NuwaII-2_CPB	gb|AHGY02063040.1|:8103-15157
NuwaII-2_CPB	gb|AHGY02110602.1|:4310-10881
NuwaII-2_CPB	gb|AHGY02110602.1|:14693-14778
NuwaII-2_CPB	gb|AHGY02008511.1|:18831-23190
NuwaII-2_CPB	gb|AHGY02043892.1|:55534-55639
NuwaII-2_CPB	gb|AHGY02043892.1|:74474-76623

NuwaII-2_CPB	gb|AHGY02259409.1|:14241-19491
NuwaII-2_CPB	gb|AHGY02105237.1|:113-2171
NuwaII-2_CPB	gb|AHGY02027094.1|:3-2574
NuwaII-2_CPB	gb|AHGY02175640.1|:9-3036
NuwaII-2_CPB	gb|AHGY02102762.1|:48-2713
NuwaII-2_CPB	gb|AHGY02068922.1|:3778-7733
NuwaII-2_CPB	gb|AHGY02252627.1|:26896-30311
NuwaII-2_CPB	gb|AHGY02252627.1|:8442-8658
NuwaII-2_CPB	gb|AHGY02013022.1|:86-2133
NuwaII-2_CPB	gb|AHGY02227854.1|:7118-12333
NuwaII-2_CPB	gb|AHGY02223466.1|:101-1365
NuwaII-2_CPB	gb|AHGY02000794.1|:11997-16993
NuwaII-2_CPB	gb|AHGY02010431.1|:7286-11352
NuwaII-2_CPB	gb|AHGY02133783.1|:1-1569
NuwaII-2_CPB	gb|AHGY02261792.1|:2936-5639
NuwaII-2_CPB	gb|AHGY02006825.1|:9290-12919
NuwaII-2_CPB	gb|AHGY02101756.1|:40-3676
NuwaII-2_CPB	gb|AHGY02019630.1|:44-3176
NuwaII-2_CPB	gb|AHGY02246969.1|:531-2937
NuwaII-2_CPB	gb|AHGY02225572.1|:448-3025
NuwaII-2_CPB	gb|AHGY02073830.1|:18437-18622
NuwaII-2_CPB	gb|AHGY02073830.1|:1-2262
NuwaII-2_CPB	gb|AHGY02262062.1|:44010-46342
NuwaII-2_CPB	gb|AHGY02026112.1|:19147-23988
NuwaII-2_CPB	gb|AHGY02028483.1|:6750-11571
NuwaII-2_CPB	gb|AHGY02028483.1|:35733-35890
NuwaII-2_CPB	gb|AHGY02023279.1|:1-2409
NuwaII-2_CPB	gb|AHGY02253796.1|:4865-5054
NuwaII-2_CPB	gb|AHGY02253796.1|:36-2593
NuwaII-2_CPB	gb|AHGY02205273.1|:40171-44056
NuwaII-2_CPB	gb|AHGY02002346.1|:2526-7470
NuwaII-2_CPB	gb|AHGY02259294.1|:40473-44028
NuwaII-2_CPB	gb|AHGY02119978.1|:21-1657
NuwaII-2_CPB	gb|AHGY02107719.1|:6178-8413
NuwaII-2_CPB	gb|AHGY02237300.1|:1113-1163
NuwaII-2_CPB	gb|AHGY02237300.1|:1-3030
NuwaII-2_CPB	gb|AHGY02262256.1|:1-2487
NuwaII-2_CPB	gb|AHGY02224144.1|:451-628
NuwaII-2_CPB	gb|AHGY02224144.1|:20271-25252
NuwaII-2_CPB	gb|AHGY02201579.1|:88-2401
NuwaII-2_CPB	gb|AHGY02066261.1|:1778-1965
NuwaII-2_CPB	gb|AHGY02066261.1|:5811-9698
NuwaII-2_CPB	gb|AHGY02015251.1|:11343-16188
NuwaII-2_CPB	gb|AHGY02098710.1|:45-2858

NuwaII-2_CPB	gb|AHGY02131089.1|:17-1852
NuwaII-2_CPB	gb|AHGY02066497.1|:1-2182
NuwaII-2_CPB	gb|AHGY02092356.1|:69-3574
NuwaII-2_CPB	gb|AHGY02023306.1|:1956-6892
NuwaII-2_CPB	gb|AHGY02248193.1|:34-2765
NuwaII-2_CPB	gb|AHGY02080869.1|:60-3514
NuwaII-2_CPB	gb|AHGY02030430.1|:11453-15140
NuwaII-2_CPB	gb|AHGY02195433.1|:35-2027
NuwaII-2_CPB	gb|AHGY02116444.1|:1180-6195
NuwaII-2_CPB	gb|AHGY02093712.1|:3240-3427
NuwaII-2_CPB	gb|AHGY02093712.1|:14320-16917
NuwaII-2_CPB	gb|AHGY02093712.1|:13447-13638
NuwaII-2_CPB	gb|AHGY02093712.1|:718-865
NuwaII-2_CPB	gb|AHGY02049334.1|:1760-2008
NuwaII-2_CPB	gb|AHGY02049334.1|:13-1359
NuwaII-2_CPB	gb|AHGY02207332.1|:15853-18176
NuwaII-2_CPB	gb|AHGY02016844.1|:1-4453
NuwaII-2_CPB	gb|AHGY02006853.1|:40-3444
NuwaII-2_CPB	gb|AHGY02029451.1|:8211-8362
NuwaII-2_CPB	gb|AHGY02029451.1|:1-2930
NuwaII-2_CPB	gb|AHGY02015881.1|:7290-10639
NuwaII-2_CPB	gb|AHGY02011805.1|:4419-9359
NuwaII-2_CPB	gb|AHGY02208965.1|:1-2139
NuwaII-2_CPB	gb|AHGY02253579.1|:1372-6268
NuwaII-2_CPB	gb|AHGY02111353.1|:2-2804
NuwaII-2_CPB	gb|AHGY02077019.1|:1-2691
NuwaII-2_CPB	gb|AHGY02056495.1|:1-3471
NuwaII-2_CPB	gb|AHGY02028612.1|:13663-13854
NuwaII-2_CPB	gb|AHGY02028612.1|:25608-25775
NuwaII-2_CPB	gb|AHGY02028612.1|:1-3610
NuwaII-2_CPB	gb|AHGY02239287.1|:41557-43936
NuwaII-2_CPB	gb|AHGY02239287.1|:14598-14787
NuwaII-2_CPB	gb|AHGY02077374.1|:48-2362
NuwaII-2_CPB	gb|AHGY02187017.1|:33-2455
NuwaII-2_CPB	gb|AHGY02135316.1|:10-1450
NuwaII-2_CPB	gb|AHGY02066739.1|:1-4090
NuwaII-2_CPB	gb|AHGY02013563.1|:14770-18162
NuwaII-2_CPB	gb|AHGY02200719.1|:6438-10499
NuwaII-2_CPB	gb|AHGY02055956.1|:259-960
NuwaII-2_CPB	gb|AHGY02055956.1|:27146-27309
NuwaII-2_CPB	gb|AHGY02055956.1|:4966-9988
NuwaII-2_CPB	gb|AHGY02252252.1|:5113-10115
NuwaII-2_CPB	gb|AHGY02251025.1|:3-1702
NuwaII-2_CPB	gb|AHGY02226247.1|:3346-6180

NuwaII-2_CPB	gb|AHGY02226247.1|:20193-20372
NuwaII-2_CPB	gb|AHGY02208090.1|:61347-61496
NuwaII-2_CPB	gb|AHGY02208090.1|:76528-76600
NuwaII-2_CPB	gb|AHGY02208090.1|:79920-80006
NuwaII-2_CPB	gb|AHGY02208090.1|:19-4471
NuwaII-2_CPB	gb|AHGY02207438.1|:684-4748
NuwaII-2_CPB	gb|AHGY02134428.1|:11-1512
NuwaII-2_CPB	gb|AHGY02108659.1|:24133-27941
NuwaII-2_CPB	gb|AHGY02238702.1|:51141-52240
NuwaII-2_CPB	gb|AHGY02238702.1|:13662-18600
NuwaII-2_CPB	gb|AHGY02195005.1|:1-2024
NuwaII-2_CPB	gb|AHGY02134822.1|:45-1400
NuwaII-2_CPB	gb|AHGY02033988.1|:2-3208
NuwaII-2_CPB	gb|AHGY02031893.1|:28552-32100
NuwaII-2_CPB	gb|AHGY02201275.1|:9899-13588
NuwaII-2_CPB	gb|AHGY02201275.1|:1502-1716
NuwaII-2_CPB	gb|AHGY02034238.1|:19036-19213
NuwaII-2_CPB	gb|AHGY02034238.1|:30005-35007
NuwaII-2_CPB	gb|AHGY02034238.1|:25239-25309
NuwaII-2_CPB	gb|AHGY02005141.1|:4846-9768
NuwaII-2_CPB	gb|AHGY02043587.1|:14527-19539
NuwaII-2_CPB	gb|AHGY02043587.1|:5528-6815
NuwaII-2_CPB	gb|AHGY02133910.1|:1-1565
NuwaII-2_CPB	gb|AHGY02083322.1|:461-3400
NuwaII-2_CPB	gb|AHGY02016712.1|:27759-31732
NuwaII-2_CPB	gb|AHGY02016712.1|:9143-14013
NuwaII-2_CPB	gb|AHGY02245103.1|:618-3227
NuwaII-2_CPB	gb|AHGY02135178.1|:1-1463
NuwaII-2_CPB	gb|AHGY02133252.1|:72-1572
NuwaII-2_CPB	gb|AHGY02021580.1|:11053-14729
NuwaII-2_CPB	gb|AHGY02247536.1|:20391-22709
NuwaII-2_CPB	gb|AHGY02228688.1|:1-2362
NuwaII-2_CPB	gb|AHGY02042662.1|:9079-9280
NuwaII-2_CPB	gb|AHGY02042662.1|:13527-18539
NuwaII-2_CPB	gb|AHGY02091310.1|:4966-5133
NuwaII-2_CPB	gb|AHGY02091310.1|:16-2831
NuwaII-2_CPB	gb|AHGY02202229.1|:53-4723
NuwaII-2_CPB	gb|AHGY02099477.1|:10-3124
NuwaII-2_CPB	gb|AHGY02261918.1|:4-2867
NuwaII-2_CPB	gb|AHGY02257750.1|:12627-16361
NuwaII-2_CPB	gb|AHGY02257750.1|:8055-8143
NuwaII-2_CPB	gb|AHGY02228594.1|:1-2754
NuwaII-2_CPB	gb|AHGY02222321.1|:29967-32361
NuwaII-2_CPB	gb|AHGY02076327.1|:15031-19901

NuwaII-2_CPB	gb|AHGY02109503.1|:1311-3552
NuwaII-2_CPB	gb|AHGY02023134.1|:31-2881
NuwaII-2_CPB	gb|AHGY02012630.1|:27-1701
NuwaII-2_CPB	gb|AHGY02033898.1|:8572-13401
NuwaII-2_CPB	gb|AHGY02254268.1|:1753-6557
NuwaII-2_CPB	gb|AHGY02076302.1|:10601-18636
NuwaII-2_CPB	gb|AHGY02076302.1|:11996-12445
NuwaII-2_CPB	gb|AHGY02061455.1|:14249-14330
NuwaII-2_CPB	gb|AHGY02061455.1|:59265-59404
NuwaII-2_CPB	gb|AHGY02061455.1|:93696-94781
NuwaII-2_CPB	gb|AHGY02061455.1|:68657-68755
NuwaII-2_CPB	gb|AHGY02061455.1|:38272-38454
NuwaII-2_CPB	gb|AHGY02061455.1|:25404-25487
NuwaII-2_CPB	gb|AHGY02061455.1|:31-3162
NuwaII-2_CPB	gb|AHGY02042144.1|:27-1466
NuwaII-2_CPB	gb|AHGY02012759.1|:26988-30544
NuwaII-2_CPB	gb|AHGY02012759.1|:12864-12943
NuwaII-2_CPB	gb|AHGY02089992.1|:39-1260
NuwaII-2_CPB	gb|AHGY02089075.1|:249-4938
NuwaII-2_CPB	gb|AHGY02247827.1|:1527-6479
NuwaII-2_CPB	gb|AHGY02246044.1|:7082-7178
NuwaII-2_CPB	gb|AHGY02246044.1|:41834-45054
NuwaII-2_CPB	gb|AHGY02246044.1|:32507-32580
NuwaII-2_CPB	gb|AHGY02194794.1|:11-2210
NuwaII-2_CPB	gb|AHGY02019494.1|:1861-5354
NuwaII-2_CPB	gb|AHGY02220976.1|:1-1480
NuwaII-2_CPB	gb|AHGY02135332.1|:78-1419
NuwaII-2_CPB	gb|AHGY02058342.1|:1-2297
NuwaII-2_CPB	gb|AHGY02235979.1|:13367-17700
NuwaII-2_CPB	gb|AHGY02223233.1|:91-3716
NuwaII-2_CPB	gb|AHGY02068345.1|:2504-7559
NuwaII-2_CPB	gb|AHGY02058152.1|:9172-10891
NuwaII-2_CPB	gb|AHGY02045373.1|:31453-35526
NuwaII-2_CPB	gb|AHGY02039531.1|:43369-43535
NuwaII-2_CPB	gb|AHGY02039531.1|:46801-51691
NuwaII-2_CPB	gb|AHGY02014072.1|:1463-3831
NuwaII-2_CPB	gb|AHGY02059205.1|:56-2460
NuwaII-2_CPB	gb|AHGY02015547.1|:490-1717
NuwaII-2_CPB	gb|AHGY02223256.1|:7562-11846
NuwaII-2_CPB	gb|AHGY02137923.1|:2-1203
NuwaII-2_CPB	gb|AHGY02048320.1|:27974-31607
NuwaII-2_CPB	gb|AHGY02138163.1|:1-1253
NuwaII-2_CPB	gb|AHGY02134936.1|:3-1478
NuwaII-2_CPB	gb|AHGY02084724.1|:2666-7669

NuwaII-2_CPB	gb|AHGY02249770.1|:15-4236
NuwaII-2_CPB	gb|AHGY02249770.1|:8920-9103
NuwaII-2_CPB	gb|AHGY02238037.1|:888-5771
NuwaII-2_CPB	gb|AHGY02195206.1|:1-2087
NuwaII-2_CPB	gb|AHGY02131009.1|:44-1872
NuwaII-2_CPB	gb|AHGY02109758.1|:18317-20695
NuwaII-2_CPB	gb|AHGY02106064.1|:81-3115
NuwaII-2_CPB	gb|AHGY02081603.1|:14-3292
NuwaII-2_CPB	gb|AHGY02076443.1|:8-4036
NuwaII-2_CPB	gb|AHGY02033851.1|:72-1847
NuwaII-2_CPB	gb|AHGY02013523.1|:2-3619
NuwaII-2_CPB	gb|AHGY02000739.1|:26-2065
NuwaII-2_CPB	gb|AHGY02057716.1|:16664-16782
NuwaII-2_CPB	gb|AHGY02057716.1|:5362-10014
NuwaII-2_CPB	gb|AHGY02002058.1|:1-4419
NuwaII-2_CPB	gb|AHGY02216597.1|:24-2605
NuwaII-2_CPB	gb|AHGY02131714.1|:82-1781
NuwaII-2_CPB	gb|AHGY02020873.1|:1-3664
NuwaII-2_CPB	gb|AHGY02260939.1|:28588-32230
NuwaII-2_CPB	gb|AHGY02256516.1|:12-1513
NuwaII-2_CPB	gb|AHGY02234742.1|:30-2872
NuwaII-2_CPB	gb|AHGY02208231.1|:6307-9626
NuwaII-2_CPB	gb|AHGY02208231.1|:9848-10039
NuwaII-2_CPB	gb|AHGY02107736.1|:3208-6189
NuwaII-2_CPB	gb|AHGY02053294.1|:2692-5801
NuwaII-2_CPB	gb|AHGY02251795.1|:33-1332
NuwaII-2_CPB	gb|AHGY02201792.1|:12-2936
NuwaII-2_CPB	gb|AHGY02111270.1|:58-129
NuwaII-2_CPB	gb|AHGY02111270.1|:11601-16678
NuwaII-2_CPB	gb|AHGY02100879.1|:26845-30001
NuwaII-2_CPB	gb|AHGY02100879.1|:778-876
NuwaII-2_CPB	gb|AHGY02062195.1|:1-3505
NuwaII-2_CPB	gb|AHGY02040711.1|:6228-9719
NuwaII-2_CPB	gb|AHGY02040711.1|:1638-1719
NuwaII-2_CPB	gb|AHGY02035606.1|:1690-4074
NuwaII-2_CPB	gb|AHGY02027812.1|:19-2434
NuwaII-2_CPB	gb|AHGY02022962.1|:1-1689
NuwaII-2_CPB	gb|AHGY02007886.1|:17-3508
NuwaII-2_CPB	gb|AHGY02252428.1|:28380-30646
NuwaII-2_CPB	gb|AHGY02252428.1|:9-2361
NuwaII-2_CPB	gb|AHGY02235672.1|:25340-30318
NuwaII-2_CPB	gb|AHGY02096321.1|:6383-10424
NuwaII-2_CPB	gb|AHGY02211762.1|:28653-32803
NuwaII-2_CPB	gb|AHGY02211762.1|:56216-56310

NuwaII-2_CPB	gb|AHGY02005721.1|:1-3784
NuwaII-2_CPB	gb|AHGY02234067.1|:27-3518
NuwaII-2_CPB	gb|AHGY02086929.1|:89-1724
NuwaII-2_CPB	gb|AHGY02059253.1|:6778-11788
NuwaII-2_CPB	gb|AHGY02059253.1|:1669-6292
NuwaII-2_CPB	gb|AHGY02051036.1|:1-2480
NuwaII-2_CPB	gb|AHGY02032067.1|:18-2522
NuwaII-2_CPB	gb|AHGY02260332.1|:14747-17133
NuwaII-2_CPB	gb|AHGY02259547.1|:5459-7672
NuwaII-2_CPB	gb|AHGY02184476.1|:38-2494
NuwaII-2_CPB	gb|AHGY02133763.1|:48-1552
NuwaII-2_CPB	gb|AHGY02133424.1|:65-1556
NuwaII-2_CPB	gb|AHGY02088786.1|:3288-7824
NuwaII-2_CPB	gb|AHGY02084693.1|:3542-3635
NuwaII-2_CPB	gb|AHGY02084693.1|:75-2355
NuwaII-2_CPB	gb|AHGY02078281.1|:1-2332
NuwaII-2_CPB	gb|AHGY02077225.1|:27-2104
NuwaII-2_CPB	gb|AHGY02058663.1|:1-3348
NuwaII-2_CPB	gb|AHGY02054558.1|:13062-17890
NuwaII-2_CPB	gb|AHGY02027592.1|:12317-17219
NuwaII-2_CPB	gb|AHGY02027592.1|:34303-34489
NuwaII-2_CPB	gb|AHGY02015850.1|:21-2411
NuwaII-2_CPB	gb|AHGY02249228.1|:1517-4144
NuwaII-2_CPB	gb|AHGY02237535.1|:9339-9504
NuwaII-2_CPB	gb|AHGY02237535.1|:87-2472
NuwaII-2_CPB	gb|AHGY02235100.1|:4872-7981
NuwaII-2_CPB	gb|AHGY02227529.1|:144-2424
NuwaII-2_CPB	gb|AHGY02235208.1|:114-2123
NuwaII-2_CPB	gb|AHGY02220424.1|:8307-13982
NuwaII-2_CPB	gb|AHGY02136949.1|:4-1257
NuwaII-2_CPB	gb|AHGY02133624.1|:78-1584
NuwaII-2_CPB	gb|AHGY02133214.1|:83-1597
NuwaII-2_CPB	gb|AHGY02084701.1|:10713-13149
NuwaII-2_CPB	gb|AHGY02052682.1|:76-2363
NuwaII-2_CPB	gb|AHGY02044589.1|:21016-26018
NuwaII-2_CPB	gb|AHGY02044589.1|:53004-53184
NuwaII-2_CPB	gb|AHGY02044589.1|:89077-89158
NuwaII-2_CPB	gb|AHGY02254700.1|:10-2298
NuwaII-2_CPB	gb|AHGY02239896.1|:96-2108
NuwaII-2_CPB	gb|AHGY02098641.1|:7512-11158
NuwaII-2_CPB	gb|AHGY02110452.1|:18-1831
NuwaII-2_CPB	gb|AHGY02074602.1|:9634-13334
NuwaII-2_CPB	gb|AHGY02205564.1|:1-4092
NuwaII-2_CPB	gb|AHGY02073786.1|:512-3239

NuwaII-2_CPB	gb|AHGY02022627.1|:7458-10424
NuwaII-2_CPB	gb|AHGY02101434.1|:1-1984
NuwaII-2_CPB	gb|AHGY02233352.1|:13-2715
NuwaII-2_CPB	gb|AHGY02218170.1|:19434-23404
NuwaII-2_CPB	gb|AHGY02045142.1|:2732-5198
NuwaII-2_CPB	gb|AHGY02038317.1|:3-3537
NuwaII-2_CPB	gb|AHGY02199160.1|:1588-4977
NuwaII-2_CPB	gb|AHGY02072511.1|:5259-5424
NuwaII-2_CPB	gb|AHGY02072511.1|:1-2450
NuwaII-2_CPB	gb|AHGY02010546.1|:11560-15924
NuwaII-2_CPB	gb|AHGY02010546.1|:31311-36282
NuwaII-2_CPB	gb|AHGY02240194.1|:1-2274
NuwaII-2_CPB	gb|AHGY02230484.1|:14784-14968
NuwaII-2_CPB	gb|AHGY02230484.1|:28656-29850
NuwaII-2_CPB	gb|AHGY02230484.1|:9-3131
NuwaII-2_CPB	gb|AHGY02210811.1|:36587-41523
NuwaII-2_CPB	gb|AHGY02139676.1|:24-1210
NuwaII-2_CPB	gb|AHGY02084729.1|:52-4342
NuwaII-3_CPB	gb|AHGY02062631.1|:620-5472
NuwaII-3_CPB	gb|AHGY02030373.1|:3737-8354
NuwaII-3_CPB	gb|AHGY02253336.1|:36-2047
NuwaII-3_CPB	gb|AHGY02248661.1|:26276-31041
NuwaII-3_CPB	gb|AHGY02085226.1|:136-4200
NuwaII-3_CPB	gb|AHGY02043994.1|:2319-7156
NuwaII-3_CPB	gb|AHGY02246078.1|:13151-15127
NuwaII-3_CPB	gb|AHGY02246078.1|:15132-15192
NuwaII-3_CPB	gb|AHGY02036685.1|:9611-11738
NuwaII-3_CPB	gb|AHGY02081034.1|:213-4991
NuwaII-3_CPB	gb|AHGY02065594.1|:8219-12993
NuwaII-3_CPB	gb|AHGY02259578.1|:21112-24870
NuwaII-3_CPB	gb|AHGY02047970.1|:11373-16656
NuwaII-3_CPB	gb|AHGY02047970.1|:1323-6395
NuwaII-3_CPB	gb|AHGY02021688.1|:4974-8289
NuwaII-3_CPB	gb|AHGY02235459.1|:12539-12628
NuwaII-3_CPB	gb|AHGY02235459.1|:25217-27324
NuwaII-3_CPB	gb|AHGY02025067.1|:24-2158
NuwaII-3_CPB	gb|AHGY02068668.1|:35-2161
NuwaII-3_CPB	gb|AHGY02013602.1|:10423-15175
NuwaII-3_CPB	gb|AHGY02013602.1|:9671-9747
NuwaII-3_CPB	gb|AHGY02084711.1|:2093-6862
NuwaII-3_CPB	gb|AHGY02262115.1|:63-1818
NuwaII-3_CPB	gb|AHGY02213525.1|:1-1894
NuwaII-3_CPB	gb|AHGY02078271.1|:20817-22765
NuwaII-3_CPB	gb|AHGY02209651.1|:7-4367

NuwaII-3_CPB	gb|AHGY02080006.1|:1073-1164
NuwaII-3_CPB	gb|AHGY02080006.1|:26218-31058
NuwaII-3_CPB	gb|AHGY02069900.1|:1-2375
NuwaII-3_CPB	gb|AHGY02044993.1|:128-2219
NuwaII-3_CPB	gb|AHGY02083150.1|:1-3578
NuwaII-3_CPB	gb|AHGY02008577.1|:9384-14235
NuwaII-3_CPB	gb|AHGY02066658.1|:1500-6355
NuwaII-3_CPB	gb|AHGY02066658.1|:27712-27788
NuwaII-3_CPB	gb|AHGY02066658.1|:12719-16527
NuwaII-3_CPB	gb|AHGY02002939.1|:22034-22106
NuwaII-3_CPB	gb|AHGY02002939.1|:43054-47909
NuwaII-3_CPB	gb|AHGY02047980.1|:32385-37228
NuwaII-3_CPB	gb|AHGY02047980.1|:9148-12838
NuwaII-3_CPB	gb|AHGY02031136.1|:25778-30522
NuwaII-3_CPB	gb|AHGY02025559.1|:33-2558
NuwaII-3_CPB	gb|AHGY02120678.1|:561-2700
NuwaII-3_CPB	gb|AHGY02075336.1|:18259-21611
NuwaII-3_CPB	gb|AHGY02250963.1|:10640-15406
NuwaII-3_CPB	gb|AHGY02071481.1|:3109-7952
NuwaII-3_CPB	gb|AHGY02010897.1|:10527-13988
NuwaII-3_CPB	gb|AHGY02100958.1|:2333-6970
NuwaII-3_CPB	gb|AHGY02036745.1|:11553-16280
NuwaII-3_CPB	gb|AHGY02216502.1|:17144-17207
NuwaII-3_CPB	gb|AHGY02216502.1|:32795-37656
NuwaII-3_CPB	gb|AHGY02195410.1|:222-1944
NuwaII-3_CPB	gb|AHGY02114567.1|:1378-5091
NuwaII-3_CPB	gb|AHGY02256029.1|:4583-9401
NuwaII-3_CPB	gb|AHGY02093550.1|:124-2070
NuwaII-3_CPB	gb|AHGY02003176.1|:7679-9812
NuwaII-3_CPB	gb|AHGY02222626.1|:23-2421
NuwaII-3_CPB	gb|AHGY02056764.1|:1-2218
NuwaII-3_CPB	gb|AHGY02203716.1|:3942-7508
NuwaII-3_CPB	gb|AHGY02072629.1|:6692-10748
NuwaII-3_CPB	gb|AHGY02216026.1|:7880-12304
NuwaII-3_CPB	gb|AHGY02211010.1|:1-1734
NuwaII-3_CPB	gb|AHGY02022499.1|:5-1888
NuwaII-3_CPB	gb|AHGY02049758.1|:77-2208
NuwaII-3_CPB	gb|AHGY02249550.1|:30-2220
NuwaII-3_CPB	gb|AHGY02225451.1|:4628-9311
NuwaII-3_CPB	gb|AHGY02097403.1|:1519-3857
NuwaII-3_CPB	gb|AHGY02033151.1|:1-4085
NuwaII-3_CPB	gb|AHGY02023514.1|:1-2256
NuwaII-3_CPB	gb|AHGY02259549.1|:3302-8076
NuwaII-3_CPB	gb|AHGY02259549.1|:34663-34750

NuwaII-3_CPB	gb|AHGY02239373.1|:22095-23549
NuwaII-3_CPB	gb|AHGY02239373.1|:18231-21743
NuwaII-3_CPB	gb|AHGY02239373.1|:5034-5098
NuwaII-3_CPB	gb|AHGY02095966.1|:5888-10645
NuwaII-3_CPB	gb|AHGY02221199.1|:36583-38719
NuwaII-3_CPB	gb|AHGY02221199.1|:24655-24745
NuwaII-3_CPB	gb|AHGY02221199.1|:11451-11500
NuwaII-3_CPB	gb|AHGY02203662.1|:29-3670
NuwaII-3_CPB	gb|AHGY02261382.1|:20726-22957
NuwaII-3_CPB	gb|AHGY02058150.1|:1-2213
NuwaII-3_CPB	gb|AHGY02257197.1|:18189-20311
NuwaII-3_CPB	gb|AHGY02224754.1|:18-2129
NuwaII-3_CPB	gb|AHGY02198988.1|:30464-35225
NuwaII-3_CPB	gb|AHGY02011918.1|:22742-31862
NuwaII-3_CPB	gb|AHGY02247171.1|:427-3437
NuwaII-3_CPB	gb|AHGY02227594.1|:53-2293
NuwaII-3_CPB	gb|AHGY02206945.1|:5535-7588
NuwaII-3_CPB	gb|AHGY02235031.1|:11482-16298
NuwaII-3_CPB	gb|AHGY02228247.1|:59-2066
NuwaII-3_CPB	gb|AHGY02080173.1|:4647-8929
NuwaII-3_CPB	gb|AHGY02007520.1|:29215-33939
NuwaII-3_CPB	gb|AHGY02074657.1|:400-4712
NuwaII-3_CPB	gb|AHGY02028883.1|:15152-17381
NuwaII-3_CPB	gb|AHGY02023469.1|:4542-9387
NuwaII-3_CPB	gb|AHGY02012483.1|:7354-12128
NuwaII-3_CPB	gb|AHGY02048583.1|:15148-18853
NuwaII-3_CPB	gb|AHGY02012036.1|:65-2191
NuwaII-3_CPB	gb|AHGY02245108.1|:8812-12761
NuwaII-3_CPB	gb|AHGY02200537.1|:15-3110
NuwaII-3_CPB	gb|AHGY02063383.1|:2973-5739
NuwaII-3_CPB	gb|AHGY02011837.1|:24969-27238
NuwaII-3_CPB	gb|AHGY02118791.1|:72-1820
NuwaII-3_CPB	gb|AHGY02066464.1|:1344-6163
NuwaII-3_CPB	gb|AHGY02029189.1|:3303-8151
NuwaII-3_CPB	gb|AHGY02075885.1|:24-2045
NuwaII-3_CPB	gb|AHGY02233467.1|:12302-14412
NuwaII-3_CPB	gb|AHGY02081107.1|:11128-15879
NuwaII-3_CPB	gb|AHGY02255284.1|:4-1986
NuwaII-3_CPB	gb|AHGY02230390.1|:11552-13868
NuwaII-3_CPB	gb|AHGY02209259.1|:10-1768
NuwaII-3_CPB	gb|AHGY02049156.1|:9257-11729
NuwaII-3_CPB	gb|AHGY02049156.1|:313-1589
NuwaII-3_CPB	gb|AHGY02039095.1|:1-1874
NuwaII-3_CPB	gb|AHGY02246835.1|:6309-8434

NuwaII-3_CPB	gb|AHGY02033842.1|:1264-6108
NuwaII-3_CPB	gb|AHGY02223617.1|:2733-6088
NuwaII-3_CPB	gb|AHGY02109976.1|:6925-9066
NuwaII-3_CPB	gb|AHGY02104599.1|:43-2177
NuwaII-3_CPB	gb|AHGY02054669.1|:10726-14971
NuwaII-3_CPB	gb|AHGY02044913.1|:5314-10006
NuwaII-3_CPB	gb|AHGY02016288.1|:18334-23170
NuwaII-3_CPB	gb|AHGY02215575.1|:7987-9899
NuwaII-3_CPB	gb|AHGY02010506.1|:60-1977
NuwaII-3_CPB	gb|AHGY02042808.1|:1-4455
NuwaII-3_CPB	gb|AHGY02083366.1|:5-1805
NuwaII-3_CPB	gb|AHGY02052194.1|:1-2481
NuwaII-3_CPB	gb|AHGY02028624.1|:18648-21169
NuwaII-3_CPB	gb|AHGY02011842.1|:8876-8939
NuwaII-3_CPB	gb|AHGY02011842.1|:10380-15115
NuwaII-3_CPB	gb|AHGY02004111.1|:1-1948
NuwaII-3_CPB	gb|AHGY02252515.1|:16797-21467
NuwaII-3_CPB	gb|AHGY02252515.1|:3511-3598
NuwaII-3_CPB	gb|AHGY02076366.1|:15841-17978
NuwaII-3_CPB	gb|AHGY02042611.1|:34714-39529
NuwaII-3_CPB	gb|AHGY02094108.1|:27234-27307
NuwaII-3_CPB	gb|AHGY02094108.1|:7015-11047
NuwaII-3_CPB	gb|AHGY02080711.1|:298-4893
NuwaII-3_CPB	gb|AHGY02013006.1|:5-4345
NuwaII-3_CPB	gb|AHGY02088142.1|:9545-14363
NuwaII-3_CPB	gb|AHGY02261766.1|:22771-26866
NuwaII-3_CPB	gb|AHGY02235675.1|:13425-18363
NuwaII-3_CPB	gb|AHGY02235249.1|:3586-3672
NuwaII-3_CPB	gb|AHGY02235249.1|:8919-13731
NuwaII-3_CPB	gb|AHGY02130749.1|:118-1907
NuwaII-3_CPB	gb|AHGY02120469.1|:23-1695
NuwaII-3_CPB	gb|AHGY02252104.1|:27800-29933
NuwaII-3_CPB	gb|AHGY02252104.1|:29986-30300
NuwaII-3_CPB	gb|AHGY02208896.1|:21524-26455
NuwaII-3_CPB	gb|AHGY02187899.1|:336-2460
NuwaII-3_CPB	gb|AHGY02079915.1|:5-2006
NuwaII-3_CPB	gb|AHGY02078960.1|:1-2056
NuwaII-3_CPB	gb|AHGY02070896.1|:10413-15115
NuwaII-3_CPB	gb|AHGY02037921.1|:1336-3463
NuwaII-3_CPB	gb|AHGY02037921.1|:3500-3600
NuwaII-3_CPB	gb|AHGY02100920.1|:12741-14187
NuwaII-3_CPB	gb|AHGY02100920.1|:1601-6359
NuwaII-3_CPB	gb|AHGY02260562.1|:1014-5825
NuwaII-3_CPB	gb|AHGY02224392.1|:4-2483

NuwaII-3_CPB	gb|AHGY02223565.1|:1-4187
NuwaII-3_CPB	gb|AHGY02197152.1|:2655-6434
NuwaII-3_CPB	gb|AHGY02195196.1|:31-2087
NuwaII-3_CPB	gb|AHGY02073918.1|:439-2562
NuwaII-3_CPB	gb|AHGY02068593.1|:43693-48053
NuwaII-3_CPB	gb|AHGY02068593.1|:50662-51372
NuwaII-3_CPB	gb|AHGY02237460.1|:7722-9853
NuwaII-3_CPB	gb|AHGY02229282.1|:16898-19237
NuwaII-3_CPB	gb|AHGY02239184.1|:21159-24506
NuwaII-3_CPB	gb|AHGY02239184.1|:744-821
NuwaII-3_CPB	gb|AHGY02208873.1|:16670-16744
NuwaII-3_CPB	gb|AHGY02208873.1|:1300-5928
NuwaII-3_CPB	gb|AHGY02073787.1|:6778-11234
NuwaII-3_CPB	gb|AHGY02035611.1|:520-4319
NuwaII-3_CPB	gb|AHGY02253427.1|:6821-11644
NuwaII-3_CPB	gb|AHGY02253427.1|:33758-33814
NuwaII-3_CPB	gb|AHGY02210485.1|:1-1861
NuwaII-3_CPB	gb|AHGY02054512.1|:16940-21757
NuwaII-3_CPB	gb|AHGY02130682.1|:1-1901
NuwaII-3_CPB	gb|AHGY02063429.1|:64-2039
NuwaII-3_CPB	gb|AHGY02027351.1|:1-3909
NuwaII-3_CPB	gb|AHGY02245835.1|:14267-18395
NuwaII-3_CPB	gb|AHGY02106777.1|:32015-34925
NuwaII-3_CPB	gb|AHGY02105155.1|:1-1807
NuwaII-3_CPB	gb|AHGY02049090.1|:21-4196
NuwaII-3_CPB	gb|AHGY02036646.1|:65-2181
NuwaII-3_CPB	gb|AHGY02096570.1|:8368-13078
NuwaII-3_CPB	gb|AHGY02244691.1|:1-2714
NuwaII-3_CPB	gb|AHGY02240288.1|:14073-16205
NuwaII-3_CPB	gb|AHGY02235322.1|:1-4300
NuwaII-3_CPB	gb|AHGY02211388.1|:1-2206
NuwaII-3_CPB	gb|AHGY02109112.1|:4266-9080
NuwaII-3_CPB	gb|AHGY02055948.1|:82-1821
NuwaII-3_CPB	gb|AHGY02032422.1|:9313-12373
NuwaII-3_CPB	gb|AHGY02021630.1|:2-2068
NuwaII-3_CPB	gb|AHGY02200116.1|:66-2022
NuwaII-3_CPB	gb|AHGY02101834.1|:11146-13143
NuwaII-3_CPB	gb|AHGY02065083.1|:155-2414
NuwaII-3_CPB	gb|AHGY02259407.1|:6151-10924
NuwaII-3_CPB	gb|AHGY02249659.1|:4324-9216
NuwaII-3_CPB	gb|AHGY02221989.1|:28983-32266
NuwaII-3_CPB	gb|AHGY02203481.1|:16-1809
NuwaII-3_CPB	gb|AHGY02031324.1|:5202-5277
NuwaII-3_CPB	gb|AHGY02031324.1|:19331-23498

NuwaII-3_CPB	gb|AHGY02235147.1|:351-5163
NuwaII-3_CPB	gb|AHGY02064336.1|:289-446
NuwaII-3_CPB	gb|AHGY02064336.1|:9503-14365
NuwaII-3_CPB	gb|AHGY02033122.1|:1-2433
NuwaII-3_CPB	gb|AHGY02256142.1|:12937-15051
NuwaII-3_CPB	gb|AHGY02256142.1|:15060-15135
NuwaII-3_CPB	gb|AHGY02025557.1|:46831-46920
NuwaII-3_CPB	gb|AHGY02025557.1|:65227-66974
NuwaII-3_CPB	gb|AHGY02247605.1|:8063-12854
NuwaII-3_CPB	gb|AHGY02214648.1|:7030-11795
NuwaII-3_CPB	gb|AHGY02079061.1|:24621-24670
NuwaII-3_CPB	gb|AHGY02079061.1|:18928-24434
NuwaII-3_CPB	gb|AHGY02037980.1|:6511-8829
NuwaII-3_CPB	gb|AHGY02236547.1|:4382-7193
NuwaII-3_CPB	gb|AHGY02049624.1|:29290-34019
NuwaII-3_CPB	gb|AHGY02069984.1|:73-1827
NuwaII-3_CPB	gb|AHGY02010406.1|:35-2295
NuwaII-3_CPB	gb|AHGY02106178.1|:4109-6103
NuwaII-3_CPB	gb|AHGY02208607.1|:113092-115406
NuwaII-3_CPB	gb|AHGY02208607.1|:100456-100550
NuwaII-3_CPB	gb|AHGY02066690.1|:12332-14498
NuwaII-3_CPB	gb|AHGY02022435.1|:10454-10544
NuwaII-3_CPB	gb|AHGY02022435.1|:21868-24093
NuwaII-3_CPB	gb|AHGY02219281.1|:3721-7745
NuwaII-3_CPB	gb|AHGY02215668.1|:25033-27293
NuwaII-3_CPB	gb|AHGY02055803.1|:6229-11072
NuwaII-3_CPB	gb|AHGY02202541.1|:11894-16458
NuwaII-3_CPB	gb|AHGY02061860.1|:789-3259
NuwaII-3_CPB	gb|AHGY02054978.1|:13073-17345
NuwaII-3_CPB	gb|AHGY02259921.1|:230-3613
NuwaII-3_CPB	gb|AHGY02229473.1|:4-4082
NuwaII-3_CPB	gb|AHGY02055895.1|:10583-12648
NuwaII-3_CPB	gb|AHGY02182699.1|:1-1724
NuwaII-3_CPB	gb|AHGY02049858.1|:32581-34924
NuwaII-3_CPB	gb|AHGY02216618.1|:170-2167
NuwaII-3_CPB	gb|AHGY02008074.1|:6299-9103
NuwaII-3_CPB	gb|AHGY02066273.1|:11375-16316
NuwaII-3_CPB	gb|AHGY02131353.1|:1-1825
NuwaII-3_CPB	gb|AHGY02079994.1|:37684-42410
NuwaII-3_CPB	gb|AHGY02014831.1|:5246-10099
NuwaII-3_CPB	gb|AHGY02014831.1|:30-94
NuwaII-3_CPB	gb|AHGY02236528.1|:14812-19549
NuwaII-3_CPB	gb|AHGY02048430.1|:2-1829
NuwaII-3_CPB	gb|AHGY02254493.1|:36-1767

NuwaII-3_CPB	gb|AHGY02237509.1|:26456-31021
NuwaII-3_CPB	gb|AHGY02236971.1|:4160-8962
NuwaII-3_CPB	gb|AHGY02108943.1|:402-5250
NuwaII-3_CPB	gb|AHGY02204635.1|:70-2055
NuwaII-3_CPB	gb|AHGY02238783.1|:30083-33214
NuwaII-3_CPB	gb|AHGY02118177.1|:10-2247
NuwaII-3_CPB	gb|AHGY02050482.1|:3-2200
NuwaII-3_CPB	gb|AHGY02022689.1|:1-1904
NuwaII-3_CPB	gb|AHGY02083399.1|:642-2905
NuwaII-3_CPB	gb|AHGY02073425.1|:24686-29630
NuwaII-3_CPB	gb|AHGY02028955.1|:1-4813
NuwaII-3_CPB	gb|AHGY02092173.1|:2-4368
NuwaII-3_CPB	gb|AHGY02057345.1|:753-2749
NuwaII-3_CPB	gb|AHGY02057345.1|:2754-2816
NuwaII-3_CPB	gb|AHGY02238178.1|:33-1992
NuwaII-3_CPB	gb|AHGY02121237.1|:15-2142
NuwaII-3_CPB	gb|AHGY02081011.1|:5314-10095
NuwaII-3_CPB	gb|AHGY02013342.1|:7060-9313
NuwaII-3_CPB	gb|AHGY02059867.1|:6903-11706
NuwaII-3_CPB	gb|AHGY02028959.1|:18900-23890
NuwaII-3_CPB	gb|AHGY02016259.1|:2962-7813
NuwaII-3_CPB	gb|AHGY02068589.1|:48-2294
NuwaII-3_CPB	gb|AHGY02091977.1|:2486-7221
NuwaII-3_CPB	gb|AHGY02063065.1|:1-153
NuwaII-3_CPB	gb|AHGY02063065.1|:156-2146
NuwaII-3_CPB	gb|AHGY02224744.1|:34-2146
NuwaII-3_CPB	gb|AHGY02033467.1|:8208-10607
NuwaII-3_CPB	gb|AHGY02014372.1|:39081-43157
NuwaII-3_CPB	gb|AHGY02011762.1|:1092-3356
NuwaII-3_CPB	gb|AHGY02067115.1|:13478-18315
NuwaII-3_CPB	gb|AHGY02042909.1|:370-2109
NuwaII-3_CPB	gb|AHGY02248483.1|:5488-7885
NuwaII-3_CPB	gb|AHGY02248483.1|:1-2337
NuwaII-3_CPB	gb|AHGY02211982.1|:4080-8575
NuwaII-3_CPB	gb|AHGY02211982.1|:2337-2424
NuwaII-3_CPB	gb|AHGY02036113.1|:19100-23757
NuwaII-3_CPB	gb|AHGY02226503.1|:19731-23302
NuwaII-3_CPB	gb|AHGY02221638.1|:11770-15321
NuwaII-3_CPB	gb|AHGY02073294.1|:6089-8201
NuwaII-3_CPB	gb|AHGY02073294.1|:216-8118
NuwaII-3_CPB	gb|AHGY02255190.1|:47-1657
NuwaII-3_CPB	gb|AHGY02222892.1|:6497-11284
NuwaII-3_CPB	gb|AHGY02175208.1|:4-3066
NuwaII-3_CPB	gb|AHGY02109422.1|:3675-8456

NuwaII-4_CPB	#REF!-8717-411
NuwaII-4_CPB	gb|AHGY02101683.1|:990-4887
NuwaII-4_CPB	gb|AHGY02086867.1|:40546-41344
NuwaII-4_CPB	gb|AHGY02086867.1|:19915-23888
NuwaII-4_CPB	gb|AHGY02213252.1|:16626-20542
NuwaII-4_CPB	gb|AHGY02011165.1|:11183-14619
NuwaII-4_CPB	gb|AHGY02257746.1|:4-2622
NuwaII-4_CPB	gb|AHGY02221017.1|:3756-8172
NuwaII-4_CPB	gb|AHGY02093691.1|:7969-12303
NuwaII-4_CPB	gb|AHGY02000719.1|:29934-33496
NuwaII-4_CPB	gb|AHGY02260615.1|:29073-29150
NuwaII-4_CPB	gb|AHGY02260615.1|:20556-24039
NuwaII-4_CPB	gb|AHGY02084322.1|:14094-17980
NuwaII-4_CPB	gb|AHGY02237558.1|:6707-10162
NuwaII-4_CPB	gb|AHGY02214410.1|:1-2563
NuwaII-4_CPB	gb|AHGY02044953.1|:1981-5916
NuwaII-4_CPB	gb|AHGY02028326.1|:22717-27643
NuwaII-4_CPB	gb|AHGY02239448.1|:40930-45572
NuwaII-4_CPB	gb|AHGY02110679.1|:7171-11127
NuwaII-4_CPB	gb|AHGY02234830.1|:30753-32920
NuwaII-4_CPB	gb|AHGY02039133.1|:7404-11082
NuwaII-4_CPB	gb|AHGY02031362.1|:7769-11697
NuwaII-4_CPB	gb|AHGY02052881.1|:34191-37686
NuwaII-4_CPB	gb|AHGY02052881.1|:9103-9791
NuwaII-4_CPB	gb|AHGY02218703.1|:3662-7683
NuwaII-4_CPB	gb|AHGY02065298.1|:4859-8255
NuwaII-4_CPB	gb|AHGY02210088.1|:1749-5751
NuwaII-4_CPB	gb|AHGY02023196.1|:23054-26567
NuwaII-4_CPB	gb|AHGY02068485.1|:17363-20779
NuwaII-4_CPB	gb|AHGY02009560.1|:1249-5565
NuwaII-4_CPB	gb|AHGY02196252.1|:48928-48991
NuwaII-4_CPB	gb|AHGY02196252.1|:10214-14033
NuwaII-4_CPB	gb|AHGY02046875.1|:268-5498
NuwaII-4_CPB	gb|AHGY02249939.1|:27386-31926
NuwaII-4_CPB	gb|AHGY02196245.1|:83-3041
NuwaII-4_CPB	gb|AHGY02043075.1|:34981-39304
NuwaII-4_CPB	gb|AHGY02241355.1|:19229-19446
NuwaII-4_CPB	gb|AHGY02241355.1|:1515-5663
NuwaII-4_CPB	gb|AHGY02200659.1|:29579-33750
NuwaII-4_CPB	gb|AHGY02100066.1|:1106-4959
NuwaII-4_CPB	gb|AHGY02206833.1|:5970-10216
NuwaII-4_CPB	gb|AHGY02084153.1|:67798-72044
NuwaII-4_CPB	gb|AHGY02198795.1|:583-3073
NuwaII-4_CPB	gb|AHGY02106923.1|:1555-4067

NuwaII-4_CPB	gb|AHGY02238721.1|:14860-18973
NuwaII-4_CPB	gb|AHGY02198146.1|:20-2311
NuwaII-4_CPB	gb|AHGY02047322.1|:27864-31115
NuwaII-4_CPB	gb|AHGY02087765.1|:1454-6424
NuwaII-4_CPB	gb|AHGY02011125.1|:3-271
NuwaII-4_CPB	gb|AHGY02011125.1|:13734-17932
NuwaII-4_CPB	gb|AHGY02194864.1|:65-2196
NuwaII-4_CPB	gb|AHGY02014792.1|:7135-11611
NuwaII-4_CPB	gb|AHGY02223285.1|:13358-18012
NuwaII-4_CPB	gb|AHGY02044628.1|:1466-6038
NuwaII-4_CPB	gb|AHGY02202715.1|:32830-37613
NuwaII-4_CPB	gb|AHGY02055909.1|:7903-12113
NuwaII-4_CPB	gb|AHGY02196127.1|:3123-7197
NuwaII-4_CPB	gb|AHGY02062392.1|:6140-10161
NuwaII-4_CPB	gb|AHGY02064190.1|:8954-11823
NuwaII-4_CPB	gb|AHGY02095826.1|:12024-16888
NuwaII-4_CPB	gb|AHGY02210767.1|:30690-34611
NuwaII-4_CPB	gb|AHGY02235720.1|:114-2654
NuwaII-4_CPB	gb|AHGY02118825.1|:595-4717
NuwaII-4_CPB	gb|AHGY02076370.1|:404-4404
NuwaII-4_CPB	gb|AHGY02245731.1|:2671-6490
NuwaII-4_CPB	gb|AHGY02236577.1|:32093-35474
NuwaII-4_CPB	gb|AHGY02027803.1|:2-3750
NuwaII-4_CPB	gb|AHGY02052531.1|:3433-8414
NuwaII-4_CPB	gb|AHGY02246286.1|:4831-9332
NuwaII-4_CPB	gb|AHGY02097472.1|:9144-13076
NuwaII-4_CPB	gb|AHGY02089959.1|:32388-36346
NuwaII-4_CPB	gb|AHGY02066658.1|:1651-6353
NuwaII-4_CPB	gb|AHGY02066658.1|:12719-16675
NuwaII-4_CPB	gb|AHGY02239541.1|:2455-6279
NuwaII-4_CPB	gb|AHGY02039631.1|:26908-31375
NuwaII-4_CPB	gb|AHGY02039631.1|:42599-45683
NuwaII-4_CPB	gb|AHGY02090059.1|:15708-18068
NuwaII-4_CPB	gb|AHGY02087659.1|:1519-5563
NuwaII-4_CPB	gb|AHGY02216124.1|:7456-11789
NuwaII-4_CPB	gb|AHGY02036807.1|:7910-11633
NuwaII-4_CPB	gb|AHGY02012927.1|:15624-19874
NuwaII-4_CPB	gb|AHGY02218895.1|:25816-30378
NuwaII-4_CPB	gb|AHGY02062819.1|:21728-25991
NuwaII-4_CPB	gb|AHGY02032428.1|:62375-66327
NuwaII-4_CPB	gb|AHGY02100441.1|:11196-15329
NuwaII-4_CPB	gb|AHGY02247627.1|:15877-19963
NuwaII-4_CPB	gb|AHGY02049919.1|:18201-22286
NuwaII-4_CPB	gb|AHGY02224185.1|:17348-18437

NuwaII-4_CPB	gb|AHGY02224185.1|:16710-22337
NuwaII-4_CPB	gb|AHGY02035179.1|:33944-37994
NuwaII-4_CPB	gb|AHGY02035179.1|:14250-14306
NuwaII-4_CPB	gb|AHGY02074972.1|:19678-23588
NuwaII-4_CPB	gb|AHGY02008870.1|:6640-10993
NuwaII-4_CPB	gb|AHGY02232627.1|:10579-12492
NuwaII-4_CPB	gb|AHGY02195189.1|:6-2030
NuwaII-4_CPB	gb|AHGY02081464.1|:9982-14640
NuwaII-4_CPB	gb|AHGY02107866.1|:677-4659
NuwaII-4_CPB	gb|AHGY02107866.1|:15686-20225
NuwaII-4_CPB	gb|AHGY02229853.1|:1095-4008
NuwaII-4_CPB	gb|AHGY02033492.1|:833-2557
NuwaII-4_CPB	gb|AHGY02032087.1|:3837-7320
NuwaII-4_CPB	gb|AHGY02073866.1|:1350-5452
NuwaII-4_CPB	gb|AHGY02224038.1|:10954-14762
NuwaII-4_CPB	gb|AHGY02036066.1|:24909-28960
NuwaII-4_CPB	gb|AHGY02260999.1|:27478-31473
NuwaII-4_CPB	gb|AHGY02258218.1|:1598-5266
NuwaII-4_CPB	gb|AHGY02196350.1|:88698-92802
NuwaII-4_CPB	gb|AHGY02253602.1|:1981-3945
NuwaII-4_CPB	gb|AHGY02101023.1|:5989-9973
NuwaII-4_CPB	gb|AHGY02253217.1|:34729-38109
NuwaII-4_CPB	gb|AHGY02252661.1|:470-3161
NuwaII-4_CPB	gb|AHGY02044747.1|:25286-29098
NuwaII-4_CPB	gb|AHGY02044747.1|:31868-31993
NuwaII-4_CPB	gb|AHGY02047977.1|:5558-8903
NuwaII-4_CPB	gb|AHGY02047977.1|:189-2593
NuwaII-4_CPB	gb|AHGY02062608.1|:11659-15522
NuwaII-4_CPB	gb|AHGY02062608.1|:23826-25168
NuwaII-4_CPB	gb|AHGY02062608.1|:5284-8357
NuwaII-4_CPB	gb|AHGY02136829.1|:37202-37358
NuwaII-4_CPB	gb|AHGY02136829.1|:31447-37108
NuwaII-4_CPB	gb|AHGY02136829.1|:11502-15118
NuwaII-4_CPB	gb|AHGY02078873.1|:14071-17816
NuwaII-4_CPB	gb|AHGY02035115.1|:7340-11264
NuwaII-4_CPB	gb|AHGY02035115.1|:13871-15179
NuwaII-4_CPB	gb|AHGY02229406.1|:385-3422
NuwaII-4_CPB	gb|AHGY02078600.1|:19069-23152
NuwaII-4_CPB	gb|AHGY02012215.1|:16455-20718
NuwaII-4_CPB	gb|AHGY02220896.1|:32522-38833
NuwaII-4_CPB	gb|AHGY02213409.1|:1139-5208
NuwaII-4_CPB	gb|AHGY02072101.1|:28946-29102
NuwaII-4_CPB	gb|AHGY02072101.1|:1436-5598
NuwaII-4_CPB	gb|AHGY02052915.1|:2974-6958

NuwaII-4_CPB	gb|AHGY02231345.1|:1335-5101
NuwaII-4_CPB	gb|AHGY02244229.1|:14269-18461
NuwaII-4_CPB	gb|AHGY02103205.1|:1541-3864
NuwaII-4_CPB	gb|AHGY02044766.1|:5534-9946
NuwaII-4_CPB	gb|AHGY02216693.1|:9887-13924
NuwaII-4_CPB	gb|AHGY02091886.1|:4017-6284
NuwaII-4_CPB	gb|AHGY02081193.1|:24669-27796
NuwaII-4_CPB	gb|AHGY02081193.1|:29242-33265
NuwaII-4_CPB	gb|AHGY02011159.1|:3351-7442
NuwaII-4_CPB	gb|AHGY02240712.1|:354-3212
NuwaII-4_CPB	gb|AHGY02135740.1|:1-1419
NuwaII-4_CPB	gb|AHGY02073745.1|:304-4243
NuwaII-4_CPB	gb|AHGY02020972.1|:13520-17601
NuwaII-4_CPB	gb|AHGY02087258.1|:14719-18577
NuwaII-4_CPB	gb|AHGY02224111.1|:16509-21139
NuwaII-4_CPB	gb|AHGY02224111.1|:12820-13388
NuwaII-4_CPB	gb|AHGY02227515.1|:256-4268
NuwaII-4_CPB	gb|AHGY02250423.1|:27466-31360
NuwaII-4_CPB	gb|AHGY02250423.1|:2437-2513
NuwaII-4_CPB	gb|AHGY02102318.1|:5349-9497
NuwaII-4_CPB	gb|AHGY02236490.1|:27646-32888
NuwaII-4_CPB	gb|AHGY02109928.1|:1534-3168
NuwaII-4_CPB	gb|AHGY02102519.1|:5997-9979
NuwaII-4_CPB	gb|AHGY02038359.1|:8429-12167
NuwaII-4_CPB	gb|AHGY02104516.1|:1-2753
NuwaII-4_CPB	gb|AHGY02088760.1|:3752-7788
NuwaII-4_CPB	gb|AHGY02201565.1|:29144-31656
NuwaII-4_CPB	gb|AHGY02029293.1|:12191-15955
NuwaII-4_CPB	gb|AHGY02117566.1|:1-2306
NuwaII-4_CPB	gb|AHGY02208046.1|:771-4969
NuwaII-4_CPB	gb|AHGY02039312.1|:17683-21858
NuwaII-4_CPB	gb|AHGY02021000.1|:2325-6895
NuwaII-4_CPB	gb|AHGY02078346.1|:37102-42573
NuwaII-4_CPB	gb|AHGY02078346.1|:37731-38592
NuwaII-4_CPB	gb|AHGY02236016.1|:4968-9109
NuwaII-4_CPB	gb|AHGY02232190.1|:19700-23383
NuwaII-4_CPB	gb|AHGY02249603.1|:1113-5506
NuwaII-4_CPB	gb|AHGY02118084.1|:5-2200
NuwaII-4_CPB	gb|AHGY02238735.1|:2366-4168
NuwaII-4_CPB	gb|AHGY02114205.1|:924-5083
NuwaII-4_CPB	gb|AHGY02036169.1|:11256-15501
NuwaII-4_CPB	gb|AHGY02204601.1|:48054-51595
NuwaII-4_CPB	gb|AHGY02257353.1|:3825-5691
NuwaII-4_CPB	gb|AHGY02060080.1|:2436-4931

NuwaII-4_CPB	gb|AHGY02091325.1|:230-3066
NuwaII-4_CPB	gb|AHGY02052044.1|:16597-20790
NuwaII-4_CPB	gb|AHGY02028906.1|:183-2349
NuwaII-4_CPB	gb|AHGY02257451.1|:24338-29204
NuwaII-4_CPB	gb|AHGY02257451.1|:39267-43503
NuwaII-4_CPB	gb|AHGY02235039.1|:26818-31869
NuwaII-4_CPB	gb|AHGY02221873.1|:10279-14297
NuwaII-4_CPB	gb|AHGY02106778.1|:4340-8581
NuwaII-4_CPB	gb|AHGY02014858.1|:1836-6687
NuwaII-4_CPB	gb|AHGY02170614.1|:1928-3407
NuwaII-4_CPB	gb|AHGY02049830.1|:14707-18939
NuwaII-4_CPB	gb|AHGY02195746.1|:12606-15137
NuwaII-4_CPB	gb|AHGY02110327.1|:2-3145
NuwaII-4_CPB	gb|AHGY02099855.1|:3655-8326
NuwaII-4_CPB	gb|AHGY02055910.1|:26754-26875
NuwaII-4_CPB	gb|AHGY02055910.1|:6957-10615
NuwaII-4_CPB	gb|AHGY02237595.1|:3692-8614
NuwaII-4_CPB	gb|AHGY02004461.1|:28565-32310
NuwaII-4_CPB	gb|AHGY02052205.1|:9748-14013
NuwaII-4_CPB	gb|AHGY02195903.1|:25540-29634
NuwaII-4_CPB	gb|AHGY02110470.1|:1-2328
NuwaII-4_CPB	gb|AHGY02252369.1|:281-1245
NuwaII-4_CPB	gb|AHGY02252369.1|:10230-13056
NuwaII-4_CPB	gb|AHGY02248671.1|:277-2452
NuwaII-4_CPB	gb|AHGY02119746.1|:2426-2483
NuwaII-4_CPB	gb|AHGY02119746.1|:662-2725
NuwaII-4_CPB	gb|AHGY02043602.1|:73864-77236
NuwaII-4_CPB	gb|AHGY02043602.1|:22238-22332
NuwaII-4_CPB	gb|AHGY02029134.1|:19098-23674
NuwaII-4_CPB	gb|AHGY02084778.1|:5642-10478
NuwaII-4_CPB	gb|AHGY02084778.1|:15503-15616
NuwaII-4_CPB	gb|AHGY02061757.1|:4845-8559
NuwaII-4_CPB	gb|AHGY02063001.1|:2773-6957
NuwaII-4_CPB	gb|AHGY02005141.1|:4847-9768
NuwaII-4_CPB	gb|AHGY02231014.1|:30050-34282
NuwaII-4_CPB	gb|AHGY02114167.1|:1511-2278
NuwaII-4_CPB	gb|AHGY02114167.1|:5-2968
NuwaII-4_CPB	gb|AHGY02074667.1|:11178-15805
NuwaII-4_CPB	gb|AHGY02233593.1|:1014-1224
NuwaII-4_CPB	gb|AHGY02233593.1|:22527-26929
NuwaII-4_CPB	gb|AHGY02225559.1|:8183-11526
NuwaII-4_CPB	gb|AHGY02046806.1|:27-2797
NuwaII-4_CPB	gb|AHGY02056586.1|:4643-8856
NuwaII-4_CPB	gb|AHGY02074467.1|:23684-28587

NuwaII-4_CPB	gb|AHGY02045780.1|:1741-8313
NuwaII-4_CPB	gb|AHGY02045780.1|:3446-4292
NuwaII-4_CPB	gb|AHGY02199854.1|:24156-28353
NuwaII-4_CPB	gb|AHGY02220991.1|:5374-9477
NuwaII-4_CPB	gb|AHGY02089070.1|:397-2388
NuwaII-4_CPB	gb|AHGY02098629.1|:19630-23744
NuwaII-4_CPB	gb|AHGY02249088.1|:13-1561
NuwaII-4_CPB	gb|AHGY02208194.1|:52559-56704
NuwaII-4_CPB	gb|AHGY02114816.1|:3899-6388
NuwaII-4_CPB	gb|AHGY02236443.1|:4853-8778
NuwaII-4_CPB	gb|AHGY02056495.1|:26-3471
NuwaII-4_CPB	gb|AHGY02029342.1|:14577-18813
NuwaII-4_CPB	gb|AHGY02038382.1|:124680-126559
NuwaII-4_CPB	gb|AHGY02038382.1|:62975-67129
NuwaII-4_CPB	gb|AHGY02029058.1|:9408-14418
NuwaII-4_CPB	gb|AHGY02047936.1|:1-1671
NuwaII-4_CPB	gb|AHGY02219343.1|:221-3605
NuwaII-4_CPB	gb|AHGY02201719.1|:7151-7215
NuwaII-4_CPB	gb|AHGY02201719.1|:13810-17904
NuwaII-4_CPB	gb|AHGY02084662.1|:8860-12426
NuwaII-4_CPB	gb|AHGY02100799.1|:52068-53949
NuwaII-4_CPB	gb|AHGY02100799.1|:49829-52013
NuwaII-4_CPB	gb|AHGY02092044.1|:2329-5601
NuwaII-4_CPB	gb|AHGY02077374.1|:118-1912
NuwaII-4_CPB	gb|AHGY02074831.1|:4454-8611
NuwaII-4_CPB	gb|AHGY02108990.1|:21-4991
NuwaII-4_CPB	gb|AHGY02100070.1|:2228-4762
NuwaII-4_CPB	gb|AHGY02221097.1|:26400-28371
NuwaII-4_CPB	gb|AHGY02080198.1|:9741-13962
NuwaII-4_CPB	gb|AHGY02047770.1|:9267-12560
NuwaII-4_CPB	gb|AHGY02035999.1|:1221-6094
NuwaII-4_CPB	gb|AHGY02253428.1|:59240-62625
NuwaII-4_CPB	gb|AHGY02096938.1|:14798-19441
NuwaII-4_CPB	gb|AHGY02068362.1|:23702-27494
NuwaII-4_CPB	gb|AHGY02035013.1|:44127-46899
NuwaII-4_CPB	gb|AHGY02247081.1|:18375-22666
NuwaII-4_CPB	gb|AHGY02110369.1|:3-3746
NuwaII-4_CPB	gb|AHGY02072511.1|:5259-5444
NuwaII-4_CPB	gb|AHGY02072511.1|:1-2450
NuwaII-4_CPB	gb|AHGY02013523.1|:137-3619
NuwaII-4_CPB	gb|AHGY02257831.1|:17650-22254
NuwaII-4_CPB	gb|AHGY02244988.1|:1-1932
NuwaII-4_CPB	gb|AHGY02200310.1|:11742-13121
NuwaII-4_CPB	gb|AHGY02105979.1|:1203-4955

NuwaII-4_CPB	gb|AHGY02096913.1|:9782-13861
NuwaII-4_CPB	gb|AHGY02253796.1|:36-2593
NuwaII-4_CPB	gb|AHGY02221098.1|:5-1280
NuwaII-4_CPB	gb|AHGY02132194.1|:6-1672
NuwaII-4_CPB	gb|AHGY02077019.1|:152-2334
NuwaII-4_CPB	gb|AHGY02067331.1|:456-4084
NuwaII-4_CPB	gb|AHGY02062114.1|:1-4080
NuwaII-4_CPB	gb|AHGY02251025.1|:48-1690
NuwaII-4_CPB	gb|AHGY02235187.1|:13281-17480
NuwaII-4_CPB	gb|AHGY02077966.1|:5902-8447
NuwaII-4_CPB	gb|AHGY02223418.1|:219-2150
NuwaII-4_CPB	gb|AHGY02024583.1|:4-1171
NuwaII-4_CPB	gb|AHGY02235979.1|:13367-17713
NuwaII-4_CPB	gb|AHGY02020985.1|:5693-9909
NuwaII-4_CPB	gb|AHGY02079054.1|:6854-11136
NuwaII-4_CPB	gb|AHGY02256731.1|:10256-14843
NuwaII-4_CPB	gb|AHGY02085001.1|:28710-32887
NuwaII-4_CPB	gb|AHGY02083322.1|:461-3395
NuwaII-4_CPB	gb|AHGY02047358.1|:2138-4943
NuwaII-4_CPB	gb|AHGY02239339.1|:23642-25651
NuwaII-4_CPB	gb|AHGY02219338.1|:6810-10871
NuwaII-4_CPB	gb|AHGY02219338.1|:45735-47499
NuwaII-4_CPB	gb|AHGY02004209.1|:283-2832
NuwaII-5_CPB	gb|AHGY02112583.1|:631-3632
NuwaII-5_CPB	gb|AHGY02253865.1|:12550-16492
NuwaII-5_CPB	gb|AHGY02084697.1|:9010-12986
NuwaII-5_CPB	gb|AHGY02084697.1|:54413-54472
NuwaII-5_CPB	gb|AHGY02084697.1|:48394-48488
NuwaII-5_CPB	gb|AHGY02084739.1|:11815-15982
NuwaII-5_CPB	gb|AHGY02073837.1|:3924-7854
NuwaII-5_CPB	gb|AHGY02063000.1|:24772-28298
NuwaII-5_CPB	gb|AHGY02063000.1|:2386-2599
NuwaII-5_CPB	gb|AHGY02072020.1|:1-3887
NuwaII-5_CPB	gb|AHGY02097401.1|:2-2782
NuwaII-5_CPB	gb|AHGY02071178.1|:61311-65273
NuwaII-5_CPB	gb|AHGY02059148.1|:12720-15273
NuwaII-5_CPB	gb|AHGY02252145.1|:25036-25526
NuwaII-5_CPB	gb|AHGY02252145.1|:15493-19500
NuwaII-5_CPB	gb|AHGY02262159.1|:35933-36026
NuwaII-5_CPB	gb|AHGY02262159.1|:50256-50441
NuwaII-5_CPB	gb|AHGY02262159.1|:95801-95865
NuwaII-5_CPB	gb|AHGY02262159.1|:78618-82340
NuwaII-5_CPB	gb|AHGY02262159.1|:23600-23661
NuwaII-5_CPB	gb|AHGY02027047.1|:8144-10646

NuwaII-5_CPB	gb|AHGY02262225.1|:50743-50999
NuwaII-5_CPB	gb|AHGY02262225.1|:78387-82323
NuwaII-5_CPB	gb|AHGY02063067.1|:37-2182
NuwaII-5_CPB	gb|AHGY02031079.1|:84609-87643
NuwaII-5_CPB	gb|AHGY02042899.1|:7013-7186
NuwaII-5_CPB	gb|AHGY02042899.1|:34252-34413
NuwaII-5_CPB	gb|AHGY02042899.1|:30421-30697
NuwaII-5_CPB	gb|AHGY02042899.1|:196-2525
NuwaII-5_CPB	gb|AHGY02028916.1|:3866-5657
NuwaII-5_CPB	gb|AHGY02251079.1|:1599-5772
NuwaII-5_CPB	gb|AHGY02240100.1|:1-2155
NuwaII-5_CPB	gb|AHGY02216079.1|:3-2289
NuwaII-5_CPB	gb|AHGY02091533.1|:13902-16212
NuwaII-5_CPB	gb|AHGY02054134.1|:5373-9294
NuwaII-5_CPB	gb|AHGY02052614.1|:2-1829
NuwaII-5_CPB	gb|AHGY02028808.1|:4510-8071
NuwaII-5_CPB	gb|AHGY02060992.1|:10782-13477
NuwaII-5_CPB	gb|AHGY02217328.1|:10120-14040
NuwaII-5_CPB	gb|AHGY02069631.1|:300-2111
NuwaII-5_CPB	gb|AHGY02102537.1|:27-2196
NuwaII-5_CPB	gb|AHGY02214204.1|:16548-20508
NuwaII-5_CPB	gb|AHGY02214204.1|:13854-13916
NuwaII-5_CPB	gb|AHGY02207969.1|:19310-23254
NuwaII-5_CPB	gb|AHGY02207969.1|:6506-6583
NuwaII-5_CPB	gb|AHGY02204492.1|:49-2225
NuwaII-5_CPB	gb|AHGY02038028.1|:48737-52690
NuwaII-5_CPB	gb|AHGY02038028.1|:10981-11162
NuwaII-5_CPB	gb|AHGY02097519.1|:1107-2953
NuwaII-5_CPB	gb|AHGY02259418.1|:29285-31307
NuwaII-5_CPB	gb|AHGY02028893.1|:7-950
NuwaII-5_CPB	gb|AHGY02028893.1|:3836-7799
NuwaII-5_CPB	gb|AHGY02207776.1|:18-2624
NuwaII-5_CPB	gb|AHGY02034307.1|:6485-10373
NuwaII-5_CPB	gb|AHGY02251163.1|:19128-19223
NuwaII-5_CPB	gb|AHGY02251163.1|:47733-49972
NuwaII-5_CPB	gb|AHGY02251163.1|:31050-31230
NuwaII-5_CPB	gb|AHGY02008394.1|:15618-18570
NuwaII-5_CPB	gb|AHGY02229989.1|:9-2380
NuwaII-5_CPB	gb|AHGY02052647.1|:22483-25343
NuwaII-5_CPB	gb|AHGY02090877.1|:18662-22636
NuwaII-5_CPB	gb|AHGY02232118.1|:13289-13374
NuwaII-5_CPB	gb|AHGY02232118.1|:26989-29354
NuwaII-5_CPB	gb|AHGY02008232.1|:11533-15490
NuwaII-5_CPB	gb|AHGY02008232.1|:169-794

NuwaII-5_CPB	gb|AHGY02024415.1|:15691-15855
NuwaII-5_CPB	gb|AHGY02024415.1|:18170-18360
NuwaII-5_CPB	gb|AHGY02024415.1|:53-2338
NuwaII-5_CPB	gb|AHGY02090934.1|:330-2105
NuwaII-5_CPB	gb|AHGY02029661.1|:38595-41041
NuwaII-5_CPB	gb|AHGY02206339.1|:31225-33589
NuwaII-5_CPB	gb|AHGY02206339.1|:18999-19085
NuwaII-5_CPB	gb|AHGY02206339.1|:7812-7992
NuwaII-5_CPB	gb|AHGY02034986.1|:34750-34923
NuwaII-5_CPB	gb|AHGY02034986.1|:71095-73416
NuwaII-5_CPB	gb|AHGY02101056.1|:3-1766
NuwaII-5_CPB	gb|AHGY02029657.1|:22059-24762
NuwaII-5_CPB	gb|AHGY02000711.1|:4816-8862
NuwaII-5_CPB	gb|AHGY02000711.1|:808-1081
NuwaII-5_CPB	gb|AHGY02076321.1|:9-2376
NuwaII-5_CPB	gb|AHGY02243903.1|:4595-6344
NuwaII-5_CPB	gb|AHGY02243903.1|:987-1063
NuwaII-5_CPB	gb|AHGY02235207.1|:211-2329
NuwaII-5_CPB	gb|AHGY02092160.1|:10351-12699
NuwaII-5_CPB	gb|AHGY02032248.1|:17707-19985
NuwaII-5_CPB	gb|AHGY02203810.1|:1244-3214
NuwaII-5_CPB	gb|AHGY02049866.1|:21280-21369
NuwaII-5_CPB	gb|AHGY02049866.1|:5-2017
NuwaII-5_CPB	gb|AHGY02090199.1|:5994-8267
NuwaII-5_CPB	gb|AHGY02090199.1|:1018-1127
NuwaII-5_CPB	gb|AHGY02257744.1|:1300-3180
NuwaII-5_CPB	gb|AHGY02260913.1|:9672-12574
NuwaII-5_CPB	gb|AHGY02235819.1|:31405-35331
NuwaII-5_CPB	gb|AHGY02210449.1|:3-1955
NuwaII-5_CPB	gb|AHGY02100902.1|:1-1839
NuwaII-5_CPB	gb|AHGY02061094.1|:77-3422
NuwaII-5_CPB	gb|AHGY02081431.1|:20729-20913
NuwaII-5_CPB	gb|AHGY02081431.1|:5602-9508
NuwaII-5_CPB	gb|AHGY02014731.1|:3566-6497
NuwaII-5_CPB	gb|AHGY02070005.1|:9512-13450
NuwaII-5_CPB	gb|AHGY02199866.1|:10859-14813
NuwaII-5_CPB	gb|AHGY02233440.1|:3951-7235
NuwaII-5_CPB	gb|AHGY02233440.1|:142-2575
NuwaII-5_CPB	gb|AHGY02019312.1|:1172-1362
NuwaII-5_CPB	gb|AHGY02019312.1|:47603-51509
NuwaII-5_CPB	gb|AHGY02001345.1|:53640-53719
NuwaII-5_CPB	gb|AHGY02001345.1|:42825-46729
NuwaII-5_CPB	gb|AHGY02224305.1|:1-1730
NuwaII-5_CPB	gb|AHGY02213396.1|:376-2738

NuwaII-5_CPB	gb|AHGY02061676.1|:3577-5560
NuwaII-5_CPB	gb|AHGY02050758.1|:4981-7303
NuwaII-5_CPB	gb|AHGY02073849.1|:535-3557
NuwaII-5_CPB	gb|AHGY02200150.1|:43-2106
NuwaII-5_CPB	gb|AHGY02013399.1|:229-4140
NuwaII-5_CPB	gb|AHGY02010675.1|:6026-7466
NuwaII-5_CPB	gb|AHGY02010675.1|:26187-28492
NuwaII-5_CPB	gb|AHGY02010675.1|:16293-16480
NuwaII-5_CPB	gb|AHGY02235691.1|:62-2414
NuwaII-5_CPB	gb|AHGY02096926.1|:5984-8222
NuwaII-5_CPB	gb|AHGY02096926.1|:4749-5377
NuwaII-5_CPB	gb|AHGY02007578.1|:150-2530
NuwaII-5_CPB	gb|AHGY02085283.1|:11281-11420
NuwaII-5_CPB	gb|AHGY02085283.1|:16563-20519
NuwaII-5_CPB	gb|AHGY02229429.1|:3740-3912
NuwaII-5_CPB	gb|AHGY02229429.1|:55249-55435
NuwaII-5_CPB	gb|AHGY02229429.1|:42362-44279
NuwaII-5_CPB	gb|AHGY02195474.1|:274-2014
NuwaII-5_CPB	gb|AHGY02195474.1|:29-209
NuwaII-5_CPB	gb|AHGY02073875.1|:5039-8999
NuwaII-5_CPB	gb|AHGY02058598.1|:27276-31201
NuwaII-5_CPB	gb|AHGY02219416.1|:67-320
NuwaII-5_CPB	gb|AHGY02219416.1|:6089-9943
NuwaII-5_CPB	gb|AHGY02013508.1|:1445-5676
NuwaII-5_CPB	gb|AHGY02013508.1|:9973-10067
NuwaII-5_CPB	gb|AHGY02062055.1|:38715-40753
NuwaII-5_CPB	gb|AHGY02029544.1|:48941-49036
NuwaII-5_CPB	gb|AHGY02029544.1|:62629-64971
NuwaII-5_CPB	gb|AHGY02259313.1|:1-115
NuwaII-5_CPB	gb|AHGY02259313.1|:263-4126
NuwaII-5_CPB	gb|AHGY02211160.1|:1-1996
NuwaII-5_CPB	gb|AHGY02254427.1|:8460-10606
NuwaII-5_CPB	gb|AHGY02100317.1|:7090-10698
NuwaII-5_CPB	gb|AHGY02061797.1|:28869-29150
NuwaII-5_CPB	gb|AHGY02061797.1|:8-3120
NuwaII-5_CPB	gb|AHGY02233486.1|:61-2955
NuwaII-5_CPB	gb|AHGY02218494.1|:13698-17634
NuwaII-5_CPB	gb|AHGY02001098.1|:3112-5005
NuwaII-5_CPB	gb|AHGY02218450.1|:4656-4809
NuwaII-5_CPB	gb|AHGY02218450.1|:9742-13704
NuwaII-5_CPB	gb|AHGY02081650.1|:7699-11639
NuwaII-5_CPB	gb|AHGY02049726.1|:59-2820
NuwaII-5_CPB	gb|AHGY02001776.1|:11220-11479
NuwaII-5_CPB	gb|AHGY02001776.1|:1-2063

NuwaII-5_CPB	gb|AHGY02011110.1|:1-2335
NuwaII-5_CPB	gb|AHGY02222879.1|:1453-5345
NuwaII-5_CPB	gb|AHGY02078559.1|:12021-13916
NuwaII-5_CPB	gb|AHGY02034457.1|:3330-7238
NuwaII-5_CPB	gb|AHGY02032059.1|:2952-3211
NuwaII-5_CPB	gb|AHGY02032059.1|:17-2327
NuwaII-5_CPB	gb|AHGY02262217.1|:52616-52685
NuwaII-5_CPB	gb|AHGY02262217.1|:34314-34497
NuwaII-5_CPB	gb|AHGY02262217.1|:3243-7178
NuwaII-5_CPB	gb|AHGY02237664.1|:24228-28169
NuwaII-5_CPB	gb|AHGY02015972.1|:1-1749
NuwaII-5_CPB	gb|AHGY02225224.1|:25871-26039
NuwaII-5_CPB	gb|AHGY02225224.1|:27992-31948
NuwaII-5_CPB	gb|AHGY02225224.1|:4774-4961
NuwaII-5_CPB	gb|AHGY02207999.1|:1363-4831
NuwaII-5_CPB	gb|AHGY02012657.1|:4907-7357
NuwaII-5_CPB	gb|AHGY02222883.1|:6972-10919
NuwaII-5_CPB	gb|AHGY02199945.1|:3960-7908
NuwaII-5_CPB	gb|AHGY02249883.1|:1748-3911
NuwaII-5_CPB	gb|AHGY02116597.1|:562-3454
NuwaII-5_CPB	gb|AHGY02260303.1|:24203-27978
NuwaII-5_CPB	gb|AHGY02111939.1|:2-2267
NuwaII-5_CPB	gb|AHGY02032119.1|:525-4477
NuwaII-5_CPB	gb|AHGY02071971.1|:1721-5630
NuwaII-5_CPB	gb|AHGY02083207.1|:44-2388
NuwaII-5_CPB	gb|AHGY02012474.1|:8495-12439
NuwaII-5_CPB	gb|AHGY02012474.1|:28181-28415
NuwaII-5_CPB	gb|AHGY02071646.1|:1-2249
NuwaII-5_CPB	gb|AHGY02195712.1|:22-2601
NuwaII-5_CPB	gb|AHGY02061318.1|:23-1917
NuwaII-5_CPB	gb|AHGY02030796.1|:477-4029
NuwaII-5_CPB	gb|AHGY02200076.1|:14600-14673
NuwaII-5_CPB	gb|AHGY02200076.1|:8201-12129
NuwaII-5_CPB	gb|AHGY02196380.1|:24342-27489
NuwaII-5_CPB	gb|AHGY02061466.1|:149-2572
NuwaII-5_CPB	gb|AHGY02235223.1|:1-1701
NuwaII-5_CPB	gb|AHGY02105713.1|:7183-7256
NuwaII-5_CPB	gb|AHGY02105713.1|:10948-14853
NuwaII-5_CPB	gb|AHGY02020843.1|:18898-22780
NuwaII-5_CPB	gb|AHGY02211653.1|:13-3077
NuwaII-5_CPB	gb|AHGY02239567.1|:8798-10582
NuwaII-5_CPB	gb|AHGY02029316.1|:8926-12852
NuwaII-5_CPB	gb|AHGY02011693.1|:59740-63660
NuwaII-5_CPB	gb|AHGY02034603.1|:13804-15496

NuwaII-5_CPB	gb|AHGY02034603.1|:8791-8926
NuwaII-5_CPB	gb|AHGY02058454.1|:2314-4446
NuwaII-5_CPB	gb|AHGY02231444.1|:27689-27776
NuwaII-5_CPB	gb|AHGY02231444.1|:1-1691
NuwaII-5_CPB	gb|AHGY02225723.1|:1734-4487
NuwaII-5_CPB	gb|AHGY02065164.1|:50536-53964
NuwaII-5_CPB	gb|AHGY02065164.1|:1356-1424
NuwaII-5_CPB	gb|AHGY02207337.1|:11603-15541
NuwaII-5_CPB	gb|AHGY02255629.1|:7808-10595
NuwaII-5_CPB	gb|AHGY02255629.1|:10447-10555
NuwaII-5_CPB	gb|AHGY02036140.1|:52472-56412
NuwaII-5_CPB	gb|AHGY02013615.1|:7907-7994
NuwaII-5_CPB	gb|AHGY02013615.1|:22530-26489
NuwaII-5_CPB	gb|AHGY02052981.1|:1-3525
NuwaII-5_CPB	gb|AHGY02052981.1|:20603-20685
NuwaII-5_CPB	gb|AHGY02052981.1|:36209-36356
NuwaII-5_CPB	gb|AHGY02230786.1|:3-2024
NuwaII-5_CPB	gb|AHGY02230199.1|:9-1908
NuwaII-5_CPB	gb|AHGY02205502.1|:15122-19072
NuwaII-5_CPB	gb|AHGY02205502.1|:23763-29709
NuwaII-5_CPB	gb|AHGY02099240.1|:44-2273
NuwaII-5_CPB	gb|AHGY02033489.1|:6716-10639
NuwaII-5_CPB	gb|AHGY02192555.1|:3-2304
NuwaII-5_CPB	gb|AHGY02080172.1|:4839-8556
NuwaII-5_CPB	gb|AHGY02260603.1|:43120-44532
NuwaII-5_CPB	gb|AHGY02260603.1|:38433-42364
NuwaII-5_CPB	gb|AHGY02133051.1|:1-1642
NuwaII-5_CPB	gb|AHGY02093940.1|:13236-13418
NuwaII-5_CPB	gb|AHGY02093940.1|:13983-17951
NuwaII-5_CPB	gb|AHGY02012327.1|:1-1861
NuwaII-5_CPB	gb|AHGY02188293.1|:72-2448
NuwaII-5_CPB	gb|AHGY02075237.1|:5540-9441
NuwaII-5_CPB	gb|AHGY02075237.1|:46448-46638
NuwaII-5_CPB	gb|AHGY02261018.1|:8192-9888
NuwaII-5_CPB	gb|AHGY02028425.1|:1285-3277
NuwaII-5_CPB	gb|AHGY02106235.1|:2-3405
NuwaII-5_CPB	gb|AHGY02015806.1|:44229-44298
NuwaII-5_CPB	gb|AHGY02015806.1|:35612-39436
NuwaII-5_CPB	gb|AHGY02238181.1|:9-2478
NuwaII-5_CPB	gb|AHGY02032341.1|:5371-5998
NuwaII-5_CPB	gb|AHGY02252318.1|:6594-8917
NuwaII-5_CPB	gb|AHGY02198610.1|:5035-7798
NuwaII-5_CPB	gb|AHGY02226098.1|:1-2308
NuwaII-5_CPB	gb|AHGY02196812.1|:13256-15432

NuwaII-5_CPB	gb|AHGY02081089.1|:2426-2524
NuwaII-5_CPB	gb|AHGY02081089.1|:86-2214
NuwaII-5_CPB	gb|AHGY02236664.1|:56390-56988
NuwaII-5_CPB	gb|AHGY02236664.1|:33789-37105
NuwaII-5_CPB	gb|AHGY02072559.1|:66-1741
NuwaII-5_CPB	gb|AHGY02234163.1|:6-1742
NuwaII-5_CPB	gb|AHGY02206854.1|:2753-6672
NuwaII-5_CPB	gb|AHGY02199819.1|:32278-34899
NuwaII-5_CPB	gb|AHGY02073219.1|:2515-5590
NuwaII-5_CPB	gb|AHGY02027198.1|:24533-28367
NuwaII-5_CPB	gb|AHGY02251742.1|:25656-25753
NuwaII-5_CPB	gb|AHGY02251742.1|:48500-48588
NuwaII-5_CPB	gb|AHGY02251742.1|:4585-8487
NuwaII-5_CPB	gb|AHGY02085253.1|:5130-5536
NuwaII-5_CPB	gb|AHGY02085253.1|:21686-25622
NuwaII-5_CPB	gb|AHGY02087197.1|:59-2411
NuwaII-5_CPB	gb|AHGY02011942.1|:109-3244
NuwaII-5_CPB	gb|AHGY02207660.1|:26789-26961
NuwaII-5_CPB	gb|AHGY02207660.1|:37325-37512
NuwaII-5_CPB	gb|AHGY02207660.1|:17239-21148
NuwaII-5_CPB	gb|AHGY02207660.1|:1396-1588
NuwaII-5_CPB	gb|AHGY02222311.1|:1808-5784
NuwaII-5_CPB	gb|AHGY02082421.1|:29-2812
NuwaII-5_CPB	gb|AHGY02014725.1|:9177-9445
NuwaII-5_CPB	gb|AHGY02014725.1|:24142-24326
NuwaII-5_CPB	gb|AHGY02014725.1|:48594-48771
NuwaII-5_CPB	gb|AHGY02014725.1|:45977-46066
NuwaII-5_CPB	gb|AHGY02014725.1|:53-3224
NuwaII-5_CPB	gb|AHGY02252848.1|:9650-13520
NuwaII-5_CPB	gb|AHGY02221170.1|:1-1632
NuwaII-5_CPB	gb|AHGY02020840.1|:9132-11314
NuwaII-5_CPB	gb|AHGY02031415.1|:25-2530
NuwaII-5_CPB	gb|AHGY02000360.1|:1282-5190
NuwaII-5_CPB	gb|AHGY02045539.1|:31257-32867
NuwaII-5_CPB	gb|AHGY02079409.1|:2614-4185
NuwaII-5_CPB	gb|AHGY02112516.1|:22-3271
NuwaII-5_CPB	gb|AHGY02222711.1|:5406-7013
NuwaII-5_CPB	gb|AHGY02218563.1|:6062-6133
NuwaII-5_CPB	gb|AHGY02218563.1|:1-1864
NuwaII-5_CPB	gb|AHGY02203437.1|:6-3118
NuwaII-5_CPB	gb|AHGY02044121.1|:643-4465
NuwaII-5_CPB	gb|AHGY02253801.1|:36657-40580
NuwaII-5_CPB	gb|AHGY02198834.1|:2130-2186
NuwaII-5_CPB	gb|AHGY02198834.1|:12420-14351

NuwaII-5_CPB	gb|AHGY02198834.1|:4701-4776
NuwaII-5_CPB	gb|AHGY02004434.1|:1-3734
NuwaII-5_CPB	gb|AHGY02004434.1|:38220-38852
NuwaII-5_CPB	gb|AHGY02015827.1|:73-2761
NuwaII-5_CPB	gb|AHGY02248447.1|:347-4276
NuwaII-5_CPB	gb|AHGY02118642.1|:2124-3719
NuwaII-5_CPB	gb|AHGY02056834.1|:2530-4053
NuwaII-5_CPB	gb|AHGY02069400.1|:1-2228
NuwaII-5_CPB	gb|AHGY02223410.1|:15539-17057
NuwaII-5_CPB	gb|AHGY02222759.1|:21017-22591
NuwaII-5_CPB	gb|AHGY02222759.1|:9319-13602
NuwaII-5_CPB	gb|AHGY02029746.1|:1-1598
NuwaII-5_CPB	gb|AHGY02075687.1|:2633-4215
NuwaII-5_CPB	gb|AHGY02075687.1|:1543-1592
NuwaII-5_CPB	gb|AHGY02082431.1|:743-2244
NuwaII-5_CPB	gb|AHGY02247069.1|:21918-22099
NuwaII-5_CPB	gb|AHGY02247069.1|:1548-5497
NuwaII-5_CPB	gb|AHGY02228179.1|:2794-6666
NuwaII-5_CPB	gb|AHGY02110642.1|:10378-12536
NuwaII-5_CPB	gb|AHGY02245680.1|:1-1528
NuwaII-5_CPB	gb|AHGY02043873.1|:2673-7387
NuwaII-5_CPB	gb|AHGY02054252.1|:5159-8811
NuwaII-5_CPB	gb|AHGY02054252.1|:30143-33272
NuwaII-5_CPB	gb|AHGY02054252.1|:244-346
NuwaII-5_CPB	gb|AHGY02261144.1|:1-1459
NuwaII-5_CPB	gb|AHGY02134113.1|:1-1545
NuwaII-5_CPB	gb|AHGY02085430.1|:10435-14332
NuwaII-5_CPB	gb|AHGY02233875.1|:1155-1357
NuwaII-5_CPB	gb|AHGY02233875.1|:19912-22154
NuwaII-5_CPB	gb|AHGY02237618.1|:15854-19801
NuwaII-5_CPB	gb|AHGY02237618.1|:64076-64356
NuwaII-5_CPB	gb|AHGY02075263.1|:1-1480
NuwaII-5_CPB	gb|AHGY02134223.1|:1-1534
NuwaII-5_CPB	gb|AHGY02099863.1|:1-1918
NuwaII-5_CPB	gb|AHGY02039846.1|:15505-15810
NuwaII-5_CPB	gb|AHGY02039846.1|:18-2709
NuwaII-5_CPB	gb|AHGY02223230.1|:34049-35527
NuwaII-5_CPB	gb|AHGY02223230.1|:24326-24569
NuwaII-5_CPB	gb|AHGY02049824.1|:42-1495
NuwaII-5_CPB	gb|AHGY02055963.1|:24-2966
NuwaII-5_CPB	gb|AHGY02069559.1|:1-1602
NuwaII-5_CPB	gb|AHGY02109778.1|:1-1705
NuwaII-5_CPB	gb|AHGY02133785.1|:1-1573
NuwaII-5_CPB	gb|AHGY02062632.1|:517-604

NuwaII-5_CPB	gb|AHGY02062632.1|:3424-7305
NuwaII-5_CPB	gb|AHGY02032332.1|:1-2050
NuwaII-5_CPB	gb|AHGY02025682.1|:3573-7221
NuwaII-5_CPB	gb|AHGY02025682.1|:2514-2698
NuwaII-5_CPB	gb|AHGY02249935.1|:1-1500
NuwaII-5_CPB	gb|AHGY02075185.1|:6270-7757
NuwaII-5_CPB	gb|AHGY02075185.1|:3368-3547
NuwaII-5_CPB	gb|AHGY02065565.1|:31013-32542
NuwaII-5_CPB	gb|AHGY02065565.1|:26110-26205
NuwaII-5_CPB	gb|AHGY02131982.1|:2-1749
NuwaII-5_CPB	gb|AHGY02036549.1|:1-1541
NuwaII-5_CPB	gb|AHGY02042836.1|:7089-8500
NuwaII-5_CPB	gb|AHGY02111319.1|:7-2861
NuwaII-5_CPB	gb|AHGY02087476.1|:1-1509
NuwaII-5_CPB	gb|AHGY02205574.1|:35792-38865
NuwaII-5_CPB	gb|AHGY02210389.1|:1-1409
NuwaII-5_CPB	gb|AHGY02235703.1|:1-1865
NuwaII-6_CPB	gb|AHGY02063377.1|:54412-58443
NuwaII-6_CPB	gb|AHGY02224787.1|:12741-16878
NuwaII-6_CPB	gb|AHGY02216637.1|:7679-12403
NuwaII-6_CPB	gb|AHGY02054855.1|:8044-12288
NuwaII-6_CPB	gb|AHGY02065151.1|:4840-7093
NuwaII-6_CPB	gb|AHGY02082618.1|:27497-27607
NuwaII-6_CPB	gb|AHGY02082618.1|:67-1850
NuwaII-6_CPB	gb|AHGY02226801.1|:273-2211
NuwaII-6_CPB	gb|AHGY02216387.1|:3023-7155
NuwaII-6_CPB	gb|AHGY02066759.1|:11598-14823
NuwaII-6_CPB	gb|AHGY02048005.1|:61-4161
NuwaII-6_CPB	gb|AHGY02232613.1|:4909-6549
NuwaII-6_CPB	gb|AHGY02074872.1|:8555-12707
NuwaII-6_CPB	gb|AHGY02209931.1|:15866-19997
NuwaII-6_CPB	gb|AHGY02209880.1|:9336-13595
NuwaII-6_CPB	gb|AHGY02196036.1|:910-2814
NuwaII-6_CPB	gb|AHGY02075133.1|:14476-17114
NuwaII-6_CPB	gb|AHGY02035416.1|:4646-6097
NuwaII-6_CPB	gb|AHGY02013704.1|:11981-16076
NuwaII-6_CPB	gb|AHGY02247158.1|:24277-27100
NuwaII-6_CPB	gb|AHGY02062648.1|:6466-10637
NuwaII-6_CPB	gb|AHGY02239311.1|:12220-16755
NuwaII-6_CPB	gb|AHGY02229830.1|:56031-60182
NuwaII-6_CPB	gb|AHGY02019697.1|:3620-7670
NuwaII-6_CPB	gb|AHGY02206876.1|:11008-14698
NuwaII-6_CPB	gb|AHGY02200880.1|:8210-8401
NuwaII-6_CPB	gb|AHGY02200880.1|:4013-8195

NuwaII-6_CPB	gb|AHGY02044597.1|:11411-14999
NuwaII-6_CPB	gb|AHGY02037125.1|:2-2729
NuwaII-6_CPB	gb|AHGY02216281.1|:17596-21613
NuwaII-6_CPB	gb|AHGY02216281.1|:103428-103732
NuwaII-6_CPB	gb|AHGY02216281.1|:21294-21345
NuwaII-6_CPB	gb|AHGY02034127.1|:1476-5491
NuwaII-6_CPB	gb|AHGY02212087.1|:5564-9658
NuwaII-6_CPB	gb|AHGY02000854.1|:8032-12900
NuwaII-6_CPB	gb|AHGY02228504.1|:7565-10115
NuwaII-6_CPB	gb|AHGY02027110.1|:59487-59929
NuwaII-6_CPB	gb|AHGY02027110.1|:44637-48656
NuwaII-6_CPB	gb|AHGY02241170.1|:18288-20805
NuwaII-6_CPB	gb|AHGY02101362.1|:9318-13390
NuwaII-6_CPB	gb|AHGY02101362.1|:23626-25400
NuwaII-6_CPB	gb|AHGY02004098.1|:45928-50088
NuwaII-6_CPB	gb|AHGY02032153.1|:9539-13626
NuwaII-6_CPB	gb|AHGY02106870.1|:33346-36236
NuwaII-6_CPB	gb|AHGY02095841.1|:17602-19116
NuwaII-6_CPB	gb|AHGY02095513.1|:36727-40469
NuwaII-6_CPB	gb|AHGY02243397.1|:12874-16899
NuwaII-6_CPB	gb|AHGY02230567.1|:8158-12167
NuwaII-6_CPB	gb|AHGY02019520.1|:31778-35887
NuwaII-6_CPB	gb|AHGY02200963.1|:11395-15498
NuwaII-6_CPB	gb|AHGY02076392.1|:3481-7630
NuwaII-6_CPB	gb|AHGY02022217.1|:2-3820
NuwaII-6_CPB	gb|AHGY02110070.1|:2911-6988
NuwaII-6_CPB	gb|AHGY02244216.1|:4085-8060
NuwaII-6_CPB	gb|AHGY02251284.1|:39400-43505
NuwaII-6_CPB	gb|AHGY02251284.1|:5276-6029
NuwaII-6_CPB	gb|AHGY02248997.1|:26581-30742
NuwaII-6_CPB	gb|AHGY02231179.1|:34-1461
NuwaII-6_CPB	gb|AHGY02231179.1|:17448-27123
NuwaII-6_CPB	gb|AHGY02231179.1|:22676-25691
NuwaII-6_CPB	gb|AHGY02042895.1|:7169-11264
NuwaII-6_CPB	gb|AHGY02015994.1|:2651-6719
NuwaII-6_CPB	gb|AHGY02013100.1|:14708-18840
NuwaII-6_CPB	gb|AHGY02013100.1|:15-1263
NuwaII-6_CPB	gb|AHGY02248242.1|:24090-28407
NuwaII-6_CPB	gb|AHGY02248242.1|:187-654
NuwaII-6_CPB	gb|AHGY02240203.1|:1992-3884
NuwaII-6_CPB	gb|AHGY02231019.1|:71058-71675
NuwaII-6_CPB	gb|AHGY02231019.1|:69718-74487
NuwaII-6_CPB	gb|AHGY02231019.1|:12305-13056
NuwaII-6_CPB	gb|AHGY02111000.1|:18968-21847

NuwaII-6_CPB	gb|AHGY02111000.1|:565-961
NuwaII-6_CPB	gb|AHGY02006783.1|:9690-13359
NuwaII-6_CPB	gb|AHGY02042550.1|:19154-22589
NuwaII-6_CPB	gb|AHGY02082662.1|:21539-26490
NuwaII-6_CPB	gb|AHGY02096867.1|:12290-16328
NuwaII-6_CPB	gb|AHGY02222176.1|:5900-10022
NuwaII-6_CPB	gb|AHGY02057130.1|:60-192
NuwaII-6_CPB	gb|AHGY02057130.1|:24566-26036
NuwaII-6_CPB	gb|AHGY02044617.1|:6198-10172
NuwaII-6_CPB	gb|AHGY02245215.1|:15312-17098
NuwaII-6_CPB	gb|AHGY02245215.1|:8-1474
NuwaII-6_CPB	gb|AHGY02261103.1|:36661-40262
NuwaII-6_CPB	gb|AHGY02202458.1|:5749-9804
NuwaII-6_CPB	gb|AHGY02012695.1|:6339-10695
NuwaII-6_CPB	gb|AHGY02228692.1|:49174-53463
NuwaII-6_CPB	gb|AHGY02216223.1|:5518-9337
NuwaII-6_CPB	gb|AHGY02219793.1|:37294-39134
NuwaII-6_CPB	gb|AHGY02219793.1|:2922-6102
NuwaII-6_CPB	gb|AHGY02205848.1|:12344-14702
NuwaII-6_CPB	gb|AHGY02248451.1|:18572-18765
NuwaII-6_CPB	gb|AHGY02248451.1|:6762-9633
NuwaII-6_CPB	gb|AHGY02213778.1|:18-2806
NuwaII-6_CPB	gb|AHGY02076423.1|:9992-11423
NuwaII-6_CPB	gb|AHGY02062548.1|:9631-9704
NuwaII-6_CPB	gb|AHGY02062548.1|:41698-46451
NuwaII-6_CPB	gb|AHGY02020845.1|:9667-13794
NuwaII-6_CPB	gb|AHGY02115148.1|:2-1695
NuwaII-6_CPB	gb|AHGY02030762.1|:2368-6504
NuwaII-6_CPB	gb|AHGY02025969.1|:51-1976
NuwaII-6_CPB	gb|AHGY02012236.1|:11470-15528
NuwaII-6_CPB	gb|AHGY02035895.1|:3301-7372
NuwaII-6_CPB	gb|AHGY02217246.1|:31287-34830
NuwaII-6_CPB	gb|AHGY02217246.1|:208-4316
NuwaII-6_CPB	gb|AHGY02027037.1|:1512-5499
NuwaII-6_CPB	gb|AHGY02203383.1|:2068-5417
NuwaII-6_CPB	gb|AHGY02243603.1|:2502-4663
NuwaII-6_CPB	gb|AHGY02243603.1|:48-242
NuwaII-6_CPB	gb|AHGY02222243.1|:4119-7365
NuwaII-6_CPB	gb|AHGY02006947.1|:7758-11930
NuwaII-6_CPB	gb|AHGY02261603.1|:49-2827
NuwaII-6_CPB	gb|AHGY02261140.1|:1585-5654
NuwaII-6_CPB	gb|AHGY02108330.1|:843-3372
NuwaII-6_CPB	gb|AHGY02229258.1|:7923-9934
NuwaII-6_CPB	gb|AHGY02117341.1|:3332-5204

NuwaII-6_CPB	gb|AHGY02044342.1|:33589-37383
NuwaII-6_CPB	gb|AHGY02091570.1|:213-3005
NuwaII-6_CPB	gb|AHGY02080968.1|:21334-24080
NuwaII-6_CPB	gb|AHGY02229302.1|:6121-10272
NuwaII-6_CPB	gb|AHGY02197647.1|:4808-10258
NuwaII-6_CPB	gb|AHGY02061468.1|:329-4450
NuwaII-6_CPB	gb|AHGY02009540.1|:3431-7481
NuwaII-6_CPB	gb|AHGY02236304.1|:1-1054
NuwaII-6_CPB	gb|AHGY02200621.1|:46376-50500
NuwaII-6_CPB	gb|AHGY02200621.1|:25212-33583
NuwaII-6_CPB	gb|AHGY02047970.1|:11522-15459
NuwaII-6_CPB	gb|AHGY02047970.1|:1226-6565
NuwaII-6_CPB	gb|AHGY02013564.1|:48-1712
NuwaII-6_CPB	gb|AHGY02013564.1|:12198-16102
NuwaII-6_CPB	gb|AHGY02221062.1|:37507-39414
NuwaII-6_CPB	gb|AHGY02033675.1|:920-3590
NuwaII-6_CPB	gb|AHGY02015591.1|:34471-37645
NuwaII-6_CPB	gb|AHGY02015591.1|:34753-35931
NuwaII-6_CPB	gb|AHGY02235023.1|:13784-17533
NuwaII-6_CPB	gb|AHGY02252467.1|:2148-5915
NuwaII-6_CPB	gb|AHGY02098706.1|:53-2622
NuwaII-6_CPB	gb|AHGY02043207.1|:1484-3333
NuwaII-6_CPB	gb|AHGY02010528.1|:21219-25318
NuwaII-6_CPB	gb|AHGY02203426.1|:5617-7972
NuwaII-6_CPB	gb|AHGY02229872.1|:29587-33596
NuwaII-6_CPB	gb|AHGY02229872.1|:28720-29367
NuwaII-6_CPB	gb|AHGY02208647.1|:3590-7726
NuwaII-6_CPB	gb|AHGY02089448.1|:20096-25758
NuwaII-6_CPB	gb|AHGY02054098.1|:8025-9386
NuwaII-6_CPB	gb|AHGY02038236.1|:1011-5050
NuwaII-6_CPB	gb|AHGY02025790.1|:39045-39427
NuwaII-6_CPB	gb|AHGY02025790.1|:5812-9576
NuwaII-6_CPB	gb|AHGY02240336.1|:56637-60741
NuwaII-6_CPB	gb|AHGY02049093.1|:53298-57125
NuwaII-6_CPB	gb|AHGY02074617.1|:5809-8307
NuwaII-6_CPB	gb|AHGY02108723.1|:3225-3347
NuwaII-6_CPB	gb|AHGY02108723.1|:3367-6736
NuwaII-6_CPB	gb|AHGY02064380.1|:6352-10523
NuwaII-6_CPB	gb|AHGY02216813.1|:2276-6404
NuwaII-6_CPB	gb|AHGY02198001.1|:25498-27422
NuwaII-6_CPB	gb|AHGY02108814.1|:385-3977
NuwaII-6_CPB	gb|AHGY02013624.1|:5-238
NuwaII-6_CPB	gb|AHGY02013624.1|:14410-16367
NuwaII-6_CPB	gb|AHGY02013055.1|:1596-5608

NuwaII-6_CPB	gb|AHGY02011736.1|:10405-11875
NuwaII-6_CPB	gb|AHGY02062513.1|:19573-24137
NuwaII-6_CPB	gb|AHGY02222942.1|:3151-6853
NuwaII-6_CPB	gb|AHGY02249609.1|:758-3649
NuwaII-6_CPB	gb|AHGY02229908.1|:71-2516
NuwaII-6_CPB	gb|AHGY02114375.1|:101-1483
NuwaII-6_CPB	gb|AHGY02098764.1|:3249-8207
NuwaII-6_CPB	gb|AHGY02056420.1|:11789-14344
NuwaII-6_CPB	gb|AHGY02025554.1|:4290-5809
NuwaII-6_CPB	gb|AHGY02218392.1|:2452-4360
NuwaII-6_CPB	gb|AHGY02109671.1|:5539-9348
NuwaII-6_CPB	gb|AHGY02036726.1|:837-5371
NuwaII-6_CPB	gb|AHGY02033903.1|:1454-5016
NuwaII-6_CPB	gb|AHGY02227557.1|:8896-9890
NuwaII-6_CPB	gb|AHGY02020018.1|:1007-4274
NuwaII-6_CPB	gb|AHGY02223061.1|:2393-6139
NuwaII-6_CPB	gb|AHGY02202531.1|:9824-9911
NuwaII-6_CPB	gb|AHGY02202531.1|:25557-29290
NuwaII-6_CPB	gb|AHGY02009748.1|:43-3051
NuwaII-6_CPB	gb|AHGY02223194.1|:1-1302
NuwaII-6_CPB	gb|AHGY02255281.1|:10328-14075
NuwaII-6_CPB	gb|AHGY02117109.1|:2145-4196
NuwaII-6_CPB	gb|AHGY02020951.1|:15926-19511
NuwaII-6_CPB	gb|AHGY02237512.1|:3363-7472
NuwaII-6_CPB	gb|AHGY02224612.1|:4895-6467
NuwaII-6_CPB	gb|AHGY02238765.1|:1121-5382
NuwaII-6_CPB	gb|AHGY02200644.1|:43405-46432
NuwaII-6_CPB	gb|AHGY02221032.1|:11726-12936
NuwaII-6_CPB	gb|AHGY02075403.1|:10859-14988
NuwaII-6_CPB	gb|AHGY02033657.1|:5395-7842
NuwaII-6_CPB	gb|AHGY02213921.1|:22183-26306
NuwaII-6_CPB	gb|AHGY02256472.1|:4923-8361
NuwaII-6_CPB	gb|AHGY02238547.1|:45144-49270
NuwaII-6_CPB	gb|AHGY02238547.1|:117-195
NuwaII-6_CPB	gb|AHGY02088770.1|:7801-10464
NuwaII-6_CPB	gb|AHGY02013102.1|:21808-24076
NuwaII-6_CPB	gb|AHGY02033674.1|:497-4311
NuwaII-6_CPB	gb|AHGY02232571.1|:17508-21151
NuwaII-6_CPB	gb|AHGY02223293.1|:7002-11048
NuwaII-6_CPB	gb|AHGY02200282.1|:832-4871
NuwaII-6_CPB	gb|AHGY02009569.1|:7933-9777
NuwaII-6_CPB	gb|AHGY02058574.1|:10883-14602
NuwaII-6_CPB	gb|AHGY02231339.1|:8706-11899
NuwaII-6_CPB	gb|AHGY02246374.1|:2788-5617

NuwaII-6_CPB	gb|AHGY02092741.1|:5409-7583
NuwaII-6_CPB	gb|AHGY02201692.1|:2661-5461
NuwaII-6_CPB	gb|AHGY02201692.1|:40259-44328
NuwaII-6_CPB	gb|AHGY02218312.1|:1-3964
NuwaII-6_CPB	gb|AHGY02213663.1|:9012-11253
NuwaII-6_CPB	gb|AHGY02091102.1|:16729-21556
NuwaII-6_CPB	gb|AHGY02088718.1|:15488-16790
NuwaII-6_CPB	gb|AHGY02225509.1|:579-1281
NuwaII-6_CPB	gb|AHGY02225509.1|:25627-29632
NuwaII-6_CPB	gb|AHGY02090605.1|:19521-22110
NuwaII-6_CPB	gb|AHGY02212150.1|:19648-23589
NuwaII-6_CPB	gb|AHGY02212150.1|:46585-47001
NuwaII-6_CPB	gb|AHGY02199458.1|:1-3229
NuwaII-6_CPB	gb|AHGY02225478.1|:86210-89292
NuwaII-6_CPB	gb|AHGY02222227.1|:23685-27848
NuwaII-6_CPB	gb|AHGY02076970.1|:56803-57881
NuwaII-6_CPB	gb|AHGY02076970.1|:17384-21511
NuwaII-6_CPB	gb|AHGY02059654.1|:2290-6450
NuwaII-6_CPB	gb|AHGY02030766.1|:34992-38985
NuwaII-6_CPB	gb|AHGY02253655.1|:12675-16190
NuwaII-6_CPB	gb|AHGY02019636.1|:14265-16489
NuwaII-6_CPB	gb|AHGY02232582.1|:2555-2831
NuwaII-6_CPB	gb|AHGY02232582.1|:5061-9463
NuwaII-6_CPB	gb|AHGY02117918.1|:81-1746
NuwaII-6_CPB	gb|AHGY02008870.1|:6727-10993
NuwaII-6_CPB	gb|AHGY02214848.1|:241-2597
NuwaII-6_CPB	gb|AHGY02093691.1|:8063-12303
NuwaII-6_CPB	gb|AHGY02084153.1|:67892-72044
NuwaII-6_CPB	gb|AHGY02012414.1|:20808-24715
NuwaII-6_CPB	gb|AHGY02069634.1|:2-1121
NuwaII-6_CPB	gb|AHGY02205833.1|:4963-6762
NuwaII-6_CPB	gb|AHGY02205898.1|:9907-13540
NuwaII-6_CPB	gb|AHGY02009744.1|:12033-13305
NuwaII-6_CPB	gb|AHGY02009744.1|:77-1386
NuwaII-6_CPB	gb|AHGY02082787.1|:214-4099
NuwaII-6_CPB	gb|AHGY02011895.1|:9851-13231
NuwaII-6_CPB	gb|AHGY02222202.1|:21599-25847
NuwaII-6_CPB	gb|AHGY02221488.1|:2357-6605
NuwaII-6_CPB	gb|AHGY02073142.1|:8726-11654
NuwaII-6_CPB	gb|AHGY02045680.1|:10572-15116
NuwaII-6_CPB	gb|AHGY02045680.1|:46-3756
NuwaII-6_CPB	gb|AHGY02209127.1|:3901-8512
NuwaII-6_CPB	gb|AHGY02051511.1|:1179-5285
NuwaII-6_CPB	gb|AHGY02029810.1|:8027-9253

NuwaII-6_CPB	gb|AHGY02248301.1|:14996-19038
NuwaII-6_CPB	gb|AHGY02037478.1|:2023-4830
NuwaII-6_CPB	gb|AHGY02037478.1|:4586-4684
NuwaII-6_CPB	gb|AHGY02251334.1|:2354-6463
NuwaII-6_CPB	gb|AHGY02243380.1|:86-1522
NuwaII-6_CPB	gb|AHGY02142754.1|:16507-20790
NuwaII-6_CPB	gb|AHGY02090010.1|:125-1746
NuwaII-6_CPB	gb|AHGY02252152.1|:5854-11974
NuwaII-6_CPB	gb|AHGY02240331.1|:1-2082
NuwaII-6_CPB	gb|AHGY02114647.1|:7584-9230
NuwaII-6_CPB	gb|AHGY02114647.1|:1649-3859
NuwaII-6_CPB	gb|AHGY02038182.1|:1481-6147
NuwaII-6_CPB	gb|AHGY02007902.1|:9225-13316
NuwaII-6_CPB	gb|AHGY02232627.1|:10580-12492
NuwaII-6_CPB	gb|AHGY02061755.1|:12236-16452
NuwaII-6_CPB	gb|AHGY02028326.1|:22804-27643
NuwaII-6_CPB	gb|AHGY02096181.1|:11318-15201
NuwaII-6_CPB	gb|AHGY02247627.1|:15971-19963
NuwaII-6_CPB	gb|AHGY02076289.1|:3261-7317
NuwaII-6_CPB	gb|AHGY02105442.1|:411-5536
NuwaII-6_CPB	gb|AHGY02088730.1|:6374-10007
NuwaII-6_CPB	gb|AHGY02072187.1|:9607-13120
NuwaII-6_CPB	gb|AHGY02261240.1|:5726-11419
NuwaII-6_CPB	gb|AHGY02221017.1|:3756-8172
NuwaII-6_CPB	gb|AHGY02036128.1|:16537-20155
NuwaII-6_CPB	gb|AHGY02238703.1|:1-4616
NuwaII-6_CPB	gb|AHGY02012090.1|:24868-27927
NuwaII-6_CPB	gb|AHGY02104595.1|:1469-3582
NuwaII-6_CPB	gb|AHGY02104595.1|:3585-4103
NuwaII-6_CPB	gb|AHGY02055909.1|:7903-12019
NuwaII-6_CPB	gb|AHGY02234404.1|:14799-15845
NuwaII-6_CPB	gb|AHGY02210557.1|:45041-45235
NuwaII-6_CPB	gb|AHGY02210557.1|:5756-9785
NuwaII-6_CPB	gb|AHGY02199891.1|:12003-16111
NuwaII-6_CPB	gb|AHGY02002325.1|:118-1650
NuwaII-6_CPB	gb|AHGY02213409.1|:1153-5208
NuwaII-6_CPB	gb|AHGY02110369.1|:330-3659
NuwaII-6_CPB	gb|AHGY02238640.1|:459-2348
NuwaII-6_CPB	gb|AHGY02252666.1|:227-1321
NuwaII-6_CPB	gb|AHGY02051703.1|:6681-12356
NuwaII-6_CPB	gb|AHGY02022599.1|:5453-9081
NuwaII-6_CPB	gb|AHGY02214410.1|:243-2563
NuwaII-6_CPB	gb|AHGY02031802.1|:3045-7158
NuwaII-6_CPB	gb|AHGY02198795.1|:735-3073

NuwaII-6_CPB	gb|AHGY02097255.1|:8151-9555
NuwaII-7_CPB	gb|AHGY02073732.1|:19075-23323
NuwaII-7_CPB	gb|AHGY02055491.1|:173821-177993
NuwaII-7_CPB	gb|AHGY02021950.1|:14508-18743
NuwaII-7_CPB	gb|AHGY02062969.1|:23874-26895
NuwaII-7_CPB	gb|AHGY02041033.1|:13018-16467
NuwaII-7_CPB	gb|AHGY02232150.1|:7693-11529
NuwaII-7_CPB	gb|AHGY02062510.1|:59966-62239
NuwaII-7_CPB	gb|AHGY02063268.1|:14641-18132
NuwaII-7_CPB	gb|AHGY02243903.1|:5173-5908
NuwaII-7_CPB	gb|AHGY02243903.1|:386-1970
NuwaII-7_CPB	gb|AHGY02157714.1|:1597-5538
NuwaII-7_CPB	gb|AHGY02206867.1|:16518-19400
NuwaII-7_CPB	gb|AHGY02082172.1|:24-1639
NuwaII-7_CPB	gb|AHGY02038268.1|:5054-10164
NuwaII-7_CPB	gb|AHGY02001580.1|:19938-21744
NuwaII-7_CPB	gb|AHGY02260231.1|:11916-16569
NuwaII-7_CPB	gb|AHGY02058999.1|:26310-30932
NuwaII-7_CPB	gb|AHGY02040617.1|:9709-13362
NuwaII-7_CPB	gb|AHGY02230782.1|:15908-20549
NuwaII-7_CPB	gb|AHGY02230254.1|:20527-24702
NuwaII-7_CPB	gb|AHGY02259413.1|:11393-15246
NuwaII-7_CPB	gb|AHGY02008751.1|:30143-33931
NuwaII-7_CPB	gb|AHGY02260955.1|:6472-10600
NuwaII-7_CPB	gb|AHGY02038041.1|:16-1975
NuwaII-7_CPB	gb|AHGY02046947.1|:1683-4953
NuwaII-7_CPB	gb|AHGY02063454.1|:13728-17777
NuwaII-7_CPB	gb|AHGY02069749.1|:226-4250
NuwaII-7_CPB	gb|AHGY02099796.1|:3660-7786
NuwaII-7_CPB	gb|AHGY02243621.1|:4800-8959
NuwaII-7_CPB	gb|AHGY02224280.1|:14681-18700
NuwaII-7_CPB	gb|AHGY02038127.1|:22394-26439
NuwaII-7_CPB	gb|AHGY02026913.1|:31671-36502
NuwaII-7_CPB	gb|AHGY02026913.1|:11744-12530
NuwaII-7_CPB	gb|AHGY02049478.1|:4402-8225
NuwaII-7_CPB	gb|AHGY02000462.1|:23349-24615
NuwaII-7_CPB	gb|AHGY02002947.1|:57-4131
NuwaII-7_CPB	gb|AHGY02026247.1|:1822-4511
NuwaII-7_CPB	gb|AHGY02234063.1|:21643-25382
NuwaII-7_CPB	gb|AHGY02225509.1|:169-2561
NuwaII-7_CPB	gb|AHGY02225509.1|:25822-29322
NuwaII-7_CPB	gb|AHGY02085403.1|:6212-8037
NuwaII-7_CPB	gb|AHGY02085403.1|:9199-10079
NuwaII-7_CPB	gb|AHGY02039106.1|:193-1635

NuwaII-7_CPB	gb|AHGY02033491.1|:11798-15538
NuwaII-7_CPB	gb|AHGY02213927.1|:22158-24298
NuwaII-7_CPB	gb|AHGY02043988.1|:10329-16471
NuwaII-7_CPB	gb|AHGY02229990.1|:10072-12639
NuwaII-7_CPB	gb|AHGY02008333.1|:34562-37846
NuwaII-7_CPB	gb|AHGY02048607.1|:26135-30238
NuwaII-7_CPB	gb|AHGY02060811.1|:774-3788
NuwaII-7_CPB	gb|AHGY02084020.1|:493-4406
NuwaII-7_CPB	gb|AHGY02225262.1|:19327-23385
NuwaII-7_CPB	gb|AHGY02231285.1|:1-4433
NuwaII-7_CPB	gb|AHGY02099192.1|:7363-10487
NuwaII-7_CPB	gb|AHGY02218885.1|:18171-19791
NuwaII-7_CPB	gb|AHGY02067286.1|:7024-10094
NuwaII-7_CPB	gb|AHGY02259536.1|:738-4771
NuwaII-7_CPB	gb|AHGY02259536.1|:35039-36169
NuwaII-7_CPB	gb|AHGY02208176.1|:126-2803
NuwaII-7_CPB	gb|AHGY02238823.1|:22972-25549
NuwaII-7_CPB	gb|AHGY02115344.1|:4310-7619
NuwaII-7_CPB	gb|AHGY02027171.1|:17673-17812
NuwaII-7_CPB	gb|AHGY02027171.1|:108-3912
NuwaII-7_CPB	gb|AHGY02039631.1|:27860-28891
NuwaII-7_CPB	gb|AHGY02039631.1|:42123-46179
NuwaII-7_CPB	gb|AHGY02055830.1|:10437-14050
NuwaII-7_CPB	gb|AHGY02241704.1|:44996-46955
NuwaII-7_CPB	gb|AHGY02212021.1|:1007-5750
NuwaII-7_CPB	gb|AHGY02216230.1|:27789-31982
NuwaII-7_CPB	gb|AHGY02076799.1|:31611-32851
NuwaII-7_CPB	gb|AHGY02006577.1|:39322-43481
NuwaII-7_CPB	gb|AHGY02250990.1|:1066-2076
NuwaII-7_CPB	gb|AHGY02036268.1|:2908-6109
NuwaII-7_CPB	gb|AHGY02038145.1|:58-3174
NuwaII-7_CPB	gb|AHGY02199610.1|:21905-27085
NuwaII-7_CPB	gb|AHGY02222937.1|:1302-3780
NuwaII-7_CPB	gb|AHGY02101029.1|:2163-5408
NuwaII-7_CPB	gb|AHGY02048292.1|:35213-40141
NuwaII-7_CPB	gb|AHGY02083169.1|:4395-8806
NuwaII-7_CPB	gb|AHGY02222699.1|:36603-41174
NuwaII-7_CPB	gb|AHGY02222699.1|:58655-61721
NuwaII-7_CPB	gb|AHGY02001842.1|:1060-5373
NuwaII-7_CPB	gb|AHGY02001842.1|:18060-20506
NuwaII-7_CPB	gb|AHGY02217246.1|:30914-35503
NuwaII-7_CPB	gb|AHGY02217246.1|:506-3743
NuwaII-7_CPB	gb|AHGY02012515.1|:3879-9162
NuwaII-7_CPB	gb|AHGY02054468.1|:25474-29816

NuwaII-7_CPB	gb|AHGY02003252.1|:6426-8798
NuwaII-7_CPB	gb|AHGY02014546.1|:2837-4599
NuwaII-7_CPB	gb|AHGY02057423.1|:5247-7966
NuwaII-7_CPB	gb|AHGY02248066.1|:21325-24910
NuwaII-7_CPB	gb|AHGY02254798.1|:25935-28323
NuwaII-7_CPB	gb|AHGY02221225.1|:4948-6455
NuwaII-7_CPB	gb|AHGY02251164.1|:8341-12604
NuwaII-7_CPB	gb|AHGY02246333.1|:11414-13691
NuwaII-7_CPB	gb|AHGY02211124.1|:4391-7869
NuwaII-7_CPB	gb|AHGY02023366.1|:85993-90987
NuwaII-7_CPB	gb|AHGY02023366.1|:31551-34083
NuwaII-7_CPB	gb|AHGY02020437.1|:14337-19268
NuwaII-7_CPB	gb|AHGY02091678.1|:3248-7967
NuwaII-7_CPB	gb|AHGY02044075.1|:12727-14571
NuwaII-7_CPB	gb|AHGY02031994.1|:6511-10565
NuwaII-7_CPB	gb|AHGY02094541.1|:644-2129
NuwaII-7_CPB	gb|AHGY02094541.1|:2166-2227
NuwaII-7_CPB	gb|AHGY02080068.1|:30651-34777
NuwaII-7_CPB	gb|AHGY02075261.1|:9213-9886
NuwaII-7_CPB	gb|AHGY02075261.1|:67090-68777
NuwaII-7_CPB	gb|AHGY02229276.1|:1-3496
NuwaII-7_CPB	gb|AHGY02017836.1|:76701-80958
NuwaII-7_CPB	gb|AHGY02044210.1|:17379-21575
NuwaII-7_CPB	gb|AHGY02232571.1|:17829-20585
NuwaII-7_CPB	gb|AHGY02138285.1|:60-121
NuwaII-7_CPB	gb|AHGY02138285.1|:158-1276
NuwaII-7_CPB	gb|AHGY02247032.1|:21617-25638
NuwaII-7_CPB	gb|AHGY02208835.1|:6413-7658
NuwaII-7_CPB	gb|AHGY02214980.1|:1975-4959
NuwaII-7_CPB	gb|AHGY02099243.1|:283-4586
NuwaII-7_CPB	gb|AHGY02068397.1|:4999-11361
NuwaII-7_CPB	gb|AHGY02202218.1|:15972-20182
NuwaII-7_CPB	gb|AHGY02202218.1|:574-1330
NuwaII-7_CPB	gb|AHGY02241905.1|:6428-9136
NuwaII-7_CPB	gb|AHGY02068912.1|:263-2412
NuwaII-7_CPB	gb|AHGY02021725.1|:17062-20522
NuwaII-7_CPB	gb|AHGY02218569.1|:80430-84977
NuwaII-7_CPB	gb|AHGY02258230.1|:42494-42913
NuwaII-7_CPB	gb|AHGY02258230.1|:28376-31471
NuwaII-7_CPB	gb|AHGY02234971.1|:237-2544
NuwaII-7_CPB	gb|AHGY02080973.1|:5627-9226
NuwaII-7_CPB	gb|AHGY02217668.1|:3272-7459
NuwaII-7_CPB	gb|AHGY02205880.1|:44960-45654
NuwaII-7_CPB	gb|AHGY02205880.1|:2675-6077

NuwaII-7_CPB	gb|AHGY02001662.1|:9725-11133
NuwaII-7_CPB	gb|AHGY02033310.1|:4539-8531
NuwaII-7_CPB	gb|AHGY02028714.1|:45950-49802
NuwaII-7_CPB	gb|AHGY02089132.1|:2515-5431
NuwaII-7_CPB	gb|AHGY02001579.1|:9061-13019
NuwaII-7_CPB	gb|AHGY02077349.1|:2106-8895
NuwaII-7_CPB	gb|AHGY02240331.1|:972-1962
NuwaII-7_CPB	gb|AHGY02096510.1|:27793-30460
NuwaII-7_CPB	gb|AHGY02253965.1|:5401-10651
NuwaII-7_CPB	gb|AHGY02069879.1|:17085-19884
NuwaII-7_CPB	gb|AHGY02229828.1|:30317-36924
NuwaII-7_CPB	gb|AHGY02024052.1|:13804-15858
NuwaII-7_CPB	gb|AHGY02062569.1|:35514-38832
NuwaII-7_CPB	gb|AHGY02057885.1|:4661-8685
NuwaII-7_CPB	gb|AHGY02057885.1|:12745-16288
NuwaII-7_CPB	gb|AHGY02038040.1|:10087-12806
NuwaII-7_CPB	gb|AHGY02161063.1|:43-2551
NuwaII-7_CPB	gb|AHGY02070696.1|:220-4335
NuwaII-7_CPB	gb|AHGY02050197.1|:136-4757
NuwaII-7_CPB	gb|AHGY02016280.1|:53-2244
NuwaII-7_CPB	gb|AHGY02013704.1|:12272-15496
NuwaII-7_CPB	gb|AHGY02050780.1|:579-2381
NuwaII-7_CPB	gb|AHGY02234970.1|:26103-29426
NuwaII-7_CPB	gb|AHGY02052199.1|:160-2270
NuwaII-7_CPB	gb|AHGY02223293.1|:7303-10768
NuwaII-7_CPB	gb|AHGY02202319.1|:24398-28403
NuwaII-7_CPB	gb|AHGY02036953.1|:1302-4754
NuwaII-7_CPB	gb|AHGY02016102.1|:23024-27153
NuwaII-7_CPB	gb|AHGY02065889.1|:5367-10211
NuwaII-7_CPB	gb|AHGY02004802.1|:3390-4255
NuwaII-7_CPB	gb|AHGY02014595.1|:12905-14015
NuwaII-7_CPB	gb|AHGY02061755.1|:12658-15882
NuwaII-7_CPB	gb|AHGY02054739.1|:5350-8081
NuwaII-7_CPB	gb|AHGY02082990.1|:411-6149
NuwaII-7_CPB	gb|AHGY02045680.1|:11638-14076
NuwaII-7_CPB	gb|AHGY02045680.1|:310-3324
NuwaII-7_CPB	gb|AHGY02254407.1|:2791-9008
NuwaII-7_CPB	gb|AHGY02068203.1|:27037-31052
NuwaII-7_CPB	gb|AHGY02252281.1|:3517-8015
NuwaII-7_CPB	gb|AHGY02215495.1|:6785-8588
NuwaII-7_CPB	gb|AHGY02072548.1|:15242-16807
NuwaII-7_CPB	gb|AHGY02072548.1|:25543-25617
NuwaII-7_CPB	gb|AHGY02072548.1|:10865-13325
NuwaII-7_CPB	gb|AHGY02252666.1|:303-1165

NuwaII-7_CPB	gb|AHGY02228644.1|:8146-12826
NuwaII-7_CPB	gb|AHGY02218886.1|:189-2588
NuwaII-7_CPB	gb|AHGY02006359.1|:58586-62915
NuwaII-7_CPB	gb|AHGY02221062.1|:37831-38973
NuwaII-7_CPB	gb|AHGY02261285.1|:19725-22475
NuwaII-7_CPB	gb|AHGY02035034.1|:712-3516
NuwaII-7_CPB	gb|AHGY02016484.1|:16786-20931
NuwaII-7_CPB	gb|AHGY02222227.1|:24253-27426
NuwaII-7_CPB	gb|AHGY02096477.1|:297-3538
NuwaII-7_CPB	gb|AHGY02248451.1|:8079-9309
NuwaII-7_CPB	gb|AHGY02241241.1|:6509-11215
NuwaII-7_CPB	gb|AHGY02216813.1|:2609-5982
NuwaII-7_CPB	gb|AHGY02060980.1|:14445-17766
NuwaII-7_CPB	gb|AHGY02060980.1|:54527-58180
NuwaII-7_CPB	gb|AHGY02043207.1|:1800-3077
NuwaII-7_CPB	gb|AHGY02001338.1|:176-1325
NuwaII-7_CPB	gb|AHGY02196394.1|:12554-16960
NuwaII-7_CPB	gb|AHGY02240203.1|:2277-3513
NuwaII-7_CPB	gb|AHGY02200076.1|:14333-17146
NuwaII-7_CPB	gb|AHGY02200076.1|:10795-11579
NuwaII-7_CPB	gb|AHGY02051511.1|:1550-4969
NuwaII-7_CPB	gb|AHGY02199885.1|:16402-20601
NuwaII-7_CPB	gb|AHGY02249976.1|:9483-10734
NuwaII-7_CPB	gb|AHGY02032072.1|:36-2596
NuwaII-7_CPB	gb|AHGY02032072.1|:7847-11050
NuwaII-7_CPB	gb|AHGY02258389.1|:4349-7653
NuwaII-7_CPB	gb|AHGY02247499.1|:45752-48922
NuwaII-7_CPB	gb|AHGY02247499.1|:69835-72379
NuwaII-7_CPB	gb|AHGY02230421.1|:29473-29651
NuwaII-7_CPB	gb|AHGY02230421.1|:32591-36719
NuwaII-7_CPB	gb|AHGY02008273.1|:3897-8547
NuwaII-7_CPB	gb|AHGY02253126.1|:45276-47201
NuwaII-7_CPB	gb|AHGY02221848.1|:2258-6131
NuwaII-7_CPB	gb|AHGY02221848.1|:39-613
NuwaII-7_CPB	gb|AHGY02060594.1|:15263-18419
NuwaII-7_CPB	gb|AHGY02010606.1|:1055-3705
NuwaII-7_CPB	gb|AHGY02060113.1|:8589-12749
NuwaII-7_CPB	gb|AHGY02241010.1|:409-2559
NuwaII-7_CPB	gb|AHGY02066358.1|:28146-30337
NuwaII-7_CPB	gb|AHGY02036697.1|:10448-13572
NuwaII-7_CPB	gb|AHGY02073577.1|:5961-9443
NuwaII-7_CPB	gb|AHGY02014424.1|:25896-29904
NuwaII-7_CPB	gb|AHGY02199570.1|:50716-52700
NuwaII-7_CPB	gb|AHGY02047921.1|:421-3342

NuwaII-7_CPB	gb|AHGY02200281.1|:12134-15731
NuwaII-7_CPB	gb|AHGY02200281.1|:336-745
NuwaII-7_CPB	gb|AHGY02060273.1|:15358-16516
NuwaII-7_CPB	gb|AHGY02060273.1|:11759-15320
NuwaII-7_CPB	gb|AHGY02060273.1|:271-1162
NuwaII-7_CPB	gb|AHGY02251010.1|:15562-24139
NuwaII-7_CPB	gb|AHGY02042393.1|:26042-29319
NuwaII-7_CPB	gb|AHGY02238474.1|:64-3164
NuwaII-7_CPB	gb|AHGY02245600.1|:6719-11295
NuwaII-7_CPB	gb|AHGY02238473.1|:5166-6369
NuwaII-7_CPB	gb|AHGY02234178.1|:51532-55355
NuwaII-7_CPB	gb|AHGY02234178.1|:34009-34170
NuwaII-7_CPB	gb|AHGY02201665.1|:76-1596
NuwaII-7_CPB	gb|AHGY02088511.1|:5725-9172
NuwaII-7_CPB	gb|AHGY02254823.1|:50493-51385
NuwaII-7_CPB	gb|AHGY02254823.1|:3228-5564
NuwaII-7_CPB	gb|AHGY02053535.1|:6081-7031
NuwaII-7_CPB	gb|AHGY02205898.1|:10464-13442
NuwaII-7_CPB	gb|AHGY02049242.1|:27909-28661
NuwaII-7_CPB	gb|AHGY02049242.1|:10495-15553
NuwaII-7_CPB	gb|AHGY02032233.1|:5473-9125
NuwaII-7_CPB	gb|AHGY02006947.1|:7885-10850
NuwaII-7_CPB	gb|AHGY02200354.1|:6176-10524
NuwaII-7_CPB	gb|AHGY02196203.1|:835-1902
NuwaII-7_CPB	gb|AHGY02256827.1|:36355-39584
NuwaII-7_CPB	gb|AHGY02106410.1|:13195-16587
NuwaII-7_CPB	gb|AHGY02041305.1|:21296-24762
NuwaII-7_CPB	gb|AHGY02253655.1|:12870-16180
NuwaII-7_CPB	gb|AHGY02223931.1|:211-3564
NuwaII-7_CPB	gb|AHGY02214848.1|:1118-2361
NuwaII-7_CPB	gb|AHGY02033453.1|:664-2220
NuwaII-7_CPB	gb|AHGY02245408.1|:90-3171
NuwaII-7_CPB	gb|AHGY02222243.1|:4100-6863
NuwaII-7_CPB	gb|AHGY02209788.1|:11271-13947
NuwaII-7_CPB	gb|AHGY02203252.1|:2698-6616
NuwaII-7_CPB	gb|AHGY02059044.1|:2752-7139
NuwaII-7_CPB	gb|AHGY02059044.1|:3610-3664
NuwaII-7_CPB	gb|AHGY02010528.1|:21429-25002
NuwaII-7_CPB	gb|AHGY02221978.1|:12147-23270
NuwaII-7_CPB	gb|AHGY02221978.1|:22079-22253
NuwaII-7_CPB	gb|AHGY02099169.1|:31667-33920
NuwaII-7_CPB	gb|AHGY02099169.1|:79715-80498
NuwaII-7_CPB	gb|AHGY02094446.1|:841-1734
NuwaII-7_CPB	gb|AHGY02005721.1|:819-2681

NuwaII-7_CPB	gb|AHGY02259348.1|:661-1553
NuwaII-7_CPB	gb|AHGY02256279.1|:14647-16246
NuwaII-7_CPB	gb|AHGY02137204.1|:348-1240
NuwaII-7_CPB	gb|AHGY02047970.1|:11975-12867
NuwaII-7_CPB	gb|AHGY02047970.1|:1987-6251
NuwaII-7_CPB	gb|AHGY02030959.1|:420-1312
NuwaII-7_CPB	gb|AHGY02008511.1|:19904-22481
NuwaII-7_CPB	gb|AHGY02074656.1|:4910-8880
NuwaII-7_CPB	gb|AHGY02000047.1|:10055-13391
NuwaII-7_CPB	gb|AHGY02000904.1|:8945-13053
NuwaII-7_CPB	gb|AHGY02205706.1|:8185-11796
NuwaII-7_CPB	gb|AHGY02059713.1|:43243-46514
NuwaII-7_CPB	gb|AHGY02101627.1|:944-6814
NuwaII-7_CPB	gb|AHGY02064777.1|:2020-5852
NuwaII-7_CPB	gb|AHGY02064777.1|:588-849
NuwaII-7_CPB	gb|AHGY02052984.1|:201-1092
NuwaII-7_CPB	gb|AHGY02042662.1|:14648-17221
NuwaII-7_CPB	gb|AHGY02229605.1|:2501-6346
NuwaII-7_CPB	gb|AHGY02234822.1|:40927-41824
NuwaII-7_CPB	gb|AHGY02196115.1|:82278-85041
NuwaII-7_CPB	gb|AHGY02196115.1|:55013-56264
NuwaII-7_CPB	gb|AHGY02004180.1|:42459-44703
NuwaII-7_CPB	gb|AHGY02135909.1|:477-1371
NuwaII-7_CPB	gb|AHGY02084951.1|:567-3803
NuwaII-7_CPB	gb|AHGY02052682.1|:274-1171
NuwaII-7_CPB	gb|AHGY02000795.1|:7941-11141
NuwaII-8_CPB	gb|AHGY02056999.1|:2013-5418
NuwaII-8_CPB	gb|AHGY02207655.1|:2048-4272
NuwaII-8_CPB	gb|AHGY02227567.1|:3843-7231
NuwaII-8_CPB	gb|AHGY02049626.1|:7388-10751
NuwaII-8_CPB	gb|AHGY02053432.1|:44698-48205
NuwaII-8_CPB	gb|AHGY02222198.1|:15555-18315
NuwaII-8_CPB	gb|AHGY02047977.1|:4530-8009
NuwaII-8_CPB	gb|AHGY02047977.1|:48-2765
NuwaII-8_CPB	gb|AHGY02208824.1|:5392-8881
NuwaII-8_CPB	gb|AHGY02196083.1|:6824-7016
NuwaII-8_CPB	gb|AHGY02196083.1|:1-3339
NuwaII-8_CPB	gb|AHGY02223438.1|:23364-26761
NuwaII-8_CPB	gb|AHGY02044525.1|:1-2108
NuwaII-8_CPB	gb|AHGY02201823.1|:322-3827
NuwaII-8_CPB	gb|AHGY02052592.1|:17091-20565
NuwaII-8_CPB	gb|AHGY02197879.1|:1-2838
NuwaII-8_CPB	gb|AHGY02249507.1|:92533-93492
NuwaII-8_CPB	gb|AHGY02249507.1|:59371-62794

NuwaII-8_CPB	gb|AHGY02249507.1|:49-111
NuwaII-8_CPB	gb|AHGY02000634.1|:4367-7840
NuwaII-8_CPB	gb|AHGY02231058.1|:6772-9416
NuwaII-8_CPB	gb|AHGY02234716.1|:17601-20254
NuwaII-8_CPB	gb|AHGY02029420.1|:19698-20762
NuwaII-8_CPB	gb|AHGY02029420.1|:11342-14827
NuwaII-8_CPB	gb|AHGY02084653.1|:47501-50160
NuwaII-8_CPB	gb|AHGY02217123.1|:1-2311
NuwaII-8_CPB	gb|AHGY02092667.1|:7-2753
NuwaII-8_CPB	gb|AHGY02078217.1|:1-2372
NuwaII-8_CPB	gb|AHGY02225623.1|:9804-13277
NuwaII-8_CPB	gb|AHGY02225623.1|:53437-55592
NuwaII-8_CPB	gb|AHGY02098490.1|:38280-41414
NuwaII-8_CPB	gb|AHGY02254234.1|:34867-37743
NuwaII-8_CPB	gb|AHGY02000371.1|:8218-10264
NuwaII-8_CPB	gb|AHGY02222401.1|:9-2947
NuwaII-8_CPB	gb|AHGY02252963.1|:3112-5542
NuwaII-8_CPB	gb|AHGY02257471.1|:18092-21535
NuwaII-8_CPB	gb|AHGY02102600.1|:4453-7826
NuwaII-8_CPB	gb|AHGY02059078.1|:3421-6137
NuwaII-8_CPB	gb|AHGY02061857.1|:11049-14536
NuwaII-8_CPB	gb|AHGY02061857.1|:27753-29117
NuwaII-8_CPB	gb|AHGY02036610.1|:27183-29902
NuwaII-8_CPB	gb|AHGY02062631.1|:5874-9343
NuwaII-8_CPB	gb|AHGY02032783.1|:1352-4025
NuwaII-8_CPB	gb|AHGY02225872.1|:10891-13550
NuwaII-8_CPB	gb|AHGY02113302.1|:10729-14103
NuwaII-8_CPB	gb|AHGY02096161.1|:4183-7666
NuwaII-8_CPB	gb|AHGY02027290.1|:5857-9296
NuwaII-8_CPB	gb|AHGY02056031.1|:2652-6125
NuwaII-8_CPB	gb|AHGY02221948.1|:22592-24500
NuwaII-8_CPB	gb|AHGY02252853.1|:1-2008
NuwaII-8_CPB	gb|AHGY02225752.1|:5273-8008
NuwaII-8_CPB	gb|AHGY02227475.1|:34933-38482
NuwaII-8_CPB	gb|AHGY02167552.1|:1-2316
NuwaII-8_CPB	gb|AHGY02096159.1|:2168-4477
NuwaII-8_CPB	gb|AHGY02082554.1|:4262-7615
NuwaII-8_CPB	gb|AHGY02248286.1|:5319-8018
NuwaII-8_CPB	gb|AHGY02097565.1|:17508-20605
NuwaII-8_CPB	gb|AHGY02119128.1|:1-2309
NuwaII-8_CPB	gb|AHGY02055260.1|:1-2223
NuwaII-8_CPB	gb|AHGY02201719.1|:14600-17960
NuwaII-8_CPB	gb|AHGY02032008.1|:20702-24025
NuwaII-8_CPB	gb|AHGY02034535.1|:8613-11230

NuwaII-8_CPB	gb|AHGY02230430.1|:20-3033
NuwaII-8_CPB	gb|AHGY02239373.1|:21923-24216
NuwaII-8_CPB	gb|AHGY02239373.1|:20116-21580
NuwaII-8_CPB	gb|AHGY02100787.1|:11979-15368
NuwaII-8_CPB	gb|AHGY02225945.1|:7205-9312
NuwaII-8_CPB	gb|AHGY02248746.1|:9097-12588
NuwaII-8_CPB	gb|AHGY02245565.1|:17-2735
NuwaII-8_CPB	gb|AHGY02078161.1|:3372-6657
NuwaII-8_CPB	gb|AHGY02065163.1|:1-2685
NuwaII-8_CPB	gb|AHGY02082289.1|:7751-11196
NuwaII-8_CPB	gb|AHGY02065760.1|:27444-29257
NuwaII-8_CPB	gb|AHGY02246178.1|:116647-119368
NuwaII-8_CPB	gb|AHGY02050511.1|:9419-12078
NuwaII-8_CPB	gb|AHGY02110623.1|:5-2185
NuwaII-8_CPB	gb|AHGY02100196.1|:2699-6075
NuwaII-8_CPB	gb|AHGY02084662.1|:8688-12108
NuwaII-8_CPB	gb|AHGY02052358.1|:39-2759
NuwaII-8_CPB	gb|AHGY02031200.1|:17073-20516
NuwaII-8_CPB	gb|AHGY02216675.1|:4000-7367
NuwaII-8_CPB	gb|AHGY02038161.1|:33236-35323
NuwaII-8_CPB	gb|AHGY02038161.1|:24-2493
NuwaII-8_CPB	gb|AHGY02057189.1|:6-2118
NuwaII-8_CPB	gb|AHGY02218990.1|:1-2926
NuwaII-8_CPB	gb|AHGY02222710.1|:7969-11332
NuwaII-8_CPB	gb|AHGY02055955.1|:2420-4714
NuwaII-8_CPB	gb|AHGY02105093.1|:66-1951
NuwaII-8_CPB	gb|AHGY02206923.1|:8063-10069
NuwaII-8_CPB	gb|AHGY02084819.1|:4610-7246
NuwaII-8_CPB	gb|AHGY02248835.1|:7870-10588
NuwaII-8_CPB	gb|AHGY02254823.1|:49893-53301
NuwaII-8_CPB	gb|AHGY02254823.1|:4224-5703
NuwaII-8_CPB	gb|AHGY02075465.1|:2970-5977
NuwaII-8_CPB	gb|AHGY02013138.1|:18023-21361
NuwaII-8_CPB	gb|AHGY02027100.1|:1-1760
NuwaII-8_CPB	gb|AHGY02214479.1|:1-3199
NuwaII-8_CPB	gb|AHGY02214479.1|:14875-14942
NuwaII-8_CPB	gb|AHGY02076837.1|:2064-3879
NuwaII-8_CPB	gb|AHGY02093561.1|:9153-10881
NuwaII-8_CPB	gb|AHGY02216291.1|:24088-27539
NuwaII-8_CPB	gb|AHGY02223237.1|:1-1861
NuwaII-8_CPB	gb|AHGY02206144.1|:46113-49497
NuwaII-8_CPB	gb|AHGY02206144.1|:3665-3957
NuwaII-8_CPB	gb|AHGY02245200.1|:7836-11216
NuwaII-8_CPB	gb|AHGY02048031.1|:2888-6278

NuwaII-8_CPB	gb|AHGY02222055.1|:23985-27303
NuwaII-8_CPB	gb|AHGY02261568.1|:53959-57834
NuwaII-8_CPB	gb|AHGY02247107.1|:3023-4720
NuwaII-8_CPB	gb|AHGY02104717.1|:3640-5363
NuwaII-8_CPB	gb|AHGY02088762.1|:1-2494
NuwaII-8_CPB	gb|AHGY02093977.1|:1-2004
NuwaII-8_CPB	gb|AHGY02011987.1|:2619-4552
NuwaII-8_CPB	gb|AHGY02063484.1|:17903-21008
NuwaII-8_CPB	gb|AHGY02223469.1|:2786-4695
NuwaII-8_CPB	gb|AHGY02034655.1|:16094-19123
NuwaII-8_CPB	gb|AHGY02206411.1|:35280-38742
NuwaII-8_CPB	gb|AHGY02197313.1|:1444-3794
NuwaII-8_CPB	gb|AHGY02070543.1|:31832-34160
NuwaII-8_CPB	gb|AHGY02078411.1|:2458-5907
NuwaII-8_CPB	gb|AHGY02060273.1|:15624-16623
NuwaII-8_CPB	gb|AHGY02060273.1|:2-1756
NuwaII-8_CPB	gb|AHGY02197365.1|:1-2846
NuwaII-8_CPB	gb|AHGY02040509.1|:86-3108
NuwaII-8_CPB	gb|AHGY02222592.1|:9409-9741
NuwaII-8_CPB	gb|AHGY02222592.1|:42264-42376
NuwaII-8_CPB	gb|AHGY02222592.1|:45731-48019
NuwaII-8_CPB	gb|AHGY02254732.1|:11672-14399
NuwaII-8_CPB	gb|AHGY02054488.1|:8277-10147
NuwaII-8_CPB	gb|AHGY02207093.1|:1-1682
NuwaII-8_CPB	gb|AHGY02063001.1|:2724-6124
NuwaII-8_CPB	gb|AHGY02203716.1|:9-7347
NuwaII-8_CPB	gb|AHGY02041900.1|:3085-6466
NuwaII-8_CPB	gb|AHGY02214474.1|:8725-10741
NuwaII-8_CPB	gb|AHGY02061654.1|:5462-8913
NuwaII-8_CPB	gb|AHGY02200621.1|:47331-50342
NuwaII-8_CPB	gb|AHGY02200621.1|:25840-33786
NuwaII-8_CPB	gb|AHGY02252418.1|:1047-4457
NuwaII-8_CPB	gb|AHGY02113363.1|:6569-8282
NuwaII-8_CPB	gb|AHGY02091554.1|:8411-10843
NuwaII-8_CPB	gb|AHGY02220991.1|:11732-11800
NuwaII-8_CPB	gb|AHGY02220991.1|:6217-9649
NuwaII-8_CPB	gb|AHGY02243489.1|:1-2532
NuwaII-8_CPB	gb|AHGY02240340.1|:2-2758
NuwaII-8_CPB	gb|AHGY02219338.1|:6457-10046
NuwaII-8_CPB	gb|AHGY02219338.1|:45874-47499
NuwaII-8_CPB	gb|AHGY02228717.1|:9909-11805
NuwaII-8_CPB	gb|AHGY02199932.1|:52264-55620
NuwaII-8_CPB	gb|AHGY02195294.1|:10-1967
NuwaII-8_CPB	gb|AHGY02029330.1|:2-2410

NuwaII-8_CPB	gb|AHGY02221144.1|:4048-5838
NuwaII-8_CPB	gb|AHGY02214148.1|:8562-11566
NuwaII-8_CPB	gb|AHGY02224864.1|:3039-6492
NuwaII-8_CPB	gb|AHGY02050904.1|:9860-13343
NuwaII-8_CPB	gb|AHGY02100783.1|:395-3548
NuwaII-8_CPB	gb|AHGY02050305.1|:8682-10347
NuwaII-8_CPB	gb|AHGY02230485.1|:1-2832
NuwaII-8_CPB	gb|AHGY02113163.1|:1-2743
NuwaII-8_CPB	gb|AHGY02241538.1|:12112-15044
NuwaII-8_CPB	gb|AHGY02046857.1|:16043-18082
NuwaII-8_CPB	gb|AHGY02046857.1|:8915-9230
NuwaII-8_CPB	gb|AHGY02257451.1|:24257-28362
NuwaII-8_CPB	gb|AHGY02257451.1|:39437-42524
NuwaII-8_CPB	gb|AHGY02016883.1|:14554-14776
NuwaII-8_CPB	gb|AHGY02016883.1|:1-3316
NuwaII-8_CPB	gb|AHGY02057720.1|:3424-6991
NuwaII-8_CPB	gb|AHGY02131122.1|:176-1854
NuwaII-8_CPB	gb|AHGY02033227.1|:5058-6964
NuwaII-8_CPB	gb|AHGY02068978.1|:7475-10846
NuwaII-8_CPB	gb|AHGY02039525.1|:8380-11840
NuwaII-8_CPB	gb|AHGY02261907.1|:1-3058
NuwaII-8_CPB	gb|AHGY02049318.1|:894-2859
NuwaII-8_CPB	gb|AHGY02260055.1|:59747-63279
NuwaII-8_CPB	gb|AHGY02240152.1|:55215-57379
NuwaII-8_CPB	gb|AHGY02240152.1|:1-1688
NuwaII-8_CPB	gb|AHGY02260980.1|:2-3433
NuwaII-8_CPB	gb|AHGY02257336.1|:3841-7244
NuwaII-8_CPB	gb|AHGY02085001.1|:29548-33006
NuwaII-8_CPB	gb|AHGY02035302.1|:5027-7954
NuwaII-8_CPB	gb|AHGY02015990.1|:287-2769
NuwaII-8_CPB	gb|AHGY02058661.1|:25-2846
NuwaII-8_CPB	gb|AHGY02095516.1|:5704-7996
NuwaII-8_CPB	gb|AHGY02016152.1|:10048-13552
NuwaII-8_CPB	gb|AHGY02016152.1|:41641-42019
NuwaII-8_CPB	gb|AHGY02061456.1|:9448-12793
NuwaII-8_CPB	gb|AHGY02023702.1|:1-1845
NuwaII-8_CPB	gb|AHGY02245585.1|:11683-14160
NuwaII-8_CPB	gb|AHGY02195470.1|:1-1897
NuwaII-8_CPB	gb|AHGY02018612.1|:1-1835
NuwaII-8_CPB	gb|AHGY02018344.1|:23945-25070
NuwaII-8_CPB	gb|AHGY02018344.1|:8453-11845
NuwaII-8_CPB	gb|AHGY02245327.1|:1-2499
NuwaII-8_CPB	gb|AHGY02221764.1|:5402-7315
NuwaII-8_CPB	gb|AHGY02005108.1|:36-2003

NuwaII-8_CPB	gb|AHGY02001311.1|:16041-17051
NuwaII-8_CPB	gb|AHGY02001311.1|:69441-72187
NuwaII-8_CPB	gb|AHGY02019508.1|:94-2056
NuwaII-8_CPB	gb|AHGY02220311.1|:5-1563
NuwaII-8_CPB	gb|AHGY02095315.1|:262-2351
NuwaII-8_CPB	gb|AHGY02043770.1|:21408-24858
NuwaII-8_CPB	gb|AHGY02240353.1|:25196-27751
NuwaII-8_CPB	gb|AHGY02238736.1|:13289-16867
NuwaII-8_CPB	gb|AHGY02213444.1|:30-2396
NuwaII-8_CPB	gb|AHGY02248773.1|:62-1869
NuwaII-8_CPB	gb|AHGY02227488.1|:608-3993
NuwaII-8_CPB	gb|AHGY02078285.1|:553-4074
NuwaII-8_CPB	gb|AHGY02248671.1|:1-2541
NuwaII-8_CPB	gb|AHGY02008519.1|:126-2845
NuwaII-8_CPB	gb|AHGY02001913.1|:12531-15968
NuwaII-8_CPB	gb|AHGY02246497.1|:14327-16935
NuwaII-8_CPB	gb|AHGY02060981.1|:24356-27873
NuwaII-8_CPB	gb|AHGY02078052.1|:11625-14987
NuwaII-8_CPB	gb|AHGY02051205.1|:1-1575
NuwaII-8_CPB	gb|AHGY02088131.1|:1455-2891
NuwaII-8_CPB	gb|AHGY02088131.1|:13450-16922
NuwaII-8_CPB	gb|AHGY02022687.1|:15619-18921
NuwaII-8_CPB	gb|AHGY02022687.1|:178-434
NuwaII-8_CPB	gb|AHGY02081377.1|:1-3276
NuwaII-8_CPB	gb|AHGY02068362.1|:24543-27666
NuwaII-8_CPB	gb|AHGY02057344.1|:16399-19374
NuwaII-8_CPB	gb|AHGY02083276.1|:3753-7169
NuwaII-8_CPB	gb|AHGY02245566.1|:45510-47847
NuwaII-8_CPB	gb|AHGY02043727.1|:14384-17842
NuwaII-8_CPB	gb|AHGY02045820.1|:31-1995
NuwaII-8_CPB	gb|AHGY02251036.1|:17843-20602
NuwaII-8_CPB	gb|AHGY02251256.1|:17586-21058
NuwaII-8_CPB	gb|AHGY02030725.1|:35622-39023
NuwaII-8_CPB	gb|AHGY02204792.1|:3479-6957
NuwaII-8_CPB	gb|AHGY02133289.1|:1-1619
NuwaII-8_CPB	gb|AHGY02210673.1|:4923-6755
NuwaII-8_CPB	gb|AHGY02089064.1|:25335-27470
NuwaII-8_CPB	gb|AHGY02261774.1|:2794-4282
NuwaII-8_CPB	gb|AHGY02198395.1|:13709-17185
NuwaII-8_CPB	gb|AHGY02231897.1|:6570-9335
NuwaII-8_CPB	gb|AHGY02034939.1|:11-2852
NuwaII-8_CPB	gb|AHGY02059650.1|:1535-4963
NuwaII-8_CPB	gb|AHGY02220960.1|:11598-14545
NuwaII-8_CPB	gb|AHGY02220960.1|:27-1698

NuwaII-8_CPB	gb|AHGY02051899.1|:2508-4637
NuwaII-8_CPB	gb|AHGY02028632.1|:2-2435
NuwaII-8_CPB	gb|AHGY02011704.1|:7427-10011
NuwaII-8_CPB	gb|AHGY02066120.1|:14081-16221
NuwaII-8_CPB	gb|AHGY02026944.1|:6299-8130
NuwaII-8_CPB	gb|AHGY02098892.1|:1673-5110
NuwaII-8_CPB	gb|AHGY02200256.1|:10539-13366
NuwaII-8_CPB	gb|AHGY02032083.1|:3550-6759
NuwaII-8_CPB	gb|AHGY02077926.1|:1803-4523
NuwaII-8_CPB	gb|AHGY02247599.1|:322-638
NuwaII-8_CPB	gb|AHGY02247599.1|:4011-7294
NuwaII-8_CPB	gb|AHGY02224275.1|:6940-10409
NuwaII-8_CPB	gb|AHGY02057758.1|:8718-11863
NuwaII-8_CPB	gb|AHGY02049688.1|:1-1545
NuwaII-8_CPB	gb|AHGY02238205.1|:11297-14632
NuwaII-8_CPB	gb|AHGY02259587.1|:5-3024
NuwaII-8_CPB	gb|AHGY02060561.1|:13199-16612
NuwaII-8_CPB	gb|AHGY02047796.1|:1-2944
NuwaII-8_CPB	gb|AHGY02093582.1|:238-2266
NuwaII-8_CPB	gb|AHGY02043597.1|:8920-11436
NuwaII-8_CPB	gb|AHGY02224424.1|:1792-3749
NuwaII-8_CPB	gb|AHGY02258243.1|:13632-17047
NuwaII-8_CPB	gb|AHGY02031151.1|:9157-10592
NuwaII-8_CPB	gb|AHGY02254801.1|:5753-9197
NuwaII-8_CPB	gb|AHGY02090559.1|:27806-30847
NuwaII-8_CPB	gb|AHGY02007244.1|:3967-7276
NuwaII-8_CPB	gb|AHGY02016316.1|:6902-10105
NuwaII-8_CPB	gb|AHGY02025529.1|:1540-4759
NuwaII-8_CPB	gb|AHGY02216516.1|:1-2281
NuwaII-8_CPB	gb|AHGY02225004.1|:1-2561
NuwaII-8_CPB	gb|AHGY02134952.1|:1-1477
NuwaII-8_CPB	gb|AHGY02013623.1|:82198-83824
NuwaII-8_CPB	gb|AHGY02218482.1|:12911-16381
NuwaII-8_CPB	gb|AHGY02083372.1|:30400-33790
NuwaII-8_CPB	gb|AHGY02198700.1|:5341-8608
NuwaII-8_CPB	gb|AHGY02098689.1|:2551-6595
NuwaII-8_CPB	gb|AHGY02069944.1|:1881-5325
NuwaII-8_CPB	gb|AHGY02236501.1|:7183-10530
NuwaII-8_CPB	gb|AHGY02211006.1|:72-3573
NuwaII-8_CPB	gb|AHGY02011425.1|:3794-7245
NuwaII-8_CPB	gb|AHGY02031957.1|:2-1989
NuwaII-9_CPB	gb|AHGY02011165.1|:11183-14619
NuwaII-9_CPB	gb|AHGY02221017.1|:3756-8172
NuwaII-9_CPB	gb|AHGY02028326.1|:22717-27643

NuwaII-9_CPB	gb|AHGY02086867.1|:19915-23888
NuwaII-9_CPB	gb|AHGY02084153.1|:67798-72044
NuwaII-9_CPB	gb|AHGY02257746.1|:4-2622
NuwaII-9_CPB	gb|AHGY02237558.1|:6707-10162
NuwaII-9_CPB	gb|AHGY02031362.1|:7769-11697
NuwaII-9_CPB	gb|AHGY02101683.1|:990-4887
NuwaII-9_CPB	gb|AHGY02214410.1|:1-2563
NuwaII-9_CPB	gb|AHGY02196245.1|:101-3041
NuwaII-9_CPB	gb|AHGY02014792.1|:7135-11611
NuwaII-9_CPB	gb|AHGY02065298.1|:4859-8255
NuwaII-9_CPB	gb|AHGY02052531.1|:3433-8414
NuwaII-9_CPB	gb|AHGY02260615.1|:29073-29150
NuwaII-9_CPB	gb|AHGY02260615.1|:20556-24591
NuwaII-9_CPB	gb|AHGY02093691.1|:7969-12303
NuwaII-9_CPB	gb|AHGY02084322.1|:14094-17980
NuwaII-9_CPB	gb|AHGY02039133.1|:7449-11082
NuwaII-9_CPB	gb|AHGY02223285.1|:13358-18012
NuwaII-9_CPB	gb|AHGY02055909.1|:7903-12113
NuwaII-9_CPB	gb|AHGY02236577.1|:32093-35474
NuwaII-9_CPB	gb|AHGY02213252.1|:16626-20542
NuwaII-9_CPB	gb|AHGY02064190.1|:9119-11823
NuwaII-9_CPB	gb|AHGY02043075.1|:34981-39304
NuwaII-9_CPB	gb|AHGY02044953.1|:1981-5916
NuwaII-9_CPB	gb|AHGY02239448.1|:40930-45572
NuwaII-9_CPB	gb|AHGY02218703.1|:3662-7683
NuwaII-9_CPB	gb|AHGY02052881.1|:34191-37686
NuwaII-9_CPB	gb|AHGY02052881.1|:8981-9791
NuwaII-9_CPB	gb|AHGY02234830.1|:30753-32920
NuwaII-9_CPB	gb|AHGY02036066.1|:24909-28960
NuwaII-9_CPB	gb|AHGY02036066.1|:39962-40011
NuwaII-9_CPB	gb|AHGY02000719.1|:29934-34096
NuwaII-9_CPB	gb|AHGY02110679.1|:7171-10571
NuwaII-9_CPB	gb|AHGY02196127.1|:3123-7197
NuwaII-9_CPB	gb|AHGY02206833.1|:5970-10216
NuwaII-9_CPB	gb|AHGY02068485.1|:17363-20779
NuwaII-9_CPB	gb|AHGY02062819.1|:21728-25991
NuwaII-9_CPB	gb|AHGY02023196.1|:22422-26567
NuwaII-9_CPB	gb|AHGY02257358.1|:4131-8427
NuwaII-9_CPB	gb|AHGY02046875.1|:268-5498
NuwaII-9_CPB	gb|AHGY02008870.1|:6640-10993
NuwaII-9_CPB	gb|AHGY02090059.1|:15708-18068
NuwaII-9_CPB	gb|AHGY02047322.1|:27874-31115
NuwaII-9_CPB	gb|AHGY02009560.1|:1249-4828
NuwaII-9_CPB	gb|AHGY02218895.1|:25816-30378

NuwaII-9_CPB	gb|AHGY02200659.1|:29579-33750
NuwaII-9_CPB	gb|AHGY02210088.1|:1749-5751
NuwaII-9_CPB	gb|AHGY02100066.1|:1106-4959
NuwaII-9_CPB	gb|AHGY02196252.1|:48926-48991
NuwaII-9_CPB	gb|AHGY02196252.1|:10214-14033
NuwaII-9_CPB	gb|AHGY02245731.1|:2671-6490
NuwaII-9_CPB	gb|AHGY02011125.1|:1-271
NuwaII-9_CPB	gb|AHGY02011125.1|:13734-17932
NuwaII-9_CPB	gb|AHGY02232627.1|:10579-12492
NuwaII-9_CPB	gb|AHGY02044628.1|:1466-6038
NuwaII-9_CPB	gb|AHGY02198146.1|:20-2311
NuwaII-9_CPB	gb|AHGY02062392.1|:6140-10161
NuwaII-9_CPB	gb|AHGY02105442.1|:411-5638
NuwaII-9_CPB	gb|AHGY02198795.1|:583-3073
NuwaII-9_CPB	gb|AHGY02089959.1|:32388-36346
NuwaII-9_CPB	gb|AHGY02247627.1|:15877-19963
NuwaII-9_CPB	gb|AHGY02087659.1|:1519-5563
NuwaII-9_CPB	gb|AHGY02235720.1|:129-2848
NuwaII-9_CPB	gb|AHGY02224185.1|:17348-18437
NuwaII-9_CPB	gb|AHGY02224185.1|:16710-22337
NuwaII-9_CPB	gb|AHGY02066658.1|:1646-6353
NuwaII-9_CPB	gb|AHGY02066658.1|:12719-16675
NuwaII-9_CPB	gb|AHGY02241355.1|:1515-5663
NuwaII-9_CPB	gb|AHGY02078873.1|:14071-17816
NuwaII-9_CPB	gb|AHGY02238721.1|:14860-18973
NuwaII-9_CPB	gb|AHGY02249939.1|:27386-31926
NuwaII-9_CPB	gb|AHGY02210767.1|:30690-34611
NuwaII-9_CPB	gb|AHGY02033492.1|:833-2557
NuwaII-9_CPB	gb|AHGY02213409.1|:1135-5208
NuwaII-9_CPB	gb|AHGY02246286.1|:4831-9332
NuwaII-9_CPB	gb|AHGY02135740.1|:1-1419
NuwaII-9_CPB	gb|AHGY02216693.1|:9907-13924
NuwaII-9_CPB	gb|AHGY02076370.1|:404-4404
NuwaII-9_CPB	gb|AHGY02239541.1|:2455-7041
NuwaII-9_CPB	gb|AHGY02195189.1|:4-2029
NuwaII-9_CPB	gb|AHGY02216124.1|:7456-11789
NuwaII-9_CPB	gb|AHGY02202715.1|:32830-37613
NuwaII-9_CPB	gb|AHGY02047977.1|:5558-8903
NuwaII-9_CPB	gb|AHGY02047977.1|:275-2593
NuwaII-9_CPB	gb|AHGY02087765.1|:1454-5356
NuwaII-9_CPB	gb|AHGY02036807.1|:7898-11633
NuwaII-9_CPB	gb|AHGY02106923.1|:1555-4067
NuwaII-9_CPB	gb|AHGY02020972.1|:13520-17601
NuwaII-9_CPB	gb|AHGY02087258.1|:14719-18577

NuwaII-9_CPB	gb|AHGY02118825.1|:595-4717
NuwaII-9_CPB	gb|AHGY02107866.1|:677-4659
NuwaII-9_CPB	gb|AHGY02107866.1|:15686-20225
NuwaII-9_CPB	gb|AHGY02039631.1|:26908-31375
NuwaII-9_CPB	gb|AHGY02039631.1|:42599-45683
NuwaII-9_CPB	gb|AHGY02260999.1|:27478-31498
NuwaII-9_CPB	gb|AHGY02220896.1|:32522-38833
NuwaII-9_CPB	gb|AHGY02231345.1|:1335-5101
NuwaII-9_CPB	gb|AHGY02136829.1|:37202-37413
NuwaII-9_CPB	gb|AHGY02136829.1|:31447-37175
NuwaII-9_CPB	gb|AHGY02136829.1|:11502-15118
NuwaII-9_CPB	gb|AHGY02081464.1|:9982-14640
NuwaII-9_CPB	gb|AHGY02012927.1|:16351-19874
NuwaII-9_CPB	gb|AHGY02091886.1|:4017-6284
NuwaII-9_CPB	gb|AHGY02229853.1|:1095-4008
NuwaII-9_CPB	gb|AHGY02052044.1|:16597-20790
NuwaII-9_CPB	gb|AHGY02253602.1|:809-1527
NuwaII-9_CPB	gb|AHGY02062696.1|:1834-6753
NuwaII-9_CPB	gb|AHGY02062696.1|:2238-3228
NuwaII-9_CPB	gb|AHGY02049919.1|:18201-22286
NuwaII-9_CPB	gb|AHGY02100441.1|:11196-15329
NuwaII-9_CPB	gb|AHGY02097472.1|:9144-13076
NuwaII-9_CPB	gb|AHGY02078600.1|:19069-23152
NuwaII-9_CPB	gb|AHGY02074972.1|:19678-23588
NuwaII-9_CPB	gb|AHGY02032087.1|:3837-7320
NuwaII-9_CPB	gb|AHGY02250423.1|:27466-31528
NuwaII-9_CPB	gb|AHGY02250423.1|:2437-2513
NuwaII-9_CPB	gb|AHGY02059779.1|:3664-8001
NuwaII-9_CPB	gb|AHGY02035179.1|:33944-37994
NuwaII-9_CPB	gb|AHGY02196350.1|:88698-92802
NuwaII-9_CPB	gb|AHGY02088760.1|:3752-7788
NuwaII-9_CPB	gb|AHGY02073866.1|:1350-5452
NuwaII-9_CPB	gb|AHGY02035115.1|:7340-11264
NuwaII-9_CPB	gb|AHGY02035115.1|:13871-14582
NuwaII-9_CPB	gb|AHGY02224038.1|:10954-14762
NuwaII-9_CPB	gb|AHGY02027803.1|:2-3750
NuwaII-9_CPB	gb|AHGY02028906.1|:183-2349
NuwaII-9_CPB	gb|AHGY02208046.1|:771-4969
NuwaII-9_CPB	gb|AHGY02244229.1|:14269-18461
NuwaII-9_CPB	gb|AHGY02004461.1|:28565-32310
NuwaII-9_CPB	gb|AHGY02119746.1|:2426-2483
NuwaII-9_CPB	gb|AHGY02119746.1|:684-2725
NuwaII-9_CPB	gb|AHGY02240712.1|:374-3212
NuwaII-9_CPB	gb|AHGY02044747.1|:25286-29098

NuwaII-9_CPB	gb|AHGY02044747.1|:31868-31993
NuwaII-9_CPB	gb|AHGY02109928.1|:1534-3122
NuwaII-9_CPB	gb|AHGY02052915.1|:2974-6958
NuwaII-9_CPB	gb|AHGY02012215.1|:16455-20714
NuwaII-9_CPB	gb|AHGY02236443.1|:4853-8778
NuwaII-9_CPB	gb|AHGY02101023.1|:5993-9973
NuwaII-9_CPB	gb|AHGY02062608.1|:10435-15642
NuwaII-9_CPB	gb|AHGY02062608.1|:23826-25168
NuwaII-9_CPB	gb|AHGY02062608.1|:5243-8357
NuwaII-9_CPB	gb|AHGY02258218.1|:1598-5286
NuwaII-9_CPB	gb|AHGY02103205.1|:1433-3851
NuwaII-9_CPB	gb|AHGY02105979.1|:1203-4955
NuwaII-9_CPB	gb|AHGY02252369.1|:281-1245
NuwaII-9_CPB	gb|AHGY02252369.1|:10230-13055
NuwaII-9_CPB	gb|AHGY02072101.1|:28946-29112
NuwaII-9_CPB	gb|AHGY02072101.1|:1436-5598
NuwaII-9_CPB	gb|AHGY02047936.1|:19-1671
NuwaII-9_CPB	gb|AHGY02074667.1|:11820-14736
NuwaII-9_CPB	gb|AHGY02055910.1|:26754-26875
NuwaII-9_CPB	gb|AHGY02055910.1|:6957-10752
NuwaII-9_CPB	gb|AHGY02202439.1|:39-2153
NuwaII-9_CPB	gb|AHGY02044766.1|:5534-9946
NuwaII-9_CPB	gb|AHGY02117566.1|:1-2306
NuwaII-9_CPB	gb|AHGY02238735.1|:2366-4167
NuwaII-9_CPB	gb|AHGY02038359.1|:8429-13194
NuwaII-9_CPB	gb|AHGY02253217.1|:34729-38173
NuwaII-9_CPB	gb|AHGY02073745.1|:304-4243
NuwaII-9_CPB	gb|AHGY02252661.1|:470-3161
NuwaII-9_CPB	gb|AHGY02170614.1|:1928-3347
NuwaII-9_CPB	gb|AHGY02224111.1|:16509-21139
NuwaII-9_CPB	gb|AHGY02096938.1|:14798-19441
NuwaII-9_CPB	gb|AHGY02084778.1|:5642-10478
NuwaII-9_CPB	gb|AHGY02084778.1|:15503-15616
NuwaII-9_CPB	gb|AHGY02059772.1|:4531-8348
NuwaII-9_CPB	gb|AHGY02204601.1|:49179-51385
NuwaII-9_CPB	gb|AHGY02084662.1|:8860-12426
NuwaII-9_CPB	gb|AHGY02043602.1|:73784-77244
NuwaII-9_CPB	gb|AHGY02043602.1|:22238-22502
NuwaII-9_CPB	gb|AHGY02110470.1|:1-2328
NuwaII-9_CPB	gb|AHGY02102318.1|:5349-9576
NuwaII-9_CPB	gb|AHGY02068362.1|:23702-27537
NuwaII-9_CPB	gb|AHGY02238205.1|:10462-14621
NuwaII-9_CPB	gb|AHGY02235039.1|:26818-31869
NuwaII-9_CPB	gb|AHGY02232190.1|:19700-23383

NuwaII-9_CPB	gb|AHGY02096913.1|:9782-13861
NuwaII-9_CPB	gb|AHGY02114205.1|:924-5191
NuwaII-9_CPB	gb|AHGY02249603.1|:1113-5506
NuwaII-9_CPB	gb|AHGY02229406.1|:385-3422
NuwaII-9_CPB	gb|AHGY02104516.1|:1-2753
NuwaII-9_CPB	gb|AHGY02069782.1|:19520-23569
NuwaII-9_CPB	gb|AHGY02029342.1|:14676-18813
NuwaII-9_CPB	gb|AHGY02100799.1|:52114-53949
NuwaII-9_CPB	gb|AHGY02100799.1|:49829-52013
NuwaII-9_CPB	gb|AHGY02029293.1|:12191-16889
NuwaII-9_CPB	gb|AHGY02091325.1|:242-3066
NuwaII-9_CPB	gb|AHGY02257353.1|:3825-5690
NuwaII-9_CPB	gb|AHGY02225945.1|:6915-9173
NuwaII-9_CPB	gb|AHGY02201719.1|:13810-17904
NuwaII-9_CPB	gb|AHGY02199854.1|:24062-28353
NuwaII-9_CPB	gb|AHGY02118303.1|:2421-5544
NuwaII-9_CPB	gb|AHGY02110327.1|:350-3173
NuwaII-9_CPB	gb|AHGY02056586.1|:20415-20467
NuwaII-9_CPB	gb|AHGY02056586.1|:4655-8856
NuwaII-9_CPB	gb|AHGY02081193.1|:24669-27796
NuwaII-9_CPB	gb|AHGY02081193.1|:29242-33265
NuwaII-9_CPB	gb|AHGY02227515.1|:256-4268
NuwaII-9_CPB	gb|AHGY02085001.1|:28710-32887
NuwaII-9_CPB	gb|AHGY02061456.1|:23664-24610
NuwaII-9_CPB	gb|AHGY02061456.1|:8623-12708
NuwaII-9_CPB	gb|AHGY02035013.1|:44127-46900
NuwaII-9_CPB	gb|AHGY02210300.1|:5766-8897
NuwaII-9_CPB	gb|AHGY02208194.1|:52474-56704
NuwaII-9_CPB	gb|AHGY02053652.1|:6746-10240
NuwaII-9_CPB	gb|AHGY02016316.1|:6986-10932
NuwaII-9_CPB	gb|AHGY02225872.1|:10947-13362
NuwaII-9_CPB	gb|AHGY02195746.1|:12606-14966
NuwaII-9_CPB	gb|AHGY02220991.1|:5374-9649
NuwaII-9_CPB	gb|AHGY02110369.1|:3-3746
NuwaII-9_CPB	gb|AHGY02098629.1|:19630-23744
NuwaII-9_CPB	gb|AHGY02039312.1|:17683-21858
NuwaII-9_CPB	gb|AHGY02248671.1|:316-2498
NuwaII-9_CPB	gb|AHGY02236490.1|:27586-32888
NuwaII-9_CPB	gb|AHGY02233593.1|:1026-1224
NuwaII-9_CPB	gb|AHGY02233593.1|:22527-26929
NuwaII-9_CPB	gb|AHGY02074831.1|:4454-8611
NuwaII-9_CPB	gb|AHGY02071163.1|:134-3095
NuwaII-9_CPB	gb|AHGY02227475.1|:35052-38290
NuwaII-9_CPB	gb|AHGY02048031.1|:2065-6278

NuwaII-9_CPB	gb|AHGY02021000.1|:2325-6895
NuwaII-9_CPB	gb|AHGY02224275.1|:7025-11243
NuwaII-9_CPB	gb|AHGY02057720.1|:2599-6819
NuwaII-9_CPB	gb|AHGY02036128.1|:16537-20155
NuwaII-9_CPB	gb|AHGY02114681.1|:996-4415
NuwaII-9_CPB	gb|AHGY02102519.1|:6001-9979
NuwaII-9_CPB	gb|AHGY02011159.1|:3351-7442
NuwaII-9_CPB	gb|AHGY02257451.1|:24338-29204
NuwaII-9_CPB	gb|AHGY02257451.1|:39267-43503
NuwaII-9_CPB	gb|AHGY02109753.1|:11678-15784
NuwaII-9_CPB	gb|AHGY02109753.1|:6917-6986
NuwaII-9_CPB	gb|AHGY02067331.1|:456-4084
NuwaII-9_CPB	gb|AHGY02061757.1|:4845-8559
NuwaII-9_CPB	gb|AHGY02044054.1|:2627-7477
NuwaII-9_CPB	gb|AHGY02024583.1|:4-1172
NuwaII-9_CPB	gb|AHGY02085105.1|:3776-7688
NuwaII-9_CPB	gb|AHGY02049830.1|:14707-18939
NuwaII-9_CPB	gb|AHGY02036169.1|:11256-15501
NuwaII-9_CPB	gb|AHGY02004209.1|:133-2832
NuwaII-9_CPB	gb|AHGY02000634.1|:4539-8673
NuwaII-9_CPB	gb|AHGY02090732.1|:2758-6433
NuwaII-9_CPB	gb|AHGY02090732.1|:2642-2756
NuwaII-9_CPB	gb|AHGY02200310.1|:11742-13105
NuwaII-9_CPB	gb|AHGY02106778.1|:4340-8581
NuwaII-9_CPB	gb|AHGY02219338.1|:6810-10871
NuwaII-9_CPB	gb|AHGY02219338.1|:45735-47499
NuwaII-9_CPB	gb|AHGY02211461.1|:2115-6366
NuwaII-9_CPB	gb|AHGY02114167.1|:1511-2278
NuwaII-9_CPB	gb|AHGY02114167.1|:68-2968
NuwaII-9_CPB	gb|AHGY02060080.1|:2436-4773
NuwaII-9_CPB	gb|AHGY02118084.1|:5-2200
NuwaII-9_CPB	gb|AHGY02237595.1|:3692-8614
NuwaII-9_CPB	gb|AHGY02234716.1|:17657-20075
NuwaII-9_CPB	gb|AHGY02060933.1|:11712-17035
NuwaII-9_CPB	gb|AHGY02247081.1|:18375-22666
NuwaII-9_CPB	gb|AHGY02241538.1|:12269-14650
NuwaII-9_CPB	gb|AHGY02245585.1|:11683-13968
NuwaII-9_CPB	gb|AHGY02240340.1|:655-2673
NuwaII-9_CPB	gb|AHGY02211428.1|:1900-6147
NuwaII-9_CPB	gb|AHGY02211428.1|:14041-18151
NuwaII-9_CPB	gb|AHGY02210277.1|:12867-14624
NuwaII-9_CPB	gb|AHGY02195903.1|:25540-29634
NuwaII-9_CPB	gb|AHGY02077916.1|:10659-15181
NuwaII-9_CPB	gb|AHGY02047457.1|:144-1757

NuwaII-9_CPB	gb|AHGY02007244.1|:3129-7265
NuwaII-9_CPB	gb|AHGY02001913.1|:11725-15842
NuwaII-9_CPB	gb|AHGY02243489.1|:13059-13194
NuwaII-9_CPB	gb|AHGY02243489.1|:135-2413
NuwaII-9_CPB	gb|AHGY02220960.1|:11422-15419
NuwaII-9_CPB	gb|AHGY02220960.1|:30-1526
NuwaII-9_CPB	gb|AHGY02046806.1|:27-2635
NuwaII-9_CPB	gb|AHGY02052813.1|:5767-9576
NuwaII-9_CPB	gb|AHGY02029330.1|:41045-41124
NuwaII-9_CPB	gb|AHGY02029330.1|:279-2277
NuwaII-9_CPB	gb|AHGY02100070.1|:2228-4762
NuwaII-9_CPB	gb|AHGY02038382.1|:124680-126559
NuwaII-9_CPB	gb|AHGY02038382.1|:62975-67129
NuwaII-9_CPB	gb|AHGY02034535.1|:8627-11047
NuwaII-9_CPB	gb|AHGY02261907.1|:169-2932
NuwaII-9_CPB	gb|AHGY02235187.1|:13281-17480
NuwaII-9_CPB	gb|AHGY02223418.1|:93-2150
NuwaII-9_CPB	gb|AHGY02098689.1|:24340-24498
NuwaII-9_CPB	gb|AHGY02098689.1|:1724-6539
NuwaII-9_CPB	gb|AHGY02090559.1|:26978-30775
NuwaII-9_CPB	gb|AHGY02014858.1|:1836-6687
NuwaII-9_CPB	gb|AHGY02001584.1|:6840-11012
NuwaII-9_CPB	gb|AHGY02252834.1|:879-3912
NuwaII-10_CPB	gb|AHGY02086867.1|:19915-23888
NuwaII-10_CPB	gb|AHGY02052531.1|:3433-8414
NuwaII-10_CPB	gb|AHGY02245731.1|:2671-6490
NuwaII-10_CPB	gb|AHGY02011165.1|:11183-14619
NuwaII-10_CPB	gb|AHGY02214410.1|:1-2563
NuwaII-10_CPB	gb|AHGY02039133.1|:6692-11082
NuwaII-10_CPB	gb|AHGY02047322.1|:28121-31115
NuwaII-10_CPB	gb|AHGY02014792.1|:7135-11611
NuwaII-10_CPB	gb|AHGY02093691.1|:7969-12303
NuwaII-10_CPB	gb|AHGY02218895.1|:24757-30378
NuwaII-10_CPB	gb|AHGY02100066.1|:1106-4959
NuwaII-10_CPB	gb|AHGY02237558.1|:6707-10162
NuwaII-10_CPB	gb|AHGY02221017.1|:3756-8172
NuwaII-10_CPB	gb|AHGY02084322.1|:14094-17980
NuwaII-10_CPB	gb|AHGY02260615.1|:20556-24591
NuwaII-10_CPB	gb|AHGY02257746.1|:4-1982
NuwaII-10_CPB	gb|AHGY02235720.1|:321-2848
NuwaII-10_CPB	gb|AHGY02087659.1|:1519-5563
NuwaII-10_CPB	gb|AHGY02065298.1|:4859-8256
NuwaII-10_CPB	gb|AHGY02110679.1|:7171-10601
NuwaII-10_CPB	gb|AHGY02015406.1|:78-4403

NuwaII-10_CPB	gb|AHGY02066658.1|:1646-6353
NuwaII-10_CPB	gb|AHGY02066658.1|:12719-16675
NuwaII-10_CPB	gb|AHGY02076370.1|:404-4404
NuwaII-10_CPB	gb|AHGY02062392.1|:6140-10161
NuwaII-10_CPB	gb|AHGY02009560.1|:1249-5565
NuwaII-10_CPB	gb|AHGY02000719.1|:29934-34096
NuwaII-10_CPB	gb|AHGY02044953.1|:1981-5916
NuwaII-10_CPB	gb|AHGY02200659.1|:29579-33750
NuwaII-10_CPB	gb|AHGY02064190.1|:9052-11823
NuwaII-10_CPB	gb|AHGY02239448.1|:40930-45572
NuwaII-10_CPB	gb|AHGY02036807.1|:7911-11633
NuwaII-10_CPB	gb|AHGY02198146.1|:16-2311
NuwaII-10_CPB	gb|AHGY02049919.1|:18201-22286
NuwaII-10_CPB	gb|AHGY02202715.1|:32830-37613
NuwaII-10_CPB	gb|AHGY02101683.1|:990-4887
NuwaII-10_CPB	gb|AHGY02068485.1|:17363-20775
NuwaII-10_CPB	gb|AHGY02223746.1|:13-1517
NuwaII-10_CPB	gb|AHGY02028326.1|:22717-27643
NuwaII-10_CPB	gb|AHGY02206833.1|:5970-10216
NuwaII-10_CPB	gb|AHGY02090059.1|:15708-18058
NuwaII-10_CPB	gb|AHGY02087765.1|:1454-5356
NuwaII-10_CPB	gb|AHGY02052881.1|:33963-37686
NuwaII-10_CPB	gb|AHGY02052881.1|:9103-9715
NuwaII-10_CPB	gb|AHGY02213252.1|:16626-20000
NuwaII-10_CPB	gb|AHGY02218703.1|:3662-7683
NuwaII-10_CPB	gb|AHGY02107866.1|:677-4659
NuwaII-10_CPB	gb|AHGY02107866.1|:15686-20225
NuwaII-10_CPB	gb|AHGY02046875.1|:131-5498
NuwaII-10_CPB	gb|AHGY02234830.1|:31401-32920
NuwaII-10_CPB	gb|AHGY02229853.1|:1095-3402
NuwaII-10_CPB	gb|AHGY02241355.1|:1515-5663
NuwaII-10_CPB	gb|AHGY02031362.1|:7769-11697
NuwaII-10_CPB	gb|AHGY02023196.1|:613-662
NuwaII-10_CPB	gb|AHGY02023196.1|:22422-26567
NuwaII-10_CPB	gb|AHGY02105442.1|:411-5638
NuwaII-10_CPB	gb|AHGY02032428.1|:62375-66327
NuwaII-10_CPB	gb|AHGY02260999.1|:27478-31503
NuwaII-10_CPB	gb|AHGY02247667.1|:66861-70180
NuwaII-10_CPB	gb|AHGY02084153.1|:67798-72044
NuwaII-10_CPB	gb|AHGY02035179.1|:33944-37994
NuwaII-10_CPB	gb|AHGY02035115.1|:7340-11264
NuwaII-10_CPB	gb|AHGY02035115.1|:13871-15171
NuwaII-10_CPB	gb|AHGY02207552.1|:1686-5610
NuwaII-10_CPB	gb|AHGY02100441.1|:11196-15329

NuwaII-10_CPB	gb|AHGY02044747.1|:25286-29098
NuwaII-10_CPB	gb|AHGY02044747.1|:31868-31993
NuwaII-10_CPB	gb|AHGY02074972.1|:19678-23588
NuwaII-10_CPB	gb|AHGY02239541.1|:2455-6279
NuwaII-10_CPB	gb|AHGY02136829.1|:37202-37413
NuwaII-10_CPB	gb|AHGY02136829.1|:31447-37175
NuwaII-10_CPB	gb|AHGY02136829.1|:11502-15118
NuwaII-10_CPB	gb|AHGY02210088.1|:1749-5751
NuwaII-10_CPB	gb|AHGY02253602.1|:2366-3950
NuwaII-10_CPB	gb|AHGY02258218.1|:1598-5286
NuwaII-10_CPB	gb|AHGY02052915.1|:2974-6958
NuwaII-10_CPB	gb|AHGY02047977.1|:5558-8903
NuwaII-10_CPB	gb|AHGY02047977.1|:263-2765
NuwaII-10_CPB	gb|AHGY02043075.1|:34981-39304
NuwaII-10_CPB	gb|AHGY02216124.1|:7456-11789
NuwaII-10_CPB	gb|AHGY02059779.1|:3664-8001
NuwaII-10_CPB	gb|AHGY02196127.1|:3123-7197
NuwaII-10_CPB	gb|AHGY02062696.1|:1834-6753
NuwaII-10_CPB	gb|AHGY02062696.1|:2238-3228
NuwaII-10_CPB	gb|AHGY02206211.1|:8773-12616
NuwaII-10_CPB	gb|AHGY02072101.1|:28946-29112
NuwaII-10_CPB	gb|AHGY02072101.1|:1443-5598
NuwaII-10_CPB	gb|AHGY02196245.1|:298-3041
NuwaII-10_CPB	gb|AHGY02208524.1|:347-3546
NuwaII-10_CPB	gb|AHGY02073866.1|:1350-5452
NuwaII-10_CPB	gb|AHGY02011125.1|:1-271
NuwaII-10_CPB	gb|AHGY02011125.1|:13734-17932
NuwaII-10_CPB	gb|AHGY02224038.1|:10954-14762
NuwaII-10_CPB	gb|AHGY02055909.1|:7903-12113
NuwaII-10_CPB	gb|AHGY02223285.1|:13358-18012
NuwaII-10_CPB	gb|AHGY02224111.1|:16509-21139
NuwaII-10_CPB	gb|AHGY02236490.1|:33615-33718
NuwaII-10_CPB	gb|AHGY02236490.1|:27586-32888
NuwaII-10_CPB	gb|AHGY02106923.1|:1555-4063
NuwaII-10_CPB	gb|AHGY02229406.1|:385-3422
NuwaII-10_CPB	gb|AHGY02238721.1|:14860-18973
NuwaII-10_CPB	gb|AHGY02044628.1|:1466-6038
NuwaII-10_CPB	gb|AHGY02062627.1|:29974-34170
NuwaII-10_CPB	gb|AHGY02062627.1|:13313-17283
NuwaII-10_CPB	gb|AHGY02044766.1|:5534-9946
NuwaII-10_CPB	gb|AHGY02029293.1|:12191-15955
NuwaII-10_CPB	gb|AHGY02210767.1|:30690-34611
NuwaII-10_CPB	gb|AHGY02118825.1|:595-4717
NuwaII-10_CPB	gb|AHGY02038359.1|:8429-12167

NuwaII-10_CPB	gb|AHGY02198795.1|:583-3073
NuwaII-10_CPB	gb|AHGY02246286.1|:4831-9332
NuwaII-10_CPB	gb|AHGY02235039.1|:26818-31869
NuwaII-10_CPB	gb|AHGY02232190.1|:19700-23383
NuwaII-10_CPB	gb|AHGY02089959.1|:32388-36346
NuwaII-10_CPB	gb|AHGY02081193.1|:24669-27796
NuwaII-10_CPB	gb|AHGY02081193.1|:29242-33265
NuwaII-10_CPB	gb|AHGY02236577.1|:32093-35474
NuwaII-10_CPB	gb|AHGY02204601.1|:48054-51380
NuwaII-10_CPB	gb|AHGY02078873.1|:14071-17816
NuwaII-10_CPB	gb|AHGY02096913.1|:9782-14929
NuwaII-10_CPB	gb|AHGY02252661.1|:470-3161
NuwaII-10_CPB	gb|AHGY02012927.1|:16162-19874
NuwaII-10_CPB	gb|AHGY02109928.1|:1649-3171
NuwaII-10_CPB	gb|AHGY02097472.1|:9144-13076
NuwaII-10_CPB	gb|AHGY02060080.1|:2436-4773
NuwaII-10_CPB	gb|AHGY02089719.1|:12056-16407
NuwaII-10_CPB	gb|AHGY02089719.1|:137-1561
NuwaII-10_CPB	gb|AHGY02117566.1|:1-2306
NuwaII-10_CPB	gb|AHGY02078600.1|:19069-23152
NuwaII-10_CPB	gb|AHGY02062819.1|:21728-25991
NuwaII-10_CPB	gb|AHGY02227515.1|:256-4268
NuwaII-10_CPB	gb|AHGY02078346.1|:37102-42573
NuwaII-10_CPB	gb|AHGY02078346.1|:37731-38592
NuwaII-10_CPB	gb|AHGY02027803.1|:2-3750
NuwaII-10_CPB	gb|AHGY02084778.1|:6522-10478
NuwaII-10_CPB	gb|AHGY02084778.1|:15503-15615
NuwaII-10_CPB	gb|AHGY02073745.1|:304-4243
NuwaII-10_CPB	gb|AHGY02087258.1|:14766-18577
NuwaII-10_CPB	gb|AHGY02257451.1|:24338-29204
NuwaII-10_CPB	gb|AHGY02257451.1|:39267-43503
NuwaII-10_CPB	gb|AHGY02247627.1|:15877-19963
NuwaII-10_CPB	gb|AHGY02032087.1|:3837-7320
NuwaII-10_CPB	gb|AHGY02105979.1|:1203-4955
NuwaII-10_CPB	gb|AHGY02257353.1|:3825-5694
NuwaII-10_CPB	gb|AHGY02014858.1|:1836-6687
NuwaII-10_CPB	gb|AHGY02104516.1|:1-2752
NuwaII-10_CPB	gb|AHGY02102519.1|:5997-9979
NuwaII-10_CPB	gb|AHGY02096938.1|:14798-19441
NuwaII-10_CPB	gb|AHGY02061757.1|:4845-8559
NuwaII-10_CPB	gb|AHGY02195746.1|:12606-14967
NuwaII-10_CPB	gb|AHGY02106778.1|:4340-8581
NuwaII-10_CPB	gb|AHGY02224185.1|:17348-18437
NuwaII-10_CPB	gb|AHGY02224185.1|:16710-21621

NuwaII-10_CPB	gb|AHGY02036169.1|:11256-15501
NuwaII-10_CPB	gb|AHGY02232627.1|:10579-12492
NuwaII-10_CPB	gb|AHGY02102318.1|:5349-9497
NuwaII-10_CPB	gb|AHGY02039312.1|:17683-21858
NuwaII-10_CPB	gb|AHGY02029134.1|:19098-23674
NuwaII-10_CPB	gb|AHGY02103205.1|:1433-3864
NuwaII-10_CPB	gb|AHGY02249939.1|:27386-31926
NuwaII-10_CPB	gb|AHGY02240712.1|:355-3212
NuwaII-10_CPB	gb|AHGY02195189.1|:6-2030
NuwaII-10_CPB	gb|AHGY02088760.1|:3752-7788
NuwaII-10_CPB	gb|AHGY02046806.1|:1-2211
NuwaII-10_CPB	gb|AHGY02039631.1|:26908-31375
NuwaII-10_CPB	gb|AHGY02039631.1|:44704-45683
NuwaII-10_CPB	gb|AHGY02008870.1|:6640-10993
NuwaII-10_CPB	gb|AHGY02044054.1|:2627-7477
NuwaII-10_CPB	gb|AHGY02101023.1|:5989-9973
NuwaII-10_CPB	gb|AHGY02056586.1|:4655-8856
NuwaII-10_CPB	gb|AHGY02021000.1|:2325-6895
NuwaII-10_CPB	gb|AHGY02004461.1|:28565-32310
NuwaII-10_CPB	gb|AHGY02244229.1|:14269-18461
NuwaII-10_CPB	gb|AHGY02253217.1|:34719-38173
NuwaII-10_CPB	gb|AHGY02250423.1|:27466-31528
NuwaII-10_CPB	gb|AHGY02250423.1|:2437-2513
NuwaII-10_CPB	gb|AHGY02118084.1|:5-2200
NuwaII-10_CPB	gb|AHGY02110470.1|:4-2313
NuwaII-10_CPB	gb|AHGY02195903.1|:25540-29638
NuwaII-10_CPB	gb|AHGY02237595.1|:3692-8614
NuwaII-10_CPB	gb|AHGY02201565.1|:29144-31479
NuwaII-10_CPB	gb|AHGY02091325.1|:59-3066
NuwaII-10_CPB	gb|AHGY02236443.1|:4853-8778
NuwaII-10_CPB	gb|AHGY02036066.1|:24909-28960
NuwaII-10_CPB	gb|AHGY02036066.1|:39863-40003
NuwaII-10_CPB	gb|AHGY02216693.1|:9907-13924
NuwaII-10_CPB	gb|AHGY02052205.1|:9748-14013
NuwaII-10_CPB	gb|AHGY02033492.1|:833-2557
NuwaII-10_CPB	gb|AHGY02231014.1|:30050-34282
NuwaII-10_CPB	gb|AHGY02236016.1|:4968-9109
NuwaII-10_CPB	gb|AHGY02208046.1|:771-4969
NuwaII-10_CPB	gb|AHGY02256731.1|:10256-14843
NuwaII-10_CPB	gb|AHGY02100070.1|:2228-4762
NuwaII-10_CPB	gb|AHGY02246369.1|:9359-13196
NuwaII-10_CPB	gb|AHGY02077374.1|:150-2129
NuwaII-10_CPB	gb|AHGY02196350.1|:88698-92802
NuwaII-10_CPB	gb|AHGY02012215.1|:16455-20707

NuwaII-10_CPB	gb|AHGY02135740.1|:1-1419
NuwaII-10_CPB	gb|AHGY02074467.1|:23684-28587
NuwaII-10_CPB	gb|AHGY02170614.1|:1996-3407
NuwaII-10_CPB	gb|AHGY02047770.1|:9267-12383
NuwaII-10_CPB	gb|AHGY02038382.1|:124680-126555
NuwaII-10_CPB	gb|AHGY02038382.1|:62975-67129
NuwaII-10_CPB	gb|AHGY02213409.1|:1153-5208
NuwaII-10_CPB	gb|AHGY02252369.1|:285-922
NuwaII-10_CPB	gb|AHGY02252369.1|:10230-13056
NuwaII-10_CPB	gb|AHGY02223282.1|:38875-43091
NuwaII-10_CPB	gb|AHGY02005141.1|:4847-9768
NuwaII-10_CPB	gb|AHGY02020985.1|:5693-9909
NuwaII-10_CPB	gb|AHGY02098629.1|:19630-23744
NuwaII-10_CPB	gb|AHGY02083322.1|:461-3395
NuwaII-10_CPB	gb|AHGY02077019.1|:274-2530
NuwaII-10_CPB	gb|AHGY02055525.1|:82-1583
NuwaII-10_CPB	gb|AHGY02220896.1|:32522-38833
NuwaII-10_CPB	gb|AHGY02091886.1|:4017-6284
NuwaII-10_CPB	gb|AHGY02251025.1|:48-1640
NuwaII-10_CPB	gb|AHGY02043602.1|:73784-77236
NuwaII-10_CPB	gb|AHGY02043602.1|:22238-22332
NuwaII-10_CPB	gb|AHGY02011159.1|:3351-7442
NuwaII-10_CPB	gb|AHGY02114167.1|:1511-2278
NuwaII-10_CPB	gb|AHGY02114167.1|:5-2968
NuwaII-10_CPB	gb|AHGY02062608.1|:10435-15519
NuwaII-10_CPB	gb|AHGY02062608.1|:23826-25168
NuwaII-10_CPB	gb|AHGY02062608.1|:5284-8357
NuwaII-10_CPB	gb|AHGY02013523.1|:306-3619
NuwaII-10_CPB	gb|AHGY02056495.1|:148-3471
NuwaII-10_CPB	gb|AHGY02032553.1|:209-3524
NuwaII-10_CPB	gb|AHGY02029342.1|:14577-18813
NuwaII-10_CPB	gb|AHGY02055956.1|:257-959
NuwaII-10_CPB	gb|AHGY02055956.1|:26841-27268
NuwaII-10_CPB	gb|AHGY02055956.1|:4966-9988
NuwaII-10_CPB	gb|AHGY02077288.1|:38883-41824
NuwaII-10_CPB	gb|AHGY02035999.1|:1221-6094
NuwaII-10_CPB	gb|AHGY02084662.1|:8773-12426
NuwaII-10_CPB	gb|AHGY02062114.1|:1-4080
NuwaII-10_CPB	gb|AHGY02015881.1|:7290-10488
NuwaII-10_CPB	gb|AHGY02253796.1|:31-2593
NuwaII-10_CPB	gb|AHGY02231345.1|:1335-5101
NuwaII-10_CPB	gb|AHGY02222453.1|:12668-15949
NuwaII-10_CPB	gb|AHGY02208231.1|:6307-9613
NuwaII-10_CPB	gb|AHGY02114816.1|:3961-6156

NuwaII-10_CPB	gb|AHGY02118303.1|:2421-5556
NuwaII-10_CPB	gb|AHGY02110327.1|:185-3173
NuwaII-10_CPB	gb|AHGY02249088.1|:13-1709
NuwaII-10_CPB	gb|AHGY02238735.1|:2366-4171
NuwaII-10_CPB	gb|AHGY02099855.1|:3655-8326
NuwaII-10_CPB	gb|AHGY02059772.1|:4531-8348
NuwaII-10_CPB	gb|AHGY02049334.1|:1760-2001
NuwaII-10_CPB	gb|AHGY02049334.1|:8-1348
NuwaII-10_CPB	gb|AHGY02055910.1|:26759-26875
NuwaII-10_CPB	gb|AHGY02055910.1|:6957-10750
NuwaII-10_CPB	gb|AHGY02253428.1|:59240-62503
NuwaII-10_CPB	gb|AHGY02225559.1|:8183-11526
NuwaII-10_CPB	gb|AHGY02100972.1|:2029-7008
NuwaII-10_CPB	gb|AHGY02047358.1|:2138-4943
NuwaII-10_CPB	gb|AHGY02080198.1|:9741-13962
NuwaII-10_CPB	gb|AHGY02201227.1|:10269-14019
NuwaII-10_CPB	gb|AHGY02246044.1|:41834-44866
NuwaII-10_CPB	gb|AHGY02246044.1|:32507-32580
NuwaII-10_CPB	gb|AHGY02259294.1|:40473-43759
NuwaII-10_CPB	gb|AHGY02068362.1|:23702-27666
NuwaII-10_CPB	gb|AHGY02248671.1|:574-2498
NuwaII-10_CPB	gb|AHGY02226247.1|:3346-6179
NuwaII-10_CPB	gb|AHGY02219343.1|:219-3605
NuwaII-10_CPB	gb|AHGY02063001.1|:2767-6957
NuwaII-10_CPB	gb|AHGY02026112.1|:19147-23987
NuwaII-10_CPB	gb|AHGY02221873.1|:10279-14297
NuwaII-10_CPB	gb|AHGY02261612.1|:4965-9969
NuwaII-10_CPB	gb|AHGY02234742.1|:143-2872
NuwaII-10_CPB	gb|AHGY02233593.1|:1014-1224
NuwaII-10_CPB	gb|AHGY02233593.1|:22527-26929
NuwaII-10_CPB	gb|AHGY02072511.1|:5259-5444
NuwaII-10_CPB	gb|AHGY02072511.1|:1-2450
NuwaII-10_CPB	gb|AHGY02047936.1|:1-1586
NuwaII-10_CPB	gb|AHGY02235979.1|:13367-17715
NuwaII-10_CPB	gb|AHGY02106064.1|:194-3115
NuwaII-10_CPB	gb|AHGY02078045.1|:79-1380
NuwaII-10_CPB	gb|AHGY02035013.1|:44127-46900
NuwaII-10_CPB	gb|AHGY02254268.1|:1753-6557
NuwaII-10_CPB	gb|AHGY02221098.1|:5-1280
NuwaII-10_CPB	gb|AHGY02211538.1|:1736-5449
NuwaII-10_CPB	gb|AHGY02199854.1|:24062-28353
NuwaII-10_CPB	gb|AHGY02099477.1|:112-3124
NuwaII-10_CPB	gb|AHGY02253907.1|:257-4040
NuwaII-10_CPB	gb|AHGY02247081.1|:18375-22666

NuwaII-10_CPB	gb|AHGY02085105.1|:3776-7688
NuwaII-11_CPB	gb|AHGY02023530.1|:38122-40671
NuwaII-11_CPB	gb|AHGY02253346.1|:1142-4944
NuwaII-11_CPB	gb|AHGY02055480.1|:5604-10090
NuwaII-11_CPB	gb|AHGY02260424.1|:1296-5129
NuwaII-11_CPB	gb|AHGY02008041.1|:6367-8713
NuwaII-11_CPB	gb|AHGY02067253.1|:21763-25572
NuwaII-11_CPB	gb|AHGY02259507.1|:7232-10955
NuwaII-11_CPB	gb|AHGY02259507.1|:15962-16109
NuwaII-11_CPB	gb|AHGY02227284.1|:32080-35928
NuwaII-11_CPB	gb|AHGY02225606.1|:33744-38806
NuwaII-11_CPB	gb|AHGY02069054.1|:776-4692
NuwaII-11_CPB	gb|AHGY02011861.1|:41460-44810
NuwaII-11_CPB	gb|AHGY02078511.1|:297-2629
NuwaII-11_CPB	gb|AHGY02023403.1|:20339-28731
NuwaII-11_CPB	gb|AHGY02219293.1|:14345-17715
NuwaII-11_CPB	gb|AHGY02021575.1|:7415-9594
NuwaII-11_CPB	gb|AHGY02024075.1|:94-2861
NuwaII-11_CPB	gb|AHGY02199854.1|:24993-25420
NuwaII-11_CPB	gb|AHGY02199854.1|:6493-10275
NuwaII-11_CPB	gb|AHGY02055994.1|:10211-13449
NuwaII-11_CPB	gb|AHGY02020840.1|:5113-8945
NuwaII-11_CPB	gb|AHGY02019362.1|:5032-9146
NuwaII-11_CPB	gb|AHGY02023163.1|:1109-4878
NuwaII-11_CPB	gb|AHGY02208585.1|:14887-19033
NuwaII-11_CPB	gb|AHGY02042505.1|:26202-28597
NuwaII-11_CPB	gb|AHGY02105432.1|:2770-6644
NuwaII-11_CPB	gb|AHGY02251120.1|:3405-5512
NuwaII-11_CPB	gb|AHGY02043998.1|:7095-8848
NuwaII-11_CPB	gb|AHGY02043774.1|:435-3014
NuwaII-11_CPB	gb|AHGY02029582.1|:10546-14385
NuwaII-11_CPB	gb|AHGY02062973.1|:775-4546
NuwaII-11_CPB	gb|AHGY02052391.1|:6519-10018
NuwaII-11_CPB	gb|AHGY02064359.1|:108-2319
NuwaII-11_CPB	gb|AHGY02200801.1|:126-175
NuwaII-11_CPB	gb|AHGY02200801.1|:25799-29474
NuwaII-11_CPB	gb|AHGY02255404.1|:4984-7078
NuwaII-11_CPB	gb|AHGY02206134.1|:28584-30179
NuwaII-11_CPB	gb|AHGY02075886.1|:3601-6869
NuwaII-11_CPB	gb|AHGY02090596.1|:24267-28661
NuwaII-11_CPB	gb|AHGY02210811.1|:15787-19668
NuwaII-11_CPB	gb|AHGY02015111.1|:22399-24242
NuwaII-11_CPB	gb|AHGY02237381.1|:2114-3534
NuwaII-11_CPB	gb|AHGY02000073.1|:7337-11537

NuwaII-11_CPB	gb|AHGY02004061.1|:12677-13705
NuwaII-11_CPB	gb|AHGY02004061.1|:27565-28795
NuwaII-11_CPB	gb|AHGY02052264.1|:1927-5709
NuwaII-11_CPB	gb|AHGY02083231.1|:2052-5363
NuwaII-11_CPB	gb|AHGY02078228.1|:866-5278
NuwaII-11_CPB	gb|AHGY02044132.1|:12614-16875
NuwaII-11_CPB	gb|AHGY02022912.1|:3551-7126
NuwaII-11_CPB	gb|AHGY02079484.1|:3203-4629
NuwaII-11_CPB	gb|AHGY02259894.1|:24859-24914
NuwaII-11_CPB	gb|AHGY02259894.1|:41461-44067
NuwaII-11_CPB	gb|AHGY02017649.1|:33948-35628
NuwaII-11_CPB	gb|AHGY02040603.1|:6644-9828
NuwaII-11_CPB	gb|AHGY02008264.1|:11123-14134
NuwaII-11_CPB	gb|AHGY02087593.1|:8468-18956
NuwaII-11_CPB	gb|AHGY02087593.1|:3483-3910
NuwaII-11_CPB	gb|AHGY02044010.1|:11577-12829
NuwaII-11_CPB	gb|AHGY02242318.1|:16651-19704
NuwaII-11_CPB	gb|AHGY02063422.1|:34029-37765
NuwaII-11_CPB	gb|AHGY02220955.1|:9472-13353
NuwaII-11_CPB	gb|AHGY02223363.1|:7898-11448
NuwaII-11_CPB	gb|AHGY02000132.1|:51-1559
NuwaII-11_CPB	gb|AHGY02248559.1|:2647-4432
NuwaII-11_CPB	gb|AHGY02011775.1|:12151-16228
NuwaII-11_CPB	gb|AHGY02011775.1|:108-280
NuwaII-11_CPB	gb|AHGY02055967.1|:45042-47491
NuwaII-11_CPB	gb|AHGY02231483.1|:2405-5731
NuwaII-11_CPB	gb|AHGY02198038.1|:880-2988
NuwaII-11_CPB	gb|AHGY02212470.1|:83998-88148
NuwaII-11_CPB	gb|AHGY02244135.1|:29245-33845
NuwaII-11_CPB	gb|AHGY02240850.1|:28393-28442
NuwaII-11_CPB	gb|AHGY02240850.1|:21812-25293
NuwaII-11_CPB	gb|AHGY02218330.1|:1213-4057
NuwaII-11_CPB	gb|AHGY02056525.1|:2861-4159
NuwaII-11_CPB	gb|AHGY02067259.1|:8763-12177
NuwaII-11_CPB	gb|AHGY02067259.1|:30303-31047
NuwaII-11_CPB	gb|AHGY02204851.1|:17509-21643
NuwaII-11_CPB	gb|AHGY02040780.1|:1666-5402
NuwaII-11_CPB	gb|AHGY02252614.1|:8604-12359
NuwaII-11_CPB	gb|AHGY02232380.1|:2377-6147
NuwaII-11_CPB	gb|AHGY02090724.1|:35-2020
NuwaII-11_CPB	gb|AHGY02075905.1|:23052-23469
NuwaII-11_CPB	gb|AHGY02075905.1|:256-2416
NuwaII-11_CPB	gb|AHGY02260425.1|:472-1485
NuwaII-11_CPB	gb|AHGY02216267.1|:10818-12739

NuwaII-11_CPB	gb|AHGY02216267.1|:23829-25439
NuwaII-11_CPB	gb|AHGY02206775.1|:26982-30780
NuwaII-11_CPB	gb|AHGY02206775.1|:78833-81206
NuwaII-11_CPB	gb|AHGY02073340.1|:2478-8306
NuwaII-11_CPB	gb|AHGY02073340.1|:5825-9287
NuwaII-11_CPB	gb|AHGY02097227.1|:4705-9083
NuwaII-11_CPB	gb|AHGY02227334.1|:1-3213
NuwaII-11_CPB	gb|AHGY02225577.1|:24182-27681
NuwaII-11_CPB	gb|AHGY02010340.1|:2640-4491
NuwaII-11_CPB	gb|AHGY02072164.1|:5038-8884
NuwaII-11_CPB	gb|AHGY02072164.1|:21499-21564
NuwaII-11_CPB	gb|AHGY02062366.1|:461-1681
NuwaII-11_CPB	gb|AHGY02247251.1|:21924-26060
NuwaII-11_CPB	gb|AHGY02234359.1|:677-3916
NuwaII-11_CPB	gb|AHGY02244845.1|:2-2796
NuwaII-11_CPB	gb|AHGY02057300.1|:1274-1330
NuwaII-11_CPB	gb|AHGY02057300.1|:7466-11467
NuwaII-11_CPB	gb|AHGY02023637.1|:4377-8123
NuwaII-11_CPB	gb|AHGY02222390.1|:32920-36650
NuwaII-11_CPB	gb|AHGY02207868.1|:1746-2724
NuwaII-11_CPB	gb|AHGY02069380.1|:25942-27656
NuwaII-11_CPB	gb|AHGY02069380.1|:38323-41988
NuwaII-11_CPB	gb|AHGY02130517.1|:2-1948
NuwaII-11_CPB	gb|AHGY02231258.1|:20829-24546
NuwaII-11_CPB	gb|AHGY02052206.1|:31421-34957
NuwaII-11_CPB	gb|AHGY02044086.1|:1894-6403
NuwaII-11_CPB	gb|AHGY02082755.1|:349-3734
NuwaII-11_CPB	gb|AHGY02064297.1|:49-3397
NuwaII-11_CPB	gb|AHGY02070011.1|:160953-161003
NuwaII-11_CPB	gb|AHGY02070011.1|:122088-123553
NuwaII-11_CPB	gb|AHGY02076919.1|:32235-35860
NuwaII-11_CPB	gb|AHGY02076919.1|:20621-20822
NuwaII-11_CPB	gb|AHGY02076919.1|:5755-5901
NuwaII-11_CPB	gb|AHGY02199847.1|:27807-30792
NuwaII-11_CPB	gb|AHGY02221021.1|:4332-5696
NuwaII-11_CPB	gb|AHGY02221021.1|:2322-2819
NuwaII-11_CPB	gb|AHGY02251907.1|:19829-24312
NuwaII-11_CPB	gb|AHGY02044875.1|:7917-11915
NuwaII-11_CPB	gb|AHGY02074523.1|:23826-28444
NuwaII-11_CPB	gb|AHGY02210296.1|:35975-39616
NuwaII-11_CPB	gb|AHGY02210296.1|:7-176
NuwaII-11_CPB	gb|AHGY02227296.1|:7992-9382
NuwaII-11_CPB	gb|AHGY02236729.1|:241-2784
NuwaII-11_CPB	gb|AHGY02200192.1|:390-1847

NuwaII-11_CPB	gb|AHGY02257574.1|:3290-6777
NuwaII-11_CPB	gb|AHGY02212150.1|:20896-21353
NuwaII-11_CPB	gb|AHGY02212150.1|:50774-50826
NuwaII-11_CPB	gb|AHGY02212150.1|:44023-47620
NuwaII-11_CPB	gb|AHGY02019673.1|:18227-22803
NuwaII-11_CPB	gb|AHGY02019673.1|:23568-23673
NuwaII-11_CPB	gb|AHGY02244716.1|:2397-4912
NuwaII-11_CPB	gb|AHGY02080931.1|:3915-7520
NuwaII-11_CPB	gb|AHGY02230863.1|:37433-41104
NuwaII-11_CPB	gb|AHGY02254137.1|:626-2872
NuwaII-11_CPB	gb|AHGY02239700.1|:1069-4506
NuwaII-11_CPB	gb|AHGY02085428.1|:422-1884
NuwaII-11_CPB	gb|AHGY02044136.1|:53847-55749
NuwaII-11_CPB	gb|AHGY02075778.1|:8632-12471
NuwaII-11_CPB	gb|AHGY02033783.1|:13161-17692
NuwaII-11_CPB	gb|AHGY02068321.1|:21659-25688
NuwaII-11_CPB	gb|AHGY02068321.1|:32503-33290
NuwaII-11_CPB	gb|AHGY02097236.1|:19491-23096
NuwaII-11_CPB	gb|AHGY02097236.1|:34632-38955
NuwaII-11_CPB	gb|AHGY02249384.1|:6992-13705
NuwaII-11_CPB	gb|AHGY02249384.1|:59388-59437
NuwaII-11_CPB	gb|AHGY02070882.1|:37046-38889
NuwaII-11_CPB	gb|AHGY02070882.1|:38954-39006
NuwaII-11_CPB	gb|AHGY02257284.1|:16043-19185
NuwaII-11_CPB	gb|AHGY02254629.1|:562-4252
NuwaII-11_CPB	gb|AHGY02245589.1|:10528-13409
NuwaII-11_CPB	gb|AHGY02225766.1|:8785-11581
NuwaII-11_CPB	gb|AHGY02225766.1|:13526-17866
NuwaII-11_CPB	gb|AHGY02250423.1|:30197-30624
NuwaII-11_CPB	gb|AHGY02250423.1|:15219-15282
NuwaII-11_CPB	gb|AHGY02250423.1|:1707-3253
NuwaII-11_CPB	gb|AHGY02243942.1|:6476-9736
NuwaII-11_CPB	gb|AHGY02234803.1|:1585-3441
NuwaII-11_CPB	gb|AHGY02082370.1|:29675-33466
NuwaII-11_CPB	gb|AHGY02000535.1|:1-1529
NuwaII-11_CPB	gb|AHGY02211217.1|:61739-65329
NuwaII-11_CPB	gb|AHGY02211217.1|:461-888
NuwaII-11_CPB	gb|AHGY02066456.1|:12595-15294
NuwaII-11_CPB	gb|AHGY02007098.1|:37267-40471
NuwaII-11_CPB	gb|AHGY02009897.1|:7538-9830
NuwaII-11_CPB	gb|AHGY02203754.1|:28860-31323
NuwaII-11_CPB	gb|AHGY02042039.1|:17023-20682
NuwaII-11_CPB	gb|AHGY02053489.1|:1887-5664
NuwaII-11_CPB	gb|AHGY02095782.1|:2144-5763

NuwaII-11_CPB	gb|AHGY02064886.1|:17108-20329
NuwaII-11_CPB	gb|AHGY02247175.1|:3451-4628
NuwaII-11_CPB	gb|AHGY02050473.1|:2481-4591
NuwaII-11_CPB	gb|AHGY02216901.1|:2720-6378
NuwaII-11_CPB	gb|AHGY02027045.1|:74963-78333
NuwaII-11_CPB	gb|AHGY02207584.1|:2069-5877
NuwaII-11_CPB	gb|AHGY02196391.1|:12786-16082
NuwaII-11_CPB	gb|AHGY02196711.1|:6557-8143
NuwaII-11_CPB	gb|AHGY02060777.1|:593-4376
NuwaII-11_CPB	gb|AHGY02230354.1|:924-3251
NuwaII-11_CPB	gb|AHGY02057975.1|:3197-6394
NuwaII-11_CPB	gb|AHGY02079870.1|:5163-8900
NuwaII-11_CPB	gb|AHGY02216423.1|:5444-9170
NuwaII-11_CPB	gb|AHGY02062902.1|:7554-10550
NuwaII-11_CPB	gb|AHGY02031939.1|:16327-19417
NuwaII-11_CPB	gb|AHGY02025855.1|:2469-5506
NuwaII-11_CPB	gb|AHGY02088790.1|:12366-16136
NuwaII-11_CPB	gb|AHGY02259964.1|:12223-12276
NuwaII-11_CPB	gb|AHGY02259964.1|:26635-30085
NuwaII-11_CPB	gb|AHGY02086839.1|:9403-12982
NuwaII-11_CPB	gb|AHGY02236836.1|:85234-88954
NuwaII-11_CPB	gb|AHGY02027264.1|:2696-7844
NuwaII-11_CPB	gb|AHGY02010753.1|:784-2139
NuwaII-11_CPB	gb|AHGY02067676.1|:19186-22268
NuwaII-11_CPB	gb|AHGY02031418.1|:377-2962
NuwaII-11_CPB	gb|AHGY02253315.1|:3879-5541
NuwaII-11_CPB	gb|AHGY02248557.1|:921-3927
NuwaII-11_CPB	gb|AHGY02008466.1|:20149-33044
NuwaII-11_CPB	gb|AHGY02196712.1|:1-998
NuwaII-11_CPB	gb|AHGY02240343.1|:58703-60198
NuwaII-11_CPB	gb|AHGY02233444.1|:10951-13991
NuwaII-11_CPB	gb|AHGY02029414.1|:8245-11268
NuwaII-11_CPB	gb|AHGY02207501.1|:39625-41107
NuwaII-11_CPB	gb|AHGY02239837.1|:14798-21350
NuwaII-11_CPB	gb|AHGY02256890.1|:12036-14728
NuwaII-11_CPB	gb|AHGY02089934.1|:17937-19282
NuwaII-11_CPB	gb|AHGY02041911.1|:36282-37693
NuwaII-11_CPB	gb|AHGY02022579.1|:14971-16066
NuwaII-11_CPB	gb|AHGY02026754.1|:1458-5258
NuwaII-11_CPB	gb|AHGY02254867.1|:712-4297
NuwaII-11_CPB	gb|AHGY02219656.1|:148-2035
NuwaII-11_CPB	gb|AHGY02074237.1|:1604-6738
NuwaII-11_CPB	gb|AHGY02056530.1|:741-3749
NuwaII-11_CPB	gb|AHGY02054140.1|:3293-5884

NuwaII-11_CPB	gb|AHGY02255569.1|:10160-10990
NuwaII-11_CPB	gb|AHGY02238884.1|:20681-20730
NuwaII-11_CPB	gb|AHGY02238884.1|:18970-22931
NuwaII-11_CPB	gb|AHGY02021964.1|:2730-6483
NuwaII-11_CPB	gb|AHGY02199289.1|:23088-26217
NuwaII-11_CPB	gb|AHGY02199289.1|:26661-26951
NuwaII-11_CPB	gb|AHGY02215582.1|:24853-27208
NuwaII-11_CPB	gb|AHGY02215582.1|:4471-5030
NuwaII-11_CPB	gb|AHGY02096171.1|:5048-9722
NuwaII-11_CPB	gb|AHGY02258028.1|:1338-3261
NuwaII-11_CPB	gb|AHGY02048617.1|:2319-6079
NuwaII-11_CPB	gb|AHGY02052905.1|:7790-11097
NuwaII-11_CPB	gb|AHGY02045856.1|:6749-6825
NuwaII-11_CPB	gb|AHGY02045856.1|:28458-28555
NuwaII-11_CPB	gb|AHGY02045856.1|:16959-20505
NuwaII-11_CPB	gb|AHGY02100577.1|:4121-7200
NuwaII-11_CPB	gb|AHGY02070797.1|:3322-5387
NuwaII-11_CPB	gb|AHGY02199054.1|:8612-13373
NuwaII-11_CPB	gb|AHGY02057184.1|:2703-4119
NuwaII-11_CPB	gb|AHGY02091542.1|:12467-14109
NuwaII-11_CPB	gb|AHGY02097397.1|:4241-8097
NuwaII-11_CPB	gb|AHGY02066595.1|:25108-29149
NuwaII-11_CPB	gb|AHGY02231370.1|:44698-47525
NuwaII-11_CPB	gb|AHGY02050997.1|:8644-9986
NuwaII-11_CPB	gb|AHGY02067912.1|:292-2418
NuwaII-11_CPB	gb|AHGY02078431.1|:3566-6413
NuwaII-11_CPB	gb|AHGY02232929.1|:11891-15816
NuwaII-11_CPB	gb|AHGY02229815.1|:4756-7672
NuwaII-11_CPB	gb|AHGY02075224.1|:19655-24654
NuwaII-11_CPB	gb|AHGY02044811.1|:32437-35432
NuwaII-11_CPB	gb|AHGY02044811.1|:80-772
NuwaII-11_CPB	gb|AHGY02215680.1|:8272-11793
NuwaII-11_CPB	gb|AHGY02198064.1|:4762-7816
NuwaII-11_CPB	gb|AHGY02120447.1|:2165-3214
NuwaII-11_CPB	gb|AHGY02079830.1|:116-1654
NuwaII-11_CPB	gb|AHGY02073744.1|:17745-22453
NuwaII-11_CPB	gb|AHGY02074560.1|:6219-9726
NuwaII-11_CPB	gb|AHGY02022853.1|:1693-4714
NuwaII-11_CPB	gb|AHGY02064022.1|:1119-2505
NuwaII-11_CPB	gb|AHGY02016844.1|:17640-18770
NuwaII-11_CPB	gb|AHGY02016844.1|:1810-2380
NuwaII-11_CPB	gb|AHGY02222608.1|:3167-5098
NuwaII-11_CPB	gb|AHGY02222608.1|:21107-27602
NuwaII-11_CPB	gb|AHGY02222608.1|:41081-41150

NuwaII-11_CPB	gb|AHGY02222608.1|:14591-14731
NuwaII-11_CPB	gb|AHGY02222608.1|:3922-3972
NuwaII-11_CPB	gb|AHGY02216350.1|:11282-12082
NuwaII-11_CPB	gb|AHGY02013484.1|:908-4724
NuwaII-11_CPB	gb|AHGY02241891.1|:5-1602
NuwaII-11_CPB	gb|AHGY02251079.1|:6063-9589
NuwaII-11_CPB	gb|AHGY02018554.1|:10316-13137
NuwaII-11_CPB	gb|AHGY02012747.1|:7367-10384
NuwaII-11_CPB	gb|AHGY02107275.1|:7-908
NuwaII-11_CPB	gb|AHGY02064444.1|:63-885
NuwaII-11_CPB	gb|AHGY02026947.1|:48032-50000
NuwaII-11_CPB	gb|AHGY02178812.1|:198-2780
NuwaII-11_CPB	gb|AHGY02219720.1|:34758-40036
NuwaII-11_CPB	gb|AHGY02202170.1|:8693-8888
NuwaII-11_CPB	gb|AHGY02202170.1|:25429-29308
NuwaII-11_CPB	gb|AHGY02115911.1|:2115-5137
NuwaII-11_CPB	gb|AHGY02204509.1|:1371-3889
NuwaII-11_CPB	gb|AHGY02204509.1|:6366-6828
NuwaII-11_CPB	gb|AHGY02244122.1|:47161-51594
NuwaII-11_CPB	gb|AHGY02064374.1|:29437-29847
NuwaII-11_CPB	gb|AHGY02064374.1|:2380-5206
NuwaII-11_CPB	gb|AHGY02038113.1|:61-1749
NuwaII-11_CPB	gb|AHGY02027833.1|:22559-25668
NuwaII-11_CPB	gb|AHGY02208944.1|:22097-25092
NuwaII-11_CPB	gb|AHGY02062480.1|:24282-24331
NuwaII-11_CPB	gb|AHGY02062480.1|:12825-15832
NuwaII-11_CPB	gb|AHGY02145516.1|:1-788
NuwaII-11_CPB	gb|AHGY02064278.1|:1608-3922
NuwaII-11_CPB	gb|AHGY02008094.1|:6270-8054
NuwaII-11_CPB	gb|AHGY02221106.1|:7632-10826
NuwaII-11_CPB	gb|AHGY02219475.1|:15165-18424
NuwaII-11_CPB	gb|AHGY02062637.1|:12869-16607
NuwaII-11_CPB	gb|AHGY02230609.1|:20125-23033
NuwaII-11_CPB	gb|AHGY02230609.1|:16852-16901
NuwaII-11_CPB	gb|AHGY02041980.1|:16964-21170
NuwaII-11_CPB	gb|AHGY02024409.1|:28616-32516
NuwaII-12_CPB	gb|AHGY02007031.1|:5571-5767
NuwaII-12_CPB	gb|AHGY02007031.1|:65929-70257
NuwaII-12_CPB	gb|AHGY02241824.1|:6891-10077
NuwaII-12_CPB	gb|AHGY02096668.1|:6048-9801
NuwaII-12_CPB	gb|AHGY02007197.1|:16208-20070
NuwaII-12_CPB	gb|AHGY02225552.1|:7434-9470
NuwaII-12_CPB	gb|AHGY02225552.1|:3592-3714
NuwaII-12_CPB	gb|AHGY02053395.1|:55566-55691

NuwaII-12_CPB	gb|AHGY02053395.1|:38452-42497
NuwaII-12_CPB	gb|AHGY02053395.1|:21585-21770
NuwaII-12_CPB	gb|AHGY02053395.1|:5076-5201
NuwaII-12_CPB	gb|AHGY02119153.1|:2-4028
NuwaII-12_CPB	gb|AHGY02223696.1|:10624-10709
NuwaII-12_CPB	gb|AHGY02223696.1|:27970-31913
NuwaII-12_CPB	gb|AHGY02083175.1|:1993-4448
NuwaII-12_CPB	gb|AHGY02054252.1|:5043-9065
NuwaII-12_CPB	gb|AHGY02054252.1|:30265-33076
NuwaII-12_CPB	gb|AHGY02054252.1|:244-347
NuwaII-12_CPB	gb|AHGY02045186.1|:24325-28700
NuwaII-12_CPB	gb|AHGY02045186.1|:2195-2354
NuwaII-12_CPB	gb|AHGY02203826.1|:13424-16997
NuwaII-12_CPB	gb|AHGY02033449.1|:96-5455
NuwaII-12_CPB	gb|AHGY02200446.1|:3038-5331
NuwaII-12_CPB	gb|AHGY02056672.1|:5540-9157
NuwaII-12_CPB	gb|AHGY02232492.1|:13694-15603
NuwaII-12_CPB	gb|AHGY02222893.1|:4739-8702
NuwaII-12_CPB	gb|AHGY02074895.1|:9725-12613
NuwaII-12_CPB	gb|AHGY02231274.1|:19817-19930
NuwaII-12_CPB	gb|AHGY02231274.1|:33933-34163
NuwaII-12_CPB	gb|AHGY02231274.1|:64914-68930
NuwaII-12_CPB	gb|AHGY02086852.1|:15212-20423
NuwaII-12_CPB	gb|AHGY02086852.1|:13102-13283
NuwaII-12_CPB	gb|AHGY02005601.1|:15772-15852
NuwaII-12_CPB	gb|AHGY02005601.1|:3686-7682
NuwaII-12_CPB	gb|AHGY02064802.1|:6027-10080
NuwaII-12_CPB	gb|AHGY02025816.1|:1427-3241
NuwaII-12_CPB	gb|AHGY02099788.1|:9820-11452
NuwaII-12_CPB	gb|AHGY02085922.1|:58951-62945
NuwaII-12_CPB	gb|AHGY02085922.1|:49370-49459
NuwaII-12_CPB	gb|AHGY02085922.1|:24220-24398
NuwaII-12_CPB	gb|AHGY02230049.1|:1795-4868
NuwaII-12_CPB	gb|AHGY02013116.1|:8287-12324
NuwaII-12_CPB	gb|AHGY02213512.1|:3350-7836
NuwaII-12_CPB	gb|AHGY02213512.1|:14974-16475
NuwaII-12_CPB	gb|AHGY02057232.1|:2551-6353
NuwaII-12_CPB	gb|AHGY02096245.1|:10279-13168
NuwaII-12_CPB	gb|AHGY02236341.1|:12308-18205
NuwaII-12_CPB	gb|AHGY02259599.1|:6988-12393
NuwaII-12_CPB	gb|AHGY02200683.1|:16287-19696
NuwaII-12_CPB	gb|AHGY02067538.1|:1312-3084
NuwaII-12_CPB	gb|AHGY02035605.1|:33469-36543
NuwaII-12_CPB	gb|AHGY02035605.1|:27745-27835

NuwaII-12_CPB	gb|AHGY02210501.1|:7094-7266
NuwaII-12_CPB	gb|AHGY02210501.1|:26893-27248
NuwaII-12_CPB	gb|AHGY02210501.1|:17371-21398
NuwaII-12_CPB	gb|AHGY02101596.1|:713-4598
NuwaII-12_CPB	gb|AHGY02073837.1|:10856-15952
NuwaII-12_CPB	gb|AHGY02005067.1|:6177-10443
NuwaII-12_CPB	gb|AHGY02253250.1|:1068-5465
NuwaII-12_CPB	gb|AHGY02253250.1|:194-5334
NuwaII-12_CPB	gb|AHGY02098052.1|:165-275
NuwaII-12_CPB	gb|AHGY02098052.1|:3194-5719
NuwaII-12_CPB	gb|AHGY02052293.1|:16175-16335
NuwaII-12_CPB	gb|AHGY02052293.1|:54022-54103
NuwaII-12_CPB	gb|AHGY02052293.1|:10916-12702
NuwaII-12_CPB	gb|AHGY02211420.1|:16654-19338
NuwaII-12_CPB	gb|AHGY02065883.1|:11235-15145
NuwaII-12_CPB	gb|AHGY02225427.1|:15748-15871
NuwaII-12_CPB	gb|AHGY02225427.1|:46564-50609
NuwaII-12_CPB	gb|AHGY02066490.1|:4595-9116
NuwaII-12_CPB	gb|AHGY02035673.1|:28698-28767
NuwaII-12_CPB	gb|AHGY02035673.1|:29080-31806
NuwaII-12_CPB	gb|AHGY02137678.1|:2-1307
NuwaII-12_CPB	gb|AHGY02227819.1|:8631-10238
NuwaII-12_CPB	gb|AHGY02082658.1|:5053-8048
NuwaII-12_CPB	gb|AHGY02205133.1|:96623-100559
NuwaII-12_CPB	gb|AHGY02205133.1|:45649-45771
NuwaII-12_CPB	gb|AHGY02205133.1|:13350-13529
NuwaII-12_CPB	gb|AHGY02022238.1|:41001-41087
NuwaII-12_CPB	gb|AHGY02022238.1|:21391-24386
NuwaII-12_CPB	gb|AHGY02196787.1|:61268-64912
NuwaII-12_CPB	gb|AHGY02196787.1|:54525-54709
NuwaII-12_CPB	gb|AHGY02060071.1|:2-1850
NuwaII-12_CPB	gb|AHGY02249752.1|:1136-5105
NuwaII-12_CPB	gb|AHGY02034650.1|:25560-29379
NuwaII-12_CPB	gb|AHGY02034650.1|:13370-13457
NuwaII-12_CPB	gb|AHGY02245266.1|:5465-6942
NuwaII-12_CPB	gb|AHGY02202255.1|:1661-4377
NuwaII-12_CPB	gb|AHGY02029258.1|:8076-12684
NuwaII-12_CPB	gb|AHGY02014859.1|:9565-9729
NuwaII-12_CPB	gb|AHGY02014859.1|:17368-22062
NuwaII-12_CPB	gb|AHGY02122876.1|:1-1813
NuwaII-12_CPB	gb|AHGY02052664.1|:865-4914
NuwaII-12_CPB	gb|AHGY02232122.1|:8219-8335
NuwaII-12_CPB	gb|AHGY02232122.1|:21582-24838
NuwaII-12_CPB	gb|AHGY02232122.1|:12815-13000

NuwaII-12_CPB	gb|AHGY02053032.1|:1-3861
NuwaII-12_CPB	gb|AHGY02043302.1|:1120-5118
NuwaII-12_CPB	gb|AHGY02084716.1|:4043-5700
NuwaII-12_CPB	gb|AHGY02028835.1|:3217-9424
NuwaII-12_CPB	gb|AHGY02237272.1|:19542-23557
NuwaII-12_CPB	gb|AHGY02234693.1|:4262-8285
NuwaII-12_CPB	gb|AHGY02210652.1|:10331-14350
NuwaII-12_CPB	gb|AHGY02026906.1|:13836-17883
NuwaII-12_CPB	gb|AHGY02260916.1|:10217-10630
NuwaII-12_CPB	gb|AHGY02260916.1|:21937-25906
NuwaII-12_CPB	gb|AHGY02240167.1|:4510-11026
NuwaII-12_CPB	gb|AHGY02216176.1|:53419-55944
NuwaII-12_CPB	gb|AHGY02089593.1|:4135-8638
NuwaII-12_CPB	gb|AHGY02042420.1|:3-1634
NuwaII-12_CPB	gb|AHGY02039498.1|:98180-98306
NuwaII-12_CPB	gb|AHGY02039498.1|:38774-42048
NuwaII-12_CPB	gb|AHGY02011066.1|:19158-25129
NuwaII-12_CPB	gb|AHGY02244482.1|:244-1475
NuwaII-12_CPB	gb|AHGY02200425.1|:16619-17830
NuwaII-12_CPB	gb|AHGY02200425.1|:4507-4882
NuwaII-12_CPB	gb|AHGY02240842.1|:303-393
NuwaII-12_CPB	gb|AHGY02240842.1|:14527-14714
NuwaII-12_CPB	gb|AHGY02240842.1|:12158-16333
NuwaII-12_CPB	gb|AHGY02235924.1|:12086-15260
NuwaII-12_CPB	gb|AHGY02105293.1|:12793-16813
NuwaII-12_CPB	gb|AHGY02066968.1|:10167-14132
NuwaII-12_CPB	gb|AHGY02043016.1|:371-2020
NuwaII-12_CPB	gb|AHGY02025150.1|:6042-9996
NuwaII-12_CPB	gb|AHGY02221258.1|:51-2111
NuwaII-12_CPB	gb|AHGY02075384.1|:10535-13409
NuwaII-12_CPB	gb|AHGY02029685.1|:1-1890
NuwaII-12_CPB	gb|AHGY02020089.1|:42237-42363
NuwaII-12_CPB	gb|AHGY02020089.1|:28149-32105
NuwaII-12_CPB	gb|AHGY02107105.1|:19937-20121
NuwaII-12_CPB	gb|AHGY02107105.1|:11131-15089
NuwaII-12_CPB	gb|AHGY02253673.1|:1559-4926
NuwaII-12_CPB	gb|AHGY02254108.1|:15179-15360
NuwaII-12_CPB	gb|AHGY02254108.1|:1081-2482
NuwaII-12_CPB	gb|AHGY02245389.1|:450-3094
NuwaII-12_CPB	gb|AHGY02093758.1|:2837-4631
NuwaII-12_CPB	gb|AHGY02071629.1|:13198-24430
NuwaII-12_CPB	gb|AHGY02071629.1|:22865-23020
NuwaII-12_CPB	gb|AHGY02027716.1|:34586-37485
NuwaII-12_CPB	gb|AHGY02082284.1|:13939-17923

NuwaII-12_CPB	gb|AHGY02239371.1|:57950-62464
NuwaII-12_CPB	gb|AHGY02239371.1|:21032-21156
NuwaII-12_CPB	gb|AHGY02239371.1|:423-1083
NuwaII-12_CPB	gb|AHGY02248044.1|:17-2769
NuwaII-12_CPB	gb|AHGY02063434.1|:2447-5229
NuwaII-12_CPB	gb|AHGY02257570.1|:21411-24302
NuwaII-12_CPB	gb|AHGY02257570.1|:914-2683
NuwaII-12_CPB	gb|AHGY02253738.1|:8690-12728
NuwaII-12_CPB	gb|AHGY02107082.1|:15422-15615
NuwaII-12_CPB	gb|AHGY02107082.1|:31567-33930
NuwaII-12_CPB	gb|AHGY02107082.1|:29933-30055
NuwaII-12_CPB	gb|AHGY02216401.1|:8110-12147
NuwaII-12_CPB	gb|AHGY02046225.1|:6310-10579
NuwaII-12_CPB	gb|AHGY02046225.1|:33161-35029
NuwaII-12_CPB	gb|AHGY02046225.1|:19282-19351
NuwaII-12_CPB	gb|AHGY02245982.1|:17913-22823
NuwaII-12_CPB	gb|AHGY02099091.1|:9426-9514
NuwaII-12_CPB	gb|AHGY02099091.1|:23818-23908
NuwaII-12_CPB	gb|AHGY02099091.1|:21681-21749
NuwaII-12_CPB	gb|AHGY02099091.1|:123-5821
NuwaII-12_CPB	gb|AHGY02213537.1|:6020-10043
NuwaII-12_CPB	gb|AHGY02200416.1|:14179-17963
NuwaII-12_CPB	gb|AHGY02200416.1|:3771-3946
NuwaII-12_CPB	gb|AHGY02059839.1|:5801-8707
NuwaII-12_CPB	gb|AHGY02131914.1|:213-1399
NuwaII-12_CPB	gb|AHGY02254825.1|:39106-39231
NuwaII-12_CPB	gb|AHGY02254825.1|:36238-39283
NuwaII-12_CPB	gb|AHGY02224210.1|:18665-22649
NuwaII-12_CPB	gb|AHGY02120179.1|:13-1323
NuwaII-12_CPB	gb|AHGY02075847.1|:25182-27130
NuwaII-12_CPB	gb|AHGY02030678.1|:5618-9601
NuwaII-12_CPB	gb|AHGY02210850.1|:1792-5887
NuwaII-12_CPB	gb|AHGY02210850.1|:15208-15328
NuwaII-12_CPB	gb|AHGY02047911.1|:609-3979
NuwaII-12_CPB	gb|AHGY02047911.1|:26026-26209
NuwaII-12_CPB	gb|AHGY02042168.1|:76167-80199
NuwaII-12_CPB	gb|AHGY02042168.1|:21189-21268
NuwaII-12_CPB	gb|AHGY02072566.1|:9950-14144
NuwaII-12_CPB	gb|AHGY02103694.1|:32335-35892
NuwaII-12_CPB	gb|AHGY02103694.1|:41567-41732
NuwaII-12_CPB	gb|AHGY02056027.1|:14587-16606
NuwaII-12_CPB	gb|AHGY02043786.1|:4420-9040
NuwaII-12_CPB	gb|AHGY02201326.1|:37345-37522
NuwaII-12_CPB	gb|AHGY02201326.1|:18682-22635

NuwaII-12_CPB	gb|AHGY02035543.1|:3251-6119
NuwaII-12_CPB	gb|AHGY02031199.1|:40658-42346
NuwaII-12_CPB	gb|AHGY02216258.1|:40989-42171
NuwaII-12_CPB	gb|AHGY02196508.1|:12242-16254
NuwaII-12_CPB	gb|AHGY02076544.1|:1097-1217
NuwaII-12_CPB	gb|AHGY02076544.1|:15843-16029
NuwaII-12_CPB	gb|AHGY02076544.1|:54882-55020
NuwaII-12_CPB	gb|AHGY02076544.1|:68829-81224
NuwaII-12_CPB	gb|AHGY02076544.1|:48916-49100
NuwaII-12_CPB	gb|AHGY02076544.1|:23833-23906
NuwaII-12_CPB	gb|AHGY02012352.1|:15240-19415
NuwaII-12_CPB	gb|AHGY02024499.1|:18424-22721
NuwaII-12_CPB	gb|AHGY02024499.1|:16705-16886
NuwaII-12_CPB	gb|AHGY02255481.1|:467-3381
NuwaII-12_CPB	gb|AHGY02222891.1|:29726-33753
NuwaII-12_CPB	gb|AHGY02069650.1|:2383-6533
NuwaII-12_CPB	gb|AHGY02244061.1|:11868-11990
NuwaII-12_CPB	gb|AHGY02244061.1|:50228-52961
NuwaII-12_CPB	gb|AHGY02244061.1|:35629-35734
NuwaII-12_CPB	gb|AHGY02260644.1|:35004-38659
NuwaII-12_CPB	gb|AHGY02260644.1|:38715-40158
NuwaII-12_CPB	gb|AHGY02260644.1|:343-1759
NuwaII-12_CPB	gb|AHGY02039526.1|:103931-104053
NuwaII-12_CPB	gb|AHGY02039526.1|:1063-3800
NuwaII-12_CPB	gb|AHGY02250086.1|:349-4174
NuwaII-12_CPB	gb|AHGY02039614.1|:5357-5474
NuwaII-12_CPB	gb|AHGY02039614.1|:14759-20963
NuwaII-12_CPB	gb|AHGY02011937.1|:6900-11225
NuwaII-12_CPB	gb|AHGY02206634.1|:10731-12818
NuwaII-12_CPB	gb|AHGY02103692.1|:8127-12081
NuwaII-12_CPB	gb|AHGY02082260.1|:26942-27123
NuwaII-12_CPB	gb|AHGY02082260.1|:46097-50046
NuwaII-12_CPB	gb|AHGY02058667.1|:2-1237
NuwaII-12_CPB	gb|AHGY02031056.1|:9376-12689
NuwaII-12_CPB	gb|AHGY02217454.1|:37202-41255
NuwaII-12_CPB	gb|AHGY02206752.1|:19195-19274
NuwaII-12_CPB	gb|AHGY02206752.1|:3390-7384
NuwaII-12_CPB	gb|AHGY02199522.1|:6354-10378
NuwaII-12_CPB	gb|AHGY02005538.1|:906-4843
NuwaII-12_CPB	gb|AHGY02153744.1|:4318-4440
NuwaII-12_CPB	gb|AHGY02153744.1|:1528-4688
NuwaII-12_CPB	gb|AHGY02082464.1|:2962-7100
NuwaII-12_CPB	gb|AHGY02089328.1|:13117-13241
NuwaII-12_CPB	gb|AHGY02089328.1|:6554-9504

NuwaII-12_CPB	gb|AHGY02259928.1|:4009-6770
NuwaII-12_CPB	gb|AHGY02214432.1|:14304-18279
NuwaII-12_CPB	gb|AHGY02214432.1|:954-1168
NuwaII-12_CPB	gb|AHGY02101865.1|:2637-6227
NuwaII-12_CPB	gb|AHGY02136448.1|:2-1369
NuwaII-12_CPB	gb|AHGY02089464.1|:3390-6336
NuwaII-12_CPB	gb|AHGY02053288.1|:8146-8332
NuwaII-12_CPB	gb|AHGY02053288.1|:14536-18423
NuwaII-12_CPB	gb|AHGY02072652.1|:9321-12663
NuwaII-12_CPB	gb|AHGY02032397.1|:12253-16214
NuwaII-12_CPB	gb|AHGY02229555.1|:727-4651
NuwaII-12_CPB	gb|AHGY02202728.1|:53-4738
NuwaII-12_CPB	gb|AHGY02011529.1|:8761-9967
NuwaII-12_CPB	gb|AHGY02048952.1|:6696-10640
NuwaII-12_CPB	gb|AHGY02042581.1|:6638-10620
NuwaII-12_CPB	gb|AHGY02005202.1|:6605-9603
NuwaII-12_CPB	gb|AHGY02066975.1|:4311-7880
NuwaII-12_CPB	gb|AHGY02066975.1|:12791-12956
NuwaII-12_CPB	gb|AHGY02027110.1|:34936-35118
NuwaII-12_CPB	gb|AHGY02027110.1|:64770-64891
NuwaII-12_CPB	gb|AHGY02027110.1|:56705-60938
NuwaII-12_CPB	gb|AHGY02088534.1|:4667-6501
NuwaII-12_CPB	gb|AHGY02239332.1|:5333-9860
NuwaII-12_CPB	gb|AHGY02257561.1|:324-4356
NuwaII-12_CPB	gb|AHGY02067259.1|:8910-9722
NuwaII-12_CPB	gb|AHGY02067259.1|:30233-31541
NuwaII-12_CPB	gb|AHGY02224788.1|:2620-5022
NuwaII-12_CPB	gb|AHGY02208356.1|:259-4034
NuwaII-12_CPB	gb|AHGY02208356.1|:12074-12194
NuwaII-12_CPB	gb|AHGY02131387.1|:380-1822
NuwaII-12_CPB	gb|AHGY02091462.1|:7260-9668
NuwaII-12_CPB	gb|AHGY02072330.1|:1165-5575
NuwaII-12_CPB	gb|AHGY02022188.1|:9034-9207
NuwaII-12_CPB	gb|AHGY02022188.1|:44942-48500
NuwaII-12_CPB	gb|AHGY02022188.1|:51425-51610
NuwaII-12_CPB	gb|AHGY02230687.1|:30507-30692
NuwaII-12_CPB	gb|AHGY02230687.1|:38039-38365
NuwaII-12_CPB	gb|AHGY02230687.1|:23952-26354
NuwaII-12_CPB	gb|AHGY02205458.1|:1-1431
NuwaII-12_CPB	gb|AHGY02097201.1|:2896-7352
NuwaII-12_CPB	gb|AHGY02064335.1|:56536-56659
NuwaII-12_CPB	gb|AHGY02064335.1|:26996-28305
NuwaII-12_CPB	gb|AHGY02042577.1|:86-2763
NuwaII-12_CPB	gb|AHGY02105926.1|:608-8601

NuwaII-12_CPB	gb|AHGY02210476.1|:85296-90116
NuwaII-12_CPB	gb|AHGY02210476.1|:53929-54049
NuwaII-12_CPB	gb|AHGY02199625.1|:42082-45898
NuwaII-12_CPB	gb|AHGY02199625.1|:39326-39415
NuwaII-12_CPB	gb|AHGY02199625.1|:15607-15682
NuwaII-12_CPB	gb|AHGY02043157.1|:3616-7888
NuwaII-12_CPB	gb|AHGY02043157.1|:1614-1736
NuwaII-12_CPB	gb|AHGY02081969.1|:19139-19452
NuwaII-12_CPB	gb|AHGY02081969.1|:7953-12367
NuwaII-12_CPB	gb|AHGY02023202.1|:1209-5016
NuwaII-12_CPB	gb|AHGY02239429.1|:9801-13742
NuwaII-12_CPB	gb|AHGY02001065.1|:9722-13726
NuwaII-12_CPB	gb|AHGY02259144.1|:1450-5441
NuwaII-12_CPB	gb|AHGY02111240.1|:7250-8594
NuwaII-12_CPB	gb|AHGY02062926.1|:4195-5534
NuwaII-12_CPB	gb|AHGY02227870.1|:73556-76206
NuwaII-12_CPB	gb|AHGY02227870.1|:59590-59713
NuwaII-12_CPB	gb|AHGY02213850.1|:38001-42014
NuwaII-12_CPB	gb|AHGY02213850.1|:1334-1457
NuwaII-12_CPB	gb|AHGY02055356.1|:2000-5537
NuwaII-12_CPB	gb|AHGY02055356.1|:9525-14809
NuwaII-12_CPB	gb|AHGY02038181.1|:38467-40725
NuwaII-12_CPB	gb|AHGY02038181.1|:11047-11225
NuwaII-12_CPB	gb|AHGY02239732.1|:4185-8137
NuwaII-12_CPB	gb|AHGY02180728.1|:936-2737
NuwaII-12_CPB	gb|AHGY02008415.1|:287-3144
NuwaII-12_CPB	gb|AHGY02195231.1|:780-1946
NuwaII-12_CPB	gb|AHGY02207499.1|:2230-6992
NuwaII-12_CPB	gb|AHGY02258148.1|:1-3437
NuwaII-12_CPB	gb|AHGY02230525.1|:16273-16348
NuwaII-12_CPB	gb|AHGY02230525.1|:27669-27818
NuwaII-12_CPB	gb|AHGY02230525.1|:19524-23735
NuwaII-12_CPB	gb|AHGY02230525.1|:4182-4371
NuwaII-12_CPB	gb|AHGY02116929.1|:1226-2454
NuwaII-12_CPB	gb|AHGY02012449.1|:23828-27548
NuwaII-12_CPB	gb|AHGY02012449.1|:2731-8172
NuwaII-12_CPB	gb|AHGY02199052.1|:14172-14358
NuwaII-12_CPB	gb|AHGY02199052.1|:26685-28161
NuwaII-12_CPB	gb|AHGY02239787.1|:30883-34946
NuwaII-12_CPB	gb|AHGY02231004.1|:3909-7904
NuwaII-12_CPB	gb|AHGY02207906.1|:11868-18072
NuwaII-12_CPB	gb|AHGY02058668.1|:1-1177
NuwaII-12_CPB	gb|AHGY02238090.1|:1-3035
NuwaII-12_CPB	gb|AHGY02261824.1|:40020-43435

NuwaII-12_CPB	gb|AHGY02208421.1|:4658-8667
NuwaII-12_CPB	gb|AHGY02208421.1|:6219-7731
NuwaII-12_CPB	gb|AHGY02070753.1|:1515-5686
NuwaII-12_CPB	gb|AHGY02231585.1|:11097-12308
NuwaII-12_CPB	gb|AHGY02231585.1|:24160-24243
NuwaII-12_CPB	gb|AHGY02257209.1|:1816-5194
NuwaII-12_CPB	gb|AHGY02219476.1|:18086-22092
NuwaII-12_CPB	gb|AHGY02029016.1|:54829-55019
NuwaII-12_CPB	gb|AHGY02029016.1|:13784-17790
NuwaII-12_CPB	gb|AHGY02207466.1|:262-1552
NuwaII-12_CPB	gb|AHGY02214622.1|:2329-6245
NuwaII-12_CPB	gb|AHGY02013589.1|:17433-19471
NuwaII-12_CPB	gb|AHGY02005461.1|:299-4130
NuwaII-12_CPB	gb|AHGY02035886.1|:20737-24557
NuwaII-12_CPB	gb|AHGY02034783.1|:567-4721
NuwaII-12_CPB	gb|AHGY02067996.1|:422-1673
NuwaII-12_CPB	gb|AHGY02213836.1|:26823-30693
NuwaII-12_CPB	gb|AHGY02038843.1|:7859-7977
NuwaII-12_CPB	gb|AHGY02038843.1|:23372-24506
NuwaII-12_CPB	gb|AHGY02246685.1|:10400-12128
NuwaII-12_CPB	gb|AHGY02246685.1|:4357-4537
NuwaII-12_CPB	gb|AHGY02003682.1|:4798-8703
NuwaII-12_CPB	gb|AHGY02008206.1|:2306-3385
NuwaII-12_CPB	gb|AHGY02258266.1|:3661-3784
NuwaII-12_CPB	gb|AHGY02258266.1|:7851-13454
NuwaII-12_CPB	gb|AHGY02067260.1|:834-2140
NuwaII-12_CPB	gb|AHGY02050459.1|:24505-29880
NuwaII-12_CPB	gb|AHGY02050459.1|:22322-22412
NuwaII-12_CPB	gb|AHGY02013715.1|:2843-5729
NuwaII-12_CPB	gb|AHGY02201296.1|:32009-36229
NuwaII-12_CPB	gb|AHGY02261070.1|:49414-51646
NuwaII-12_CPB	gb|AHGY02261070.1|:31138-31203
NuwaII-12_CPB	gb|AHGY02261070.1|:9557-16725
NuwaII-12_CPB	gb|AHGY02089407.1|:11377-14476
NuwaII-12_CPB	gb|AHGY02225738.1|:12181-16101
NuwaII-12_CPB	gb|AHGY02007322.1|:165-1351
NuwaII-12_CPB	gb|AHGY02050541.1|:35311-40294
NuwaII-12_CPB	gb|AHGY02050541.1|:1231-1416
NuwaII-12_CPB	gb|AHGY02234045.1|:679-769
NuwaII-12_CPB	gb|AHGY02234045.1|:2896-9491
NuwaII-12_CPB	gb|AHGY02098636.1|:596-665
NuwaII-12_CPB	gb|AHGY02098636.1|:16084-16194
NuwaII-12_CPB	gb|AHGY02098636.1|:741-4796
NuwaII-12_CPB	gb|AHGY02206463.1|:31944-32034

NuwaII-12_CPB	gb|AHGY02206463.1|:56362-60391
NuwaII-12_CPB	gb|AHGY02050909.1|:4145-7520
NuwaII-12_CPB	gb|AHGY02254100.1|:4358-14623
NuwaII-12_CPB	gb|AHGY02198239.1|:1362-3843
NuwaII-12_CPB	gb|AHGY02077320.1|:665-3833
NuwaII-13_CPB	gb|AHGY02099169.1|:31304-34105
NuwaII-13_CPB	gb|AHGY02099169.1|:80187-80264
NuwaII-13_CPB	gb|AHGY02221848.1|:2254-6182
NuwaII-13_CPB	gb|AHGY02221848.1|:60-619
NuwaII-13_CPB	gb|AHGY02021725.1|:16521-20688
NuwaII-13_CPB	gb|AHGY02260471.1|:197-3682
NuwaII-13_CPB	gb|AHGY02224423.1|:12214-14310
NuwaII-13_CPB	gb|AHGY02244437.1|:12969-13057
NuwaII-13_CPB	gb|AHGY02244437.1|:683-2515
NuwaII-13_CPB	gb|AHGY02256739.1|:5610-9282
NuwaII-13_CPB	gb|AHGY02222699.1|:36984-40573
NuwaII-13_CPB	gb|AHGY02222699.1|:58462-62278
NuwaII-13_CPB	gb|AHGY02209788.1|:10911-14716
NuwaII-13_CPB	gb|AHGY02019070.1|:19049-23262
NuwaII-13_CPB	gb|AHGY02196203.1|:401-2455
NuwaII-13_CPB	gb|AHGY02225623.1|:10423-11198
NuwaII-13_CPB	gb|AHGY02225623.1|:52413-56469
NuwaII-13_CPB	gb|AHGY02053277.1|:3495-7166
NuwaII-13_CPB	gb|AHGY02060980.1|:14684-17766
NuwaII-13_CPB	gb|AHGY02060980.1|:54175-58387
NuwaII-13_CPB	gb|AHGY02061497.1|:3961-8140
NuwaII-13_CPB	gb|AHGY02207732.1|:291-2739
NuwaII-13_CPB	gb|AHGY02207732.1|:2963-3019
NuwaII-13_CPB	gb|AHGY02025362.1|:27665-29101
NuwaII-13_CPB	gb|AHGY02229605.1|:1980-6468
NuwaII-13_CPB	gb|AHGY02212684.1|:41037-46257
NuwaII-13_CPB	gb|AHGY02260503.1|:9898-12000
NuwaII-13_CPB	gb|AHGY02234822.1|:40390-41824
NuwaII-13_CPB	gb|AHGY02233711.1|:4528-8672
NuwaII-13_CPB	gb|AHGY02225684.1|:34307-38491
NuwaII-13_CPB	gb|AHGY02089132.1|:2451-5435
NuwaII-13_CPB	gb|AHGY02203189.1|:9230-13301
NuwaII-13_CPB	gb|AHGY02036953.1|:822-4946
NuwaII-13_CPB	gb|AHGY02247499.1|:45748-49116
NuwaII-13_CPB	gb|AHGY02247499.1|:69835-70609
NuwaII-13_CPB	gb|AHGY02247499.1|:81339-82124
NuwaII-13_CPB	gb|AHGY02247499.1|:98508-98586
NuwaII-13_CPB	gb|AHGY02205086.1|:17780-21985
NuwaII-13_CPB	gb|AHGY02033754.1|:28002-32203

NuwaII-13_CPB	gb|AHGY02201718.1|:2583-6789
NuwaII-13_CPB	gb|AHGY02252995.1|:47367-50215
NuwaII-13_CPB	gb|AHGY02245600.1|:6037-11442
NuwaII-13_CPB	gb|AHGY02064909.1|:694-2430
NuwaII-13_CPB	gb|AHGY02205706.1|:7647-11984
NuwaII-13_CPB	gb|AHGY02205528.1|:16963-21352
NuwaII-13_CPB	gb|AHGY02004180.1|:41941-44897
NuwaII-13_CPB	gb|AHGY02230421.1|:29443-29552
NuwaII-13_CPB	gb|AHGY02230421.1|:32398-37285
NuwaII-13_CPB	gb|AHGY02203252.1|:2694-6701
NuwaII-13_CPB	gb|AHGY02072548.1|:14688-16832
NuwaII-13_CPB	gb|AHGY02072548.1|:25552-25617
NuwaII-13_CPB	gb|AHGY02072548.1|:10779-14018
NuwaII-13_CPB	gb|AHGY02205190.1|:9871-13961
NuwaII-13_CPB	gb|AHGY02064908.1|:511-2797
NuwaII-13_CPB	gb|AHGY02009676.1|:2637-5465
NuwaII-13_CPB	gb|AHGY02080973.1|:5431-9558
NuwaII-13_CPB	gb|AHGY02050197.1|:2-4847
NuwaII-13_CPB	gb|AHGY02202603.1|:4811-6772
NuwaII-13_CPB	gb|AHGY02069927.1|:3535-6653
NuwaII-13_CPB	gb|AHGY02161063.1|:1-2608
NuwaII-13_CPB	gb|AHGY02009677.1|:1-1376
NuwaII-13_CPB	gb|AHGY02041305.1|:20893-24940
NuwaII-13_CPB	gb|AHGY02043710.1|:17753-20304
NuwaII-13_CPB	gb|AHGY02237003.1|:22582-26774
NuwaII-13_CPB	gb|AHGY02261489.1|:10781-13949
NuwaII-13_CPB	gb|AHGY02230248.1|:5463-11043
NuwaII-13_CPB	gb|AHGY02036291.1|:1-268
NuwaII-13_CPB	gb|AHGY02036291.1|:579-3982
NuwaII-13_CPB	gb|AHGY02036031.1|:24105-27536
NuwaII-13_CPB	gb|AHGY02036031.1|:3948-4741
NuwaII-13_CPB	gb|AHGY02241241.1|:6150-11403
NuwaII-13_CPB	gb|AHGY02074656.1|:4708-9071
NuwaII-13_CPB	gb|AHGY02200354.1|:6116-11067
NuwaII-13_CPB	gb|AHGY02046986.1|:54872-57767
NuwaII-13_CPB	gb|AHGY02032072.1|:30-827
NuwaII-13_CPB	gb|AHGY02032072.1|:7653-11088
NuwaII-13_CPB	gb|AHGY02011094.1|:5576-6476
NuwaII-13_CPB	gb|AHGY02211182.1|:19115-24333
NuwaII-13_CPB	gb|AHGY02256695.1|:10152-14935
NuwaII-13_CPB	gb|AHGY02065416.1|:14680-14782
NuwaII-13_CPB	gb|AHGY02065416.1|:9836-14372
NuwaII-13_CPB	gb|AHGY02210286.1|:47765-54313
NuwaII-13_CPB	gb|AHGY02196523.1|:35303-36669

NuwaII-13_CPB	gb|AHGY02258114.1|:55944-60001
NuwaII-13_CPB	gb|AHGY02258114.1|:1685-1905
NuwaII-13_CPB	gb|AHGY02051087.1|:1649-3343
NuwaII-13_CPB	gb|AHGY02035222.1|:21205-25861
NuwaII-13_CPB	gb|AHGY02031885.1|:12016-16344
NuwaII-13_CPB	gb|AHGY02031885.1|:189-260
NuwaII-13_CPB	gb|AHGY02001706.1|:5265-7897
NuwaII-13_CPB	gb|AHGY02237625.1|:5751-9426
NuwaII-13_CPB	gb|AHGY02022476.1|:9811-9915
NuwaII-13_CPB	gb|AHGY02022476.1|:46015-46648
NuwaII-13_CPB	gb|AHGY02022476.1|:26272-30378
NuwaII-13_CPB	gb|AHGY02066364.1|:4961-6240
NuwaII-13_CPB	gb|AHGY02198436.1|:10119-14281
NuwaII-13_CPB	gb|AHGY02080238.1|:38629-40406
NuwaII-13_CPB	gb|AHGY02026602.1|:1-906
NuwaII-13_CPB	gb|AHGY02001164.1|:21668-21925
NuwaII-13_CPB	gb|AHGY02001164.1|:12098-14495
NuwaII-13_CPB	gb|AHGY02026603.1|:2-906
NuwaII-13_CPB	gb|AHGY02038291.1|:869-3237
NuwaII-13_CPB	gb|AHGY02009126.1|:7019-10979
NuwaII-13_CPB	gb|AHGY02046360.1|:4781-5867
NuwaII-13_CPB	gb|AHGY02104049.1|:248-3185
NuwaII-13_CPB	gb|AHGY02048068.1|:15927-19130
NuwaII-13_CPB	gb|AHGY02001097.1|:17-88
NuwaII-13_CPB	gb|AHGY02001097.1|:208-5712
NuwaII-13_CPB	gb|AHGY02233366.1|:29215-32633
NuwaII-13_CPB	gb|AHGY02044595.1|:62591-63945
NuwaII-13_CPB	gb|AHGY02032097.1|:3343-7507
NuwaII-13_CPB	gb|AHGY02058544.1|:2753-4430
NuwaII-13_CPB	gb|AHGY02030045.1|:10751-13485
NuwaII-13_CPB	gb|AHGY02196202.1|:4879-7341
NuwaII-13_CPB	gb|AHGY02245060.1|:18466-20469
NuwaII-13_CPB	gb|AHGY02245060.1|:19860-19953
NuwaII-13_CPB	gb|AHGY02046361.1|:116-3199
NuwaII-13_CPB	gb|AHGY02011918.1|:28248-32431
NuwaII-13_CPB	gb|AHGY02049749.1|:2328-3556
NuwaII-13_CPB	gb|AHGY02244324.1|:32218-33077
NuwaII-13_CPB	gb|AHGY02219856.1|:284-3684
NuwaII-13_CPB	gb|AHGY02054914.1|:12051-16211
NuwaII-13_CPB	gb|AHGY02228287.1|:8157-9847
NuwaII-13_CPB	gb|AHGY02217243.1|:194-776
NuwaII-13_CPB	gb|AHGY02217243.1|:24438-28499
NuwaII-13_CPB	gb|AHGY02247486.1|:3653-6616
NuwaII-13_CPB	gb|AHGY02056818.1|:33-2538

NuwaII-13_CPB	gb|AHGY02022770.1|:5567-10152
NuwaII-13_CPB	gb|AHGY02075891.1|:11904-15444
NuwaII-13_CPB	gb|AHGY02196038.1|:15850-19271
NuwaII-13_CPB	gb|AHGY02260472.1|:743-1413
NuwaII-13_CPB	gb|AHGY02130349.1|:500-1489
NuwaII-13_CPB	gb|AHGY02040878.1|:6638-8001
NuwaII-13_CPB	gb|AHGY02251335.1|:314-4425
NuwaII-13_CPB	gb|AHGY02056817.1|:15877-17792
NuwaII-13_CPB	gb|AHGY02244820.1|:14059-15005
NuwaII-13_CPB	gb|AHGY02251659.1|:3261-4049
NuwaII-13_CPB	gb|AHGY02196073.1|:1826-3444
NuwaII-13_CPB	gb|AHGY02026416.1|:6134-7052
NuwaII-13_CPB	gb|AHGY02080713.1|:25-3207
NuwaII-13_CPB	gb|AHGY02209789.1|:8-1686
NuwaII-13_CPB	gb|AHGY02156834.1|:135-705
NuwaII-13_CPB	gb|AHGY02248046.1|:4159-5584
NuwaII-13_CPB	gb|AHGY02169370.1|:2-619
NuwaII-13_CPB	gb|AHGY02034290.1|:29625-31222
NuwaII-13_CPB	gb|AHGY02036369.1|:2190-6229
NuwaII-13_CPB	gb|AHGY02076887.1|:27718-29447
NuwaII-13_CPB	gb|AHGY02201705.1|:70-10372
NuwaII-13_CPB	gb|AHGY02059159.1|:12274-13488
NuwaII-13_CPB	gb|AHGY02207731.1|:11921-13498
NuwaII-13_CPB	gb|AHGY02222001.1|:3471-7314
NuwaII-13_CPB	gb|AHGY02044596.1|:2237-5037
NuwaII-13_CPB	gb|AHGY02262198.1|:26091-29216
NuwaII-13_CPB	gb|AHGY02041033.1|:13007-16070
NuwaII-13_CPB	gb|AHGY02065986.1|:71-1605
NuwaII-13_CPB	gb|AHGY02233367.1|:551-1095
NuwaII-13_CPB	gb|AHGY02074645.1|:2595-4977
NuwaII-13_CPB	gb|AHGY02222002.1|:747-1630
NuwaII-13_CPB	gb|AHGY02230254.1|:21065-24555
NuwaII-13_CPB	gb|AHGY02016287.1|:17787-20730
NuwaII-13_CPB	gb|AHGY02044093.1|:29512-33128
NuwaII-13_CPB	gb|AHGY02085403.1|:6591-7557
NuwaII-13_CPB	gb|AHGY02085403.1|:9301-10085
NuwaII-13_CPB	gb|AHGY02035608.1|:4605-6047
NuwaII-13_CPB	gb|AHGY02053278.1|:1-467
NuwaII-13_CPB	gb|AHGY02036290.1|:434-614
NuwaII-13_CPB	gb|AHGY02036290.1|:23439-24724
NuwaII-13_CPB	gb|AHGY02260955.1|:6571-10040
NuwaII-13_CPB	gb|AHGY02234063.1|:22138-25446
NuwaII-13_CPB	gb|AHGY02033491.1|:12030-15549
NuwaII-13_CPB	gb|AHGY02224280.1|:14865-18645

NuwaII-13_CPB	gb|AHGY02209245.1|:4896-6615
NuwaII-13_CPB	gb|AHGY02073732.1|:19150-23135
NuwaII-13_CPB	gb|AHGY02243903.1|:5173-5518
NuwaII-13_CPB	gb|AHGY02243903.1|:388-1402
NuwaII-13_CPB	gb|AHGY02046947.1|:2075-4864
NuwaII-13_CPB	gb|AHGY02238823.1|:23061-24957
NuwaII-13_CPB	gb|AHGY02082172.1|:52-1084
NuwaII-13_CPB	gb|AHGY02030433.1|:34550-36511
NuwaII-13_CPB	gb|AHGY02075408.1|:6099-10185
NuwaII-13_CPB	gb|AHGY02080068.1|:30575-34777
NuwaII-13_CPB	gb|AHGY02038041.1|:32-1394
NuwaII-13_CPB	gb|AHGY02051088.1|:21-1683
NuwaII-13_CPB	gb|AHGY02062510.1|:59967-61663
NuwaII-13_CPB	gb|AHGY02157714.1|:1974-5549
NuwaII-13_CPB	gb|AHGY02063268.1|:15201-18132
NuwaII-13_CPB	gb|AHGY02057885.1|:4393-8695
NuwaII-13_CPB	gb|AHGY02057885.1|:12705-16528
NuwaII-13_CPB	gb|AHGY02260231.1|:12047-15527
NuwaII-13_CPB	gb|AHGY02099243.1|:213-4460
NuwaII-13_CPB	gb|AHGY02038268.1|:5618-9978
NuwaII-13_CPB	gb|AHGY02208176.1|:883-2754
NuwaII-13_CPB	gb|AHGY02205557.1|:1999-3612
NuwaII-13_CPB	gb|AHGY02002947.1|:288-3534
NuwaII-13_CPB	gb|AHGY02026601.1|:2-1187
NuwaII-13_CPB	gb|AHGY02055491.1|:173815-177911
NuwaII-13_CPB	gb|AHGY02001778.1|:8199-10424
NuwaII-13_CPB	gb|AHGY02208835.1|:6407-7905
NuwaII-13_CPB	gb|AHGY02232150.1|:7694-11140
NuwaII-13_CPB	gb|AHGY02258230.1|:42488-42910
NuwaII-13_CPB	gb|AHGY02258230.1|:28574-31214
NuwaII-13_CPB	gb|AHGY02089463.1|:5619-6687
NuwaII-13_CPB	gb|AHGY02206867.1|:16898-19192
NuwaII-13_CPB	gb|AHGY02225262.1|:19450-22804
NuwaII-13_CPB	gb|AHGY02212021.1|:1616-5765
NuwaII-13_CPB	gb|AHGY02001580.1|:20137-21173
NuwaII-13_CPB	gb|AHGY02001096.1|:36585-38477
NuwaII-13_CPB	gb|AHGY02230782.1|:16226-19962
NuwaII-13_CPB	gb|AHGY02186119.1|:1030-2403
NuwaII-13_CPB	gb|AHGY02229990.1|:10565-11877
NuwaII-13_CPB	gb|AHGY02237626.1|:970-1456
NuwaII-13_CPB	gb|AHGY02150056.1|:13-815
NuwaII-13_CPB	gb|AHGY02040617.1|:10271-13230
NuwaII-13_CPB	gb|AHGY02200076.1|:14084-17137
NuwaII-13_CPB	gb|AHGY02200076.1|:11499-11570

NuwaII-13_CPB	gb|AHGY02213927.1|:22774-23783
NuwaII-13_CPB	gb|AHGY02084020.1|:1306-3870
NuwaII-13_CPB	gb|AHGY02075261.1|:9247-9919
NuwaII-13_CPB	gb|AHGY02075261.1|:67294-68282
NuwaII-13_CPB	gb|AHGY02021950.1|:14886-18550
NuwaII-13_CPB	gb|AHGY02008333.1|:34756-37242
NuwaII-13_CPB	gb|AHGY02039201.1|:15633-17016
NuwaII-13_CPB	gb|AHGY02256541.1|:25362-26382
NuwaII-13_CPB	gb|AHGY02039631.1|:27872-28643
NuwaII-13_CPB	gb|AHGY02039631.1|:42253-45683
NuwaII-13_CPB	gb|AHGY02252556.1|:470-1438
NuwaII-13_CPB	gb|AHGY02221225.1|:5170-5923
NuwaII-13_CPB	gb|AHGY02254407.1|:2633-8934
NuwaII-13_CPB	gb|AHGY02255660.1|:5655-7010
NuwaII-13_CPB	gb|AHGY02252666.1|:319-1084
NuwaII-13_CPB	gb|AHGY02026913.1|:32242-35455
NuwaII-13_CPB	gb|AHGY02026913.1|:12144-12521
NuwaII-13_CPB	gb|AHGY02232571.1|:17829-20591
NuwaII-13_CPB	gb|AHGY02212087.1|:6143-9245
NuwaII-13_CPB	gb|AHGY02043207.1|:2057-3035
NuwaII-13_CPB	gb|AHGY02006947.1|:8355-9370
NuwaII-13_CPB	gb|AHGY02196394.1|:12358-17136
NuwaII-13_CPB	gb|AHGY02047339.1|:11626-13332
NuwaII-13_CPB	gb|AHGY02039106.1|:569-1562
NuwaII-13_CPB	gb|AHGY02259413.1|:11762-15206
NuwaII-13_CPB	gb|AHGY02221062.1|:38075-38973
NuwaII-13_CPB	gb|AHGY02067286.1|:7621-10076
NuwaII-13_CPB	gb|AHGY02055830.1|:10438-13666
NuwaII-13_CPB	gb|AHGY02013704.1|:12397-15483
NuwaII-13_CPB	gb|AHGY02000462.1|:23414-24615
NuwaII-13_CPB	gb|AHGY02251010.1|:15511-24631
NuwaII-13_CPB	gb|AHGY02077349.1|:5493-8639
NuwaII-13_CPB	gb|AHGY02240331.1|:1070-1968
NuwaII-13_CPB	gb|AHGY02207102.1|:26054-29984
NuwaII-13_CPB	gb|AHGY02251164.1|:8330-12038
NuwaII-13_CPB	gb|AHGY02056420.1|:12365-13243
NuwaII-13_CPB	gb|AHGY02096510.1|:27789-30438
NuwaII-13_CPB	gb|AHGY02035416.1|:4740-5514
NuwaII-13_CPB	gb|AHGY02187310.1|:5-536
NuwaII-13_CPB	gb|AHGY02240336.1|:57682-60195
NuwaII-13_CPB	gb|AHGY02047970.1|:12176-12528
NuwaII-13_CPB	gb|AHGY02047970.1|:2112-6001
NuwaII-13_CPB	gb|AHGY02101029.1|:2402-5255
NuwaII-13_CPB	gb|AHGY02243621.1|:5392-8756

NuwaII-13_CPB	gb|AHGY02228692.1|:49806-52445
NuwaII-13_CPB	gb|AHGY02096477.1|:455-3527
NuwaII-13_CPB	gb|AHGY02196036.1|:1511-2518
NuwaII-13_CPB	gb|AHGY02035034.1|:897-3516
NuwaII-13_CPB	gb|AHGY02065456.1|:5457-8455
NuwaII-13_CPB	gb|AHGY02043988.1|:10475-16269
NuwaII-13_CPB	gb|AHGY02249603.1|:2451-3238
NuwaII-13_CPB	gb|AHGY02223293.1|:7429-10511
NuwaII-13_CPB	gb|AHGY02250990.1|:1404-2076
NuwaII-13_CPB	gb|AHGY02115344.1|:5011-7465
NuwaII-13_CPB	gb|AHGY02094449.1|:991-3791
NuwaII-13_CPB	gb|AHGY02078873.1|:14837-15612
NuwaII-13_CPB	gb|AHGY02012515.1|:3899-9032
NuwaII-13_CPB	gb|AHGY02095841.1|:18167-18952
NuwaII-13_CPB	gb|AHGY02259536.1|:1315-4835
NuwaII-13_CPB	gb|AHGY02259536.1|:35409-35975
NuwaII-13_CPB	gb|AHGY02069879.1|:17044-19497
NuwaII-13_CPB	gb|AHGY02057423.1|:5812-6825
NuwaII-13_CPB	gb|AHGY02000904.1|:8426-13165
NuwaII-13_CPB	gb|AHGY02071290.1|:5234-6298
NuwaII-13_CPB	gb|AHGY02040030.1|:34865-35658
NuwaII-13_CPB	gb|AHGY02214848.1|:1126-2136
NuwaII-13_CPB	gb|AHGY02115148.1|:94-1107
NuwaII-13_CPB	gb|AHGY02065985.1|:13454-14789
NuwaII-13_CPB	gb|AHGY02254798.1|:26512-27878
NuwaII-13_CPB	gb|AHGY02040781.1|:4069-5435
NuwaII-13_CPB	gb|AHGY02001842.1|:1631-4986
NuwaII-13_CPB	gb|AHGY02001842.1|:19730-20511
NuwaII-14_CPB	gb|AHGY02239776.1|:1905-1958
NuwaII-14_CPB	gb|AHGY02239776.1|:22130-26039
NuwaII-14_CPB	gb|AHGY02239776.1|:4739-4837
NuwaII-14_CPB	gb|AHGY02203391.1|:341-4230
NuwaII-14_CPB	gb|AHGY02027031.1|:8172-12146
NuwaII-14_CPB	gb|AHGY02253632.1|:3155-7115
NuwaII-14_CPB	gb|AHGY02253632.1|:20045-20107
NuwaII-14_CPB	gb|AHGY02043482.1|:974-5028
NuwaII-14_CPB	gb|AHGY02261793.1|:13051-16932
NuwaII-14_CPB	gb|AHGY02233455.1|:7072-10975
NuwaII-14_CPB	gb|AHGY02206922.1|:12478-16457
NuwaII-14_CPB	gb|AHGY02102347.1|:1413-3225
NuwaII-14_CPB	gb|AHGY02073802.1|:6-3040
NuwaII-14_CPB	gb|AHGY02101604.1|:7299-10214
NuwaII-14_CPB	gb|AHGY02261768.1|:991-4949
NuwaII-14_CPB	gb|AHGY02261968.1|:25-129

NuwaII-14_CPB	gb|AHGY02261968.1|:13069-16938
NuwaII-14_CPB	gb|AHGY02102344.1|:267-4240
NuwaII-14_CPB	gb|AHGY02252858.1|:31993-35937
NuwaII-14_CPB	gb|AHGY02252858.1|:81011-81095
NuwaII-14_CPB	gb|AHGY02252858.1|:94704-94805
NuwaII-14_CPB	gb|AHGY02252858.1|:110326-110422
NuwaII-14_CPB	gb|AHGY02252858.1|:40508-40590
NuwaII-14_CPB	gb|AHGY02055034.1|:14529-18115
NuwaII-14_CPB	gb|AHGY02055034.1|:13386-13436
NuwaII-14_CPB	gb|AHGY02078393.1|:28-82
NuwaII-14_CPB	gb|AHGY02078393.1|:7187-11133
NuwaII-14_CPB	gb|AHGY02237554.1|:9330-13275
NuwaII-14_CPB	gb|AHGY02049148.1|:14017-18666
NuwaII-14_CPB	gb|AHGY02030974.1|:10153-10216
NuwaII-14_CPB	gb|AHGY02030974.1|:75293-77964
NuwaII-14_CPB	gb|AHGY02030974.1|:68910-68978
NuwaII-14_CPB	gb|AHGY02030974.1|:9933-10034
NuwaII-14_CPB	gb|AHGY02213642.1|:3617-7592
NuwaII-14_CPB	gb|AHGY02237724.1|:2573-6518
NuwaII-14_CPB	gb|AHGY02233200.1|:20087-23483
NuwaII-14_CPB	gb|AHGY02233200.1|:31514-31598
NuwaII-14_CPB	gb|AHGY02066243.1|:14454-16547
NuwaII-14_CPB	gb|AHGY02238994.1|:1677-19463
NuwaII-14_CPB	gb|AHGY02238994.1|:14686-18641
NuwaII-14_CPB	gb|AHGY02258239.1|:19252-19315
NuwaII-14_CPB	gb|AHGY02258239.1|:42096-44185
NuwaII-14_CPB	gb|AHGY02258239.1|:28653-29289
NuwaII-14_CPB	gb|AHGY02092663.1|:27976-32111
NuwaII-14_CPB	gb|AHGY02178704.1|:656-2860
NuwaII-14_CPB	gb|AHGY02106293.1|:1011-2799
NuwaII-14_CPB	gb|AHGY02093847.1|:5655-5874
NuwaII-14_CPB	gb|AHGY02093847.1|:2650-5519
NuwaII-14_CPB	gb|AHGY02080715.1|:2732-6682
NuwaII-14_CPB	gb|AHGY02248126.1|:2802-7563
NuwaII-14_CPB	gb|AHGY02077733.1|:4925-8574
NuwaII-14_CPB	gb|AHGY02223512.1|:62-132
NuwaII-14_CPB	gb|AHGY02223512.1|:131-3324
NuwaII-14_CPB	gb|AHGY02027055.1|:3298-5036
NuwaII-14_CPB	gb|AHGY02083126.1|:30978-31079
NuwaII-14_CPB	gb|AHGY02083126.1|:21176-25212
NuwaII-14_CPB	gb|AHGY02114668.1|:309-4469
NuwaII-14_CPB	gb|AHGY02044818.1|:22274-26160
NuwaII-14_CPB	gb|AHGY02232335.1|:34578-39872
NuwaII-14_CPB	gb|AHGY02249382.1|:7283-11213

NuwaII-14_CPB	gb|AHGY02115113.1|:3186-5375
NuwaII-14_CPB	gb|AHGY02115113.1|:5375-5784
NuwaII-14_CPB	gb|AHGY02058526.1|:6-2915
NuwaII-14_CPB	gb|AHGY02185362.1|:1-2297
NuwaII-14_CPB	gb|AHGY02031609.1|:10197-12821
NuwaII-14_CPB	gb|AHGY02077071.1|:2136-2237
NuwaII-14_CPB	gb|AHGY02077071.1|:1-3872
NuwaII-14_CPB	gb|AHGY02183108.1|:895-2584
NuwaII-14_CPB	gb|AHGY02067455.1|:7844-11777
NuwaII-14_CPB	gb|AHGY02014668.1|:3068-7019
NuwaII-14_CPB	gb|AHGY02048034.1|:44024-47746
NuwaII-14_CPB	gb|AHGY02048034.1|:24900-28807
NuwaII-14_CPB	gb|AHGY02245208.1|:11641-11694
NuwaII-14_CPB	gb|AHGY02245208.1|:30306-34223
NuwaII-14_CPB	gb|AHGY02109687.1|:22230-22283
NuwaII-14_CPB	gb|AHGY02109687.1|:3756-8199
NuwaII-14_CPB	gb|AHGY02101396.1|:666-5223
NuwaII-14_CPB	gb|AHGY02048764.1|:15837-19670
NuwaII-14_CPB	gb|AHGY02048764.1|:29970-30026
NuwaII-14_CPB	gb|AHGY02048764.1|:29657-29754
NuwaII-14_CPB	gb|AHGY02253811.1|:21148-21249
NuwaII-14_CPB	gb|AHGY02253811.1|:1728-5354
NuwaII-14_CPB	gb|AHGY02211940.1|:15235-18631
NuwaII-14_CPB	gb|AHGY02241859.1|:709-2566
NuwaII-14_CPB	gb|AHGY02208219.1|:15928-19650
NuwaII-14_CPB	gb|AHGY02208219.1|:10727-10785
NuwaII-14_CPB	gb|AHGY02180710.1|:700-2766
NuwaII-14_CPB	gb|AHGY02013646.1|:5089-8148
NuwaII-14_CPB	gb|AHGY02078359.1|:6-4193
NuwaII-14_CPB	gb|AHGY02001773.1|:13384-17059
NuwaII-14_CPB	gb|AHGY02034994.1|:1-3566
NuwaII-14_CPB	gb|AHGY02245640.1|:1705-5438
NuwaII-14_CPB	gb|AHGY02042282.1|:2140-4209
NuwaII-14_CPB	gb|AHGY02095856.1|:766-829
NuwaII-14_CPB	gb|AHGY02095856.1|:573-7195
NuwaII-14_CPB	gb|AHGY02092628.1|:4495-6194
NuwaII-14_CPB	gb|AHGY02114237.1|:1-2708
NuwaII-14_CPB	gb|AHGY02057716.1|:15518-18849
NuwaII-14_CPB	gb|AHGY02057716.1|:8296-8435
NuwaII-14_CPB	gb|AHGY02235242.1|:2-3837
NuwaII-14_CPB	gb|AHGY02235242.1|:4367-4430
NuwaII-14_CPB	gb|AHGY02256369.1|:158-260
NuwaII-14_CPB	gb|AHGY02256369.1|:1158-5059
NuwaII-14_CPB	gb|AHGY02237208.1|:1635-5593

NuwaII-14_CPB	gb|AHGY02247155.1|:30-131
NuwaII-14_CPB	gb|AHGY02247155.1|:22830-27679
NuwaII-14_CPB	gb|AHGY02085439.1|:21460-25319
NuwaII-14_CPB	gb|AHGY02099365.1|:4257-7343
NuwaII-14_CPB	gb|AHGY02258094.1|:8336-12220
NuwaII-14_CPB	gb|AHGY02258094.1|:1-1384
NuwaII-14_CPB	gb|AHGY02099430.1|:4516-6286
NuwaII-14_CPB	gb|AHGY02254570.1|:10086-10196
NuwaII-14_CPB	gb|AHGY02254570.1|:4982-8742
NuwaII-14_CPB	gb|AHGY02060637.1|:758-4414
NuwaII-14_CPB	gb|AHGY02060637.1|:20598-20662
NuwaII-14_CPB	gb|AHGY02060637.1|:20487-20587
NuwaII-14_CPB	gb|AHGY02026223.1|:13525-17220
NuwaII-14_CPB	gb|AHGY02234308.1|:14090-16063
NuwaII-14_CPB	gb|AHGY02083479.1|:25559-29505
NuwaII-14_CPB	gb|AHGY02246366.1|:9134-13885
NuwaII-14_CPB	gb|AHGY02246366.1|:74403-74499
NuwaII-14_CPB	gb|AHGY02236618.1|:24571-27055
NuwaII-14_CPB	gb|AHGY02079787.1|:44-1822
NuwaII-14_CPB	gb|AHGY02253970.1|:2493-6433
NuwaII-14_CPB	gb|AHGY02227615.1|:11499-14152
NuwaII-14_CPB	gb|AHGY02058367.1|:28-2571
NuwaII-14_CPB	gb|AHGY02259327.1|:1263-4992
NuwaII-14_CPB	gb|AHGY02011449.1|:27535-27589
NuwaII-14_CPB	gb|AHGY02011449.1|:3628-7534
NuwaII-14_CPB	gb|AHGY02216281.1|:102424-105173
NuwaII-14_CPB	gb|AHGY02011510.1|:1842-8392
NuwaII-14_CPB	gb|AHGY02216961.1|:335-4306
NuwaII-14_CPB	gb|AHGY02061455.1|:30810-30873
NuwaII-14_CPB	gb|AHGY02061455.1|:58135-61953
NuwaII-14_CPB	gb|AHGY02061455.1|:90489-90591
NuwaII-14_CPB	gb|AHGY02061455.1|:94819-94888
NuwaII-14_CPB	gb|AHGY02061455.1|:33857-33963
NuwaII-14_CPB	gb|AHGY02061455.1|:1097-1237
NuwaII-14_CPB	gb|AHGY02240108.1|:93-2369
NuwaII-14_CPB	gb|AHGY02238883.1|:59-3382
NuwaII-14_CPB	gb|AHGY02252539.1|:11123-11185
NuwaII-14_CPB	gb|AHGY02252539.1|:11007-14845
NuwaII-14_CPB	gb|AHGY02114163.1|:1-3211
NuwaII-14_CPB	gb|AHGY02259429.1|:21736-25396
NuwaII-14_CPB	gb|AHGY02259429.1|:14260-14309
NuwaII-14_CPB	gb|AHGY02044706.1|:3986-4081
NuwaII-14_CPB	gb|AHGY02044706.1|:11246-16719
NuwaII-14_CPB	gb|AHGY02011989.1|:15155-20776

NuwaII-14_CPB	gb|AHGY02011989.1|:20535-20605
NuwaII-14_CPB	gb|AHGY02011989.1|:5406-9093
NuwaII-14_CPB	gb|AHGY02213473.1|:20456-20566
NuwaII-14_CPB	gb|AHGY02213473.1|:64746-64829
NuwaII-14_CPB	gb|AHGY02213473.1|:40510-44648
NuwaII-14_CPB	gb|AHGY02074948.1|:19935-23601
NuwaII-14_CPB	gb|AHGY02225605.1|:3293-5379
NuwaII-14_CPB	gb|AHGY02225605.1|:29721-29798
NuwaII-14_CPB	gb|AHGY02074834.1|:16242-20016
NuwaII-14_CPB	gb|AHGY02198533.1|:30956-34895
NuwaII-14_CPB	gb|AHGY02012431.1|:2533-6202
NuwaII-14_CPB	gb|AHGY02012431.1|:8639-8735
NuwaII-14_CPB	gb|AHGY02036728.1|:8450-12340
NuwaII-14_CPB	gb|AHGY02102728.1|:7829-10338
NuwaII-14_CPB	gb|AHGY02102728.1|:10360-10423
NuwaII-14_CPB	gb|AHGY02060708.1|:6535-10357
NuwaII-14_CPB	gb|AHGY02222939.1|:30901-34749
NuwaII-14_CPB	gb|AHGY02073957.1|:17674-17743
NuwaII-14_CPB	gb|AHGY02073957.1|:5512-9404
NuwaII-14_CPB	gb|AHGY02055356.1|:1856-5734
NuwaII-14_CPB	gb|AHGY02055356.1|:16749-16815
NuwaII-14_CPB	gb|AHGY02055356.1|:10049-14610
NuwaII-14_CPB	gb|AHGY02029237.1|:9098-19213
NuwaII-14_CPB	gb|AHGY02208115.1|:3869-7454
NuwaII-14_CPB	gb|AHGY02008738.1|:16108-17553
NuwaII-14_CPB	gb|AHGY02078647.1|:9749-12945
NuwaII-14_CPB	gb|AHGY02078647.1|:22079-22199
NuwaII-14_CPB	gb|AHGY02001458.1|:29125-32768
NuwaII-14_CPB	gb|AHGY02080158.1|:20432-24106
NuwaII-14_CPB	gb|AHGY02058686.1|:7-2060
NuwaII-14_CPB	gb|AHGY02261183.1|:10915-11005
NuwaII-14_CPB	gb|AHGY02261183.1|:32118-32219
NuwaII-14_CPB	gb|AHGY02261183.1|:31777-31840
NuwaII-14_CPB	gb|AHGY02261183.1|:10367-13175
NuwaII-14_CPB	gb|AHGY02118048.1|:777-878
NuwaII-14_CPB	gb|AHGY02118048.1|:15-2248
NuwaII-14_CPB	gb|AHGY02043425.1|:2848-6774
NuwaII-14_CPB	gb|AHGY02077797.1|:7360-10882
NuwaII-14_CPB	gb|AHGY02244713.1|:13102-16887
NuwaII-14_CPB	gb|AHGY02008211.1|:2112-6059
NuwaII-14_CPB	gb|AHGY02251896.1|:17279-20870
NuwaII-14_CPB	gb|AHGY02088708.1|:26221-26325
NuwaII-14_CPB	gb|AHGY02088708.1|:53-1930
NuwaII-14_CPB	gb|AHGY02196445.1|:6953-10908

NuwaII-14_CPB	gb|AHGY02030407.1|:9160-12970
NuwaII-14_CPB	gb|AHGY02240577.1|:27597-27661
NuwaII-14_CPB	gb|AHGY02240577.1|:21585-21687
NuwaII-14_CPB	gb|AHGY02240577.1|:697-4169
NuwaII-14_CPB	gb|AHGY02213328.1|:21463-25359
NuwaII-14_CPB	gb|AHGY02213328.1|:34950-35019
NuwaII-14_CPB	gb|AHGY02213328.1|:18751-18851
NuwaII-14_CPB	gb|AHGY02044057.1|:6725-8678
NuwaII-14_CPB	gb|AHGY02116597.1|:541-3454
NuwaII-14_CPB	gb|AHGY02095459.1|:676-4849
NuwaII-14_CPB	gb|AHGY02051419.1|:19712-23538
NuwaII-14_CPB	gb|AHGY02073252.1|:2014-6959
NuwaII-14_CPB	gb|AHGY02073252.1|:6598-6648
NuwaII-14_CPB	gb|AHGY02247069.1|:1761-5493
NuwaII-14_CPB	gb|AHGY02236493.1|:4335-4390
NuwaII-14_CPB	gb|AHGY02236493.1|:22987-25381
NuwaII-14_CPB	gb|AHGY02236493.1|:3936-3993
NuwaII-14_CPB	gb|AHGY02224684.1|:1778-6060
NuwaII-14_CPB	gb|AHGY02253865.1|:27973-28079
NuwaII-14_CPB	gb|AHGY02253865.1|:11418-16488
NuwaII-14_CPB	gb|AHGY02253865.1|:243-412
NuwaII-14_CPB	gb|AHGY02085430.1|:10632-14328
NuwaII-14_CPB	gb|AHGY02060164.1|:31767-32441
NuwaII-14_CPB	gb|AHGY02060164.1|:9216-12878
NuwaII-14_CPB	gb|AHGY02259627.1|:651-3456
NuwaII-14_CPB	gb|AHGY02008872.1|:2071-5402
NuwaII-14_CPB	gb|AHGY02029656.1|:10436-16268
NuwaII-14_CPB	gb|AHGY02202301.1|:4424-8129
NuwaII-14_CPB	gb|AHGY02027347.1|:15-84
NuwaII-14_CPB	gb|AHGY02027347.1|:2094-5938
NuwaII-14_CPB	gb|AHGY02073837.1|:3928-7640
NuwaII-14_CPB	gb|AHGY02071612.1|:1141-3934
NuwaII-14_CPB	gb|AHGY02237386.1|:2523-4473
NuwaII-14_CPB	gb|AHGY02043873.1|:2668-7181
NuwaII-14_CPB	gb|AHGY02063067.1|:137-2178
NuwaII-14_CPB	gb|AHGY02084697.1|:9011-22414
NuwaII-14_CPB	gb|AHGY02084697.1|:55253-55350
NuwaII-14_CPB	gb|AHGY02252145.1|:25013-25557
NuwaII-14_CPB	gb|AHGY02252145.1|:15699-19496
NuwaII-14_CPB	gb|AHGY02058598.1|:35627-35696
NuwaII-14_CPB	gb|AHGY02058598.1|:22482-31222
NuwaII-14_CPB	gb|AHGY02078424.1|:6556-10377
NuwaII-14_CPB	gb|AHGY02013124.1|:278-382
NuwaII-14_CPB	gb|AHGY02013124.1|:13416-18420

NuwaII-14_CPB	gb|AHGY02013124.1|:32023-32124
NuwaII-14_CPB	gb|AHGY02013124.1|:7937-8071
NuwaII-14_CPB	gb|AHGY02252341.1|:13920-17510
NuwaII-14_CPB	gb|AHGY02094518.1|:960-4677
NuwaII-14_CPB	gb|AHGY02024508.1|:2-1678
NuwaII-14_CPB	gb|AHGY02260603.1|:54893-54962
NuwaII-14_CPB	gb|AHGY02260603.1|:38631-42360
NuwaII-14_CPB	gb|AHGY02112583.1|:635-2730
NuwaII-14_CPB	gb|AHGY02225485.1|:67305-67962
NuwaII-14_CPB	gb|AHGY02225485.1|:78039-78156
NuwaII-14_CPB	gb|AHGY02225485.1|:103442-107375
NuwaII-14_CPB	gb|AHGY02067897.1|:2325-12013
NuwaII-14_CPB	gb|AHGY02078579.1|:1033-3459
NuwaII-14_CPB	gb|AHGY02024415.1|:164-2334
NuwaII-14_CPB	gb|AHGY02063000.1|:24776-28298
NuwaII-14_CPB	gb|AHGY02063000.1|:2369-2599
NuwaII-14_CPB	gb|AHGY02222879.1|:1456-5154
NuwaII-14_CPB	gb|AHGY02061094.1|:77-3418
NuwaII-14_CPB	gb|AHGY02249483.1|:8216-12082
NuwaII-14_CPB	gb|AHGY02249483.1|:39963-40020
NuwaII-14_CPB	gb|AHGY02261845.1|:5356-8056
NuwaII-14_CPB	gb|AHGY02261845.1|:9223-9297
NuwaII-14_CPB	gb|AHGY02013508.1|:1449-5482
NuwaII-14_CPB	gb|AHGY02004434.1|:1-3520
NuwaII-14_CPB	gb|AHGY02004434.1|:38224-38393
NuwaII-14_CPB	gb|AHGY02119305.1|:397-4350
NuwaII-14_CPB	gb|AHGY02097401.1|:2-2803
NuwaII-14_CPB	gb|AHGY02044731.1|:103-2505
NuwaII-14_CPB	gb|AHGY02262217.1|:13729-13797
NuwaII-14_CPB	gb|AHGY02262217.1|:30594-30657
NuwaII-14_CPB	gb|AHGY02262217.1|:30061-30161
NuwaII-14_CPB	gb|AHGY02262217.1|:2478-7174
NuwaII-14_CPB	gb|AHGY02223920.1|:18365-20116
NuwaII-14_CPB	gb|AHGY02223920.1|:8416-8494
NuwaII-14_CPB	gb|AHGY02259418.1|:29289-31307
NuwaII-14_CPB	gb|AHGY02259418.1|:8216-8303
NuwaII-14_CPB	gb|AHGY02052091.1|:3054-3117
NuwaII-14_CPB	gb|AHGY02052091.1|:20124-20172
NuwaII-14_CPB	gb|AHGY02052091.1|:17972-18074
NuwaII-14_CPB	gb|AHGY02052091.1|:214-1998
NuwaII-14_CPB	gb|AHGY02214204.1|:16552-20294
NuwaII-14_CPB	gb|AHGY02214204.1|:31172-31256
NuwaII-14_CPB	gb|AHGY02052389.1|:16930-16985
NuwaII-14_CPB	gb|AHGY02052389.1|:59666-59754

NuwaII-14_CPB	gb|AHGY02052389.1|:89691-89762
NuwaII-14_CPB	gb|AHGY02052389.1|:115619-121458
NuwaII-14_CPB	gb|AHGY02052389.1|:89576-89639
NuwaII-14_CPB	gb|AHGY02052389.1|:16655-16749
NuwaII-14_CPB	gb|AHGY02084739.1|:11819-15536
NuwaII-14_CPB	gb|AHGY02201737.1|:47588-51443
NuwaII-14_CPB	gb|AHGY02058257.1|:7-1646
NuwaII-14_CPB	gb|AHGY02054134.1|:5587-9315
NuwaII-14_CPB	gb|AHGY02206854.1|:2757-6487
NuwaII-14_CPB	gb|AHGY02237664.1|:24442-28177
NuwaII-14_CPB	gb|AHGY02237664.1|:6528-6629
NuwaII-14_CPB	gb|AHGY02023614.1|:1264-4995
NuwaII-14_CPB	gb|AHGY02203810.1|:583-636
NuwaII-14_CPB	gb|AHGY02203810.1|:1251-3210
NuwaII-14_CPB	gb|AHGY02078559.1|:12025-13915
NuwaII-14_CPB	gb|AHGY02058265.1|:67-1511
NuwaII-14_CPB	gb|AHGY02261559.1|:25320-29059
NuwaII-14_CPB	gb|AHGY02010675.1|:6026-7480
NuwaII-14_CPB	gb|AHGY02010675.1|:26166-28227
NuwaII-14_CPB	gb|AHGY02007578.1|:481-2526
NuwaII-14_CPB	gb|AHGY02099863.1|:72-1918
NuwaII-14_CPB	gb|AHGY02073396.1|:36737-39540
NuwaII-14_CPB	gb|AHGY02073396.1|:33597-33647
NuwaII-14_CPB	gb|AHGY02066259.1|:24608-24709
NuwaII-14_CPB	gb|AHGY02066259.1|:37547-37648
NuwaII-14_CPB	gb|AHGY02066259.1|:29418-36755
NuwaII-14_CPB	gb|AHGY02035674.1|:4-1933
NuwaII-14_CPB	gb|AHGY02199945.1|:3962-7698
NuwaII-14_CPB	gb|AHGY02199945.1|:332-462
NuwaII-14_CPB	gb|AHGY02261731.1|:88-2609
NuwaII-14_CPB	gb|AHGY02102537.1|:1-2191
NuwaII-14_CPB	gb|AHGY02058454.1|:2318-4344
NuwaII-14_CPB	gb|AHGY02255732.1|:400-3105
NuwaII-14_CPB	gb|AHGY02083253.1|:2266-6195
NuwaII-14_CPB	gb|AHGY02245405.1|:40-3142
NuwaII-14_CPB	gb|AHGY02232118.1|:26993-29059
NuwaII-14_CPB	gb|AHGY02072020.1|:2-3673
NuwaII-14_CPB	gb|AHGY02034986.1|:71099-73141
NuwaII-14_CPB	gb|AHGY02059148.1|:12698-15288
NuwaII-14_CPB	gb|AHGY02059148.1|:15282-15358
NuwaII-14_CPB	gb|AHGY02000436.1|:454-4303
NuwaII-14_CPB	gb|AHGY02075237.1|:5544-9264
NuwaII-14_CPB	gb|AHGY02058799.1|:6770-9178
NuwaII-14_CPB	gb|AHGY02029657.1|:19195-19252

NuwaII-14_CPB	gb|AHGY02029657.1|:42056-42131
NuwaII-14_CPB	gb|AHGY02029657.1|:22059-24758
NuwaII-14_CPB	gb|AHGY02071178.1|:61315-65059
NuwaII-14_CPB	gb|AHGY02134154.1|:1-1541
NuwaII-14_CPB	gb|AHGY02257471.1|:19016-19496
NuwaII-14_CPB	gb|AHGY02257471.1|:564-2286
NuwaII-14_CPB	gb|AHGY02032248.1|:17711-19774
NuwaII-14_CPB	gb|AHGY02032248.1|:7313-7367
NuwaII-14_CPB	gb|AHGY02262159.1|:43801-43903
NuwaII-14_CPB	gb|AHGY02262159.1|:78668-82336
NuwaII-14_CPB	gb|AHGY02262159.1|:19511-19600
NuwaII-14_CPB	gb|AHGY02200150.1|:43-2102
NuwaII-14_CPB	gb|AHGY02202387.1|:15813-19777
NuwaII-14_CPB	gb|AHGY02188293.1|:45-2442
NuwaII-14_CPB	gb|AHGY02259664.1|:17292-19809
NuwaII-14_CPB	gb|AHGY02011110.1|:165-2331
NuwaII-14_CPB	gb|AHGY02105713.1|:11153-14851
NuwaII-14_CPB	gb|AHGY02219416.1|:6295-9939
NuwaII-14_CPB	gb|AHGY02120913.1|:354-1517
NuwaII-14_CPB	gb|AHGY02013615.1|:3-88
NuwaII-14_CPB	gb|AHGY02013615.1|:22534-26293
NuwaII-14_CPB	gb|AHGY02013615.1|:13518-13584
NuwaII-14_CPB	gb|AHGY02233924.1|:36228-40086
NuwaII-14_CPB	gb|AHGY02049726.1|:7-2782
NuwaII-14_CPB	gb|AHGY02027047.1|:8171-10672
NuwaII-14_CPB	gb|AHGY02012474.1|:8497-12225
NuwaII-14_CPB	gb|AHGY02241249.1|:19253-23200
NuwaII-14_CPB	gb|AHGY02081431.1|:3772-3859
NuwaII-14_CPB	gb|AHGY02081431.1|:5816-9504
NuwaII-14_CPB	gb|AHGY02042899.1|:26675-26738
NuwaII-14_CPB	gb|AHGY02042899.1|:88-2521
NuwaII-14_CPB	gb|AHGY02252318.1|:6569-8944
NuwaII-14_CPB	gb|AHGY02061318.1|:23-1937
NuwaII-14_CPB	gb|AHGY02213227.1|:23-2104
NuwaII-14_CPB	gb|AHGY02204492.1|:35-2221
NuwaII-14_CPB	gb|AHGY02061797.1|:1-3116
NuwaII-14_CPB	gb|AHGY02096926.1|:5988-8142
NuwaII-14_CPB	gb|AHGY02096926.1|:1914-5373
NuwaII-14_CPB	gb|AHGY02001098.1|:3116-4897
NuwaII-14_CPB	gb|AHGY02133785.1|:1-1568
NuwaII-14_CPB	gb|AHGY02008232.1|:11733-15486
NuwaII-14_CPB	gb|AHGY02008232.1|:625-790
NuwaII-14_CPB	gb|AHGY02249883.1|:1752-3937
NuwaII-14_CPB	gb|AHGY02081089.1|:2428-2596

NuwaII-14_CPB	gb|AHGY02081089.1|:183-2239
NuwaII-14_CPB	gb|AHGY02245030.1|:293-3658
NuwaII-14_CPB	gb|AHGY02020840.1|:9106-11156
NuwaII-14_CPB	gb|AHGY02016019.1|:97-6346
NuwaII-14_CPB	gb|AHGY02251079.1|:1603-12457
NuwaII-14_CPB	gb|AHGY02251079.1|:12046-12153
NuwaII-15_CPB	gb|AHGY02084693.1|:75-2355
NuwaII-15_CPB	gb|AHGY02133214.1|:42-1625
NuwaII-15_CPB	gb|AHGY02035606.1|:1690-4074
NuwaII-15_CPB	gb|AHGY02006853.1|:45-3444
NuwaII-15_CPB	gb|AHGY02202229.1|:53-4723
NuwaII-15_CPB	gb|AHGY02260939.1|:28588-31478
NuwaII-15_CPB	gb|AHGY02222321.1|:29967-32361
NuwaII-15_CPB	gb|AHGY02088786.1|:3288-7117
NuwaII-15_CPB	gb|AHGY02099477.1|:1-3124
NuwaII-15_CPB	gb|AHGY02109758.1|:18317-20695
NuwaII-15_CPB	gb|AHGY02234067.1|:31-3518
NuwaII-15_CPB	gb|AHGY02024197.1|:52638-55316
NuwaII-15_CPB	gb|AHGY02131009.1|:44-1776
NuwaII-15_CPB	gb|AHGY02000770.1|:18039-20352
NuwaII-15_CPB	gb|AHGY02053294.1|:2692-5811
NuwaII-15_CPB	gb|AHGY02002346.1|:3349-7470
NuwaII-15_CPB	gb|AHGY02052682.1|:34-2363
NuwaII-15_CPB	gb|AHGY02029193.1|:20940-24511
NuwaII-15_CPB	gb|AHGY02029193.1|:1-3247
NuwaII-15_CPB	gb|AHGY02048320.1|:27974-31607
NuwaII-15_CPB	gb|AHGY02210811.1|:36587-40693
NuwaII-15_CPB	gb|AHGY02107736.1|:3208-6210
NuwaII-15_CPB	gb|AHGY02048707.1|:56976-59676
NuwaII-15_CPB	gb|AHGY02069790.1|:7206-11138
NuwaII-15_CPB	gb|AHGY02007886.1|:17-3508
NuwaII-15_CPB	gb|AHGY02230484.1|:29529-29835
NuwaII-15_CPB	gb|AHGY02230484.1|:1-3131
NuwaII-15_CPB	gb|AHGY02205389.1|:76614-79932
NuwaII-15_CPB	gb|AHGY02026112.1|:19147-23164
NuwaII-15_CPB	gb|AHGY02086929.1|:43-1724
NuwaII-15_CPB	gb|AHGY02020947.1|:1-2784
NuwaII-15_CPB	gb|AHGY02061455.1|:59265-59404
NuwaII-15_CPB	gb|AHGY02061455.1|:94459-94781
NuwaII-15_CPB	gb|AHGY02061455.1|:15-3162
NuwaII-15_CPB	gb|AHGY02087355.1|:25-3729
NuwaII-15_CPB	gb|AHGY02201748.1|:6966-11127
NuwaII-15_CPB	gb|AHGY02134575.1|:1-1508
NuwaII-15_CPB	gb|AHGY02056495.1|:1-3471

NuwaII-15_CPB	gb|AHGY02246277.1|:1-2603
NuwaII-15_CPB	gb|AHGY02221535.1|:24-2429
NuwaII-15_CPB	gb|AHGY02074602.1|:9634-13334
NuwaII-15_CPB	gb|AHGY02042662.1|:14364-18539
NuwaII-15_CPB	gb|AHGY02002058.1|:359-4419
NuwaII-15_CPB	gb|AHGY02133763.1|:1-1578
NuwaII-15_CPB	gb|AHGY02261818.1|:29449-31991
NuwaII-15_CPB	gb|AHGY02247126.1|:17428-18394
NuwaII-15_CPB	gb|AHGY02247126.1|:46-2797
NuwaII-15_CPB	gb|AHGY02217147.1|:4574-8241
NuwaII-15_CPB	gb|AHGY02091310.1|:2-2831
NuwaII-15_CPB	gb|AHGY02022881.1|:24500-28627
NuwaII-15_CPB	gb|AHGY02084701.1|:10713-13315
NuwaII-15_CPB	gb|AHGY02022962.1|:41-1689
NuwaII-15_CPB	gb|AHGY02084724.1|:2666-6836
NuwaII-15_CPB	gb|AHGY02052138.1|:3-3117
NuwaII-15_CPB	gb|AHGY02032067.1|:18-2522
NuwaII-15_CPB	gb|AHGY02235100.1|:4872-7981
NuwaII-15_CPB	gb|AHGY02259530.1|:24950-25113
NuwaII-15_CPB	gb|AHGY02259530.1|:52142-56262
NuwaII-15_CPB	gb|AHGY02257750.1|:12627-16360
NuwaII-15_CPB	gb|AHGY02233911.1|:1-2762
NuwaII-15_CPB	gb|AHGY02203695.1|:109-1801
NuwaII-15_CPB	gb|AHGY02062555.1|:949-4160
NuwaII-15_CPB	gb|AHGY02195206.1|:1-2088
NuwaII-15_CPB	gb|AHGY02111270.1|:12437-16678
NuwaII-15_CPB	gb|AHGY02203891.1|:1344-5478
NuwaII-15_CPB	gb|AHGY02030739.1|:668-3883
NuwaII-15_CPB	gb|AHGY02236908.1|:5-2884
NuwaII-15_CPB	gb|AHGY02227878.1|:37-1632
NuwaII-15_CPB	gb|AHGY02074533.1|:7878-11972
NuwaII-15_CPB	gb|AHGY02184476.1|:42-2598
NuwaII-15_CPB	gb|AHGY02042144.1|:27-1466
NuwaII-15_CPB	gb|AHGY02208885.1|:1-2558
NuwaII-15_CPB	gb|AHGY02090192.1|:4030-7734
NuwaII-15_CPB	gb|AHGY02130371.1|:1-1790
NuwaII-15_CPB	gb|AHGY02078239.1|:30-2433
NuwaII-15_CPB	gb|AHGY02068345.1|:3336-7559
NuwaII-15_CPB	gb|AHGY02033898.1|:9415-13410
NuwaII-15_CPB	gb|AHGY02098641.1|:7512-11158
NuwaII-15_CPB	gb|AHGY02091136.1|:2079-6229
NuwaII-15_CPB	gb|AHGY02057964.1|:14412-17401
NuwaII-15_CPB	gb|AHGY02260332.1|:14747-17133
NuwaII-15_CPB	gb|AHGY02225441.1|:39908-42380

NuwaII-15_CPB	gb|AHGY02194794.1|:1-2210
NuwaII-15_CPB	gb|AHGY02089075.1|:1092-4938
NuwaII-15_CPB	gb|AHGY02039531.1|:47641-51691
NuwaII-15_CPB	gb|AHGY02036558.1|:11011-15159
NuwaII-15_CPB	gb|AHGY02261313.1|:751-11517
NuwaII-15_CPB	gb|AHGY02261313.1|:1449-2283
NuwaII-15_CPB	gb|AHGY02236793.1|:1-2541
NuwaII-15_CPB	gb|AHGY02057716.1|:16664-16782
NuwaII-15_CPB	gb|AHGY02057716.1|:6202-10084
NuwaII-15_CPB	gb|AHGY02010431.1|:8120-11352
NuwaII-15_CPB	gb|AHGY02262310.1|:49915-52757
NuwaII-15_CPB	gb|AHGY02094446.1|:1-3040
NuwaII-15_CPB	gb|AHGY02076959.1|:10018-12585
NuwaII-15_CPB	gb|AHGY02064491.1|:13927-17780
NuwaII-15_CPB	gb|AHGY02040711.1|:6228-9750
NuwaII-15_CPB	gb|AHGY02013969.1|:14477-16907
NuwaII-15_CPB	gb|AHGY02196121.1|:146-1828
NuwaII-15_CPB	gb|AHGY02090210.1|:4050-6589
NuwaII-15_CPB	gb|AHGY02085237.1|:28047-32228
NuwaII-15_CPB	gb|AHGY02019494.1|:1861-5354
NuwaII-15_CPB	gb|AHGY02133624.1|:14-1587
NuwaII-15_CPB	gb|AHGY02132275.1|:15-1721
NuwaII-15_CPB	gb|AHGY02033868.1|:14154-16567
NuwaII-15_CPB	gb|AHGY02030436.1|:1888-5990
NuwaII-15_CPB	gb|AHGY02017025.1|:22-3406
NuwaII-15_CPB	gb|AHGY02253567.1|:49-3848
NuwaII-15_CPB	gb|AHGY02023134.1|:1-2799
NuwaII-15_CPB	gb|AHGY02015974.1|:1767-1842
NuwaII-15_CPB	gb|AHGY02015974.1|:30-2470
NuwaII-15_CPB	gb|AHGY02008511.1|:19620-23129
NuwaII-15_CPB	gb|AHGY02195981.1|:1-2894
NuwaII-15_CPB	gb|AHGY02043618.1|:4432-8463
NuwaII-15_CPB	gb|AHGY02013563.1|:14770-18162
NuwaII-15_CPB	gb|AHGY02216597.1|:1-2605
NuwaII-15_CPB	gb|AHGY02022931.1|:1-2898
NuwaII-15_CPB	gb|AHGY02237535.1|:87-2472
NuwaII-15_CPB	gb|AHGY02055802.1|:12882-21491
NuwaII-15_CPB	gb|AHGY02055802.1|:13588-14501
NuwaII-15_CPB	gb|AHGY02007375.1|:56-3298
NuwaII-15_CPB	gb|AHGY02000739.1|:2-2065
NuwaII-15_CPB	gb|AHGY02239896.1|:74-2108
NuwaII-15_CPB	gb|AHGY02235780.1|:391-3929
NuwaII-15_CPB	gb|AHGY02093551.1|:5614-8666
NuwaII-15_CPB	gb|AHGY02088695.1|:1-3622

NuwaII-15_CPB	gb|AHGY02083346.1|:74788-77079
NuwaII-15_CPB	gb|AHGY02081210.1|:1069-5220
NuwaII-15_CPB	gb|AHGY02020973.1|:7834-9635
NuwaII-15_CPB	gb|AHGY02020973.1|:50913-55139
NuwaII-15_CPB	gb|AHGY02002949.1|:7954-12097
NuwaII-15_CPB	gb|AHGY02213579.1|:3-3902
NuwaII-15_CPB	gb|AHGY02097469.1|:10505-14308
NuwaII-15_CPB	gb|AHGY02077288.1|:38883-41903
NuwaII-15_CPB	gb|AHGY02023322.1|:23756-27907
NuwaII-15_CPB	gb|AHGY02006825.1|:10123-12919
NuwaII-15_CPB	gb|AHGY02033333.1|:1-3599
NuwaII-15_CPB	gb|AHGY02029498.1|:12239-15061
NuwaII-15_CPB	gb|AHGY02017003.1|:9142-13276
NuwaII-15_CPB	gb|AHGY02097044.1|:515-3752
NuwaII-15_CPB	gb|AHGY02234999.1|:45-2607
NuwaII-15_CPB	gb|AHGY02197989.1|:33-2254
NuwaII-15_CPB	gb|AHGY02103494.1|:75-3639
NuwaII-15_CPB	gb|AHGY02083173.1|:1-3422
NuwaII-15_CPB	gb|AHGY02082329.1|:6462-9088
NuwaII-15_CPB	gb|AHGY02222486.1|:16-2984
NuwaII-15_CPB	gb|AHGY02101434.1|:1-1984
NuwaII-15_CPB	gb|AHGY02030013.1|:5555-9634
NuwaII-15_CPB	gb|AHGY02201716.1|:10653-14764
NuwaII-15_CPB	gb|AHGY02134586.1|:10-1506
NuwaII-15_CPB	gb|AHGY02038317.1|:62-3537
NuwaII-15_CPB	gb|AHGY02208651.1|:2-1764
NuwaII-15_CPB	gb|AHGY02089970.1|:9751-13875
NuwaII-15_CPB	gb|AHGY02013187.1|:1-3198
NuwaII-15_CPB	gb|AHGY02012660.1|:1-1773
NuwaII-15_CPB	gb|AHGY02047817.1|:27-2634
NuwaII-15_CPB	gb|AHGY02206063.1|:1-3073
NuwaII-15_CPB	gb|AHGY02106648.1|:64-3214
NuwaII-15_CPB	gb|AHGY02074467.1|:24517-28587
NuwaII-15_CPB	gb|AHGY02257479.1|:2-3138
NuwaII-15_CPB	gb|AHGY02256516.1|:1-1513
NuwaII-15_CPB	gb|AHGY02227529.1|:144-2424
NuwaII-15_CPB	gb|AHGY02207643.1|:20-1705
NuwaII-15_CPB	gb|AHGY02088514.1|:1-2129
NuwaII-15_CPB	gb|AHGY02098962.1|:16-2462
NuwaII-15_CPB	gb|AHGY02084729.1|:300-4342
NuwaII-15_CPB	gb|AHGY02238037.1|:1724-5771
NuwaII-15_CPB	gb|AHGY02234151.1|:68-3693
NuwaII-15_CPB	gb|AHGY02130392.1|:50-1965
NuwaII-15_CPB	gb|AHGY02076327.1|:15031-19064

NuwaII-15_CPB	gb|AHGY02082582.1|:1-1989
NuwaII-15_CPB	gb|AHGY02069832.1|:43039-45541
NuwaII-15_CPB	gb|AHGY02012831.1|:9710-12939
NuwaII-15_CPB	gb|AHGY02262256.1|:1-2487
NuwaII-15_CPB	gb|AHGY02201579.1|:88-2401
NuwaII-15_CPB	gb|AHGY02197133.1|:1-3815
NuwaII-15_CPB	gb|AHGY02028483.1|:6750-10737
NuwaII-15_CPB	gb|AHGY02246969.1|:531-2937
NuwaII-15_CPB	gb|AHGY02206523.1|:33549-35091
NuwaII-15_CPB	gb|AHGY02200719.1|:7272-10499
NuwaII-15_CPB	gb|AHGY02175640.1|:9-3036
NuwaII-15_CPB	gb|AHGY02083179.1|:8-3164
NuwaII-15_CPB	gb|AHGY02051414.1|:6741-10858
NuwaII-15_CPB	gb|AHGY02233661.1|:1770-4491
NuwaII-15_CPB	gb|AHGY02080869.1|:60-3466
NuwaII-15_CPB	gb|AHGY02073786.1|:512-3239
NuwaII-15_CPB	gb|AHGY02253796.1|:26-2593
NuwaII-15_CPB	gb|AHGY02198802.1|:1-3307
NuwaII-15_CPB	gb|AHGY02077019.1|:1-2691
NuwaII-15_CPB	gb|AHGY02248193.1|:34-2765
NuwaII-15_CPB	gb|AHGY02133213.1|:1-1621
NuwaII-15_CPB	gb|AHGY02040004.1|:81-3359
NuwaII-15_CPB	gb|AHGY02019326.1|:159-1742
NuwaII-15_CPB	gb|AHGY02251795.1|:33-1372
NuwaII-15_CPB	gb|AHGY02225076.1|:2-2855
NuwaII-15_CPB	gb|AHGY02031893.1|:28552-32100
NuwaII-15_CPB	gb|AHGY02016844.1|:1-4453
NuwaII-15_CPB	gb|AHGY02111498.1|:3410-6094
NuwaII-15_CPB	gb|AHGY02074040.1|:33-2366
NuwaII-15_CPB	gb|AHGY02251753.1|:6607-8971
NuwaII-15_CPB	gb|AHGY02221952.1|:21905-26083
NuwaII-15_CPB	gb|AHGY02207685.1|:59-3232
NuwaII-15_CPB	gb|AHGY02201778.1|:1-3704
NuwaII-15_CPB	gb|AHGY02044589.1|:21016-25179
NuwaII-15_CPB	gb|AHGY02244977.1|:2-2493
NuwaII-15_CPB	gb|AHGY02169580.1|:1-3503
NuwaII-15_CPB	gb|AHGY02064314.1|:19273-22423
NuwaII-15_CPB	gb|AHGY02062138.1|:26595-29110
NuwaII-15_CPB	gb|AHGY02062004.1|:70552-74688
NuwaII-15_CPB	gb|AHGY02062004.1|:40300-44431
NuwaII-15_CPB	gb|AHGY02050133.1|:14802-17177
NuwaII-15_CPB	gb|AHGY02034509.1|:22971-25130
NuwaII-15_CPB	gb|AHGY02222453.1|:12668-16280
NuwaII-15_CPB	gb|AHGY02038045.1|:33779-36176

NuwaII-15_CPB	gb|AHGY02248327.1|:1-3088
NuwaII-15_CPB	gb|AHGY02134211.1|:9-1535
NuwaII-15_CPB	gb|AHGY02060723.1|:37-2475
NuwaII-15_CPB	gb|AHGY02033851.1|:66-1847
NuwaII-15_CPB	gb|AHGY02013161.1|:8323-12446
NuwaII-15_CPB	gb|AHGY02245115.1|:9576-12991
NuwaII-15_CPB	gb|AHGY02224144.1|:20271-24428
NuwaII-15_CPB	gb|AHGY02161929.1|:3-3338
NuwaII-15_CPB	gb|AHGY02111294.1|:3-3333
NuwaII-15_CPB	gb|AHGY02104663.1|:12135-15744
NuwaII-15_CPB	gb|AHGY02253579.1|:2205-6268
NuwaII-15_CPB	gb|AHGY02235893.1|:67-3164
NuwaII-15_CPB	gb|AHGY02235893.1|:27368-29134
NuwaII-15_CPB	gb|AHGY02253428.1|:59240-62816
NuwaII-15_CPB	gb|AHGY02259294.1|:40473-44036
NuwaII-15_CPB	gb|AHGY02135461.1|:13-1437
NuwaII-15_CPB	gb|AHGY02034521.1|:3951-8085
NuwaII-15_CPB	gb|AHGY02034521.1|:1-465
NuwaII-15_CPB	gb|AHGY02238839.1|:1-3069
NuwaII-15_CPB	gb|AHGY02230890.1|:4343-8530
NuwaII-15_CPB	gb|AHGY02068922.1|:3751-6907
NuwaII-15_CPB	gb|AHGY02104204.1|:2453-5845
NuwaII-15_CPB	gb|AHGY02092385.1|:18961-23086
NuwaII-15_CPB	gb|AHGY02249288.1|:50230-54413
NuwaII-15_CPB	gb|AHGY02028612.1|:1-3610
NuwaII-15_CPB	gb|AHGY02026189.1|:9949-13032
NuwaII-15_CPB	gb|AHGY02095500.1|:37914-41375
NuwaII-15_CPB	gb|AHGY02067778.1|:29054-33204
NuwaII-15_CPB	gb|AHGY02064428.1|:35522-38325
NuwaII-15_CPB	gb|AHGY02011805.1|:4419-8555
NuwaII-15_CPB	gb|AHGY02208090.1|:304-4471
NuwaII-15_CPB	gb|AHGY02106490.1|:5917-9464
NuwaII-15_CPB	gb|AHGY02252247.1|:38579-42447
NuwaII-15_CPB	gb|AHGY02133252.1|:31-1624
NuwaII-15_CPB	gb|AHGY02077225.1|:27-2147
NuwaII-15_CPB	gb|AHGY02247536.1|:20391-22709
NuwaII-15_CPB	gb|AHGY02187017.1|:1-2497
NuwaII-15_CPB	gb|AHGY02089276.1|:1195-5209
NuwaII-15_CPB	gb|AHGY02136949.1|:3-1298
NuwaII-15_CPB	gb|AHGY02023095.1|:571-3564
NuwaII-15_CPB	gb|AHGY02220976.1|:1-1480
NuwaII-15_CPB	gb|AHGY02219617.1|:7999-12089
NuwaII-15_CPB	gb|AHGY02201733.1|:3943-8055
NuwaII-15_CPB	gb|AHGY02095686.1|:12071-15584

	NuwaII-15_CPB	gb|AHGY02095686.1|:5171-5566
	NuwaII-15_CPB	gb|AHGY02070019.1|:66-2390
	NuwaII-15_CPB	gb|AHGY02021157.1|:16218-19804
	NuwaII-15_CPB	gb|AHGY02223233.1|:67-3716
	NuwaII-15_CPB	gb|AHGY02198686.1|:63-3066
	NuwaII-15_CPB	gb|AHGY02083322.1|:461-3400
	NuwaII-15_CPB	gb|AHGY02077374.1|:35-2362
Ciona savignyi	*NuwaII_CSav*	gb|AACT01011809.1|:7362-7546
	NuwaII_CSav	gb|AACT01009888.1|:15271-15985
	NuwaII_CSav	gb|AACT01016146.1|:2127-2311
	NuwaII_CSav	gb|AACT01000617.1|:7766-10283
	NuwaII_CSav	gb|AACT01010137.1|:15279-15434
	NuwaII_CSav	gb|AACT01010138.1|:1095-1275
	NuwaII_CSav	gb|AACT01010138.1|:52098-54816
	NuwaII_CSav	gb|AACT01055618.1|:2751-2858
	NuwaII_CSav	gb|AACT01044125.1|:12496-12555
	NuwaII_CSav	gb|AACT01019061.1|:8192-12295
	NuwaII_CSav	gb|AACT01022926.1|:25510-29578
	NuwaII_CSav	gb|AACT01055643.1|:2916-6406
	NuwaII_CSav	gb|AACT01007918.1|:12440-12848
	NuwaII_CSav	gb|AACT01007618.1|:6716-8082
	NuwaII_CSav	gb|AACT01057071.1|:36897-39189
	NuwaII_CSav	gb|AACT01021080.1|:4246-4851
	NuwaII_CSav	gb|AACT01005279.1|:5514-5977
	NuwaII_CSav	gb|AACT01064773.1|:2065-3858
	NuwaII_CSav	gb|AACT01000039.1|:285-1490
	NuwaII_CSav	gb|AACT01018712.1|:46004-48185
	NuwaII_CSav	gb|AACT01059875.1|:29181-29313
	NuwaII_CSav	gb|AACT01039155.1|:3036-8417
	NuwaII_CSav	gb|AACT01001228.1|:4616-9997
Citrus clementina	*NuwaII_CCle*	gb|AMZM01000178.1|:93495-97304
	NuwaII_CCle	gb|AMZM01001971.1|:41119-44861
	NuwaII_CCle	gb|AMZM01004101.1|:100415-104143
	NuwaII_CCle	gb|AMZM01004721.1|:40192-50694
	NuwaII_CCle	gb|AMZM01000002.1|:39196-40054
	NuwaII_CCle	gb|AMZM01003139.1|:891-1527
	NuwaII_CCle	gb|AMZM01001992.1|:27331-28001
	NuwaII_CCle	gb|AMZM01002645.1|:53481-54142
	NuwaII_CCle	gb|AMZM01000973.1|:80656-81310
	NuwaII_CCle	gb|AMZM01003793.1|:197599-198255
	NuwaII_CCle	gb|AMZM01002679.1|:28484-29144
	NuwaII_CCle	gb|AMZM01002198.1|:7989-8648
	NuwaII_CCle	gb|AMZM01002066.1|:200-846
	NuwaII_CCle	gb|AMZM01002793.1|:913-1569

NuwaII_CCle	gb|AMZM01004748.1|:90546-91083
NuwaII_CCle	gb|AMZM01003146.1|:25186-25721
NuwaII_CCle	gb|AMZM01003845.1|:242761-243296
NuwaII_CCle	gb|AMZM01001040.1|:315370-315905
NuwaII_CCle	gb|AMZM01003930.1|:161489-162023
NuwaII_CCle	gb|AMZM01001280.1|:1440-1974
NuwaII_CCle	gb|AMZM01002233.1|:8647-9181
NuwaII_CCle	gb|AMZM01002520.1|:19773-20306
NuwaII_CCle	gb|AMZM01003135.1|:17462-17995
NuwaII_CCle	gb|AMZM01001791.1|:14366-14899
NuwaII_CCle	gb|AMZM01001069.1|:35124-35658
NuwaII_CCle	gb|AMZM01004205.1|:68148-68684
NuwaII_CCle	gb|AMZM01003189.1|:37019-37550
NuwaII_CCle	gb|AMZM01003286.1|:73734-74268
NuwaII_CCle	gb|AMZM01004613.1|:33288-33821
NuwaII_CCle	gb|AMZM01002351.1|:170934-171469
NuwaII_CCle	gb|AMZM01004595.1|:29455-29991
NuwaII_CCle	gb|AMZM01000632.1|:74732-75267
NuwaII_CCle	gb|AMZM01002279.1|:53431-53962
NuwaII_CCle	gb|AMZM01004502.1|:10750-11285
NuwaII_CCle	gb|AMZM01003206.1|:68721-69257
NuwaII_CCle	gb|AMZM01003739.1|:160722-161257
NuwaII_CCle	gb|AMZM01000429.1|:97256-97788
NuwaII_CCle	gb|AMZM01002259.1|:25703-26238
NuwaII_CCle	gb|AMZM01000424.1|:200367-200901
NuwaII_CCle	gb|AMZM01000888.1|:42152-42685
NuwaII_CCle	gb|AMZM01004825.1|:11921-12454
NuwaII_CCle	gb|AMZM01005042.1|:36595-37130
NuwaII_CCle	gb|AMZM01000667.1|:48853-49386
NuwaII_CCle	gb|AMZM01003737.1|:384977-385511
NuwaII_CCle	gb|AMZM01002795.1|:130803-131337
NuwaII_CCle	gb|AMZM01002645.1|:10974-11509
NuwaII_CCle	gb|AMZM01001040.1|:402181-402715
NuwaII_CCle	gb|AMZM01004185.1|:3667-4200
NuwaII_CCle	gb|AMZM01004844.1|:53238-53772
NuwaII_CCle	gb|AMZM01004602.1|:139716-140250
NuwaII_CCle	gb|AMZM01003023.1|:36167-36702
NuwaII_CCle	gb|AMZM01001247.1|:114327-114861
NuwaII_CCle	gb|AMZM01003608.1|:474-1008
NuwaII_CCle	gb|AMZM01003691.1|:68201-68732
NuwaII_CCle	gb|AMZM01002103.1|:33571-34107
NuwaII_CCle	gb|AMZM01004613.1|:209975-210509
NuwaII_CCle	gb|AMZM01004603.1|:115105-115641
NuwaII_CCle	gb|AMZM01000360.1|:114037-114571

NuwaII_CCle	gb|AMZM01002378.1|:21189-21722
NuwaII_CCle	gb|AMZM01003869.1|:104382-104916
NuwaII_CCle	gb|AMZM01004746.1|:26088-26623
NuwaII_CCle	gb|AMZM01000944.1|:68527-69061
NuwaII_CCle	gb|AMZM01005228.1|:187457-187988
NuwaII_CCle	gb|AMZM01000466.1|:90438-90970
NuwaII_CCle	gb|AMZM01000981.1|:75814-76347
NuwaII_CCle	gb|AMZM01000402.1|:49259-49792
NuwaII_CCle	gb|AMZM01004250.1|:9433-9965
NuwaII_CCle	gb|AMZM01001178.1|:28247-28779
NuwaII_CCle	gb|AMZM01002412.1|:16048-16581
NuwaII_CCle	gb|AMZM01001942.1|:188673-189205
NuwaII_CCle	gb|AMZM01003240.1|:188562-189095
NuwaII_CCle	gb|AMZM01003895.1|:234053-234585
NuwaII_CCle	gb|AMZM01001966.1|:413716-414252
NuwaII_CCle	gb|AMZM01002570.1|:81422-81957
NuwaII_CCle	gb|AMZM01000273.1|:138246-138784
NuwaII_CCle	gb|AMZM01001739.1|:53378-53910
NuwaII_CCle	gb|AMZM01002799.1|:129302-130502
NuwaII_CCle	gb|AMZM01002370.1|:45668-46200
NuwaII_CCle	gb|AMZM01003852.1|:369-900
NuwaII_CCle	gb|AMZM01001139.1|:59432-59959
NuwaII_CCle	gb|AMZM01000446.1|:274963-275499
NuwaII_CCle	gb|AMZM01004131.1|:46550-47083
NuwaII_CCle	gb|AMZM01003230.1|:48409-48938
NuwaII_CCle	gb|AMZM01004292.1|:74513-75046
NuwaII_CCle	gb|AMZM01002314.1|:97252-97781
NuwaII_CCle	gb|AMZM01001127.1|:10302-10835
NuwaII_CCle	gb|AMZM01000428.1|:555-1073
NuwaII_CCle	gb|AMZM01004672.1|:167354-167880
NuwaII_CCle	gb|AMZM01003845.1|:334943-335477
NuwaII_CCle	gb|AMZM01003755.1|:221769-222306
NuwaII_CCle	gb|AMZM01000935.1|:40461-40995
NuwaII_CCle	gb|AMZM01001718.1|:9569-10079
NuwaII_CCle	gb|AMZM01001954.1|:42855-43387
NuwaII_CCle	gb|AMZM01000004.1|:112928-113443
NuwaII_CCle	gb|AMZM01003229.1|:42551-43053
NuwaII_CCle	gb|AMZM01004336.1|:42314-42844
NuwaII_CCle	gb|AMZM01003746.1|:64044-64581
NuwaII_CCle	gb|AMZM01003714.1|:49660-50199
NuwaII_CCle	gb|AMZM01003940.1|:237584-238122
NuwaII_CCle	gb|AMZM01003324.1|:53593-54122
NuwaII_CCle	gb|AMZM01004242.1|:6095-6632
NuwaII_CCle	gb|AMZM01000406.1|:33312-33846

NuwaII_CCle	gb|AMZM01003956.1|:41822-42348
NuwaII_CCle	gb|AMZM01003180.1|:5418-6139
NuwaII_CCle	gb|AMZM01004687.1|:21419-21954
NuwaII_CCle	gb|AMZM01003897.1|:169233-169764
NuwaII_CCle	gb|AMZM01001072.1|:173911-174445
NuwaII_CCle	gb|AMZM01003138.1|:1081-1604
NuwaII_CCle	gb|AMZM01001082.1|:115616-116147
NuwaII_CCle	gb|AMZM01005216.1|:75254-75787
NuwaII_CCle	gb|AMZM01000402.1|:35851-36379
NuwaII_CCle	gb|AMZM01000620.1|:87853-88378
NuwaII_CCle	gb|AMZM01004266.1|:23827-24344
NuwaII_CCle	gb|AMZM01001591.1|:122896-123449
NuwaII_CCle	gb|AMZM01000722.1|:248463-248998
NuwaII_CCle	gb|AMZM01003232.1|:6209-6737
NuwaII_CCle	gb|AMZM01004069.1|:57148-57677
NuwaII_CCle	gb|AMZM01001748.1|:129201-129644
NuwaII_CCle	gb|AMZM01001790.1|:19426-19961
NuwaII_CCle	gb|AMZM01003026.1|:85241-85764
NuwaII_CCle	gb|AMZM01000357.1|:151242-151685
NuwaII_CCle	gb|AMZM01000857.1|:37644-38176
NuwaII_CCle	gb|AMZM01002003.1|:42838-43375
NuwaII_CCle	gb|AMZM01004788.1|:1764-2296
NuwaII_CCle	gb|AMZM01001247.1|:70930-71441
NuwaII_CCle	gb|AMZM01004217.1|:88424-91115
NuwaII_CCle	gb|AMZM01004600.1|:6823-7354
NuwaII_CCle	gb|AMZM01002018.1|:154545-154982
NuwaII_CCle	gb|AMZM01004251.1|:123860-124358
NuwaII_CCle	gb|AMZM01001998.1|:26534-27032
NuwaII_CCle	gb|AMZM01004602.1|:26452-26980
NuwaII_CCle	gb|AMZM01005135.1|:44602-45130
NuwaII_CCle	gb|AMZM01003026.1|:35580-36018
NuwaII_CCle	gb|AMZM01001662.1|:1703-2121
NuwaII_CCle	gb|AMZM01001422.1|:8004-8530
NuwaII_CCle	gb|AMZM01000053.1|:84354-85401
NuwaII_CCle	gb|AMZM01007384.1|:34-290
NuwaII_CCle	gb|AMZM01003124.1|:45426-45681
NuwaII_CCle	gb|AMZM01005300.1|:5723-5946
NuwaII_CCle	gb|AMZM01005298.1|:18636-18859
NuwaII_CCle	gb|AMZM01001966.1|:135804-136073
NuwaII_CCle	gb|AMZM01000666.1|:221526-221698
NuwaII_CCle	gb|AMZM01002645.1|:33317-34998
NuwaII_CCle	gb|AMZM01000132.1|:115058-115217
NuwaII_CCle	gb|AMZM01003845.1|:75387-75504
NuwaII_CCle	gb|AMZM01002351.1|:32299-32412

NuwaII_CCle	gb|AMZM01004672.1|:31497-31607
NuwaII_CCle	gb|AMZM01003895.1|:104704-104784
NuwaII_CCle	gb|AMZM01002445.1|:64895-64973
NuwaII_CCle	gb|AMZM01002445.1|:75725-75803
NuwaII_CCle	gb|AMZM01004556.1|:142152-142229
NuwaII_CCle	gb|AMZM01001040.1|:9072-9148
NuwaII_CCle	gb|AMZM01004205.1|:132814-132875
NuwaII_CCle	gb|AMZM01000053.1|:10666-10715
NuwaII_CCle	gb|AMZM01003661.1|:11640-15359
NuwaII_CCle	gb|AMZM01000148.1|:28178-33948
NuwaII_CCle	gb|AMZM01003273.1|:13276-14026
NuwaII_CCle	gb|AMZM01004171.1|:95843-96584
NuwaII_CCle	gb|AMZM01000944.1|:31484-32482
NuwaII_CCle	gb|AMZM01002403.1|:23818-24482
NuwaII_CCle	gb|AMZM01000305.1|:412-1068
NuwaII_CCle	gb|AMZM01003020.1|:33591-34228
NuwaII_CCle	gb|AMZM01000148.1|:8936-9720
NuwaII_CCle	gb|AMZM01004008.1|:135226-135888
NuwaII_CCle	gb|AMZM01003150.1|:27977-28640
NuwaII_CCle	gb|AMZM01003307.1|:107887-108545
NuwaII_CCle	gb|AMZM01001180.1|:13086-13747
NuwaII_CCle	gb|AMZM01001966.1|:133685-134312
NuwaII_CCle	gb|AMZM01003846.1|:1320-1855
NuwaII_CCle	gb|AMZM01001142.1|:509-1045
NuwaII_CCle	gb|AMZM01003177.1|:61422-61957
NuwaII_CCle	gb|AMZM01005263.1|:77767-78302
NuwaII_CCle	gb|AMZM01003001.1|:2443-2977
NuwaII_CCle	gb|AMZM01003198.1|:1849-2384
NuwaII_CCle	gb|AMZM01000822.1|:53211-53747
NuwaII_CCle	gb|AMZM01002639.1|:23876-24407
NuwaII_CCle	gb|AMZM01003862.1|:23997-24530
NuwaII_CCle	gb|AMZM01001799.1|:4540-5074
NuwaII_CCle	gb|AMZM01001119.1|:28314-28846
NuwaII_CCle	gb|AMZM01001591.1|:138185-138719
NuwaII_CCle	gb|AMZM01003659.1|:250306-250840
NuwaII_CCle	gb|AMZM01000466.1|:110763-111298
NuwaII_CCle	gb|AMZM01002595.1|:84555-85089
NuwaII_CCle	gb|AMZM01001738.1|:66001-66538
NuwaII_CCle	gb|AMZM01001040.1|:272300-272834
NuwaII_CCle	gb|AMZM01001870.1|:4567-5100
NuwaII_CCle	gb|AMZM01001759.1|:7868-8401
NuwaII_CCle	gb|AMZM01002948.1|:12346-12879
NuwaII_CCle	gb|AMZM01002132.1|:4624-5159
NuwaII_CCle	gb|AMZM01002039.1|:11826-12356

NuwaII_CCle	gb|AMZM01001992.1|:61206-61740
NuwaII_CCle	gb|AMZM01002445.1|:16421-16952
NuwaII_CCle	gb|AMZM01000445.1|:190543-191076
NuwaII_CCle	gb|AMZM01004775.1|:9980-10515
NuwaII_CCle	gb|AMZM01001531.1|:1814-2347
NuwaII_CCle	gb|AMZM01000822.1|:76158-76691
NuwaII_CCle	gb|AMZM01003900.1|:69228-69759
NuwaII_CCle	gb|AMZM01002045.1|:13598-14136
NuwaII_CCle	gb|AMZM01001237.1|:7577-8111
NuwaII_CCle	gb|AMZM01003177.1|:93754-94282
NuwaII_CCle	gb|AMZM01002351.1|:68121-68647
NuwaII_CCle	gb|AMZM01004593.1|:7636-8173
NuwaII_CCle	gb|AMZM01003725.1|:33141-33676
NuwaII_CCle	gb|AMZM01003147.1|:16806-17341
NuwaII_CCle	gb|AMZM01004276.1|:38095-38630
NuwaII_CCle	gb|AMZM01003056.1|:124626-125160
NuwaII_CCle	gb|AMZM01004337.1|:24272-24805
NuwaII_CCle	gb|AMZM01003759.1|:145810-146344
NuwaII_CCle	gb|AMZM01002273.1|:297716-298251
NuwaII_CCle	gb|AMZM01003465.1|:24105-24638
NuwaII_CCle	gb|AMZM01001898.1|:44893-45428
NuwaII_CCle	gb|AMZM01003846.1|:44091-44624
NuwaII_CCle	gb|AMZM01001951.1|:12060-12595
NuwaII_CCle	gb|AMZM01000357.1|:196528-197062
NuwaII_CCle	gb|AMZM01002941.1|:1162-1696
NuwaII_CCle	gb|AMZM01001872.1|:8492-9032
NuwaII_CCle	gb|AMZM01002486.1|:65044-65580
NuwaII_CCle	gb|AMZM01001720.1|:3133-3665
NuwaII_CCle	gb|AMZM01002369.1|:15525-16059
NuwaII_CCle	gb|AMZM01005009.1|:21953-22490
NuwaII_CCle	gb|AMZM01004221.1|:7986-8520
NuwaII_CCle	gb|AMZM01005156.1|:56949-57480
NuwaII_CCle	gb|AMZM01004748.1|:51425-51950
NuwaII_CCle	gb|AMZM01004224.1|:54785-55317
NuwaII_CCle	gb|AMZM01000431.1|:32671-33205
NuwaII_CCle	gb|AMZM01004405.1|:96512-97042
NuwaII_CCle	gb|AMZM01002141.1|:26669-27204
NuwaII_CCle	gb|AMZM01000729.1|:228869-229403
NuwaII_CCle	gb|AMZM01003681.1|:19515-20050
NuwaII_CCle	gb|AMZM01005878.1|:101-634
NuwaII_CCle	gb|AMZM01003303.1|:66990-67522
NuwaII_CCle	gb|AMZM01002812.1|:104996-105528
NuwaII_CCle	gb|AMZM01004148.1|:3089-3621
NuwaII_CCle	gb|AMZM01001034.1|:87145-87676

NuwaII_CCle	gb|AMZM01003897.1|:289317-289848
NuwaII_CCle	gb|AMZM01002289.1|:100456-100988
NuwaII_CCle	gb|AMZM01001442.1|:27145-27674
NuwaII_CCle	gb|AMZM01001887.1|:218314-218847
NuwaII_CCle	gb|AMZM01003896.1|:196408-196941
NuwaII_CCle	gb|AMZM01002473.1|:31982-32495
NuwaII_CCle	gb|AMZM01002854.1|:137428-137959
NuwaII_CCle	gb|AMZM01000053.1|:84469-85002
NuwaII_CCle	gb|AMZM01001887.1|:108300-108829
NuwaII_CCle	gb|AMZM01004946.1|:66753-67284
NuwaII_CCle	gb|AMZM01000487.1|:92190-92722
NuwaII_CCle	gb|AMZM01003241.1|:105743-106279
NuwaII_CCle	gb|AMZM01001085.1|:318606-319138
NuwaII_CCle	gb|AMZM01002015.1|:39967-40509
NuwaII_CCle	gb|AMZM01000132.1|:145714-146241
NuwaII_CCle	gb|AMZM01000657.1|:13314-13845
NuwaII_CCle	gb|AMZM01002018.1|:134250-134778
NuwaII_CCle	gb|AMZM01001471.1|:72262-72776
NuwaII_CCle	gb|AMZM01000970.1|:135041-135575
NuwaII_CCle	gb|AMZM01005133.1|:65361-65878
NuwaII_CCle	gb|AMZM01002834.1|:7518-8047
NuwaII_CCle	gb|AMZM01000051.1|:340-873
NuwaII_CCle	gb|AMZM01001782.1|:30867-31285
NuwaII_CCle	gb|AMZM01001893.1|:26346-26856
NuwaII_CCle	gb|AMZM01000620.1|:57805-58325
NuwaII_CCle	gb|AMZM01004700.1|:157570-158097
NuwaII_CCle	gb|AMZM01000017.1|:96076-96605
NuwaII_CCle	gb|AMZM01004672.1|:20113-20647
NuwaII_CCle	gb|AMZM01000002.1|:42930-43460
NuwaII_CCle	gb|AMZM01001736.1|:44913-45360
NuwaII_CCle	gb|AMZM01001468.1|:109756-110205
NuwaII_CCle	gb|AMZM01001727.1|:18850-19366
NuwaII_CCle	gb|AMZM01004038.1|:30928-31460
NuwaII_CCle	gb|AMZM01002375.1|:78723-79166
NuwaII_CCle	gb|AMZM01000709.1|:59196-59639
NuwaII_CCle	gb|AMZM01002213.1|:64721-65254
NuwaII_CCle	gb|AMZM01004556.1|:58729-59267
NuwaII_CCle	gb|AMZM01004278.1|:86134-86637
NuwaII_CCle	gb|AMZM01004491.1|:29794-30328
NuwaII_CCle	gb|AMZM01000432.1|:15779-16289
NuwaII_CCle	gb|AMZM01003268.1|:12785-13229
NuwaII_CCle	gb|AMZM01002100.1|:14622-15130
NuwaII_CCle	gb|AMZM01002485.1|:9480-10008
NuwaII_CCle	gb|AMZM01003793.1|:99861-100398

	NuwaII_CCle	gb|AMZM01004603.1|:475251-475786
	NuwaII_CCle	gb|AMZM01000655.1|:9071-9584
	NuwaII_CCle	gb|AMZM01002150.1|:1979-2482
	NuwaII_CCle	gb|AMZM01003257.1|:181665-182202
	NuwaII_CCle	gb|AMZM01004195.1|:183763-184265
	NuwaII_CCle	gb|AMZM01000445.1|:389001-389527
	NuwaII_CCle	gb|AMZM01005169.1|:12256-12755
	NuwaII_CCle	gb|AMZM01005106.1|:268200-268736
	NuwaII_CCle	gb|AMZM01002714.1|:21-504
	NuwaII_CCle	gb|AMZM01001908.1|:50701-51233
	NuwaII_CCle	gb|AMZM01000688.1|:205619-206108
	NuwaII_CCle	gb|AMZM01004248.1|:14865-15328
	NuwaII_CCle	gb|AMZM01001992.1|:26880-27255
	NuwaII_CCle	gb|AMZM01000666.1|:221034-221456
	NuwaII_CCle	gb|AMZM01003961.1|:23434-23698
	NuwaII_CCle	gb|AMZM01002503.1|:43694-43949
	NuwaII_CCle	gb|AMZM01004302.1|:19199-19453
	NuwaII_CCle	gb|AMZM01002630.1|:16847-17097
	NuwaII_CCle	gb|AMZM01001759.1|:23702-23936
	NuwaII_CCle	gb|AMZM01004672.1|:94741-94906
	NuwaII_CCle	gb|AMZM01004788.1|:1544-1689
	NuwaII_CCle	gb|AMZM01004603.1|:133229-134773
	NuwaII_CCle	gb|AMZM01001085.1|:277249-277366
	NuwaII_CCle	gb|AMZM01001247.1|:138367-138478
	NuwaII_CCle	gb|AMZM01004251.1|:73870-73966
	NuwaII_CCle	gb|AMZM01003895.1|:351324-351768
	NuwaII_CCle	gb|AMZM01001748.1|:295417-295495
	NuwaII_CCle	gb|AMZM01002799.1|:163688-163759
	NuwaII_CCle	gb|AMZM01004602.1|:26497-26557
	NuwaII_CCle	gb|AMZM01003895.1|:129328-129382
	NuwaII_CCle	gb|AMZM01004603.1|:117489-117542
Vitis vinifera	*NuwaII_VVin*	emb|CAAP03009629.1|:95016-89121
	NuwaII_VVin	emb|CAAP03010027.1|:106908-112692
	NuwaII_VVin	emb|CAAP03011183.1|:8111-13897
	NuwaII_VVin	emb|CAAP03014288.1|:14494-16070
	NuwaII_VVin	emb|CAAP03002240.1|:66634-68996
	NuwaII_VVin	emb|CAAP03003527.1|:6204-9868
	NuwaII_VVin	emb|CAAP03009123.1|:56749-58943
	NuwaII_VVin	emb|CAAP03011736.1|:173808-175002
	NuwaII_VVin	emb|CAAP03007432.1|:4346-5139
	NuwaII_VVin	emb|CAAP03006142.1|:76077-83464
	NuwaII_VVin	emb|CAAP03004598.1|:20033-21795
	NuwaII_VVin	emb|CAAP03009953.1|:75072-77100
	NuwaII_VVin	emb|CAAP03004445.1|:35466-39505

NuwaII_VVin	emb|CAAP03009164.1|:25487-34491
NuwaII_VVin	emb|CAAP03010027.1|:1-4824
NuwaII_VVin	emb|CAAP03009961.1|:25121-29711
NuwaII_VVin	emb|CAAP03011290.1|:9118-12177
NuwaII_VVin	emb|CAAP03007823.1|:105963-108681
NuwaII_VVin	emb|CAAP03010091.1|:3320-6293
NuwaII_VVin	emb|CAAP03001819.1|:60068-63093
NuwaII_VVin	emb|CAAP03009390.1|:45750-50244
NuwaII_VVin	emb|CAAP03011695.1|:102805-105787
NuwaII_VVin	emb|CAAP03011183.1|:31855-34765
NuwaII_VVin	emb|CAAP03011667.1|:129732-132753
NuwaII_VVin	emb|CAAP03005681.1|:37916-42470
NuwaII_VVin	emb|CAAP03010297.1|:95631-98227
NuwaII_VVin	emb|CAAP03014135.1|:4486-6996
NuwaII_VVin	emb|CAAP03012147.1|:40925-42019
NuwaII_VVin	emb|CAAP03009010.1|:139553-141841
NuwaII_VVin	emb|CAAP03004352.1|:233011-235055
NuwaII_VVin	emb|CAAP03008670.1|:116011-117189
NuwaII_VVin	emb|CAAP03000041.1|:222111-223025
NuwaII_VVin	emb|CAAP03006722.1|:62756-63747
NuwaII_VVin	emb|CAAP03010871.1|:10086-10956
NuwaII_VVin	emb|CAAP03009879.1|:15172-16028
NuwaII_VVin	emb|CAAP03011039.1|:28214-29693
NuwaII_VVin	emb|CAAP03004350.1|:86836-87460
NuwaII_VVin	emb|CAAP03008089.1|:128258-128867
NuwaII_VVin	emb|CAAP03001181.1|:11900-12449
NuwaII_VVin	emb|CAAP03013952.1|:84063-84648
NuwaII_VVin	emb|CAAP03005331.1|:16965-17543
NuwaII_VVin	emb|CAAP03007727.1|:16898-17556
NuwaII_VVin	emb|CAAP03012578.1|:29187-29678
NuwaII_VVin	emb|CAAP03006827.1|:153880-154470
NuwaII_VVin	emb|CAAP03013826.1|:118250-118802
NuwaII_VVin	emb|CAAP03004086.1|:41369-41822
NuwaII_VVin	emb|CAAP03001000.1|:46929-47315
NuwaII_VVin	emb|CAAP03013368.1|:61358-62027
NuwaII_VVin	emb|CAAP03002808.1|:30330-30651
NuwaII_VVin	emb|CAAP03012326.1|:112380-112910
NuwaII_VVin	emb|CAAP03007212.1|:146281-146765
NuwaII_VVin	emb|CAAP03000494.1|:47015-47540
NuwaII_VVin	emb|CAAP03010262.1|:10005-10484
NuwaII_VVin	emb|CAAP03003888.1|:52063-52314
NuwaII_VVin	emb|CAAP03001286.1|:42793-43041
NuwaII_VVin	emb|CAAP03008833.1|:136376-136623
NuwaII_VVin	emb|CAAP03002450.1|:195945-196279

NuwaII_VVin	emb|CAAP03005263.1|:412-587
NuwaII_VVin	emb|CAAP03012886.1|:72438-72571
NuwaII_VVin	emb|CAAP03009989.1|:42921-43052
NuwaII_VVin	emb|CAAP03014378.1|:135944-136138
NuwaII_VVin	emb|CAAP03000275.1|:110334-110458
NuwaII_VVin	emb|CAAP03000176.1|:60096-60183
NuwaII_VVin	emb|CAAP03002374.1|:79534-79614
NuwaII_VVin	emb|CAAP03005843.1|:17763-17836
NuwaII_VVin	emb|CAAP03004373.1|:97884-97949
NuwaII_VVin	emb|CAAP03005739.1|:22204-22268
NuwaII_VVin	emb|CAAP03005594.1|:32640-32699
NuwaII_VVin	emb|CAAP03014656.1|:107074-113036
NuwaII_VVin	emb|CAAP03002476.1|:4536-5817
NuwaII_VVin	emb|CAAP03000054.1|:38031-39079
NuwaII_VVin	emb|CAAP03001968.1|:6711-13037
NuwaII_VVin	emb|CAAP03003290.1|:50800-56907
NuwaII_VVin	emb|CAAP03002670.1|:3413-6905
NuwaII_VVin	emb|CAAP03006231.1|:95573-95995
NuwaII_VVin	emb|CAAP03006198.1|:19999-22015
NuwaII_VVin	emb|CAAP03003082.1|:81386-87206
NuwaII_VVin	emb|CAAP03003774.1|:21733-22255
NuwaII_VVin	emb|CAAP03001959.1|:30594-32963
NuwaII_VVin	emb|CAAP03006052.1|:50269-53339
NuwaII_VVin	emb|CAAP03002444.1|:100737-103767
NuwaII_VVin	emb|CAAP03005534.1|:6031-8833
NuwaII_VVin	emb|CAAP03003084.1|:113063-113610
NuwaII_VVin	emb|CAAP03001817.1|:12650-15779
NuwaII_VVin	emb|CAAP03006261.1|:3868-6617
NuwaII_VVin	emb|CAAP03013303.1|:6217-8965
NuwaII_VVin	emb|CAAP03013335.1|:2139-3034
NuwaII_VVin	emb|CAAP03004798.1|:114805-117569
NuwaII_VVin	emb|CAAP03006554.1|:18063-20841
NuwaII_VVin	emb|CAAP03008496.1|:166350-168467
NuwaII_VVin	emb|CAAP03009961.1|:18270-20282
NuwaII_VVin	emb|CAAP03009068.1|:16407-19896
NuwaII_VVin	emb|CAAP03003050.1|:27122-28849
NuwaII_VVin	emb|CAAP03001959.1|:44496-46427
NuwaII_VVin	emb|CAAP03011686.1|:76757-78261
NuwaII_VVin	emb|CAAP03001487.1|:73447-74753
NuwaII_VVin	emb|CAAP03007727.1|:60580-61738
NuwaII_VVin	emb|CAAP03004354.1|:67966-68937
NuwaII_VVin	emb|CAAP03009493.1|:7501-8314
NuwaII_VVin	emb|CAAP03007452.1|:128589-130157
NuwaII_VVin	emb|CAAP03012702.1|:18870-19543

	NuwaII_VVin	emb|CAAP03005069.1|:16651-17148
	NuwaII_VVin	emb|CAAP03002207.1|:79627-80355
	NuwaII_VVin	emb|CAAP03001964.1|:4714-4900
	NuwaII_VVin	emb|CAAP03011573.1|:40608-40966
	NuwaII_VVin	emb|CAAP03010926.1|:241877-242292
	NuwaII_VVin	emb|CAAP03001620.1|:49893-50202
	NuwaII_VVin	emb|CAAP03010467.1|:88770-89248
	NuwaII_VVin	emb|CAAP03001257.1|:67909-68438
	NuwaII_VVin	emb|CAAP03009699.1|:124014-124538
	NuwaII_VVin	emb|CAAP03005480.1|:38386-38875
	NuwaII_VVin	emb|CAAP03000632.1|:6160-6406
	NuwaII_VVin	emb|CAAP03011937.1|:4845-5087
	NuwaII_VVin	emb|CAAP03003112.1|:46275-46494
	NuwaII_VVin	emb|CAAP03011417.1|:75474-75693
	NuwaII_VVin	emb|CAAP03002271.1|:186634-186810
	NuwaII_VVin	emb|CAAP03002271.1|:158531-158712
	NuwaII_VVin	emb|CAAP03002651.1|:21418-21754
	NuwaII_VVin	emb|CAAP03001800.1|:2496-2832
	NuwaII_VVin	emb|CAAP03006983.1|:107472-107591
	NuwaII_VVin	emb|CAAP03008089.1|:47937-48096
	NuwaII_VVin	emb|CAAP03002579.1|:59247-59390
	NuwaII_VVin	emb|CAAP03004839.1|:9928-10219
	NuwaII_VVin	emb|CAAP03009484.1|:71498-73210
	NuwaII_VVin	emb|CAAP03002569.1|:4701-4833
	NuwaII_VVin	emb|CAAP03001452.1|:4410-4543
	NuwaII_VVin	emb|CAAP03012188.1|:65626-65757
	NuwaII_VVin	emb|CAAP03013616.1|:33772-33871
	NuwaII_VVin	emb|CAAP03011896.1|:124034-124133
	NuwaII_VVin	emb|CAAP03010918.1|:78286-78382
	NuwaII_VVin	emb|CAAP03000347.1|:30432-30526
	NuwaII_VVin	emb|CAAP03013228.1|:92298-92392
	NuwaII_VVin	emb|CAAP03002838.1|:7762-7856
	NuwaII_VVin	emb|CAAP03014378.1|:239845-239923
	NuwaII_VVin	emb|CAAP03012775.1|:73608-73683
	NuwaII_VVin	emb|CAAP03012233.1|:20144-20219
	NuwaII_VVin	emb|CAAP03004103.1|:71272-71347
	NuwaII_VVin	emb|CAAP03008965.1|:37106-37166
	NuwaII_VVin	emb|CAAP03014650.1|:9758-9812
	NuwaII_VVin	emb|CAAP03011931.1|:21451-21505
	NuwaII_VVin	emb|CAAP03010346.1|:35309-35362
	NuwaII_VVin	emb|CAAP03005829.1|:168677-168729
Maylandia zebra	*NuwaII-1_MZeb*	gb|AGTA02049441.1|:8321-10146
	NuwaII-1_MZeb	gb|AGTA02038823.1|:2489-3893
	NuwaII-1_MZeb	gb|AGTA02055764.1|:6385-8179

NuwaII-1_MZeb	gb|AGTA02064340.1|:1338-3124
NuwaII-1_MZeb	gb|AGTA02011159.1|:9268-11063
NuwaII-1_MZeb	gb|AGTA02029877.1|:12-992
NuwaII-1_MZeb	gb|AGTA02046794.1|:2004-5683
NuwaII-1_MZeb	gb|AGTA02056225.1|:4-914
NuwaII-1_MZeb	gb|AGTA02060812.1|:1960-3147
NuwaII-1_MZeb	gb|AGTA02017514.1|:1789-2531
NuwaII-1_MZeb	gb|AGTA02068744.1|:1187-2067
NuwaII-1_MZeb	gb|AGTA02066913.1|:12411-13756
NuwaII-1_MZeb	gb|AGTA02023972.1|:1628-1818
NuwaII-1_MZeb	gb|AGTA02023972.1|:1818-2553
NuwaII-1_MZeb	gb|AGTA02036514.1|:3298-3984
NuwaII-1_MZeb	gb|AGTA02019636.1|:8527-9097
NuwaII-1_MZeb	gb|AGTA02044485.1|:1-596
NuwaII-1_MZeb	gb|AGTA02019560.1|:1-519
NuwaII-1_MZeb	gb|AGTA02028936.1|:19679-20195
NuwaII-1_MZeb	gb|AGTA02022660.1|:27806-28398
NuwaII-1_MZeb	gb|AGTA02065649.1|:505-1674
NuwaII-1_MZeb	gb|AGTA02067980.1|:9067-9545
NuwaII-1_MZeb	gb|AGTA02049471.1|:1-1003
NuwaII-1_MZeb	gb|AGTA02028865.1|:1627-2633
NuwaII-1_MZeb	gb|AGTA02007511.1|:13588-14022
NuwaII-1_MZeb	gb|AGTA02042694.1|:18302-18735
NuwaII-1_MZeb	gb|AGTA02034585.1|:16237-16624
NuwaII-1_MZeb	gb|AGTA02064766.1|:4217-4602
NuwaII-1_MZeb	gb|AGTA02046691.1|:3-445
NuwaII-1_MZeb	gb|AGTA02019558.1|:1-384
NuwaII-1_MZeb	gb|AGTA02067536.1|:1-807
NuwaII-1_MZeb	gb|AGTA02012304.1|:16006-16345
NuwaII-1_MZeb	gb|AGTA02064974.1|:11442-12097
NuwaII-1_MZeb	gb|AGTA02003901.1|:4-368
NuwaII-1_MZeb	gb|AGTA02039641.1|:1656-1945
NuwaII-1_MZeb	gb|AGTA02065198.1|:62509-62815
NuwaII-1_MZeb	gb|AGTA02024266.1|:853-1397
NuwaII-1_MZeb	gb|AGTA02019637.1|:1-362
NuwaII-1_MZeb	gb|AGTA02068308.1|:2988-3411
NuwaII-1_MZeb	gb|AGTA02051896.1|:1-310
NuwaII-1_MZeb	gb|AGTA02049063.1|:1-393
NuwaII-1_MZeb	gb|AGTA02047005.1|:499-1339
NuwaII-1_MZeb	gb|AGTA02008729.1|:1-569
NuwaII-1_MZeb	gb|AGTA02008299.1|:2655-3090
NuwaII-1_MZeb	gb|AGTA02054565.1|:7416-7716
NuwaII-1_MZeb	gb|AGTA02066091.1|:21-309
NuwaII-1_MZeb	gb|AGTA02065199.1|:1-408

NuwaII-1_MZeb	gb|AGTA02057533.1|:7886-8282
NuwaII-1_MZeb	gb|AGTA02033766.1|:1269-1689
NuwaII-1_MZeb	gb|AGTA02011561.1|:10551-10986
NuwaII-1_MZeb	gb|AGTA02009066.1|:29-317
NuwaII-1_MZeb	gb|AGTA02065897.1|:527-1364
NuwaII-1_MZeb	gb|AGTA02059423.1|:1-943
NuwaII-1_MZeb	gb|AGTA02051728.1|:832-1108
NuwaII-1_MZeb	gb|AGTA02057720.1|:2764-3048
NuwaII-1_MZeb	gb|AGTA02028248.1|:14930-15325
NuwaII-1_MZeb	gb|AGTA02069193.1|:2341-2658
NuwaII-1_MZeb	gb|AGTA02064975.1|:1-612
NuwaII-1_MZeb	gb|AGTA02059464.1|:944-1264
NuwaII-1_MZeb	gb|AGTA02046738.1|:38162-38596
NuwaII-1_MZeb	gb|AGTA02036286.1|:3169-4100
NuwaII-1_MZeb	gb|AGTA02023971.1|:6121-6736
NuwaII-1_MZeb	gb|AGTA02052944.1|:3276-4086
NuwaII-1_MZeb	gb|AGTA02030378.1|:1-262
NuwaII-1_MZeb	gb|AGTA02016256.1|:12934-13223
NuwaII-1_MZeb	gb|AGTA02046831.1|:2202-2778
NuwaII-1_MZeb	gb|AGTA02003386.1|:26478-26740
NuwaII-1_MZeb	gb|AGTA02065067.1|:1203-1638
NuwaII-1_MZeb	gb|AGTA02057375.1|:16-759
NuwaII-1_MZeb	gb|AGTA02030153.1|:993-1801
NuwaII-1_MZeb	gb|AGTA02030153.1|:1831-1910
NuwaII-1_MZeb	gb|AGTA02020517.1|:20204-20738
NuwaII-1_MZeb	gb|AGTA02012305.1|:1-410
NuwaII-1_MZeb	gb|AGTA02004752.1|:1-1503
NuwaII-1_MZeb	gb|AGTA02003742.1|:3878-4179
NuwaII-1_MZeb	gb|AGTA02052347.1|:1-300
NuwaII-1_MZeb	gb|AGTA02043841.1|:1-280
NuwaII-1_MZeb	gb|AGTA02068767.1|:7830-8110
NuwaII-1_MZeb	gb|AGTA02047371.1|:1-248
NuwaII-1_MZeb	gb|AGTA02038299.1|:6842-7142
NuwaII-1_MZeb	gb|AGTA02029454.1|:1-237
NuwaII-1_MZeb	gb|AGTA02049062.1|:61910-62161
NuwaII-1_MZeb	gb|AGTA02042917.1|:1-251
NuwaII-1_MZeb	gb|AGTA02014574.1|:1-244
NuwaII-1_MZeb	gb|AGTA02010729.1|:2575-2809
NuwaII-1_MZeb	gb|AGTA02029453.1|:8455-8694
NuwaII-1_MZeb	gb|AGTA02051895.1|:9313-9538
NuwaII-1_MZeb	gb|AGTA02018328.1|:14971-15204
NuwaII-1_MZeb	gb|AGTA02006654.1|:47342-47578
NuwaII-1_MZeb	gb|AGTA02026674.1|:11785-12012
NuwaII-1_MZeb	gb|AGTA02051526.1|:1196-1421

NuwaII-1_MZeb	gb|AGTA02050185.1|:6567-6783
NuwaII-1_MZeb	gb|AGTA02044113.1|:1-220
NuwaII-1_MZeb	gb|AGTA02057374.1|:6760-6991
NuwaII-1_MZeb	gb|AGTA02069902.1|:1-250
NuwaII-1_MZeb	gb|AGTA02063692.1|:1053-1275
NuwaII-1_MZeb	gb|AGTA02068341.1|:6540-6764
NuwaII-1_MZeb	gb|AGTA02042441.1|:5394-5593
NuwaII-1_MZeb	gb|AGTA02009214.1|:7444-7664
NuwaII-1_MZeb	gb|AGTA02041276.1|:7356-7572
NuwaII-1_MZeb	gb|AGTA02033730.1|:1-232
NuwaII-1_MZeb	gb|AGTA02003743.1|:1-528
NuwaII-1_MZeb	gb|AGTA02031095.1|:1518-2039
NuwaII-1_MZeb	gb|AGTA02024348.1|:2-224
NuwaII-1_MZeb	gb|AGTA02008300.1|:1-393
NuwaII-1_MZeb	gb|AGTA02007753.1|:60432-60613
NuwaII-1_MZeb	gb|AGTA02004424.1|:366-551
NuwaII-1_MZeb	gb|AGTA02013227.1|:3-186
NuwaII-1_MZeb	gb|AGTA02058841.1|:181-375
NuwaII-1_MZeb	gb|AGTA02055478.1|:1-191
NuwaII-1_MZeb	gb|AGTA02033149.1|:1513-1794
NuwaII-1_MZeb	gb|AGTA02032770.1|:1-169
NuwaII-1_MZeb	gb|AGTA02038050.1|:2539-2956
NuwaII-1_MZeb	gb|AGTA02031495.1|:3-215
NuwaII-1_MZeb	gb|AGTA02029627.1|:1-204
NuwaII-1_MZeb	gb|AGTA02028200.1|:3522-3705
NuwaII-1_MZeb	gb|AGTA02046548.1|:1-195
NuwaII-1_MZeb	gb|AGTA02031017.1|:42753-42930
NuwaII-1_MZeb	gb|AGTA02053690.1|:3117-3296
NuwaII-1_MZeb	gb|AGTA02012855.1|:1-184
NuwaII-1_MZeb	gb|AGTA02042123.1|:3316-3487
NuwaII-1_MZeb	gb|AGTA02036816.1|:1729-1908
NuwaII-1_MZeb	gb|AGTA02038051.1|:1-171
NuwaII-1_MZeb	gb|AGTA02042124.1|:1-165
NuwaII-1_MZeb	gb|AGTA02039183.1|:9414-9591
NuwaII-1_MZeb	gb|AGTA02020616.1|:6-183
NuwaII-1_MZeb	gb|AGTA02060721.1|:9-188
NuwaII-1_MZeb	gb|AGTA02062015.1|:1-165
NuwaII-1_MZeb	gb|AGTA02028419.1|:1-158
NuwaII-1_MZeb	gb|AGTA02065555.1|:1-161
NuwaII-1_MZeb	gb|AGTA02004751.1|:3690-3849
NuwaII-1_MZeb	gb|AGTA02045746.1|:11007-11177
NuwaII-1_MZeb	gb|AGTA02030377.1|:67336-67495
NuwaII-1_MZeb	gb|AGTA02020790.1|:8664-8841
NuwaII-1_MZeb	gb|AGTA02011769.1|:1-157

NuwaII-1_MZeb	gb|AGTA02069975.1|:2546-2729
NuwaII-1_MZeb	gb|AGTA02063848.1|:7-151
NuwaII-1_MZeb	gb|AGTA02053477.1|:1-156
NuwaII-1_MZeb	gb|AGTA02048618.1|:4-184
NuwaII-1_MZeb	gb|AGTA02010721.1|:9205-9360
NuwaII-1_MZeb	gb|AGTA02062267.1|:1-247
NuwaII-1_MZeb	gb|AGTA02042442.1|:1-168
NuwaII-1_MZeb	gb|AGTA02054835.1|:5038-5190
NuwaII-1_MZeb	gb|AGTA02004423.1|:35159-35311
NuwaII-1_MZeb	gb|AGTA02056182.1|:1-156
NuwaII-1_MZeb	gb|AGTA02052564.1|:1325-1472
NuwaII-1_MZeb	gb|AGTA02044805.1|:1-156
NuwaII-1_MZeb	gb|AGTA02003302.1|:30592-30762
NuwaII-1_MZeb	gb|AGTA02057280.1|:2557-2732
NuwaII-1_MZeb	gb|AGTA02031577.1|:6620-6787
NuwaII-1_MZeb	gb|AGTA02007386.1|:1-151
NuwaII-1_MZeb	gb|AGTA02059752.1|:2865-3461
NuwaII-1_MZeb	gb|AGTA02028418.1|:1-172
NuwaII-1_MZeb	gb|AGTA02010727.1|:5338-5491
NuwaII-1_MZeb	gb|AGTA02008483.1|:1-178
NuwaII-1_MZeb	gb|AGTA02069049.1|:1-148
NuwaII-1_MZeb	gb|AGTA02065548.1|:1-149
NuwaII-1_MZeb	gb|AGTA02050051.1|:1330-1478
NuwaII-1_MZeb	gb|AGTA02028937.1|:1-156
NuwaII-1_MZeb	gb|AGTA02018329.1|:1-152
NuwaII-1_MZeb	gb|AGTA02017219.1|:32437-32601
NuwaII-1_MZeb	gb|AGTA02001395.1|:1525-1680
NuwaII-1_MZeb	gb|AGTA02065898.1|:1-152
NuwaII-1_MZeb	gb|AGTA02059422.1|:1825-1972
NuwaII-1_MZeb	gb|AGTA02065674.1|:1-155
NuwaII-1_MZeb	gb|AGTA02053185.1|:1-151
NuwaII-1_MZeb	gb|AGTA02031666.1|:741-893
NuwaII-1_MZeb	gb|AGTA02028201.1|:1-146
NuwaII-1_MZeb	gb|AGTA02003552.1|:1-166
NuwaII-1_MZeb	gb|AGTA02064886.1|:1379-1526
NuwaII-1_MZeb	gb|AGTA02041353.1|:1-156
NuwaII-1_MZeb	gb|AGTA02069536.1|:3544-3707
NuwaII-1_MZeb	gb|AGTA02051880.1|:3966-4133
NuwaII-1_MZeb	gb|AGTA02047370.1|:28229-28376
NuwaII-1_MZeb	gb|AGTA02031667.1|:1-148
NuwaII-1_MZeb	gb|AGTA02012854.1|:8474-8637
NuwaII-1_MZeb	gb|AGTA02069268.1|:4699-4836
NuwaII-1_MZeb	gb|AGTA02059859.1|:11101-11362
NuwaII-1_MZeb	gb|AGTA02037953.1|:3683-3829

NuwaII-1_MZeb	gb|AGTA02022661.1|:1-142
NuwaII-1_MZeb	gb|AGTA02050624.1|:1426-1562
NuwaII-1_MZeb	gb|AGTA02040191.1|:1-141
NuwaII-1_MZeb	gb|AGTA02032256.1|:22444-22609
NuwaII-1_MZeb	gb|AGTA02012515.1|:46602-46763
NuwaII-1_MZeb	gb|AGTA02056413.1|:1-144
NuwaII-1_MZeb	gb|AGTA02052565.1|:1-141
NuwaII-1_MZeb	gb|AGTA02025140.1|:4-163
NuwaII-1_MZeb	gb|AGTA02060539.1|:1-156
NuwaII-1_MZeb	gb|AGTA02016257.1|:1-160
NuwaII-1_MZeb	gb|AGTA02008728.1|:8331-8470
NuwaII-1_MZeb	gb|AGTA02005486.1|:9600-9759
NuwaII-1_MZeb	gb|AGTA02054836.1|:1-147
NuwaII-1_MZeb	gb|AGTA02001601.1|:2689-2819
NuwaII-1_MZeb	gb|AGTA02000011.1|:9604-9745
NuwaII-1_MZeb	gb|AGTA02063731.1|:1-145
NuwaII-1_MZeb	gb|AGTA02025969.1|:21886-22025
NuwaII-1_MZeb	gb|AGTA02049120.1|:8562-8711
NuwaII-1_MZeb	gb|AGTA02039334.1|:1-143
NuwaII-1_MZeb	gb|AGTA02007198.1|:1417-1553
NuwaII-1_MZeb	gb|AGTA02059867.1|:8055-8194
NuwaII-1_MZeb	gb|AGTA02064191.1|:3491-3617
NuwaII-1_MZeb	gb|AGTA02024109.1|:7103-7246
NuwaII-1_MZeb	gb|AGTA02033772.1|:1376-1518
NuwaII-1_MZeb	gb|AGTA02019998.1|:29238-29375
NuwaII-1_MZeb	gb|AGTA02017362.1|:1-147
NuwaII-1_MZeb	gb|AGTA02050052.1|:1-121
NuwaII-1_MZeb	gb|AGTA02038916.1|:1-121
NuwaII-1_MZeb	gb|AGTA02004553.1|:5264-5384
NuwaII-1_MZeb	gb|AGTA02036817.1|:15-412
NuwaII-1_MZeb	gb|AGTA02062249.1|:2482-2604
NuwaII-1_MZeb	gb|AGTA02048561.1|:1-126
NuwaII-1_MZeb	gb|AGTA02020791.1|:1-126
NuwaII-1_MZeb	gb|AGTA02061647.1|:3416-3553
NuwaII-1_MZeb	gb|AGTA02056999.1|:31-148
NuwaII-1_MZeb	gb|AGTA02048617.1|:3161-3281
NuwaII-1_MZeb	gb|AGTA02033013.1|:13709-13825
NuwaII-1_MZeb	gb|AGTA02039119.1|:3095-3226
NuwaII-1_MZeb	gb|AGTA02035717.1|:5844-5968
NuwaII-1_MZeb	gb|AGTA02066919.1|:1812-1926
NuwaII-1_MZeb	gb|AGTA02051667.1|:1-128
NuwaII-1_MZeb	gb|AGTA02049121.1|:1-144
NuwaII-1_MZeb	gb|AGTA02070775.1|:1347-1464
NuwaII-1_MZeb	gb|AGTA02055520.1|:1647-1769

NuwaII-1_MZeb	gb|AGTA02004554.1|:1-119
NuwaII-1_MZeb	gb|AGTA02064767.1|:1-121
NuwaII-1_MZeb	gb|AGTA02051670.1|:4405-4525
NuwaII-1_MZeb	gb|AGTA02045941.1|:1-113
NuwaII-1_MZeb	gb|AGTA02019559.1|:8084-8196
NuwaII-1_MZeb	gb|AGTA02046547.1|:1426-1562
NuwaII-1_MZeb	gb|AGTA02033014.1|:1-129
NuwaII-1_MZeb	gb|AGTA02059090.1|:129-247
NuwaII-1_MZeb	gb|AGTA02051527.1|:1-127
NuwaII-1_MZeb	gb|AGTA02019999.1|:1-139
NuwaII-1_MZeb	gb|AGTA02039104.1|:24679-24791
NuwaII-1_MZeb	gb|AGTA02038706.1|:1-113
NuwaII-1_MZeb	gb|AGTA02026675.1|:1-113
NuwaII-1_MZeb	gb|AGTA02017195.1|:1-113
NuwaII-1_MZeb	gb|AGTA02059070.1|:6949-7061
NuwaII-1_MZeb	gb|AGTA02046784.1|:1-131
NuwaII-1_MZeb	gb|AGTA02024598.1|:1-123
NuwaII-1_MZeb	gb|AGTA02066684.1|:1-130
NuwaII-1_MZeb	gb|AGTA02064312.1|:1139-1236
NuwaII-1_MZeb	gb|AGTA02053165.1|:1-106
NuwaII-1_MZeb	gb|AGTA02051881.1|:1-113
NuwaII-1_MZeb	gb|AGTA02032981.1|:1-113
NuwaII-1_MZeb	gb|AGTA02010728.1|:1-113
NuwaII-1_MZeb	gb|AGTA02008007.1|:1810-1922
NuwaII-1_MZeb	gb|AGTA02003551.1|:13953-14065
NuwaII-1_MZeb	gb|AGTA02048854.1|:1-108
NuwaII-1_MZeb	gb|AGTA02027644.1|:1-104
NuwaII-1_MZeb	gb|AGTA02024267.1|:1-104
NuwaII-1_MZeb	gb|AGTA02071555.1|:149-263
NuwaII-2_MZeb	gb|AGTA02010421.1|:11528-12641
NuwaII-2_MZeb	gb|AGTA02022206.1|:30734-31843
NuwaII-2_MZeb	gb|AGTA02038903.1|:20355-21464
NuwaII-2_MZeb	gb|AGTA02026247.1|:26581-27690
NuwaII-2_MZeb	gb|AGTA02067983.1|:5902-7011
NuwaII-2_MZeb	gb|AGTA02008309.1|:3306-4414
NuwaII-2_MZeb	gb|AGTA02052077.1|:4698-5806
NuwaII-2_MZeb	gb|AGTA02055499.1|:9645-10746
NuwaII-2_MZeb	gb|AGTA02023643.1|:8115-9220
NuwaII-2_MZeb	gb|AGTA02052506.1|:450-1364
NuwaII-2_MZeb	gb|AGTA02001486.1|:3639-4275
NuwaII-2_MZeb	gb|AGTA02060052.1|:4711-7748
NuwaII-2_MZeb	gb|AGTA02050326.1|:6486-7122
NuwaII-2_MZeb	gb|AGTA02035474.1|:1-487
NuwaII-2_MZeb	gb|AGTA02024328.1|:46893-47349

	NuwaII-2_MZeb	gb|AGTA02063916.1|:1-422
	NuwaII-2_MZeb	gb|AGTA02012741.1|:1-450
	NuwaII-2_MZeb	gb|AGTA02035432.1|:503-882
	NuwaII-2_MZeb	gb|AGTA02008706.1|:47689-48057
	NuwaII-2_MZeb	gb|AGTA02004322.1|:10168-10549
	NuwaII-2_MZeb	gb|AGTA02029063.1|:916-1269
	NuwaII-2_MZeb	gb|AGTA02063312.1|:1-309
	NuwaII-2_MZeb	gb|AGTA02008707.1|:1-278
	NuwaII-2_MZeb	gb|AGTA02029769.1|:21568-21850
	NuwaII-2_MZeb	gb|AGTA02035473.1|:81068-81339
	NuwaII-2_MZeb	gb|AGTA02000583.1|:1-197
	NuwaII-2_MZeb	gb|AGTA02063915.1|:4758-4938
	NuwaII-2_MZeb	gb|AGTA02024095.1|:10987-11159
	NuwaII-2_MZeb	gb|AGTA02002458.1|:1-158
	NuwaII-2_MZeb	gb|AGTA02000582.1|:11243-11404
	NuwaII-2_MZeb	gb|AGTA02020816.1|:17043-17199
	NuwaII-2_MZeb	gb|AGTA02041527.1|:3363-3509
	NuwaII-2_MZeb	gb|AGTA02033253.1|:1-147
	NuwaII-2_MZeb	gb|AGTA02067579.1|:4361-4506
	NuwaII-2_MZeb	gb|AGTA02031429.1|:1-143
	NuwaII-2_MZeb	gb|AGTA02033252.1|:7952-8093
	NuwaII-2_MZeb	gb|AGTA02000550.1|:30216-30359
	NuwaII-2_MZeb	gb|AGTA02029790.1|:3503-3636
	NuwaII-2_MZeb	gb|AGTA02041528.1|:1-108
	NuwaII-2_MZeb	gb|AGTA02004323.1|:1-108
	NuwaII-2_MZeb	gb|AGTA02050237.1|:13018-13127
	NuwaII-2_MZeb	gb|AGTA02025907.1|:4904-4996
	NuwaII-2_MZeb	gb|AGTA02063311.1|:18737-18823
	NuwaII-2_MZeb	gb|AGTA02010513.1|:16579-16664
	NuwaII-2_MZeb	gb|AGTA02012740.1|:3023-3091
	NuwaII-2_MZeb	gb|AGTA02035431.1|:10291-10357
	NuwaII-2_MZeb	gb|AGTA02018634.1|:1-61
	NuwaII-2_MZeb	gb|AGTA02050238.1|:1-68
	NuwaII-2_MZeb	gb|AGTA02005493.1|:6146-6229
Poecilia formosa	*NuwaII_PFor*	gb|AYCK01015489.1|:24823-25417
	NuwaII_PFor	gb|AYCK01000517.1|:9426-10020
	NuwaII_PFor	gb|AYCK01014261.1|:651-1245
	NuwaII_PFor	gb|AYCK01001717.1|:197061-197654
	NuwaII_PFor	gb|AYCK01000751.1|:22360-22953
	NuwaII_PFor	gb|AYCK01015431.1|:11592-12185
	NuwaII_PFor	gb|AYCK01018035.1|:32633-33226
	NuwaII_PFor	gb|AYCK01009731.1|:54619-55211
	NuwaII_PFor	gb|AYCK01008077.1|:144876-145467
	NuwaII_PFor	gb|AYCK01015507.1|:44457-45048

NuwaII_PFor	gb|AYCK01008623.1|:4511-5102
NuwaII_PFor	gb|AYCK01006975.1|:62860-63451
NuwaII_PFor	gb|AYCK01010213.1|:24577-25168
NuwaII_PFor	gb|AYCK01009520.1|:50701-51292
NuwaII_PFor	gb|AYCK01008078.1|:1434-2025
NuwaII_PFor	gb|AYCK01021035.1|:17850-18441
NuwaII_PFor	gb|AYCK01019071.1|:25800-26391
NuwaII_PFor	gb|AYCK01019519.1|:47911-48502
NuwaII_PFor	gb|AYCK01011123.1|:42-633
NuwaII_PFor	gb|AYCK01008067.1|:62350-62941
NuwaII_PFor	gb|AYCK01001913.1|:67108-67699
NuwaII_PFor	gb|AYCK01026302.1|:3102-3692
NuwaII_PFor	gb|AYCK01010269.1|:6147-6737
NuwaII_PFor	gb|AYCK01012156.1|:2669-3259
NuwaII_PFor	gb|AYCK01010270.1|:1341-1931
NuwaII_PFor	gb|AYCK01000565.1|:2989-3579
NuwaII_PFor	gb|AYCK01019130.1|:11411-12001
NuwaII_PFor	gb|AYCK01003473.1|:23226-23816
NuwaII_PFor	gb|AYCK01017785.1|:48233-48823
NuwaII_PFor	gb|AYCK01008460.1|:4975-5565
NuwaII_PFor	gb|AYCK01010307.1|:10615-11205
NuwaII_PFor	gb|AYCK01003640.1|:29702-30292
NuwaII_PFor	gb|AYCK01007992.1|:1793-2383
NuwaII_PFor	gb|AYCK01012290.1|:7533-8123
NuwaII_PFor	gb|AYCK01028230.1|:109-699
NuwaII_PFor	gb|AYCK01002109.1|:143-733
NuwaII_PFor	gb|AYCK01020740.1|:13735-14325
NuwaII_PFor	gb|AYCK01006899.1|:103213-103803
NuwaII_PFor	gb|AYCK01005505.1|:41223-41812
NuwaII_PFor	gb|AYCK01000595.1|:93829-94418
NuwaII_PFor	gb|AYCK01005927.1|:38001-38590
NuwaII_PFor	gb|AYCK01019298.1|:10130-10719
NuwaII_PFor	gb|AYCK01026813.1|:6940-7529
NuwaII_PFor	gb|AYCK01013735.1|:12388-12977
NuwaII_PFor	gb|AYCK01026491.1|:19622-20211
NuwaII_PFor	gb|AYCK01018366.1|:21388-21977
NuwaII_PFor	gb|AYCK01021036.1|:921-1510
NuwaII_PFor	gb|AYCK01001780.1|:53441-54030
NuwaII_PFor	gb|AYCK01000531.1|:24692-25281
NuwaII_PFor	gb|AYCK01010451.1|:138277-138866
NuwaII_PFor	gb|AYCK01010245.1|:2179-2768
NuwaII_PFor	gb|AYCK01025428.1|:239-828
NuwaII_PFor	gb|AYCK01005425.1|:20721-21310
NuwaII_PFor	gb|AYCK01010357.1|:2194-2783

NuwaII_PFor	gb|AYCK01002626.1|:11113-11702
NuwaII_PFor	gb|AYCK01026862.1|:3504-4092
NuwaII_PFor	gb|AYCK01007806.1|:136392-136980
NuwaII_PFor	gb|AYCK01014379.1|:24081-24669
NuwaII_PFor	gb|AYCK01008077.1|:19280-19868
NuwaII_PFor	gb|AYCK01007918.1|:3743-4331
NuwaII_PFor	gb|AYCK01007917.1|:3301-3889
NuwaII_PFor	gb|AYCK01025757.1|:5942-6530
NuwaII_PFor	gb|AYCK01010036.1|:21644-22232
NuwaII_PFor	gb|AYCK01024564.1|:588-1176
NuwaII_PFor	gb|AYCK01014907.1|:313-900
NuwaII_PFor	gb|AYCK01023078.1|:2362-2949
NuwaII_PFor	gb|AYCK01012510.1|:18631-19218
NuwaII_PFor	gb|AYCK01015257.1|:16075-16662
NuwaII_PFor	gb|AYCK01021312.1|:13093-13680
NuwaII_PFor	gb|AYCK01000651.1|:19168-19755
NuwaII_PFor	gb|AYCK01022493.1|:7477-8064
NuwaII_PFor	gb|AYCK01020579.1|:471-1058
NuwaII_PFor	gb|AYCK01020567.1|:3685-4272
NuwaII_PFor	gb|AYCK01013953.1|:13474-14061
NuwaII_PFor	gb|AYCK01009758.1|:70243-70829
NuwaII_PFor	gb|AYCK01010198.1|:1344-1930
NuwaII_PFor	gb|AYCK01029940.1|:90-676
NuwaII_PFor	gb|AYCK01007738.1|:2341-2926
NuwaII_PFor	gb|AYCK01029445.1|:6677-7262
NuwaII_PFor	gb|AYCK01024771.1|:10434-11018
NuwaII_PFor	gb|AYCK01012148.1|:2362-2946
NuwaII_PFor	gb|AYCK01029609.1|:2195-2779
NuwaII_PFor	gb|AYCK01020566.1|:22877-23461
NuwaII_PFor	gb|AYCK01003303.1|:12140-12724
NuwaII_PFor	gb|AYCK01023495.1|:39391-39974
NuwaII_PFor	gb|AYCK01022522.1|:7909-8490
NuwaII_PFor	gb|AYCK01013057.1|:12994-13574
NuwaII_PFor	gb|AYCK01007155.1|:26950-27529
NuwaII_PFor	gb|AYCK01025105.1|:5072-5650
NuwaII_PFor	gb|AYCK01014524.1|:82858-83436
NuwaII_PFor	gb|AYCK01006130.1|:49037-49614
NuwaII_PFor	gb|AYCK01014406.1|:38590-39167
NuwaII_PFor	gb|AYCK01009304.1|:4689-5290
NuwaII_PFor	gb|AYCK01013799.1|:14935-15532
NuwaII_PFor	gb|AYCK01013499.1|:56163-56756
NuwaII_PFor	gb|AYCK01025209.1|:563-2519
NuwaII_PFor	gb|AYCK01025110.1|:4920-5513
NuwaII_PFor	gb|AYCK01002622.1|:37112-37702

	NuwaII_PFor	gb|AYCK01027470.1|:5843-6431
	NuwaII_PFor	gb|AYCK01018239.1|:13876-15830
	NuwaII_PFor	gb|AYCK01018113.1|:7306-7901
	NuwaII_PFor	gb|AYCK01015507.1|:112921-113499
	NuwaII_PFor	gb|AYCK01010434.1|:14474-15049
	NuwaII_PFor	gb|AYCK01013971.1|:12342-12922
	NuwaII_PFor	gb|AYCK01025089.1|:1100-1676
	NuwaII_PFor	gb|AYCK01021035.1|:4528-5104
	NuwaII_PFor	gb|AYCK01018860.1|:9621-10195
	NuwaII_PFor	gb|AYCK01012612.1|:41638-42210
	NuwaII_PFor	gb|AYCK01018661.1|:12170-12742
	NuwaII_PFor	gb|AYCK01013858.1|:2812-3382
	NuwaII_PFor	gb|AYCK01022638.1|:26742-27310
	NuwaII_PFor	gb|AYCK01006284.1|:106914-107482
	NuwaII_PFor	gb|AYCK01020284.1|:19406-19974
	NuwaII_PFor	gb|AYCK01011237.1|:1431-2007
	NuwaII_PFor	gb|AYCK01006321.1|:1571-2144
	NuwaII_PFor	gb|AYCK01015332.1|:86774-87347
	NuwaII_PFor	gb|AYCK01006874.1|:1404-1970
	NuwaII_PFor	gb|AYCK01002659.1|:15279-15853
	NuwaII_PFor	gb|AYCK01026016.1|:5054-5628
	NuwaII_PFor	gb|AYCK01015257.1|:3405-3977
	NuwaII_PFor	gb|AYCK01017205.1|:8221-8793
	NuwaII_PFor	gb|AYCK01014235.1|:13369-14051
	NuwaII_PFor	gb|AYCK01004401.1|:16438-17042
	NuwaII_PFor	gb|AYCK01010434.1|:28675-29252
	NuwaII_PFor	gb|AYCK01000214.1|:34670-35299
	NuwaII_PFor	gb|AYCK01003887.1|:1597-2172
	NuwaII_PFor	gb|AYCK01004341.1|:1788-2366
	NuwaII_PFor	gb|AYCK01003291.1|:63418-63978
	NuwaII_PFor	gb|AYCK01015456.1|:84407-84991
	NuwaII_PFor	gb|AYCK01014582.1|:3231-3808
	NuwaII_PFor	gb|AYCK01014583.1|:1468-2045
	NuwaII_PFor	gb|AYCK01006284.1|:145071-145625
	NuwaII_PFor	gb|AYCK01002043.1|:101485-102049
	NuwaII_PFor	gb|AYCK01021679.1|:12557-13120
	NuwaII_PFor	gb|AYCK01013587.1|:2758-3283
	NuwaII_PFor	gb|AYCK01026862.1|:3309-4092
Puccinia graminis	*NuwaII_PGra*	gb|AAWC01004180.1|:607-4122
	NuwaII_PGra	gb|AAWC01002289.1|:3626-10386
	NuwaII_PGra	gb|AAWC01000164.1|:3887-7392
	NuwaII_PGra	gb|AAWC01001403.1|:122844-126189
	NuwaII_PGra	gb|AAWC01004341.1|:9169-10453
	NuwaII_PGra	gb|AAWC01000728.1|:18694-20944

NuwaII_PGra	gb|AAWC01001561.1|:312-3773
NuwaII_PGra	gb|AAWC01003410.1|:4615-5951
NuwaII_PGra	gb|AAWC01000040.1|:563-1182
NuwaII_PGra	gb|AAWC01002924.1|:49661-50448
NuwaII_PGra	gb|AAWC01001620.1|:21723-22475
NuwaII_PGra	gb|AAWC01000593.1|:10080-11254
NuwaII_PGra	gb|AAWC01001978.1|:8156-8921
NuwaII_PGra	gb|AAWC01000530.1|:53664-54427
NuwaII_PGra	gb|AAWC01002538.1|:6694-7457
NuwaII_PGra	gb|AAWC01001565.1|:267-1030
NuwaII_PGra	gb|AAWC01002083.1|:20-614
NuwaII_PGra	gb|AAWC01001609.1|:111297-112043
NuwaII_PGra	gb|AAWC01003252.1|:64105-64301
NuwaII_PGra	gb|AAWC01000530.1|:24427-24617
NuwaII_PGra	gb|AAWC01004267.1|:4493-4683
NuwaII_PGra	gb|AAWC01001961.1|:5311-5501
NuwaII_PGra	gb|AAWC01003411.1|:66-231
NuwaII_PGra	gb|AAWC01001389.1|:285-450
NuwaII_PGra	gb|AAWC01001421.1|:46436-47378
NuwaII_PGra	gb|AAWC01002978.1|:1218-1367
NuwaII_PGra	gb|AAWC01002460.1|:99264-102571
NuwaII_PGra	gb|AAWC01000063.1|:52607-52745
NuwaII_PGra	gb|AAWC01002182.1|:7404-7995
NuwaII_PGra	gb|AAWC01002881.1|:49770-50421
NuwaII_PGra	gb|AAWC01002420.1|:30241-30767
NuwaII_PGra	gb|AAWC01003772.1|:5449-5738
NuwaII_PGra	gb|AAWC01004551.1|:2115-2701
NuwaII_PGra	gb|AAWC01003095.1|:321-908
NuwaII_PGra	gb|AAWC01002198.1|:25085-25667
NuwaII_PGra	gb|AAWC01000034.1|:63676-64894
NuwaII_PGra	gb|AAWC01000834.1|:976-1561
NuwaII_PGra	gb|AAWC01000118.1|:553-1138
NuwaII_PGra	gb|AAWC01001261.1|:23500-25560
NuwaII_PGra	gb|AAWC01001558.1|:2191-2866
NuwaII_PGra	gb|AAWC01003834.1|:28247-28337
NuwaII_PGra	gb|AAWC01003485.1|:37381-37471
NuwaII_PGra	gb|AAWC01003951.1|:70676-70765
NuwaII_PGra	gb|AAWC01002444.1|:643-732
NuwaII_PGra	gb|AAWC01000310.1|:62011-62098
NuwaII_PGra	gb|AAWC01003210.1|:22021-22108
NuwaII_PGra	gb|AAWC01001305.1|:6339-6426
NuwaII_PGra	gb|AAWC01000728.1|:33963-34045
NuwaII_PGra	gb|AAWC01000795.1|:24718-24794
NuwaII_PGra	gb|AAWC01004325.1|:4498-4570

NuwaII_PGra	gb|AAWC01002621.1|:29532-29597
NuwaII_PGra	gb|AAWC01003260.1|:44024-44087
NuwaII_PGra	gb|AAWC01003399.1|:43040-43100
NuwaII_PGra	gb|AAWC01002817.1|:30978-31038
NuwaII_PGra	gb|AAWC01002180.1|:26094-26150
NuwaII_PGra	gb|AAWC01000689.1|:10671-10727
NuwaII_PGra	gb|AAWC01004438.1|:2813-6022
NuwaII_PGra	gb|AAWC01000785.1|:13605-16971
NuwaII_PGra	gb|AAWC01001642.1|:10437-14011
NuwaII_PGra	gb|AAWC01000785.1|:68376-69591
NuwaII_PGra	gb|AAWC01002704.1|:185-696
NuwaII_PGra	gb|AAWC01001421.1|:11645-12651
NuwaII_PGra	gb|AAWC01001583.1|:49037-49781
NuwaII_PGra	gb|AAWC01001936.1|:31615-32379
NuwaII_PGra	gb|AAWC01001809.1|:43563-44330
NuwaII_PGra	gb|AAWC01001334.1|:1836-3587
NuwaII_PGra	gb|AAWC01000157.1|:34990-36734
NuwaII_PGra	gb|AAWC01001669.1|:13383-14149
NuwaII_PGra	gb|AAWC01003620.1|:35268-36040
NuwaII_PGra	gb|AAWC01000465.1|:11929-12674
NuwaII_PGra	gb|AAWC01000222.1|:11120-11844
NuwaII_PGra	gb|AAWC01003067.1|:2644-4163
NuwaII_PGra	gb|AAWC01002750.1|:16042-16631
NuwaII_PGra	gb|AAWC01003982.1|:15699-15863
NuwaII_PGra	gb|AAWC01003925.1|:8918-9083
NuwaII_PGra	gb|AAWC01000550.1|:63656-63820
NuwaII_PGra	gb|AAWC01003497.1|:26085-26243
NuwaII_PGra	gb|AAWC01000982.1|:20781-21373
NuwaII_PGra	gb|AAWC01001833.1|:38239-38381
NuwaII_PGra	gb|AAWC01001476.1|:87960-88546
NuwaII_PGra	gb|AAWC01000510.1|:1354-2017
NuwaII_PGra	gb|AAWC01000310.1|:63770-64332
NuwaII_PGra	gb|AAWC01003641.1|:16930-17038
NuwaII_PGra	gb|AAWC01002408.1|:8923-9602
NuwaII_PGra	gb|AAWC01001998.1|:67261-69715
NuwaII_PGra	gb|AAWC01004480.1|:3951-4625
NuwaII_PGra	gb|AAWC01001888.1|:191203-191883
NuwaII_PGra	gb|AAWC01000357.1|:510-599
NuwaII_PGra	gb|AAWC01004474.1|:2671-2758
NuwaII_PGra	gb|AAWC01002317.1|:8-79
NuwaII_PGra	gb|AAWC01002764.1|:13450-13519
NuwaII_PGra	gb|AAWC01001646.1|:14153-14221
NuwaII_PGra	gb|AAWC01001065.1|:100208-100276
NuwaII_PGra	gb|AAWC01000267.1|:23988-24056

	NuwaII_PGra	gb|AAWC01000783.1|:9109-9176
	NuwaII_PGra	gb|AAWC01002538.1|:18622-18678
	NuwaII_PGra	gb|AAWC01003496.1|:92841-92892
	NuwaII_PGra	gb|AAWC01001609.1|:62139-62188
	NuwaII_PGra	gb|AAWC01000397.1|:17488-17537
Puccinia striiformis	*NuwaII_PStr*	gb|ANHQ01001561.1|:595-5251
	NuwaII_PStr	gb|ANHQ01007519.1|:25671-28107
	NuwaII_PStr	gb|ANHQ01004300.1|:20626-23062
	NuwaII_PStr	gb|ANHQ01003896.1|:3946-7070
	NuwaII_PStr	gb|ANHQ01011133.1|:2054-5181
	NuwaII_PStr	gb|ANHQ01012080.1|:684-1969
	NuwaII_PStr	gb|ANHQ01006309.1|:17349-18636
	NuwaII_PStr	gb|ANHQ01001938.1|:16506-17900
	NuwaII_PStr	gb|ANHQ01004712.1|:36853-39678
	NuwaII_PStr	gb|ANHQ01004575.1|:4667-6838
	NuwaII_PStr	gb|ANHQ01000661.1|:5346-6550
	NuwaII_PStr	gb|ANHQ01009175.1|:7819-10917
	NuwaII_PStr	gb|ANHQ01001341.1|:424-1713
	NuwaII_PStr	gb|ANHQ01002156.1|:100-1512
	NuwaII_PStr	gb|ANHQ01002937.1|:2210-3598
	NuwaII_PStr	gb|ANHQ01012081.1|:76-976
	NuwaII_PStr	gb|ANHQ01001680.1|:92-990
	NuwaII_PStr	gb|ANHQ01000127.1|:2412-2945
	NuwaII_PStr	gb|ANHQ01006282.1|:596-2074
	NuwaII_PStr	gb|ANHQ01000752.1|:1-514
	NuwaII_PStr	gb|ANHQ01002181.1|:11480-12046
	NuwaII_PStr	gb|ANHQ01009318.1|:2458-2824
	NuwaII_PStr	gb|ANHQ01005883.1|:483-707
	NuwaII_PStr	gb|ANHQ01008793.1|:8563-8787
	NuwaII_PStr	gb|ANHQ01009392.1|:18677-18901
	NuwaII_PStr	gb|ANHQ01012241.1|:10402-10758
	NuwaII_PStr	gb|ANHQ01003557.1|:14428-14784
	NuwaII_PStr	gb|ANHQ01003275.1|:12272-12476
	NuwaII_PStr	gb|ANHQ01012091.1|:5092-5512
	NuwaII_PStr	gb|ANHQ01011870.1|:9066-9744
	NuwaII_PStr	gb|ANHQ01008232.1|:12757-13434
	NuwaII_PStr	gb|ANHQ01008790.1|:1928-2273
	NuwaII_PStr	gb|ANHQ01012227.1|:2818-2967
	NuwaII_PStr	gb|ANHQ01007747.1|:14597-14746
	NuwaII_PStr	gb|ANHQ01005608.1|:5045-5270
	NuwaII_PStr	gb|ANHQ01010122.1|:28-170
	NuwaII_PStr	gb|ANHQ01012111.1|:15981-16113
	NuwaII_PStr	gb|ANHQ01004740.1|:4852-4981
	NuwaII_PStr	gb|ANHQ01008256.1|:10714-10843

NuwaII_PStr	gb|ANHQ01007633.1|:165-289
NuwaII_PStr	gb|ANHQ01011134.1|:7445-7561
NuwaII_PStr	gb|ANHQ01003982.1|:72007-72097
NuwaII_PStr	gb|ANHQ01004968.1|:6011-6099
NuwaII_PStr	gb|ANHQ01000547.1|:45308-45370
NuwaII_PStr	gb|ANHQ01008667.1|:893-951
NuwaII_PStr	gb|ANHQ01001068.1|:6861-6919
NuwaII_PStr	gb|ANHQ01001045.1|:2017-2075
NuwaII_PStr	gb|ANHQ01008444.1|:4473-4522
NuwaII_PStr	gb|ANHQ01007774.1|:15471-19065
NuwaII_PStr	gb|ANHQ01007761.1|:5111-8623
NuwaII_PStr	gb|ANHQ01006704.1|:8059-11571
NuwaII_PStr	gb|ANHQ01007561.1|:2697-4046
NuwaII_PStr	gb|ANHQ01011086.1|:2226-4015
NuwaII_PStr	gb|ANHQ01009342.1|:1691-4488
NuwaII_PStr	gb|ANHQ01006790.1|:8622-11379
NuwaII_PStr	gb|ANHQ01010845.1|:622-3833
NuwaII_PStr	gb|ANHQ01006206.1|:10587-11783
NuwaII_PStr	gb|ANHQ01009053.1|:50550-52515
NuwaII_PStr	gb|ANHQ01005807.1|:1389-3354
NuwaII_PStr	gb|ANHQ01007319.1|:28-1302
NuwaII_PStr	gb|ANHQ01001559.1|:614-1610
NuwaII_PStr	gb|ANHQ01000322.1|:2-964
NuwaII_PStr	gb|ANHQ01005232.1|:20683-21639
NuwaII_PStr	gb|ANHQ01007559.1|:69174-70074
NuwaII_PStr	gb|ANHQ01009950.1|:8042-8931
NuwaII_PStr	gb|ANHQ01003127.1|:7977-8866
NuwaII_PStr	gb|ANHQ01009346.1|:7516-8376
NuwaII_PStr	gb|ANHQ01003143.1|:19923-20476
NuwaII_PStr	gb|ANHQ01000321.1|:614-1019
NuwaII_PStr	gb|ANHQ01004038.1|:357-762
NuwaII_PStr	gb|ANHQ01001193.1|:384-789
NuwaII_PStr	gb|ANHQ01008280.1|:1-1287
NuwaII_PStr	gb|ANHQ01000320.1|:1640-2071
NuwaII_PStr	gb|ANHQ01010004.1|:1635-2053
NuwaII_PStr	gb|ANHQ01011817.1|:34119-34537
NuwaII_PStr	gb|ANHQ01003970.1|:50120-50486
NuwaII_PStr	gb|ANHQ01011732.1|:1244-2434
NuwaII_PStr	gb|ANHQ01003365.1|:2655-3072
NuwaII_PStr	gb|ANHQ01007961.1|:7934-8312
NuwaII_PStr	gb|ANHQ01007233.1|:8681-8905
NuwaII_PStr	gb|ANHQ01000342.1|:1450-1674
NuwaII_PStr	gb|ANHQ01001558.1|:2488-3127
NuwaII_PStr	gb|ANHQ01007923.1|:15359-15698

	NuwaII_PStr	gb|ANHQ01006370.1|:111-269
	NuwaII_PStr	gb|ANHQ01002707.1|:635-791
	NuwaII_PStr	gb|ANHQ01011670.1|:2685-2835
	NuwaII_PStr	gb|ANHQ01009884.1|:36256-36481
	NuwaII_PStr	gb|ANHQ01006369.1|:1532-2515
	NuwaII_PStr	gb|ANHQ01011331.1|:616-751
	NuwaII_PStr	gb|ANHQ01012110.1|:21188-21320
	NuwaII_PStr	gb|ANHQ01001192.1|:6924-7053
	NuwaII_PStr	gb|ANHQ01007853.1|:2806-2884
	NuwaII_PStr	gb|ANHQ01002708.1|:170-245
	NuwaII_PStr	gb|ANHQ01002709.1|:72-138
	NuwaII_PStr	gb|ANHQ01009679.1|:17180-17245
	NuwaII_PStr	gb|ANHQ01007560.1|:525-583
	NuwaII_PStr	gb|ANHQ01007728.1|:2979-3037
	NuwaII_PStr	gb|ANHQ01000154.1|:16849-16898
Xenopus tropicalis	*NuwaII-1_XTro*	gb|AAMC02030921.1|:17334-17688
	NuwaII-1_XTro	gb|AAMC02002687.1|:21796-22071
	NuwaII-1_XTro	gb|AAMC02011404.1|:74730-74351
	NuwaII-1_XTro	gb|AAMC02008245.1|:133330-132987
	NuwaII-1_XTro	gb|AAMC02005706.1|:155731-155479
	NuwaII-1_XTro	gb|AAMC02029897.1|:9609-9967
	NuwaII-1_XTro	gb|AAMC02005522.1|:38047-38386
	NuwaII-1_XTro	gb|AAMC02004927.1|:51864-51464
	NuwaII-1_XTro	gb|AAMC02003566.1|:49331-49728
	NuwaII-1_XTro	gb|AAMC02035177.1|:67389-67075
	NuwaII-1_XTro	gb|AAMC02032071.1|:7677-7283
	NuwaII-1_XTro	gb|AAMC02026681.1|:100545-100146
	NuwaII-1_XTro	gb|AAMC02030712.1|:186690-186333
	NuwaII-1_XTro	gb|AAMC02016994.1|:68297-68550
	NuwaII-1_XTro	gb|AAMC02007550.1|:56104-56501
	NuwaII-1_XTro	gb|AAMC02003158.1|:43915-43570
	NuwaII-1_XTro	gb|AAMC02035219.1|:6948-7352
	NuwaII-1_XTro	gb|AAMC02025987.1|:8380-8696
	NuwaII-1_XTro	gb|AAMC02010850.1|:8342-963
	NuwaII-1_XTro	gb|AAMC02035210.1|:35958-35598
	NuwaII-1_XTro	gb|AAMC02034192.1|:4062-3694
	NuwaII-1_XTro	gb|AAMC02031943.1|:83565-83306
	NuwaII-1_XTro	gb|AAMC02016750.1|:4555-4234
	NuwaII-1_XTro	gb|AAMC02011473.1|:500-118
	NuwaII-1_XTro	gb|AAMC02011415.1|:42593-42973
	NuwaII-1_XTro	gb|AAMC02011415.1|:136963-137324
	NuwaII-1_XTro	gb|AAMC02011415.1|:313250-313632
	NuwaII-1_XTro	gb|AAMC02009042.1|:9221-9445
	NuwaII-1_XTro	gb|AAMC02002465.1|:32410-32036

NuwaII-1_XTro	gb|AAMC02000402.1|:9076-8682
NuwaII-1_XTro	gb|AAMC02031102.1|:15989-15710
NuwaII-1_XTro	gb|AAMC02030568.1|:139989-140352
NuwaII-1_XTro	gb|AAMC02027469.1|:229573-229227
NuwaII-1_XTro	gb|AAMC02023741.1|:38470-38825
NuwaII-1_XTro	gb|AAMC02021620.1|:56937-57335
NuwaII-1_XTro	gb|AAMC02021620.1|:24350-23962
NuwaII-1_XTro	gb|AAMC02008483.1|:17519-17136
NuwaII-1_XTro	gb|AAMC02008330.1|:120752-120368
NuwaII-1_XTro	gb|AAMC02008330.1|:35126-34850
NuwaII-1_XTro	gb|AAMC02007762.1|:6506-6907
NuwaII-1_XTro	gb|AAMC02007382.1|:80382-80082
NuwaII-1_XTro	gb|AAMC02006123.1|:90074-90442
NuwaII-1_XTro	gb|AAMC02003021.1|:210439-210036
NuwaII-1_XTro	gb|AAMC02032266.1|:8305-10138
NuwaII-1_XTro	gb|AAMC02032266.1|:52840-53200
NuwaII-1_XTro	gb|AAMC02032266.1|:51541-51163
NuwaII-1_XTro	gb|AAMC02030169.1|:18577-18953
NuwaII-1_XTro	gb|AAMC02026443.1|:23934-24334
NuwaII-1_XTro	gb|AAMC02023681.1|:74447-74171
NuwaII-1_XTro	gb|AAMC02020918.1|:341-685
NuwaII-1_XTro	gb|AAMC02020339.1|:51515-51137
NuwaII-1_XTro	gb|AAMC02019204.1|:1582-1188
NuwaII-1_XTro	gb|AAMC02017352.1|:74471-74108
NuwaII-1_XTro	gb|AAMC02017065.1|:287456-287059
NuwaII-1_XTro	gb|AAMC02017065.1|:111348-110971
NuwaII-1_XTro	gb|AAMC02010695.1|:17356-17717
NuwaII-1_XTro	gb|AAMC02008007.1|:44980-45320
NuwaII-1_XTro	gb|AAMC02007346.1|:87972-87643
NuwaII-1_XTro	gb|AAMC02005890.1|:10006-10358
NuwaII-1_XTro	gb|AAMC02044575.1|:6368-6758
NuwaII-1_XTro	gb|AAMC02034194.1|:86587-86209
NuwaII-1_XTro	gb|AAMC02030770.1|:106934-107292
NuwaII-1_XTro	gb|AAMC02027282.1|:8785-9184
NuwaII-1_XTro	gb|AAMC02027282.1|:29746-30126
NuwaII-1_XTro	gb|AAMC02026069.1|:105199-105600
NuwaII-1_XTro	gb|AAMC02023643.1|:106312-106068
NuwaII-1_XTro	gb|AAMC02019921.1|:2107-2403
NuwaII-1_XTro	gb|AAMC02014682.1|:18723-18392
NuwaII-1_XTro	gb|AAMC02014573.1|:101069-101464
NuwaII-1_XTro	gb|AAMC02011478.1|:28401-28022
NuwaII-1_XTro	gb|AAMC02007776.1|:25183-25523
NuwaII-1_XTro	gb|AAMC02006330.1|:17349-17659
NuwaII-1_XTro	gb|AAMC02000916.1|:451-761

NuwaII-1_XTro	gb|AAMC02049611.1|:2858-2497
NuwaII-1_XTro	gb|AAMC02032351.1|:106609-106945
NuwaII-1_XTro	gb|AAMC02028984.1|:204579-204178
NuwaII-1_XTro	gb|AAMC02027836.1|:42239-42629
NuwaII-1_XTro	gb|AAMC02027474.1|:39770-40127
NuwaII-1_XTro	gb|AAMC02025838.1|:100911-100548
NuwaII-1_XTro	gb|AAMC02025457.1|:44409-44845
NuwaII-1_XTro	gb|AAMC02025457.1|:2924-2577
NuwaII-1_XTro	gb|AAMC02023629.1|:31282-31669
NuwaII-1_XTro	gb|AAMC02023439.1|:5423-5007
NuwaII-1_XTro	gb|AAMC02023175.1|:55933-55721
NuwaII-1_XTro	gb|AAMC02020928.1|:119204-119471
NuwaII-1_XTro	gb|AAMC02019521.1|:11890-11529
NuwaII-1_XTro	gb|AAMC02017877.1|:20322-20696
NuwaII-1_XTro	gb|AAMC02017702.1|:14327-13968
NuwaII-1_XTro	gb|AAMC02015334.1|:20524-20184
NuwaII-1_XTro	gb|AAMC02014755.1|:117416-123737
NuwaII-1_XTro	gb|AAMC02012458.1|:18616-18994
NuwaII-1_XTro	gb|AAMC02006982.1|:12166-12477
NuwaII-1_XTro	gb|AAMC02003556.1|:245678-246049
NuwaII-1_XTro	gb|AAMC02003556.1|:116596-116221
NuwaII-1_XTro	gb|AAMC02001199.1|:100746-100400
NuwaII-1_XTro	gb|AAMC02043603.1|:1083-743
NuwaII-1_XTro	gb|AAMC02035465.1|:9764-10164
NuwaII-1_XTro	gb|AAMC02035192.1|:25764-26169
NuwaII-1_XTro	gb|AAMC02026917.1|:65923-66274
NuwaII-1_XTro	gb|AAMC02025437.1|:200804-200398
NuwaII-1_XTro	gb|AAMC02025437.1|:180551-180241
NuwaII-1_XTro	gb|AAMC02024989.1|:45657-43312
NuwaII-1_XTro	gb|AAMC02023935.1|:1211-1653
NuwaII-1_XTro	gb|AAMC02023601.1|:186367-185889
NuwaII-1_XTro	gb|AAMC02023601.1|:142058-141697
NuwaII-1_XTro	gb|AAMC02021255.1|:106264-105871
NuwaII-1_XTro	gb|AAMC02021189.1|:6061-6462
NuwaII-1_XTro	gb|AAMC02021187.1|:47564-47917
NuwaII-1_XTro	gb|AAMC02021187.1|:9594-9202
NuwaII-1_XTro	gb|AAMC02021115.1|:23825-23423
NuwaII-1_XTro	gb|AAMC02019007.1|:4853-4584
NuwaII-1_XTro	gb|AAMC02018911.1|:17549-17183
NuwaII-1_XTro	gb|AAMC02016665.1|:130515-128758
NuwaII-1_XTro	gb|AAMC02012198.1|:35942-35549
NuwaII-1_XTro	gb|AAMC02008718.1|:71633-71998
NuwaII-1_XTro	gb|AAMC02008200.1|:23054-22654
NuwaII-1_XTro	gb|AAMC02006131.1|:21266-20911

NuwaII-1_XTro	gb|AAMC02004782.1|:41997-42329
NuwaII-1_XTro	gb|AAMC02002245.1|:115700-116104
NuwaII-1_XTro	gb|AAMC02038844.1|:33924-34284
NuwaII-1_XTro	gb|AAMC02032228.1|:44011-43630
NuwaII-1_XTro	gb|AAMC02032156.1|:56573-56968
NuwaII-1_XTro	gb|AAMC02029985.1|:27618-28018
NuwaII-1_XTro	gb|AAMC02026287.1|:26977-26588
NuwaII-1_XTro	gb|AAMC02025876.1|:11618-11988
NuwaII-1_XTro	gb|AAMC02024017.1|:194241-193874
NuwaII-1_XTro	gb|AAMC02023321.1|:145713-145371
NuwaII-1_XTro	gb|AAMC02022218.1|:6669-6288
NuwaII-1_XTro	gb|AAMC02021898.1|:19382-19803
NuwaII-1_XTro	gb|AAMC02021783.1|:26253-26530
NuwaII-1_XTro	gb|AAMC02021358.1|:31193-30877
NuwaII-1_XTro	gb|AAMC02019584.1|:149595-149241
NuwaII-1_XTro	gb|AAMC02019373.1|:198841-198452
NuwaII-1_XTro	gb|AAMC02018763.1|:3480-3099
NuwaII-1_XTro	gb|AAMC02014444.1|:39971-39585
NuwaII-1_XTro	gb|AAMC02014264.1|:35446-35806
NuwaII-1_XTro	gb|AAMC02013897.1|:14717-15112
NuwaII-1_XTro	gb|AAMC02012445.1|:19252-19637
NuwaII-1_XTro	gb|AAMC02011414.1|:82203-81843
NuwaII-1_XTro	gb|AAMC02010181.1|:60210-59819
NuwaII-1_XTro	gb|AAMC02007688.1|:43600-43999
NuwaII-1_XTro	gb|AAMC02006616.1|:428179-427868
NuwaII-1_XTro	gb|AAMC02006290.1|:5271-4869
NuwaII-1_XTro	gb|AAMC02001720.1|:46868-46469
NuwaII-1_XTro	gb|AAMC02001550.1|:6455-6102
NuwaII-1_XTro	gb|AAMC02001247.1|:58745-59014
NuwaII-1_XTro	gb|AAMC02049689.1|:1179-864
NuwaII-1_XTro	gb|AAMC02043971.1|:1939-2281
NuwaII-1_XTro	gb|AAMC02036934.1|:51403-51717
NuwaII-1_XTro	gb|AAMC02036081.1|:179610-179210
NuwaII-1_XTro	gb|AAMC02035556.1|:412-1459
NuwaII-1_XTro	gb|AAMC02033416.1|:35249-35518
NuwaII-1_XTro	gb|AAMC02032905.1|:92204-90379
NuwaII-1_XTro	gb|AAMC02032505.1|:124033-123765
NuwaII-1_XTro	gb|AAMC02032505.1|:113666-113306
NuwaII-1_XTro	gb|AAMC02032505.1|:83018-82728
NuwaII-1_XTro	gb|AAMC02032354.1|:7314-7714
NuwaII-1_XTro	gb|AAMC02030259.1|:189951-190323
NuwaII-1_XTro	gb|AAMC02030259.1|:10005-9648
NuwaII-1_XTro	gb|AAMC02030003.1|:4856-5225
NuwaII-1_XTro	gb|AAMC02025307.1|:2327-2717

NuwaII-1_XTro	gb|AAMC02025307.1|:48061-48461
NuwaII-1_XTro	gb|AAMC02025304.1|:77753-77385
NuwaII-1_XTro	gb|AAMC02020552.1|:23218-23620
NuwaII-1_XTro	gb|AAMC02019636.1|:64548-64874
NuwaII-1_XTro	gb|AAMC02015177.1|:34028-33666
NuwaII-1_XTro	gb|AAMC02011137.1|:125927-126286
NuwaII-1_XTro	gb|AAMC02008936.1|:13288-13684
NuwaII-1_XTro	gb|AAMC02008334.1|:16803-17201
NuwaII-1_XTro	gb|AAMC02008276.1|:18638-18282
NuwaII-1_XTro	gb|AAMC02007167.1|:42956-43271
NuwaII-1_XTro	gb|AAMC02004566.1|:6742-6352
NuwaII-1_XTro	gb|AAMC02004028.1|:39465-39068
NuwaII-1_XTro	gb|AAMC02001145.1|:155876-156238
NuwaII-1_XTro	gb|AAMC02000849.1|:11562-11962
NuwaII-1_XTro	gb|AAMC02036954.1|:13425-13002
NuwaII-1_XTro	gb|AAMC02036940.1|:33453-33687
NuwaII-1_XTro	gb|AAMC02035837.1|:68499-68902
NuwaII-1_XTro	gb|AAMC02035242.1|:35340-35700
NuwaII-1_XTro	gb|AAMC02035242.1|:622-220
NuwaII-1_XTro	gb|AAMC02035040.1|:39374-39703
NuwaII-1_XTro	gb|AAMC02033743.1|:129296-129690
NuwaII-1_XTro	gb|AAMC02026989.1|:32492-32846
NuwaII-1_XTro	gb|AAMC02026426.1|:19203-18931
NuwaII-1_XTro	gb|AAMC02025461.1|:192725-193074
NuwaII-1_XTro	gb|AAMC02025461.1|:14417-14027
NuwaII-1_XTro	gb|AAMC02021924.1|:92561-92161
NuwaII-1_XTro	gb|AAMC02021915.1|:27966-28377
NuwaII-1_XTro	gb|AAMC02021915.1|:12847-12468
NuwaII-1_XTro	gb|AAMC02021349.1|:58943-58553
NuwaII-1_XTro	gb|AAMC02019305.1|:9676-10045
NuwaII-1_XTro	gb|AAMC02019051.1|:756-372
NuwaII-1_XTro	gb|AAMC02016728.1|:3586-3215
NuwaII-1_XTro	gb|AAMC02015404.1|:14969-14608
NuwaII-1_XTro	gb|AAMC02014463.1|:63477-63099
NuwaII-1_XTro	gb|AAMC02014463.1|:19320-16404
NuwaII-1_XTro	gb|AAMC02010513.1|:65564-65335
NuwaII-1_XTro	gb|AAMC02008456.1|:10398-10163
NuwaII-1_XTro	gb|AAMC02008235.1|:21972-21698
NuwaII-1_XTro	gb|AAMC02000988.1|:42780-44201
NuwaII-1_XTro	gb|AAMC02046014.1|:1530-1219
NuwaII-1_XTro	gb|AAMC02043215.1|:3958-4358
NuwaII-1_XTro	gb|AAMC02038108.1|:16518-16920
NuwaII-1_XTro	gb|AAMC02038108.1|:27583-27974
NuwaII-1_XTro	gb|AAMC02036107.1|:39212-39591

NuwaII-1_XTro	gb|AAMC02035457.1|:67193-66816
NuwaII-1_XTro	gb|AAMC02034218.1|:16509-16857
NuwaII-1_XTro	gb|AAMC02034218.1|:109804-110183
NuwaII-1_XTro	gb|AAMC02033671.1|:75294-75626
NuwaII-1_XTro	gb|AAMC02031404.1|:2300-2684
NuwaII-1_XTro	gb|AAMC02031404.1|:17206-17546
NuwaII-1_XTro	gb|AAMC02030443.1|:60881-60560
NuwaII-1_XTro	gb|AAMC02029780.1|:6711-6341
NuwaII-1_XTro	gb|AAMC02027751.1|:115803-116121
NuwaII-1_XTro	gb|AAMC02027751.1|:146627-147031
NuwaII-1_XTro	gb|AAMC02027342.1|:503-856
NuwaII-1_XTro	gb|AAMC02027338.1|:62771-63115
NuwaII-1_XTro	gb|AAMC02026910.1|:100903-100602
NuwaII-1_XTro	gb|AAMC02025162.1|:17144-17507
NuwaII-1_XTro	gb|AAMC02025162.1|:242171-241771
NuwaII-1_XTro	gb|AAMC02024985.1|:16518-16117
NuwaII-1_XTro	gb|AAMC02024033.1|:37350-36908
NuwaII-1_XTro	gb|AAMC02020876.1|:36172-35796
NuwaII-2_XTro	gb|AAMC02010330.1|:3243-2936
NuwaII-2_XTro	gb|AAMC02015608.1|:11764-11323
NuwaII-2_XTro	gb|AAMC02033007.1|:61132-61490
NuwaII-2_XTro	gb|AAMC02033007.1|:72526-72805
NuwaII-2_XTro	gb|AAMC02006937.1|:15571-15125
NuwaII-2_XTro	gb|AAMC02003568.1|:58045-58376
NuwaII-2_XTro	gb|AAMC02018428.1|:71851-72231
NuwaII-2_XTro	gb|AAMC02005843.1|:105735-105425
NuwaII-2_XTro	gb|AAMC02005843.1|:20158-19622
NuwaII-2_XTro	gb|AAMC02022216.1|:70055-70539
NuwaII-2_XTro	gb|AAMC02022216.1|:75566-73947
NuwaII-2_XTro	gb|AAMC02022216.1|:2371-2155
NuwaII-2_XTro	gb|AAMC02036645.1|:95140-95429
NuwaII-2_XTro	gb|AAMC02036645.1|:133966-134341
NuwaII-2_XTro	gb|AAMC02032546.1|:110233-110690
NuwaII-2_XTro	gb|AAMC02032546.1|:77283-76974
NuwaII-2_XTro	gb|AAMC02029997.1|:91772-92057
NuwaII-2_XTro	gb|AAMC02029997.1|:110494-109875
NuwaII-2_XTro	gb|AAMC02025941.1|:54077-53763
NuwaII-2_XTro	gb|AAMC02004725.1|:5828-6847
NuwaII-2_XTro	gb|AAMC02018800.1|:83835-84291
NuwaII-2_XTro	gb|AAMC02018800.1|:99131-99507
NuwaII-2_XTro	gb|AAMC02018800.1|:135392-145672
NuwaII-2_XTro	gb|AAMC02017098.1|:54825-54511
NuwaII-2_XTro	gb|AAMC02004573.1|:21025-21432
NuwaII-2_XTro	gb|AAMC02001216.1|:60702-61238

NuwaII-2_XTro	gb|AAMC02002416.1|:42732-43301
NuwaII-2_XTro	gb|AAMC02002416.1|:200491-198950
NuwaII-2_XTro	gb|AAMC02018173.1|:185556-185109
NuwaII-2_XTro	gb|AAMC02018173.1|:13313-12882
NuwaII-2_XTro	gb|AAMC02029716.1|:132676-133124
NuwaII-2_XTro	gb|AAMC02029716.1|:143182-142766
NuwaII-2_XTro	gb|AAMC02010695.1|:117669-117371
NuwaII-2_XTro	gb|AAMC02008244.1|:123878-124821
NuwaII-2_XTro	gb|AAMC02010697.1|:20428-21151
NuwaII-2_XTro	gb|AAMC02010697.1|:118526-117880
NuwaII-2_XTro	gb|AAMC02010697.1|:58502-57976
NuwaII-2_XTro	gb|AAMC02019235.1|:5155-4697
NuwaII-2_XTro	gb|AAMC02032582.1|:157917-158534
NuwaII-2_XTro	gb|AAMC02032404.1|:91477-92348
NuwaII-2_XTro	gb|AAMC02032404.1|:3782-3319
NuwaII-2_XTro	gb|AAMC02018027.1|:40696-41097
NuwaII-2_XTro	gb|AAMC02018364.1|:3955-3649
NuwaII-2_XTro	gb|AAMC02018331.1|:59793-60187
NuwaII-2_XTro	gb|AAMC02017589.1|:31356-30927
NuwaII-2_XTro	gb|AAMC02013904.1|:16541-16850
NuwaII-2_XTro	gb|AAMC02010478.1|:80670-80231
NuwaII-2_XTro	gb|AAMC02011749.1|:117934-116659
NuwaII-2_XTro	gb|AAMC02040391.1|:11498-11753
NuwaII-2_XTro	gb|AAMC02038630.1|:38608-37753
NuwaII-2_XTro	gb|AAMC02032303.1|:93960-94544
NuwaII-2_XTro	gb|AAMC02032303.1|:106789-107278
NuwaII-2_XTro	gb|AAMC02030835.1|:59098-60364
NuwaII-2_XTro	gb|AAMC02029533.1|:5108-4539
NuwaII-2_XTro	gb|AAMC02026433.1|:15487-15097
NuwaII-2_XTro	gb|AAMC02023154.1|:222316-222000
NuwaII-2_XTro	gb|AAMC02007550.1|:30773-31478
NuwaII-2_XTro	gb|AAMC02017970.1|:31605-32093
NuwaII-2_XTro	gb|AAMC02017867.1|:6152-6773
NuwaII-2_XTro	gb|AAMC02017867.1|:49821-50211
NuwaII-2_XTro	gb|AAMC02014756.1|:155064-155381
NuwaII-2_XTro	gb|AAMC02004422.1|:24329-23564
NuwaII-2_XTro	gb|AAMC02023174.1|:50440-42861
NuwaII-2_XTro	gb|AAMC02023114.1|:66987-66647
NuwaII-2_XTro	gb|AAMC02017508.1|:48010-55614
NuwaII-2_XTro	gb|AAMC02005763.1|:16569-16009
NuwaII-2_XTro	gb|AAMC02022765.1|:44024-44440
NuwaII-2_XTro	gb|AAMC02037195.1|:162651-162923
NuwaII-2_XTro	gb|AAMC02037195.1|:39286-38949
NuwaII-2_XTro	gb|AAMC02035138.1|:30156-30433

NuwaII-2_XTro	gb|AAMC02029466.1|:34036-33611
NuwaII-2_XTro	gb|AAMC02026127.1|:82754-82486
NuwaII-2_XTro	gb|AAMC02018473.1|:40784-41196
NuwaII-2_XTro	gb|AAMC02015851.1|:9362-9098
NuwaII-2_XTro	gb|AAMC02014551.1|:83945-85681
NuwaII-2_XTro	gb|AAMC02014551.1|:104897-108671
NuwaII-2_XTro	gb|AAMC02014371.1|:1005-548
NuwaII-2_XTro	gb|AAMC02013825.1|:14006-13681
NuwaII-2_XTro	gb|AAMC02012486.1|:73678-74014
NuwaII-2_XTro	gb|AAMC02012486.1|:136295-135937
NuwaII-2_XTro	gb|AAMC02012486.1|:8067-7672
NuwaII-2_XTro	gb|AAMC02007325.1|:24524-25052
NuwaII-2_XTro	gb|AAMC02007325.1|:38712-39241
NuwaII-2_XTro	gb|AAMC02007325.1|:27906-27633
NuwaII-2_XTro	gb|AAMC02007251.1|:82641-82330
NuwaII-2_XTro	gb|AAMC02002198.1|:149990-149700
NuwaII-2_XTro	gb|AAMC02022938.1|:109886-109520
NuwaII-2_XTro	gb|AAMC02022918.1|:109771-109497
NuwaII-2_XTro	gb|AAMC02022918.1|:64685-64458
NuwaII-2_XTro	gb|AAMC02022180.1|:160994-161504
NuwaII-2_XTro	gb|AAMC02022180.1|:168685-168288
NuwaII-2_XTro	gb|AAMC02022180.1|:133040-132565
NuwaII-2_XTro	gb|AAMC02022180.1|:53723-53206
NuwaII-2_XTro	gb|AAMC02018988.1|:38967-38462
NuwaII-2_XTro	gb|AAMC02017823.1|:11127-11494
NuwaII-2_XTro	gb|AAMC02015421.1|:26283-26695
NuwaII-2_XTro	gb|AAMC02014196.1|:4004-4377
NuwaII-2_XTro	gb|AAMC02042473.1|:19158-9224
NuwaII-2_XTro	gb|AAMC02036167.1|:116088-117182
NuwaII-2_XTro	gb|AAMC02034176.1|:57511-56485
NuwaII-2_XTro	gb|AAMC02033952.1|:168318-167179
NuwaII-2_XTro	gb|AAMC02033424.1|:1171-769
NuwaII-2_XTro	gb|AAMC02033422.1|:333-784
NuwaII-2_XTro	gb|AAMC02033344.1|:53274-53633
NuwaII-2_XTro	gb|AAMC02033344.1|:97442-97796
NuwaII-2_XTro	gb|AAMC02033177.1|:100714-102114
NuwaII-2_XTro	gb|AAMC02032622.1|:613-1811
NuwaII-2_XTro	gb|AAMC02032271.1|:82902-83539
NuwaII-2_XTro	gb|AAMC02032271.1|:112444-108564
NuwaII-2_XTro	gb|AAMC02031042.1|:6633-6086
NuwaII-2_XTro	gb|AAMC02030997.1|:49998-49234
NuwaII-2_XTro	gb|AAMC02030555.1|:106883-106294
NuwaII-2_XTro	gb|AAMC02030509.1|:166940-166320
NuwaII-2_XTro	gb|AAMC02030218.1|:8003-7694

NuwaII-2_XTro	gb|AAMC02028149.1|:32517-32866
NuwaII-2_XTro	gb|AAMC02027304.1|:5506-6069
NuwaII-2_XTro	gb|AAMC02027239.1|:28333-28694
NuwaII-2_XTro	gb|AAMC02027009.1|:2337-2690
NuwaII-2_XTro	gb|AAMC02027009.1|:129551-129907
NuwaII-2_XTro	gb|AAMC02027009.1|:50277-49919
NuwaII-2_XTro	gb|AAMC02026939.1|:74111-79398
NuwaII-2_XTro	gb|AAMC02026700.1|:98536-96865
NuwaII-2_XTro	gb|AAMC02026678.1|:72327-72677
NuwaII-2_XTro	gb|AAMC02026122.1|:72539-72039
NuwaII-2_XTro	gb|AAMC02026027.1|:59869-59511
NuwaII-2_XTro	gb|AAMC02024006.1|:52687-54117
NuwaII-2_XTro	gb|AAMC02023663.1|:32349-32700
NuwaII-2_XTro	gb|AAMC02022934.1|:54378-54058
NuwaII-2_XTro	gb|AAMC02022840.1|:5148-4799
NuwaII-2_XTro	gb|AAMC02022812.1|:185913-185537
NuwaII-2_XTro	gb|AAMC02022252.1|:28926-29277
NuwaII-2_XTro	gb|AAMC02022002.1|:155214-154787
NuwaII-2_XTro	gb|AAMC02019471.1|:3513-3939
NuwaII-2_XTro	gb|AAMC02019092.1|:113743-116153
NuwaII-3_XTro	gb|AAMC02014762.1|:49978-50224
NuwaII-3_XTro	gb|AAMC02014762.1|:50321-49894
NuwaII-3_XTro	gb|AAMC02004603.1|:98466-98897
NuwaII-3_XTro	gb|AAMC02004603.1|:98813-98567
NuwaII-3_XTro	gb|AAMC02021355.1|:97163-96732
NuwaII-3_XTro	gb|AAMC02032173.1|:24312-24763
NuwaII-3_XTro	gb|AAMC02032173.1|:24679-24433
NuwaII-3_XTro	gb|AAMC02018654.1|:2806-3237
NuwaII-3_XTro	gb|AAMC02018654.1|:3153-2907
NuwaII-3_XTro	gb|AAMC02011171.1|:27085-27308
NuwaII-3_XTro	gb|AAMC02011171.1|:124121-124367
NuwaII-3_XTro	gb|AAMC02011171.1|:124468-124037
NuwaII-3_XTro	gb|AAMC02011171.1|:27421-27001
NuwaII-3_XTro	gb|AAMC02027196.1|:6574-6820
NuwaII-3_XTro	gb|AAMC02027196.1|:6941-6490
NuwaII-3_XTro	gb|AAMC02014565.1|:34449-34900
NuwaII-3_XTro	gb|AAMC02014565.1|:34816-34570
NuwaII-3_XTro	gb|AAMC02008434.1|:5956-6476
NuwaII-3_XTro	gb|AAMC02008434.1|:27918-28409
NuwaII-3_XTro	gb|AAMC02008434.1|:42657-42903
NuwaII-3_XTro	gb|AAMC02008434.1|:43024-42573
NuwaII-3_XTro	gb|AAMC02008434.1|:6390-6074
NuwaII-3_XTro	gb|AAMC02021682.1|:82228-82658
NuwaII-3_XTro	gb|AAMC02021682.1|:93976-94500

NuwaII-3_XTro	gb|AAMC02021682.1|:94414-94095
NuwaII-3_XTro	gb|AAMC02021682.1|:82574-82329
NuwaII-3_XTro	gb|AAMC02030302.1|:75893-76139
NuwaII-3_XTro	gb|AAMC02030302.1|:111694-111255
NuwaII-3_XTro	gb|AAMC02030302.1|:76260-75809
NuwaII-3_XTro	gb|AAMC02021326.1|:6530-6981
NuwaII-3_XTro	gb|AAMC02021326.1|:6897-6651
NuwaII-3_XTro	gb|AAMC02018312.1|:72449-72900
NuwaII-3_XTro	gb|AAMC02018312.1|:72816-72568
NuwaII-3_XTro	gb|AAMC02037728.1|:26189-26435
NuwaII-3_XTro	gb|AAMC02037728.1|:26556-26105
NuwaII-3_XTro	gb|AAMC02008194.1|:13611-14062
NuwaII-3_XTro	gb|AAMC02008194.1|:45013-44570
NuwaII-3_XTro	gb|AAMC02008194.1|:13978-13732
NuwaII-3_XTro	gb|AAMC02034190.1|:122789-123037
NuwaII-3_XTro	gb|AAMC02034190.1|:123156-122705
NuwaII-3_XTro	gb|AAMC02030646.1|:50096-50567
NuwaII-3_XTro	gb|AAMC02030646.1|:50483-50237
NuwaII-3_XTro	gb|AAMC02023701.1|:13157-13405
NuwaII-3_XTro	gb|AAMC02023701.1|:13524-13073
NuwaII-3_XTro	gb|AAMC02023175.1|:59239-59487
NuwaII-3_XTro	gb|AAMC02023175.1|:59606-59155
NuwaII-3_XTro	gb|AAMC02023175.1|:26944-26454
NuwaII-3_XTro	gb|AAMC02023021.1|:71864-72110
NuwaII-3_XTro	gb|AAMC02023021.1|:72231-71780
NuwaII-3_XTro	gb|AAMC02019511.1|:135071-135522
NuwaII-3_XTro	gb|AAMC02019511.1|:135438-135192
NuwaII-3_XTro	gb|AAMC02019511.1|:108541-108181
NuwaII-3_XTro	gb|AAMC02019511.1|:37665-37231
NuwaII-3_XTro	gb|AAMC02017237.1|:93754-94026
NuwaII-3_XTro	gb|AAMC02017237.1|:97516-97065
NuwaII-3_XTro	gb|AAMC02014799.1|:15052-15503
NuwaII-3_XTro	gb|AAMC02014799.1|:15419-15173
NuwaII-3_XTro	gb|AAMC02005843.1|:117005-117251
NuwaII-3_XTro	gb|AAMC02005843.1|:117372-116921
NuwaII-3_XTro	gb|AAMC02005843.1|:95031-94499
NuwaII-3_XTro	gb|AAMC02005843.1|:71016-70488
NuwaII-3_XTro	gb|AAMC02003336.1|:45264-45510
NuwaII-3_XTro	gb|AAMC02003336.1|:45631-45180
NuwaII-3_XTro	gb|AAMC02032643.1|:10532-10985
NuwaII-3_XTro	gb|AAMC02032643.1|:10901-10651
NuwaII-3_XTro	gb|AAMC02017615.1|:61964-62212
NuwaII-3_XTro	gb|AAMC02017615.1|:62331-61880
NuwaII-3_XTro	gb|AAMC02001515.1|:54817-55265

NuwaII-3_XTro	gb|AAMC02001515.1|:72434-72706
NuwaII-3_XTro	gb|AAMC02001515.1|:114733-114363
NuwaII-3_XTro	gb|AAMC02001515.1|:72877-72371
NuwaII-3_XTro	gb|AAMC02029457.1|:77693-78004
NuwaII-3_XTro	gb|AAMC02029457.1|:283096-283344
NuwaII-3_XTro	gb|AAMC02029457.1|:283463-283012
NuwaII-3_XTro	gb|AAMC02029457.1|:78154-77630
NuwaII-3_XTro	gb|AAMC02014199.1|:7495-8013
NuwaII-3_XTro	gb|AAMC02014199.1|:49977-50222
NuwaII-3_XTro	gb|AAMC02014199.1|:50344-49893
NuwaII-3_XTro	gb|AAMC02014199.1|:7909-7610
NuwaII-3_XTro	gb|AAMC02011858.1|:40149-40523
NuwaII-3_XTro	gb|AAMC02011858.1|:259343-259794
NuwaII-3_XTro	gb|AAMC02011858.1|:317473-317787
NuwaII-3_XTro	gb|AAMC02011858.1|:317913-317385
NuwaII-3_XTro	gb|AAMC02011858.1|:259710-259464
NuwaII-3_XTro	gb|AAMC02006616.1|:122349-122800
NuwaII-3_XTro	gb|AAMC02006616.1|:240188-240443
NuwaII-3_XTro	gb|AAMC02006616.1|:240564-240125
NuwaII-3_XTro	gb|AAMC02006616.1|:122716-122468
NuwaII-3_XTro	gb|AAMC02005896.1|:94482-94730
NuwaII-3_XTro	gb|AAMC02005896.1|:94849-94398
NuwaII-3_XTro	gb|AAMC02008654.1|:100115-100546
NuwaII-3_XTro	gb|AAMC02008654.1|:143511-143908
NuwaII-3_XTro	gb|AAMC02008654.1|:100462-100216
NuwaII-3_XTro	gb|AAMC02006044.1|:15758-16210
NuwaII-3_XTro	gb|AAMC02006044.1|:20141-15878
NuwaII-3_XTro	gb|AAMC02030687.1|:5382-5817
NuwaII-3_XTro	gb|AAMC02030687.1|:5733-5481
NuwaII-3_XTro	gb|AAMC02029962.1|:18837-19083
NuwaII-3_XTro	gb|AAMC02029962.1|:19204-18742
NuwaII-3_XTro	gb|AAMC02009258.1|:13193-13438
NuwaII-3_XTro	gb|AAMC02009258.1|:13559-13109
NuwaII-3_XTro	gb|AAMC02007591.1|:7207-7726
NuwaII-3_XTro	gb|AAMC02007591.1|:11841-11390
NuwaII-3_XTro	gb|AAMC02003115.1|:2274-2522
NuwaII-3_XTro	gb|AAMC02003115.1|:2641-2190
NuwaII-3_XTro	gb|AAMC02023102.1|:32197-32513
NuwaII-3_XTro	gb|AAMC02023102.1|:62737-63188
NuwaII-3_XTro	gb|AAMC02023102.1|:63104-62858
NuwaII-3_XTro	gb|AAMC02023102.1|:32639-32111
NuwaII-3_XTro	gb|AAMC02033648.1|:41969-42355
NuwaII-3_XTro	gb|AAMC02033648.1|:189012-189464
NuwaII-3_XTro	gb|AAMC02033648.1|:189380-189133

NuwaII-3_XTro	gb|AAMC02022417.1|:10578-10826
NuwaII-3_XTro	gb|AAMC02022417.1|:10945-10494
NuwaII-3_XTro	gb|AAMC02010520.1|:10640-11092
NuwaII-3_XTro	gb|AAMC02010520.1|:11008-10759
NuwaII-3_XTro	gb|AAMC02002174.1|:83281-83733
NuwaII-3_XTro	gb|AAMC02002174.1|:83649-83400
NuwaII-3_XTro	gb|AAMC02029275.1|:1-317
NuwaII-3_XTro	gb|AAMC02029275.1|:436-107
NuwaII-3_XTro	gb|AAMC02008733.1|:80711-80957
NuwaII-3_XTro	gb|AAMC02008733.1|:81078-80627
NuwaII-3_XTro	gb|AAMC02004849.1|:103547-104028
NuwaII-3_XTro	gb|AAMC02004849.1|:118841-119088
NuwaII-3_XTro	gb|AAMC02004849.1|:119207-118746
NuwaII-3_XTro	gb|AAMC02004849.1|:88910-88420
NuwaII-3_XTro	gb|AAMC02014811.1|:1506-1063
NuwaII-3_XTro	gb|AAMC02017675.1|:98137-98589
NuwaII-3_XTro	gb|AAMC02017675.1|:98505-98258
NuwaII-3_XTro	gb|AAMC02017675.1|:22292-21723
NuwaII-3_XTro	gb|AAMC02029729.1|:54654-54901
NuwaII-3_XTro	gb|AAMC02029729.1|:55020-54570
NuwaII-3_XTro	gb|AAMC02029729.1|:18845-18455
NuwaII-3_XTro	gb|AAMC02028936.1|:19830-20249
NuwaII-3_XTro	gb|AAMC02028936.1|:20197-19950
NuwaII-3_XTro	gb|AAMC02026774.1|:2312-2565
NuwaII-3_XTro	gb|AAMC02026774.1|:2685-2228
NuwaII-3_XTro	gb|AAMC02015671.1|:10887-11338
NuwaII-3_XTro	gb|AAMC02015671.1|:11254-11008
NuwaII-3_XTro	gb|AAMC02015316.1|:10836-11287
NuwaII-3_XTro	gb|AAMC02015316.1|:11203-10957
NuwaII-3_XTro	gb|AAMC02014792.1|:26159-26406
NuwaII-3_XTro	gb|AAMC02014792.1|:26527-26075
NuwaII-3_XTro	gb|AAMC02014761.1|:11056-11302
NuwaII-3_XTro	gb|AAMC02014761.1|:11423-10972
NuwaII-3_XTro	gb|AAMC02013978.1|:3377-3626
NuwaII-3_XTro	gb|AAMC02013978.1|:3926-3293
NuwaII-3_XTro	gb|AAMC02022406.1|:2982-8954
NuwaII-3_XTro	gb|AAMC02022406.1|:3349-2898
NuwaII-3_XTro	gb|AAMC02013103.1|:42024-42673
NuwaII-3_XTro	gb|AAMC02013103.1|:42780-42294
NuwaII-3_XTro	gb|AAMC02013103.1|:3599-3149
NuwaII-3_XTro	gb|AAMC02002647.1|:11491-11738
NuwaII-3_XTro	gb|AAMC02002647.1|:11839-11407
NuwaII-3_XTro	gb|AAMC02001379.1|:18232-18480
NuwaII-3_XTro	gb|AAMC02014112.1|:28517-28968

NuwaII-3_XTro	gb|AAMC02014112.1|:28884-28638
NuwaII-3_XTro	gb|AAMC02006035.1|:51583-52014
NuwaII-3_XTro	gb|AAMC02006035.1|:102072-102390
NuwaII-3_XTro	gb|AAMC02006035.1|:115965-115526
NuwaII-3_XTro	gb|AAMC02006035.1|:102509-101988
NuwaII-3_XTro	gb|AAMC02006035.1|:51930-51684
NuwaII-3_XTro	gb|AAMC02014924.1|:2616-2863
NuwaII-3_XTro	gb|AAMC02014924.1|:35674-35984
NuwaII-3_XTro	gb|AAMC02014924.1|:9184-8656
NuwaII-3_XTro	gb|AAMC02030555.1|:28579-29032
NuwaII-3_XTro	gb|AAMC02030555.1|:28948-28699
NuwaII-3_XTro	gb|AAMC02025377.1|:51036-51284
NuwaII-3_XTro	gb|AAMC02025377.1|:51403-50952
NuwaII-3_XTro	gb|AAMC02023643.1|:98401-98853
NuwaII-3_XTro	gb|AAMC02023643.1|:98769-98522
NuwaII-3_XTro	gb|AAMC02018173.1|:153933-154465
NuwaII-3_XTro	gb|AAMC02018173.1|:197504-197752
NuwaII-3_XTro	gb|AAMC02018173.1|:197871-197420
NuwaII-3_XTro	gb|AAMC02018028.1|:3379-3625
NuwaII-3_XTro	gb|AAMC02018028.1|:3746-3295
NuwaII-3_XTro	gb|AAMC02016758.1|:39705-39954
NuwaII-3_XTro	gb|AAMC02016758.1|:40073-39621
NuwaII-3_XTro	gb|AAMC02022706.1|:81164-81407
NuwaII-3_XTro	gb|AAMC02022706.1|:81530-81089
NuwaII-3_XTro	gb|AAMC02003789.1|:89715-90145
NuwaII-3_XTro	gb|AAMC02003789.1|:130266-129840
NuwaII-3_XTro	gb|AAMC02033542.1|:31765-31313
NuwaII-3_XTro	gb|AAMC02030791.1|:175173-181343
NuwaII-3_XTro	gb|AAMC02030791.1|:181259-175030
NuwaII-3_XTro	gb|AAMC02029678.1|:103598-104052
NuwaII-3_XTro	gb|AAMC02029678.1|:103966-103720
NuwaII-3_XTro	gb|AAMC02014901.1|:163384-163819
NuwaII-3_XTro	gb|AAMC02014901.1|:194940-194621
NuwaII-3_XTro	gb|AAMC02014901.1|:163735-163485
NuwaII-3_XTro	gb|AAMC02004370.1|:79815-80266
NuwaII-3_XTro	gb|AAMC02004370.1|:80182-79936
NuwaII-3_XTro	gb|AAMC02001743.1|:35471-35721
NuwaII-3_XTro	gb|AAMC02001743.1|:35820-35385
NuwaII-3_XTro	gb|AAMC02001135.1|:95830-96076
NuwaII-3_XTro	gb|AAMC02001135.1|:96197-95746
NuwaII-3_XTro	gb|AAMC02001682.1|:5226-5473
NuwaII-3_XTro	gb|AAMC02001682.1|:5572-5142
NuwaII-3_XTro	gb|AAMC02004100.1|:29200-29642
NuwaII-3_XTro	gb|AAMC02004100.1|:29558-29312

NuwaII-3_XTro	gb|AAMC02003410.1|:16304-16553
NuwaII-3_XTro	gb|AAMC02003410.1|:16672-16220
NuwaII-3_XTro	gb|AAMC02001248.1|:1234-1482
NuwaII-3_XTro	gb|AAMC02001248.1|:1601-1150
NuwaII-3_XTro	gb|AAMC02025220.1|:46071-46466
NuwaII-3_XTro	gb|AAMC02032621.1|:72975-73426
NuwaII-3_XTro	gb|AAMC02032621.1|:81379-80889
NuwaII-3_XTro	gb|AAMC02022710.1|:71175-71637
NuwaII-3_XTro	gb|AAMC02022710.1|:71542-71296
NuwaII-3_XTro	gb|AAMC02022710.1|:51228-50799
NuwaII-3_XTro	gb|AAMC02014198.1|:34161-34410
NuwaII-3_XTro	gb|AAMC02014198.1|:34509-34077
NuwaII-3_XTro	gb|AAMC02014292.1|:5277-5729
NuwaII-3_XTro	gb|AAMC02014292.1|:5645-5398
NuwaII-3_XTro	gb|AAMC02035350.1|:71107-71355
NuwaII-3_XTro	gb|AAMC02035350.1|:71474-71023
NuwaII-3_XTro	gb|AAMC02026917.1|:12007-12457
NuwaII-3_XTro	gb|AAMC02026917.1|:12373-12128
NuwaII-3_XTro	gb|AAMC02025882.1|:10027-10274
NuwaII-3_XTro	gb|AAMC02025882.1|:10394-9943
NuwaII-3_XTro	gb|AAMC02022131.1|:78033-78484
NuwaII-3_XTro	gb|AAMC02022131.1|:78400-78153
NuwaII-3_XTro	gb|AAMC02003001.1|:28877-29126
NuwaII-3_XTro	gb|AAMC02003001.1|:29245-28793
NuwaII-3_XTro	gb|AAMC02002524.1|:6452-6698
NuwaII-3_XTro	gb|AAMC02002524.1|:6799-6368
NuwaII-3_XTro	gb|AAMC02001930.1|:15150-15395
NuwaII-3_XTro	gb|AAMC02001930.1|:15506-15066
NuwaII-3_XTro	gb|AAMC02001905.1|:27272-27799
NuwaII-3_XTro	gb|AAMC02001905.1|:40899-41150
NuwaII-3_XTro	gb|AAMC02001905.1|:41269-40815
NuwaII-3_XTro	gb|AAMC02001905.1|:27715-27397
NuwaII-3_XTro	gb|AAMC02022380.1|:7307-7558
NuwaII-3_XTro	gb|AAMC02022380.1|:7659-7223
NuwaII-3_XTro	gb|AAMC02026760.1|:96448-96931
NuwaII-3_XTro	gb|AAMC02026760.1|:181252-181497
NuwaII-3_XTro	gb|AAMC02026760.1|:181618-181168
NuwaII-3_XTro	gb|AAMC02026760.1|:96846-96568
NuwaII-3_XTro	gb|AAMC02022414.1|:89279-89806
NuwaII-3_XTro	gb|AAMC02022414.1|:111277-111728
NuwaII-3_XTro	gb|AAMC02022414.1|:111644-111398
NuwaII-3_XTro	gb|AAMC02026356.1|:216537-216991
NuwaII-3_XTro	gb|AAMC02026356.1|:216907-216656
NuwaII-3_XTro	gb|AAMC02025378.1|:88676-89128

NuwaII-3_XTro	gb|AAMC02025378.1|:89044-88795
NuwaII-3_XTro	gb|AAMC02021834.1|:30647-31062
NuwaII-3_XTro	gb|AAMC02021834.1|:60102-59633
NuwaII-3_XTro	gb|AAMC02001551.1|:35408-34956
NuwaII-3_XTro	gb|AAMC02033205.1|:60904-61355
NuwaII-3_XTro	gb|AAMC02033205.1|:61271-61023
NuwaII-3_XTro	gb|AAMC02037325.1|:44793-45262
NuwaII-3_XTro	gb|AAMC02037325.1|:45199-44925
NuwaII-3_XTro	gb|AAMC02037325.1|:25075-24630
NuwaII-3_XTro	gb|AAMC02029846.1|:118368-118615
NuwaII-3_XTro	gb|AAMC02029846.1|:138993-139459
NuwaII-3_XTro	gb|AAMC02029846.1|:118736-118284
NuwaII-3_XTro	gb|AAMC02014157.1|:100287-100719
NuwaII-3_XTro	gb|AAMC02014157.1|:100635-100386
NuwaII-3_XTro	gb|AAMC02011792.1|:28512-28968
NuwaII-3_XTro	gb|AAMC02011792.1|:28884-28631
NuwaII-3_XTro	gb|AAMC02030719.1|:109152-109663
NuwaII-3_XTro	gb|AAMC02030719.1|:122286-122604
NuwaII-3_XTro	gb|AAMC02030719.1|:234402-234853
NuwaII-3_XTro	gb|AAMC02030719.1|:234769-234523
NuwaII-3_XTro	gb|AAMC02030719.1|:122730-122202
NuwaII-3_XTro	gb|AAMC02021425.1|:430-885
NuwaII-3_XTro	gb|AAMC02021425.1|:117488-117874
NuwaII-3_XTro	gb|AAMC02021425.1|:801-549
NuwaII-3_XTro	gb|AAMC02011657.1|:24499-24751
NuwaII-3_XTro	gb|AAMC02011657.1|:24788-24415
NuwaII-3_XTro	gb|AAMC02001309.1|:2874-3309
NuwaII-3_XTro	gb|AAMC02001309.1|:3225-2975
NuwaII-3_XTro	gb|AAMC02012121.1|:29343-29799
NuwaII-3_XTro	gb|AAMC02012121.1|:29715-29464
NuwaII-3_XTro	gb|AAMC02015305.1|:52160-52615
NuwaII-3_XTro	gb|AAMC02015305.1|:52531-49884
NuwaII-3_XTro	gb|AAMC02007133.1|:22753-23203
NuwaII-3_XTro	gb|AAMC02007133.1|:23119-22874
NuwaII-3_XTro	gb|AAMC02006334.1|:26394-26644
NuwaII-3_XTro	gb|AAMC02006334.1|:26765-26310
NuwaII-3_XTro	gb|AAMC02034194.1|:81145-81390
NuwaII-3_XTro	gb|AAMC02034194.1|:81511-81061
NuwaII-3_XTro	gb|AAMC02032587.1|:26729-27181
NuwaII-3_XTro	gb|AAMC02032587.1|:27097-26850
NuwaII-3_XTro	gb|AAMC02030778.1|:98477-98726
NuwaII-3_XTro	gb|AAMC02030778.1|:98845-98393
NuwaII-3_XTro	gb|AAMC02006826.1|:95065-95381
NuwaII-3_XTro	gb|AAMC02006826.1|:111661-111905

NuwaII-3_XTro	gb|AAMC02006826.1|:112026-111577
NuwaII-3_XTro	gb|AAMC02006826.1|:95507-94979
NuwaII-3_XTro	gb|AAMC02006189.1|:77847-78092
NuwaII-3_XTro	gb|AAMC02006189.1|:183025-182615
NuwaII-3_XTro	gb|AAMC02006189.1|:78213-77763
NuwaII-3_XTro	gb|AAMC02015554.1|:25683-26138
NuwaII-3_XTro	gb|AAMC02015554.1|:26054-25804
NuwaII-3_XTro	gb|AAMC02014291.1|:23739-23989
NuwaII-3_XTro	gb|AAMC02014291.1|:31724-23655
NuwaII-3_XTro	gb|AAMC02029032.1|:90168-90618
NuwaII-3_XTro	gb|AAMC02029032.1|:168404-168007
NuwaII-3_XTro	gb|AAMC02029032.1|:90534-90287
NuwaII-3_XTro	gb|AAMC02027115.1|:59556-60010
NuwaII-3_XTro	gb|AAMC02027115.1|:116620-116192
NuwaII-3_XTro	gb|AAMC02027115.1|:59926-52579
NuwaII-3_XTro	gb|AAMC02023174.1|:42945-43195
NuwaII-3_XTro	gb|AAMC02023174.1|:43316-42861
NuwaII-3_XTro	gb|AAMC02006850.1|:224754-225004
NuwaII-3_XTro	gb|AAMC02006850.1|:261199-261658
NuwaII-3_XTro	gb|AAMC02006850.1|:261581-261324
NuwaII-3_XTro	gb|AAMC02006850.1|:225125-224670
NuwaII-3_XTro	gb|AAMC02002730.1|:74163-77676
NuwaII-3_XTro	gb|AAMC02002730.1|:125060-125479
NuwaII-3_XTro	gb|AAMC02002730.1|:125395-125159
NuwaII-3_XTro	gb|AAMC02002730.1|:77592-74097
NuwaII-3_XTro	gb|AAMC02001727.1|:32476-32931
NuwaII-3_XTro	gb|AAMC02001727.1|:32847-32597
NuwaII-3_XTro	gb|AAMC02014749.1|:120582-120830
NuwaII-3_XTro	gb|AAMC02008738.1|:35257-35501
NuwaII-3_XTro	gb|AAMC02008738.1|:35622-35173
NuwaII-3_XTro	gb|AAMC02003660.1|:22774-23185
NuwaII-3_XTro	gb|AAMC02003660.1|:23101-22859
NuwaII-3_XTro	gb|AAMC02021349.1|:204006-204456
NuwaII-3_XTro	gb|AAMC02021349.1|:204372-204127
NuwaII-3_XTro	gb|AAMC02016119.1|:13184-13436
NuwaII-3_XTro	gb|AAMC02016119.1|:45073-45581
NuwaII-3_XTro	gb|AAMC02016119.1|:49001-48641
NuwaII-3_XTro	gb|AAMC02016119.1|:13473-13100
NuwaII-3_XTro	gb|AAMC02013797.1|:63725-64180
NuwaII-3_XTro	gb|AAMC02013797.1|:64096-63844
NuwaII-3_XTro	gb|AAMC02006675.1|:55320-55570
NuwaII-3_XTro	gb|AAMC02006675.1|:55911-55236
NuwaII-3_XTro	gb|AAMC02003426.1|:120193-120445
NuwaII-3_XTro	gb|AAMC02003426.1|:120564-120109

NuwaII-3_XTro	gb|AAMC02036318.1|:63384-63629
NuwaII-3_XTro	gb|AAMC02036318.1|:63750-63300
NuwaII-3_XTro	gb|AAMC02016150.1|:29778-30232
NuwaII-3_XTro	gb|AAMC02016150.1|:30148-29899
NuwaII-3_XTro	gb|AAMC02019396.1|:17816-18342
NuwaII-3_XTro	gb|AAMC02019396.1|:309612-309909
NuwaII-3_XTro	gb|AAMC02019396.1|:386987-387479
NuwaII-3_XTro	gb|AAMC02019396.1|:310034-309548
NuwaII-3_XTro	gb|AAMC02019396.1|:88804-88412
NuwaII-3_XTro	gb|AAMC02019396.1|:18237-17955
NuwaII-3_XTro	gb|AAMC02012432.1|:42450-42702
NuwaII-3_XTro	gb|AAMC02012432.1|:42821-42366
NuwaII-3_XTro	gb|AAMC02002986.1|:23399-23649
NuwaII-3_XTro	gb|AAMC02002986.1|:23771-23315
NuwaII-3_XTro	gb|AAMC02030628.1|:44558-51635
NuwaII-3_XTro	gb|AAMC02030628.1|:51551-44495
NuwaII-3_XTro	gb|AAMC02019367.1|:3179-3423
NuwaII-3_XTro	gb|AAMC02019367.1|:3524-3095
NuwaII-3_XTro	gb|AAMC02011864.1|:126156-126607
NuwaII-3_XTro	gb|AAMC02011864.1|:126523-126277
NuwaII-3_XTro	gb|AAMC02004027.1|:5987-6442
NuwaII-3_XTro	gb|AAMC02004027.1|:6358-6108
NuwaII-3_XTro	gb|AAMC02003344.1|:132325-132575
NuwaII-3_XTro	gb|AAMC02003344.1|:132696-132241
NuwaII-3_XTro	gb|AAMC02002128.1|:25511-25761
NuwaII-3_XTro	gb|AAMC02002128.1|:25882-25427
NuwaII-3_XTro	gb|AAMC02006040.1|:121293-121537
NuwaII-3_XTro	gb|AAMC02006040.1|:284865-285393
NuwaII-3_XTro	gb|AAMC02006040.1|:285307-284991
NuwaII-3_XTro	gb|AAMC02006040.1|:121656-121209
NuwaII-3_XTro	gb|AAMC02006040.1|:33645-33155
NuwaII-3_XTro	gb|AAMC02022036.1|:65894-66350
NuwaII-3_XTro	gb|AAMC02022036.1|:66266-66013
NuwaII-3_XTro	gb|AAMC02022036.1|:32133-31631
NuwaII-3_XTro	gb|AAMC02032938.1|:5667-5916
NuwaII-3_XTro	gb|AAMC02022837.1|:10489-10806
NuwaII-3_XTro	gb|AAMC02022837.1|:195253-194964
NuwaII-3_XTro	gb|AAMC02022837.1|:10905-10405
NuwaII-3_XTro	gb|AAMC02011519.1|:4808-5264
NuwaII-3_XTro	gb|AAMC02011519.1|:5180-4929
NuwaII-3_XTro	gb|AAMC02012095.1|:157166-157410
NuwaII-3_XTro	gb|AAMC02012095.1|:157529-157082
NuwaII-3_XTro	gb|AAMC02005263.1|:190674-190916
NuwaII-3_XTro	gb|AAMC02005263.1|:191043-190590

NuwaII-3_XTro	gb|AAMC02018318.1|:17376-17732
NuwaII-3_XTro	gb|AAMC02037393.1|:30761-30995
NuwaII-3_XTro	gb|AAMC02026037.1|:127114-128617
NuwaII-3_XTro	gb|AAMC02026037.1|:273289-272774
NuwaII-3_XTro	gb|AAMC02026037.1|:237637-237248
NuwaII-3_XTro	gb|AAMC02026037.1|:127520-127030
NuwaII-3_XTro	gb|AAMC02021544.1|:52527-52772
NuwaII-3_XTro	gb|AAMC02014414.1|:77163-77661
NuwaII-3_XTro	gb|AAMC02003481.1|:295-1
NuwaII-3_XTro	gb|AAMC02001840.1|:53307-53797
NuwaII-3_XTro	gb|AAMC02023265.1|:16692-22193
NuwaII-3_XTro	gb|AAMC02023265.1|:22314-21875
NuwaII-3_XTro	gb|AAMC02015526.1|:230892-231190
NuwaII-3_XTro	gb|AAMC02015526.1|:231318-230808
NuwaII-3_XTro	gb|AAMC02015526.1|:98183-97694
NuwaII-3_XTro	gb|AAMC02015526.1|:16956-16473
NuwaII-3_XTro	gb|AAMC02012195.1|:75072-75581
NuwaII-3_XTro	gb|AAMC02022181.1|:632-877
NuwaII-3_XTro	gb|AAMC02022181.1|:996-546
NuwaII-3_XTro	gb|AAMC02012468.1|:143986-144237
NuwaII-3_XTro	gb|AAMC02012468.1|:144358-143902
NuwaII-3_XTro	gb|AAMC02002277.1|:6785-7064
NuwaII-3_XTro	gb|AAMC02014145.1|:11252-11733
NuwaII-3_XTro	gb|AAMC02001809.1|:42614-42901
NuwaII-3_XTro	gb|AAMC02001809.1|:42901-33132
NuwaII-3_XTro	gb|AAMC02033285.1|:2768-3224
NuwaII-3_XTro	gb|AAMC02033285.1|:3140-2887
NuwaII-3_XTro	gb|AAMC02021346.1|:44146-44382
NuwaII-3_XTro	gb|AAMC02021346.1|:44501-44062
NuwaII-3_XTro	gb|AAMC02012094.1|:71218-71455
NuwaII-3_XTro	gb|AAMC02012094.1|:71574-71134
NuwaII-3_XTro	gb|AAMC02001373.1|:33812-34183
NuwaII-3_XTro	gb|AAMC02006977.1|:49666-50006
NuwaII-3_XTro	gb|AAMC02006977.1|:12355-11963
NuwaII-3_XTro	gb|AAMC02019598.1|:14998-15341
NuwaII-3_XTro	gb|AAMC02019598.1|:30735-31201
NuwaII-3_XTro	gb|AAMC02019598.1|:31138-30866
NuwaII-3_XTro	gb|AAMC02033334.1|:3109-3535
NuwaII-3_XTro	gb|AAMC02033334.1|:3451-3227
NuwaII-3_XTro	gb|AAMC02027150.1|:60729-61081
NuwaII-3_XTro	gb|AAMC02017371.1|:38655-38966
NuwaII-3_XTro	gb|AAMC02017371.1|:73397-73623
NuwaII-3_XTro	gb|AAMC02017371.1|:73623-73313
NuwaII-3_XTro	gb|AAMC02017371.1|:39158-38592

NuwaII-3_XTro	gb|AAMC02005882.1|:36778-37015
NuwaII-3_XTro	gb|AAMC02005882.1|:37052-36694
NuwaII-3_XTro	gb|AAMC02008967.1|:18670-19102
NuwaII-3_XTro	gb|AAMC02039015.1|:1654-1905
NuwaII-3_XTro	gb|AAMC02039015.1|:2029-1570
NuwaII-3_XTro	gb|AAMC02007482.1|:44783-45009
NuwaII-3_XTro	gb|AAMC02018951.1|:105135-105565
NuwaII-3_XTro	gb|AAMC02018951.1|:105481-105246
NuwaII-3_XTro	gb|AAMC02018444.1|:130314-130673
NuwaII-3_XTro	gb|AAMC02013868.1|:4687-4944
NuwaII-3_XTro	gb|AAMC02013868.1|:5075-4624
NuwaII-3_XTro	gb|AAMC02005645.1|:17266-17484
NuwaII-3_XTro	gb|AAMC02005645.1|:17603-17182
NuwaII-3_XTro	gb|AAMC02021620.1|:2905-2511
NuwaII-3_XTro	gb|AAMC02004189.1|:115634-116072
NuwaII-3_XTro	gb|AAMC02004189.1|:115988-115753
NuwaII-3_XTro	gb|AAMC02002518.1|:26772-27009
NuwaII-3_XTro	gb|AAMC02002518.1|:27128-26688
NuwaII-3_XTro	gb|AAMC02038381.1|:2724-12090
NuwaII-3_XTro	gb|AAMC02038381.1|:3247-2661
NuwaII-3_XTro	gb|AAMC02016694.1|:6-290
NuwaII-3_XTro	gb|AAMC02013989.1|:25891-26331
NuwaII-3_XTro	gb|AAMC02013989.1|:26247-26010
NuwaII-3_XTro	gb|AAMC02013437.1|:11116-11468
NuwaII-3_XTro	gb|AAMC02010508.1|:20235-19898
NuwaII-3_XTro	gb|AAMC02022717.1|:22394-21865
NuwaII-3_XTro	gb|AAMC02022717.1|:3894-3428
NuwaII-3_XTro	gb|AAMC02017437.1|:88588-88949
NuwaII-3_XTro	gb|AAMC02017437.1|:88865-88625
NuwaII-3_XTro	gb|AAMC02010543.1|:17011-17515
NuwaII-3_XTro	gb|AAMC02010543.1|:202925-202435
NuwaII-3_XTro	gb|AAMC02010543.1|:85226-84840
NuwaII-3_XTro	gb|AAMC02010543.1|:404-77
NuwaII-3_XTro	gb|AAMC02008261.1|:7693-7223
NuwaII-3_XTro	gb|AAMC02007706.1|:103784-103370
NuwaII-3_XTro	gb|AAMC02019553.1|:83011-83451
NuwaII-3_XTro	gb|AAMC02019553.1|:83367-83130
NuwaII-3_XTro	gb|AAMC02010699.1|:1617-1864
NuwaII-3_XTro	gb|AAMC02010699.1|:1983-1533
NuwaII-3_XTro	gb|AAMC02004375.1|:21265-21583
NuwaII-3_XTro	gb|AAMC02004375.1|:29132-28760
NuwaII-3_XTro	gb|AAMC02035435.1|:17940-18373
NuwaII-3_XTro	gb|AAMC02015670.1|:4067-4511
NuwaII-3_XTro	gb|AAMC02015670.1|:4427-4186

	NuwaII-3_XTro	gb|AAMC02014770.1|:1666-2120
	NuwaII-3_XTro	gb|AAMC02014770.1|:82149-82670
	NuwaII-3_XTro	gb|AAMC02014770.1|:82584-82268
	NuwaII-3_XTro	gb|AAMC02014770.1|:2036-1790
	NuwaII-3_XTro	gb|AAMC02011334.1|:72900-73793
	NuwaII-3_XTro	gb|AAMC02011334.1|:114127-114648
	NuwaII-3_XTro	gb|AAMC02011334.1|:114564-114246
	NuwaII-3_XTro	gb|AAMC02011334.1|:73709-73021
	NuwaII-3_XTro	gb|AAMC02042084.1|:5920-5584
	NuwaII-3_XTro	gb|AAMC02012169.1|:160067-160596
	NuwaII-3_XTro	gb|AAMC02014281.1|:61395-61875
	NuwaII-3_XTro	gb|AAMC02014281.1|:74175-74594
	NuwaII-3_XTro	gb|AAMC02014281.1|:74508-74193
	NuwaII-3_XTro	gb|AAMC02011314.1|:45797-46197
	NuwaII-3_XTro	gb|AAMC02009439.1|:11475-11152
	NuwaII-3_XTro	gb|AAMC02002382.1|:10226-9945
	NuwaII-3_XTro	gb|AAMC02001333.1|:260040-260307
	NuwaII-3_XTro	gb|AAMC02001245.1|:25022-25458
	NuwaII-3_XTro	gb|AAMC02001245.1|:25378-25141
	NuwaII-3_XTro	gb|AAMC02032483.1|:26633-26259
	NuwaII-3_XTro	gb|AAMC02026403.1|:2355-2794
	NuwaII-3_XTro	gb|AAMC02026403.1|:2710-2474
	NuwaII-3_XTro	gb|AAMC02017941.1|:38075-40206
	NuwaII-3_XTro	gb|AAMC02016723.1|:75382-75821
	NuwaII-3_XTro	gb|AAMC02016723.1|:75737-70156
	NuwaII-3_XTro	gb|AAMC02002984.1|:13380-13780
	NuwaII-3_XTro	gb|AAMC02002984.1|:64615-64128
	NuwaII-3_XTro	gb|AAMC02002984.1|:13696-13480
	NuwaII-3_XTro	gb|AAMC02031873.1|:17653-18184
	NuwaII-3_XTro	gb|AAMC02028149.1|:13067-16750
	NuwaII-3_XTro	gb|AAMC02028149.1|:16666-16425
	NuwaII-3_XTro	gb|AAMC02026533.1|:15544-15088
	NuwaII-3_XTro	gb|AAMC02014427.1|:70543-71002
	NuwaII-3_XTro	gb|AAMC02014427.1|:111589-111987
	NuwaII-3_XTro	gb|AAMC02017185.1|:55152-55643
	NuwaII-3_XTro	gb|AAMC02013757.1|:39576-39173
	NuwaII-3_XTro	gb|AAMC02011824.1|:126507-127037
	NuwaII-3_XTro	gb|AAMC02011824.1|:126933-126633
Takifugu rubripes	*NuwaII-1_TRub*	emb|CAAB02008253.1|:636-3832
	NuwaII-1_TRub	emb|CAAB02006227.1|:106507-107922
	NuwaII-1_TRub	emb|CAAB02002286.1|:103-1020
	NuwaII-1_TRub	emb|CAAB02007516.1|:15946-16848
	NuwaII-1_TRub	emb|CAAB02024196.1|:241-1770
	NuwaII-1_TRub	emb|CAAB02004112.1|:20165-21082

NuwaII-1_TRub	emb|CAAB02006021.1|:4219-5139
NuwaII-1_TRub	emb|CAAB02003577.1|:173895-175647
NuwaII-1_TRub	emb|CAAB02007153.1|:13973-14882
NuwaII-1_TRub	emb|CAAB02009949.1|:5938-6832
NuwaII-1_TRub	emb|CAAB02022738.1|:511-1821
NuwaII-1_TRub	emb|CAAB02000010.1|:659-1544
NuwaII-1_TRub	emb|CAAB02021106.1|:1864-2753
NuwaII-1_TRub	emb|CAAB02004857.1|:14240-15137
NuwaII-1_TRub	emb|CAAB02023606.1|:2280-3182
NuwaII-1_TRub	emb|CAAB02012788.1|:3291-4203
NuwaII-1_TRub	emb|CAAB02010003.1|:1115-2017
NuwaII-1_TRub	emb|CAAB02009738.1|:38840-39829
NuwaII-1_TRub	emb|CAAB02014720.1|:13340-14234
NuwaII-1_TRub	emb|CAAB02011270.1|:1828-2701
NuwaII-1_TRub	emb|CAAB02010789.1|:32852-33756
NuwaII-1_TRub	emb|CAAB02013492.1|:9883-10745
NuwaII-1_TRub	emb|CAAB02009254.1|:18638-19509
NuwaII-1_TRub	emb|CAAB02001107.1|:14358-15148
NuwaII-1_TRub	emb|CAAB02013704.1|:3005-3869
NuwaII-1_TRub	emb|CAAB02001801.1|:41348-42216
NuwaII-1_TRub	emb|CAAB02004233.1|:103694-104588
NuwaII-1_TRub	emb|CAAB02005975.1|:18966-19849
NuwaII-1_TRub	emb|CAAB02007618.1|:8937-9830
NuwaII-1_TRub	emb|CAAB02003133.1|:3919-4743
NuwaII-1_TRub	emb|CAAB02009893.1|:1537-2421
NuwaII-1_TRub	emb|CAAB02014132.1|:334-1101
NuwaII-1_TRub	emb|CAAB02007194.1|:28293-29119
NuwaII-1_TRub	emb|CAAB02011730.1|:27747-28634
NuwaII-1_TRub	emb|CAAB02010008.1|:62-938
NuwaII-1_TRub	emb|CAAB02030082.1|:207-1078
NuwaII-1_TRub	emb|CAAB02011074.1|:5785-6605
NuwaII-1_TRub	emb|CAAB02019420.1|:1-837
NuwaII-1_TRub	emb|CAAB02007190.1|:45312-46192
NuwaII-1_TRub	emb|CAAB02002087.1|:26945-27743
NuwaII-1_TRub	emb|CAAB02017971.1|:2786-3451
NuwaII-1_TRub	emb|CAAB02002413.1|:48366-49190
NuwaII-1_TRub	emb|CAAB02000545.1|:173284-174090
NuwaII-1_TRub	emb|CAAB02024661.1|:7-766
NuwaII-1_TRub	emb|CAAB02011753.1|:32731-33856
NuwaII-1_TRub	emb|CAAB02017878.1|:1273-2018
NuwaII-1_TRub	emb|CAAB02010201.1|:1-872
NuwaII-1_TRub	emb|CAAB02010884.1|:10476-12745
NuwaII-1_TRub	emb|CAAB02006300.1|:20846-22217
NuwaII-1_TRub	emb|CAAB02005069.1|:102061-102729

NuwaII-1_TRub	emb|CAAB02006599.1|:25000-25743
NuwaII-1_TRub	emb|CAAB02018276.1|:2224-2947
NuwaII-1_TRub	emb|CAAB02017078.1|:5287-5992
NuwaII-1_TRub	emb|CAAB02007091.1|:20980-21622
NuwaII-1_TRub	emb|CAAB02013272.1|:11257-12124
NuwaII-1_TRub	emb|CAAB02008855.1|:26532-27203
NuwaII-1_TRub	emb|CAAB02029400.1|:262-1170
NuwaII-1_TRub	emb|CAAB02013867.1|:4929-5698
NuwaII-1_TRub	emb|CAAB02003435.1|:998-1599
NuwaII-1_TRub	emb|CAAB02018359.1|:65-639
NuwaII-1_TRub	emb|CAAB02027448.1|:1096-1834
NuwaII-1_TRub	emb|CAAB02008134.1|:45537-46312
NuwaII-1_TRub	emb|CAAB02010347.1|:1348-2107
NuwaII-1_TRub	emb|CAAB02011805.1|:4465-5064
NuwaII-1_TRub	emb|CAAB02029481.1|:132-740
NuwaII-1_TRub	emb|CAAB02018018.1|:125-733
NuwaII-1_TRub	emb|CAAB02011520.1|:4720-7112
NuwaII-1_TRub	emb|CAAB02016003.1|:1-750
NuwaII-1_TRub	emb|CAAB02004439.1|:2847-3433
NuwaII-1_TRub	emb|CAAB02013788.1|:6319-7104
NuwaII-1_TRub	emb|CAAB02018673.1|:887-1412
NuwaII-1_TRub	emb|CAAB02028761.1|:116-641
NuwaII-1_TRub	emb|CAAB02019562.1|:466-991
NuwaII-1_TRub	emb|CAAB02021554.1|:855-1392
NuwaII-1_TRub	emb|CAAB02015809.1|:85-767
NuwaII-1_TRub	emb|CAAB02006227.1|:124030-124913
NuwaII-1_TRub	emb|CAAB02012744.1|:8703-9339
NuwaII-1_TRub	emb|CAAB02006025.1|:29557-30619
NuwaII-1_TRub	emb|CAAB02020303.1|:3936-4643
NuwaII-1_TRub	emb|CAAB02011876.1|:714-1272
NuwaII-1_TRub	emb|CAAB02017450.1|:6244-6781
NuwaII-1_TRub	emb|CAAB02004463.1|:10212-10758
NuwaII-1_TRub	emb|CAAB02019746.1|:1-512
NuwaII-1_TRub	emb|CAAB02014551.1|:919-1514
NuwaII-1_TRub	emb|CAAB02008627.1|:43379-43858
NuwaII-1_TRub	emb|CAAB02007671.1|:13214-13652
NuwaII-1_TRub	emb|CAAB02016441.1|:5794-6217
NuwaII-1_TRub	emb|CAAB02021660.1|:54-482
NuwaII-1_TRub	emb|CAAB02006848.1|:6292-6974
NuwaII-1_TRub	emb|CAAB02004895.1|:709-1157
NuwaII-1_TRub	emb|CAAB02016044.1|:4649-5097
NuwaII-1_TRub	emb|CAAB02022573.1|:479-934
NuwaII-1_TRub	emb|CAAB02007316.1|:63128-63548
NuwaII-1_TRub	emb|CAAB02011641.1|:8005-8418

NuwaII-1_TRub	emb|CAAB02002038.1|:13607-15190
NuwaII-1_TRub	emb|CAAB02000945.1|:224947-225448
NuwaII-1_TRub	emb|CAAB02007260.1|:8044-8467
NuwaII-1_TRub	emb|CAAB02017725.1|:1724-2259
NuwaII-1_TRub	emb|CAAB02000199.1|:25889-26327
NuwaII-1_TRub	emb|CAAB02020843.1|:2106-2505
NuwaII-1_TRub	emb|CAAB02016148.1|:2733-3074
NuwaII-1_TRub	emb|CAAB02004203.1|:5239-5674
NuwaII-1_TRub	emb|CAAB02003764.1|:340-871
NuwaII-1_TRub	emb|CAAB02000713.1|:388-753
NuwaII-1_TRub	emb|CAAB02015983.1|:7695-8033
NuwaII-1_TRub	emb|CAAB02007315.1|:13966-14264
NuwaII-1_TRub	emb|CAAB02001137.1|:9066-9381
NuwaII-1_TRub	emb|CAAB02024024.1|:1-291
NuwaII-1_TRub	emb|CAAB02002287.1|:901-1218
NuwaII-1_TRub	emb|CAAB02000643.1|:8239-8665
NuwaII-1_TRub	emb|CAAB02003434.1|:2525-2853
NuwaII-1_TRub	emb|CAAB02007501.1|:63561-64115
NuwaII-1_TRub	emb|CAAB02002038.1|:39149-39504
NuwaII-1_TRub	emb|CAAB02000699.1|:83755-84023
NuwaII-1_TRub	emb|CAAB02011252.1|:21911-22292
NuwaII-1_TRub	emb|CAAB02012988.1|:2181-2440
NuwaII-1_TRub	emb|CAAB02009328.1|:87069-87402
NuwaII-1_TRub	emb|CAAB02014364.1|:43-290
NuwaII-1_TRub	emb|CAAB02016296.1|:403-650
NuwaII-1_TRub	emb|CAAB02004924.1|:53269-53552
NuwaII-1_TRub	emb|CAAB02009544.1|:28-302
NuwaII-1_TRub	emb|CAAB02026457.1|:994-1225
NuwaII-1_TRub	emb|CAAB02003727.1|:51109-51317
NuwaII-1_TRub	emb|CAAB02000883.1|:1399-1595
NuwaII-1_TRub	emb|CAAB02030585.1|:566-1508
NuwaII-1_TRub	emb|CAAB02003038.1|:14429-14611
NuwaII-1_TRub	emb|CAAB02016236.1|:38-226
NuwaII-1_TRub	emb|CAAB02018350.1|:1-174
NuwaII-1_TRub	emb|CAAB02006228.1|:1-1016
NuwaII-1_TRub	emb|CAAB02014363.1|:3350-3512
NuwaII-1_TRub	emb|CAAB02000700.1|:1-160
NuwaII-1_TRub	emb|CAAB02025471.1|:48-193
NuwaII-1_TRub	emb|CAAB02023403.1|:25-170
NuwaII-1_TRub	emb|CAAB02007455.1|:1-137
NuwaII-1_TRub	emb|CAAB02000712.1|:68414-68528
NuwaII-1_TRub	emb|CAAB02030416.1|:1672-1783
NuwaII-1_TRub	emb|CAAB02008985.1|:7997-8104
NuwaII-1_TRub	emb|CAAB02017079.1|:1-100

NuwaII-1_TRub	emb|CAAB02027978.1|:1-98
NuwaII-1_TRub	emb|CAAB02005814.1|:9968-10062
NuwaII-1_TRub	emb|CAAB02011932.1|:31670-31763
NuwaII-1_TRub	emb|CAAB02007146.1|:6583-6634
NuwaII-1_TRub	emb|CAAB02001533.1|:55466-58632
NuwaII-1_TRub	emb|CAAB02014720.1|:30405-36086
NuwaII-1_TRub	emb|CAAB02018384.1|:934-3208
NuwaII-1_TRub	emb|CAAB02008928.1|:7235-11607
NuwaII-1_TRub	emb|CAAB02009281.1|:12200-13681
NuwaII-1_TRub	emb|CAAB02013083.1|:12265-13697
NuwaII-1_TRub	emb|CAAB02007891.1|:947-2396
NuwaII-1_TRub	emb|CAAB02011524.1|:7977-11910
NuwaII-1_TRub	emb|CAAB02012593.1|:934-2546
NuwaII-1_TRub	emb|CAAB02010228.1|:4410-6216
NuwaII-1_TRub	emb|CAAB02004463.1|:7115-10210
NuwaII-1_TRub	emb|CAAB02026736.1|:337-1379
NuwaII-1_TRub	emb|CAAB02002649.1|:44085-44994
NuwaII-1_TRub	emb|CAAB02005639.1|:9075-9983
NuwaII-1_TRub	emb|CAAB02002962.1|:12974-13874
NuwaII-1_TRub	emb|CAAB02011352.1|:6860-7806
NuwaII-1_TRub	emb|CAAB02018644.1|:264-1167
NuwaII-1_TRub	emb|CAAB02000491.1|:9188-10081
NuwaII-1_TRub	emb|CAAB02009597.1|:8183-9076
NuwaII-1_TRub	emb|CAAB02012389.1|:744-2017
NuwaII-1_TRub	emb|CAAB02007925.1|:27058-28610
NuwaII-1_TRub	emb|CAAB02010637.1|:76086-76971
NuwaII-1_TRub	emb|CAAB02004459.1|:22646-23521
NuwaII-1_TRub	emb|CAAB02010804.1|:11997-12884
NuwaII-1_TRub	emb|CAAB02003370.1|:62872-63726
NuwaII-1_TRub	emb|CAAB02004144.1|:4082-4984
NuwaII-1_TRub	emb|CAAB02009188.1|:604-1500
NuwaII-1_TRub	emb|CAAB02012332.1|:5339-6253
NuwaII-1_TRub	emb|CAAB02007273.1|:14053-16166
NuwaII-1_TRub	emb|CAAB02003332.1|:5532-6409
NuwaII-1_TRub	emb|CAAB02009240.1|:76257-77146
NuwaII-1_TRub	emb|CAAB02010917.1|:1583-2474
NuwaII-1_TRub	emb|CAAB02005407.1|:2967-4080
NuwaII-1_TRub	emb|CAAB02007397.1|:15878-16712
NuwaII-1_TRub	emb|CAAB02013551.1|:10658-11529
NuwaII-1_TRub	emb|CAAB02011457.1|:37586-38486
NuwaII-1_TRub	emb|CAAB02019519.1|:1-902
NuwaII-1_TRub	emb|CAAB02022684.1|:2546-3483
NuwaII-1_TRub	emb|CAAB02009717.1|:22416-23353
NuwaII-1_TRub	emb|CAAB02002520.1|:123364-124207

NuwaII-1_TRub	emb|CAAB02016256.1|:4472-5358
NuwaII-1_TRub	emb|CAAB02006873.1|:28373-29341
NuwaII-1_TRub	emb|CAAB02010224.1|:9572-10396
NuwaII-1_TRub	emb|CAAB02005398.1|:4479-5344
NuwaII-1_TRub	emb|CAAB02003465.1|:27532-28452
NuwaII-1_TRub	emb|CAAB02002459.1|:16330-17134
NuwaII-1_TRub	emb|CAAB02014768.1|:652-1576
NuwaII-1_TRub	emb|CAAB02025619.1|:86-884
NuwaII-1_TRub	emb|CAAB02016320.1|:1630-2407
NuwaII-1_TRub	emb|CAAB02009381.1|:581-1323
NuwaII-1_TRub	emb|CAAB02020014.1|:2196-3759
NuwaII-1_TRub	emb|CAAB02011932.1|:31763-32560
NuwaII-1_TRub	emb|CAAB02012740.1|:2623-3338
NuwaII-1_TRub	emb|CAAB02004940.1|:32769-33472
NuwaII-1_TRub	emb|CAAB02013272.1|:12342-13161
NuwaII-1_TRub	emb|CAAB02011709.1|:42738-43492
NuwaII-1_TRub	emb|CAAB02021034.1|:15-697
NuwaII-1_TRub	emb|CAAB02027306.1|:1-671
NuwaII-1_TRub	emb|CAAB02023610.1|:216-776
NuwaII-1_TRub	emb|CAAB02009167.1|:6279-7069
NuwaII-1_TRub	emb|CAAB02000134.1|:48079-48767
NuwaII-1_TRub	emb|CAAB02006772.1|:37-679
NuwaII-1_TRub	emb|CAAB02009660.1|:30019-30718
NuwaII-1_TRub	emb|CAAB02006366.1|:12000-12781
NuwaII-1_TRub	emb|CAAB02016047.1|:340-1079
NuwaII-1_TRub	emb|CAAB02001555.1|:85279-86049
NuwaII-1_TRub	emb|CAAB02010098.1|:41925-42613
NuwaII-1_TRub	emb|CAAB02018645.1|:245-804
NuwaII-1_TRub	emb|CAAB02003507.1|:5681-6293
NuwaII-1_TRub	emb|CAAB02007796.1|:3811-4444
NuwaII-1_TRub	emb|CAAB02023559.1|:202-799
NuwaII-1_TRub	emb|CAAB02004895.1|:6625-7300
NuwaII-1_TRub	emb|CAAB02020635.1|:380-910
NuwaII-1_TRub	emb|CAAB02008398.1|:5501-6056
NuwaII-1_TRub	emb|CAAB02011730.1|:42020-42625
NuwaII-1_TRub	emb|CAAB02022206.1|:532-1064
NuwaII-1_TRub	emb|CAAB02010916.1|:1-533
NuwaII-1_TRub	emb|CAAB02016845.1|:530-2470
NuwaII-1_TRub	emb|CAAB02018039.1|:1-522
NuwaII-1_TRub	emb|CAAB02001459.1|:50681-51118
NuwaII-1_TRub	emb|CAAB02008926.1|:7657-8088
NuwaII-1_TRub	emb|CAAB02003401.1|:29437-29870
NuwaII-1_TRub	emb|CAAB02008986.1|:13726-14164
NuwaII-1_TRub	emb|CAAB02004175.1|:8-477

NuwaII-1_TRub	emb|CAAB02013570.1|:2798-3280
NuwaII-1_TRub	emb|CAAB02026057.1|:717-1507
NuwaII-1_TRub	emb|CAAB02008037.1|:25191-25783
NuwaII-1_TRub	emb|CAAB02018671.1|:78-703
NuwaII-1_TRub	emb|CAAB02006963.1|:9402-9815
NuwaII-1_TRub	emb|CAAB02007506.1|:9807-10220
NuwaII-1_TRub	emb|CAAB02005891.1|:7162-7580
NuwaII-1_TRub	emb|CAAB02010033.1|:3293-3822
NuwaII-1_TRub	emb|CAAB02020753.1|:1619-2062
NuwaII-1_TRub	emb|CAAB02024825.1|:370-784
NuwaII-1_TRub	emb|CAAB02015807.1|:8-411
NuwaII-1_TRub	emb|CAAB02005027.1|:24972-25326
NuwaII-1_TRub	emb|CAAB02020233.1|:1374-1740
NuwaII-1_TRub	emb|CAAB02030585.1|:128-550
NuwaII-1_TRub	emb|CAAB02030416.1|:1234-1656
NuwaII-1_TRub	emb|CAAB02012592.1|:488-808
NuwaII-1_TRub	emb|CAAB02010229.1|:710-1048
NuwaII-1_TRub	emb|CAAB02010612.1|:78634-78892
NuwaII-1_TRub	emb|CAAB02007149.1|:1680-1946
NuwaII-1_TRub	emb|CAAB02017290.1|:5-252
NuwaII-1_TRub	emb|CAAB02027028.1|:776-1029
NuwaII-1_TRub	emb|CAAB02007707.1|:14432-15228
NuwaII-1_TRub	emb|CAAB02016800.1|:5066-5305
NuwaII-1_TRub	emb|CAAB02011316.1|:38309-38605
NuwaII-1_TRub	emb|CAAB02004857.1|:14007-14229
NuwaII-1_TRub	emb|CAAB02027027.1|:680-911
NuwaII-1_TRub	emb|CAAB02004203.1|:18010-18234
NuwaII-1_TRub	emb|CAAB02012084.1|:12662-12866
NuwaII-1_TRub	emb|CAAB02009550.1|:11414-11618
NuwaII-1_TRub	emb|CAAB02030677.1|:331-542
NuwaII-1_TRub	emb|CAAB02018038.1|:1007-1184
NuwaII-1_TRub	emb|CAAB02004176.1|:1-175
NuwaII-1_TRub	emb|CAAB02007146.1|:15810-15959
NuwaII-1_TRub	emb|CAAB02017745.1|:682-823
NuwaII-1_TRub	emb|CAAB02008841.1|:1712-1856
NuwaII-1_TRub	emb|CAAB02015163.1|:378-505
NuwaII-1_TRub	emb|CAAB02013571.1|:1-103
NuwaII-1_TRub	emb|CAAB02006227.1|:107925-107985
